# Role of APOBEC3 in Genetic Diversity among Endogenous Murine Leukemia Viruses

**DOI:** 10.1371/journal.pgen.0030183

**Published:** 2007-10-26

**Authors:** Patric Jern, Jonathan P Stoye, John M Coffin

**Affiliations:** 1 Department of Molecular Biology and Microbiology, Tufts University School of Medicine, Boston, Massachusetts, United States of America; 2 Division of Virology, MRC National Institute for Medical Research, London, United Kingdom; The Jackson Laboratory, United States of America

## Abstract

The ability of human and murine APOBECs (specifically, APOBEC3) to inhibit infecting retroviruses and retrotransposition of some mobile elements is becoming established. Less clear is the effect that they have had on the establishment of the endogenous proviruses resident in the human and mouse genomes. We used the mouse genome sequence to study diversity and genetic traits of nonecotropic murine leukemia viruses (polytropic [Pmv], modified polytropic [Mpmv], and xenotropic [Xmv] subgroups), the best-characterized large set of recently integrated proviruses. We identified 49 proviruses. In phylogenetic analyses, Pmvs and Mpmvs were monophyletic, whereas Xmvs were divided into several clades, implying a greater number of replication cycles between the integration events. Four distinct primer binding site types (Pro, Gln1, Gln2 and Thr) were dispersed within the phylogeny, indicating frequent mispriming. We analyzed the frequency and context of G-to-A mutations for the role of mA3 in formation of these proviruses. In the Pmv and Mpmv (but not Xmv) groups, mutations attributable to mA3 constituted a large fraction of the total. A significant number of nonsense mutations suggests the absence of purifying selection following mutation. A strong bias of G-to-A relative to C-to-T changes was seen, implying a strand specificity that can only have occurred prior to integration. The optimal sequence context of G-to-A mutations, TTC, was consistent with mA3. At least in the Pmv group, a significant 5′ to 3′ gradient of G-to-A mutations was consistent with mA3 editing. Altogether, our results for the first time suggest mA3 editing immediately preceding the integration event that led to retroviral endogenization, contributing to inactivation of infectivity.

## Author Summary

Vertebrate genomes are littered with remnants from earlier retroviral infections, in the form of endogenous retroviruses (ERVs). Cellular host defenses against retroviruses, including the APOBEC3 family of cytidine deaminases, have been described previously. APOBEC3 proteins have been shown to edit some retroviruses and other retrotransposing elements during their replication by deamination of C to U during negative-strand synthesis, resulting in G-to-A mutations in the sense strand. Here, we studied the possible effects that the APOBEC-protein family might have had in the establishing ERVs. We identified 49 endogenous (nonecotropic) murine leukemia viruses, divided into three groups; polytropic, modified polytropic, and xenotropic, in the sequenced C57BL/6J mouse genome. We analyzed genetic variation within and among subgroups and found mutation patterns consistent with APOBEC3 editing of Pmv and Mpmv, but not Xmv proviruses. Evidence such as (i) significantly higher G-to-A mutation frequencies compared to controls and large fractions leading to inactivating stop mutations, (ii) optimal sequence contexts surrounding the mutation positions, and (iii) editing gradient following the time course of retroviral replication, implicate APOBEC3 as a factor contributing to inactivation of these ERVs in the mouse genome.

## Introduction

Retroviruses that integrate into the germ line may be inherited vertically as endogenous retroviral sequences (ERVs) [1]. A considerable fraction of mammalian genomes consists of ERVs [2–4], most with numerous inactivating mutations, thus presenting the only known viral “fossil” record. One of the recently discovered cellular defense mechanisms against retroviral propagation involves APOBEC3 (A3)-induced C-to-U deamination in negative-strand retroviral DNA during reverse transcription [5], resulting in a G-to-A hypermutated provirus [6–9].

The role of human and murine A3 (hA3 and mA3, respectively) family members in inhibiting infection by exogenous retroviruses and retrotransposition of some mobile elements is becoming well established [10–12]. Less clear is the possible effect that these restriction factors may have had on the establishment of the many thousands of endogenous proviruses present in vertebrate genomes [13]. Although A3-induced G-to-A mutations can be readily detected in experimental infection, such mutations are difficult to discern in elements that have had long residence in the germline and that have suffered considerable post-integration mutagenesis.

The mouse genome harbors a diversity of endogenous (nonecotropic) murine leukemia viruses (MLVs), which form the best-characterized large set of recently integrated proviruses, as indicated by their insertional polymorphism among inbred mouse strains [14], and by the presence of some infectious members [15,16]. The group can be subdivided into the polytropic (Pmv), modified polytropic (Mpmv), and the xenotropic (Xmv) proviruses [17]. Each common inbred mouse strain contains about 20 proviruses of each type, and shares about half of them with any other inbred strain [18]. Although several infectious Xmv loci, including Bxv1 (Xmv43), have been described [15,18–20], and functional Pmv and Mpmv *env* genes can be rescued by recombination [17,21–23], no infectious Pmv or Mpmv has yet been detected.

Here, we have taken advantage of the well-characterized endogenous nonecotropic MLVs as an appropriate model for studying recent evolution of the host–virus interaction, in an attempt to demonstrate probable events associated with endogenization. Genetic studies [18] have revealed 54 nonecotropic proviruses in C57BL/6J mice; we have now identified 49 of these proviruses within the genome sequence of these mice (http://genome.ucsc.edu/). We analyzed genetic variation within and among subgroups and found mutation patterns consistent with mA3 editing of Pmv and Mpmv DNA, but not Xmv DNA, as a plausible factor contributing to inactivation of these ERVs in the mouse genome.

## Materials and Methods

### Data Collection

We mined the C57BL/6J genome sequence for sequences of proviruses we had previously identified using a restriction mapping strategy [18]. We used the sequences of MLV *env* probes JS-4, JS-5, and JS-6 [14] in BLAST searches (http://www.ensembl.org) and BLAT searches (http://genome.ucsc.edu/). Based on predicted reactivity with the specific probes, predicted restriction fragment size, and other features, we were able to identify 49 (23 Pmv, 13 Mpmv, and 13 Xmv) of the known 54 non-Y-linked nonecotropic proviruses in this strain (Table S1; Figure S1) [18]. Sequences encoding viral proteins were verified using RetroTector as described in earlier papers [13,24]. Automated PERL scripts were used to verify integration sites and proviral orientation and to extract target site duplications from the C57BL/6J genome version mm8 freeze date Feb. 2006 (Figure S2). *gag, pol* and *env* genes were concatenated and aligned using ClustalX [25] followed by manual tuning to reconstruct open reading frames (ORFs) for each provirus compared to alignment majority rule consensus sequences (Figure S3). Stop codons were mainly caused by G-to-A mutations, which we altered to maintain a nonsynonymous substitution for PAML analyses [26] (see below). Additionally, to extend the analysis outside coding genes and retrieve as many detectable mutations as possible, full provirus nucleotide sequences were aligned using BLASTalign [27], with no additional attention to ORFs, and consensus provirus sequences were constructed.

### Phylogenetic Analyses

Maximum parsimony, maximum likelihood, and Bayesian methods, using MEGA3 [28], PHYML [29] and MrBayes [30], were utilized for different steps and confirmations of phylogenetic reconstructions. A maximum likelihood phylogeny was reconstructed for the codon and ORF adjusted internal regions (*gag*, *pol*, and *env*) of the nonecotropic MLVs and reference sequences (MoMLV, MLV-ecotropic, and HuXmv) using PHYML, with the HKY + γ model (parameter values estimated from dataset). Nonsynonymous versus synonymous substitution ratios (*d*_N_/*d*_S_) were calculated for the branches in the maximum likelihood tree by using PAML [26]. A single *d*_N_/*d*_S_ ratio for the subtrees (one-ratio model) and separate estimated values for the inner and outer branches (two-ratio model) were estimated for each subgroup. Significance of the differences between the two models was evaluated by likelihood ratio tests, by comparing twice the difference of log likelihoods of subtrees to the χ^2^ distribution with 1 degree of freedom [31]. We tested the internal branches for deviation from neutrality by fixing their *d*_N_/*d*_S_ to 1 and comparing the difference by using the likelihood ratio test.

### Mutation Analyses

Mutations of aligned sequences compared to each respective group consensus were collected for *gag*, *pol*, and *env* using automated PERL scripts*.* Codons from each provirus alignment position including at least one G-to-A mutation compared to respective subgroup consensus sequence were recorded for each gene. Codons were aligned and analyzed for synonymous- and nonsynonymous mutations. For each gene, we also analyzed which codon positions had G-to-A mutations and if stop codons were introduced by the mutations.

### Simulation of Mutation Gradients

Using automated PERL scripts, we tested if G-to-A mutations followed a distribution suggestive of A3 editing correlating with the persistence of (−) single-stranded DNA during reverse transcription [32–34]. Alignment positions between the primer binding site (PBS) and polypurine tract in the full provirus alignments (without additional attention to ORFs, see above) of each subgroup were collected and divided into ten equally large bins varying slightly in size among subgroups due to different alignment lengths. Thereafter, the fraction of G-to-A mutations divided by the number of consensus sequence G positions was recorded for each bin. A similar analysis was conducted for C-to-T mutations. The sum of all G-to-A mutations for each subgroup was calculated and used for simulation of theoretical G-to-A mutations at possible sites (G nucleotide positions) in the consensus sequences. We applied two probability models: (i) An equal random probability model for a G-to-A mutation to occur for every alignment consensus G nucleotide; and (ii) A triangular skewed random probability model with a minimum probability for G-to-A mutations at consensus G nucleotides at the 5′-end of the genome and a maximum at the 3′ end. Fractions of G-to-A mutations in simulations were calculated as for the observed data above. Likewise, simulations were conducted for C-to-T mutations.

## Results

### Nonecotropic MLV Subgroups

**Figure 1:**
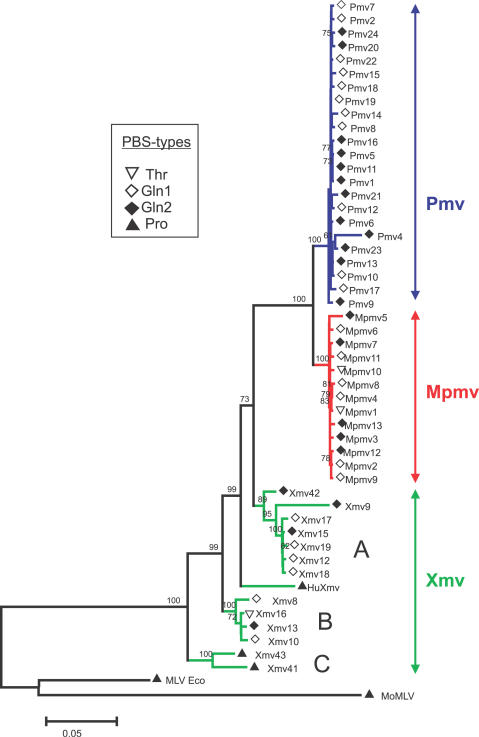
Phylogenetic Reconstruction of Nonecotropic MLV Proviruses in the C57BL/6J Genome A maximum likelihood analysis of codon adjusted internal region ORFs (*gag*, *pol*, and *env*) of nonecotropic MLVs and related reference sequences is shown. Bootstrap supports >60% are shown next to branch nodes. The Pmv and Mpmv proviruses are monophyletic and group separately from each other, while the Xmv proviruses form three clades, marked A, B, and C. The ecotropic endogenous provirus Emv 2 (MLV Eco), and the exogenous virus MoMLV are also included as outgroups, as is the Xmv-like retrovirus (HuXmv) recently described in human prostate cancer [50]. tRNA primer types inferred from the PBS sequences are noted by the symbols next to sequence names.

Because of their relatively large copy number, relatively recent insertion into the host germline, and thorough genetic characterization, we took advantage of the nonecotropic MLVs [14,16,18] to examine events surrounding endogenization of these elements in the mouse genome. Of particular interest is the apparent absence, as judged by the absence of reports to the contrary, of infectious virus from two of the three subgroups (Pmv and Mpmv). BLAST searches (http://www.ensembl.org) using MLV *env* probes JS-4, JS-5, and JS-6 [14], and BLAT searches (http://genome.ucsc.edu/) led to identification of sequences of 49 nonecotropic proviruses (23 Pmv, 13 Mpmv, and 13 Xmv) of the 54 known to be present in this strain of mouse (Table S1; Figures S1 and S2) [18]. To examine the relationship of the three subgroups, we performed maximum likelihood phylogenetic analyses on the manually adjusted *gag*, *pol*, and *env* regions, as well as on internal regions from three reference sequences (Figure 1). The Pmv and Mpmv subgroups were monophyletic, whereas the Xmv sequences were not, and could themselves be divided into three well-supported clades, with branch lengths implying larger numbers of viral replication cycles between the integration events than in the other two groups (Figure 1). The tree also implies that the most recent common ancestor of all the proviruses was xenotropic.

### Endogenous Nonecotropic MLV Proliferation

To investigate differences in proliferation and possible purifying selection between the subgroups, we analyzed the nonsynonymous-to-synonymous substitution ratios (*d*_N_/*d*_S_) for the branches of subtrees derived from the maximum likelihood tree (Figure 1). We tested two models: (i) A one-ratio model with the same *d*_N_/*d*_S_ calculated for each branch, and (ii) A two-ratio model distinguishing internal branches from terminal branches. A *d*_N_/*d*_S_ ratio less than 1 implies purifying selection and would normally be expected in a group of retroviruses that is actively replicating. When a provirus becomes immobilized in the genome and is no longer subject to purifying selection, it adapts a neutral mutation rate (*d*_N_/*d*_S_ = 1). These properties are expected to result in phylogenetic reconstruction for endogenous retroviruses where internal branches show lower *d*_N_/*d*_S_ ratios than terminal branches [35]. The difference between the two models can then be estimated by a likelihood ratio test; i.e., twice the difference in likelihood of the two different trees, compared to the χ^2^ distribution with 1 degree of freedom [31]. With a caveat for small sequence differences (Figure 1) and thus low analysis power, we found that the Pmv and Mpmv proviruses showed high *d*_N_/*d*_S_ ratios, not significantly different from 1 with no significant difference between the two models (Table 1), a result that may be attributable to the small intragroup differences observed in short branch lengths and low bootstrap supports in the maximum likelihood tree (Figure 1). However, the Xmv proviruses, taken as a whole, had *d*_N_/*d*_S_ significantly below 1, with significantly lower values for the internal branches (Table 1), indicative of purifying selection and, therefore, more cycles of active proliferation in both internal and terminal branches compared to the other two subgroups.

**Table 1:**
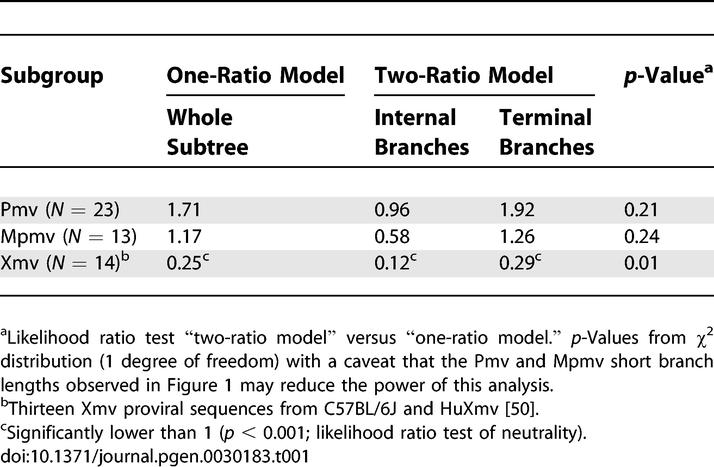
*d*_N_/*d*_S_ Ratios in *gag*, *pol*, and *env* Regions

### Different PBS Sequences

MLV PBSs have previously been reported to vary in sequence, implying use of both Pro and Gln1 tRNAs as primers for reverse transcription [36,37]. Analysis of the endogenous nonecotropic MLV dataset showed a mix of PBSs corresponding to four types of tRNA (Pro, Gln1, Gln2, and Thr) dispersed within the maximum likelihood tree, indicating changes probably resulting from mispriming during reverse transcription (Figure 1; Table S2). Although an exact pattern of PBS replacement could not be inferred, the presence of common PBS types in the three different provirus types separated with moderate to high bootstrap supports implies that such mispriming must have been a frequent event (Figure 1).

### Analysis of G-to-A Mutations

From the codon-adjusted *gag*, *pol*, and *env* alignments, all proviruses were analyzed for mutations relative to the consensus for their group. The Xmvs differed on average by 1.8% (0.3%–5.9%) from the group consensus (estimated from the alignment used for Figure 1 and extreme values excluded), compared to differences of 0.18% (0%–0.5%) and 0.21% (0.1%–0.3%) for Pmv and Mpmv from their respective consensuses. Between the groups, consensus sequences differed by 2.3% comparing Pmv to Mpmv, and 5.2% and 5.1%, respectively, comparing either to Xmv.

**Figure 2:**
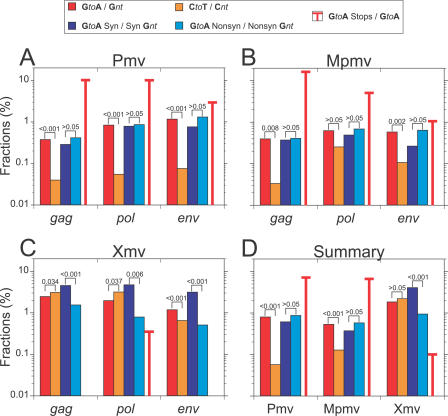
Mutation Dstribution within Endogenous Nonecotropic MLV Genes G-to-A mutation frequencies relative to total G nucleotides in the consensus sequence of each subgroup are compared to C-to-T mutations relative to total C nucleotides in the consensus sequence of each subgroup, as are frequencies of G-to-A mutations leading to synonymous and nonsynonymous changes relative to possible consensus G nucleotides, and to fractions of all G-to-A mutations that introduce stop codons in the coding region of (A) Pmv, (B) Mpmv, and (C) Xmv. (D) shows the genome totals for each group. Significance levels for the comparisons shown by brackets were calculated using the Wilcoxon matched-pairs signed-ranks test, a nonparametric alternative to the more commonly used paired Student's *t*-test, favored for normally distributed data.

The two proviruses excluded from the consensus analysis (Pmv4 and Mpmv5, Figure 1) exhibited a high frequency of G-to-A mutations compared to C-to-T mutations (123 versus two and 63 versus five, respectively; see below). In other retroviruses, such mutations are associated with the activity of cytosine deaminases, including human hA3G and hA3F, and mouse mA3, on minus-strand DNA during reverse transcription [6,9,10,38]. We therefore performed a more detailed analysis of the mutation spectrum in all proviruses. Sites with G nucleotides in the consensus sequence and A in any provirus sequence were collected and analyzed. To control for mutations occurring after reverse transcription and integration, the same procedure was conducted for C-to-T mutation sites. A significant bias of G-to-A relative to C-to-T changes was seen in both Pmv and Mpmv proviruses (Figure 2D), implying a strand specificity that can only have occurred prior to integration. Within these two groups, G-to-A mutations constituted a large fraction of total mutations with no preference for codon position in any of the genes (unpublished data), and a significant fraction of these mutations led to introduction of stop codons and nonsynonymous changes in all genes (Figure 2), implying an absence of purifying selection following mutation consistent with the *d*_N_/*d*_S_ ratios from the maximum likelihood tree (Table 1), with the caveat that the sequence differences in the Pmv and Mpmv subgroups were small (Figure 1), resulting in somewhat low analysis power. In fact, all but one of the nonsense mutations in these proviruses were the result of G-to-A mutations, and the high *d*_N_/*d*_S_ ratios for Pmv and Mpmv (Table 1) could be attributed entirely to the nonsynonymous G-to-A mutations (Figure 2A and 2B).

By contrast, the Xmv proviruses did not display the same clear mutational pattern as Pmv and Mpmv. Although the Xmvs had higher intragroup sequence diversity (Figure 1), which could have masked some G-to-A mutational bias, the predominance of purifying selection (Table 1), the lack of significant bias for G-to-A as compared to C-to-T mutations, and an almost complete lack of stop codons in all genes (Figure 2C), implies that the Xmvs were less subject to editing than the other two groups. Analysis of the three Xmv clades separately also confirms the lack of an excess of G-to-A over C-to-T mutations (unpublished data).

### Mutational Target Site Preferences

**Figure 3:**
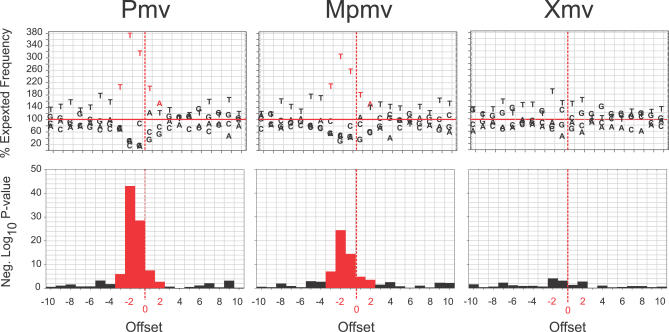
Preference Sequences for G-to-A Mutations Upper panels: (−) DNA nucleotide frequencies are plotted relative to expected frequencies for each of ten positions up- and downstream of the putative mA3 deamination targets for each group of proviruses. Lower panels: Significance (negative Log_10_ *p*-value, χ^2^ tests with 3 degrees of freedom) of the deviations from expected frequencies is plotted for each position.

To determine whether there was a preferred sequence context for the G-to-A mutations observed, alignments of observed nucleotide frequencies relative to expected nucleotide frequencies, ten nucleotides up- and downstream of the putative mA3 target C nucleotide in the viral (−) strand DNA were plotted (Figure 3). This analysis revealed a highly significant optimal sequence context for dC deamination, identical in both Pmv and Mpmv. This sequence, TTC, was consistent with the preferred sequence for mA3 [39], but differed slightly from the TCC (however, possibly also TTC) reported earlier for mA3 [9]. The sequence context may be extended somewhat (to TTTCTW) by combining the Pmv and Mpmv results (Figure 3). No significant consensus was seen in the Xmv subgroup (Figure 3), again consistent with a lack of effect of mA3 on these proviruses.

### Analysis and Simulation of APOBEC3-Mediated G-to-A Mutations

**Figure 4:**
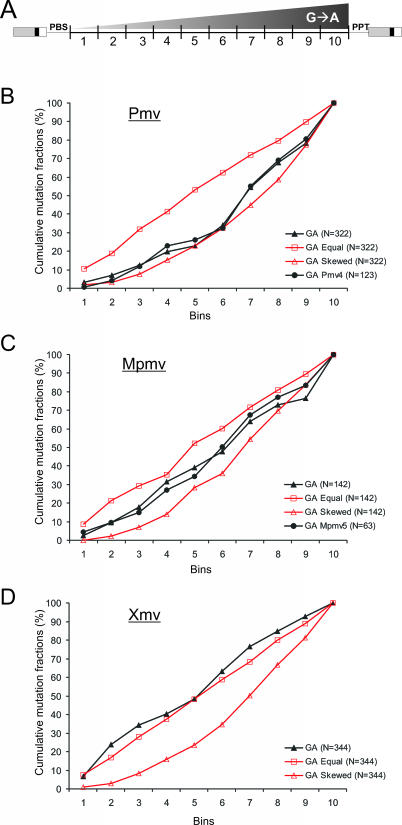
Gradients of G-to-A Mutations in Endogenous Nonecotropic MLVs (A) The provirus sequences between the primer sites from the BLAST alignment of each subgroup were divided into ten equal bins and fractions of observed G-to-A mutations relative to the number of G nucleotides within consensus sequences were pooled for each bin and plotted cumulatively 5′ to 3′ for (B) Pmv, (C) Mpmv, and (D) Xmv. Plots were normalized for direct comparison. The total numbers of mutations analyzed for each plot are presented next to the names in each legend. The same numbers of mutations were used in the two simulation models (equal and skewed random models) and plotted (open symbols) next to the observed data for each subgroup.

The preference of A3G editing for single-stranded DNA and the mechanism of reverse transcription lead to a gradient of mutation frequency in proviral DNA, increasing from 5′ to 3′ between the sites of priming [32,33]. To determine whether such a gradient could also be observed in the nonecotropic proviruses, we divided the genomes of each subgroup into ten equally large bins and plotted the 5′ to 3′ cumulative fraction of each G-to-A mutation relative to the consensus sequence (Figure 4). For comparison purposes, we performed simulations based on the total number of mutations (Figure 4, open symbols). The two simulation models plotted were based on: (i) equal random probability for a G-to-A mutation to occur for every alignment consensus G nucleotide, and (ii) a triangular skewed random probability model with a minimum probability for G-to-A mutations at the 5′ end and a maximum at the 3′ end. All plots were normalized for direct comparisons. Thus, the cumulative and normalized equal random simulation plot is linear and the cumulative normalized skewed random simulation plot would be expected to follow a power function. In the case of Pmv, the plot of G-to-A mutations was not distinguishable from the skewed distribution (*p* = 0.83, χ^2^ test) and was significantly different from the equal random distribution (*p =* 0.01, Figure 4B), whereas the distribution of C-to-T mutations followed an equal random distribution (unpublished data). This result suggests that G-to-A mutations were introduced at a rate corresponding to the persistence of (−) strand DNA during reverse transcription, in accordance with previous studies on lentiviruses [32–34]. The pattern with the Mpmvs (Figure 4C), although also suggestive of a gradient of mA3 activity, was much less clear, due to a lower overall frequency of G-to-A changes, and a higher frequency of background mutations. Again, no evidence for A3 activity could be seen with the Xmvs (Figure 4D).

### Highly Edited Nonecotropic Proviruses

**Figure 5:**
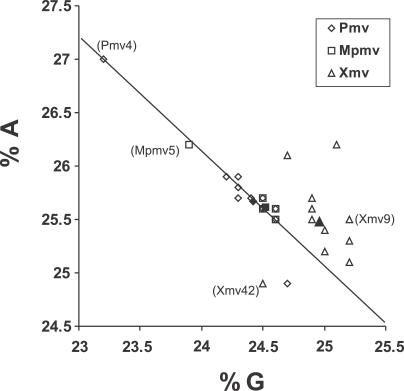
G and A Nucleotide Compositions of Nonecotropic Proviruses The frequency of A is plotted against the frequency of G in the indicated proviruses. Filled symbols represent consensuses for each group. One Pmv and one Mpmv provirus, indicated with names, were “hypermutated.” Two Xmv proviruses with long branch lengths (Figure 1) that do not show G-to-A hypermutation are also marked.

We further explored mA3 editing by comparing the total nucleotide compositions of the nonecotropic proviruses (Figure 5). For both Pmv and Mpmv subgroups, an increase of A was correlated with a depletion of G nucleotides (Figure 5, *R* ^2^ = 0.90). In each these two subgroups, one provirus exhibited a much greater degree of G-to-A mutations than the others. When compared to their respective subgroup consensus sequences, the Pmv4 provirus had more than 60 times as many G-to-A as C-to-T mutations (123 versus two), and the Mpmv5 provirus had about 12 times as many G-to-A mutations compared to C-to-T mutations (63 versus 5), resulting in the skewed G and A nucleotide distributions observed in Figure 5. Furthermore, the distribution of mutations across the Pmv4 genome followed the skewed random pattern (Figure 4B). We conclude that this provirus had suffered a higher deamination rate during reverse transcription [32,33]. We could not conclude the same distribution for Mpmv5 due to higher background of mutations. Since Pmv4 and Mpmv5 each contributed roughly 40% of the observed G-to-A mutations to the totals for their respective subgroup, we were concerned that they might have biased the results for each subgroup as a whole. To examine the extent of the contribution from these two “hypermutated” proviruses in the mA3 target site analysis (Figure 3), all analyses were repeated after their removal. Clear signs of mA3 editing could still be observed for the remaining Pmv and Mpmv proviruses (Figures S4–S7). Two Xmv proviruses—9 and 42—also exhibited much longer terminal branch lengths than others in the same clade (Figure 1). In the case of Xmv42, there were clear signs of recombination with a provirus of the Pmv group [40]. Closer examination (Figure 5) reveals that the increased branch length was not associated with G-to-A hypermutation in either of these two proviruses.

## Discussion

Endogenous proviruses constitute a large fraction of the genomes of well-characterized animal species, and also contribute in important ways to the phenotypes of these organisms. In this study, we used the best-characterized dataset of recently integrated endogenous retroviruses [14,16,18] to study probable factors involved in their endogenization, genetic variation, and replicative silencing.

For this purpose, we searched the C57BL/6J mouse genome sequence (http://genome.ucsc.edu/) to extract the sequences corresponding to the previously described nonecotropic (Pmv, Mpmv, and Xmv) proviruses of inbred mice [18]. These closely related proviruses were initially grouped by their reactivity with oligonucleotide probes corresponding to a highly variable region in *env* and individual proviruses were identified by the size of provirus–host junction restriction fragments. They were localized on the mouse genome using classical genetic mapping techniques [14,41]. Using an analogous in silico approach, we were able to positively identify only 49 of the 54 known proviruses in the C57BL/6J strain. For several reasons, we believe that the discrepancy is the result of errors in the reported sequence, not of errors in initial identification of the proviruses or substrain differences.

The five missing proviruses—Pmv3 (Chromosome 12), Xmv6 (Chromosome 6), Xmv14 and Xmv44 (both Chromosome 4), and Xmv45 (Chromosome 5)—are all present in more than one mouse strain; all loci are inherited within the AXB, BXA, BXH, and BXD collections of recombinant inbred mice confirming their presence in the C57BL/6J substrain [18]. Flanking sequences from Xmv14 and Xmv44 have been determined and their genetic linkage to one another (as well as Xmv 8 and 9) on distal mouse Chromosome 4 was confirmed in a large genetic cross [42]. BLAST analyses with these flanking sequences demonstrate the presence of sequences corresponding to these provirus host junctions within the pool of sequences assembled to generate the whole mouse genome sequence. However, they are present only on very small contigs that have not been assigned within the whole assembly. Moreover, the flanking sequences in Xmv44 also yield multiple high similarity hits within a cluster of zinc finger repeat genes present on mouse Chromosome 4. The Xmv14 flank also yields a multitude of high similarity hits but with different genomic regions. We speculate that the absence of Xmv14 and 44, and by extension the other missing proviral loci, results from difficulties in assembling final genome sequence in regions containing lengthy repeats.

Phylogenetic analysis of the complete coding regions of the nonecotropic proviruses revealed a grouping into clades partially consistent with that inferred from the use of a single probe. The Pmv and Mpmv proviruses each form a single well-supported clade, and share a common ancestor relative to the Xmvs. The Xmvs, by contrast, form three well-supported clades, one of which appears to have given rise to the other two groups. Thus, the common ancestor for the whole group was most likely an Xmv-like provirus, possibly with some additional recombination involving the *env* genes (our unpublished data) and there appear to have been considerably more cycles of viral replication separating the Xmv proviruses than the other two groups, a conclusion supported by their greater diversity and relatively low *d*_N_/*d*_S_ ratios, particularly in the internal branches of the phylogenetic tree. The low *d*_N_/*d*_S_ ratios in the terminal branches of the Xmv proviruses imply that these branches represent both repeated cycles of virus replication as well as events proximal to and following integration of each individual provirus. Of the three groups, only the Xmvs have been seen to give rise to infectious virus, although functional Pmv and Mpmv *env* genes have been recovered in polytropic viruses derived by recombination with ecotropic MLV [22]. Examination of the sequences of the recovered proviruses implies that, at least in part, this difference is due to much higher rates of nonsynonymous mutation in the latter groups: more than half (20/35) of the undeleted Pmv and Mpmv proviruses have one or more G-to-A mutations leading to stop codons, while only one of eight undeleted Xmv proviruses has been so affected (Figure 2; Table S1). Of the remaining Pmvs and Mpmvs, all have suffered G-to-A mutations relative to the likely ancestor, (an average of seven and five nonsynonymous changes per provirus, respectively). While the effects of each of these mutations on the function of the virus genes is unknown, it is likely that the net effect is to reduce or eliminate the ability of most or all of these proviruses to yield replication-competent virus.

The high frequencies of G-to-A changes in the Pmv and Mpmv groups led us to consider a possible role for mA3-mediated deamination in the generation of genetic diversity among the proviruses. Previous studies of A3 editing have been done mainly in lentiviruses [6,9,43], and mostly with hA3 (for a recent review see Holmes et al. [44]). mA3 has been shown to restrict retrotransposition of endogenous MusD and IAP mobile elements in mouse cells in culture, although there is less evidence for its action on the corresponding endogenous proviruses [11]. mA3 activity has also recently been shown to partially restrict infection with mouse mammary tumor virus [12]. Thus, there is evidence for mA3 editing of murine betaretrovirus-like elements.

In the present study, we observed mutation patterns indicative of mA3 editing in some gammaretroviruses, as well; specifically, the nonecotropic Pmv and Mpmv subgroups. Several lines of evidence support the conclusion that a large fraction of the mutations that distinguish the individual proviruses from their consensus were caused by mA3 editing. First, the high ratios (9:1 and 3:1) of G-to-A relative to C-to-T changes and the absence of purifying selection subsequent to mutation imply that most of the mutations arose during the last cycle of reverse transcription prior to integration of each provirus, and that, like human and mouse A3, deamination was specific for single-stranded DNA. Second, the inferred consensus sequence for C deamination (on the minus strand), TTC, is identical to that observed for mA3 in more direct experiments [39]. Third, at least in the Pmv group, there is a clear 5′–3′ gradient of G-to-A (but not C-to-T) mutations across the provirus. As has been pointed out before [32], such a gradient reflects the facts that A3 can only deaminate single-stranded DNA, and that minus-strand DNA near the 3′ end of the genome remains single stranded for a longer time than 5′ DNA during reverse transcription. We should note that, although we consider mA3 to be the most likely mediator of the G-to-A mutations observed, we cannot exclude participation of other cytidine deaminases, such as APOBEC1 [45] in these modifications. Experiments to examine the expression of the various APOBECs in germ line cells may help to resolve this issue.

The Xmv proviruses, with at least one infectious member, exhibit none of the mutational characteristics suggestive of mA3 editing and have evolved differently from Pmv and Mpmv (Figure 1; Table 1). Indeed, in contrast to the other two groups, Xmv proviruses exhibit a significantly higher ratio of C-to-T relative to G-to-A changes (Figure 2), possibly reflecting effects of purifying selection during their replication as viruses. This difference is not due to masking of G-to-A mutation by the higher overall diversity in the Xmv group. Thus, it appears that either (i) the xenotropic MLVs evolved a function to block the activity of mA3, perhaps by exclusion from virions, or (ii) the Pmv and Mpmv have lost this function. Given that the Xmv proviruses represent the ancestral group, the latter possibility seems much more likely. Loss of such a function might also provide a partial explanation for previous failures to isolate infectious Pmvs/Mpmvs from mouse cells by coculture despite the presence of multiple ERVs with a full complement of ORFs. A third possibility, given the complex origin of inbred mice and of their coevolution with murine retroviruses [1], is that germline integration of the Pmv and Mpmv proviruses occurred in a subspecies that expressed mA3 in the germline, while the host for the Xmvs did not. We are initiating studies to examine these possibilities, as well as the possibility that deaminase independent effects [46] might also have played a role in endogenous provirus formation.

In other retroviral genera, evasion of A3 activity is related to the ability of the virus to prevent incorporation of A3 into virions. For example, at least some lentiviruses and spumaviruses encode proteins (Vif and Bet) for this purpose, and deltaretroviruses, such as HTLV-1, use a C-terminal extension of NC to prevent interaction of APOBEC and RNA [47]. Variation of mA3 packaging into MLV virions has been proposed as a probable cause of observed variation in editing [43], but these results are controversial, since other studies showed no inhibition of MLV by mA3 [9,48,49]. This effect has been attributed to both its exclusion from the virion and proteolytic processing of the APOBEC that does get incorporated. The evasion of A3 deamination by MLV is specific for mA3, since MLV is not resistant to hA3G [48,49], analogous to the sensitivity of HIV to mA3 [9].

In an attempt to identify differences that might contribute to the variation of mA3 editing among the provirus groups, we parsed the NC region of the alignment used to construct the maximum likelihood tree (Figure 1). Variable positions in Gag, particularly in NC, are being evaluated for possible roles in preventing mA3 activity.

In summary, to our knowledge, we have shown here for the first time mA3 editing immediately preceding the integration event of endogenous gammaretroviruses. This activity is likely to have contributed to the inactivation of infectivity of two of the three nonecotropic MLV subgroups.

## Supporting Information

Figure S1 Genomic Nonecotropic MLV Provirus Sequences (FASTA Format) (215 KB PDF)

Figure S2 Nonecotropic Endogenous MLV Integration Junctions and Target Site Duplications (14 KB PDF)

Figure S3 ORF Adjusted Concatenated *gag-pol-env* Codon Alignments for Pmv, Mpmv, and Xmv with Highlighted Nucleotide Differences to Consensus of Their Subgroup (142 KB PDF)

Figure S4 Phylogenetic (ML Tree) Reconstruction of Nonecotropic MLV Proviruses in the C57BL/6J Genome, with Highly Edited Pmv4 and Mpmv5 Excluded (19 KB PDF)

Figure S5 Mutation Distribution within Endogenous Nonecotropic MLV Genes, with Highly Edited Pmv4 and Mpmv5 Excluded (20 KB PDF)

Figure S6 Preference Sequences for G-to-A Mutations with Highly Edited Pmv4 and Mpmv5 Excluded (22 KB PDF)

Figure S7 Gradients of G-to-A Mutations in Endogenous Nonecotropic MLVs, with Highly Edited Pmv4 and Mpmv5 Excluded (21 KB PDF)

Table S1 Nonecotropic Endogenous MLVs (12 KB PDF)

Table S2 PBS Types among Nonecotropic MLVs (7 KB PDF)

### Accession Numbers

The National Center for Biotechnology Information (NCBI) Entrez database (http://www.ncbi.nlm.nih.gov/sites/gquery?itool=toolbar) for the reference sequences discussed in this paper are MoMLV, NC_001501; MLV-Ecotropic, DQ366147; and HuXmv, EF185282.

## Abbreviations

A3: APOBEC3
ERV: endogenous retrovirus
hA3: human APOBEC3
mA3: murine APOBEC3
MLV: murine leukemia virus
Mpmv: modified polytropic MLV
ORF: open reading frame
PBS: primer binding site
Pmv: polytropic MLV
Xmv: xenotropic MLV
