# Supplementary material for: Role of APOBEC3 in Genetic Diversity among Endogenous Murine Leukemia Viruses
Source: PLoS Genet. 2007 Oct 26;3(10):e183. doi: 10.1371/journal.pgen.0030183 (PMC2041998; doi:10.1371/journal.pgen.0030183)
Supplement: Figure S1 — (215 KB PDF) [file pgen.0030183.sg001.pdf]

>Mpmv1
TGAAAGACCCCACCATAAGGCTTAGCAAGCTAGCTGCAGTAACGCCATTTTGCAAGGCATGAAAAAGTACCAGAGCTGGG
TTCTCAAAAGTTACAAGGAAGTTCAGTTATAGATTAACAGTTAAAGATCAAGGCTGAATAGCACTGGGACAGGGGCCGAA
CAGGATATCGGTGGTCAAGCACCTGGGCCCCGGCTCAGGGCCAAGAACAGATGGTTCTCAGATAAAGCGGAACCAGCAAC
AGACACAGAAGCCCCGATAGACGTCAGTGTTAGCAGAACTAGCTTCACTGATTTAGAAAAATAGAGGTGCACAGTGCTCT
GGTCACTCCTTGAACCTGTGTGTCTGCCAATGTTCTGACCAGATATGTGCCCATTGCTGAACCTTCATTAGACTCTTTCC
TTGTACCCCTCCCCTACCCATTTCTTGAAAATAGACATTGTTTAGATCTAAAAAGTCCCACCTCAGTTTCCCCAAATGAC
CGGGAAGTACCCCAAACCTTATTCGAACTAACCAACCAGCTCGCTTCTCGCTTCTGTAACCGCGCTTTTTGCTCCCCAGC
CCTAGCCCTATAAAAAGGGTAAAAACTCCACACTCGGCGCGCCAGTCCTCCGATAGACTGAGTCGCCCGGGTACCCGTGT
TCCCAATAAAGCCTCTTGCTGTTTACATCCGAATCGTGGACTCGCTGATCCTTGGGAGGGTCTCCTCAGATTGATTGACT
GCCCACCTGGGGGGTCTTTCATTTGGAGGCCCCAGCGAGATCAGGAGACCCCTGCCCAGGGACCACCGACCCCCGCCGGG
AGGTAAGCTGGCCAGCGGTCGTTTTGTGTCTGTCTCTGTCTCCGTGCGTGTTTGTGCCGGCATCTAATGTTTGCGCCTGC
GTCTGTACTAGTTGGCTAACTAGATCTGTATCTGGCGGTTCCGCGGAAGAACTGACGAGTTCGTATTCCCGGCCGCAGCC
CCTGGGAGACGTCCCAGCGGCCTCGGGGGCCCGTTTTGTGGCCCATTCTGTATCAGTTAACCTACCCGAGTCGGACTTTT
TGGAGCTCCGCCACTGTACGTGGCTTTGTTGGGGGACGAGAGACAGAGACACTTCCCGCCCCCGTCTGGATTTTTGCTTT
CGGTTTTACGCCGAAACCGCGCTGCGCGTCTGATTTGTTTTATTGCTCTTTTGTTCTTCGTTAGTTTTTTCTGTCTTTAA
GTGTTTTCAAGATCATGGGACAGACCGTAACTACCCCTCTGAGTCTAACCTTGCAGCACTGGGGAGATGTCCAGCGCATT
GCATCCAACCAGTCTGTGGATGTCAGGAAGAGGCGCTGGATTACCTTCTGTTCCGCCGAATGGCCAACTTTCAATGTAGG
ATGGCCTCAGGATGGTACTTTCAATTTAAGTATTATCTCTCAGGTTAAGTCTAGAGTGTTTTGTCCTGGTCCCCACGGAC
ACCCGGATCAGGTCCCATATATCGTTACCTGGGAGGCACTTGCCTATGACCCCCCTCCGTGGGTCAAACCGTTTGTTTCT
CCTAAACCTCCTCCCTTGCCGACAGCTCCCGTCCTCCCGCCCGGTCCTTCTGCGCAACCTCCGTCCCGATCTGCCCTTTA
CCCTGCCCTTACCCCCTCTATAAAGTCCAAACCTCCTAAGCCCCAGGTTCTCCCTGATAGCGGCGGACCTCTCATTGACC
TTCTCACAGAGGACCCCCCGCCGTACGGAGCACAACCTTCCTCCTCTGCCAGAGAGAACGATGAAGAAGAGGCGGCCACC
ACCTCCGAGGTTTCCCCCCCTTCTCCCATGGCGTCTCGACTGCGGGGAAGGAGAGACCCTCCCGCAGCGGACTCCACCTC
CTCCCAGGCATTCCCACTCCGTATGGGGGGAGATGGCCAGCTTCAGTACTGGCCGTTTTCCTCCTCTGACTTATATAATT
GGAAAAATAATAACCCTTCCTTTTCTGAAGATCCAGGTAAATTGACGGCCTTGATTGAGTCCGTCCTCATCACCCACCAG
CCCACCTGGGACGACTGTCAGCAGTTGTTGGGGACCCTGCTGACCGGAGAAGAAAAGCAGCGGGTGCTCCTAGAGGCTAG
AAAGGCAGTCCGGGGCAATGATGGACGCCCCACTCAGTTGCCTAATGAAATCAATGCTGCCTTTCCCCTTGAGCGCCCTG
ATTGGGATTACACCACTACAGAAGGTAGGAACCACCTAGTCCTCTATCGCCAGTTGCTCTTAGCGGGTCTCCAAAACGCG
GGCAGAAGCCCCACCAATTTGGCCAAGGTAAAAGGGATAACCCAGGGACCTAATGAGTCTCCCTCAGCCTTTTTAGAGAG
ACTCAAGGAGGCCTATCGCAGGTACACTCCTTATGACCCTGAGGACCCAGGGCAAGAAACCAATGTGTCTATGTCATTCA
TCTGGCAGTCTGCCCCGGATATCGGGCGAAAGTTAGAGCGGTTAGAAGATTTAAAGAGCAAGACCTTAGGAGACTTAGTG
AGGGAAGCTGAAAAGATCTTTAATAAACGAGAAACCCCGGAAGAAAGAGAGGAACGTATCAGGAGAGAAACAGAGGAAAA
AGAAGAACGCCGTAGGGCAGAGGATGAGCAGAAAGAGAAAGAAAGGGACCGCAGAAGACATAGAGAGATGAGCAAGCTCT
TGGCCACTGTAGTTATTGGTCAGAGACAGGATAGACAGGGGGGAGAGCGGAAGAGGCCCCAACTTGATAAGGATCAATGC
GCCTACTGCAAAGAAAAGGGACACTGGGCTAAAGACTGCCCAAAGAAGCCACGAGGGCCCCGAGGACCGAGGCCCCAGAC
CTCCCTCCTGACCTTAGGTGACTAGGGAGGTCAGGGTCAGGAGCCCCCCCCTGAACCCAGGATAACCCTCAAAGTCGGGG
GGCAACCCGTCACCTTCCTGGTAGATACTGGGGCCCAACACTCCGTGCTGACCCAAAATCTTGGACCCCTAAGTGACAAG
TCTGCCTGGGTCCAAGGGGCTACTGGAGGAAAGCGGTATCGCTGGACCACGGATCGCAAAGTGCATCTAGCTACCGGTAA
GGTCACCCACTCTTTCCTCCATGTACCAGACTGCCCCTATCCTCTGCTAGGAAGAGATTTGCTGACTAAACTAAAAGCCC
AAATCCACTTTGAGGGATCAGGAGCTCAGGTTATGGGACCAATGGGACAGCCCCTGCAAGTGCTGACCCTAAACATAGAA
GATGAGTATCGGCTACATGAGACCCCAAAAGAGCCGGATGTTTCTCTAGGGTCCACATGGCTTTCTGATTTTCCCCAGGC
CTGGGCGGAAACCGGGGGCATGGGACTGGCAGTTCGCCAAGCTCCTCTGATCATACCTCTGAAGGCAACCTCTACCCCCG
TGTCCATAAAACAATACCCCATGTCACAAGAAGCCAGACTGGGGATCAAGTCCCACATACAGAGACTGTTGGACCAGGGA
ATACTGGTACCCTGCCAGTCCCCCTGGAACACGCCCCTGCTACCCGTTAAGAAACCAGGGACTAATGATTACAGGCCTGT
CCAAGATCTGAGAGAAGTCAACAAGCGGGTGGAAGACATCCACCCCACCGTGCCCAACCCTTACAACCTCTTGAGCGGGC
TCCCACCGTCCCACCAGTGGTACACTGTGCTTGACTTAAAGGATGCCTTTTTCTGCCTGAGACTCCACCCCACCAGTCAG
CCTCTCTTCGCCTTTGAGTGGAGAGACCCAGAGATGGGAATCTCAGGACAATTAACCTGGACCAGACTCCCACAGGGTTT
CAAAAACAGTCCCACCCTGTTTGATGAGGCACTGCACAGAGACCTAGCAGGCTTCCGGATCCAGCACCCAGACTTGATCC
TGCTACAGTACGTGGATGATTTACTGCTGGCCGCCACTTCTGAGCTCGACTGCCAACAAGGTACTCGGGCCCTGTTACAA
ACCCTAGGGGACCTCGGGTATCGGGCCTCGGCCAAGAAAGCCCAAATTTGCCAGAAACAGGTCAAGTATCTGGGGTATCT
TCTAAAAGAGGGTCAGAGATGGCTGACTGAGGCCAGAAAAGAGACTGTGATGGGGCAGCCTATTCCGAAGACCCCTCGAC
AACTAAGGGAGTTCCTAGGGACGGCAGGCTTCTGTCGCCTCTGGATCCCTGGGTTTGCAGAAATGGCCGCCCCCTTGTAC
CCTCTCACCAAAACGGGGACTCTGTTTAATTGGGGCCCAGACCAGCAAAAGGCCTATCAAGAAATCAAACAGGCTCTTCT
AACTGCCCCAGCCCTGGGGTTGCCAGATTTGACTAAGCCCTTTGAACTCTTTGTCGACGAGAAGCAGGGCTACGCCAAAG
GCGTCCTAACGCAAAAACTGGGACCTTGGCGTCGGCCGGTGGCCTACCTGTCCAAAAAGCTAGACCCAGTGGCAGCTGGG
TGGCCCCCTTGCCTACGGATGGTAGCAGCCATTGCCGTTCTGACAAAGGATGCAGGCAAGCTAACTATGGGACAGCCGCT

AGTCATTCTGGCCCCCCATGCGGTAGAAGCACTGGTCAAACAACCCCCTGACCGCTGGCTATCCAATGCTCGCATGACCC
ACTATCAGGCAATGCTCCTAGATACGGACCGAGTTCAGTTCGGACCGGTGGTAGCCTTAAACCCGGCCACGTTGCTCCCC
TTGCCGGGAAAAGAGACCCCCCACGACTGCCTCGAGATCTTGGCTGAGACACACGGCACCAGACCGGACCTCACGGACCA
GCCCCTCCCAAATGCCGACCACACCTGGTATACAGATGGAAGCAGCTTCCTGCAGGAGGGGCAACGTAAGGCTGGAGCAG
CGGTGACCACCGAGACCGAGGTAATCTGGGCCAAGGCGTTGCCAGTCGGGACATCCGCCCAGCGAGCTGAACTAATAGCA
CTCACCCAGGCCCTAAAGATGGCAGAAGGTAAGAAGCTAAATGTTTATACTGATAGCCGCTATGCCTTTGCTACCGCCCA
TGTCCATGGAGAAATATATAGGAGACGTGGGTTGCTCACCTCAGAAGGCAAGGAGATCAAGAACAAGGGCGAAATCTTGG
CCTTACTGAAAGCTCTCTTTCTGCCCAAAAGACTCAGTATAATTCACTGCCCAGGACATCAGAAAGGCAATAGTGCTGAA
GCTAAAGGCAACCGAATGGCGGACCAGGCAGCCCGGGAAGCAGCCATGGGGACTGACACAAAGGCCTCCTCACTTCTCAT
AGAGACCTCAACCCCGTACACTCCAGACTTCTTCCATTATACTGAAACAGATATAAAGAACCTACGAGAGTTGGGAGCCA
CATATGATAGAGAGAAAAAATATTGGGTCCTGCAAGGTAAACCTGTGATGCCTGACCAGTTCACCTTTGAATTATTAGAC
TTCCTTCACCAGCTCACCCACCTTAGCTATCAGAAGATGAGGGCACTTCTAGACAGAAAAGAAAGCCCTTATTACATGCT
AAATAAAGATAAGATCCTCCACGAGGTGGCGGAATCATGCCAAGCCTGTGTCCAAGTAAATGCCAGTAAAGCTAAGGTCG
GTCCCGGGGTGCGAGTAAGAGGACATCGACCAGGCACCCATTGGGAAATTGACTTTACTGAAGTAAGGCCCGGACTGTAT
GGGCATAAGTATCTTCTGGTGTTTGTGGACACGTTCTCTGGCTGGGTGGAAGCCTTCCCAACCAAGCATGAGACTGCCAA
AGTTGTGACCAAAAAGCTTCTAGAAGAAATATTTCCAAGGTTTGGAATGCCCCAAGTATTGGGGACTGATAATGGGCCTG
CCTTCGTCTCCCAGGTAAGTCAGTCGGTGGCCAAGCTACTGGGGATTGATTGGAAACTACATTGTGCTTACAGACCCCAG
AGTTCAGGTCAGGTAGAAAGAATGAATAGGACAATCAAGGAGACTTTGACCAAATTAACGCTTGCAACTGGCACTAGAGA
CTGGGTACTCCTACTTCCCTTGGCCCTCTACCGAGCCCGCAACACTCCGGGCCCCCATGGACTCACTCCGTATGAAATCC
TGTATGGGGCGCCCCCGCCCCTTGTTAATTTCCATGATCCTGAAATGTCAAAGTTTACTAATAGCCCCTCTCTCCAAGCT
CACTTACAGGCCCTCCAAGCAGTACAACGAGAGGTCTGGAAGCCTCTGGCCGCTGCCTATCAGGACCAACAAGACCAGCC
TGTGATACCACACCCCTTCCGTGTCGGCGACACCGTGTGGGTACGCCGGCACCAGACTAAGAACTTGGAACCTCGCTGGA
AAGGACCCTACACCGTCCTGCTGACCACCCCCACCGCTCTCAAAGTAGACGGCATCGCTGCGTGGATCCACGCCGCTCAC
GTAAAAGCGGCGACAACCCCTCCGGCCGGAACAGCATCAGGACCGACATGGAAGGTCCAGCGTTCTCAAAACCCCTTAAA
GATAAGATTAACCCGTGGGCCCCCCTGATAGTCCTGGGGATCTTAATAAGGGCAGGAGTATCAGTACCACATGACAGCCC
TCATCAGGTCTTCAATGTTACTTGGAGAGTTACCAACTTAATGACAGGACAAACAGCTAATGCTACCTCCCTCCTGGGGA
CAATGACCGATGCCTTTCCCAAACTGTACTTTGACTTGTGCGATTTAATAGGGGACGACTGGGATGAGACTGGACTCGGG
TGTCGCACTCCCGGGGGAAGAAAAAGGGCAAGAACATTTGACTTCTATGTTTGCCCCGGGCATACTGTACCAACAGGGTG
TGGAGGCCCGAGAGAGGGCTACTGTGGCAAATGGGGCTGTGAGACCACTGGACAGGCATACTGGAAGCCATCATCATCAT
GGGACCTAATTTCCCTTAAGCGAGGAAACACCCCTCGGAATCAGGGCCCCTGTTATGATTCCTCAGCGGTCTCCAGTGGC
ATCCAGGGTGCCACACCGGGGGGTCGATGCAATCCCCTAGTCCTAGAATTCACTGACGCGGGTAAAAAGGCCAGCTGGGA
TGGCCCCAAAGTATGGGGACTAAGACTGTACCGATCCACAGGAACCGACCCGGTGACCCGGTTCTCTTTGACCCGCCAGG
TCCTCAATATAGGGCCCCGCATCCCCATTGGGCCTAATCCCGTGATCACTGACCAGTTACCCCCCTCCCGACCCGTGCAG
ATCATGCTCCCCAGGCCTCCTCAGCCTTCCCCTACAGGCGCAGCCTCTATACAACCTGGGACGGGAGACAGACTGCTGAA
CCTGGTAGATGGAGCCTACCAAGCACTCAACCTCACCAGTCCTGACAAAACCCAAGAGTGCTGGTTGTGTCTGGTATCGG
GACCCCCCTATTACGAAGGGGTTGCCGTCCTAGGTACCTACTCCAACCATACCTCTGCCCCAGCTAACTGCTCCGTGGCC
TCCCAACACAAGCTGACCCTGTCCGAAGTAACCGGACAGGGACTCTGCGTAGGAGCAGTTCCCAAAACCCATCAGGCCCT
GTGTAATACCACCCAGAATACAAGCGACGGGTCCTACTATCTGGCTGCTCCCGCCGGGACCATTTGGGCTTGCAACACCG
GGCTCACTCCCTGCCTATCTACTACTGTACTCAACCTCACCACCGATTACTGTGTCCTGGTTGAGCTCTGGCCAAAGGTG
ACCTACCACTCCCCTGGTTATGTTTATGGCCAGTTTGAGAGAAAAACCAAATATAAAAGAGAGCCGGTGTCATTAACTCT
GGCCCTGCTGTTGGGAGGACTTACTATGGGCGGCATAGCTGCAGGAGTAGGAACAGGGACTACAGCCCTAGTGGCCACCA
AGCAATTCGAGCAGCTCCAGGCAGCCATACATACAGACCTTGGGGCCTTAAAAAAATCAGTCAGTGCCCTAGAAAAGTCT
CTGACCTCGTTGTCTGAGGTGGTCCTACAGAACCGGAGAGGATTAGATCTACTGTTCCTAAAAGAAGGAGGATTATGTGC
TGCCCTAAAAGAAGAATGCTGTTTCTACGCGGACCACACTGGCGTAGTGAGAGATAGCATGGCAAAGCTAAGAGAAAGGT
TAAACCAGAGACAAAAATTGTTCGAATCAGGACAAGGGTGGTTTGAGGGACTGTTTAACAGGTCCCCATGGTTCACGACC
TTAATATCCACCATTATGGGCCCCTTGATAATACTCTTATTAATCCTACTCTTCGGACCCTGTATTCTCAACCGCTTGGT
CCAGTTTGTAAAAGACAGAATTTCGGTGGTGCAGGCCCTGGTTCTGACCCAACAGTATCACCAACTCAAATCAATAGGTC
CAGAAGAAGTGAAATCACGTGAATAAAAGATTTTATTCAGTTTCCATAAAGAGGGGGGAATGAAAGACCCCACCATAAGG
CTTAGCAAGCTAGCTGCAGTAACGCCATTTTGCAAGGCATGAAAAAGTACCAGAGCTGGGTTCTCAAAAGTTACAAGGAA
GTTCAGTTATAGATTAACAGTTAAAGATCAAGGCTGAATAGCACTGGGACAGGGGCCGAACAGGATATCGGTGGTCAAGC
ACCTGGGCCCCGGCTCAGGGCCAAGAACAGATGGTTCTCAGATAAAGCGGAACCAGCAACAGACACAGAAGCCCCGATAG
ACGTCAGTGTTAGCAGAACTAGCTTCACTGATTTAGAAAAATAGAGGTGCACAGTGCTCTGGTCACTCCTTGAACCTGTG
TGTCTGCCAATGTTCTGACCAGATATGTGCCCATTGCTGAACCTTCATTAGACTCTTTCCTTGTACCCCTCCCCTACCCA
TTTCTTGAAAATAGACATTGTTTAGATCTAAAAAGTCCCACCTCAGTTTCCCCAAATGACCGGGAAGTACCCCAAACCTT
ATTCGAACTAACCAACCAGCTCGCTTCTCGCTTCTGTAACCGCGCTTTTTGCTCCCCAGCCCTAGCCCTATAAAAAGGGT
AAAAACTCCACACTCGGCGCGCCAGTCCTCCGATAGACTGAGTCGCCCGGGTACCCGTGTTCCCAATAAAGCCTCTTGCT

GTTTACATCCGAATCGTGGACTCGCTGATCCTTGGGAGGGTCTCCTCAGATTGATTGACTGCCCACCTGGGGGGTCTTTC
A
>Mpmv10
TGAAAGACCCCACCATAAGGCTTAGCAAGCTAGCTGCAGTAACGCCATTTTTGCAAGGCATGAAAAAGTACCAGAGCTGG
GTTCTCAAAAGTTACAAGGAAGTTCAGTTATAGATTAACAGTTAAAGATCAAGGCTGAATAGCACTGGGACAGGGGCCGA
ACAGGATATCGGTGATCAAGCACCTGGGCCCCGGCTCAGGGCCAAGAACAGATGGTTCTCAGATAAAGCGGAACCAGCAA
CAGACACAGAAGCCCCGATAGACGTCAGTGTTAGCAGAACTAGCTTCACTGATTTAGAAAAATAGAGGTGCACAGTGCTC
TGGTCACTCCTTGAACCTGTGTGTCTGCCAATGTTCTGACCAGATATGTGCCCATTGCTGAACCTTCATTAGACTCTTTC
CTTGTACCCCTCCCCTACCCATTTCTTGAAAATAGACATTGTTTAGATCTAAAAAGTCCCACCTCAGTTTCCCCAAATGA
CCGGGAAGTACCCCAAACCTTATTCGAACTAACCAACCAGCTCGCTTCTCGCTTCTGTAACCGCGCTTTTTGCTCCCCAG
CCCTAGCCCTATAAAAAGGGTAAAAACTCCACACTCGGCGCGCCAGTCCTCCGATAGACTGAGTCGCCCGGGTACCCGTG
TTCCCAATAAAGCCTCTTGCTGTTTACATCCGAATCGTGGACTCGCTGATCCTTGGGAGGGTCTCCTCAGATTGATTGAC
TGCCCACCTGGGGGGTCTTTCATTTGGAGGCCCCAGCGAGATCAGGAGACCCCTGCCCAGGGACCACCGACCCCCGCCG
GGAGGTAAGCTGGCCAGCGGTCGTTTCGTGTCTGTCTCTGTCTCCGTGCGTGTTTGAGCCGGCATCTAATGTTTGCGCCT
GCGTCTGTACTAGTTGGCTAACTAGATCTGTATCTGGCGGTTCCGCGGAAGAACTGACGAGTTCGTATTCCCGGCCGCAG
CCCCTGGGAGACGTCCCAGCGGCCTCGGGGGCCCGTTTTGTGGCCCATTCTGTATCAGTTAACCTACCCGAGTCGGACTT
TTTGGAGCTCCGCCACTGTACGTGGCTTTGTTGGGGGACGAGAGACAGAGACACTTCCCGCCCCCGTCTGGATTTTTGCT
TTCGGTTTTACGCCGAAACCGCGCTGCGCGTCTGATTTGTTTTATTGCTCTTTTGTTCTTCGTTAGTTTTTTCTGTCTTT
AAGTGTTTTCAAGATCATGGGACAGACCGTAACTACCCCTCTGAGTCTAACCTTGCAGCACTGGGGAGATGTCCAGCGCA
TTGCATCCAACCAGTCTGTGGATGTCAGGAAGAGGCGCTGGATTACCTTCTGTTCCGCCGAATGGCCAACTTTCAATGTA
GGATGGCCTCAGGATGGTACTTTCAATTTAAGTATTATCTCTCAGGTTAAGTCTAGAGTGTTTTGTCCTGGTCCCCACGG
ACACCCGGATCAGGTCCCATATATCGTTACCTGGGAGGCACTTGCCTATGACCCCCCTCCGTGGGTCAAACCGTTTGTTT
CTCCTAAACCTCCTCCCTTGCCGACAGCTCCCGTCCTCCCGCCCGGTCCTTCTGCGCAACCTCCGTCCCGATCTGCCCTT
TACCCTGCCCTTACCCCCTCTATAAAGTCCAAACCTCCTAAGCCCCAGGTTCTCCCTGATAGCGGCGGACCTCTCATTGA
CCTTCTCACAGAGGACCCCCCGCCGTACGGAGCACAACCTTCCTCCTCTGCCAGAGAGAACGATGAAGAAGAGGCGGCCA
CCACCTCCGAGGTTTCCCCCCCTTCTCCCATGGCGTCTCGACTGCGGGGAAGGAGAGACCCTCCCGCAGCGGACTCCACC
TCCTCCCAGGCATTCCCACTCCGTATGGGGGGAGATGGCCAGCTTCAGTACTGGCCGTTTTCCTCCTCTGACTTATATAA
TTGGAAAAATAATAACCCTTCCTTTTCTGAAGATCCAGGTAAATTGACGGCCTTGATTGAGTCCGTCCTCATCACCCACC
AGCCCACCTGGGACGACTGTCAGCAGTTGTTGGGGACCCTGCTGACCGGAGAAGAAAAGCAGCGGGTGCTCCTAGAGGCT
AGAAAGGCAGTCCGGGGCAATGATGGACGCCCCACTCAGTTGCCTAATGAAATCAATGCTGCCTTTCCCCTTGAGCGCCC
TGATTGGGATTACACCACTACAGAAGGTAGGAACCACCTAGTCCTCTATCGCCAGTTGCTCTTAGCGGGTCTCCAAAACG
CGGGCAGAAGCCCCACCAATTTGGCCAAGGTAAAAGGGATAACCCAGGGACCTAATGAGTCTCCCTCAGCCTTTTTAGAG
AGACTCAAGGAGGCCTATCGCAGGTACACTCCTTATGACCCTGAGGACCCAGGGCAAGAAACCAATGTGTCTATGTCATT
CATCTGGCAGTCTGCCCCGGATATCGGGCGAAAGTTAGAGCGGTTAGAAGATTTAAAGAGCAAGACCTTAGGAGACTTAG
TGAGGGAAGCTGAAAAGATCTTTAATAAACGAGAAACCCCGGAAGAAAGAGAGGAACGTATCAGGAGAGAAACAGAGGAA
AAAGAAGAACGCCGTAGGGCAGAGGATGAGCAGAAAGAGAAAGAAAGGGACCGCAGAAGACATAGAGAGATGAGCAAGCT
CTTGGCCACTGTAGTTATTGGTCAGAGACAGGATAGACAGGGGGGAGAGCGGAAGAGGCCCCAACTTGATAAGGATCAAT
GCGCCTACTGCAAAGAAAAGGGACACTGGGCTAAAGACTGCCCAAAGAAGCCACGAGGGCCCCGAGGACCGAGGCCCCAG
ACCTCCCTCCTGACCTTAGGTGACTAGGGAGGTCAGGGTCAGGAGCCCCCCCTGAACCCAGGATAACCCTCAAAGTCGG
GGGGCAACCCGTCACCTTCCTGGTAGATACTGGGGCCCAACACTCCGTGCTGACCCAAAATCCTGGACCCCTAAGTGACA
AGTCTGCCTGGGTCCAAGGGGCTACTGGAGGAAAGCGGTATCGCTGGACCACGGATCGCAAAGTGCATCTAGCTACCGGT
AAGGTCACCCACTCCTTCCTCCATGTACCAGACTGCCCCTATCCTCTGCTAGGAAGAGATTTGCTGACTAAACTAAAAGC
CCAAATCCACTTTGAGGGATCAGGAGCTCAGGTTGTGGGACCAATGGGACAGCCTCTGCAAGTGCTGACCCTAAACATAG
AAGATGAGTATCGGCTACATGAGACCTCAAAAGAGCCGGATGTTTCTCTAGGGTCCACATGGCTTTCTGATTTTCCCCAG
GCCTGGGCGGAAACCGGGGGCATGGGACTGGCAGTTCGCCAAGCTCCTCTGATCATACCTCTGAAGGCAACCTCTACCCC
CGTGTCCATAAAATACGGACCGAGTTCAGTTCGGACCGGTGGTAGCCTTAAACCCGGCCACGTTGCTCCCCTTGCCGGGA
AAAGAGACCCCCCACGACTGCCTCGAGATCCTGGCTGAGACACACGGCACCAGACCGGACCTCACGGACCAGCCCCTCCC
AAATGCCGACCACACCTGGTATACAGATGGAAGCAGCTTCCTGCAGGAGGGGCAACGTAAGGCTGGAGCAGCGGTGACCA
CCGAGACCGAGGTAATCTGGGCCAAGGCGTTGCCAGCCGGGACATCCGCCCAGCGAGCTGAACTAATAGCACTCACCCAG
GCCCTAAAGATGGCAGAAGGTAAGAAGCTAAATGTTTATACTGATAGCCGCTATGCCTTTGCTACCGCCCATGTCCATGG
AGAAATATATAGGAGACGTGGGTTGCTCACCTCAGAAGGCAAGGAGATCAAGAACAAGGGCGAAATCTTGGCCTTACTGA
AAGCTCTCTTTCTGCCCAAAAGACTCAGTATAATTCACTGCCCAGGACATCAGAAAGGCAATAGTGCTGAAGCTAAAGGC
AACCGAATGGCGGACCAGGCAGCCCGGGAAGCAGCCATGGGGACTGACACAAAGGCCTCCTCACTTCTCATAGAGACCTC
AACCCCGTACACTCCAGACTTCTTCCATTATACTGAAACAGATATAAAGAACCTACGAGAGTTGGGAGCCACATATGATA
GAGAGAAAAAATATTGGGTCCTGCAAGGCAAACCTGTGATGCCTGACCAGTTCACCTTTGAATTATTAGACTTCCTTCAC
CAGCTCACCCACCTTAGCTATCAGAAGATGAGGGCACTTCTAGACAAAAAAGAAAGCCCCTATTACATGCTAAATAAAAA
TAAGATCCTCCACGAGGTGGCGGAATCATGCCAAGCCTGTGTCCAAGTAAATGCCAGTAAAGCTAAGGTCGGTCCCGGGG

TGCGAGTAAGAGGACATCGACCAGGCACCCATTGGGAAATTGACTTTACTGAAGTAAGGCCCGGACTGTATGGGCATAAG
TATCTTCTGGTGTTTGTGGACACGTTCTCTGGCTGGGTGGAAGCCTTCCCAACCAAGCATGAGATTGCCAAAGTTGTGAC
CAAAAAGCTTCTAGAAGAAATATTTCCAAGGTTTGGAATGCCCCAAGTATTGGGGACTGATAATGGGCCTGCCTTCGTCT
CCCAGGTAAGTCAGTCGGTGGCCAAGCTACTGGGGATTGATTAGAAACTACATTGTGCTTACAGACCCCAGAGTTCAGGT
CAGGTAAAAAGAATGAATAGGACAATCAAGGAGACTTTGACCAAATTAACGCTTGCAACTGGCACTAGAGACTGGGTACT
CCTACTTCCCTTGGCCCTCTACCGAGCCCGCAACACTCCGGGCCCCCATGGACTCACTCCGTATGAAATCCTGTATGGGG
CGCCCCCGCCCCTTGTTAATTTCCATGATCCTGAAATGTCAAAGTTTACTAATAGCCCCTCTCTCCAAGCTCACTTACAG
GCCCTCCAAGCAGTACAACAAGAGGTCTGGAAGCCGCTGGCCGCTGCCTATCAGGACCAACAAGACCAGCCTGTGATACC
ACACCCCTTCCGTGTCGGCGACACCGTGTGGGTACGCCGGCACCAGACTAAGAACTTGGAACCTCGCTGGAAAGGACCCT
ACACCGTCCTGCTGACCACCCCCACCGCTCTCAAAGTAGACGGCATCGCTGCGTGGATCCACGCCGCTCACGTAAAAGCG
GCGACAACCCCTCCGGCCGGAACAGCATCAGGACCGACATGGAAGGTCCAGCGTTCTCAAAACCCCTTAAAGATAAGATT
AACCCGTGGGCCCCCCTGATAGTCCTGGAGATCTTAATAAGGGCAGGAGTATCAGTACCACATGACAGCCCTCATCAGGT
CTTCAATGTTACTTGGAGAGTTACCAACTTAATGACAGGACAAACAGCTAATGCTACCTCCCTCCTGGGGACAATGACCG
ATGCCTTTCCCAAACTGTACTTTGACTTGTGCGATTTAATAGGGGACGACTGGGATGAGACTGGACTCGGGTGTCGCACT
CCCGGGGGAAGAAAAAGGGCAAGAACATTTGACTTCTATGTTTGCCCCGGGCATACTGTACCAACAGGGTGTGGAGGCCC
GAGAGAGGGCTACTGTGGCAAATGGGGCTGTGAGACCACTGGACAGGCATACTGGAAGCCATCATCATCATGGGACCTAA
TTTCCCTTAAGCGAGGAAACACCCCTCGGAATCAGGGCCCCTGTTATGATTCCTCAGCGGTCTCCAGTGGCATCCAGGGT
GCCACACCGGGGGGTCGATGCAATCCCCTAGTCCTAGAATTCACTGACGCGGGTAAAAAGGCCAGCTGGGATGGCCCCAA
AGTATGGGGACTAAGACTGTACCGATCCACAGGAACCGACCCGGTGACCCGGTTCTCTTTGACCCGCCAGGTCCTCAATA
TAGGGCCCCGCATCCCCATTGGGCCTAATCCCGTGATCACTGACCAGTTACCCCCCTCCCGACCCGTGCAGATCATGCTC
CCCAGGCCTCCTCAGCCTTCCCCTACAGGCGCAGCCTCTATACAACCTGGGACGGGAGACAGACTGCTGAACCTGGTAGA
TGGAGCCTACCAAGCACTCAACCTCACCAGTCCTGACAAAACCCAAGAGTGCTGGTTGTGTCTGGTATCGGGACCCCCCT
ATTACGAAGGGGTTGCCGTCCTAGGTACCTACTCCAACCATACCTCTGCCCCAGCTAACTGCTCCGTGGCCTCCCAACAC
AAGCTGACCCTGTCCGAAGTAACCGGACAGGGACTCTGCGTAGGAGCAGTTCCCAAAACCCATCAGGCCCTGTGTAATAC
CACCCAGAATACAAGCGACGGGTCCTACTATCTGGCTGCTCCCGCCGGGACCATTTGGGCTTGCAACACCGGGCTCACTC
CCTGCCTATAAAAGAGAGCCGGTGTCATTAACTCTGGCCCTGCTGTTGGGAGGACTTACTATGGGCGGCATAGCTGCAGG
AGTAAGAACAGGGACTACAGCCCTAGTGGCCACCAAGCAATTCGAGCAGCTCCAGGCAGCCATACATACAGACCTTGGGG
CCTTAGAAAAATCAGTCAGTGCCCTAGAAAAGTCTCTGACCTCGTTGTCTGAGGTGGTCCTACAGAACCGGAGAGGATTA
GATCTACTGTTCCTAAAAGAAGGAGGATTATGTGCTGCCCTAAAAGAAGAATGCTGCTTCTACGCGGACCACACTGGCGT
AGTGAGAGATAGCATGGCAAAGCTAAGAGAAAGGTTAAACCAGAGACAAAAATTGTTCGAATCAGGACAAGGGTGGTTTG
AGGGACTGTTTAACAGGTCCCCATGGTTCACGACCTTGATATCCACCATTATGGGCCCCTTGATAATACTCTTATTAATC
CTACTCTTCGGACCCTGTATTCTCAACCGCTTGGTCCAGTTTGTAAAAGACAGAATTTCGGTGGTGCAGGCCCTGGTTCT
GACCCAACAGTATCACCAACTCAAATCAATAGGTCCAGAAGAAGTGGAATCACGTGAATAAAAGATTTTATTCAGTTTCC
AGAAAGAGGGGGGAATGAAAGACCCCACCATAAGGCTTAGCAAGCTAGCTGCAGTAACGCCATTTTTGCAAGGCATGAAA
AAGTACCAGAGCTGGGTTCTCAAAAGTTACAAGGAAGTTCAGTTATAGATTAACAGTTAAAGATCAAGGCTGAATAGCAC
TGGGACAGGGGCCGAACAGGATATCGGTGATCAAGCACCTGGGCCCCGGCTCAGGGCCAAGAACAGATGGTTCTCAGATA
AAGCGGAACCAGCAACAGACACAGAAGCCCCGATAGACGTCAGTGTTAGCAGAACTAGCTTCACTGATTTAGAAAAATAG
AGGTGCACAGTGCTCTGGTCACTCCTTGAACCTGTGTGTCTGCCAATGTTCTGACCAGATATGTGCCCATTGCTGAACCT
TCATTAGACTCTTTCCTTGTACCCCTCCCCTACCCATTTCTTGAAAATAGACATTGTTTAGATCTAAAAAGTCCCACCTC
AGTTTCCCCAAATGACCGGGAAGTACCCCAAACCTTATTCGAACTAACCAACCAGCTCGCTTCTCGCTTCTGTAACCGCG
CTTTTTGCTCCCCAGCCCTAGCCCTATAAAAAGGGTAAAAACTCCACACTCGGCGCGCCAGTCCTCCGATAGACTGAGTC
GCCCGGGTACCCGTGTTCCCAATAAAGCCTCTTGCTGTTTACATCCGAATCGTGGACTCGCTGATCCTTGGGAGGGTCTC
CTCAGATTGATTGACTGCCCACCTGGGGGGTCTTTCA
>Mpmv11
TGAAAGACCCCACCATAAGGCTTAGCAAGCTAGCTGCAGTAACGCCATTTTGCAAGGCATGAAAAAGTACCAGAGCTGGG
TTCTCAAAAGTTACAAGGAAGTTCAGTTATAGATTAACAGTTAAAGATCAAGGCTGAATAGCACTGGGACAGGGGCCGAA
CAGGATATCGGTGGTCAAGCACCTGGGCCCCGGCTCAGGGCCAAGAACAGATGGTTCTCAGATAAAGCGGAACCAGCAAC
AGACACAGAAGCCCCGATAGACGTCAGTGTTAGCAGAACTAGCTTCACTGATTTAGAAAAATAGAGGTGCACAGTGCTCT
GGTCACTCCTTGAACCTGTGTGTCTGCCAATGTTCTGACCAGATATGTGCCCATTGCTGAACCTTCATTAGACTCTTTCC
TTGTACCCCTCCCCTACCCATTTCTTGAAAATAGACATTGTTTAGATCTAAAAAGTCCCACCTCAGTTTCCCCAAATGAC
CGGGAAGTACCCCAAACCTTATTCGAACTAACCAACCAGCTCGCTTCTCGCTTCTGTAACCGCGCTTTTTGCTCCCCAGC
CCTAGCCCTATAAAAAGGGTAAAAACTCCACACTCGGCGCGCCAGTCCTCCGATAGACTGAGTCGCCCGGGTACCCGTGT
TCCCAATAAAGCCTCTTGCTGTTTACATCCGAATCGTGGACTCGCTGATCCTTGGGAGGGTCTCCTCAGATTGATTGACT
GCCCACCAGGGGGGTCTTTCATTTGGAGGTTCCACCGAGATCAGGAGACCCCTGCCCAGGGACCACCGACCCCCGCCGGG
AGGTAAGCTGGCCAGCGGTCGTTTTGTGTCTGTCTCTGTCTCCGTGCGTGTTTGTGCCGGCATCTAATGTTTGCGCCTGC
GTCTGTACTAGTTGGCTAACTAGATCTGTATCTGGCGGTTCCGCGGAAGAACTGACGAGTTCGTATTCCCGGCCGCAGCC
CCTGGGAGACGTCCCAGCGGCCTCGGGGGCCCGTTTTGTGGCCCATTCTGTATCAGTTAACCTACCCGAGTCGGACTTTT

TGGAGCTCCGCCACTGTACGTGGCTTTGTTGGGGGACGAGAGACAGAGACACTTCCCGCCCCCGTCTGGATTTTTGCTTT
CGGTTTTACGCCGAAACCGCGCTGCGCGTCTGATTTGTTTTATTGCTCTTTTGTTCTTCGTTAGTTTTTTCTGTCTTTAA
GTGTTTTCAAGATCATGGGACAGACCGTAACTACCCCTCTGAGTCTAACCTTGCAGCACTGGGGAGATGTCCAGCGCATT
GCATCCAACCAGTCTGTGGATGTCAGGAAGAGGCGCTGGATTACCTTCTGTTCCGCCGAATGGCCAACTTTCAATGTAGG
ATGGCCTCAGGATGGTACTTTCAATTTAAGTATTATCTCTCAGGTTAAGTCTAGAGTGTTTTGTCCTGGTCCCCACGGAC
ACCCGGATCAGGTCCCATATATCGTTACCTGGGAGGCACTTGCCTATGACCCCCCTCCGTGGGTCAAACCGTTTGTTTCT
CCTAAACCTCCTCCCTTGCCGACAGCTCCCGTCCTCCCGCCCGGTCCTTCTGCGCAACCTCCGTCCCGATCTGCCCTTTA
CCCTGCCCTTACCCCCTCTATAAAGTCCAAACCTCCTAAGCCCCAGGTTCTCCCTGATAGCGGCGGACCTCTCATTGACC
TTCTCACAGAGGACCCCCCGCCGTACGGAGCACAACCTTCCTCCTCTGCCAGAGAGAACGATGAAGAAGAGGCGGCCACC
ACCTCCGAGGTTTCCCCCCCTTCTCCCATGGCGTCTCGACTGCGGGGAAGGAGAGACCCTCCCGCAGCGGACTCCACCTC
CTCCCAGGCATTCCCACTCCGTATGGGGGGAGATGGCCAGCTTCAGTACTGGCCGTTTTCCTCCTCTGACTTATATAATT
AGAAAAATAATAACCCTTCCTTTTCTGAAGATCCAGGTAAATTGACGGCCTTGATTGAGTCCGTCCTCATCACCCACCAG
CCCACCTGGGACGACTGTCAGCAGTTGTTGGGGACCCTGCTGACCGGAGAAGAAAAGCAGCGGGTGCTCCTAGAGGCTAG
AAAGGCAGTCCGGGGCAATGATGGACGCCCCACTCAGTTGCCTAATGAAATCAATGCTGCCTTTCCCCTCGAGCGCCCTG
ATTGGGATTACACCACTACAGAAGGTAGGAACCACCTAGTCCTCTATCGCCAGTTGCTCTTAGCGGGTCTCCAAAACGCG
GGCAGAAGCCCCACCAATTTGGCCAAGGTAAAAGGGATAACCCAGGGACCTAATGAGTCTCCCTCAGCCTTTTTAGAGAG
ACTCAAGGAGGCCTATCGCAGGTACACTCCTTATGACCCTGAGGACCCAGGGCAAGAAACCAATGTGTCTATGTCATTCA
TCTGGCAGTCTGCCCCGGATATCGGGCGAAAGTTAGAGCGGTTAGAAGATTTAAAGAGCAAGACCTTAGGAGACTTAGTG
AGGGAAGCTGAAAAGATCTTTAATAAACGAGAAACCCCGGAAGAAAGAGAGGAACGTATCAGGAGAGAAACAGAGGAAAA
AGAAGAACGCCGTAGGGCAGAGGATGAGCAAAAAGAGAAAGAAAGGGACCGCAGAAGACATAGAGAGATGAGCAAGCTCT
TGGCCACTGTAGTTATTGGTCAGAGACAGGATAGACAGGGGGGAGAGCGGAAGAGGCCCCAACTTGATAAAGATCAATGC
GCCTACTGCAAAGAAAAGGGACACTGGGCTAAAGACTGCCCAAAGAAGCCACGAGGGCCCCGAGGACCGAGGCCCCAGAC
CTCCCTCCTGACCTTAGGTGACTAGGGAGGTCAGGGTCAGGAGCCCCCCCCTGAACCCAGGATAACCCTCAAAGTCGGGG
GGCAACCCGTCACCTTCCTGGTAGATACTGGGGCCCAACACTCCGTGCTGACCCAAAATCCTGGACCCCTAAGTGACAAG
TCTGCCTGGGTCCAAGGGGCTACTGGAGGAAAGCGGTATCGCTGGACCACGGATCGCAAAGTGCATCTAGCTACCGGTAA
GGTCACCCACTCTTTCCTCCATGTACCAGACTGCCCCTATCCTCTGCTAGGAAGAGATTTGCTGACTAAACTAAAAGCCC
AAATCCACTTTGAGGGATCAGGAGCTCAGGTTATGGGACCAATGGGACAGCCCCTGCAAGTGCTGACCCTAAACATAGAA
GATGAGTATCGGCTACATGAGACCTCAAAAGAGCCGGATGTTTCTCTAGGGTCCACATGGCTTTCTGATTTTCCCCAGGC
CTGGGCGGAAACCGGGGGCATGGGACTGGCAGTTCGCCAAGCTCCTCTGATCATACCTCTGAAGGCAACCTCTACCCCCG
TGTCCATAAAACAATACCCCATGTCACAAGAAGCCAGACTGGGGATCAAGCCCCACATACAGAGACTGTTGGACCAGGGA
ATACTGGTACCCTGCCAGTCCCCCTGGAACATGCCCCTGCTACCCGTTAAGAAACCAGGGACTAATGATTACAGGCCTGT
CCAAGATCTGAGAGAAGTCAACAAGCGGGTGGAAGACATCCACCCCACCGTGCCCAACCCTTACAACCTCTTGAGCGGGC
TCCCACCGTCCCACCAGTGGTACACTGTGCTTGACTTAAAGGATGCCTTTTTCTGCCTAAGACTCCACCCCACCAGTCAG
CCTCTCTTCGCCTTTGAGTGGAGAGACCCAGAGATGGGAATCTCAGGACAATTAACCTGGACCAGACTCCCACAGGGTTT
CAAAAACAGTCCCACCCTGTTTGATGAGGCACTGCACAGAGACCTAGCAGGCTTCCGGATCCAGCACCCAGACTTGATCC
TGCTACAGTACGTGGATGACTTACTGCTGGCCGCCACTTCTGAGCTCGACTGCCAACAAGGTACTCGGGCCCTGTTACAA
ACCTTAGGGGACCTCGGGTATCGGGCCTCGGCCAAGAAAGCCCAAATTTGCCAGAAACAGGTCAAGTATCTGGGGTATCT
TCTAAAAGAGGGTCAGAGATGGCTGACTGAGGCCAGAAAAGAGACTGTGATGGGGCAGCCTATTCCGAAGACCCCTCGAC
AACTAAGGGAGTTCCTAGGGACGGCAGGCTTCTGTCGCCTCTGGATCCCTGGGTTTGCAGAAATGGCAGCCCCCTTGTAC
CCTCTCACCAAAACGGGGACTCTGTTTAATTGGGGCCCAGACCAGCAAAAGGCCTATCAAGAAATCAAACAGGCTCTTCT
AACTGCCCCAGCCCTGGGGTTGCCAGATTTGACTAAGCCCTTTGAACTCTTTGTCGACGAGAAGCAGGGCTACGCCAAAG
GCGTCCTAACGCAAAAACTGGGACCTTGGCGTCGGCCGGTGGCCTACCTGTCCAAAAAGCTAGACCCAGTGGCAGCTGGG
TGGCCCCCTTGCCTACGAATGGTAGCAGCCATTGCCGTTCTGACAAAGGATGCAGGCAAGCTAACTATGGGACAGCCGCT
AGTCATTCTGGCCCCCCATGCGGTAGAAGCACTGGTCAAACAACCCCCTGACCGCTGGCTATCCAATGCTCGCATGACCC
ACTATCAGGCAATGCTCCTAGATACGGACCGAGTTCAGTTCGGACCGGTGGTAGCCTTAAACCCGGCCACGTTGCTCCCC
TTGCCGGGAAAAGAGACCCCCCACGACTGCCTCGAGATCTTGGCTGAGACACACGGCACCAGACCGGACCTCACGGACCA
GCCCCTCCCAAATGCCGACCACACCTGGTATACAGATGGAAGCAGCTTCCTGCAGGAGGGGCAACGTAAGGCTGGAGCAG
CGGTGACCACCGAGACCGAGGTAATCTGGGCCAAGGCGTTGCCAGTCGGGACATCCGCCCAGCGAGCTGAACTAATAGCA
CTCACCCAGGCCCTAAAGATGGCAGAAGGTAAGAAGCTAAATGTTTATACTGATAGCCGCTATGCCTTTGCTACCGCCCA
TGTCCATGGAGAAATATATAGGAGACGTGGGTTGCTCACCTCAGAAGGCAAGGAGATCAAGAACAAGGGCGAAATCTTGG
CCTTACTGAAAGCTCTCTTTCTGCCCAAAAGACTCAGTATAATTCACTGCCCAGGACATCAGAAAGGCAATAGTGCTGAA
GCTAAAGGCAACCGAATGGCGGACCAGGCAGCCCGGGAAGCAGCCATGGGGACTGACACAAAGGCCTCCTCACTTCTCAT
AGAGACCTCAACCCCGTACACTCCAGACTTCTTCCATTATACTGAAACAGATATAAAGAACCTACGAGAGTTGGGAGCCA
CATATGATAGAGAGAAAAAATATTGGGTCCTGCAAGGTAAACCTGTGATGCCTGACCAGTTCACCTTTGAATTATTAGAC
TTCCTTCACCAGCTCACCCACCTTAGCTATCAGAAGATGAGGGCACTTCTAGACAGAAAAGAAAGCCCTTATTACATGCT
AAATAAAGATAAGATCCTCCACGAGGTGGCGGAATCATGCCAAGCCTGTGTCCAAGTAAATGCCAGTAAAGCTAAGGTCG
GTCCCGGGGTGCGAGTAAGAGGACATCGACCAGGCACCCATTGGGAAATTGACTTTACTGAAGTAAGGCCCGGACTGTAT

GGGCATAAGTATCTTCTGGTGTTTGTGGACACGTTCTCTGGCTGGGTGGAAACCTTCCCAACCAAGCATGAGACTGCCAA
AGTTGTGACCAAAAAGCTTCTAGAAGAAATATTTCCAAGGTTTGGAATGCCCCAAGTATTGGGGACTGATAATGGGCCTG
CCTTCGTCTCCCAGGTAAGTCAGTCGGTGGCCAAGCTACTGGGGATTGATTGGAAACTACATTGTGCTTACAGACCCCAG
AGTTCAGGTCAGGTAGAAAGAATGAATAGGACAATCAAGGAGACTTTGACCAAATTAACGCTTGCAACTGGCACTAGAGA
CTGGGTACTCCTACTTCCCTTGGCCCTCTACCGAGCCCGCAACACTCCGGGCCCCCATGGACTCACTCCGTATGAAATCC
TGTATGGGGCGCCCCCGCCCCTTGTTAATTTCCATGATCCTGAAATGTCAAAGTTTACTAATAGCCCCTCTCTCCAAGCT
CACTTACAGGCCCTCCAAGCAGTACAACGAGAGGTCTGGAAGCCTCTGGCCGCTGCCTATCAGGACCAACAAGACCAGCC
TGTGATACCACACCCCTTCCGTGTCGGCGACACCGTGTGGGTACGCCGGCACCAGACTAAGAACTTGGAACCTCGCTGGA
AAGGACCCTACACCGTCCTGCTGACCACCCCCACCGCTCTCAAAGTAGACGGCATCGCTGCGTGGATCCACGCCGCTCAC
GTAAAAGCGGCGACAACCCCTCCGGCCGGAACAGCATCAGGACCGACATGGAAGGTCCAGCGTTCTCAAAACCCCTTAAA
GATAAGATTAACCCGTGGGCCCCCCTGATAGTCCTGGGGATCTTAATAAGGGCAGGAGTATCAGTACCACATGACAGCCC
TCATCAGGTCTTCAATGTTACTTGGAGAGTTACCAACTTAATGACAGGACAAACAGCTAATGCTACCTCCCTCCTGGGGA
CAATGACCGATGCCTTTCCCAAACTGTACTTTGACTTGTGCGATTTAATAGGGGACGACTGGGATGAGACTGGACTCGGG
TGTCGCACTCCCGGGGGAAGAAAAAGGGCAAGAACATTTGACTTCTATGTTTGCCCCGGGCATACTGTACCAACAGGGTG
TGGAGGCCCGAGAGAGGGCTACTGTGGCAAATGGGGCTGTGAGACCACTGGACAGGCATACTGGAAGCCATCATCATCAT
GGGACCTAATTTCCCTTAAGCGAGGAAACACCCCTCGGAATCAGGGCCCCTGTTATGATTCCTCAGCGGTCTCCAGTGGC
ATCCAGGGTGCCACACCGGGGGGTCGATGCAATCCCCTAGTCCTAGAATTCACTGACGCGGGTAAAAAGGCCAGCTGGGA
TGGCCCCAAAGTATGGGGACTAAGACTGTACCGATCCACAGGAACCGACCCGGTGACCCGGTTCTCTTTGACCCGCCAGG
TCCTCAATATAGGGCCCCGCATCCCCATTGGGCCTAATCCCGTGATCACTGACCAGTTACCCCCCTCCCGACCCGTGCAG
ATCATGCTCCCCAGGCCTCCTCAGCCTTCCCCTACAGGCGCAGCCTCTATACAACCTGGGACGGGAGACAGACTGCTGAA
CCTGGTAGATGGAGCCTACCAAGCACTCAACCTCACCAGTCCTGACAAAACCCAAGAGTGCTGGTTGTGTCTGGTATCGG
GACCCCCCTATTACGAAGGGGTTGCCGTCCTAGGTACCTACTCCAACCATACCTCTGCCCCAGCTAACTGCTCCGTGGCC
TCCCAACACAAGCTGACCCTGTCCGAAGTAACCGGACAGGGACTCTGCGTAGGAGCAGTTCCCAAAACCCATCAGGCCCT
GTGTAATACCACCCAGAATACAAGCGACGGGTCCTACTATCTGGCTGCTCCCGCCGGGACCATTTGGGCTTGCAACACCG
GGCTCACTCCCTGCCTATCTACTACTGTACTCAACCTCACCACCGATTACTGTGTCCTGGTTGAGCTCTGGCCAAAGGTG
ACCTACCACTCCCCTGGTTATGTTTATGGCCAGTTTGAGAGAAAAACCAAATATAAAAGAGAGCCGGTGTCATTAACTCT
GGCCCTGCTGTTGGGAGGACTTACTATGGGCGGCATAGCTGCAGGAGTAGGAACAGGGACTACAGCCCTAGTGGCCACCA
AGCAATTCGAGCAGCTCCAGGCAGCCATACATACAGACCTTGGGGCCTTAGAAAAATCAGTCAGTGCCCTAAAAAAGTCT
CTGACCTCGTTGTCTGAGGTGGTCCTACAGAACCGGAGAGGATTAGATCTACTGTTCCTAAAAGAAGGAGGATTATGTGC
TGCCCTAAAAGAAGAATGCTGTTTCTACGCGGACCACACTGGCGTAGTGAGAGATAGCATGGCAAAGCTAAGAGAAAGGT
TAAACCAGAGACAAAAATTGTTCGAATCAGGACAAGGGTGGTTTGAGGGACTGTTTAACAGGTCCCCATGGTTCACGACC
TTGATATCCACCATTATGGGCCCCTTGATAATACTCTTATTAATCCTACTCTTCGGACCCTGTATTCTCAACCGCTTGGT
CCAGTTTGTAAAAGACAGAATTTCGGTGGTGCAGGCCCTGGTTCTGACCCAACAGTATCACCAACTCAAATCAATAGGTC
CAGAAGAAGTGGAATCACGTGAATAAAAGATTTTATTCAGTTTCCAGAAAGAGGGGGGAATGAAAGACCCCACCATAAGG
CTTAGCAAGCTAGCTGCAGTAACGCCATTTTGCAAGGCATGAAAAAGTACCAGAGCTGGGTTCTCAAAAGTTACAAGGAA
GTTCAGTTATAGATTAACAGTTAAAGATCAAGGCTGAATAGCACTGGGACAGGGGCCGAACAGGATATCGGTGGTCAAGC
ACCTGGGCCCCGGCTCAGGGCCAAGAACAGATGGTTCTCAGATAAAGCGGAACCAGCAACAGACACAGAAGCCCCGATAG
ACGTCAGTGTTAGCAGAACTAGCTTCACTGATTTAGAAAAATAGAGGTGCACAGTGCTCTGGTCACTCCTTGAACCTGTG
TGTCTGCCAATGTTCTGACCAGATATGTGCCCATTGCTGAACCTTCATTAGACTCTTTCCTTGTACCCCTCCCCTACCCA
TTTCTTGAAAATAGACATTGTTTAGATCTAAAAAGTCCCACCTCAGTTTCCCCAAATGACCGGGAAGTACCCCAAACCTT
ATTCGAACTAACCAACCAGCTCGCTTCTCGCTTCTGTAACCGCGCTTTTTGCTCCCCAGCCCTAGCCCTATAAAAAGGGT
AAAAACTCCACACTCGGCGCGCCAGTCCTCCGATAGACTGAGTCGCCCGGGTACCCGTGTTCCCAATAAAGCCTCTTGCT
GTTTACATCCGAATCGTGGACTCGCTGATCCTTGGGAGGGTCTCCTCAGATTGATTGACTGCCCACCAGGGGGGTCTTTC
A
>Mpmv12
TGAAAGACCCCACCATAAGGCTTAGCAAGCTAGCTGCAGTAACGCCATTTTGCAAGGCATGAAAAAGTACCCGAGCTGGG
TTCTCAAAAGTTACAAGGAAGTTCAGTTATAGATTAACAGTTAAAGATCAAGGCTGAATAGCACTGGGACAGGGGCCGAA
CAGGATATCGGTGATCAAGCACCTGGGCCCCGGCTCAGGGCCAAGAACAGATGGTTCTCAGATAAAGCGGAACCAGCAAC
AGACACAGAAGCCCCGATAGACGTCAGTGTTAGCAGAACTAGCTTCACTGATTTAGAAAAATAGAGGTGCACAGTGCTCT
GGTCACTCCTTGAACCTGTGTGTCTGCCAATGTTCTGACCAGATATGTGCCCATTGCTGAACCTTCATTAGACTCTTTCC
TTGTACCCCTCCCCTACCCATTTCTTGAAAATAGACATTGTTTAGATCTAAAAAGTCCCACCTCAGTTTCCCCAAATGAC
CGGGAAGTACCCCAAACCTTATTCGAACTAACCAACCAGCTCGCTTCTCGCTTCTGTAACCGCGCTTTTTGCTCCCCAGC
CCTAGCCCTATAAAAAGGGTAAAAACTCCACACTCGGCGCGCCAGTCCTCCGATAGACTGAGTCGCCCGGGTACCCGTGT
TCCCAATAAAGCCTCTTGCTGTTTACATCCGAATCGTGGACTCGCTGATCCTTGGGAGGGTCTCCTCAGATTGATTGACT
GCCCACCTGGGGGGGTCTTTCATTTGGAGGTCCCACCGAGATCAGGAGACCCCTGCCCAGGGACCACCGACCCCCGCCG
GGAGGTAAGCTGGCCAGCGGTCGTTTCGTGTCTGTCTCTGTCTCCGTGCGTGTTTGTGCCGGCATCTAATGTTTGCGCCT
GCGTCTGTACTAGTTGGCTAACTAGATCTGTATCTGGCGGTTCCGCGGAAGAACTGACGAGTTCGTATTCCCGGCCGCAG

CCCCTGGGAGACGTCCCAGCGGCCTCGGGGGCCCGTTTTGTGGCCCATTCTGTATCAGTTAACCTACCCGAGTCGGACTT
TTTGGAGCTCCGCCACTGTACGTGGCTTTGTTGGGGGACGAGAGACAGAGACACTTCCCGCCCCCGTCTGGATTTTTGCT
TTCGGTTTTACGCCGAAACCGCGCTGCGCGTCTGATTTGTTTTATTGCTCTTTTGTTCTTCGTTAGTTTTTTCTGTCTTT
AAGTGTTTTCAAGATCATGGGACAGACCGTAACTACCCCTCTGAGTCTAACCTTGCAGCACTGGGGAGATGTCCAGCGCA
TTGCATCCAACCAGTCTGTGGATGTCAGGAAGAGGCGCTGGATTACCTTCTGTTCCGCCGAATGGCCAACTTTCAATGTA
GGATGGCCTCAGGATGGTACTTTCAATTTAAGTATTATCTCTCAGGTTAAGTCTAGAGTGTTTTGTCCTGGTCCCCACGG
ACACCCGGATCAGGTCCCATATATCGTTACCTGGGAGGCACTTGCCTATGACCCCCCTCCGTGGGTCAAACCGTTTGTTT
CTCCTAAACCTCCTCCCTTGCCGACAGCTCCCGTCCTCCCGCCCGGTCCTTCTGCGCAACCTCCGTCCCGATCTGCCCTT
TACCCTGCCCTTACCCCCTCTATAAAGTCCAAACCTCCTAAGCCCCAGGTTCTCCCTGATAGCGGCGGACCTCTCATTGA
CCTTCTCACAGAGGACCCCCCGCCGTACGGAGCACAACCTTCCTCCTCTGCCAGAGAGAACGATGAAGAAGAGGCGGCCA
CCACCTCCGAGGTTTCCCCCCCTTCTCCCATGGCGTCTCGACTGCGGGGAAGGAGAGACCCTCCCGCAGCGGACTCCACC
TCCTCCCAGGCATTCCCACTCCGTATGGGGGGAGATGGCCAGCTTCAGTACTGGCCGTTTTCCTCCTCTGACTTATATAA
TTGGAAAAATAATAACCCTTCCTTTTCTGAAGATCCAGGTAAATTGACGGCCTTGATTGAGTCCGTCCTCATCACCCACC
AGCCCACCTGGGACGACTGTCAGCAGTTGTTGGGGACCCTGCTGACCGGAGAAGAAAAGCAGCGGGTGCTCCTAGAGGCT
AGAAAGGCAGTCCGGGGCAATGATGGACGCCCCACTCAGTTGCCTAATGAAATCAATGCTGCCTTTCCCCTTGAGCGCCC
TGATTGGGATTACACCACTACAGAAGGTAGGAACCACCTAGTCCTCTATCGCCAGTTGCTCTTAGCGGGTCTCCAAAACG
CGGGCAGAAGCCCCACCAATTTGGCCAAGGTAAAAGGGATAACCCAGGGACCTAATGAGTCTCCCTCAGCCTTTTTAGAG
AGACTCAAGGAGGCCTATCGCAGGTACACTCCTTATGACCCTGAGGACCCAGGGCAAGAAACCAATGTGTCTATGTCATT
CATCTGGCAGTCTGCCCCGGATATCGGGCGAAAGTTAGAGCGGTTAGAAGATTTTAAGAGCAAGACCTTAGGAGACTTAG
TGAGGGAAGCTGAAAAGATCTTTAATAAACGAGAAACCCCGGAAGAAAGAGAGGAACGTATCAGGAGAGAAACAGAGGAA
AAAGAAGAACGCCGTAGGGCAGAAGATGAGCAGAAAGAGAAAGAAAGGGACCGCAGAAGACATAGAGAGATGAGCAAGCT
CTTGGCCACTGTAGTTATTGGTCAGAGACAGGATAGACAGGGGGGAGAGCGGAAGAGGCCCCAACTTGATAAGGATCAAT
GCGCCTACTGCAAAGAAAAGGGACACTGGGCTAAAGACTGCCCAAAGAAGCCACGAGGGCCCCGAGGACCGAGGCCCCAG
ACCTCCCTCCTGACCTTAGGTGACTAGGGAGGTCAGGGTCAGGAGCCCCCCCCTGAACCCAGGATAACCCTCAAAGTCGG
GGGGCAACCCGTCACCTTCCTGGTGGATACTGGGGCCCAACACTCCGTGCTGACCCAAAATCCTGGACCCCTAAGTGACA
AGTCTGCCTGGGTCCAAGGGGCTACTGGAGGAAAGCGGTATCGCTGGACCACGGATCGCAAAGTGCATCTAGCTACCGGT
AAGGTCACCCACTCTTTCCTCCATGTACCAGACTGCCCCTATCCTCTGCTAGGAAGAGATTTGCTGACTAAACTAAAAGC
CCAAATCCACTTTGAGGGATCAGGAGCTCAGGTTGTGGGACCAATGGGACAGCCCCTGCAAGTGCTGACCCTAAACATAG
AAGATGAGTATCGGCTACATGAGACCTCAAAAGAGCCGGATGTTTCTCTAGGGTCCACATGGCTTTCTGATTTTCCCCAG
GCCTGGGCGGAAACCGGGGGCATGGGACTGGCAGTTCGCCAAGCTCCTCTGATCATACCTCTGAAGGCAACCTCTACCCC
CGTGTCCATAAAACAATACCCCATGTCACAAGAAGCCAGACTGGGGATCAAGCCCCACATACAGAGACTGTTGGACCAGG
GAATACTGGTACCCTGCCAGTCCCCCTGGAACACGCCCCTGCTACCCGTTAAGAAACCAGGGACTAATGATTACAGGCCT
GTCCAAGATCTGAGAGAAGTCAACAAGCGGGTGGAAGACATCCACCCCACCGTGCCCAACCCTTACAACCTCTTGAGCGG
GCTCCCACCGTCCCACCAGTGGTACACTGTGCTTGACTTAAAGGATGCCTTTTTCTGCCTGAGACTCCACCCCACCAGTC
AGCCTCTCTTCGCCTTTGAGTGGAGAGACCCAGAGATGGGAATCTCAGGACAATTAACCTGGACCAGACTCCCACAGGGT
TTCAAAAACAGTCCCACCCTGTTTGATGAGGCACTGCACAGAGACCTAGCAGGCTTCCGGATCCAGCACCCAGACTTGAT
CCTGCTACAGTACGTGGATGACTTACTGCTGGCCGCCACTTCTGAGCTCGACTGCCAACAAGGTACTCGGGCCCTGTTAC
AAACCCTAGGGGACCTCGGGTATCGGGCCTCGGCCAAGAAAGCCCAAATTTGCCAGAAACAGGTCAAGTATCTGGGGTAT
CTTCTAAAAGAGGGTCAGAGATGGCTGACTGAGGCCAGAAAAGAGACTGTGATGGGGCAGCCTATTCCGAAGACCCCTCG
ACAACTAAGGGAGTTCCTAGGGACGGCAGGCTTCTGTCGCCTCTGGATCCCTGGGTTTGCAGAAATGGCAGCCCCCTTGT
ATCCTCTCACCAAAACGGGGACTCTGTTTAATTGGGGCCCAGACCAGCAAAAGGCCTATCAAGAAATCAAACAGGCTCTT
CTAACTGCCCCAGCCCTGGGGTTGCCAGATTTGACTAAGCCCTTTGAACTCTTTGTCGACGAGAAGCAGGGCTACGCCAA
AGGCGTCCTAACGCAAAAACTGGGACCTTGGCGTCGGCCGGTGGCCTACCTGTCCAAAAAGCTAGACCCAGTGGCAGCTG
GGTGGCCCCCTTGCCTACGGATGGTAGCAGCCATTGCCGTTCTGACAAAGGATGCAGGCAAGCTAACTATGGGACAGCCG
CTAGTCATTCTGGCCCCCCATGCGGTAGAAGCACTGGTCAAACAACCCCCTGACCGCTGGCTATCCAATGCTCGCATGAC
CCACTATCAGGCAATGCTCCTAGATACGGACCGAGTTCAGTTCGGACCGGTGGTAGCCTTAAACCCGGCCACGTTGCTCC
CCTTGCCGGGAAAAGAGACCCCCCACGACTGCCTCGAGATCTTGGCTGAGACACACGGCACCAGACCGGACCTCACGGAC
CAGCCCCTCCCAAATGCCGACCACACCTGGTATACAGATGGAAGCAGCTTCCTGCAGGAGGGGCAACGTAAGGCTGGAGC
AGCGGTGACCACCGAGACCGAGGTAATCTGGGCCAAGGCGTTGCCAGCCGGGACATCCGCCCAGCGAGCTGAACTAATAG
CACTCACCCAGGCCCTAAAGATGGCAGAAGGTAAGAAGCTAAATGTTTATACTGATAGCCGCTATGCCTTTGCTACCGCC
CATGTCCATGGAGAAATATATAGGAGACGTGGGTTGCTCACCTCAGAAGGCAAGGAGATCAAGAACAAGGGCGAAATCTT
GGCCTTACTGAAAGCTCTCTTTCTGCCCAAAAGACTCAGTATAATTCACTGCCCAGGACATCAGAAAGGCAATAGTGCTG
AAGCTAAAGGCAACCGAATGGCGGACCAGGCAGCCCGGGAAGCAGCCATGGGGACTGACACAAAGGCCTCCTCACTTCTC
ATAGAGACCTCAACCCCGTACACTCCAGACTTCTTCCATTATACTGAAACAGATATAAAGAACCTACGAGAGTTGGGAGC
CACATATAATAGAGAAAAAAAATATTGGGTCCTGCAAGGTAAACCTGTGATGCCTGACCAGTTCACCTTTGAATTATTAG
ACTTCCTTCACCAGCTCACCCACCTTAGCTATCAGAAGATGAGGGCACTTCTAGACAGAAAAGAAAGCCCCTATTACATG
CTAAATAAAGATAAGATCCTCCACGAGGTGGCGGAATCATGCCAAGCCTGTGTCCAAGTAAATGCCAGTAAAGCTAAGGT

CGGTCCCGGGGTGCGAGTAAGAGGACATCGACCAGGCACCCATTGGGAAATTGACTTTACTGAAGTAAGGCCCGGACTGT
ATGGGCATAAGTATCTTCTGGTGTTTGTGGACACGTTCTCTGGCTGGGTGGAAGCCTTCCCAACCAAGCATGAGACTGCC
AAAGTTGTGACCAAAAAGCTTCTAGAAGAAATATTTCCAAGGTTTGGAATGCCCCAAGTATTGGGGACTGATAATGGGCC
TGCCTTCGTCTCCCAGGTAAGTCAGTCGGTGGCCAAGCTACTGGGGATTGATTGGAAACTACATTGTGCTTACAGACCCC
AGAGTTCAGGTCAGGTAGAAAGAATGAATAGGACAATCAAGGAGACTTTGACCAAATTAACGCTTGCAACTGGCACTAGA
GACTGGGTACTCCTACTTCCCTTGGCCCTCTACCGAGCCCGCAACACTCCGGGCCCCCATGGACTCACTCCGTATGAAAT
CCTGTATGGGGCGCCCCCGCCCCTTGTTAATTTCCATGATCCTGAAATGTCAAAGTTTACTAATAGCCCCTCTCTCCAAG
CTCACTTACAGGCCCTCCAAGCGGTACAACGAGAGGTCTGGAAGCCTCTGGCCGCTGCCTATCAGGACCAACAAGACCAG
CCTGTGATACCACACCCCTTCCGTGTCGGCGACACCGTGTGGGTACGCCGGCACCAGACTAAGAACTTGGAACCTCGCTG
GAAAGGACCCTACACCGTCCTGCTGACCACCCCCACCGCTCTCAAAGTAGACGGCATCGCTGCGTGGATCCACGCCGCTC
ACGTAAAAGCGGCGACAACCCCTCCGGCCGGAACAGCATCAGGACCGACATGGAAGGTCCAGCGTTCTCAAAACCCTTTA
AAGATAAGATTAACCCGTGGGCCCCCCTGATAGTCCTGGGGATCTTAATAAGGGCAGGAGTATCAGTACCACATGACAGC
CCTCATCAGGTCTTCAATGTTACTTGGAGAGTTACCAACTTAATGACAGGACAAACAGCTAATGCTACCTCCCTCCTGGG
GACAATGACCGATGCCTTTCCCAAACTGTACTTTGACTTGTGCGACTTAATAGGGGACGACTGGGATGAGACTGGACTCG
GGTGTCGCACTCCCGGGGGAAGAAAAAGGGCAAGAACATTTGACTTCTATGTTTGCCCCGGGCATACTGTACCAACAGGG
TGTGGAGGCCCGAGAGAGGGCTACTGTGGCAAATGGGGCTGTGAGACCACTGGACAGGCATACTGGAAGCCATCATCATC
ATGGGACCTAATTTCCCTTAAGCGAGGAAACACCCCTCGGAATCAGGGCCCCTGTTATGATTCCTCAGCGGTCTCCAGTG
GCATCCAGGGTGCCACACCGGGGGGTCGATGCAATCCCCTAGTCCTAGAATTCACTGACGCGGGTAAAAAGGCCAGCTGG
GATGGCCCCAAAGTATGGGGACTAAGACTGTACCGATCCACAGGAACCGACCCGGTGACCCGGTTCTCTTTGACCCGCCA
GGTCCTCAATATAGGGCCCCGCATCCCCATTGGGCCTAATCCCGTGATCACTGACCAGTTACCCCCCTCCCGACCCGTGC
AGATCATGCTCCCCAGGCCTCCTCAGCCTTCCCCTACAGGCGCAGCCTCTATACAACCTGGGACGGGAGACAGACTGCTG
AACCTGGTAGATGGAGCCTACCAAGCACTCAACCTCACCAGTCCTGACAAAACCCAAGAGTGCTGGTTGTGTCTGGTATC
GGGACCCCCCTATTACGAAGGGGTTGCCGTCCTAGGTACCTACTCCAACCATACCTCTGCCCCAGCTAACTGCTCCGTGG
CCTCCCAACACAAGCTGACCCTGTCCGAAGTAACCGGACAGGGACTCTGCGTAGGAGCAGTTCCCAAAACCCATCAGGCC
CTGTGTAATACCACCCAGAATACAAGCGACGGGTCCTACTATCTGGCTGCTCCCGCCGGGACCATTTGGGCTTGCAACAC
CGGGCTCACTCCCTGCCTATCTACTACTGTACTCAACCTCACCACCGATTACTGTGTCCTGGTTGAGCTCTGGCCAAAGG
TGACCTACCACGCCCCTGGTTATGTTTATGGCCAGTTTGAGAGAAAAACCAAATATAAAAGAGAGCCGGTGTCATTAACT
CTGGCCCTGCTGTTGGGAGGACTTACTATGGGCGGCATAGCTGCAGGAGTAGGAACAGGGACTACAGCCCTAGTGGCCAC
CAAGCAATTCGAGCAGCTCCAGGCAGCCATACATACAGACCTTGGGGCCTTAGAAAAATCAGTCAGTGCCCTAGAAAAGT
CTCTGACCTCGTTGTCTGAGGTGGTCCTACAGAACCGGAGAAGATTAGATCTACTGTTCCTAAAAGAAGGAGGATTATGT
GCTGCCCTAAAAGAAGAATGCTGTTTCTACGCGGACCACACTGGCGTAGTGAGAGATAGCATGGCAAAGCTAAGAGAAAG
GTTAAACCAGAGACAAAAATTGTTCGAATCAGGACAAGGGTGGTTTGAGGGACTGTTTAACAGGTCCCCATGGTTCACGA
CCTTGATATCCACCATTATGGGCCCCTTGATAATACTCTTATTAATCCTACTCTTCGGACCCTGTATTCTCAACCGCTTG
GTCCAGTTTGTAAAAGACAGAATTTCGGTGGTGCAGACCCTGGTTCTGACCCAACAGTATCATCAACTCAAATCAATAGG
TCCAGAAGAAGTGGAATCACGTGAATAAAAGATTTTATTCAGTTTCCAGAAAGAGGGGGGAATGAAAGACCCCACCATAA
GGCTTAGCAAGCTAGCTGCAGTAACGCCATTTTGCAAGGCATGAAAAAGTACCCGAGCTGGGTTCTCAAAAGTTACAAGG
AAGTTCAGTTATAGATTAACAGTTAAAGATCAAGGCTGAATAGCACTGGGACAGGGGCCGAACAGGATATCGGTGATCAA
GCACCTGGGCCCCGGCTCAGGGCCAAGAACAGATGGTTCTCAGATAAAGCGGAACCAGCAACAGACACAGAAGCCCCGAT
AGACGTCAGTGTTAGCAGAACTAGCTTCACTGATTTAGAAAAATAGAGGTGCACAGTGCTCTGGTCACTCCTTGAACCTG
TGTGTCTGCCAATGTTCTGACCAGATATGTGCCCATTGCTGAACCTTCATTAGACTCTTTCCTTGTACCCCTCCCCTACC
CATTTCTTGAAAATAGACATTGTTTAGATCTAAAAAGTCCCACCTCAGTTTCCCCAAATGACCGGGAAGTACCCCAAACC
TTATTCGAACTAACCAACCAGCTCGCTTCTCGCTTCTGTAACCGCGCTTTTTGCTCCCCAGCCCTAGCCCTATAAAAAGG
GTAAAAACTCCACACTCGGCGCGCCAGTCCTCCGATAGACTGAGTCGCCCGGGTACCCGTGTTCCCAATAAAGCCTCTTG
CTGTTTACATCCGAATCGTGGACTCGCTGATCCTTGGGAGGGTCTCCTCAGATTGATTGACTGCCCACCTGGGGGGGGTC
TTTCA
>Mpmv13
TGAAAGACCCCACCATAAGGCTTAGCAAGCTAGCTGCAGTAACGCCATTTTGCAAGGCATGAAAAAGTACCAGAGCTGGG
TTCTCAAAAGTTACAAGGAAGTTCAGTTATAGATTAACAGTTAAAGATCAAGGCTGAATAGCACTGGGACAGGGGCCGAA
CAGGATATCGGTGGTCAAGCACCTGGGCCCCGGCTCAGGGCCAAGAACAGATGGTTCTCAGATAAAGCGGAACCAGCAAC
AGACACAGAAGCCCCGATAGACGTCAGTGTTAGCAGAACTAGCTTCACTGATTTAGAAAAATAGAGGTGCACAGTGCTCT
GGTCACTCCTTGAACCTGTGTGTCTGCCAATGTTCTGACCAGATATGTGCCCATTGCTGAACCTTCATTAGACTCTTTCC
TTGTACCCCTCCCCTACCCATTTCTTGAAAATAGACATTGTTTAGATCTAAAAAGTCCCACCTCAGTTTCCCCAAATGAC
CGGGAAGTACCCCAAACCTTATTCGAACTAACCAACCAGCTCGCTTCTCGCTTCTGTAACCGCGCTTTTTGCTCCCCAGC
CCTAGCCCTATAAAAAGGGTAAAAACTCCACACTCGGCGCGCCAGTCCTCCGATAGACTGAGTCGCCCGGGTACCCGTGT
TCCCAATAAAGCCTCTTGCTGTTTACATCCGAATCGTGGACTCGCTGATCCTTGGGAGGGTCTCCTCAGATTGATTGACT
GCCCACCTGGGGGTCTTTCATTTGGAGGTCCCACCGAGATCAGGAGACCCCTGCCCAGGGACCACCGACCCCCGCCGGG
AGGTAAGCTGGCCAGCGGTCGTTTCGTGTCTGTCTCTGTCTCCGTGCGTGTTTGTGCCGGCATCTAATGTTTGCGCCTGC

GTCTGTACTAGTTGGCTAACTAGATCTGTATCTGGCGGTTCCGCGGAAGAACTGACGAGTTCGTATTCCCGGCCGCAGCC
CCTGGGAGACGTCCCAGCGGCCTCGGGGGCCCGTTTTGTGGCCCATTCTGTATCAGTTAACCTACCCGAGTCGGACTTTT
TGGAGCTCCGCCACTGTACGTGGCTTTGTTGGGGGACGAGAGACAGAGACACTTCCCGCCCCCGTCTGGATTTTTGCTTT
CGGTTTTACGCCGAAACCGCGCTGCGCGTCTGATTTGTTTTATTGCTCTTTTGTTCTTCGTTAGTTTTTTCTGTCTTTAA
GTGTTTTCAAGATCATGGGACAGACCGTAACTACCCCTCTGAGTCTAACCTTGCAGCACTGGGGAGATGTCCAGCGCATT
GCATCCAACCAGTCTGTGGATGTCAGGAAGAGGCGCTGGATTACCTTCTGTTCCGCCGAATGGCCAACTTTCAATGTAGG
ATGGCCTCAGGATGGTACTTTCAATTTAAGTATTATCTCTCAGGTTAAGTCTAGAGTGTTTTGTCCTGGTCCCCACGGAC
ACCCGGATCAGGTCCCATATATCGTTACCTGGGAGGCACTTGCCTATGACCCCCCTCCGTGGGTCAAACCGTTTGTTTCT
CCTAAACCTCCTCCCTTGCCGACAGCTCCCGTCCTCCCGCCCGGTCCTTCTGCGCAACCTCCGTCCCGATCTGCCCTTTA
CCCTGCCCTTACCCCCTCTATAAAGTCCAAACCTCCTAAGCCCCAGGTTCTCCCTGATAGCGGCGGACCTCTCATTGACC
TTCTCACAGAGGACCCCCCGCCGTACGGAGCACAACCTTCCTCCTCTGCCAGAGAGAACGATGAAGAAGAGGCGGCCACC
ACCTCCGAGGTTTCCCCCCCTTCTCCCATGGCGTCTCGACTGCGGGGAAGGAGAGACCCTCCCGCAGCGGACTCCACCTC
CTCCCAGGCATTCCCACTCCGTATGGGGGGAGATGGCCAGCTTCAGTACTGGCCGTTTTCCTCCTCTGACTTATATAATT
GGAAAAATAATAACCCTTCCTTTTCTGAAGATCCAGGTAAATTGACGGCCTTGATTGAGTCCGTCCTCATCACCCACCAG
CCCACCTGGGACGACTGTCAGCAGTTGTTGGGGACCCTGCTGACCGGAGAAGAAAAGCAGCGGGTGCTCCTAGAGGCTAG
AAAGGCAGTCCGGGGCAATGATGGACGCCCCACTCAGTTGCCTAATGAAATCAATGCTGCCTTTCCCCTTGAGCGCCCTG
ATTGGGATTACACCACTACAGAAGGTAGGAACCACCTAGTCCTCTATCGCCAGTTGCTCTTAGCGGGTCTCCAAAACGCG
GGCAGAAGCCCCACCAATTTGGCCAAGGTAAAAGGGATAACCCAGGGACCTAATGAGTCTCCCTCAGCCTTTTTAGAGAG
ACTCAAGGAGGCCTATCGCAGGTACACTCCTTATGACCCTGAGGACCCAGGGCAAGAAACCAATGTGTCTATGTCATTCA
TCTGGCAGTCTGCCCCGGATATCGGGCGAAAGTTAGAGCGGTTAGAAGATTTAAAGAGCAAGACCTTAGGAGACTTAGTG
AGGGAAGCTGAAAAGATCTTTAATAAACGAGAAACCCCGGAAGAAAGAGAGGAACGTATCAGGAGAGAAACAGAGGAAAA
AGAAAAACGCCGTAGGGCAGAGGATGAGCAGAAAGAGAAAGAAAGGGACCGCAGAAGACATAGAGAGATGAGCAAGCTCT
TGGCCACTGTAGTTATTGGTCAGAGACAGGATAGACAAGGGGGAGAGCGGAAGAGGCCCCAACTTGATAAGGATCAATGC
GCCTACTGCAAAGAAAAGGGACACTGGGCTAAAGACTGCCCAAAGAAGCCACGAGGGCCCCGAGGACCGAGGCCCCAGAC
CTCCCTCCTGACCTTAGGTGACTAGAGAGGTCAGGGTCAGGAGCCCCCCCTGAACCCAGGATAACCCTCAAAGTCGGGG
GGCAACCCGTCACCTTCCTGGTAGATACTGGGGCCCAACACTCCGTGCTGACCCAAAATCCTGGACCCCTAAGTGACAAG
TCTGCCTGGGTCCAAGGGGCTACTGGAGGAAAGCGGTATCGCTGGACCACGGATCGCAAAGTGCATCTAGCTACCGGTAA
GGTCACCCACTCTTTCCTCCATGTACCAGACTGCCCCTATCCTCTGCTAGGAAGAAATTTGCTGACTAAACTAAAAGCCC
AAATCCACTTTGAGGGATCAGGAGCTCAGGTTGTGGGACCAATGGGACAGCCCCTGCAAGTGCTGACCCTAAACATAGAA
GATGAGTATCGGCTACATGAGACCTCAAAAGAGCCGGATGTTTCTCTAGGGTCCACATGGCTTTCTGATTTTCCCCAGGC
CTGGGCGGAAACCGGGGGCATGGGACTGGCAGTTCGCCAAGCTCCTCTGATCATACCTCTGAAGGCAACCTCTACCCCCG
TGTCCATAAAACAATACCCCATGTCACAAGAAGCCAGACTGGGGATCAAGCCCCACATACAGAGACTGTTGGACCAGGGA
ATACTGGTACCCTGCCAGTCCCCCTGGAACACGCCCCTGCTACCCGTTAAGAAACCAGGGACTAATGATTACAGGCCTGT
CCAAGATCTGAGAGAAGTCAACAAGCGGGTGGAAGACATCCACCCCACCGTGCCCAACCCTTACAACCTCTTGAGCGGGC
TCCCACCGTCCCACCAGTGGTACACTGTGCTTGACTTAAAGGATGCCTTTTTCTGCCTGAGACTCCACCCCACCAGTCAG
CCTCTCTTCGCCTTTGAGTGGAGAGACCCAGAGATGGGAATCTCAGGACAATTAACCTGGACCAGACTCCCACAGGGTTT
CAAAAACAGTCCCACCCTGTTTGATGAGGCACTGCACAGAGACCTAGCAGGCTTCCGGATCCAGCACCCAGACTTGATCC
TGCTACAGTACGTGGATGACTTACTGCTGGCCGCCACTTCTGAGCTCGACTGCCAACAAGGTACTCGGGCCCTGTTACAA
ACCCTAGGGGACCTCGGGTATCGGGCCTCGGCCAAGAAAGCCCAAATTTGCCAGAAACAGGTCAAGTATCTGGGGTATCT
TCTAAAAGAGGGTCAGAGATGGCTGACTGAGGCCAGAAAAGAGACTGTGATGGGGCAGCCTATTCCGAAGACCCCTCGAC
AACTAAGGGAGTTCCTAGGGACGGCAGGCTTCTGTCGCCTCTGGATCCCTGGGTTTGCAGAAATGGCAGCCCCCTTGTAC
CCTCTCACCAAAACGGGGACTCTGTTTAATTGGGGCCCAGACCAGCAAAAGGCCTATCAAGAAATCAAACAGGCTCTTCT
AACTGCCCCAGCCCTGGGGTTGCCAGATTTGACTAAGCCCTTTGAACTCTTTGTCGACGAAAAGCAGGGCTACGCCAAAG
GCGTCCTAACGCAAAAACTGGGACCTTGGCGTCGGCCGGTGGCCTACCTGTCCAAAAAGCTAGACCCAGTGGCAGCTGGG
TGGCCCCCTTGCCTACGGATGGTAGCAGCCATTGCCGTTCTGACAAAGGATGCAGGCAAGCTAACTATGGGACAGCCGCT
AGTCATTCTGGCCCCCCATGCGGTAAAAGCACTGGTCAAACAACCCCCTGACCGCTGGCTATCCAATGCTCGCATGACCC
ACTATCAGGCAATGCTCCTAGATACGGACCGAGTTCAGTTCGGACTGGTGGTAGCCTTAAACCCGGCCACGTTGCTCCCC
TTGCCGGGAAAAGAGACCCCCCACGACTGCCTCGAGATCTTGGCTGAGACACACGGCACCAGACCGGACCTCACGGACCA
GCCCCTCCCAAATGCCGACCACACCTGGTATACAGATGGAAGCAGCTTCCTGCAGGAGGGGCAACGTAAGGCTGGAGCAG
CGGTGACCACCGAGACCGAGGTAATCTGGGCCAAGGCGTTGCCAGCCGGGACATCCGCCCAGCGAGCTGAACTAATAGCA
CTCACCCAGGCCCTAAAGATGGCAGAAGGTAAGAAGCTAAATGTTTATACTGATAGCCGCTATGCCTTTGCTACCGCCCA
TGTCCATGGAGAAATATATAGGAGACGTGGGTTGCTCACCTCAGAAGGCAAGGAGATCAAGAACAAGGGCGAAATCTTGG
CCTTACTGAAAGCTCTCTTTCTGCCCAAAAGACTCAGTATAATTCACTGCCCAGGACATCAGAAAGGCAATAGTGCTGAA
GCTAAAGGCAACCGAATGGCGGACCAGGCAGCCCGGGAAGCAGCCATGGGGACTGACACAAAGGCCTCCTCACTTCTCAT
AGAGACCTCAACCCCGTACACTCCAGACTTCTTCCATTATACTGAAACAGATATAAAGAACCTACGAGAGTTGGGAGCCA
CATATGATAGAGAGAAAAAATATTGGGTCCTGCAAGGTAAACCTGTGATGCCTGACCAGTTCACCTTTGAATTATTAGAC
TTCCTTCACCAGCTCACCCACCTTAGCTATCAGAAGATGAGGGCACTTCTAGACAGAAAAGAAAGCCCCTATTACATGCT

AAATAAAGATAAGATCCTCCACGAGGTGGCGGAATCATGCCAAGCCTGTGTCCAAGTAAATGCCAGTAAAGCTAAGGTCG
GTCCCGGGGTGCGAGTAAGAGGACATCGACCAGGCACCCATTGGGAAATTGACTTTACTGAAGTAAGGCCCGGACTGTAT
GGGCATAAGTATCTTCTGGTGTTTGTGGACACGTTCTCTGGCTGGGTGGAAGCCTTCCCAACCAAGCATGAGACTGCCAA
AGTTGTGACCAAAAAGCTTCTAGAAGAAATATTTCCAAGGTTTAGAATGCCCCAAGTATTGGGGACTGATAATGGGCCTG
CCTTCGTCTCCCAGGTAAGTCAGTCGGTGGCCAAGCTACTGGGGATTGATTGGAAACTACATTGTGCTTACAGACCCCAG
AGTTCAGGTCAGGTAGAAAGAATGAATAGGACAATCAAGGAGACTTTGACCAAATTAACGCTTGCAACTGGCACTAGAGA
CTGGGTACTCCTACTTCCCTTGGCCCTCTACCGAGCCCGCAACACTCCGGGCCCCCATGGACTCACTCCGTATGAAATCC
TGTATGGGGCGCCCCCGCCCCTTGTTAATTTCCATGATCCTGAAATGTCAAAGTTTACTAATAGCCCCTCTCTCCAAGCT
CACTTACAGGCCCTCCAAGCAGTACAACGAGAGGTCTGGAAGCCTCTGGCCGCTGCCTATCAGGACCAACAAGACCAGCC
TGTGATACCACACCCCTTCCGTGTCGGCGACACCGTGTGGGTACGCCGGCACCAGACTAAGAACTTGGAACCTCGCTGAA
AAGGACCCTACACCGTCCTGCTGACCACCCCCACCGCTCTCAAAGTAGACGGCATCGCTGCGTGGATCCACGCCGCTCAC
GTAAAAGCGGCGACAACCCCTCCGGCCGGAACAGCATCAGGACCGACATGGAAGGTCCAGCGTTCTCAAAACCCCTTAAA
GATAAGATTAACCCGTGGGCCCCCCTGATAGTCCTGGGGATCTTAATAAGGGCAGGAGTATCAGTACCACATGACAGCCC
TCATCAGGTCTTCAATGTTACTTGGAGAGTTACCAACTTAATGACAGGACAAACAGCTAATGCTACCTCCCTCCTGGGGA
CAATGACCGATGCCTTTCCCAAACTGTACTTTGACTTGTGCGATTTAATAGGGGACGACTGGGATGAGACTGGACTCGGG
TGTCGCACTCCCGGGGAAAAAAAAAGGGCAAGAACATTTGACTTCTATGTTTGCCCCGGGCATACTGTATCAACAGGGTG
TGGAGGCCCGAGAGAGGGCTACTGTGGCAAATGGGGCTGTGAGACCACTGGACAGGCATACTGGAAGCCATCATCATCAT
GGGACCTAATTTCCCTTAAGCGAGGAAACACCCCTCGGAATCAGGGCCCCTGTTATGATTCCTCAGCGGTCTCCAGTGGC
ATCCAGGGTGCCACACCGGGGGGTCGATGCAATCCCCTAGTCCTAGAATTCACTGACGCGGGTAAAAAGGCCAGCTGGGA
TGGCCCCAAAGTATGGGGACTAAGACTGTACCGATCCACAGGAACCGACCCGGTGACCCGGTTCTCTTTGACCCGCCAGG
TCCTCAATATAGGGCCCCGCATCCCCATTGGGCCTAATCCCGTGATCACTGACCAGTTACCCCCCTCCCGACCCGTGCAG
ATCATGCTCCCCAGGCCTCCTCAGCCTTCCCCTACAGGCGCAGCCTCTATACAACCTGGGACGGGAGACAGACTGCTGAA
CCTGGTAGATGGAGCCTACCAAGCACTCAACCTCACCAGTCCTGACAAAACCCAAGAGTGCTGGTTGTGTCTGGTATCGG
GACCCCCCTATTACGAAGGGGTTGCCGTCCTAGGTACCTACTCCAACCATACCTCTGCCCCAGCTAACTGCTCCGTGGCC
TCCCAACACAAGCTGACCCTGTCCGAAGTAACCGGACAGGGACTCTGCGTAGGAGCAGTTCCCAAAACCCATCAGGCCCT
GTGTAATACCACCCAGAATACAAGCGACGGGTCCTACTATCTGGCTGCTCCCGCCGGGACCATTTGGGCTTGCAACACCG
GGCTCACTCCCTGCCTATCTACTACTGTACTCAACCTCACCACCGATTACTGTGTCCTGGTTGAGCTCTGGCCAAAGGTG
ACCTACCACTCCCCTGGTTATGTTTATGGCCAGTTTGAGAGAAAAACCAAATATAAAAGAGAGCCGGTGTCATTAACTCT
GGCCCTGCTGTTGGGAGGACTTACTATGGGCGGCATAGCCTCAGCAGCTTCATGCACTGCACTGACTGGAGTCAGTGCCC
TAGAAAAGTCTCTGACCTCGTTGTCTGAGGTGGTCCTACAGAACCGGAGAGGATTAGATCTACTGTTCCTAAAAAAAGGA
AGATTATGTGCTGCCCTAAAAGAAGAATGCTGTTTCTACGCGGACCACACTGGCGTAGTGAGAGATAGCATGGCAAAGCT
AAGAGAAAGGTTAAACCAGAGACAAAAATTGTTCGAATCAGGACAAGGGTGGTTTGAGGGACTGTTTAACAGGTCCCCAT
GGTTCACGACCTTGATATCCACCATTATGGGCCCCTTGATAATACTCTTATTAATCCTACTCTTCGGACCCTGTATTCTC
AACCGCTTGGTCCAGTTTGTAAAAGACAGAATTTCGGTGGTGCAGGCCCTGGTTCTGACCCAACAGTATCACCAACTCAA
ATCAATAGGTCCAGAAAAAGTGGAATCACGTGAATAAAAGATTTTATTCAGTTTCCAGAAAGAGGGGGGAATGAAAGACC
CCACCATAAGGCTTAGCAAGCTAGCTGCAGTAACGCCATTTTGCAAGGCATGAAAAAGTACCAGAGCTGGGTTCTCAAAA
GTTACAAGGAAGTTCAGTTATAGATTAACAGTTAAAGATCAAGGCTGAATAGCACTGGGACAGGGGCCGAACAGGATATC
GGTGGTCAAGCACCTGGGCCCCGGCTCAGGGCCAAGAACAGATGGTTCTCAGATAAAGCGGAACCAGCAACAGACACAGA
AGCCCCGATAGACGTCAGTGTTAGCAGAACTAGCTTCACTGATTTAGAAAAATAGAGGTGCACAGTGCTCTGGTCACTCC
TTGAACCTGTGTGTCTGCCAATGTTCTGACCAGATATGTGCCCATTGCTGAACCTTCATTAGACTCTTTCCTTGTACCCC
TCCCCTACCCATTTCTTGAAAATAGACATTGTTTAGATCTAAAAAGTCCCACCTCAGTTTCCCCAAATGACCGGGAAGTA
CCCCAAACCTTATTCGAACTAACCAACCAGCTCGCTTCTCGCTTCTGTAACCGCGCTTTTTGCTCCCCAGCCCTAGCCCT
ATAAAAAGGGTAAAAACTCCACACTCGGCGCGCCAGTCCTCCGATAGACTGAGTCGCCCGGGTACCCGTGTTCCCAATAA
AGCCTCTTGCTGTTTACATCCGAATCGTGGACTCGCTGATCCTTGGGAGGGTCTCCTCAGATTGATTGACTGCCCACCTG
GGGGGTCTTTCA
>Mpmv2
TGAAAGACCCCACCATAAGGCTTAGCAAGCTAGCTGTAGTAACGCCATTTTGCAAGGCATGAAAAAGTACCCGAGCTGGG
TTCTCAAAAGTTACAAGGAAGTTCAGTTATAGATTAACAGTTAAAGATCAAGGCTGAATAGCACTGGGACAGGGGCCGAA
CAGGATATCGGTGATCAAGCACCTGGGCCCCGGCTCAGGGCCAAGAACAGATGGTTCTCAGATAAAGCGGAACCAGCAAC
AGACACAGAAGCCCCGATAGACGTCAGTGTTAGCAGAACTAGCTTCACTGATTTAGAAAAATAGAGGTGCACAGTGCTCT
GGTCACTCCTTGAACCTGTGTGTCTGCCAATGTTCTGACCAGATATGTGCCCATTGCTGAACCTTCATTAGACTCTTTCC
TTGTACCCCTCCCCTACCCATTTCTTGAAAATAGACATTGTTTAGATCTAAAAAGTCCCACCTCAGTTTCCCCAAATGAC
CGGGAAGTACCCCAAACCTTATTCGAACTAACCAACCAGCTCGCTTCTCGCTTCTGTAACCGCGCTTTTTGCTCCCCAGC
CCTAGCCCTATAAAAAGGGTAAAAACTCCACACTCGGCGCGCCAGTCCTCCGATAGACTGAGTCGCCCGGGTACCCGTGT
TCCCAATAAAGCCTCTTGCTGTTTACATCCGAATCGTGGACTCGCTGATCCTTGGGAGGGTCTCCTCAGATTGATTGACT
GCCCACCTGGGGGGGGTCTTTCATTTGGAGGTTCCACCGAGATCAGGAGACCCCTGCCCAGGGACCACCGACCCCCGCCG
GGAGGTAAGCTGGCCAGCGGTCGTTTCGTGTCTGTCTCTGTCTCCGTGCGTGTTTGTGCCGGCATCTAATGTTTGCGCCT

GCGTCTGTACTAGTTGGCTAACTAGATCTGTATCTGGCGGTTCCGCGGAAGAACTGACGAGTTCGTATTCCCGGCCGCAG
CCCCTGGGAGACGTCCCAGCGGCCTCGGGGGCCCGTTTTGTGGCCCATTCTGTATCAGTTAACCTACCCGAGTCGGACTT
TTTGGAGCTCCGCCACTGTACGTGGCTTTGTTGGGGGACGAGAGACAGAGACACTTCCCGCCCCCGTCTGGATTTTTGCT
TTCGGTTTTACGCCGAAACCGCGCTGCGCGTCTGATTTGTTTTATTGCTCTTTTGTTCTTCGTTAGTTTTTTCTGTCTTT
AAGTGTTTTCAAGATCATGGGACAGACCGTAACTACCCCTCTGAGTCTAACCTTGCAGCACTGGGGAGATGTCCAGCGCA
TTGCATCCAACCAGTCTGTGGATGTCAGGAAGAGGCGCTGGATTACCTTCTGTTCCGCCGAATGGCCAACTTTCAATGTA
GGATGGCCTCAGGATGGTACTTTCAATTTAAGTATTATCTCTCAGGTTAAGTCTAGAGTGTTTTGTCCTGGTCCCCACGG
ACACCCGGATCAGGTCCCATATATCGTTACCTGGGAGGCACTTGCCTATGACCCCCCTCCGTGGGTCAAACCGTTTGTTT
CTCCTAAACCTCCTCCCTTGCCGACAGCTCCCGTCCTCCCGCCCGGTCCTTCTGCGCAACCTCCGTCCCGATCTGCCCTT
TACCCTGCCCTTACCCCCTCTATAAAGTCCAAACCTCCTAAGCCCCAGGTTCTCCCTGATAGCGGCGGACCTCTCATTGA
CCTTCTCACAGAGGACCCCCCGCCGTACGGAGCACAACCTTCCTCCTCTGCCAGAGAGAACGATGAAGAAGAGGCGGCCA
CCACCTCCGAGGTTTCCCCCCCTTCTCCCATGGCGTCTCGACTGCGGGGAAGGAGAGACCCTCCCGCAGCGGACTCCACC
TCCTCCCAGGCATTCCCACTCCGTATGGGGGGAGATGGCCAGCTTCAGTACTGGCCGTTTTCCTCCTCTGACTTATATAA
TTGGAAAAATAATAACCCTTCCTTTTCTGAAGATCCAGGTAAATTGACGGCCTTGATTGAGTCCGTCCTCATCACCCACC
AGCCCACCTGGGACGACTGTCAGCAGTTGTTGGGGACCCTGCTGACCGGAGAAGAAAAGCAGCGGGTGCTCCTAGAGGCT
AGAAAGGCAGTCCGGGGCAATGATGGACGCCCCACTCAGTTGCCTAATGAAATCAATGCTGCCTTTCCCCTTGAGCGCCC
TGATTGGGATTACACCACTACAGAAGGTAGGAACCACCTAGTCCTCTATCGCCAGTTGCTCTTAGCGGGTCTCCAAAACG
CGGGCAGAAGCCCCACCAATTTGGCCAAGGTAAAAGGGATAACCCAGGGACCTAATGAGTCTCCCTCAGCCTTTTTAGAG
AGACTCAAGGAGGCCTATCGCAGGTACACTCCTTATGACCCTGAGGACCCAGGGCAAGAAACCAATGTGTCTATGTCATT
CATCTGGCAGTCTGCCCCGGATATCGGGCGAAAGTTAGAGCGGTTAGAAGATTTAAAGAGCAAGACCTTAGGAGACTTAG
TGAGGGAAGCTGAAAAGATCTTTAATAGACGAGAAACCCCGGAAGAAAGAGAGGAACGTATCAGGAGAGAAACAGAGGAA
AAAGAAGAACGCCGTAGGGCAGAGGATGAGCAGAAAGAGAAAGAAAGGGACCGCAGAAGACATAGAGAGATGAGCAAGCT
CTTGGCCACTGTAGTTATTGGTCAGAGACAGGATAGACAGGGGGGAGAGCGGAAGAGGCCCCAACTTGATAAGGATCAAT
GCGCCTACTGCAAAGAAAAGGGACACTGGGCTAAAGACTGCCCAAAGAAGCCACGAGGGCCCCGAGGACCGAGGCCCCAG
ACCTCCCTCCTGACCTTAGGTGACTAGGGAGGTCAGGGTCAGGAGCCCCCCCCTGAACCCAGGATAACCCTCAAAGTCGG
GGGGCAACCCGTCACCTTCCTGGTGGATACTGGGGCCCAACACTCCGTGCTGACCCAAAATCCTGGACCCCTAAGTGACA
AGTCTGCCTGGGTCCAAGGGGCTACTGGAGGAAAGCGGTATCGCTGGACCACGGATCGCAAAGTGCATCTAGCTACCGGT
AAGGTCACCCACTCTTTCCTCCATGTACCAGACTGCCCCTATCCTCTGCTAGGAAGAGATTTGCTGACTAAACTAAAAGC
CCAAATCCACTTTGAGGGATCAGGAGCTCAGGTTGTGGGACCAATGGGACAGCCCCTGCAAGTGCTGACCCTAAACATAA
AAGATGAGTATCGGCTACATGAGACCTCAAAAGAGCCGGATGTTTCTCTAGGGTCCACATGGCTTTCTGATTTTCCCCAG
GCCTGGGCGGAAACCGGGGGCATGGGACTGGCAGTTCGCCAAGCTCCTCTGATCATACCTCTGAAGGCAACCTCTACCCC
CGTGTCCATAAAACAATACCCCATGTCACAAGAAGCCAGACTGGGGATCAAGCCCCACATACAGAGACTGTTGGACCAGG
GAATACTGGTACCCTGCCAGTCCCCCTGGAACACGCCCCTGCTACCCGTTAAGAAACCAGGGACTAATGATTACAGGCCT
GTCCAAGATCTGAGAGAAGTCAACAAGCGGGTGGAAGACATCCACCCCACCGTGCCCAACCCTTACAACCTCTTGAGCGG
GCTCCCACCGTCCCACCAGTGGTACACTGTGCTTGACTTAAAGGATGCCTTTTTCTGCCTGAGACTCCACCCCACCAGTC
AGCCTCTCTTCGCCTTTGAGTGGAGAGACCCAGAGATGGGAATCTCAGGACAATTAACCTGGACCAGACTCCCACAGGGT
TTCAAAAACAGTCCCACCCTGTTTGATGAGGCACTGCACAGAGACCTAGCAGGCTTCCGGATCCAGCACCCAGACTTGAT
CCTGCTACAGTACGTGGATGACTTACTGCTGGCCGCCACTTCTGAGCTCGACTGCCAACAAGGTACTCGGGCCCTGTTAC
AAACCCTAGGGGACCTCGGGTATCGGGCCTCGGCCAAGAAAGCCCAAATTTGCCAGAAACAGGTCAAGTATCTGGGGTAT
CTTCTAAAAGAGGGTCAGAGATGGCTGACTGAGGCCAGAAAAGAGACTGTGATGGGGCAGCCTATTCCGAAGACCCCTCG
ACAACTAAGGGAGTTCCTAGGGACGGCAGGCTTCTGTCGCCTCTGGATCCCTGGGTTTGCAGAAATGGCAGCCCCCTTGT
ATCCTCTCACCAAAACGGGGACTCTGTTTAATTGGGGCCCAGACCAGCAAAAGGCCTATCAAGAAATCAAACAGGCTCTT
CTAACTGCCCCAGCCCTGGGGTTGCCAGATTTGACTAAGCCCTTTGAACTCTTTGTCGACGAGAAGCAGGGCTACGCCAA
AGGCGTCCTAACGCAAAAACTGGGACCTTGGCGTCGGCCGGTGGCCTACCTGTCCAAAAAGCTAGACCCAGTGGCAGCTG
GGTGGCCCCCTTGCCTACGGATGGTAGCAGCCATTGCCGTTCTGACAAAGGATGCAGGCAAGCTAACTATGGGACAGCCG
CTAGTCATTCTGGCCCCCCATGCGGTAGAAGCACTGGTCAAACAACCCCCTGACCGCTGGCTATCCAATGCTCGCATGAC
CCACTATCAGGCAATGCTCCTAGATACGGACCGAGTTCAGTTCGGACCGGTGGTAGCCTTAAACCCGGCCACGTTGCTCC
CCTTGCCGGGAAAAGAGACCCCCCACGACTGCCTCGAGATCTTGGCTGAGACACACGGCACCAGACCGGACCTCACGGAC
CAGCCCCTCCCAAATGCCGACCACACCTGGTATACAGATGGAAGCAGCTTCCTGCAGGAGGGGCAACGTAAGGCTGGAGC
AGCGGTGACCACCGAGACCGAGGTAATCTGGGCCAAGGCGTTGCCAGCCGGGACATCCGCCCAGCGAGCTGAACTAATAG
CACTCACCCAGGCCCTAAAGATGGCAGAAGGTAAAAAGCTAAATGTTTATACTGATAGCCGCTATGCCTTTGCTACCGCC
CATGTCCATGGAGAAATATATAGGAGACGTGGGTTGCTCACCTCAGAAGGCAAGGAGATCAAGAACAAGGGCGAAATCTT
GGCCTTACTGAAAGCTCTCTTTCTGCCCAAAAGACTCAGTATAATTCACTGCCCAGGACATCAGAAAGGCAATAGTGCTG
AAGCTAAAGGCAACCGAATGGCGGACCAGGCAGCCCGGGAAGCAGCCATGGGGACTGACACAAAGGCCTCCTCACTTCTC
ATAGAGACCTCAACCCCGTACACTCCAGACTTCTTCCATTATACTGAAACAGATATAAAGAACCTACGAGAGTTGGGAGC
CACATATGATAGAGAGAAAAAATATTGGGTCCTGCAAGGTAAACCTGTGATGCCTGACCAGTTCACCTTTGAATTATTAG
ACTTCCTTCACCAGCTCACCCACCTTAGCTATCAGAAGATGAGGGCACTTCTAGACAGAAAAGAAAGCCCCTATTACATG

CTAAATAAAGATAAGATCCTCCACGAGGTGGCGGAATCATGCCAAGCCTGTGTCCAAGTAAATGCCAGTAAAGCTAAGGT
CGGTCCCGGGGTGCGAGTAAGAGGACATCGACCAGGCACCCATTGGGAAATTGACTTTACTGAAGTAAGGCCCGGACTGT
ATGGGCATAAGTATCTTCTGGTGTTTGTGGACACGTTCTCTGGCTGGGTGGAAGCCTTCCCAACCAAGCATGAGACTGCC
AAAGTTGTGACCAAAAAGCTTCTAGAAGAAATATTTCCAAGGTTTGGAATGCCCCAAGTATTGGGGACTGATAATGGGCC
TGCCTTCGTCTCCCAGGTAAGTCAGTCGGTGGCCAAGCTACTGGGGATTGATTGGAAACTACATTGTGCTTACAGACCCC
AGAGTTCAGGTCAGGTAGAAAGAATGAATAGGACAATCAAGGAGACTTTGACCAAATTAACGCTTGCAACTGGCACTAGA
GACTGGGTACTCCTACTTCCCTTGGCCCTCTACCGAGCCCGCAACACTCCGGGCCCCCATGGACTCACTCCGTATGAAAT
CCTGTATGGGGCGCCCCCGCCCCTTGTTAATTTCCATGATCCTGAAATGTCAAAGTTTACTAATAGCCCCTCTCTCCAAG
CTCACTTACAGGCCCTCCAAGCGGTACAACGAGAGGTCTGGAAGCCTCTGGCCGCTGCCTATCAGGACCAACAAGACCAG
CCTGTGATACCACACCCCTTCCGTGTCGGCGACACCGTGTGGGTACGCCGGCACCAGACTAAGAACTTGGAACCTCGCTG
GAAAGGACCCTACACCGTCCTGCTGACCACCCCCACCGCTCTCAAAGTAGACGGCATCGCTGCGTGGATCCACGCCGCTC
ACGTAAAAGCGGCGACAACCCCTCCGGCCGGAACAGCATCAGGACCGACATGGAAGGTCCAGCGTTCTCAAAACCCTTTA
AAGATAAGATTAACCCGTGGGCCCCCCTGATAGTCCTGGGGATCTTAATAAGGGCAGGAGTATCAGTACCACATGACAGC
CCTCATCAGGTCTTCAATGTTACTTGGAGAGTTACCAACTTAATGACAGGACAAACAGCTAATGCTACCTCCCTCCTGGG
GACAATGACCGATGCCTTTCCCAAACTGTACTTTGACTTGTGCGACTTAATAGGGGACGACTGGGATGAGACTGGACTCG
GGTGTCGCACTCCCGGGGGAAGAAAAAGGGCAAGAACATTTGACTTCTATGTTTGCCCCGGGCATACTGTACCAACAGGG
TGTGGAGGCCCGAGAGAGGGCTACTGTGGCAAATGGGGCTGTGAGACCACTGGACAGGCATACTGGAAGCCATCATCATC
ATGGGACCTAATTTCCCTTAAGCGAGGAAACACCCCTCGGAATCAGGGCCCCTGTTATGATTCCTCAGCGGTCTCCAGTG
GCATCCAGGGTGCCACACCGGGGGGTCGATGCAATCCCCTAGTCCTAGAATTCACTGACGCGGGTAAAAAGGCCAGCTGG
GATGGCCCCAAAGTATGGGGACTAAGACTGTACCGATCCACAGGAACCGACCCGGTGACCCGGTTCTCTTTGACCCGCCA
GGTCCTCAATATAGGGCCCCGCATCCCCATTGGGCCTAATCCCGTGATCACTGACCAGTTACCCCCCTCCCGACCCGTGC
AGATCATGCTCCCCAGGCCTCCTCAGCCTTCCCCTACAGGCGCAGCCTCTATACAACCTGGGACGGGAGACAGACTGCTG
AACCTGGTAGATGGAGCCTACCAAGCACTCAACCTCACCAGTCCTGACAAAACCCAAGAGTGCTGGTTGTGTCTGGTATC
GGGACCCCCCTATTACGAAGGGGTTGCCGTCCTAGGTACCTACTCCAACCATACCTCTGCCCCAGCTAACTGCTCCGTGG
CCTCCCAACACAAGCTGACCCTGTCCGAAGTAACCGGACAGGGACTCTGCGTAGGAGCAGTTCCCAAAACCCATCAGGCC
CTGTGTAATACCACCCAGAATACAAGCGACGGGTCCTACTATCTGGCTGCTCCCGCCGGGACCATTTGGGCTTGCAACAC
CGGGCTCACTCCCTGCCTATCTACTACTGTACTCAACCTCACCACCGATTACTGTGTCCTGGTTGAGCTCTGGCCAAAGG
TGACCTACCACTCCCCTGGTTATGTTTATGGCCAGTTTGAGAGAAAAACCAAATATAAAAGAGAGCCGGTGTCATTAACT
CTGGCCCTGCTGTTGGGAGGACTTACTATGGGCGGCATAGCTGCAGGAGTAGGAACAGGGACTACAGCCCTAGTGGCCAC
CAAGCAATTCGAGCAGCTCCAGGCAGCCATACATACAGACCTTGGGGCCTTAGAAAAATCAGTCAGTGCCCTAGAAAAGT
CTCTGACCTCGTTGTCTGAGGTGGTCCTACAGAACCGGAGAGGATTAGATCTACTGTTCCTAAAAGAAGGAGGATTATGT
GCTGCCCTAAAAGAAGAATGCTGTTTCTACGCGGACCACACTGGCGTAGTGAGAGATAGCATGGCAAAGCTAAGAGAAAG
GTTAAACCAGAGACAAAAATTGTTCGAATCAGGACAAGGGTGGTTTGAGGGACTGTTTAACAGGTCCCCATGGTTCACGA
CCTTGATATCCACCATTATGGGCCCCTTGATAATACTCTTATTAATCCTACTCTTCGGACCCTGTATTCTCAACCGCTTG
GTCCAGTTTGTAAAAGACAGAATTTCGGTGGTGCAGACCCTGGTTCTGACCCAACAGTATCATCAACTCAAATCAATAGG
TCCAGAAGAAGTGGAATCACGTGAATAAAAGATTTTATTCAGTTTCCAGAAAGAGGGGGGAATGAAAGACCCCACCATAA
GGCTTAGCAAGCTAGCTGTAGTAACGCCATTTTGCAAGGCATGAAAAAGTACCCGAGCTGGGTTCTCAAAAGTTACAAGG
AAGTTCAGTTATAGATTAACAGTTAAAGATCAAGGCTGAATAGCACTGGGACAGGGGCCGAACAGGATATCGGTGATCAA
GCACCTGGGCCCCGGCTCAGGGCCAAGAACAGATGGTTCTCAGATAAAGCGGAACCAGCAACAGACACAGAAGCCCCGAT
AGACGTCAGTGTTAGCAGAACTAGCTTCACTGATTTAGAAAAATAGAGGTGCACAGTGCTCTGGTCACTCCTTGAACCTG
TGTGTCTGCCAATGTTCTGACCAGATATGTGCCCATTGCTGAACCTTCATTAGACTCTTTCCTTGTACCCCTCCCCTACC
CATTTCTTGAAAATAGACATTGTTTAGATCTAAAAAGTCCCACCTCAGTTTCCCCAAATGACCGGGAAGTACCCCAAACC
TTATTCGAACTAACCAACCAGCTCGCTTCTCGCTTCTGTAACCGCGCTTTTTGCTCCCCAGCCCTAGCCCTATAAAAAGG
GTAAAAACTCCACACTCGGCGCGCCAGTCCTCCGATAGACTGAGTCGCCCGGGTACCCGTGTTCCCAATAAAGCCTCTTG
CTGTTTACATCCGAATCGTGGACTCGCTGATCCTTGGGAGGGTCTCCTCAGATTGATTGACTGCCCACCTGGGGGGGGTC
TTTCA
>Mpmv3
TGAAAGACCCCACCATAAGGCTTAGCAAGCTAGCTGCAGTAACGCCATTTTGCAAGGCATGAAAAAGTACCAGAGCTGGG
TTCTCAAAAGTTACAAGGAAGTTCAGTTATAGATTAACAGTTAAAGATCAAGGCTGAATAGCACTGGGACAGGGGCCGAA
CAGGATATCGGTGGTCAAGCACCTGGGCCCCGGCTCAGGGCCAAGAACAGATGGTTCTCAGATAAAGCGGAACCAGCAAC
AGACACAGAAGCCCCGATAGACGTCAGTGTTAGCAGAACTAGCTTCACTGATTTAGAAAAATAGAGGTGCACAGTGCTCT
GGTCACTCCTTAAACCTGTGTGTCTGCCAATGTTCTGACCAGATATGTGCCCATTGCTGAACCTTCATTAGACTCTTTCC
TTGTACCCCTCCCCTACCCATTTCTAGAAAATAGACATTGTTTAGATCTAAAAAGTCCCACCTCAGTTTCCCCAAATGAC
CGGGAAGTACCCCAAACCTTATTCGAACTAACCAACCAGCTCGCTTCTCGCTTCTGTAACCGCGCTTTTTGCTCCCCAGC
CCTAGCCCTATAAAAAGAGTAAAAACTCCACACTCGGCGCGCCAGTCCTCCGATAGACTGAGTCGCCCGGGTACCCGTGT
TCCCAATAAAGCCTCTTGCTGTTTACATCCGAATCGTGGACTCGCTGATCCTTGGGAGGGTCTCCTCAGATTGATTGACT
GCCCACCTGGGGGGTCTTTCATTTGGAGGTCCCACCGAGATCAGGAGACCCCTGCCCAGGGACCACCGACCCCCGCCGGG

AGGTAAGCTGGCCAGCGGTCGTTTCGTGTCTGTCTCTGTCTCCGTGCGTGTTTGTGCCGGCATCTAATGTTTGCGCCTGC
GTCTGTACTAGTTGGCTAACTAGATCTGTATCTGGCGGTTCCGCGGAAGAACTGACGAGTTCGTATTCCCGGCCGCAGCC
CCTGGGAGACGTCCCAGCGGCCTCGGGGGCCCGTTTTGTGGCCCATTCTGTATCAGTTAACTTACCCGAGTCGGACTTTT
TGGAGCTCCGCCACTGTACGTGGCTTTGTTGGGGGACGAGAGACAGAGACACTTCCCGCCCCCGTCTGGATTTTTGCTTT
CGGTTTTACGCCGAAACCGCGCTGCGCGTCTGATTTGTTTTATTGCTCTTTTGTTCTTCGTTAGTTTTTTCTGTCTTTAA
GTGTTTTCAAGATCATGGGACAGACCGTAACTACCCCTCTGAGTCTAACCTTGCAGCACTGGGGAGATGTCCAGCGCATT
GCATCCAACCAGTCTGTGGATGTCAGGAAGAGGCGCTGGATTACCTTCTGTTCCGCCGAATGGCCAACTTTCAATGTAGG
ATGGCCTCAGGATGGTACTTTCAATTTAAGTATTATCTCTCAGGTTAAGTCTAGAGTGTTTTGTCCTGGTCCCCACGGAC
ACCCGGATCAGGTCCCATATATCGTTACCTGGGAGGCACTTGCCTATGACCCCCCTCCGTGGGTCAAACCGTTTGTTTCT
CCTAAACCTCCTCCCTTGCCGACAGCTCCCGTCCTCCCGCCCGGTCCTTCTGCGCAACCTCCGTCCCGATCTGCCCTTTA
CCCTGCCCTTACCCCCTCTATAAAGTCCAAACCTCCTAAGCCCCAGGTTCTCCCTGATAGCGGCGGACCTCTCATTGATC
TTCTCACAGAGGACCCCCCGCCGTACGGAGCACAACCTTCCTCCTCTGCCAGAGAGAACGATGAAAAAGAGGCGGCCAC
CACCTCCGAGGTTTCCCCCCCTTCTCCCATGGCGTCTCGACTGCGGGGAAGGAGAGACCCTCCCGCAGCGGACTCCACCT
CCTCCCAGGCATTCCCACTCCGTATGGGGGGAGATGGCCAGCTTCAGTACTGGCCGTTTTCCTCCTCTGACTTATATAAT
TGGAAAAATAATAACCCTCCCTTTTCTGAAGATCCAGGTAAATTGACGGCCTTGATTGAGTCCGTCCTCATCACCCACCA
GCCCACCTGGGACGACTGTCAGCAGTTGTTGGGGACCCTGCTGACCGGAGAAGAAAAGCAGCGGGTGCTCCTAGAGGCTA
GAAAGGCAGTCCGGGGCAATGATGGACGCCCCACTCAGTTGCCTAATGAAATCAATGCTGCCTTTCCCCTTGAGCGCCCT
GATTGGGATTACACCACTACAGAAGGTAGGAACCACCTAGTCCTCTATCGCCAGTTGCTCTTAGCGGGTCTCCAAAACGC
GGGCAGAAGCCCCACCAATTTGGCCAAGGTAAAAGGGATAACCCAGGGACCTAATGAGTCTCCCTCAGCCTTTTTAGAGA
GACTCAAGGAGGCCTATCGCAGGTACACTCCTTATGACCCTGAGGACCCAGGGCAAGAAACCAATGTGTCTATGTCATTC
ATCTGGCAGTCTGCCCCGGATATCGGGCGAAAGTTAGAGCGGTTAGAAGATTTAAAGAGCAAGACCTTAGGAGACTTAGT
GAGGGAAGCTAAAAAGATCTTTAATAAACGAGAAACCCCGGAAGAAAGAGAGGAACGTATCAGGAGAGAAACAGAGGAAA
AAGAAGAACGCCGTAGGGCAGAGGATGAGCAGAAAGAGAAAGAAAGGGACCGCAGAAGACATAGAGAGATGAGCAAGCTC
TTGGCCACTGTAGTTATTGGTCAGAGACAGGATAGACAGGGGGGAGAGCGGAAGAGGCCCCAACTTGATAAGGATCAATG
CGCCTACTGCAAAGAAAAGGGACACTGGGCTAAAGACTGCCCAAAGAAGCCACGAGGGCCCCGAGGACCGAGGCCCCAGA
CCTCCCTCCTGACCTTAGGTGACTAGGGAGGTCAGGGTCAGGAGCCCCCCCCTGAACCCAGGATAACCCTCAAAGTCGGG
GGGCAACCCGTCACCTTCCTGGTAGATACTGGGGCCCAACACTCCGTGCTGACCCAAAATCCTGGACCCCTAAGTGACAA
GTCTGCCTGGGTCCAAGGGGCTACTGGAGGAAAGCGGTATCGCTGGACCACGGATCGCAAAGTGCATCTAGCTACCGGTA
AGGTCACCCACTCTTTCCTCCATGTACCAGACTGCCCCTATCCTCTGCTAGGAAGAGATTTGCTGACTAAACTAAAAGCC
CAAATCCACTTTGAGGGATCAGGAGCTCAGGTTGTGGGACCAATGGGACAGCCCCTGCAAGTGCTGACCCTAAACATAGA
AGATGAGTATCGGCTACATGAGACCTCAAAAGAGCCGGATGTTTCTCTAGGGTCCACATGGCTTTCTGATTTTCCCCAGG
CCTGGGCGGAAACCGGGGGCATGGGACTGGCAGTTCGCCAAGCTCCTCTGATCATACCTCTGAAGGCAACCTCTACCCCC
GTGTCCATAAAACAATACCCCATGTCACAAGAAGCCAGACTGGGGATCAAGCCCCACATACAGAGACTGTTGGACCAGGG
AATACTGGTACCCTGCCAGTCCCCCTGGAACACGCCCCTGCTACCCGTTAAGAAACCAGGGACTAATGATTACAGGCCTG
TCCAAGATCTGAGAGAAGTCAACAAGCGGGTGGAAGACATCCACCCCACCGTGCCCAACCCTTACAACCTCTTGAGCGGG
CTCCCACCGTCCCACCAGTGGTACACTGTGCTTGACTTAAAGGATGCCTTTTTCTGCCTGAGACTCCACCCCACCAGTCA
GCCTCTCTTCGCCTTTGAGTGGAGAGACCCAGAGATGGGAATCTCAGGACAATTAACCTGGACCAGACTCCCACAGGGTT
TCAAAAACAGTCCCACCCTGTTTGATGAGGCACTGCACAGAGACCTAGCAGGCTTCCGGATCCAGCACCCAGACTTGATC
CTGCTACAGTACGTGGATGACTTACTGCTGGCCGCCACTTCTGAGCTCGACTGCCAACAAGGTACTCGGGCCCTGTTACA
AACCCTAGGGGACCTCGGGTATCGGGCCTCGGCCAAGAAAGCCCAAATTTGCCAGAAACAGGTCAAGTATCTGGGGTATC
TTCTAAAAGAGGGTCAGAGATGGCTGACTGAGGCCAGAAAAGAGACTGTGATGGGGCAGCCTATTCCGAAGACCCCTCGA
CAACTAAGGGAGTTCCTAGGGACGGCAGGCTTCTGTCGCCTCTGGATCCCTGGGTTTGCAGAAATGGCAGCCCCCTTGTA
CCCTCTCACCAAAACGGGGACTCTGTTTAATTGGGGCCCAGACCAGCAAAAGGCCTATCAAGAAATCAAACAGGCTCTTC
TAACTGCCCCAGCCCTGGGGTTGCCAGATTTGACTAAGCCCTTTGAACTCTTTGTCGACGAGAAGCAGGGCTACGCCAAA
GGCGTCCTAACGCAAAAACTGGGACCTTGGCGTCGGCCGGTGGCCTACCTGTCCAAAAAGCTAGACCCAGTGGCAGCTGG
GTGGCCCCCTTGCCTACGGATGGTAGCAGTCATTGCCGTTCTGACAAAGGATGCAGGCAAGCTAACTATGGGACAGCCGC
TAGTCATTCTGGCCCCCCATGCGGTAGAAGCACTGGTCAAACAACCCCCTGACCGCTGGCTATCCAATGCTCGCATGACC
CACTATCAGGCAATGCTCCTAGATACGGACCGAGTTCAGTTCGGACCGGTGGTAGCCTTAAACCCGGCCACGTTGCTCCC
CTTGCCGGGAAAAGAGACCCCCCACGACTGCCTCGAGATCTTGGCTGAGACACACGGCACCAGACCGGACCTCACGGACC
AGCCCCTCCCAAATGCCGACCACACCTGGTATACAGATGGAAGCAGCTTCCTGCAGGAGGGGCAACGTAAGGCTGGAGCA
GCGGTGACCACCGAGACCGAGGTAATCTGGGCCAAGGCGTTGCCAGCCGGGACATCCGCCCAGCGAGCTGAACTAATAGC
ACTCACCCAGGCCCTAAAGATGGCAGAAGGTAAGAAGCTAAATGTTTATACTGATAGCCGCTATGCCTTTGCTACCGCCC
ATGTCCATGGAGAAATATATAGGAGACGTGGGTTGCTCACCTCAGAAGGCAAGGAGATCAAGAACAAGGGCGAAATCTTG
GCCTTACTGAAAGCTCTCTTTCTGCCCAAAAGACTCAGTATAATTCACTGCCCAGGACATCAGAAAGGCAATAGTGCTGA
AGCTAAAGGCAACCGAATGGCGGACCAGGCAGCCCGGGAAGCAGCCATGGGGACTGACACAAAGGCCTCCTCATTTCTCA
TAGAGACCTCAACCCCGTACACTCCAGACTTCTTCCATTATACTGAAACAGATATAAAGAACCTACGAGAGTTGGGAGCC
ACATATGATAGAGAGAAAAAATATTGGGTCCTGCAAGGTAAACCTGTGATGCCTGACCAGTTCACCTTTGAATTATTAGA

CTTCCTTCACCAGCTCACCCACCTTAGCTATCAGAAGATGAGGGCACTTCTAGACAGAAAAGAAAGCCCCTATTACATGC
TAAATAAAGATAAGATCCTCCACGAGGTGGCGGAATCATGCCAAGCCTGTGTCCAAGTAAATGCCAGTAAAGCTAAGGTC
GGTCCCGGGGTGCGAGTAAGAGGACATCGACCAGGCACCCATTGGGAAATTGACTTTACTGAAGTAAGGCCCGGACTGTA
TGGGCATAAGTATCTTCTGGTGTTTGTGGACACGTTCTCTGGCTGGGTGGAAGCCTTCCCAACCAAGCATGAGACTGCCA
AAGTTGTGACCAAAAAGCTTCTAGAAGAAATATTTCCAAGGTTTGGAATGCCCCAAGTATTGGGGACTGATAATGGGCCT
GCCTTCGTCTCCCAGGTAAGTCAGTCGGTGGCCAAGCTACTGGGGATTGATTGGAAACTACATTGTGCTTACAGACCCCA
GAGTTCAGGTCAGGTAGAAAGAATGAATAGGACAATCAAGGAGACTTTGACCAAATTAACGCTTGCAACTGGCACTAGAG
ACTGGGTACTCCTACTTCCCTTGGCCCTCTACCGAGCCCGCAACACTCCGGGCCCCCATGGACTCACTCCGTATGAAATC
CTGTATGGGGCGCCCCCGCCCCTTGTTAATTTCCATGATCCTGAAATGTCAAAGTTTACTAATAGCCCCTCTCTCCAAGC
TCACTTACAGGCCCTCCAAGCAGTACAACGAGAGGTCTGGAAGCCTCTGGCCGCTGCCTATCAGGACCAACAAGACCAGC
CTGTAATACCACACCCCTTCCGTGTCGGCGACACCGTGTGGGTACGCCGGCACCAGACTAAGAACTTGGAACCTCGCTGG
AAAGGACCCTACACCGTCCTGCTGACCACCCCCACCGCTCTCAAAGTAGACGGCATCGCTGCGTGGATCCACGCCGCTCA
CGTAAAAGCGGCGACAACCCCTCCGGCCGGAACAGCATCAGGACCGACATGGAAGGTCCAGCGTTCTCAAAACCCCTTAA
AGATAAGATTAACCCGTGGGCCCCCCTGATAGTCCTGGGGATCTTAATAAGGGCAGGAGTATCAGTACCACATGACAGCC
CTCATCAGGTCTTCAATGTTACTTGGAGAGTTACCAACTTAATGACAGGACAAACAGCTAATGCTACCTCCCTCCTGGGG
ACAATGACCGATGCCTTTCCCAAACTGTACTTTGACTTGTGCGATTTAATAGGGGACGACTGGGATGAGACTGGACTCGG
GTGTCGCACTCCCGGGGGAAGAAAAAGGGCGAGAACATTTGACTTCTATGTTTGCCCCGGGCATACTGTACCAACAGGGT
GTGGAGGCCCGAGAGAGGGCTACTGTGGCAAATGGGGCTGTGAGACCACTGGACAGGCATACTGGAAGCCATCATCATCA
TGGGACCTAATTTCCCTTAAGCGAGGAAACACCCCTCGGAATCAGGGCCCCTGTTATGATTCCTCAGCGGTCTCCAGTGG
CATCCAGGGTGCCACACCGGGGGGTCGATGCAATCCCCTAGTCCTAGAATTCACTGACGCGGGTAAAAAGGCCAGCTGGG
ATGGCCCCAAAGTATGGGGACTAAGACTGTACCGATCCACAGGAACCGACCCGGTGACCCGGTTCTCTTTGACCCGCCAG
GTCCTCAATATAGGGCCCCGCATCCCCATTGGGCCTAATCCCGTGATCACTGACCAGTTACCCCCCTCCCGACCCGTGCA
GATCATGCTCCCCAGGCCTCCTCAGCCTTCCCCTACAGGCGCAGCCTCTATACAACCTGGGACGGGAGACAGACTGCTGA
ACCTGGTAGATGGAGCCTACCAAGCACTCAACCTCACCAGTCCTGACAAAACCCAAGAGTGCTGGTTGTGTCTGGTATCG
GGACCCCCCTATTACGAAGGGGTTGCCGTCCTAGGTACCTACTCCAACCATACCTCTGCCCCAGCTAACTGCTCCGTGGC
CTCCCAACACAAGCTGACCCTGTCCGAAGTAACCGGACAGGGACTCTGCGTAGGAGCAGTTCCCAAAACCCATCAGGCCC
TGTGTAATACCACCCAGAATACAAGCGACGGGTCCTACTATCTGGCTGCTCCCGCCGGGACCATTTGGGCTTGCAACACC
GGGCTCACTCCCTGCCTATCTACTACTGTACTCAACCTCACCACCGATTACTGTGTCCTGGTTGAGCTCTGGCCAAAGGT
GACCTACCACTCCCCTGGTTATGTTTATGGCCAGTTTGAGAGAAAAATCAAATATAAAAGAGAGCCGGTGTCATTAACTC
TGGCCCTGCTGTTGGGAGGACTTACTATGGGCGGCATAGCTGCAGGAGTAGGAACAGGGACTACAGCCCTAGTGGCCACC
AAGCAATTCGAGCAGCTCCAGGCAGCCATACATACAGACCTTGGGGCCTTAGAAAAATCAGTCAGTGCCCTAGAAAAGTC
TCTGACCTCGTTGTCTGAGGTGGTCCTACAGAACCGGAGAGGATTAGATCTACTGTTCCTAAAAGAAGGAGGATTATGTG
CTGCCCTAAAAGAAGAATGCTGTTTCTACGCGGACCACACTGGCGTAGTGAGAGATAGCATGACAAAGCTAAGAGAAAGG
TTAAACCAGAGACAAAAATTGTTCGAATCAGGACAAGGGTGGTTTGAGGGACTGTTTAACAGGTCCCCATGGTTCACGAC
CTTGATATCCACCATTATGGGCCCCTTGATAATACTCTTATTAATCCTACTCTTCGGACCCTGTATTCTCAACCGCTTGG
TCCAGTTTATAAAAGACAGAATTTCGGTGGTGCAGGCCCTGGTTCTGACCCAACAGTATCACCAACTCAAATCAATAGGT
CCAGAAAAAGTGGAATCACGTGAATAAAAGATTTTATTCAGTTTCCAGAAAGAGGGGGGAATGAAAGACCCCACCATAAG
GCTTAGCAAGCTAGCTGCAGTAACGCCATTTTGCAAGGCATGAAAAAGTACCAGAGCTGGGTTCTCAAAAGTTACAAGGA
AGTTCAGTTATAGATTAACAGTTAAAGATCAAGGCTGAATAGCACTGGGACAGGGGCCGAACAGGATATCGGTGGTCAAG
CACCTGGGCCCCGGCTCAGGGCCAAGAACAGATGGTTCTCAGATAAAGCGGAACCAGCAACAGACACAGAAGCCCCGATA
GACGTCAGTGTTAGCAGAACTAGCTTCACTGATTTAGAAAAATAGAGGTGCACAGTGCTCTGGTCACTCCTTAAACCTGT
GTGTCTGCCAATGTTCTGACCAGATATGTGCCCATTGCTGAACCTTCATTAGACTCTTTCCTTGTACCCCTCCCCTACCC
ATTTCTAGAAAATAGACATTGTTTAGATCTAAAAAGTCCCACCTCAGTTTCCCCAAATGACCGGGAAGTACCCCAAACCT
TATTCGAACTAACCAACCAGCTCGCTTCTCGCTTCTGTAACCGCGCTTTTTGCTCCCCAGCCCTAGCCCTATAAAAAGAG
TAAAAACTCCACACTCGGCGCGCCAGTCCTCCGATAGACTGAGTCGCCCGGGTACCCGTGTTCCCAATAAAGCCTCTTGC
TGTTTACATCCGAATCGTGGACTCGCTGATCCTTGGGAGGGTCTCCTCAGATTGATTGACTGCCCACCTGGGGGGTCTTT
CA
>Mpmv4
TGAAAGACCCCACCATAAGGCTTAGCAAGCTAGCTGCAGTAACGCCATTTCGCAAGGCATGAAAAAGTACCAGAGCTGGG
TTCTCAAAAGTTACAAGGAAGTTCAGTTATAGATTAACAGTTAAAGATCAAGGCTGAATAGCACTGGGACAGGGGCCGAA
CAGGATATCGGTGGTCAAGCACCTGGGCCCCGGCTCAGGGCCAAGAACAGATGGTTCTCAGATAAAGCGGAACCAGCAAC
AGACACAGAAGCCCCGATAGACGTCAGTGTTAGCAGAACTAGCTTCACTGATTTAGAAAAATAGAGGTGCACAATGCTCT
GGTCACTCCTTGAACCTGTGTGTCTGCCAATGTTCTGACCAGATATGTGCCCATTGCTGAACCTTCATTAGACTCTTTCC
TTGTACCCCTCCCCTACCCATTTCTTGAAAATAGACATTGTTTAGATCTAAAAAGTCCCACCTCAGTTTCCCCAAATGAC
CGGGAAGTACCCCAAACCTTATTCGAACTAACCAACCAGCTCGCTTCTCGCTTCTGTAACCACGCTTTTTGCTCCCCAGC
CCTAGCCCTATAAAAAGGGTAAAAACTCCACACTCGGCGCGCCAGTCCTCCGATAGACTGAGTCGCCCGGGTACCCGTGT
TCCCAATAAAGCCTCTTGCTGTTTACATCCGAATCGTGGACTCGCTGATCCTTGGGAGGGTCTCCTCAGATTGATTGACT

GCCCACCTGGGGGGTCTTTCATTTGGAGGTCCCACCGAGATCAGGAGACCCCTGCCCAGGGACCACCGACCCCCGCCGGG
AGGTAAGCTGGCCAGCGGTCGTTTCGTGTCTGTCTCTGTCTCCGTGCGTGTTTGTGCCGGCATCTAATGTTTGCGCCTGC
GTCTGTACTAGTTGGCTAACTAGATCTGTATCTGGCGGTTCCGCGGAAGAACTGACGAGTTCGTATTCCCGGCCGCAGCC
CCTGGGAGACGTCCCAGCGGCCTCGGGGGCCCGTTTTGTGGCCCATTCTGTATCAGTTAACCTACCCGAGTCGGACTTTT
TGGAGCTCCGCCACTGTACGTGGCTTTGTTGGGGGACGAGAGACAGAGACACTTCCCGCCCCCGTCTGGATTTTTGCTTT
CGGTTTTACGCCGAAACCGCGCTGCGCGTCTGATTTGTTTTATTGCTCTTTTGTTCTTCGTTAGTTTTTTCTGTCTTTAA
GTGTTTTCAAGATCATGGGACAGACCGTAACTACCCCTCTGAGTCTAACCTTGCAGCACTGGGGAGATGTCCAGCGCATT
GCATCCAACCAGTCTGTGGATGTCAGGAAGAGGCGCTGGATTACCTTCTGTTCCGCCGAATGGCCAACTTTCAATGTAGG
ATGGCCTCAGGATGGTACTTTCAATTTAAGTATTATCTCTCAGGTTAAGTCTAGAGTGTTTTGTCCTGGTCCCCACGGAC
ACCCGGATCAGGTCCCATATATCGTTACCTGGGAGGCACTTGCCTATGACCCCCCTCCGTGGGTCAAACCGTTTGTTTCT
CCTAAACCTCCTCCCTTGCCGACAGCTCCCGTCCTCCCGCCCGGTCCTTCTGCGCAACCTCCGTCCCGATCTGCCCTTTA
CCCTGCCCTTACCCCCTCTATAAAGTCCAAACCTCCTAAGCCCCAGGTTCTCCCTGATAGCGGCGGACCTCTCATTGACC
TTCTCACAGAGGACCCCCCGCCGTACGGAGCACAACCTTCCTCCTCTGCCAGAGAGAACGATGAAGAAGAGGCGGCCACC
ACCTCCGAGGTTTCCCCCCCTTCTCCCATGGCGTCTCGACTGCGGGGAAGGAGAGACCCTCCCGCAGCGGACTCCACCTC
CTCCCAGGCATTCCCACTCCGTATGGGGGGAGATGGCCAGCTTCAGTACTGGCCGTTTTCCTCCTCTGACTTATATAATT
GGAAAAATAATAACCCTTCCTTTTCTGAAGATCCAGGTAAATTGACGGCCTTGATTGAGTCCGTCCTCATCACCCACCAG
CCCACCTGGGACGACTGTCAGCAGTTGTTGGGGACCCTGCTGACCGGAGAAGAAAAGCAGCGGGTGCTCCTAGAGGCTAG
AAAGGCAGTCCGGGGCAATGATGGACGCCCCACTCAGTTGCCTAATGAAATCAATGCTGCCTTTCCCCTTGAGCGCCCTG
ATTGGGATTACACCACTACAGAAGGTAGGAACCACCTAGTCCTCTATCGCCAGTTGCTCTTAGCGGGTCTCCAAAACGCG
GGCAGAAGCCCCACCAATTTGGCCAAGGTAAAAGGGATAACCCAGGGACCTAATGAGTCTCCCTCAGCCTTTTTAGAGAG
ACTCAAGGAGGCCTATCGCAGGTACACTCCTTATGACCCTGAGGACCCAGGGCAAGAAACCAATGTGTCTATGTCATTCA
TCTGGCAGTCTGCCCCGGATATCGGGCGAAAGTTAGAGCGGTTAGAAGATTTAAAGAGCAAGACCTTAGGAGACTTAGTG
AGGGAAGCTGAAAAGATCTTTAATAAACGAGAAACCCCGGAAGAAAGAGAGGAACGTATCAGGAGAGAAACAGAGGAAAA
AGAAGAACGCCGTAGGGCAGAGGATGAGCAGAAAGAGAAAGAAAGGGACCGCAGAAGACATAGAGAGATGAGCAAGCTCT
TGGCCACTGTAGTTATTGGTCAGAGACAGGATAGACAGGGGGGAGAGCGGAAGAGGCCCCAACTTGATAAGGATCAATGC
GCCTACTGCAAAGAAAAGGGACACTGGGCTAAAGACTGCCCAAAGAAGCCACGAGGGCCCCGAGGACCGAGGCCCCAGAC
CTCCCTCCTGACCTTAGGTGACTAGGGAGGTCAGGGTCAGGAGCCCCCCCCTGAACCCAGGATAACCCTCAAAGTCGGGG
GGCAACCCGTCACCTTCCTGGTAGATACTGGGGCCCAACACTCCGTGCTGACCCAAAATCTTGGACCCCTAAGTGACAAG
TCTGCCTGGGTCCAAGGGGCTACTGGAGGAAAGCGGTATCGCTGGACCACGGATCGCAAAGTGCATCTAGCTACCGGTAA
GGTCACCCACTCTTTCCTCCATGTACCAGACTGCCCCTATCCTCTGCTAGGAAGAGATTTGCTGACTAAACTAAAAGCCC
AAATCCACTTTGAGGGATCAGGAGCTCAGGTTATGGGACCAATGGGACAGCCCCTGCAAGTGCTGACCCTAAACATAGAA
GATGAGTATCGGCTACATGAGACCCCAAAAGAGCCGGATGTTTCTCTAGGGTCCACATGGCTTTCTGATTTTCCCCAGGC
CTGGGCGGAAACCGGGGGCATGGGACTGGCAGTTCGCCAAGCTCCTCTGATCATACCTCTGAAGGCAACCTCTACCCCCG
TGTCCATAAAACAATACCCCATGTCACAAGAAGCCAGACTGGGGATCAAGTCCCACATACAGAGACTGTTGGACCAGGGA
ATACTGGTACCCTGCCAGTCCCCCTGGAACACGCCCCTGCTACCCGTTAAGAAACCAGGGACTAATGATTACAGGCCTGT
CCAAGATCTGAGAGAAGTCAACAAGCGGGTGGAAGACATCCACCCCACCGTGCCCAACCCTTACAACCTCTTGAGCGGGC
TCCCACCGTCCCACCAGTGGTACACTGTGCTTGACTTAAAGGATGCCTTTTTCTGCCTGAGACTCCACCCCACCAGTCAG
CCTCTCTTCGCCTTTGAGTGGAGAGACCCAGAGATGGGAATCTCAGGACAATTAACCTGGACCAGACTCCCACAGGGTTT
CAAAAACAGTCCCACCCTGTTTGATGAGGCACTGCACAGAGACCTAGCAGGCTTCCGGATCCAGCACCCAGACTTGATCC
TGCTACAGTACGTGGATGATTTACTGCTGGCCGCCACTTCTGAGCTCGACTGCCAACAAGGTACTCGGGCCCTGTTACAA
ACCCTAGGGGACCTCGGGTATCGGGCCTCGGCCAAGAAAGCCCAAATTTGCCAGAAACAGGTCAAGTATCTGGGGTATCT
TCTAAAAGAGGGTCAGAGATGGCTGACTGAGGCCAGAAAAGAGACTGTGATGGGGCAGCCTATTCCGAAGACCCCTCGAC
AACTAAGGGAGTTCCTAGGGACGGCAGGCTTCTGTCGCCTCTGGATCCCTGGGTTTGCAGAAATGGCCGCCCCCTTGTAC
CCTCTCACCAAAACGGGGACTCTGTTTAATTGGGGCCCAGACCAGCAAAAGGCCTATCAAGAAATCAAACAGGCTCTTCT
AACTGCCCCAGCCCTGGGGTTGCCAGATTTGACTAAGCCCTTTGAACTCTTTGTCGACGAGAAGCAGGGCTACGCCAAAG
GCGTCCTAACGCAAAAACTGGGACCTTGGCGTCGGCCGGTGGCCTACCTGTCCAAAAAGCTAGACCCAGTGGCAGCTGGG
TGGCCCCCTTGCCTACGGATGGTAGCAGCCATTGCCGTTCTGACAAAGGATGCAGGCAAGCTAACTATGGGACAGCCGCT
AGTCATTCTGGCCCCCCATGCGGTAGAAGCACTGGTCAAACAACCCCCTGACCGCTGGCTATCCAATGCTCGCATGACCC
ACTATCAGGCAATGCTCCTAGATACGGACCGGGTTCAGTTCGGACCGGTGGTAGCCTTAAACCCGGCCACGTTGCTCCCC
TTGCCGGGAAAAGAGACCCCCCACGACTGCCTCGAGATCTTGGCTGAGACACACGGCACCAGACCGGACCTCACGGACCA
GCCCCTCCCAAATGCCGACCACACCTGGTATACAGATGGAAGCAGCTTCCTGCAGGAGGGGCAACGTAAGGCTGGAGCAG
CGGTGACCACCGAGACCGAGGTAATCTGGGCCAAGGCGTTGCCAGCCGGGACATCCGCCCAGCGAGCTGAACTAATAGCA
CTCACCCAGGCCCTAAAGATGGCAGAAGGTAAGAAGCTAAATGTTTATACTGATAGCCGCTATGCCTTTGCTACCGCCCA
TGTCCATGGAGAAATATATAGGAGACGTGGGTTGCTCACCTCAGAAGGCAAGGAGATCAAGAACAAGGGCGAAATCTTGG
CCTTACTGAAAGCTCTCTTTCTGCCCAAAAGACTCAGTATAATTCACTGCCCAGGACATCAGAAAGGCAATAGTGCTGAA
GCTAAAGGCAACCGAATGGCGGACCAGGCAGCCCGGGAAGCAGCCATGGGGACTGACACAAAGGCCTCCTCACTTCTCAT
AGAGACCTCAACCCCGTACACTCCAGACTTCTTCCATTATACTGAAACAGATATAAAGAACCTACGAGAGTTGGGAGCCA

CATATGATAGAGAGAAAAAATATTGGGTCCTGCAAGGTAAACCTGTGATGCCTGACCAGTTCACCTTTGAATTATTAGAC
TTCCTTCACCAGCTCACCCACCTTAGCTATCAGAAGATGAGGGCACTTCTAGACAGAAAAGAAAGCCCCTATTACATGCT
AAATAAAGATAAGATCCTCCACGAGGTGGCGGAATCATGCCAAGCCTGTGTCCAAGTAAATGCCAGTAAAGCTAAGGTCG
GTCCCGGGGTGCGAGTAAGAGGACATCGACCAGGCACCCATTGGGAAATTGACTTTACTGAAGTAAGGCCCGGACTGTAT
GGGCATAAGTATCTTCTGGTGTTTGTGGACACGTTCTCTGGCTGGGTGGAAGCCTTCCCAACCAAGCATGAGACTGCCAA
AGTTGTGACCAAAAAGCTTCTAGAAGAAATATTTCCAAGGTTTGGAATGCCCCAAGTATTGGGGACTGATAATGGGCCTG
CCTTCGTCTCCCAGGTAAGTCAGTCGGTGGCCAAGCTACTGGGGATTGATTGGAAACTACATTGTGCTTACAGACCCCAG
AGTTCAGGTCAGGTAGAAAGAATGAATAGGACAATCAAGGAGACTTTGACTAAATTAACGCTTGCAACTGGCACTAGAGA
CTGGGTACTCCTACTTCCCTTGGCCCTCTACCGAGCCCGCAACACTCCGGGCCCCCATGGACTCACTCCGTATGAAATCC
TGTATGGGGCGCCCCCGCCCCTTGTTAATTTCCATGATCCTGAAATGTCAAAGTTTACTAATAGCCCCTCTCTCCAAGCT
CACTTACAGGCCCTCCAAGCAGTACAACGAGAGGTCTGGAAGCCTCTGGCCGCTGCCTATCAGGACCAACAAGACCAGCC
TGTGATACCACACCCCTTCCGTGTCGGCGACACCGTGTGGGTACGCCGGCACCAGACTAAGAACTTGGAACCTCGCTGGA
AAGGACCCTACACCGTCCTGCTGACCACCCCCACCGCTCTCAAAGTAGACGGCATCGCTGCGTGGATCCACGCCGCTCAC
GTAAAAGCGGCGACAACCCCTCCGGCCGGAACAGCATCAGGACCGACATGGAAGGTCCAGCGTTCTCAAAACCCCTTAAA
GATAAGATTAACCCGTGGGCCCCCCTGATAGTCCTGGGGATCTTAATAAGGGCAGGAGTATCAGTACCACATGACAGCCC
TCATCAGGTCTTCAATGTTACTTGGAGAGTTACCAACTTAATGACAGGACAAACAGCTAATGCTACCTCCCTCCTGGGGA
CAATGACCGATGCCTTTCCCAAACTGTACTTTGACTTGTGCGATTTAATAGGGGACGACTGGGATGAGACTGGACTCGGG
TGTCGCACTCCCGGGGGAAGAAAAAGGGCAAGAACATTTGACTTCTATGTTTGCCCCGGGCATACTGTACCAACAGGGTG
TGGAGGCCCGAGAGAGGGCTACTGTGGCAAATGGGGCTGTGAGACCACTGGACAGGCATACTGGAAGCCATCATCATCAT
GGGACCTAATTTCCCTTAAGCGAGGAAACACCCCTCGGAATCAGGGCCCCTGTTATGATTCCTCAGCGGTCTCCAGTGGC
ATCCAGGGTGCCACACCGGGGGGTCGATGCAATCCCCTAGTCCTAGAATTCACTGACGCGGGTAAAAAGGCCAGCTGGGA
TGGCCCCAAAGTATGGGGACTAAGACTGTACCGATCCACAGGAACCGACCCGGTGACCCGGTTCTCTTTGACCCGCCAGG
TCCTCAATATAGGGCCCCGCATCCCCATTGGGCCTAATCCCGTGATCACTGACCAGTTACCCCCCTCCCGACCCGTGCAG
ATCATGCTCCCCAGGCCTCCTCAGCCTTCCCCTACAGGCGCAGCCTCTATACAACCTGGGACGGGAGACAGACTGCTGAA
CCTGGTAGATGGAGCCTACCAAGCACTCAACCTCACCAGTCCTGACAAAACCCAAGAGTGCTGGTTGTGTCTGGTATCGG
GACCCCCCTATTACGAAGGGGTTGCCGTCCTAGGTACCTACTCCAACCATACCTCTGCCCCAGCTAACTGCTCCGTGGCC
TCCCAACACAAGCTGACCCTGTCCGAAGTAACCGGACAGGGACTCTGCGTAGGAGCAGTTCCCAAAACCCATCAGGCCCT
GTGTAATACCACCCAGAATACAAGCGACGGGTCCTACTATCTGGCTGCTCCCGCCGGGACCATTTGGGCTTGCAACACCG
GGCTCACTCCCTGCCTATCTACTACTGTACTCAACCTCACCACCGATTACTGTGTCCTGGTTGAGCTCTGGCCAAAGGTG
ACCTACCACTCCCCTGGTTATGTTTATGGCCAGTTTGAGAGAAAAACCAAATATAAAAGAGAGCCGGTGTCATTAACTCT
GGCCCTGCTGTTGGGAGGACTTACTATGGGCGGCATAGCTGCAGGAGTAGGAACAGGGACTACAGCCCTAGTGGCCACCA
AGCAATTCGAGCAGCTCCAGGCAGCCATACATACAGACCTTGGGGCCTTAAAAAAATCAGTCAGTGCCCTAGAAAAGTCT
CTGACCTCGTTGTCTGAGGTGGTCCTACAGAACCGGAGAGGATTAGATCTACTGTTCCTAAAAGAAGGAGGATTATGTGC
TGCCCTAAAAGAAGAATGCTGTTTCTACGCGGACCACACTGGCGTAGTGAGAGATAGCATGGCAAAGCTAAGAGAAAGGT
TAAACCAGAGACAAAAATTGTTCGAATCAGGACAAGGGTGGTTTGAGGGACTGTTTAACAGGTCCCCATGGTTCACGACC
TTGGTATCCACCATTATGGGCCCCTTGATAATACTCTTATTAATCCTACTCTTCGGACCCTGTATTCTCAACCGCTTGGT
CCAGTTTGTAAAAGACAGAATTTCGGTGGTGCAGGCCCTGGTTCTGACCCAACAGTATCACCAACTCAAATCAATAGGTC
CAGAAGAAGTGGAATCACGTGAATAAAAGATTTTATTCAGTTTCCAGAAAGAGGGGGGAATGAAAGACCCCACCATAAGG
CTTAGCAAGCTAGCTGCAGTAACGCCATTTCGCAAGGCATGAAAAAGTACCAGAGCTGGGTTCTCAAAAGTTACAAGGAA
GTTCAGTTATAGATTAACAGTTAAAGATCAAGGCTGAATAGCACTGGGACAGGGGCCGAACAGGATATCGGTGGTCAAGC
ACCTGGGCCCCGGCTCAGGGCCAAGAACAGATGGTTCTCAGATAAAGCGGAACCAGCAACAGACACAGAAGCCCCGATAG
ACGTCAGTGTTAGCAGAACTAGCTTCACTGATTTAGAAAAATAGAGGTGCACAATGCTCTGGTCACTCCTTGAACCTGTG
TGTCTGCCAATGTTCTGACCAGATATGTGCCCATTGCTGAACCTTCATTAGACTCTTTCCTTGTACCCCTCCCCTACCCA
TTTCTTGAAAATAGACATTGTTTAGATCTAAAAAGTCCCACCTCAGTTTCCCCAAATGACCGGGAAGTACCCCAAACCTT
ATTCGAACTAACCAACCAGCTCGCTTCTCGCTTCTGTAACCACGCTTTTTGCTCCCCAGCCCTAGCCCTATAAAAAGGGT
AAAAACTCCACACTCGGCGCGCCAGTCCTCCGATAGACTGAGTCGCCCGGGTACCCGTGTTCCCAATAAAGCCTCTTGCT
GTTTACATCCGAATCGTGGACTCGCTGATCCTTGGGAGGGTCTCCTCAGATTGATTGACTGCCCACCTGGGGGGTCTTTC
A
>Mpmv5
TGAAAGACCCCACCATAAGGCTTAGCAAGCTAGCTGCAGTAACGCCATTTTGCAAGGCATGAAAAAGTACCAGAGCTGGG
TTCTCAAAAGTTACAAGGAAGTTCAGTTATAGATTAACAGTTAAAGATCAAGGCTGAATAGCACTGGGACAGGGGCCGAA
CAGGATATCGGTGGTCAAGCACCTGGGCCCCGGCTCAGGGCCAAGAACAGATGGTTCTCAGATAAAGCGGAACCAGCAAC
AGACACAGAAGCCCCGATAGACGTCAGTGTTAGCAGAACTAGCTTCACTGATTTAGAAAAATAGAGGTGCACAGTGCTCT
GGTCACTCCTTGAACCTGTGTGTCTGCCAATGTTCTGACCAGATATGTGCCCATTGCTGAACCTTCATTAGACTCTTTCC
TTGTACCCCTCCCCTACCCATTTCTTAAAAATAGACATTGTTTAGATCTAAAAAGTCCCACCTCAGTTTCCCCAAATGAC
CGGGAAGTACCCCAAACCTTATTCGAACTAACCAACCAGCTCGCTTCTCGCTTCTGTAACCGCGCTTTTTGCTCCCCAGC
CCTAGCCCTATAAAAAGGGTAAAAACTCCACACTCGGCGCGCCAGTCCTCCGATAGACTGAGTCGCCCGGGTACCCGTGT

TCCCAATAAAGCCTCTTGCTGTTTACATCCGAATCGTGGACTCGCTGATCCTTGGGAGGGTCTCCTCAGATTGATTGACT
GCCCACCTGGGGGGTCTTTCATTTGGAGGTCCCACCGAGATCAGGAGACCCCTGCCCAGGGACCACCGACCCCCGCCGGG
AGGTAAGCTGGCCAGCGGTCGTTTCGTGTCTGTCTCTGTCTCCGTGCGTGTTTGTGCCGGCATCTAATGTTTGCGCCTGC
GTCTGTACTAGTTGGCTAACTAGATCTGTATCTGGCGGTTCCGCGGAAGAACTGACGAGTTCGTATTCCCGACCGCAGCC
CCTGGGAGACGTCCCAGCGGCCTCGGGGGCCCGTTTTGTGGCCCATTCTGTATCAGTTAACCTACCCGAGTCAGACTTTT
TAGAGCTCCGCCACTGTACGTGGCTTTGTTGGGGGACGAGAGACAGAGACACTTCCCGCCCCCGTCTGGATTTTTGCTTT
CGGTTTTACGCCGAAACCGCGCTGCGCGTCTGATTTGTTTTATTGTTCTTTTGTTCTTCGTTAGTTTTTTTTCTGTCTTT
AAGTGTTTTCAAGATCATGGGACAGACCGTAACTACCCCTCTGAGTCTAACCTTGCAGCACTGGGGAGATGTCCAGCGCA
TTGCATCCAACCAGTCTGTGGATGTCAGGAAGAGGCGCTGGATTACCTTCTGTTCCGCCGAATGGCCAACTTTCAATGTA
GGATGGCCTCAGGATGGTACTTTCAATTTAAGTATTATCTCTCAGGTTAAGTCTAGAGTGTTTTGTCCTGGTCCCCACGG
ACACCCGGATCAGGTCCCATATATCGTTACCTGGGAGGCACTTGCCTATGACCCCCCTCCGTGGGTCAAACCGTTTGTTT
CTCCTAAACCTCCTCCCTTGCCGACAGCTCCCGTCCTCCCGCCCGGTCCTTCTGCGCAACCTCCGTCCCGATCTGCCCTT
TACCCTGCCCTTACCCCCTCTATAAAGTCCAAACCTCCTAAGCCCCAGGTTCTCCCTGATAGCGGCGGACCTCTCATTGA
CCTTCTCACAGAGGACCCCCCGACGGAGCACAACCTTCCTCCTCTGCCAGAGAGAACAATGAAAAAGAGGCGGCCACCAC
CTCCGAGGTTTCCCCCCCTTCTCCCATGGCGTCTCGACTGCGGGGAAGGAGAGACCCTCCCGCAGCGGACTCCACCTCCT
CCCAGGCATTCCCACTCCGTATGGGGGGAGATGGCCAGCTTCAGTACTGGCCGTTTTCCTCCTCTGACTTATATAATTGG
AAAAATAATAACCCTTCCTTTTCTGAAGATCCAGGTAAATTGACGGCCTTGATTGAGTCCGTCCTCATCACCCACCAGCC
CACCTGGGACGACTGTCAGCAGTTGTTGGGGACCCTGCTGACCGGAGAAAAAAAGCAGCGGGTGCTCCTAGAGGCTAGAA
AGGCAGTCCGGGGCAATGATGGACGCCCCACTCAGTTGCCTAATGAAATCAATGCTGCCTTTCCCCTTGAGCGCCCTGAT
TGGGATTACACCACTACAGAAGGTAGGAACCACCTAGTCCTCTATCGCCAGTTGCTCTTAGCGGGTCTCCAAAACGCGGG
CAGAAGTCCCACCAATTTGGCCAAGGTAAAAGGGATAACCCAGGGACCTAATGAGTCTCCCTCAGCCTTTTTAGAGAGAC
TCAAGGAGGCCTATCGCAGGTACACTCCTTATGACCCTGAGGACCCAGGGCAAGAAACCAATGTGTCTATGTCATTCATC
TGGCAGTCTGCCCCGGATATCGGGCGAAAGTTAGAGCGGTTAAAAGATTTAAAGAGCAAGACCTTAGGAGACTTAGTGAG
GGAAGCTAAAAAGATCTTTAATAAACGAGAAACCCCGGAAGAAAGAGAGGAACGTATCAGGAGAGAAACAGAGGAAAAAG
AAGAACGCCGTAGGGCAGAAGATGAGCAGAAAGAGAAAGAAAGGGACCGCAGAAGACATAGAGAGATGAGCAAGCTCTTG
GCCACTGTAGTTATTGGTCAGAGACAGGATAGACAGGGGGGAGAGCGGAAGAGGCCCCAACTTGATAAGGATCAATGCGC
CTACTGCAAAGAAAAGGGACACTGGGCTAAAGACTGCCCAAAGAAGCCACGAGGGCCCCGAGGACCGAGGCCCCAGACCT
CCCTCCTGACCTTAGGTGACTAGAGAGGTCAGGGTCAGGAGCCCCCCCTGAACCCAGGATAACCCTCAAAGTCGGGGGG
CAACCCGTCACCTTCCTGGTAGATACTGGGGCCCAACACTCCGTGCTGACCCAAAATCCTGGACCCCTAAGTGACAAGTC
TGCCTGGGTCCAAGGGGCTACTGGAGGAAAGCGGTATCGCTGGACCACGGATCGCAAAGTACATCTAGCTACCGGTAAGG
TCACCCACTCTTTCCTCCATGTACCAGACTGCCCCTATCCTCTGCTAAGAAGAGATTTGCTGACTAAACTAAAAGCCCAA
ATCCACTTTAAGGGATCAGGAGCTCAGGTTGTGGGACCAATGGGACAGCCCCTGCAAGTGCTGACCCTAAACATAGAAGA
TGAGTATCGGCTACATGAGACCTCAAAAGAGCCGGATGTTTCTCTAGGGTCCACATGGCTTTCTGATTTTCCCCAGGCCT
GGGCAAAAACCGGGGGCATGGGACTGGCAGTTCGCCAAGCTCCTCTGATTATACCTCTGAAGGCAACCTCTACCCCCGTG
TCCATAAAACAATACCCCATGTCACAAGAAGCCAGACTGGGGATCAAGCCCCACATACAGAGACTGTTGGACCAGGGAAT
ACTGGTACCCTGCCAGTCCCCCTGGAACACGCCCCTGCTACCCGTTAAGAAACCAGGGACTAATGATTACAGGCCTGTCC
AAGATCTGAGAAAAGTCAACAAGCGGGTGGAAGACATCCACCCCACCGTGCCCAACCCTTACAACCTCTTGAGCGGGCTC
CCACCGTCCCACCAGTGGTACACTGTGCTTGACTTAAAGGATGCCTTTTTCTGCCTGAGACTCCACCCCACCAGTCAGCC
TCTCTTCGCCTTTGAGTAGAGAGACCCAGAGATGGGAATCTCAGGACAATTAACCTGGACCAGACTCCCACAGGGTTTCA
AAAACAGTCCCACCCTGTTTAATGAGGCACTGCACAGAGACCTAGCAGGCTTCCGGATCCAGCACCCAGACTTGATCCTG
CTACAGTACGTGGATGACTTACTGCTGGCCGCCACTTCTGAGCTCGACTGCCAACAAGGTACTCGGGCCCTGTTACAAAC
CCTAGGGGACCTCGGGTATCGGGCCTCGGCCAAGAAAGCCCAAATTTGCCAGAAACAGGTCAAGTATCTGGGGTATCTTC
TAAAAGAGGGTCAGAGATGGCTGACTGAGGCCAGAAAAGAGACTGTGATGGGGCAGCCTACTCCGAAGACCCCTCGACAA
CTAAGGGAGTTCCTAGGGACGGCAGGCTTCTGTCGCCTCTGGATCCCTGGGTTTGCAGAAATGGCAGCCCCCTTGTACCC
TCTCACCAAAACGGGGACTCTGTTTAATTGGGGCCCAGACCAGCAAAAGGCCTATCAAAAAATCAAACAGGCTCTTCTAA
CTGCCCCAGCCCTGAGATTGCCAGATTTGACTAAGCCCTTTGAACTCTTTGTCGACGAGAAGCAGGGCTACGCCAAAGGC
GTCCTAACGCAAAAACTGGGACCTTGGCGTCGGCCGGTGGCCTACCTGTCCAAAAAGCTAGACCCAGTGGCAGCTGGGTG
GCCCCCTTGCCTACGGATGGTAGCAGCCATTGCCGTTCTGACAAAGGATGCAGGCAAACTAACTATGGGACAGCCGCTAG
TCATTCTGGCCCCCCATGCGGTAGAAGCACTGGTCAAACAACCCCCTGACCGCTGGCTATCCAATGCTCGCATGACCCAC
TATCAGGCAATGCTCCTAGATACGGACCGAGTTCAGTTCGGACCGGTGGTAGCCTTAAACCCGGCCACGTTGCTCCCCTT
GCCGGGAAAAGAGACCCCCCACGACTGCCTCGAGATCTTGGCTGAGACACACGGCACCAGACCGGACCTCACGGACCAGC
CCCTCCCAAATGCCGACCACACCTGGTATACAGATAAAAGCAGCTTCCTGCAGGAGGGGCAACGTAAGGCTGGAGCAGCG
GTGACCACCGAGACCGAGGTAATCTGGGCCAAGGCGTTGCCAGCCGGGACATCCGCCCAGCGAGCTGAACTAATAGCACT
CACCCAGGCCCTAAAGATGGCAGAAGGTAAGAAGCTAAATGTTTATACTGATAGCCGCTATGCCTTTGCTACCGCCCATG
TCCATAAAAAAATATATAGGAGACGTGGGTTGCTCACCTCAAAAGGCAAGAAGATCAAGAACAAGGGCAAAATCTTGGCC
TTACTGAAAGCTCTCTTTCTGCCCAAAAGACTCAGTATAATTCACTGCCCAGGACATCAGAAAGACAATAGTGCTGAAGC
TAAAGGCAACCGAATGGCGGACCAGGCAGCCCGGGAAGCAGCCATGGGGACTGACACAAAGGCCTCCTCACTTCTCATAG

AGACCTCAACCCCGTACACTCCAGACTTCTTCCATTATACTGAAACAGATATAAAGAACCTACAAGAGTTGGGAGCCACA
TATGATAGAGAGAAAAAATATTGGGTCCTGCAAGGTAAACCTGTGATGCCTGACCAGTTCACCTTTAAATTATTAGACTT
CCTTCACCAGCTCACCCACCTTAGCTATCAGAAGATAAGGGCACTTCTAGACAGAAAAGAAAGCCCCTATTACATGCTAA
ATAAAGATAAGATCCTCCACGAGGTGGCGGAATCATGCCAAGCCTGTGTCCAAGTAAATGCCAGTAAAGCTAAGGTCGGT
CCCGGGGTGCGAGTAAGAGGACATCGACCAGGCACCCATTGAGAAATTGACTTTACTGAAGTAAGGCCCGGACTGTATGG
GTATAAGTATCTTCTGGTGTTTGTGGACACGTTCTCTGGCTGGGTAGAAGCCTTCCCAACCAAGCATGAGACTGCCAAAG
TTGTGACCAAAAAGCTTCTAGAAAAAATATTTCCAAGGTTTGGAATGCCCCAAGTATTGGGGACTGATAATGGGCCTGCC
TTCGTCTCCCAGGTAAGTCAGTCGGTGGCCAAGCTACTGGGGATTAATTAAAAACTACATTGTGCTTACAGACCCCAGAG
TTCAGGTCAGGTAAAAAGAATGAATAAGACAATCAAGGAGACTTTGACCAAATTAACGCTTGCAACTGGCACTAGAGACT
GGGTACTCCTACTTCCCTTGGCCCTCTACCGAGCCCGCAACACTCCGGGCCCCCATGGACTCACTCCGTATAAAATCCTG
TATGGGGCGCCCCCGCCCCTTGTTAATTTCCATGATCCTAAAATGTCAAAGTTTACTAATAGCCCCTCTCTCCAAGCTCA
CTTACAGGCCCTCCAAGCAGTACAACGAGAGGTCTGGAAGCCTCTGGCCGCTGCCTATCAGGACCAACAAGACCAGCCTG
TGATACCACACCCCTTCCGTGTCGGCGACACCGTGTGGGTACGCCGGCACCAGACTAAGAACTTGAAACCTCGCTGGAAA
GGACCCTACACCGTCCTGCTGACCACTCCCACCGCTCTCAAAGTAGACGGCATCGCTGCGTGGATCCACGCCGCTCACGT
AAAAGCGGCGACAACCCCTCCGGCCGGAACAGCATCAGGACCGACATGGAAGGTCCAGCGTTCTCAAAACCCCTTAAAGA
TAAGATTAACCCGTGGGCCCCCCTGATAGTCCTGGGGATCTTAATAAGGGCAGGAGTATCAGTACCACATGACAGCCCTC
ATCAGGTCTTCAATGTTACTTAGAGAGTTACCAACTTAATGACAGGACAAACAGCTAATGCTACCTCCCTCCTGGGGACA
ATGACCGATGCCTTTCCCAAACTGTACTTTGACTTGTGCAATTTAATAGGGGACGACTGGGATGAGACTGGACTCGGGTG
TCGCACTCCCGGGGAAAGAAAAAGGGCAAAAACATTTGACTTCTATGTTTGCCCCGGGCATACTGTACCAACAGGGTGTG
GAGGCCCGAGAGAGGGCTACTGTGGCAAATGGGGCTGTGAGACCACTGGACAGGCATACTGGAAGCCATCATCATCATGG
GACCTAATTTCCCTTAAGCGAGGAAACACCCCTCGGAATCAGGGCCCCTGTTATGATTCCTCAGCGGTCTCCAGTGACAT
CCAGGGTGCCACACCGGGGGGTCGATGCAATCCCCTAGTCCTAGAATTCACTGACGCGGGTAAAAAGGCCAGCTGGGATG
GCCCCAAAGTATGGGGACTAAGACTGTACCGATCCACAAGAACCGACCCGGTGACCCGGTTCTCTTTGACCCGCCAGGTC
CTCAATATAGGGCCCCGCATCCCCATTGGGCCTAATCCCGTGATCACTGACCAGTTACCCCCCTCCCGACCCGTGCAGAT
CATGCTCCCCAGGCCTCCTCAGCCTTCCCCTACAGGCGCAGCCTCTATACAACCTGGGACGGGAGACAGACTGCTGAACC
TGGTAGATAGAGCCTACCAAGCACTCAACCTCACCAGTCCTGACAAAACCCAAGAGTGCTGGTTGTGTCTGGTATCGGGA
CCCCCCTATTACGAAGGGGTTGCCGTCCTAGGTACCTACTCCAACCATACCTCTGCCCCAGCTAACTGCTCCGTGGCCTC
CCAACACAAGCTGACCCTGTCCGAAGTAACCGGACAGGGACTCTGCGTAGGAGCAGTTCCCAAAACCCATCAGGCCCTGT
GTAATACCACCCAAAATACAAGCGACGGGTCCTACTATCTGGCTGCTCCCGCCGGGACCATTTGGGCTTGCAACACCGGG
CTCACTCCCTGCCTATCTACTACTGTACTCAACCTCACCACCGATTACTGTGTCCTGGTTGAGCTCTGGCCAAAAGTGAC
CTACCACTCCCCTGGTTATGTTTATGGCCAGTTTGAGAGAAAAACCAAATATAAAAGAGAGCCGGTGTCATTAACTCTGG
CCCTGCTGTTGGGAGGACTTACTATGGGCGGCATAGCTACAGGAGTAAAAACAGGGACTACAGCCCTAGTGGCCACCAAG
CAATTCGAGCAGCTCCAGGCAGCCATACATACAGACCTTGGGGCCTTAGAAAAATCAGTCAGTGCCCTAAAAAAGTCTCT
GACCTCGTTGTCTGAGGTGGTCCTACAGAACCGGAGAGGATTAGATCTACTGTTCCTAAAAAAAGGAAGATTATGTGCTG
CCCTAAAAGAAGAATGCTGTTTCTACGCGGACCACACTGGCGTAGTGAGAGATAGCATGGCAAAGCTAAGAAAAAGGTTA
AACCAGAGACAAAAATTGTTCGAATCAGGACAAGGGTGGTTTGAGGGACTGTTTAACAGGTCCCCATAGTTCACGACCTT
GATATCCACCATTATGGGCCCCTTGATAATACTCTTATTAATCCTACTCTTCGGACCCTGTATTCTCAACCGCTTGGTCC
AGTTTGTAAAAGACAGAATTTCGGTGGTGCAGGCCCTGGTTCTGACCCAACAGTATCACCAACTCAAATCAATAGGTCCA
GAAGAAGTGAAATCACGTGAATAAAAGATTTTATTCAGTTTCCAGAAAGAGGGGGGAATGAAAGACCCCACCATAAGGCT
TAGCAAGCTAGCTGCAGTAACGCCATTTTGCAAGGCATGAAAAAGTACCAGAGCTGGGTTCTCAAAAGTTACAAGAAAGT
TCAGTTATAGATTAACAGTTAAAGATCAAGGCTGAATAGCACTGGGACAGGGGCCGAACAGAATATCGGTGGTCAAGCAC
CTGGGCCCCGGCTCAGGGCCAAGAACAGATGGTTCTCAGATAAAGCGGAACCAGCAACAGACACAGAAGCCCCGATAGAC
GTCAGTGTTAGCAGAACTAGCTTCACTGATTTAAAAAAATAGAGGTGCACAGTGCTCTGGTCACTCCTTGAACCTGTGTG
TCTGCCAATGTTCTGACCAGATATGTGCCCATTGCTGAACCTTCATTAGACTCTTTCCTTGTACCCCTCCCCTACCCATT
TCTTAAAAATAGACATTGTTTAGATCTAAAAAGTCCCACCTCAGTTTCCCCAAATGACCGGGAAGTACCCCAAACCTTAT
TCGAACTAACCAACCAGCTCGCTTCTCGCTTCTGTAACCGCGCTTTTTGCTCCCCAGCCCTAGCCCTATAAAAAGGGTAA
AAACTCCACACTCGGCGCGCCAGTCCTCCGATAGACTGAGTCGCCCGGGTACCCGTGTTCCCAATAAAGCCTCTTGCTGT
TTACATCCGAATCGTGGACTCGCTGATCCTTGAGAGGGTCTCCTCAGATTGATTGACTGCCCACCTGGGGGGTCTTTCA
>Mpmv6
TGAAAGACCCCACCATAAGGCTTAGCAAGCTAGCTGCAGTAACGCCATTTTGCAAGGCATAAAAAAAGTACCAGAGCTGG
GTTCTCAAAAGTTACAAGGAAGTTCAGTTATAGATTAACAGTTAAAGATCAAGACTGAATAGCACTGGGACAGGGGCCGA
ACAGGATATCGGTGGTCAAGCACCTGGGCCCCGGCTCAGGGCCAAGAATAGATGGTTCTCAGATAAAGCGGAACCAGCAA
CAGACACAGAAGCCCCGATAGACGTCAGTGTTAGCAGAACTAGCTTCACTGATTTAGAAAAATAGAGGTGCACAGTGCTC
TGGTCACTCCTTGAACCTGTGTGTCTGCCAATGTTCTGACCTGATATGTGCCCATTGCTGAACCTTCATTAGACTCTTTC
CTTGTACCCCTCCCCTACCCATTTCTTGAAAATAGACATTGTTTAGATCTAAAAAGTCCCACCTCAGTTTCCCCAAATGA
CCGGGAAGTACCCCAAACCTTATTCGAACTAACCAACCAGCTCGCTTCTCGCTTCTGTAACCACGCTTTTTGCTCCCCAG
CCCTAGCCCTATAAAAAGGGTAAAAACTCCACACTCGGCGCGCCAGTCCTCCGATAGACTGAGTCGCCCGGGTACCCGTG

TTCCCAATAAAGCCTCTTGCTGTTTACATCCGAATCGTGGACTCGCTGATCCTTGGGAGGGTCTCCTCAGATTGATTGAC
TGCCCACCTGGGGGGGGGGGGTCTTTCATTTGGAGGTTCCACCGAGATCAGGAGACCCCTGCCCAGGGACCACCGACCCC
CGCCGGGAGGTAAGCTGGCCAGCGGTCGTTTCGTGTCTGTCTCTGTCTCCGTGCGTGTTTGTGCCGGCATCTAATGTTTG
CGCCTGCGTCTGTACTAGTTGGCTAACTAGATCTGTATCTGGCGGTTCCGCGGAAGAACTGACGAGTTCGTATTCCCGGC
CGCAGCCCCTGGGAGACGTCCCAGCGGCCTCGGGGGCCCGTTTTGTGGCCCATTCTGTATCAGTTAACCTACCCGAGTCG
GACTTTTTGGAGCTCCGCCACTGTACGTGGCTTTGTTGGGGGACGAGAGACAGAGACACTTCCCGCCCCCGTCTGGATTT
TTGCTTTCGGTTTTACGCCGAAACCGCGCTGCGCGTCTGATTTGTTTTATTGTTCTTTTGTTCTTCGTTAGTTTTTTTCT
GTCTTTAAGTGTTTTCAAGATCATGGGACAGACCGTAACTACCCCTCTGAGTCTAACCTTGCAGCACTGGGGAGATGTCC
AGCGCATTGCATCCAACCAGTCTGTGGATGTCAGGAAGAGGCGCTGGATTACCTTCTGTTCCGCCGAATGGCCAACTTTC
AATGTAGGATGGCCTCAGGATGGTACTTTCAATTTAAGTATTATCTCTCAGGTTAAGTCTAGAGTGTTTTGTCCTGGTCC
CCACGGACACCCGGATCAGGTCCCATATATCGTTACCTGAGAGGCACTTGCCTATGACCCCCCTCCGTGGGTCAAACCGT
TTGTTTCTCCTAAACCTCCTCCCTTGCCGACAGCTCCCGTCCTCCCGCCCGGTCCTTCTGCGCAACCTCCGTCCCGATCT
GCCCTTTACCCTGCCCTTACCCCCTCTATAAAGTCCAAACCTCCTAAGCCCCAGGTTCTCCCTGATAGCGGCGGACCTCT
CATTGACCTTCTCACAGAGGACCCCCCGCCGTACGGAGCACAACCTTCCTCCTCTGCCAGAGAGAACGATGAAGAAGAGG
CAGCCACCACCTCCGAGGTTTCCCCCCCTTCTCCCATGGCGTCTCGACTGCGGGAAAGGAGAGACCCTCCCGCAGCGGAC
TCCACCTCCTCCCAGGCATTCCCACTCCGTATGGGGGGAGATGGCCAGCTTCAGTACTGGCCGTTTTCCTCCTCTGACTT
ATATAATTGGAAAAATAATAACCCTTCCTTTTCTGAAGATCCAGGTAAATTGACGGCCTTGATTGAGTCCGTCCTCATCA
CCCACCAGCCCACCTGGGACGACTGTCAGCAGTTGTTGGGGACCCTGCTGACCGGAGAAGAAAAGCAGCGGGTGCTCCTA
GAGGCTAGAAAGGCAGTCCGGGGCAATGATGGACGCCCCACTCAGTTGCCTAATGAAATCAATGCTGCCTTTCCCCTTGA
GCGCCCTGATTGGGATTACACCACTACAGAAGGTAGGAACCACCTAGTCCTCTATCGCCAGTTGCTCTTAGCGGGTCTCC
AAAACGCGGGCAGAAGCCCCACCAATTTGGCCAAGGTAAAAGGGATAACCCAGGGACCTAATGAGTCTCCCTCAGCCTTT
TTAGAGAGACTCAAGGAGGCCTATCGCAGGTACACTCCTTATGACCCTGAGGACCCAGGGCAAGAAACCAATGTGTCTAT
GTCATTCATCTGGCAGTCTGCCCCGGATATCGGGCGAAAGTTAGAGCGGTTAGAAGATTTAAAGAGCAAGACCTTAGGAG
ACTTAGTGAGGGAAGCTGAAAAGATCTTTAATAAACGAGAAACCCCGGAAGAAAGAGAGGAACGTATCAGGAGAGAAACA
GAGGAAAAAGAAGAACGCCGTAGGGCAGAGGATGAGCAGAAAGAGAAAGAAAGGGACCGCAGAAGACATAGAGAGATGAG
CAAGCTCTTGGCCACTGTAGTTATTGGTCAGAGACAGGATAGACAGGGGGGAGAGCGGAAGAGGCCCCAACTTGATAAGG
ATCAATGCGCCTACTGCAAAGAAAAGGGACACTGGGCTAAAGACTGCCCAAAGAAGCCACGAGGGCCCCGAGGACCGAGG
CCCCAGACCTCCCTCCTGACCTTAGGTGACTAGGGAGGTCAGGGTCAGGAGCCCCCCCCTGAACCCAGGATAACCCTCAA
AGTCGGGGGGCAACCCGTCACCTTCCTGGTAGATACTGGGGCCCAACATTCCGTGCTGACCCAAAATCCTGGACCCCTAA
GTGACAAGTCTGCCTGGGTCCAAGGGGCTACTGGAAGAAAGCGGTATCGCTGGACCACGGATCGCAAAGTACATCTAGCT
ACCGGTAAGGTCACCCACTCTTTCCTCCATGTACCAGACTGCCCCTATCCTCTGCTAGGAAGAGATTTGCTGACTAAACT
AAAAGCCCAAATCCACTTTGAGGGATCAGGAGCTCAGGTTGTGGGACCAATGGGACAGCCCCTGCAAGTGCTGACCCTAA
ACATAGAAGATGAGTATCGGCTACATGAGACCTCAAAAGAGCCGGATGTTTCTCTAGGGTCCACATGGCTTTCTGATTTT
CCCCAGGCCTGGGCGGAAACCGGGGGCATGGGACTGGCAGTTCGCCAAGCTCCTCTGATCATACCTCTGAAGGCAACCTC
TACCCCCGTGTCCATAAAACAATACCCCATGTCACAAGAAGCCAGACTAGGGATCAAGCCCCACATACAGAGACTGTTGG
ACCAGGGAATACTGGTACCCTGCCAGTCCCCCTGGAACACGCCCCTGCTACCCGTTAAGAAACCAGGGACTAATGATTAC
AGGCCTGTCCAAGATCTGAGAGAAGTCAACAAGCGGGTGGAAGACATCCACCCCACCGTGCCCAACCCTTACAACCTCTT
GAGCGGGCTCCCACCGTCCCACCAGTGGTACACTGTGCTTGACTTAAAGGATGCCTTTTTCTGCCTGAGACTCCACCCCA
CCAGTCAGCCTCTCTTCGCCTTTGAGTGGAGAGACCCAGAGATGGGAATCTCAGGACAATTAACCTGGACCAGACTCCCA
CAGGGTTTCAAAAACAGTCCCACCCTGTTTAATGAGGCACTGCACAGAGACCTAGCAGGCTTCCGGATCCAGCACCCAGA
CTTGATCCTGCTACAGTACGTGGATGACTTACTGCTGGCCGCCACTTCTGAGCTCGACTGCCAACAAGGTACTCGGGCCC
TGTTACAAACCCTAGGGGACCTCGGGTATCGGGCCTCGGCCAAGAAAGCCCAAATTTGCCAGAAACAGGTCAAGTATCTG
GGGTATCTCCTAAAAGAGGGTCAGAGATGGCTGACTGAGGCCAGAAAAGAGACTGTGATGGGGCAGCCTACTCCGAAGAC
CCCTCGACAACTAAGGGAGTTCCTAGGGACGGCAGGCTTCTGTCGCCTCTGGATCCCTGGATTTGCAGAAATGGCAGCCC
CCTTGTACCCTCTCACCAAAACGGGGACTCTGTTTAATTGGGGCCCAGACCAGCAAAAGGCCTATCAAGAAATCAAACAG
GCTCTTCTAACTGCCCCAGCCCTGGGATTGCCAGATTTGACTAAGCCCTTTGAACTCTTTGTCGACGAGAAGCAGGGCTA
CGCCAAAGGCGTCCTAACGCAAAAACTGGGACCTTGGCGTCGGCCGGTGGCCTACCTGTCCAAAAAGCTAGACCCAGTGG
CAGCTGGGTGGCCCCCTTGCCTACGGATGGTAGCAGCCATTGCCGTTCTGACAAAGGATGCAGGCAAGCTAACTATGGGA
CAGCCGCTAGTCATTCTGGCCCCCCATGCGGTAGAAGCACTGGTCAAACAACCCCCTGACCGCTGGCTATCCAATGCTCG
CATGACTCACTATCAGGCAATGCTCCTAGATACGGACCGAGTTCAGTTCGGACCGGTGGTAGCCTTAAACCCGGCCACGT
TGCTCCCCTTGCCGGGGAAAGAGACCCCCCACGACTGCCTCGAGATCTTGGCTGAGACACACGGCACCAGACCGGACCTC
ACGGACCAGCCCCTCCCAAATGCCGACCACACCTGGTATACAGATGGAAGCAGCTTCCTGCAGGAGGGGCAACGTAAGGC
TGGAGCAGCGGTGACCACCGAGACCGAGGTAATCTGGGCCAAGGCGTTGCCAGCCGGGACATCCGCCCAGCGAGCTGAAC
TAATAGCACTCACCCAGGCCCTAAAGATGGCAGAAGGTAAGAAGCTAAATGTTTATACTGATAGCCGCTATGCCTTTGCT
ACCGCCCATGTCCATGGAGAAATATATAGGAGACGTGGGTTGCTCACCTCAGAAGGCAAGGAGATCAAGAACAAGGGCGA
AATCTTGGCCTTACTGAAAGCTCTCTTTCTGCCCAAAAGACTCAGTATAATTCACTGCCCAGGACATCAGAAAGGCAATA
GTGCTGAAGCTAAAGGCAACCGAATGGCGGACCAGGCAGCCCGGGAAGCAGCCATGGGGACTGACACAAAGGCCTCCTCA

CTTCTCATAGAGACCTCAACCCCGTACACTCCAGACTTCTTCCATTATACTGAAACAGATATAAAGAACCTACAAGAGTT
GGGAGCCACATATGATAGAGAGAAAAAATATTGGGTCCTGCAAGGTAAACCTGTGATGCCTGACCAGTTCACCTTTGAAT
TATTAGACTTCCTTCACCAGCTCACCCACCTTAGCTATCAGAAGATGAGGGCACTTCTAGACAGAAAAGAAAGCCCCTAT
TACATGCTAAATAAAGATAAGATCCTCCACGAGGTGGCGGAATCATGCCAAGCCTGTGTCCAAGTAAATGCCAGTAAAGC
TAAGGTCGGTCCCGGGGTGCGAGTAAGAGGACATCGACCAGGCACCCATTGGGAAATTGACTTTACTGAAGTAAGGCCCG
GACTGTATGGGTATAAGTATCTTCTGGTGTTTGTGGACACGTTCTCTGGCTGGGTGGAAGCCTTCCCAACCAAGCATGAG
ACTGCCAAAGTTGTGACCAAAAAGCTTCTAGAAGAAATATTTCCAAGGTTTGGAATGCCCCAAGTATTGGGGACTGATAA
TGGGCCTGCCTTCGTCTCCCAGGTAAGTCAGTCGGTGGCCAAGCTACTGGGGATTGATTGGAAACTACATTGTGCTTACA
GACCCCAGAGTTCAGGTCAGGTAGAAAGAATGAATAGGACAATCAAGGAGACTTTGACCAAATTAACGCTTGCAACTGGC
ACTAGAGACTGGGTACTCCTACTTCCCTTGGCCCTCTACCGAGCCCGCAACACTCCGGGCCCCCATGGACTCACTCCGTA
TGAAATCCTGTATGGGGCGCCACCGCCCCTTGTTAATTTCCATGATCCTGAAATGTCAAAGTTTACTAATAGCCCCTCTC
TCCAAGCTCACTTACAGGCCCTCCAAGCAGTACAACGAGAGGTCTGGAAGCCTCTGGCCGCTGCCTATCAGGACCAACAA
GACCAGCTTGTGATACCACACCCCTTCCGTGTCGGCGACACCGTGTGGGTACGCCGGCACCAGACTAAGAACTTGGAACC
TCGCTGGAAAGGACCCTACACCGTCCTGCTGACCACCCCCACCGCTCTCAAAGTAGACGGCATCGCTGCGTGGATCCACG
CCGCTCACGTAAAAGCGGCGACAACCCCTCCGGCCGGAACAGCATCAGGACCGACATGGAAGGTCCAGCGTTCTCAAAAC
CCCTTAAAGATAAGATTAACCCGTGGGCCCCCCTGATAGTCCTGGGGATCTTAATAAGGGCAGGAGTATCAGTACCACAT
GACAGCCCTCATCAGGTCTTCAATGTTACTTGGAGAGTTACCAACTTAATGACAGGACAAACAGCTAATGCTACCTCCCT
CCTGGGGACAATGACCGATGCCTTTCCCAAACTGTACTTTGACTTGTGCGATTTAATAGGGGACGACTGGGATGAGACTG
GACTCGGGTGTCGCACTCCCGGGGGAAGAAAAAGGGCAAGAACATTTGACTTCTATGTTTGCCCCGGGCATACTGTACCA
ACAGGGTGTGGAGGCCCGAGAGAGGGCTACTGTGGCAAATGGGGCTGTGAGACCACTGGACAGGCATACTGGAAGCCATC
ATCATCATGGGACCTAATTTCCCTTAAGCGAGGAAACACCCCTCGGAATCAGGGCCCCTGTTATGATTCCTCAGCGGTCT
CCAGTGGCATCCAGGGTGCCACACAGGGGGGTCGATGCAATCCCCTAGTCCTAGAATTCACTGACGCGGGTAAAAAGGCC
AGCTGGGATGGCCCCAAAGTATGGGGACTAAGACTGTACCGATCCACAGGAACCGACCCGGTGACCCGGTTCTCTTTGAC
CCGCCAGGTCCTCAATATAGGGCCCCGCATCCCCATTGGGCCTAATCCCGTGATCACTGACCAGTTACCCCCCTCCCGAC
CCGTGCAGATTATGCTCCCCAGGCCTCCTCAGCCTTCCCCTACAGGCGCAGCCTCTATACAACCTGGGACGGGAGACAGA
CTGCTGAACCTGGTAGATGGAGCCTACCAAGCACTCAACCTCACCAGTCCTGACAAAACCCAAGAGTGCTGGTTGTGTCT
GGTATCGGGACCCCCCTATTACGAAGGGGTTGCCGTCCTAGGTACCTACTCCAACCATACCTCTGCCCCAGCTAACTGCT
CCGTGGCCTCCCAACACAAGCTGACCCTGTCCGAAGTAACCGGACAGGGACTCTGCGTAGGAGCAGTTCCCAAAACCCAT
CAGGCCCTGTGTAATACCACCCAGAATACAAGCGACGGGTCCTACTATCTGGCTGCTCCCGCCGGGACCATTTGGGCTTG
CAACACCGGGCTCACTCCCTGCCTATCTACTACTGTACTCAACCTCACCACCGATTACTGTGTCCTGGTTGAGCTCTGGC
CAAAGGTGACCTACCACTCCCCTGGTTATGTTTATGGCCAGTTTGAGAGAAAAACCAAATATAAAAGAGAGCCGGTGTCA
TTAACTCTGGCCCTGCTGTTGGGAGGACTTACTATGGGCGGCATAGCTGCAGGAGTAGGAACAGGGACTACAGCCCTAGT
GGCCACCAAGCAATTCGAGCAGCTCCAGGCAGCCATACATACAGACCTTGGGGCCTTAGAAAAATCAGTCAGTGCCCTAG
AAAAGTCTCTGACCTCGTTGTCCGAGGTGGTCCTACAGAACCGGAGAGGATTAGATCTACTGTTCCTAAAAGAAGGAGGA
TTATGTGCTGCCCTAAAAGAAGAATGCTGTTTCTACGCGGACCACACTGGCGTAGTGAGAGATAGCATGGCAAAGCTAAG
AGAAAGGTTAAACCAGAGACAAAAATTGTTCGAATCAGGACAAGGGTGGTTTGAGGGACTGTTTAACAGGTCCCCATGGT
TCACGACCTTGATATCCACCATTATGGGCCCCTTGATAATACTCTTATTAATCCTACTCTTCGGACCCTGTATTCTCAAC
CGCTTGGTCCAGTTTGTAAAAGACAGAATTTCGGTGGTGCAGGCCCTGGTTCTGACCCAACAGTATCACCAACTCAAATC
AATAGGTCCAGAAGAAGTGGAATCACGTGAATAAAAGATTTTATTCAGTTTCCAGAAAGAGGGGGGAATGAAAGACCCCA
CCATAAGGCTTAGCAAGCTAGCTGCAGTAACGCCATTTTGCAAGGCATAAAAAAAGTACCAGAGCTGGGTTCTCAAAAGT
TACAAGGAAGTTCAGTTATAGATTAACAGTTAAAGATCAAGACTGAATAGCACTGGGACAGGGGCCGAACAGGATATCGG
TGGTCAAGCACCTGGGCCCCGGCTCAGGGCCAAGAATAGATGGTTCTCAGATAAAGCGGAACCAGCAACAGACACAGAAG
CCCCGATAGACGTCAGTGTTAGCAGAACTAGCTTCACTGATTTAGAAAAATAGAGGTGCACAGTGCTCTGGTCACTCCTT
GAACCTGTGTGTCTGCCAATGTTCTGACCTGATATGTGCCCATTGCTGAACCTTCATTAGACTCTTTCCTTGTACCCCTC
CCCTACCCATTTCTTGAAAATAGACATTGTTTAGATCTAAAAAGTCCCACCTCAGTTTCCCCAAATGACCGGGAAGTACC
CCAAACCTTATTCGAACTAACCAACCAGCTCGCTTCTCGCTTCTGTAACCACGCTTTTTGCTCCCCAGCCCTAGCCCTAT
AAAAAGGGTAAAAACTCCACACTCGGCGCGCCAGTCCTCCGATAGACTGAGTCGCCCGGGTACCCGTGTTCCCAATAAAG
CCTCTTGCTGTTTACATCCGAATCGTGGACTCGCTGATCCTTGGGAGGGTCTCCTCAGATTGATTGACTGCCCACCTGGG
GGGGGGGTCTTTCA
>Mpmv7
TAAAAGACCCCACCATAAGGCTTAGCAAGCTAGCTGCAGTAACGCCATTTTGCAAGGCATGAAAAAGTACCAGAGCTGGG
TTCTCAAAAGTTACAAAGAAGTTCAGTTATAGATTAACAGTTAAAGATCAAGGCTGAATAGCACTGGGACAGGGGCCGAA
CAGGATATCGGTGGTCAAGCACCTGGGCCCCGGCTCAGGGCCAAGAACAGATGGTTCTCAGATAAAGCGGAACCAGCAAC
AGACACAGAAGCCCCGATAGACGTCAGTGTTAGCAGAACTAGCTTCACTGATTTAGAAAAATAGAGGTGCACAGTGCTCT
GGTCACTCCTTGAACCTGTGTGTCTGCCAATGTTCTGACCAGATATGTGCCCATTGCTGAACCTTCATTAGACTCTTTCC
TTGTACCCCTCCCCTACCCATTTCTTGAAAATAGACATTGTTTAGATCTAAAAAGTCCCACCTCAGTTTCCCCAAATGAC
CGGGAAGTACCCCAAACCTTATTCGAACTAACCAACCAGCTCGCTTCTCGCTTCTGTAACCGCGCTTTTTGCTCCCCAGC

CCTAGCCCTATAAAAAGGGTAAAAACTCCACACTCGGCGCGCCAGTCCTCCGATAGACTGAGTCGCCCGGGTACCCGTGT
TCCCAATAAAGCCTCTTGCTGTTTACATCCGAATCGTGGACTCGCTGATCCTTGGGAGGGTCTCCTCAGATTGATTGACT
GCCCACCTGGGGGGTCTTTCATTTGGAGGTCCCACCGAGATCAGGAGACCCCTGCCCAGGGACCACCGACCCCCGCCGGG
AGGTAAGCTGGCCAGCGGTCGTTTTGTGTCTGTCTCTGTCTCCGTGCGTGTTTGTGCCGGCATCTAATGTTTGCGCCTGC
GTCTGTACTAGTTGGCTAACTAGATCTGTATCTGGCGGTTCCGCGGAAGAACTGACGAGTTCGTATTCCCGGCCGCAGCC
CCTGGGAGACGTCCCAGCGGCCTCGGGGGCCCGTTTTGTGGCCCATTCTGTATCAGTTAACCTACCCGAGTCGGACTTTT
TGGAGCTCCGCCACTGTACGTGGCTTTGTTGGGGGACGAGAGACAGAGACACTTCCCGCCCCCGTCTGGATTTTTGCTTT
CGGTTTTACGCCGAAACCGCGCTGCGCGTCTGATTTGTTTTATTGCTCTTTTGTTCTTCGTTAGTTTTTTCTGTCTTTAA
GTGTTTTCAAGATCATGGGACAGACCGTAACTACCCCTCTGAGTCTAACCTTGCAGCACTGGGGAGATGTCCAGCGCATT
GCATCCAACCAGTCTGTGGATGTCAGGAAGAGGCGCTGGATTACCTTCTGTTCCGCCGAATGGCCAACTTTCAATGTAGG
ATGGCCTCAGGATGGTACTTTCAATTTAAGTATTATCTCTCAGGTTAAGTCTAGAGTGTTTTGTCCTGGTCCCCACGGAC
ACCCGGATCAGGTCCCATATATCGTTACCTGGGAGGCACTTGCCTATGACCCCCCTCCGTGGGTCAAACCGTTTGTTTCT
CCTAAACCTCCTCCCTTGCCGACAGCTCCCGTCCTCCCGCCCGGTCCTTCTGCGCAACCTCCGTCCCGATCTGCCCTTTA
CCCTGCCCTTACCCCCTCTATAAAGTCCAAACCTCCTAAGCCCCAGGTTCTCCCTGATAGCGGCGGACCTCTCATTGACC
TTCTCACAGAGGACCCCCCGCCGTACGGAGCACAACCTTCCTCCTCTGCCAGAGAGAACGATGAAGAAGAGGCGGCCACC
ACCTCCGAGGTTTCCCCCCCTTCTCCCATGGCGTCTCGACTGCGGGGAAGGAGAGACCCTCCCGCAGCGGACTCCACCTC
CTCCCAGGCATTCCCACTCCGTATGGGGGGAGATGGCCAGCTTCAGTACTGGCCGTTTTCCTCCTCTGACTTATATAATT
GGAAAAATAATAACCCTTCCTTTTCTGAAGATCCAGGTAAATTGACGGCCTTGATTGAGTCCGTCCTCATCACCCACCAG
CCCACCTGGGACGACTGTCAGCAGTTGTTGGGGACCCTGCTGACCGGAGAAGAAAAGCAGCGGGTGCTCCTAGAGGCTAG
AAAGGCAGTCCGGGGCAATGATGGACGCCCCACTCAGTTGCCTAATGAAATCAATGCTGCCTTTCCCCTTGAGCGCCCTG
ATTGGGATTACACCACTACAGAAGGTAGGAACCACCTAGTCCTCTATCGCCAGTTGCTCTTAGCGGGTCTCCAAAACGCG
GGCAGAAGCCCCACCAATTTGGCCAAGGTAAAAGGGATAACCCAGGGACCTAATGAGTCTCCCTCAGCCTTTTTAGAGAG
ACTCAAGGAGGCCTATCGCAGGTACACTCCTTATGACCCTGAGGACCCAGGGCAAGAAACCAATGTGTCTATGTCATTCA
TCTGGCAGTCTGCCCCGGATATCGGGCGAAAGTTAGAGCGGTTAAAAGATTTAAAGAGCAAGACCTTAGGAGACTTAGTG
AGGGAAGCTGAAAAGATCTTTAATAAACGAGAAACCCCGGAAGAAAGAGAGGAACGTATCAGGAGAGAAACAGAGGAAAG
AGAAGAACGCCGTAGGGCAGAGGATGAGCAGAAAGAGAAAGAAAGGGACCGCAGAAGACATAGAGAGATGAGCAAGCTCT
TGGCCACTGTAGTTATTGGTCAGAGACAGGATAGACAGGGGGGAGAGCGGAAGAGGCCCCAACTTGATAAGGATCAATGC
GCCTACTGCAAAGAAAAGGGACACTGGGCTAAAGACTGCCCAAAGAAGCCACGAGGGCCCCGAGGACCGAGGCCCCAGAC
CTCCCTCCTGACCTTAGGTGACTAGGGAGGTCAGGGTCAGGAGCCCCCCCCTGAACCCAGGATAACCCTCAAAGTCGGGG
GGCAACCCGTCACCTTCCTGGTAGATACTGGGGCCCAACACTCCGTGCTGACCCAAAATCCTGGACCCCTAAGTGACAAG
TCTGCCTGGGTCCAAGGGGCTACTGGAGGAAAGCGGTATCGCTGGACCACGGATCGCAAAGTGCATCTAGCTACCGGTAA
GGTCACCCACTCTTTCCTCCATGTACCAGACTGCCCCTATCCTCTGCTAGGAAGAGATTTGCTGACTAAACTAAAAGCCC
AAATCCACTTTGAGGGATCAGGAGCTCAGGTTGTGGGACCAATGGGACAGCCCCTGCAAGTGCTGACCCTAAACATAGAA
GATGAGTATCGGCTACATGAGACCTCAAAAGAGCCGGATGTTTCTCTAGGGTCCACATGGCTTTCTGATTTTCCCCAGGC
CTGGGCGGAAACCGGGGGCATGGGACTGGCAGTTCGCCAAGCTCCTCTGATCATACCTCTGAAGGCAACCTCTACCCCCG
TGTCCATAAAACAATACCCCATGTCACAAGAAGCCAGACTGGGGATCAAGCCCCACATACAGAGACTGTTGGACCAGGGA
ATACTGGTACCCTGCCAGTCCCCCTGGAACACGCCCCTGCTACCCGTTAAGAAACCAGGGACTAATGATTACAGGCCTGT
CCAAGATCTGAGAGAAGTCAACAAGCGGGTGGAAGACATCCACCCCACCGTGCCCAACCCTTACAACCTCTTGAGCGGGC
TCCCACCGTCCCACCAGTGGTACACTGTGCTTGACTTAAAGGATGCCTTTTTCTGCCTAAGACTCCACCCCACCAGTCAG
CCTCTCTTCGCCTTTGAGTGGAGAGACCCAGAGATGGGAATCTCAGGACAATTAACCTGGACCAGACTCCCACAGGGTTT
CAAAAACAGTCCCACCCTGTTTGATGAGGCACTGCACAGAGACCTAGCAGGCTTCCGGATCCAGCACCCAGACTTGATCC
TGCTACAGTACGTGGATGACTTACTGCTGGCCGCCACTTCTGAGCTCGACTGCCAACAAGGTACTCGGGCCCTGTTACAA
ACCCTAGGGGACCTCGGGTATCGGGCCTCGGCCAAGAAAGCCCAAATTTGCCAGAAACAGGTCAAGTATCTGGGGTATCT
TCTAAAAGAGGGTCAGAGATGGCTGACTGAGGCCAGAAAAGAGACTGTGATGGGGCTGCCTATTCCGAAGACCCCTCGAC
AACTAAGAGAGTTCCTAGGGACGGCAGGCTTCTGTCGCCTCTGGATCCCTGGGTTTGCAGAAATGGCAGCCCCCTTGTAC
CCTCTCACCAAAACGGGGACTCTGTTTAATTGGGGCCCAGACCAGCAAAAGGCCTATCAAGAAATCAAACAGGCTCTTCT
AACTGCCCCAGCCCTGGGGTTGCCAGATTTGACTAAGCCCTTTGAACTCTTTGTCGACGAGAAGCAGGGCTACGCCAAAG
GCGTCCTAACGCAAAAACTGGGACCTTGGCGTCGGCCGGTGGCCTACCTGTCCAAAAAGCTAGACCCAGTGGCAGCTGGG
TGGCCCCCTTGCCTACGGATGGTAGCAGCCATTGCCGTTCTGACAAAGGATGCAGGCAAGCTAACTATGGGACAGCCGCT
AGTCATTCTGGCCCCCCATGCGGTAGAAGCACTGGTCAAACAACCCCCTGACCGCTGGCTATCCAATGCTCGCATGACCC
ACTATCAGGCAATGCTCCTAGATACGGACCGAGTTCAGTTCGGACCGGTGGTAGCCTTAAACCCGGCCACGTTGCTCCCC
TTGCCGGGAAAAGAGACCCCCCACGACTGCCTCGAGATCTTGGCTGAGACACACGGCACCAGACCGGACCTCACGGACCA
GCCCCTCCCAAATGCCGACCACACCTGGTATACAGATGGAAGCAGCTTCCTGCAGGAGGGGCAACGTAAGGCTGGAGCAG
CGGTGACCACCGAGACCGAGGTAATCTGGGCCAAGGCGTTGCCAGCCGGGACATCCGCCCAGCGAGCTGAACTAATAGCA
CTCACCCAGGCCCTAAAGATGGCAGAAGGTAAGAAGCTAAATGTTTATACTGATAGCCGCTATGCCTTTGCTACCGCCCA
TGTCCATGGAGAAATATATAGGAGACGTGGGTTGCTCACCTCAGAAGGCAAGGAGATCAAGAACAAGGGCGAAATCTTGG
CCTTACTGAAAGCTCTCTTTCTGCCCAAAAGACTCAGTATAATTCACTGCCCAGGACATCAGAAAGGCAATAGTGCTGAA

GCTAAAGGCAACCGAATGGCGGACCAGGCAGCCCGGGAAGCAGCCATGGGGACTGACACAAAGGCCTCCTCACTTCTCAT
AGAGACCTCAACCCCGTACACTCCAGACTTCTTCCATTATACTGAAACAGATATAAAGAACCTACGAGAGTTGGGAGCCA
CATATGATAGAGAGAAAAAATATTGGGTCCTGCAAGGTAAACCTGTGATGCCTGACCAGTTCACCTTTAAATTATTAGAC
TTCCTTCACCAGCTCACCCACCTTAGCTATCAGAAGATGAGGGCACTTCTAGACAGAAAAGAAAGCCCCTATTACATGCT
AAATAAAGATAAGATCCTCCACGAGGTGGCGGAATCATGCCAAGCCTGTGTCCAAGTAAATGCCAGTAAAGCTAAGGTCG
GTCCCGGGGTGCGAGTAAGAGGACATCGACCAGGCACCCATTGGGAAATTGACTTTACTGAAGTAAGGCCCGGACTGTAT
GGGCATAAGTATCTTCTGGTGTTTGTGGACACGTTCTCTGGCTGGGTGGAAGCCTTCCCAACCAAGCATGAGACTGCCAA
AGTTGTGACCAAAAAGCTTCTAGAAGAAATATTTCCAAGGTTTGGAATGCCCCAAGTATTGGGGACTGATAATGGGCCTG
CCTTCGTCTCCCAGGTAAGTCAGTCGGTGGCCAAGCTACTGGGGATTGATTGGAAACTACATTGTGCTTACAGACCCCAG
AGTTCAGGTCAGGTAGAAAGAATAAATAGGACAATCAAGGAGACTTTGACCAAATTAACGCTTGCAACTGGCACTAGAGA
CTGGGTACTCCTACTTCCCTTGGCCCTCTACCGAGCCCGCAACACTCCGGGCCCCCATGGACTCACTCCGTATGAAATCC
TGTATGGGGCGCCCCCGCCCCTTGTTAATTTCCATGATCCTGAAATGTCAAAGTTTACTAATAGCCCCTCTCTCCAAGCT
CACTTACAGGCCCTCCAAGCAGTACAACGAGAGGTCTGGAAGCCTCTGGCCGCTGCCTATCAGGACCAACAAGACCAGCC
TGTGATACCACACCCCTTCCGTGTCGGCGACACCGTGTGGGTACGCCGGCACCAGACTAAGAACTTGGAACCTCGCTGGA
AAGGACCCTACACCGTCCTGCTGACCACCCCCACCGCTCTCAAAGTAGACGGCATCGCTGCGTGGATCCACGCCGCTCAC
GTAAAAGCGGCGACAACCCCTCCGGCCGGAACAGCATCAGGACCGACATGGAAGGTCCAGCGTTCTCAAAACCCCTTAAA
GATAAGATTAACCCGTGGGCCCCCCTGATAGTCCTGGGGATCTTAATAAGGGCAGGAGTATCAGTACCACATGACAGCCC
TCATCAGGTCTTCAATGTTACTTGGAGAGTTACCAACTTAATGACAGGACAAACAGCTAATGCTACCTCCCTCCTGGGGA
CAATGACCGATGCCTTTCCCAAACTGTACTTTGACTTGTGCGATTTAATAGGGGACGACTGGGATGAGACTGGACTCGGG
TGTCGCACTCCCGGGGGAAGAAAAAGGGCAAGAACATTTGACTTCTATGTTTGCCCCGGGCATACTGTACCAACAGGGTG
TGGAGGCCCGAGAGAGGGCTACTGTGGCAAATGGGGCTGTGAGACCACTGGACAGGCATACTGGAAGCCATCATCATCAT
GGGACCTAATTTCCCTTAAGCGAGGAAACACCCCTCGGAATCAGGGCCCCTGTTATGATTCCTCAGCGGTCTCCAGTGGC
ATCCAGGGTGCCACACCGGGGGGTCGATGCAATCCCCTAGTCCTAGAATTCACTGACGCGGGTAAAAAGGCCAGCTGGGA
TGGCCCCAAAGTATGGGGACTAAGACTGTACCGATCCACAGGAACCGACCCGGTGACCCGGTTCTCTTTGACCCGCCAGG
TCCTCAATATAGGGCCCCGCATCCCCATTGGGCCTAATCCCGTGATCACTGACCAGTTACCCCCCTCCCGACCCGTGCAG
ATCATGCTCCCCAGGCCTCCTCAGCCTTCCCCTACAGGCGCAGCCTCTATACAACCTGGGACGGGAGACAGACTGCTGAA
CCTGGTAGATGGAGCCTACCAAGCACTCAACCTCACCAGTCCTGACAAAACCCAAGAGTGCTGGTTGTGTCTGGTATCGG
GACCCCCCTATTACGAAGGGGTTGCCGTCCTAGGTACCTACTCCAACCATACCTCTGCCCCAGCTAACTGCTCCGTGGCC
TCCCAACACAAGCTGACCCTGTCCGAAGTAACCGGACAGGGACTCTGCGTAGGAGCAGTTCCCAAAACCCATCAGGCCCT
GTGTAATACCACCCAGAATACAAGCGACGGGTCCTACTATCTGGCTGCTCCCGCCGGGACCATTTGGGCTTGCAACACCG
GGCTCACTCCCTGCCTATCTACTACTGTACTCAACCTCACCACCGATTACTGTGTCCTGGTTGAGCTCTGGCCAAAGGTG
ACCTACCACTCCCCTGGTTATGTTTATGGCCAGTTTGAAAGAAAAACCAAATATAAAAGAGAGCCGGTGTCATTAACTCT
GGCCCTGCTGTTGGGAGGACTTACTATGGGCGGCATAGCTGCAGGAGTAGGAACAGGGACTACAGCCCTAGTGGCCACCA
AGCAATTCGAGCAGCTCCAGGCAGCCATACATACAGACCTTGGGGCCTTAGAAAAATCAGTCAGTGCCCTAGAAAAGTCT
CTGACCTCGTTGTCTGAGGTGGTCCTACAGAACCGGAGAGGATTAGATCTACTGTTCCTAAAAGAAGGAGGATTATGTGC
TGCCCTAAAAGAAGAATGCTGTTTCTACGCGGACCACACTGGCGTAGTGAGAGATAGCATGGCAAAGCTAAGAGAAAGGT
TAAACCAGAGACAAAAATTGTTCGAATCAGGACAAGGGTGGTTTGAGGGACTGTTTAACAGGTCCCCATGGTTCACGACC
TTGATATCCACCATTATGGGCCCCTTGATAATACTCTTATTAATCCTACTCTTCGGACCCTGTATTCTCAACCGCTTGGT
CCAGTTTGTAAAAGACAGAATTTCGGTGGTGCAGGCCCTGGTTCTGACCCAACAGTATCACCAACTCAAATCAATAGGTC
CAGAAAAAGTGGAATCACGTGAATAAAAGATTTTATTCAGTTTCCAGAAAGAGGGGGGAATAAAAGACCCCACCATAAGG
CTTAGCAAGCTAGCTGCAGTAACGCCATTTTGCAAGGCATAAAAAAGTACCAGAGCTGGGTTCTCAAAAGTTACAAAGAA
GTTCAGTTATAGATTAACAGTTAAAGATCAAGGCTGAATAGCACTGGGACAGGGGCCGAACAGGATATCGGTGGTCAAGC
ACCTGGGCCCCGGCTCAGGGCCAAGAACAGATGGTTCTCAGATAAAGCGGAACCAGCAACAGACACAGAAGCCCCGATAG
ACGTCAGTGTTAGCAGAACTAGCTTCACTGATTTAGAAAAATAGAGGTGCACAGTGCTCTGGTCACTCCTTGAACCTGTG
TGTCTGCCAATGTTCTGACCAGATATGTGCCCATTGCTGAACCTTCATTAGACTCTTTCCTTGTACCCCTCCCCTACCCA
TTTCTTGAAAATAGACATTGTTTAGATCTAAAAAGTCCCACCTCAGTTTCCCCAAATGACCGGGAAGTACCCCAAACCTT
ATTCGAACTAACCAACCAGCTCGCTTCTCGCTTCTGTAACCGCGCTTTTTGCTCCCCAGCCCTAGCCCTATAAAAAGGGT
AAAAACTCCACACTCGGCGCGCCAGTCCTCCGATAGACTGAGTCGCCCGGGTACCCGTGTTCCCAATAAAGCCTCTTGCT
GTTTACATCCGAATCGTGGACTCGCTGATCCTTGGGAGGGTCTCCTCAGATTGATTGACTGCCCACCTGGGGGGTCTTTC
A
>Mpmv8
TGAAAGACCCCACCATAAGGCTTAGCAAGCTAGCTGCAGTAACGCCATTTCGCAAGGCATGAAAAAGTACCAGAGCTGGG
TTCTCAAAAGTTACAAGGAAGTTCAGTTATAGATTAACAGTTAAAGATCAAGGCTGAATAGCACTGGGACAGGGGCCGAA
CAGGATATCGGTGGTCAAGCACCTGGGCCCCGGCTCAGGGCCAAGAACAGATGGTTCTCAGATAAAGCGGAACCAGCAAC
AGACACAGAAGCCCCGATAGACGTCAGTGTTAGCAGAACTAGCTTCACTGATTTAAAAAAATAGAGGTGCACAATGCTCT
GGTCACTCCTTAAACCTGTGTGTCTGCCAATGTTCTGACCAGATATGTGCCCATTGCTGAACCTTCATTAGACTCTTTCC
TTGTACCCCTCCCCTACCCATTTCTTGAAAATAGACATTGTTTAGATCTAAAAAGTCCCACCTCAGTTTCCCCAAATGAC

CGGGAAGTACCCCAAACCTTATTCGAACTAACCAACCAGCTCGCTTCTCGCTTCTGTAACCACGCTTTTTGCTCCCCAGC
CCTAGCCCTATAAAAAGGGTAAAAACTCCACACTCGGCGCGCCAGTCCTCCGATAGACTGAGTCGCCCGGGTACCCGTGT
TCCCAATAAAGCCTCTTGCTGTTTACATCCGAATCGTGGACTCGCTGATCCTTGGGAGGGTCTCCTCAGATTGATTGACT
GCCCACCTGGGGGGTCTTTCATTTGGAGGTTCCACCGAGATCAGGAGACCCCTGCCCAGGGACCACCGACCCCCGCCGGG
AGGTAAGCTGGCCAGCGGTCGTTTCGTGTCTGTCTCTGTCTCCGTGCGTGTTTGTGCCGGCATCTAATGTTTGCGCCTGC
GTCTGTACTAGTTGGCTAACTAGATCTGTATCTGGCGGTTCCGCGGAAGAACTGACGAGTTCGTATTCCCGGCCGCAGCC
CCTGGGAGACGTCCCAGCGGCCTCGGGGGCCCGTTTTGTGGCCCATTCTGTATCAGTTAACCTACCCGAGTCGGACTTTT
TGGAGCTCCGCCACTGTACGTGGCTTTGTTGGGGGACGAGAGACAGAGACACTTCCCGCCCCCGTCTGGATTTTTGCTTT
CGGTTTTACGCCGAAACCGCGCTGCGCGTCTGATTTGTTTTATTGCTCTTTTGTTCTTCGTTAGTTTTTTTCTGTCTTTA
AGTGTTTTCAAGATCATGGGACAGACCGTAACTACCCCTCTGAGTCTAACCTTGCAGCACTGGGGAGATGTCCAGCGCAT
TGCATCCAACCAGTCTGTGGATGTCAGGAAGAGGCGCTGGATTACCTTCTGTTCCGCCGAATGGCCAACTTTCAATGTAG
GATGGCCTCAGGATGGTACTTTCAATTTAAGTATTATCTCTCAGGTTAAGTCTAGAGTGTTTTGTCCTGGTCCCCACGGA
CACCCGGATCAGGTCCCATATATCGTTACCTGGGAGGCACTTGCCTATGACCCCCCTCCGTGGGTCAAACCGTTTGTTTC
TCCTAAACCTCCTCCCTTGCCGACAGCTCCCGTCCTCCCGCCCGGTCCTTCTGCGCAACCTCCGTCCCGATCTGCCCTTT
ACCCTGCCCTTACCCCCTCTATAAAGTCCAAACCTCCTAAGCCCCAGGTTCTCCCTGATAGCGGCGGACCTCTCATTGAC
CTTCTCACAGAGGACCCCCCGCCGTACGGAGCACAACCTTCCTCCTCTGCCAGAGAGAACGATGAAGAAGAGGCGGCCAC
CACCTCCGAGGTTTCCCCCCCTTCTCCCATGGCGTCTCGACTGCGGGGAAGGAGAGACCCTCCCGCAGCGGACTCCACCT
CCTCCCAGGCATTCCCACTCCGTATGGGGGGAGATGGCCAGCTTCAGTACTGGCCGTTTTCCTCCTCTGACTTATATAAT
TAAAAAAATAATAACCCTTCCTTTTCTGAAGATCCAGGTAAATTGACGGCCTTGATTGAGTCCGTCCTCATCACCCACCA
GCCCACCTGGGACGACTGTCAGCAGTTGTTGGGGACCCTGCTGACCGGAGAAGAAAAGCAGCGGGTGCTCCTAGAGGCTA
GAAAGGCAGTCCGGGGCAATGATGGACGCCCCACTCAGTTGCCTAATGAAATCAATGCTGCCTTTCCCCTTGAGCGCCCT
GATTGGGATTACACCACTACAGAAGGTAGGAACCACCTAGTCCTCTATCGCCAGTTGCTCTTAGCGGGTCTCCAAAACGC
GGGCAGAAGCCCCACCAATTTGGCCAAGGTAAAAGAGATAACCCAGGGACCTAATGAGTCTCCCTCAGCCTTTTTAGAGA
GACTCAAGGAGGCCTATCGCAGGTACACTCCTTATGACCCTGAGGACCCAGGGCAAGAAACCAATGTGTCTATGTCATTC
ATCTGGCAGTCTGCCCCGGATATCGGGCGAAAGTTAGAGCGGTTAGAAGATTTAAAGAGCAAGACCTTAGGAGACTTAGT
GAGGGAAGCTGAAAAGATCTTTAATAAACGAGAAACCCCGGAAGAAAGAGAGGAACGTATCAGGAGAGAAACAGAGGAAA
AAGAAGAACGCCGTAGGGCAGAGGATGAGCAGAAAGAGAAAGAAAGGGACCGCAGAAGACATAGAGAGATGAGCAAGCTC
TTGGCCACTGTAGTTATTGGTCAGAGACAGGATAGACAGGGGGGAGAGCGGAAGAGGCCCCAACTTGATAAGGATCAATG
CGCCTACTGCAAAGAAAAGGGACACTGGGCTAAAGACTGCCCAAAGAAGCCACGAGGGCCCCGAGGACCGAGGCCCCAGA
CCTCCCTCCTGACCTTAGGTGACTAGGGAGGTCAGGGTCAGGAGCCCCCCCCTGAACCCAGGATAACCCTCAAAGTCGGG
GGGCAACCCGTCACCTTCCTGGTAGATACTGGGGCCCAACACTCCGTGCTGACCCAAAATCTTGGACCCCTAAGTGACAA
GTCTGCCTGGGTCCAAGGGGCTACTGGAGGAAAGCGGTATCGCTGGACCACGGATCGCAAAGTGCATCTAGCTACCGGTA
AGGTCACCCACTCTTTCCTCCATGTACCAGACTGCCCCTATCCTCTGCTAGGAAGAGATTTGCTGACTAAACTAAAAGCC
CAAATCCACTTTGAGGGATCAGGAGCTCAGGTTATGGGACCAATGGGACAGCCCCTGCAAGTGCTGACCCTAAACATAGA
AGATGAGTATCGGCTACATGAGACCCCAAAAGAGCCGGATGTTTCTCTAGGGTCCACATGGCTTTCTGATTTTCCCCAGG
CCTGGGCGGAAACCGGGGGCATGGGACTGGCAGTTCGCCAAGCTCCTCTGATCATACCTCTGAAGGCAACCTCTACCCCC
GTGTCCATAAAACAATACCCCATGTCACAAGAAGCCAGACTGGGGATCAAGTCCCACATACAGAGACTGTTAGACCAGGG
AATACTGGTACCCTGCCAGTCCCCCTGGAACACGCCCCTGCTACCCGTTAAGAAACCAGGGACTAATGATTACAGGCCTG
TCCAAGATCTGAGAGAAGTCAACAAGCGGGTGGAAGACATCCACCCCACCGTGCCCAACCCTTACAACCTCTTGAGCGGG
CTCCCACCGTCCCACCAGTGGTACACTGTGCTTGACTTAAAGGATGCCTTTTTCTGCCTGAGACTCCACCCCACCAGTCA
GCCTCTCTTCGCCTTTGAGTGGAGAGACCCAGAGATGGGAATCTCAGGACAATTAACCTGGACCAGACTCCCACAGGGTT
TCAAAAACAGTCCCACCCTGTTTGATGAGGCACTGCACAGAGACCTAGCAGGCTTCCGGATCCAGCACCCAGACTTGATC
CTGCTACAGTACGTGGATGATTTACTGCTGGCCGCCACTTCTGAGCTCGACTGCCAACAAGGTACTCGGGCCCTGTTACA
AACCCTAGGGGACCTCGGGTATCGGGCCTCGGCCAAGAAAGCCCAAATTTGCCAGAAACAGGTCAAGTATCTGGGGTATC
TTCTAAAAGAGGGTCAGAGATGGCTGACTGAGGCCAGAAAAGAGACTGTGATGGGGCAGCCTATTCCGAAGACCCCTCGA
CAACTAAGGGAGTTCCTAGGGACGGCAGGCTTCTGTCGCCTCTGGATCCCTGGGTTTGCAGAAATGGCCGCCCCCTTGTA
CCCTCTCACCAAAACGGGGACTCTGTTTAATTGGGGCCCAGACCAGCAAAAGGCCTATCAAGAAATCAAACAGGCTCTTC
TAACTGCCCCAGCCCTGGGGTTGCCAGATTTGACTAAGCCCTTTGAACTCTTTGTCGACGAGAAGCAGGGCTACGCCAAA
GGCGTCCTAACGCAAAAACTGGGACCTTGGCGTCGGCCGGTGGCCTACCTGTCCAAAAAGCTAGACCCAGTGGCAGCTGG
GTGGCCCCCTTGCCTACGGATGGTAGCAGCCATTGCCGTTCTGACAAAGGATGCAGGCAAGCTAACTATGGGACAGCCGC
TAGTCATTCTGGCCCCCCATGCGGTAGAAGCACTGGTCAAACAACCCCCTGACCGCTGGCTATCCAATGCTCGCATGACC
CACTATCAGGCAATGCTCCTAGATACGGACCGGGTTCAGTTCGGACCGGTGGTAGCCTTAAACCCGGCCACGTTGCTCCC
CTTGCCGGGAAAAGAGACCCCCCACGACTGCCTCGAGATCTTGGCTGAGACACACGGCACCAGACCGGACCTCACGGACC
AGCCCCTCCCAAATGCCGACCACACCTGGTATACAGATGGAAGCAGCTTCCTGCAGGAGGGGCAACGTAAGGCTGGAGCA
GCGGTGACCACCGAGACCGAGGTAATCTGGGCCAAGGCGTTGCCAGCCGGGACATCCGCCCAGCGAGCTGAACTAATAGC
ACTCACCCAGGCCCTAAAGATGGCAGAAGGTAAGAAGCTAAATGTTTATACTGATAGCCGCTATGCCTTTGCTACCGCCC
ATGTCCATGGAGAAATATATAGGAGACGTGGGTTGCTCACCTCAGAAGGCAAGGAGATCAAGAACAAGGGCGAAATCTTG

GCCTTACTGAAAGCTCTCTTTCTGCCCAAAAGACTCAGTATAATTCACTGCCCAGGACATCAGAAAGGCAATAGTGCTGA
AGCTAAAGGCAACCGAATGGCGGACCAGGCAGCCCGGGAAGCAGCCATGGGGACTGACACAAAGGCCTCCTCACTTCTCA
TAGAGACCTCAACCCCGTACACTCCAGACTTCTTCCATTATACTGAAACAGATATAAAGAACCTACGAGAGTTGGGAGCC
ACATATGATAGAGAGAAAAAATATTGGGTCCTGCAAGGTAAACCTGTGATGCCTGACCAGTTCACCTTTGAATTATTAGA
CTTCCTTCACCAGCTCACCCACCTTAGCTATCAGAAGATGAGGGCACTTCTAGACAGAAAAGAAAGCCCCTATTACATGC
TAAATAAAGATAAGATCCTCCACGAGGTGGCGGAATCATGCCAAGCCTGTGTCCAAGTAAATGCCAGTAAAGCTAAGGTC
GGTCCCGGGGTGCGAGTAAGAGGACATCGACCAGGCACCCATTGGGAAATTGACTTTACTGAAGTAAGGCCCGGACTGTA
TGGGCATAAGTATCTTCTGGTGTTTGTGGACACGTTCTCTGGCTGGGTGGAAGCCTTCCCAACCAAGCATGAGACTGCCA
AAGTTGTGACCAAAAAGCTTCTAGAAGAAATATTTCCAAGGTTTGGAATGCCCCAAGTATTGGGGACTGATAATGGGCCT
GCCTTCGTCTCCCAGGTAAGTCAGTCGGTGGCCAAGCTACTGGGGATTGATTGGAAACTACATTGTGCTTACAGACCCCA
GAGTTCAGGTCAGGTAAAAAGAATGAATAGGACAATCAAGGAGACTTTGACTAAATTAACGCTTGCAACTGGCACTAGAG
ACTGGGTACTCCTACTTCCCTTGGCCCTCTACCGAGCCCGCAACACTCCGGGCCCCCATGGACTCACTCCGTATGAAATC
CTGTATGGGGCGCCCCCGCCCCTTGTTAATTTCCATGATCCTGAAATGTCAAAGTTTACTAATAGCCCCTCTCTCCAAGC
TCACTTACAGGCCCTCCAAGCAGTACAACGAGAGGTCTGAAAGCCTCTGGCCGCTGCCTATCAGGACCAACAAGACCAGC
CTGTGATACCACACCCCTTCCGTGTCGGCGACACCGTGTGGGTACGCCGGCACCAGACTAAGAACTTGGAACCTCGCTGG
AAAGGACCCTACACCGTCCTGCTGACCACCCCCACCGCTCTCAAAGTAGACGGCATCGCTGCGTGGATCCACGCCGCTCA
CGTAAAAGCGGCGACAACCCCTCCGGCCGGAACAGCATCAGGACCGACATGGAAGGTCCAGCGTTCTCAAAACCCCTTAA
AGATAAGATTAACCCGTGGGCCCCCCTGATAGTCCTGGGGATCTTAATAAGGGCAGGAGTATCAGTACCACATGACAGCC
CTCATCAGGTCTTCAATGTTACTTGGAGAGTTACCAACTTAATGACAGGACAAACAGCTAATGCTACCTCCCTCCTGGGG
ACAATGACCGATGCCTTTCCCAAACTGTACTTTGACTTGTGCGATTTAATAGGGGACGACTGGGATGAGACTGGACTCGG
GTGTCGCACTCCCGGGGGAAGAAAAAGGGCAAGAACATTTGACTTCTATGTTTGCCCCGGGCATACTGTACCAACAGGGT
GTGGAGGCCCGAGAGAGGGCTACTGTGGCAAATGGGGCTGTGAGACCACTGGACAGGCATACTGGAAGCCATCATCATCA
TGGGACCTAATTTCCCTTAAGCGAGGAAACACCCCTCGGAATCAGGGCCCCTGTTATGATTCCTCAGCGGTCTCCAGTGG
CATCCAGGGTGCCACACCGGGGGGTCGATGCAATCCCCTAGTCCTAGAATTCACTGACGCGGGTAAAAAGGCCAGCTGGG
ATGGCCCCAAAGTATGGGGACTAAGACTGTACCGATCCACAGGAACCGACCCGGTGACCCGGTTCTCTTTGACCCGCCAG
GTCCTCAATATAGGGCCCCGCATCCCCATTGGGCCTAATCCCGTGATCACTGACCAGTTACCCCCCTCCCGACCCGTGCA
GATCATGCTCCCCAGGCCTCCTCAGCCTTCCCCTACAGGCGCAGCCTCTATACAACCTGGGACGGGAGACAGACTGCTGA
ACCTGGTAGATGGAGCCTACCAAGCACTCAACCTCACCAGTCCTGACAAAACCCAAGAGTGCTGGTTGTGTCTGGTATCG
GGACCCCCCTATTACGAAGGGGTTGCCGTCCTAGGTACCTACTCCAACCATACCTCTGCCCCAGCTAACTGCTCCGTGGC
CTCCCAACACAAGCTGACCCTGTCCGAAGTAACCGGACAGGGACTCTGCGTAGGAGCAGTTCCCAAAACCCATCAGGCCC
TGTGTAATACCACCCAGAATACAAGCGACGGGTCCTACTATCTGGCTGCTCCCGCCGGGACCATTTGGGCTTGCAACACC
GGGCTCACTCCCTGCCTATCTACTACTGTACTCAACCTCACCACCGATTACTGTGTCCTGGTTGAGCTCTGGCCAAAGGT
GACCTACCACTCCCCTGGTTATGTTTATGGCCAGTTTGAGAGAAAAACCAAATATAAAAGAGAGCCGGTGTCATTAACTC
TGGCCCTGCTGTTGGGAGGACTTACTATGGGCGGCATAGCTGCAGGAGTAGGAACAGGGACTACAGCCCTAGTGGCCACC
AAGCAATTCGAGCAGCTCCAGGCAGCCATACATACAGACCTTGGGGCCTTAGAAAAATCAGTCAGTGCCCTAGAAAAGTC
TCTGACCTCGTTGTCTGAGGTGGTCCTACAGAACCGGAGAAGATTAGATCTACTGTTCCTAAAAGAAGGAGGATTATGTG
CTGCCCTAAAAGAAGAATGCTGTTTCTACGCGGACCACACTGGCGTAGTGAGAGATAGCATGGCAAAGCTAAGAGAAAGG
TTAAACCAGAGACAAAAATTGTTCGAATCAGGACAAGGGTGGTTTGAGGGACTGTTTAACAGGTCCCCATGGTTCACGAC
CTTGGTATCCACCATTATGGGCCCCTTGATAATACTCTTATTAATCCTACTCTTCGGACCCTGTATTCTCAACCGCTTGG
TCCAGTTTGTAAAAGACAGAATTTCGGTGGTGCAGGCCCTGGTTCTGACCCAACAGTATCACCAACTCAAATCAATAGGT
CCAGAAGAAGTAGAATCACGTAAATAAAAGATTTTATTCAGTTTCCAGAAAGAGGGGGGAATGAAAGACCCCACCATAAG
GCTTAGCAAGCTAGCTGCAGTAACGCCATTTCGCAAGGCATGAAAAAGTACCAGAGCTGGGTTCTCAAAAGTTACAAGGA
AGTTCAGTTATAGATTAACAGTTAAAGATCAAGGCTGAATAGCACTGGGACAGGGGCCGAACAGGATATCGGTGGTCAAG
CACCTGGGCCCCGGCTCAGGGCCAAGAACAGATGGTTCTCAGATAAAGCGGAACCAGCAACAGACACAGAAGCCCCGATA
GACGTCAGTGTTCGCAGAACTAGCTTCACTGATTTAAAAAAATAGAGGTGCACAATGCTCTGGTCACTCCTTAAACCTGT
GTGTCTGCCAATGTTCTGACCAGATATGTGCCCATTGCTGAACCTTCATTAGACTCTTTCCTTGTACCCCTCCCCTACCC
ATTTCTTGAAAATAGACATTGTTTAGATCTAAAAAGTCCCACCTCAGTTTCCCCAAATGACCGGGAAGTACCCCAAACCT
TATTCGAACTAACCAACCAGCTCGCTTCTCGCTTCTGTAACCACGCTTTTTGCTCCCCAGCCCTAGCCCTATAAAAAGGG
TAAAAACTCCACACTCGGCGCGCCAGTCCTCCGATAGACTGAGTCGCCCGGGTACCCGTGTTCCCAATAAAGCCTCTTGC
TGTTTACATCCGAATCGTGGACTCGCTGATCCTTGGGAGGGTCTCCTCAGATTGATTGACTGCCCACCTGGGGGGTCTTT
CA
>Mpmv9
TGAAAGACCCCACCATAAGGCTTAGCAAGCTAGCTGTAGTAACGCCATTTTGCAAGGCATGAAAAAGTACCCGAGCTGGG
TTCTCAAAAGTTACAAGGAAGTTCAGTTATAGATTAACAGTTAAAGATCAAGGCTGAATAGCACTGGGACAGGGGCCGAA
CAGGATATCGGTGATCAAGCACCTGGGCCCCGGCTCAGGGCCAAGAACAGATGGTTCTCAGATAAAGCGGAACCAGCAAC
AGACACAGAAGCCCCGATAGACGTCAGTGTTAGCAGAACTAGCTTCACTGATTTAGAAAAATAGAGGTGCACAGTGCTCT
GGTCACTCCTTGAACCTGTGTGTCTGCCAATGTTCTGACCAGATATGTGCCCATTGCTGAACCTTCATTAGACTCTTTCC

TTGTACCCCTCCCCTACCCATTTCTTGAAAATAGACATTGTTTAGATCTAAAAAGTCCCACCTCAGTTTCCCCAAATGAC
CGGGAAGTACCCCAAACCTTATTCGAACTAACCAACCAGCTCGCTTCTCGCTTCTGTAACCGCGCTTTTTGCTCCCCAGC
CCTAGCCCTATAAAAAGGGTAAAAACTCCACACTCGGCGCGCCAGTCCTCCGATAGACTGAGTCGCCCGGGTACCCGTGT
TCCCAATAAAGCCTCTTGCTGTTTACATCCGAATCGTGGACTCGCTGATCCTTGGGAGGGTCTCCTCAGATTGATTGACT
GCCCACCTGGGGGGGTCTTTCATTTGGAGGTTCCACCGAGATCAGGAGACCCCTGCCCAGGGACCACCGACCCCCGCCGG
GAGGTAAGCTGGCCAGCGGTCGTTTCGTGTCTGTCTCTGTCTCCGTGCGTGTTTGTGCCGGCATCTAATGTTTGCGCCTG
CGTCTGTACTAGTTGGCTAACTAGATCTGTATCTGGCGGTTCCGCGGAAGAACTGACGAGTTCGTATTCCCGGCCGCAGC
CCCTGGGAGACGTCCCAGCGGCCTCGGGGGCCCGTTTTGTGGCCCATTCTGTATCAGTTAACCTACCCGAGTCGGACTTT
TTGGAGCTCCGCCACTGTACGTGGCTTTGTTGGGGGACGAGAGACAGAGACACTTCCCGCCCCCGTCTGGATTTTTGCTT
TCGGTTTTACGCCGAAACCGCGCTGCGCGTCTGATTTGTTTTATTGCTCTTTTGTTCTTCGTTAGTTTTTTCTGTCTTTA
AGTGTTTTCAAGATCATGGGACAGACCGTAACTACCCCTCTGAGTCTAACCTTGCAGCACTGGGGAGATGTCCAGCGCAT
TGCATCCAACCAGTCTGTGGATGTCAGGAAGAGGCGCTGGATTACCTTCTGTTCCGCCGAATGGCCAACTTTCAATGTGG
GATGGCCTCAGGATGGTACTTTCAATTTAAGTATTATCTCTCAGGTTAAGTCTAGAGTGTTTTGTCCTGGTCCCCACGGA
CACCCGGATCAGGTCCCATATATCGTTACCTGGGAGGCACTTGCCTATGACCCCCCTCCGTGGGTCAAACCGTTTGTTTC
TCCTAAACCTCCTCCCTTGCCGACAGCTCCCGTCCTCCCGCCCGGTCCTTCTGCGCAACCTCCGTCCCGATCTGCCCTTT
ACCCTGCCCTTACCCCCTCTATAAAGTCCAAACCTCCTAAGCCCCAGGTTCTCCCTGATAGCGGCGGACCTCTCATTGAC
CTTCTCACAGAGGACCCCCCGCCGTACGGAGCACAACCTTCCTCCTCTGCCAGAGAGAACGATGAAGAAGAGGCGGCCAC
CACCTCCGAGGTTTCCCCCCCTTCTCCCATGGCGTCTCGACTGCGGGGAAGGAGAGACCCTCCCGCAGCGGACTCCACCT
CCTCCCAGGCATTCCCACTCCGTATGGGGGGAGATGGCCAGCTTCAGTACTGGCCGTTTTCCTCCTCTGACTTATATAAT
TGGAAAAATAATAACCCTTCCTTTTCTGAAGATCCAGGTAAATTGACGGCCTTGATTGAGTCCGTCCTCATCACCCACCA
GCCCACCTGGGACGACTGTCAGCAGTTGTTGGGGACCCTGCTGACCGGAGAAGAAAAGCAGCGGGTGCTCCTAGAGGCTA
GAAAGGCAGTCCGGGGCAATGATGGACGCCCCACTCAGTTGCCTAATGAAATCAATGCTGCCTTTCCCCTTGAGCGCCCT
GATTGGGATTACACCACTACAGAAGGTAGGAACCACCTAGTCCTCTATCGCCAGTTGCTCTTAGCGGGTCTCCAAAACGC
GGGCAGAAGCCCCACCAATTTGGCCAAGGTAAAAGGGATAACCCAGGGACCTAATGAGTCTCCCTCAGCCTTTTTAGAGA
GACTCAAGGAGGCCTATCGCAGGTACACTCCTTATGACCCTGAGGACCCAGGGCAAGAAACCAATGTGTCTATGTCATTC
ATCTGGCAGTCTGCCCCGGATATCGGGCGAAAGTTAGAGCGGTTAGAAGATTTAAAGAGCAAGACCTTAGGAGACTTAGT
GAGGGAAGCTGAAAAGATCTTTAATAAACGAGAAACCCCGGAAGAAAGAGAGGAACGTATCAGGAGAGAAACAGAGGAAA
AAGAAGAACGCCGTAGGGCAGAGGATGAGCAGAAAGAGAAAGAAAGGGACCGCAGAAGACATAGAGAGATGAGCAAGCTC
TTGGCCACTGTAGTTATTGGTCAGAGACAGGATAGACAGGGGGGAGAGCGGAAGAGGCCCCAACTTGATAAGGATCAATG
CGCCTACTGCAAAGAAAAGGGACACTGGGCTAAAGACTGCCCAAAGAAGCCACGAGGGCCCCGAGGACCGAGGCCCCAGA
CCTCCCTCCTGACCTTAGGTGACTAGGGAGGTCAGGGTCAGGAGCCCCCCCCTGAACCCAGGATAACCCTCAAAGTCGGG
GGGCAACCCGTCACCTTCCTGGTGGATACTGGGGCCCAACACTCCGTGCTGACCCAAAATCCTGGACCCCTAAGTGACAA
GTCTGCCTGGGTCCAAGGGGCTACTGGAGGAAAGCGGTATCGCTGGACCACGGATCGCAAAGTGCATCTAGCTACCGGTA
AGGTCACCCACTCTTTCCTCCATGTACCAGACTGCCCCTATCCTCTGCTAGGAAGAGATTTGCTGACTAAACTAAAAGCC
CAAATCCACTTTGAGGGATCAGGAGCTCAGGTTGTGGGACCAATGGGACAGCCCCTGCAAGTGCTGACCCTAAACATAGA
AGATGAGTATCGGCTACATGAGACCTCAAAAGAGCCGGATGTTTCTCTAGGGTCCACATGGCTTTCTGATTTTCCCCAGG
CCTGGGCGGAAACCGGGGGCATGGGACTGGCAGTTCGCCAAGCTCCTCTGATCATACCTCTGAAGGCAACCTCTACCCCC
GTGTCCATAAAACAATACCCCATGTCACAAGAAGCCAGACTGGGGATCAAGCCCCACATACAGAGACTGTTGGACCAGGG
AATACTGGTACCCTGCCAGTCCCCCTGGAACACGCCCCTGCTACCCGTTAAGAAACCAGGGACTAATGATTACAGGCCTG
TCCAAGATCTGAGAGAAGTCAACAAGCGGGTGGAAGACATCCACCCCACCGTGCCCAACCCTTACAACCTCTTGAGCGGG
CTCCCACCGTCCCACCAGTGGTACACTGTGCTTGACTTAAAGGATGCCTTTTTCTGCCTGAGACTCCACCCCACCAGTCA
GCCTCTCTTCGCCTTTGAGTGGAGAGACCCAGAGATGGGAATCTCAGGACAATTAACCTGGACCAGACTCCCACAGGGTT
TCAAAAACAGTCCCACCCTGTTTGATGAGGCACTGCACAGAGACCTAGCAGGCTTCCGGATCCAGCACCCAGACTTGATC
CTGCTACAGTACGTGGATGACTTACTGCTGGCCGCCACTTCTGAGCTCGACTGCCAACAAGGTACTCGGGCCCTGTTACA
AACCCTAGGGGACCTCGGGTATCGGGCCTCGGCCAAGAAAGCCCAAATTTGCCAGAAACAGGTCAAGTATCTGGGGTATC
TTCTAAAAGAGGGTCAGAGATGGCTGACTGAGGCCAGAAAAGAGACTGTGATGGGGCAGCCTATTCCGAAGACCCCTCGA
CAACTAAGGGAGTTCCTAGGGACGGCAGGCTTCTGTCGCCTCTGGATCCCTGGGTTTGCAGAAATGGCAGCCCCCTTGTA
TCCTCTCACCAAAACGGGGACTCTGTTTAATTGGGGCCCAGACCAGCAAAAGGCCTATCAAGAAATCAAACAGGCTCTTC
TAACTGCCCCAGCCCTGGGGTTGCCAGATTTGACTAAGCCCTTTGAACTCTTTGTCGACGAGAAGCAGGGCTACGCCAAA
GGCGTCCTAACGCAAAAACTGGGACCTTGGCGTCGGCCGGTGGCCTACCTGTCCAAAAAGCTAGACCCAGTGGCAGCTGG
GTGGCCCCCTTGCCTACGGATGGTAGCAGCCATTGCCGTTCTGACAAAGGATGCAGGCAAGCTAACTATGGGACAGCCGC
TAGTCATTCTGGCCCCCCATGCGGTAGAAGCACTGGTCAAACAACCCCCTGACCGCTGGCTATCCAATGCTCGCATGACC
CACTATCAGGCAATGCTCCTAGATACGGACCGAGTTCAGTTCGGACCGGTGGTAGCCTTAAACCCGGCCACGTTGCTCCC
CTTGCCGGGAAAAGAGACCCCCCACGACTGCCTCGAGATCTTGGCTGAGACACACGGCACCAGACCGGACCTCACGGACC
AGCCCCTCCCAAATGCCGACCACACCTGGTATACAGATGGAAGCAGCTTCCTGCAGGAGGGGCAACGTAAGGCTGGAGCA
GCGGTGACCACCGAGACCGAGGTAATCTGGGCCAAGGCGTTGCCAGCCGGGACATCCGCCCAGCGAGCTGAACTAATAGC
ACTCACCCAGGCCCTAAAGATGGCAGAAGGTAAGAAGCTAAATGTTTATACTGATAGCCGCTATGCCTTTGCTACCGCCC

ATGTCCATGGAGAAATATATAGGAGACGTGGGTTGCTCACCTCAGAAGGCAAGGAGATCAAGAACAAGGGCGAAATCTTG
GCCTTACTGAAAGCTCTCTTTCTGCCCAAAAGACTCAGTATAATTCACTGCCCAGGACATCAGAAAGGCAATAGTGCTGA
AGCTAAAGGCAACCGAATGGCGGACCAGGCAGCCCGGGAAGCAGCCATGGGGACTGACACAAAGGCCTCCTCACTTCTCA
TAGAGACCTCAACCCCGTACACTCCAGACTTCTTCCATTATACTGAAACAGATATAAAGAACCTACGAGAGTTGGGAGCC
ACATATGATAGAGAGAAAAAATATTGGGTCCTGCAAGGTAAACCTGTGATGCCTGACCAGTTCACCTTTGAATTATTAGA
CTTCCTTCACCAGCTCACCCACCTTAGCTATCAGAAGATGAGGGCACTTCTAGACAGAAAAGAAAGCCCCTATTACATGC
TAAATAAAGATAAGATCCTCCACGAGGTGGCGGAATCATGCCAAGCCTGTGTCCAAGTAAATGCCAGTAAAGCTAAGGTC
GGTCCCGGGGTGCGAGTAAGAGGACATCGACCAGGCACCCATTGGGAAATTGACTTTACTGAAGTAAGGCCCGGACTGTA
TGGGCATAAGTATCTTCTGGTGTTTGTGGACACGTTCTCTGGCTGGGTGGAAGCCTTCCCAACCAAGCATGAGACTGCCA
AAGTTGTGACCAAAAAGCTTCTAGAAGAAATATTTCCAAGGTTTGGAATGCCCCAAGTATTGGGGACTGATAATGGGCCT
GCCTTCGTCTCCCAGGTAAGTCAGTCGGTGGCCAAGCTACTGGGGATTGATTGGAAACTACATTGTGCTTACAGACCCCA
GAGTTCAGGTCAGGTAAAAAGAATGAATAGGACAATCAAGGAGACTTTGACCAAATTAACGCTTGCAACTGGCACTAGAG
ACTGGGTACTCCTACTTCCCTTGGCCCTCTACCGAGCCCGCAACACTCCGGGCCCCCATGGACTCACTCCGTATGAAATC
CTGTATGGGGCGCCCCCGCCCCTTGTTAATTTCCATGATCCTGAAATGTCAAAGTTTACTAATAGCCCCTCTCTCCAAGC
TCACTTACAGGCCCTCCAAGCGGTACAACGAGAGGTCTGGAAGCCTCTGGCCGCTGCCTATCAGGACCAACAAGACCAGC
CTGTGATACCACACCCCTTCCGTGTCGGCGACACCGTGTGGGTACGCCGGCACCAGACTAAGAACTTGGAACCTCGCTGG
AAAGGACCCTACACCGTCCTGCTGACCACCCCCACCGCTCTCAAAGTAGACGGCATCGCTGCGTGGATCCACGCCGCTCA
CGTAAAAGCGGCGACAACCCCTCCGGCCGGAACAGCATCAGGACCGACATGGAAGGTCCAGCGTTCTCAAAACCCTTTAA
AGATAAGATTAACCCGTGGGCCCCCCTGATAGTCCTGGGGATCTTAATAAGGGCAGGAGTATCAGTACCACATGACAGCC
CTCATCAGGTCTTCAATGTTACTTGGAGAGTTACCAACTTAATGACAGGACAAACAGCTAATGCTACCTCCCTCCTGGGG
ACAATGACCGATGCCTTTCCCAAACTGTACTTTGACTTGTGCGACTTAATAGGGGACGACTGGGATGAGACTGGACTCGG
GTGTCGCACTCCCGGGGGAAGAAAAAGGGCAAGAACATTTGACTTCTATGTTTGCCCCGGGCATACTGTACCAACAGGGT
GTGGAGGCCCGAGAGAGGGCTACTGTGGCAAATGGGGCTGTGAGACCACTGGACAGGCATACTGGAAGCCATCATCATCA
TGGGACCTAATTTCCCTTAAGCGAGGAAACACCCCTCGGAATCAGGGCCCCTGTTATGATTCCTCAGCGGTCTCCAGTGG
CATCCAGGGTGCCACACCGGGGGGTCGATGCAATCCCCTAGTCCTAGAATTCACTGACGCGGGTAAAAAGGCCAGCTGGG
ATGGCCCCAAAGTATGGGGACTAAGACTGTACCGATCCACAGGAACCGACCCGGTGACCCGGTTCTCTTTGACCCGCCAG
GTCCTCAATATAGGGCCCCGCATCCCCATTGGGCCTAATCCCGTGATCACTGACCAGTTACCCCCCTCCCGACCCGTGCA
GATCATGCTCCCCAGGCCTCCTCAGCCTTCCCCTACAGGCGCAGCCTCTATACAACCTGGGACGGGAGACAGACTGCTGA
ACCTGGTAGATGGAGCCTACCAAGCACTCAACCTCACCAGTCCTGACAAAACCCAAGAGTGCTGGTTGTGTCTGGTATCG
GGACCCCCCTATTACGAAGGGGTTGCCGTCCTAGGTACCTACTCCAACCATACCTCTGCCCCAGCTAACTGCTCCGTGGC
CTCCCAACACAAGCTGACCCTGTCCAAAGTAACCGGACAGGGACTCTGCGTAGGAGCAGTTCCCAAAACCCATCAGGCCC
TGTGTAATACCACCCAGAATACAAGCGACGGGTCCTACTATCTGGCTGCTCCCGCCGGGACCATTTGGGCTTGCAACACC
GGGCTCACTCCCTGCCTATCTACTACTGTACTCAACCTCACCACCGATTACTGTGTCCTGGTTGAGCTCTGGCCAAAGGT
GACCTACCACTCCCCTGGTTATGTTTATGGCCAGTTTGAGAGAAAAACCAAATATAAAAGAGAGCCGGTGTCATTAACTC
TGGCCCTGCTGTTGGGAGGACTTACTATGGGCGGCATAGCTGCAGGAGTAAGAACAGGGACTACAGCCCTAGTGGCCACC
AAGCAATTCGAGCAGCTCCAGGCAGCCATACATACAGACCTTGGGGCCTTAGAAAAATCAGTCAGTGCCCTAGAAAAGTC
TCTGACCTCGTTGTCTGAGGTGGTCCTACAGAACCGGAGAGGATTAGATCTACTGTTCCTAAAAGAAGGAGGATTATGTG
CTGCCCTAAAAGAAGAATGCTGTTTCTACGCGGACCACACTGGCGTAGTGAGAGATAGCATGGCAAAGCTAAGAGAAAGG
TTAAACCAGAGACAAAAATTGTTCGAATCAGGACAAGGGTGGTTTGAGGGACTGTTTAACAGGTCCCCATGGTTCACGAC
CTTGATATCCACCATTATGGGCCCCTTGATAATACTCTTATTAATCCTACTCTTCGGACCCTGTATTCTCAACCGCTTGG
TCCAGTTTGTAAAAGACAGAATTTCGGTGGTGCAGACCCTGGTTCTGACCCAACAGTATCATCAACTCAAATCAATAGGT
CCAGAAGAAGTGGAATCACGTGAATAAAAGATTTTATTCAGTTTCCAGAAAGAGGGGGGAATGAAAGACCCCACCATAAG
GCTTAGCAAGCTAGCTGTAGTAACGCCATTTTGCAAGGCATGAAAAAGTACCCGAGCTGGGTTCTCAAAAGTTACAAGGA
AGTTCAGTTATAGATTAACAGTTAAAGATCAAGGCTGAATAGCACTGGGACAGGGGCCGAACAGGATATCGGTGATCAAG
CACCTGGGCCCCGGCTCAGGGCCAAGAACAGATGGTTCTCAGATAAAGCGGAACCAGCAACAGACACAGAAGCCCCGATA
GACGTCAGTGTTAGCAGAACTAGCTTCACTGATTTAGAAAAATAGAGGTGCACAGTGCTCTGGTCACTCCTTGAACCTGT
GTGTCTGCCAATGTTCTGACCAGATATGTGCCCATTGCTGAACCTTCATTAGACTCTTTCCTTGTACCCCTCCCCTACCC
ATTTCTTGAAAATAGACATTGTTTAGATCTAAAAAGTCCCACCTCAGTTTCCCCAAATGACCGGGAAGTACCCCAAACCT
TATTCGAACTAACCAACCAGCTCGCTTCTCGCTTCTGTAACCGCGCTTTTTGCTCCCCAGCCCTAGCCCTATAAAAAGGG
TAAAAACTCCACACTCGGCGCGCCAGTCCTCCGATAGACTGAGTCGCCCGGGTACCCGTGTTCCCAATAAAGCCTCTTGC
TGTTTACATCCGAATCGTGGACTCGCTGATCCTTGGGAGGGTCTCCTCAGATTGATTGACTGCCCACCTGGGGGGGTCTT
TCA
>Pmv1
TGAAAGACCCCACCATCAGGCTTAGCAAGCTAGCTGCAGTAACGCCATTTTGCAAGGCATGAAAAAGTACCAGAGCTGAG
TTCTCAAAAGTTACAAGAAAGTTCAGTTAAAGATTAACAGTTAAAGATTAAGGCTGAATAATACTGGGACAGGGGCCAAA
TATCGGTGGTCAAGCACCTGGGCCCCGGCTCAGGGCCAAGAACAGATGGCTCTCAGACGTCAGTGTTAGCAGAACTAGCT
TCACTGATTTAGAAAAATAGAGGTGCACAGTGCTCTGGCCACTCCTTGAACCTGTGTGTCTGCCAATGTTCTGACCAGGT

GTGTGCCCATTGTTGAACCTTCATTAGACCCTTTCCTCGTACCCCTCCCATACCCATTTCTTGAAAATAGACATTGTTTA
GAACTAAAAAGTCCCACCTCAGTTTCCCCAAATGACCGAGAAATACCCCAAGCCTTATTCGAACTAACCAACCAGCTCGC
TTCTCGCTTCTGTAACCGCGCTTTTTGCTCCCCAGCCCCAGCCCTATAAAAAGGGTAAAAACTCCACACTCGGCGCGCCA
GTCCTCCGATAGACTGCGTCGCCCGGGTACCCGTGCTCCCAATAAAGCCTCTTGCTGTTTGCATCCGAATCGTGGTCTCG
CTGGTCCTTGAGAGGGTCTCCTCAGATTGATTGACTACCCACGTCGGGGGTCTTTCATTTGGAGGTCCCACCGAGATTTG
GAGACCCCTGCCCAGGGACCACCGACCCCCCGCCGGGAGGTAAGCTGGCCAGCGGTCGTTTCGTGTCTGTCTCTGTCTTC
GTGCGTGTTTGTGCCGGCATCCAATGTTTGCGCCTGCGTCTGTACTAGTTAGCTAACTAGATCTGTATCTGGCGGTTCCG
CGGAAGAACTGACGAGTTCGTATTCCCGGCCGCAGCCCCTGGGAGACGTCCCAGCGGCCTCGGGGGCCCGTTTTGTGGCC
CATTCTGTATCAGTTAACCTACCCGAGTCGGACTTTTTGGAGCTCCGCCACTGTACGTGGCTTTGTTGGGGGACGAGAGA
CAGAGACACTTCCCGCCCCCGTCTGAATTTTTGCTTTCGGTTTTACGCCGAAGCCGCGCCGCGCGTCTGATTTGTTTGTT
GTTCTTTTGTTCTTCGTTAGTTTTCTTCTGTCTTTAAGTGTTTTCGAGATCATGGGACAGACCGTAACTACCCCTCTGAG
TTTAACCTTGCAGCACTGGGGAGATGTCCAGCGCATTGCATCCAACCAGTCTGTGGATGTCAAGAAGAGGCGCTGGATTA
CCTTCTGTTCCGCTGAATGGCCAACTTTCAATGTGGGATGGCCTCAGGATGGTACTTTCAATTTAAGTATTATCTCTCAG
GTTAAGTCTAGAGTGTTTTGTCCTGGTCCCCACGGACACCCGGATCAGGTCCCATATATCGTCACCTGGGAGGCACTTGC
CTATGACCCCCCTCCGTGGGTCAAACCGTTTGTGTCTCCTAAACTTCCTCCCTTGCCGACAGCTCCCGTCCTCCCGCCCG
GTCCTTCTGCGCAACCTCCGTCCCGATCTGCCCTTTACCCTGCCCTTACCCCCTCTATAAAGTCCAAACCTCCTAAGCCC
CAGGTTCTCCCTGATAGCGGCGGACCCCTCATTGACCTTCTCACAGAGGACCCCCCGCCGTACAGAGCACAACCCTCCTC
CTCTGCCAGGGAGAACGACGAAGAAGAGGCGGCCACCACCTCCGAGGTTTCCCCCCCTTCTCCCATGGTGTCTCGACTGC
GGGGAAGGAGAGACCCTCCCGCAGTGGACTCCACCACCTCCCAGGCATTTCCACTCCGCATGGGGGGAGATGGCCAGCTT
CAGTATTGGCCGTTTTCCTCTTCGGATTTATACAATTGGAAAAATAATAACCCTTCCTTTTCTGAAGATCCAGGTAAATT
GACGGCCTTGATTGAGTCCGTCCTCATCACCCACCAGCCCACCTGGGACGACTGTCAGCAGTTGTTGGGGACCCTGCTGA
CCGGAGAAGAAAAGCAGCGGGTGCTCCTAGAGGCTAGAAAGGCAGTCCGGGGCAATGATGGACGCCCCACTCAGTTGCCT
AATGAAGTCAATGCTGCTTTTCCCCTTGAACGCCCCGGTTGGGATTACACCACTACAGAAGGTAGGAACCACCTAGTCCT
CTATCGCCAGTTGCTCTTAGCGGGTCTCCAGAACGCGGGCAGAAGTCCCACCAATTTGGCCAAGGTAAAAGGGATAACCC
AGGGACCTAATGAGTCTCCCTCAGCCTTTTTAGAGAGACTCAAAGAGGCCTATCGCAGGTACACTCCTTATGACCCTGAG
GACCCAGGGCAAGAAACCAATGTGTCTATGTCATTCATCTGGCAGTCTGCCCCGGATATCGGGCGAAAGTTAGAGCGGTT
AGAAGATTTAAAGAGCAAGACCTTAGGAGACTTAGTGAGGGAAGCTGAAAAGATCTTTAATAAGCGAGAAACCCCGGAAG
AAAGAGAGGAACGTATCAAGAGAGAAACAGAGGAAAAAGAAGAACGCCGTAGGGCAGAGGATGAGCAGAGAGAGAAAGAA
AGGGACCGCAGAAGACATAGAGAGATGAGCAAGCTCTTGGCCACTGTAGTTATTGGTCAGAGACAGGATAGACAGGGGGG
AGAGCGGAGGAGGCCCCAACTTGATAAGGACCAATGCGCCTACTGCAAAGAAAAGGGACACTGGGCTAAGGACTGCCCAA
AGAAGCCACGAGGGCCCCGAGGACTGAGGCCCCAGACCTCCCTCCTAACCTTAGGTGACTAGGGAGGTCAGGGTCAGGAG
CCCCCCCCTGAACCCAGGATAACCCTCAAAGTCGGGGGGCAACCCGTCACCTTCCTGGTAGATACTGGGGCCCAACACTC
CGTGCTGACCCAAAATCCTGGACCCCTAAGTGACAAGGCTGCCTGGGTCCAAGGGGCTACTGGAGGAAAGCGGTATCGCT
GGACCACGGATCGCAAAGTACATCTAGCTACCGGTAAGGTCACCCACTCTTTCCTCCATGTACCAGACTGCCCCTATCCT
CTGCTAGGAAGAGACTTGCTGACTAAACTAAAAGCCCAAATCCACTTCGAGGGATCAGGAGCTCAGGTTATGGGACCAAT
GGGACTGCCCCTGCAAGTGCTGACCCTAAACATAGAAGATGAGTATCGGCTACATGAGACCTCAAAAGAGCCGGATGTTT
CTCTAGGGTCCACCTGGCTTTCTGATTTTCCCCAGGTCTGGGCGGAAACCGGGGGCATGGGACTGGCAGTTCGCCAAGCT
CCTCTGATCATACCTCTGAAGGCAACCTCTACCCCCGTGTCCATAAAACAATACCCCATGTCACAAGAAGCCAGACTGGG
GATCAAGCCCCACATACAGAGACTGTTGGACCAGGGAATACTGGTACCCTGCCAGTCCCCCTGGAACACGCCCCTGCTAC
CCGTTAAGAAACCAGGGACTAATGATTATAGGCCTGTCCAGGATCTGAGAGAAGTCAACAAGCGGGTGGAAGACATCCAC
CCCACCGTGCCCAACCCTTACAACCTCTTGAGCGGGCTCCCACCGTCCCACCAGTGGTACACTGTGCTTGATTTAAAGGA
TGCCTTTTTCTGCCTGAGACTCCACCCCACCAGTCAGCCTCTCTTCGCCTTTGAGTGGAGAGATCCAGAGATGGGAATCT
CAGGACAATTGACCTGGACCAGACTCCCACAGGGTTTCAAAAACAGTCCCACCCTGTTTGATGAGGCACTGCACAGAGAC
CTAGCAGACTTCCGGATCCAGCACCCAGACTTGATCCTGCTACAGTACGTGGATGACTTACTGCTGGCCGCCACTTCTGA
GCTCGACTGCCAACAAGGTACTCGGGCCCTGTTACAAACCCTAGGGGACCTCGGGTATCGGGCCTCGGCCAAGAAAGCCC
AAATTTGCCAGAAACAGGTCAAGTATCTGGGGTATCTTCTAAAAGAGGGTCAGAGATGGCTGACTGAGGCCAGAAAAGAG
ACTGTGATGGGGCAGCCTACTCCGAAGACCCCTCGACAACTAAGGGAGTTCCTAGGGACGGCAGGCTTCTGTCGCCTCTG
GATCCCTGGGTTTGCAGAAATGGCAGCCCCCTTGTACCCTCTCACCAAAACGGGGACTCTGTTTAATTGGGGCCCAGACC
AGCAAAAGGCCTATCAAGAAATCAAACAGGCTCTTCTAACTGCCCCAGCCCTGGGATTGCCAGATTTGACTAAGCCCTTT
GAACTCTTTGTTGACGAGAAGCAGGGCTACGCCAAAGGCGTCCTAACGCAAAAACTGGGACCTTGGCGTCGGCCGGTGGC
CTACCTGTCCAAAAAGCTAGACCCAGTGGCAGCTGGGTGGCCCCCTTGCCTACGGATGGTAGCAGCCATTGCCGTTCTGA
CAAAAGATGCAGGCAAGCTAACCATGGGACAGCCGCTAGTCATCCTGGCCCCCCATGCAGTAGAGGCACTAGTCAAGCAA
CCCCCTGACCGCTGGCTATCCAACGCCCGCATGACCCACTATCAGGCGATGCTCCTAGATACGGACCGGGTCCAGTTCGG
ACCGGTGGTAGCCCTAAACCCGGCTACGTTGCTCCCCCTACCGGGGAAAGAGCCTCACCATGACTGCCTCGAGATCTTGG
CCGAGACACACGGAACCAGACCAGACCTCACGGACCAGCCCCTCCCAGACGCCGACCACACCTGGTATACAGATGGAAGC
AGCTTCCTGCAAGAGGGACAACGTAGGGCTGGAGCAGCGGTGACCACCGAGACCGAGGTAATCTGGGCCAAGGCGTTGCC
AGCCGGGACATCCGCCCAGCGAGCTGAACTAATAGCACTCACCCAGGCCCTAAAGATGGCAGAAGGTAAGAAGCTAAATG

TTTATACTGATAGCCGCTATGCCTTTGCTACCGCCCATATCCATGGAGAAATATATAGGAGACGTGGGTTGCTCACCTCA
GAAGGCAAGGAGATCAAGAACAAGGGCGAAATCTTGGCCTTACTGAAAGCTCTCTTTCTGCCCAAAAGACTCAGTATAAT
TCACTGCCCAGGACATCAGAAAGGCAATAGTGCTGAAGCTAAAGGCAACCGAATGGCGGACCAGGCAGCCCGGGAAGCGG
CCATGGGGACTGACACAAAGGCCTCCTCACTTCTCATAGAGACCTCAACCCCGTACACTCCAGACTTCTTCCATTATACT
GAGACAGATATAAAGAACCTACAAGAGTTGGGAGCCACATATGATAGAGAGAAAAAATATTGGGTCCTGCAAGGTAAACC
TGTGATGCCTGACCAGTTCACCTTTGAATTATTAGACTTCCTTCACCAGCTCACCCACCTTAGCTATCAGAAGATGAGGG
CACTTCTAGACAGGAAAGAAAGCCCCTATTACATGCTAAATAAAGATAAGATCCTCCACGAGGTGGCGGAATCATGCCAA
GCCTGTGTCCAAGTAAATGCCAGTAAGACTAAGATCAGGGCCGGAACACGAGTAAGAGGACATCGACCAGGCACCCATTG
GGAAATTGACTTTACTGAAGTGAAGCCCGGACTGTATGGGTACAAGTATCTCCTGGTATTCGTGGACACGTTCTCTGGCT
GGGTTGAAGCCTTCCCAACCAAACATGAGACTGCCAAAATAGTGACCAAGAAACTTCTGGAAGAAATATTTCCAAGGTTT
GGAATGCCCCAAGTGTTGGGGACTGATAATGGGCCTGCCTTCGTCTCCCAGGTAAGTCAGTCGGTGGCCAAGCTACTGGG
GATTGATTGGAAACTACATTGTGCTTACAGACCCCAGAGTTCAGGTCAGGTAGAAAGAATGAATAGGACAATCAAGGAGA
CTTTGACCAAATTAACGCTTGCAACTGGCACTAGAGACTGGGTACTCCTACTTCCCTTAGCCCTCTACCGAGCCCGCAAC
ACTCCGGGCCCCCATGGACTCACTCCGTATGAAATCCTGTATGGGGCGCCCCCGCCCCTTGTTAATTTCCATGATCCTGA
AATGTCAAAGTTTACTAATAGCCCCTCTCTCCAAGCTCACTTACAGGCCCTCCAAGCAGTACAACGAGAGGTCTGGAAGC
CACTGGCCGCTGCCTATCAGGACTAGCTGGACCAGCCAGTGATACCACACCCCTTCCGTGTCGGCGACACCGTGTGGGTA
CGCCGGCACCAGACTAAGAACTTGGAACCTCGCTGGAAAGGACCCTACACCGTCCTGCTGACCACCCCCACCGCTCTCAA
AGTAGACGGCATCGCTGCGTGGATCCACGCCGCTCACGTAAAAGCGGCGACAACCCCTCCGGCCGGAACAGCATCAGGAC
CGACATGGAAGGTCCAGCGTTCTCAAAACCCCTTAAAGATAAGATTAACCCGTGGGGCCCCCTAATAATCCTGGGGATCT
TAATAAGAGCAGGAGTATCAGTACAACATGACAGCCCTCATCAGGTCTTCAATGTTACTTGGAGAGTTACCAACTTAATG
ACAGGACAAACAGCTAATGCTACCTCCCTCCTGGGGACAATGACCGATGCCTTTCCTAAACTGTACTTTGACTTGTGCGA
TTTAATAGGGGACGACTGGGATGAGACTGGACTCGGGTGTCGCACTCCCGGGGGAAGAAAAAGGGCAAGAACATTTGACT
TCTATGTTTGCCCCGGGCATACTGTACCAACAGGGTGTGGAGGGCCGAGAGAGGGCTACTGTGGCAAATGGGGCTGTGAG
ACCACTGGACAGGCATACTGGAAGCCATCATCATCATGGGACCTAATTTCCCTTAAGCGAGGAAACACCCCTCGGAATCA
GGGCCCCTGTTATGATTCCTCAGCGGTCTCCAGTGACATCAAGGGCGCCACACCGGGGGGTCGATGCAATCCCCTAGTCC
TGGAATTCACTGACGCGGGCAAAAAGGCCAGCTGGGATGGCCCCAAAGTATGGGGACTAAGACTGTACCGATCCACAGGG
ACCGACCCGGTGACCCGGTTCTCTTTGACCCGCCAGGTCCTCAATATAGGGCCCCGCGTCCCCATTGGGCCTAATCCCGT
GATCACTGACCAGTTACCCCCCTCCCGACCCGTGCAGATCATGCTCCCCAGGCCTCCTCAGCCTCCTCCTCCAGGCGCAG
CCTCTATAGTCCCTGAGACTGCCCCACCTTCTCAACAACCTGGGACGGGAGACAGGCTGCTAAACCTGGTAGATGGAGCC
TACCAAGCTCTCAACCTCACCAGTCCTGACAAAACCCAAGAGTGCTGGTTGTGTCTGGTAGCGGGACCCCCCTACTACGA
AGGGGTTGCCGTCCTAGGTACTTATTCCAACCATACCTCTGCCCCAGCTAACTGCTCCGTGGCCTCCCAACACAAGCTGA
CCCTGTCCGAAGTGACCGGACAGGGACTCTGCGTAGGAGCAGTTCCCAAAACCCATCAGGCCCTGTGTAATACCACCCAG
AAGACGAGCGACGGGTCCTACTATCTGGCTGCTCCCGCCGGGACCATTTGGGCTTGCAACACCGGGCTCACTCCCTGCCT
ATCTACCACTGTACTCGACCTCACCACCGATTACTGTGTCCTGGTTGAGCTCTGGCCAAAGGTGACCTACCACTCCCCTG
GTTATGTTTATGGCCAGTTTGAGAAAAAAACCAAATATAAAAGAGAGCCGGTGTCATTAACTCTGGCCCTGCTGTTGGGA
GGACTTACTATGGGCGGCATAGCTGCAGGAGTAGGAACCGGGACTACAGCCCTAGTGGCCACCAAACAATTCGAGCAGCT
CCAGGCAGCCATACATACAGACCTTGGGGCTTTAGAAAAGTCAGTCAGTGCCCTAGAAAAGTCTCTGACCTCGTTGTCTG
AGGTGGTCCTACAGAACCGGAGGGGATTAGATCTACTGTTCCTAAAAGAAGGAGGATTATGTGCTGCCCTAAAAGAAGAA
TGCTGTTTCTACGCGGACCACACTGGCGTAGTAAGAGATAGCATGGCAAAGCTAAGAGAAAGGTTGAACCAGAGACAAAA
ATTGTTCGAATCAGGACAAGGGTGGTTTGAGGGACTGTTTAACAGGTCCCCATGGTTCACGACCTTAATATCTACCATTA
TGGGCCCCTTGATAATACTTTTATTAATCCTACTCTTCGGACCCTGTATTCTCAACCGCTTGGTCCAGTTTGTAAAAGAC
AGAATTTCGGTAGTGCAGGCCCTGGTTTTGACCCAACAGTATCACCAACTCAAATCAATAGATCCAGAAGAAGTGGAATC
ACGTGAATAAAAGATTTTATTCAGTTTCCAGAAAGAGGGGGGAATGAAAGACCCCACCATCAGGCTTAGCAAGCTAGCTG
CAGTAACGCCATTTTGCAAGGCATGAAAAAGTACCAGAGCTGAGTTCTCAAAAGTTACAAGAAAGTTCAGTTAAAGATTA
ACAGTTAAAGATTAAGGCTGAATAATACTGGGACAGGGGCCAAATATCGGTGGTCAAGCACCTGGGCCCCGGCTCAGGGC
CAAGAACAGATGGCTCTCAGACGTCAGTGTTAGCAGAACTAGCTTCACTGATTTAGAAAAATAGAGGTGCACAGTGCTCT
GGCCACTCCTTGAACCTGTGTGTCTGCCAATGTTCTGACCAGGTGTGTGCCCATTGTTGAACCTTCATTAGACCCTTTCC
TCGTACCCCTCCCATACCCATTTCTTGAAAATAGACATTGTTTAGAACTAAAAAGTCCCACCTCAGTTTCCCCAAATGAC
CGAGAAATACCCCAAGCCTTATTCGAACTAACCAACCAGCTCGCTTCTCGCTTCTGTAACCGCGCTTTTTGCTCCCCAGC
CCCAGCCCTATAAAAAGGGTAAAAACTCCACACTCGGCGCGCCAGTCCTCCGATAGACTGCGTCGCCCGGGTACCCGTGC
TCCCAATAAAGCCTCTTGCTGTTTGCATCCGAATCGTGGTCTCGCTGGTCCTTGAGAGGGTCTCCTCAGATTGATTGACT
ACCCACGTCGGGGGTCTTTCA
>Pmv10
TGAAAGACCCCACCATCAGGCTTAGCAAGCTAGCTGCAGTAACGCCATTTTGCAAGGCATGAAAAAGTACCAGAGCTAAG
TTCTCAAAAGTTACAAGAAAGTTCAGTTAAAGATTAACAGTTAAAGATTAAGGCTGAATAATACTGGGACAGGGGCCAAA
TATCGGTGGTCAAGCACCTGGGCCCCGGCTCAGGGCCAAGAACAGATGGCTCTCAGACGTCAGTGTTAGCAGAACTAGCT
TCACTGATTTAGAAAAATAGAGGTGCACAGTGCTCTGGCCACTCCTTGAACCTGTGTGTCTGCCAATGTTCTGACCAGGT

GTGTGCCCATTGTTGAACCTTCATTAGACCCTCTCCTCGTACCCCTCCCATACCCATTTCTTGAAAATAGACATTGTTTA
GAACTAAAAAGTCCCACCTCAGTTTCCCCAAATGACCGAGAAATACCCCAAGCCTTATTCGAACTAACCAACCAGCTCGC
TTCTCGCTTCTGTAACCGCGCTTTTTGCTCCCCAGCCCCAGCCCTATAAAAAGGGTAAAAACTCCACACTCGGCGCGCCA
GTCCTCCGATAGACTGCGTCGCCCGGGTACCCGTGTTCTCAATAAAGCCTCTTGCTGTTTGCATCCGAATCGTGGTCTCG
CTGGTCCTTGAGAGGGTCTCCTCAGATTGATTGACTACCCACGTCGGGGGTCTTTCATTTGGAGGTTCCACCGAGATTTG
GAGACCCCTGCCCAGGGACCACCGACCCCCCGCCGGGAGGTAAGCTGGCCAGCGGTCGTTTCGTGTCTGTCTCTGTCTTC
GTGCGTGTTTGTGCCGGCATCCAATGTTTGCGCCTGCGTCTGTACTAGTTAGCTAACTAGATCTGTATCTGGCGGGTCCG
CGGAAGAACTGACGAGTTCGTATTCCCGGCCGCAGCCCCTGGGAGACGTCCCAGCGGCCTCGGGGGCCCGTTTTGTGGCC
CATTCTGTATCAGTTAACCTACCCGAGTCGGACTTTTTGGAGCTCCGCCACTGTACGTGGCTTTGTTGGGGGACGAGAGA
CAGAGACACTTCCCGCCCCCGTCTGAATTTTTGCTTTCGGTTTTACGCCGAAGCCGCGCCGCGCGTCTGATTTGTTTGTT
GTTCTTTTGTTCTTCGTCAGTTTTCTTCTGTCTTTAAGTGTTTTCGAGATCATGGGACAGACCGTAACTACCCCTCTGAG
TTTAACCTTGCAGCACTGGGGGGATGTCCAGCGCATTGCATCCAACCAGTCTGTGGATGTCAGGAAGAGGCGCTGGATTA
CCTTCTGTTCCGCTGAATGGCCAACTTTCAATGTGGGATGGCCTCAGGATGGTACTTTCAATTTAAGTATTATCTCTCAG
GTTAAGTCTAGAGTGTTTTGTCCTGGTCCCCACGGACACCCGGATCAGGTCCCATATATCGTCACCTGGGAGGCACTTGC
CTATGACCCCCCTCCGTGGGTCAAACCGTTTGTGTCTCCTAAACTTCCTCCCTTGCCGACAGCTCCCGTCCTCCCGCCCG
GTCCTTCTGCGCAACCTCCGTCCCGATCTGCCCTTTACCCTGCCCTTACCCCCTCTATAAAGTCCAAACCTCCTAAGCCC
CAGGTTCTCCCTGATAGCGGCGGACCCCTCATTGACCTTCTCACAGAGGACCCCCCGCCGTACAGAGCACAACCCTCCT
CCTCTGCCAGGGAGAACGACGAAGAAGAGGCGGCCACCACCTCCGAGGTTTCCCCCCCTTCTCCCATGGTGTCTCGACTG
CGGGGAAGGAGAGACCCTCCCGCAGCGGACTCCACCACCTCCCAGGCATTCCCACTCCGCATGGGGGGAGATGGCCAGCT
TCAGTACTGGCCGTTTTCCTCCTCTGATTTAACCCTTCCTTTTCTGAAGATCCAGGTAAATTGACGGCCTTGATTGAGTC
CGTCCTCATCACCCACCAGCCCACCTGGGACGACTGTCAGCAGTTGTTGGGGACCCTGCTGACCGGAGAAGAAAAGCAGC
GGGTGCTCCTAGAGGCTAGAAAGGCAGTCCGGGGCGATGATGGACGCCCCACTCAGTTGCCTAATGAAGTCAATGCTGCT
TTTCCCCTTGAACGCCCCGGTTGGGATTACACCACTACAGAAGGTAGGAACCACCTAGTCCTCTATCGCCAGTTGCTCTT
AGCGGGTCTCCAGAACGCGGGCAGAAGTCCCACCAATTTGGCCAAGGTAAAAGGGATAACCCAGGGACCTAATGAGTCTC
CCTCAGCCTTTTTAGAGAGACTCAAAGAGGCCTATCGCAGGTACACTCCTTATGACCCTGAGGACCCAGGGCAAGAAACC
AATGTGTCTATGTCATTCATCTGGCAGTCTGCCCCGGATATCGGGCGAAAGTTAGAGCGGTTAGAAGATTTAAAGAGCAA
GACCTTAGGAGACTTAGTGAGGGAAGCTGAAAAGATCTTTAATAAGCGAGAAACCCCGGAAGAAAGAGAGGAACGTATCA
AGAGAGAAACAGAGGAAAAAGAAGAACGCCGTAGGGCAGAGGATGAGCAGAGAGAGAAAGAAAGGGACCGCAGAAGACAT
AGAGAGATGAGCAAGCTCTTGGCCACTGTAGTTATTGGTCAGAGACAGGATAGACAGGGGGGAGAGCGGAGGAGGCCCCA
ACTTGATAAGGACCAATGCGCCTACTGCAAAGAAACGGGACACTGGGCTAAGGACTGCCCCTGCCCTGGGATTGCCAGAT
TTGACTAAGCCCTTTGAACTCTTTGTTGACGAGAAGCAGGGCTACGCCAAAGGCGTCCTAACGCAAAAACTGGGACCTTG
GCGTCGGCCGGTGGCCTACCTGTCCAAAAAGCTAGACCCAGTGGCAGCTGGGTGGCCCCCTTGCCTACGGATGGTAGCAG
CCATTGCCGTTCTGACAAAAGATGCAGGCAAGCTAACCATGGGACAGCCGCTAGTCATCCTGGCCCCCCATGCAGTAGAG
GCACTAGTCAAGCAACCCCCTGACCGCTGGCTATCCAACGCCCGCATGACCCACTATCAGGCGATGCTCCTAGATACGGA
CCGGGTCCAGTTCGGACCGGTGGTAGCCCTAAACCCGGCTACGTTGCTCCCCCTACCGGGGAAAGAGCCTCACCATGACT
GCCTCGAGATCTTGGCCGAGACACACGGAACCAGACCAGACCTCACGGACCAGCCCCTCCCAGACGCCGACCACACCTGG
TATACAGATGGAAGCAGCTTCCTACAAGAGGGACAACGTAGGGCTGGAGCAGCGGTGACCACCGAGACCGAGGTAATCTG
GGCCAAGGCGTTGCCAGCCGGGACATCCGCCCAGCGAGCTGAACTAATAGCACTCACCCAGGCCCTAAAGATGGCAGAAG
GTAAGAAGCTAAATGTTTATACTGATAGCCGCTATGCCTTTGCTACCGCCCATGTCCATGGAGAAATATATAGGAGACGT
GGGTTGCTCACCTCAGAAGGCAAGGAGATCAAGAACAAGGGCGAAATCTTGGCCTTACTGAAAGCTCTCTTTCTGCCCAA
AAGACTCAGTATAATTCACTGCCCAGGACATCAGAAAGGCAATAGTGCTGAAGCTAAAGGCAACCGAATGGCGGACCAGG
CAGCCCGGGAAGCAGCCATGGGGACTGACACAAAGGCCTCCTCACTTCTCATAGAGACCTCAACCCCGTACACTCCAGAC
TTCTTCCATTATACTGAGACAGATATAAAGAACCTACAAGAGTTGGGAGCCACATATGATAGAGAGAAAAAATATTGGGT
CCTGCAAGGTAAACCTGTGATGCCTGACCAGTTCACCTTTGAATTATTAGACTTCCTTCACCAGCTCACCCACCTTAGCT
ATCAGAAGATGAGGGCACTTCTAGACAGGAAAGAAAGCCCCTATTACATGCTAAATAAAGATAAGATCCTCCACGAGGTG
GCGGAATCATGCCAAGCCTGTGTCCAAGTAAATGCCAGTAAGACTAAGATCAGGGCCGGAACACGAGTAAGAGGACATCG
ACCAGGCACCCATTGGGAAATTGACTTTACTGAAGTGAAGCCCGGACTGTATGGGTACAAGTATCTCCTGGTATTCGTGG
ACACGTTCTCTGGCTGGGTTGAAGCCTTCCCAACCAAACATGAGACTGCCAAAATAGTGACCAAGAAACTTCTGAAAGAA
ATATTTCCAAGGTTTGGAATGCCCCAAGTGTTGGGGACTGATAATGGGCCTGCCTTCGTCTCCCAGGTAAGTCAGTCGGT
GGCCAAGCTACTGGGGATTGATTGGAAACTACATTGTGCTTACAGACCCCAGAGTTCAGGTCAGGTACAAAGAATAAATA
GGACAATCAAGGAGACTTTGACCAAATTAACGCTTGCAACTGGCACTAGAGACTGGGTACTCCTACTTCCCTTGGCCCTC
TACCGAGCCCGCAACACTCCGGGCCCCCATGGACTCATTCCGTATGAAATCCTGTATGGGGCGCCCCCGCCCCTTGTTAA
TTTCCATGATCCTGAAATGTCAAAGTTTACTAATAGCCCCTCTCTCCAAGCTCACTTACAGGCCCTCCAAGCAGTACAAC
GAGAGGTCTGGAAGCCACTGGCCGCTGCCTATCAGGACCAGCTGGACCAGCCAGTGATACCACACCCCTTCCGTGTCGGC
GACACCGTGTGGGTACGCCGGCACCAGACTAAGAACTTGGAACTTCGCTGGAAAGGACCCTACACCGTCCTGCTGACCAC
CCCCACCGCTCTCAAAGTAGACGGCATCGCTGCGTGGATCCACGCCGCTCACGTAAAAGCGGCGACAACCCCTCCGGCCG
GAACAGCATCAGGACCGACATGGAAGGTCCAGCGTTCTCAAAACCCCTTAAAGATAAGATTAACCCGTGGGGCCCCCTAA

TAGTCCTGAGAATCTTAATAAGGGCAGGAGTATCAGTACAACATGACAGCCCTCATCAGGTCTTCAATGTTACTTGGAGA
GTTACCAACTTAATGACAGGACAAACAGCTAATGCTACCTCCCTCCTGGGGACAATGACCGATGCCTTTCCTAAACTGTA
CTTTGACTTGTGCGATTTAATAGGGGACGACTGGGATGAGACTGGACTCGGGTGTCGCACTCCCGGGGGAAGAAAAAGGG
CAAGAACATTTGACTTCTATGTTTGCCCCGGGCATACTGTACCAACAGGGTGTGGAGGGCCGAGAGAGGGCTACTGTGGC
AAATGGGGCTGTGAGACCACTGGACAGGCATACTGGAAGCCATCATCATCATGGGACCTAATTTCCCTTAAGCGAAGAAA
CACCCCTCAGAATCAGGGCCCCTGTTATGATTCCTCAGCGGTCTCCAGTGACATCAAGGGCGCCACACCGGGGGGTCGAT
GCAATCCCCTAGTCCTGGAATTCACTGACGCGGGCAAAAAGGCCAGCTGGGATGGCCCCAAAGTATGGGGACTAAGACTG
TACCGATCCACAGGGACCGACCCGGTGACCCGGTTCTCTTTGACCCGCCAGGTCCTCAATATAGGGCCCCGCGTCCCCAT
TGGGCCTAATCCCGTGATCACTGACCAGTTACCCCCCTCCCGACCCGTGCAGATCATGCTCCCCAGGCCTCCTCAGCCTC
CTCCTCCAGGCGCCGCCTCTATAGTCCCTGAGACTGCCCCACCTTCTCAACAACCTGGGACGGGAGACAGGCTGCTAAAC
CTGGTAGATGGAGCCTACCAAGCTCTCAACCTCACCAGTCCTGACAAAACCCAAGAGTGCTGGTTGTGTCTGGTAGCGGG
ACCCCCCTACTACGAAGGGGTTGCCGTCCTAGGTACTTATTCCAACCATACCTCTGCCCCAGCTAACTGCTCCGTGGCCT
CCCAACACAAGCTGACCCTGTCCGAAGTGACCGGACAGGGACTCTGCGTAAGAGCAGTTCCCAAAACCCATCAGGCCCTG
TGTAATACCACCCAGAAGACGAGCGACGGGTCCTACTATCTGGCTGCTCCCGCCGGGACCATTTGGGCTTGCAACACCGG
GCTCACTCCCTGCCTATCTACCACTGTGCTCGACCTCACCACCGATTACTGTGTCCTGGTTGAGCTCTGGCCAAAGGTGA
CCTACCACTCCCCTGGTTATGTTTATGGCCAGTTTGAGAGAAAAACCAAATATAAAAGAGAGCCGGTGTCATTAACTCTG
GCCCTGCTGTTGGGAGGACTTACTATGGGCGGCATAGCTGCAGGAGTAGGAACCGGGACTACAGCCCTAGTGGCCACCAA
ACAATTCGAGCAGCTCCAGGCAGCCATACATACAGACCTTGGGGCTTTAGAAAAGTCAGTCAGTGCCCTAGAAAAGTCTC
TGACCTCGTTGTCTGAGGTGGTCCTACAGAACCGGAGGGGATTAGATCTACTGTTCCTAAAAGAAGGAGGATTATGTGCT
GCCCTAAAAAAAGAATGCTGTTTCTACGCGGACCACACTGGCGTAGTAAGAGATAGCATGGCAAAGCTAAGAGAAAGGTT
GAACCAGAGACAAAAATTGTTCGAATCAGGACAAGGGTGGTTTGAGGGACTGTTTAACAGGTCCCCATGGTTCACGACCT
TAATATCCACCATTATGGGCCCCTTGATAATACTTTTATTAATCCTACTCTTCGGACCCTGTATTCTCAACCGCTTGGTC
CAGTTTGTAAAAGACAGAATTTCGGTAGTGCAGGCCCTGGTTTTGACCCAACAGTATCACCAACTCAAATCAATAGATCC
AGAAAAAGTGGAATCACGTGAATAAAAGATTTTATTCAGTTTCCAGAAAGAGGGGGGAATGAAAGACCCCACCATCAGGC
TTAGCAAGCTAGCTGCAGTAACGCCATTTTGCAAGGCATGAAAAAGTACCAGAGCTAAGTTCTCAAAAGTTACAAGAAAG
TTCAGTTAAAGATTAACAGTTAAAGATTAAGGCTGAATAATACTGGGACAGGGGCCAAATATCGGTGGTCAAGCACCTGG
GCCCCGGCTCAGGGCCAAGAACAGATGGCTCTCAGACGTCAGTGTTAGCAGAACTAGCTTCACTGATTTAGAAAAATAGA
GGTGCACAGTGCTCTGGCCACTCCTTGAACCTGTGTGTCTGCCAATGTTCTGACCAGGTGTGTGCCCATTGTTGAACCTT
CATTAGACCCTCTCCTCGTACCCCTCCCATACCCATTTCTTGAAAATAGACATTGTTTAGAACTAAAAAGTCCCACCTCA
GTTTCCCCAAATGACCGAGAAATACCCCAAGCCTTATTCGAACTAACCAACCAGCTCGCTTCTCGCTTCTGTAACCGCGC
TTTTTGCTCCCCAGCCCCAGCCCTATAAAAAGGGTAAAAACTCCACACTCGGCGCGCCAGTCCTCCGATAGACTGCGTCG
CCCGGGTACCCGTGTTCTCAATAAAGCCTCTTGCTGTTTGCATCCGAATCGTGGTCTCGCTGGTCCTTGAGAGGGTCTCC
TCAGATTGATTGACTACCCACGTCGGGGGTCTTTCA
>Pmv11
TGAAAGACCCCACCATCAGGCTTAGCAAGCTAGCTGCAGTAACGCCATTTTGCAAGGCATGAAAAAGTACCAGAGCTGAG
TTCTCAAAAGTTACAAGAAAGTTCAGTTAAAGATTAACAGTTAAAGATTAAGGCTGAATAATACTGGGACAGGGGCCAAA
TATCGGTGGTCAAGCACCTGGGCCCCGGCTCAGGGCCAAGAACAGATGGCTCTCAGACGTCAGTGTTAGCAGAACTAGCT
TCACTGATTTAGAAAAATAGAGGTGCACAGTGCTCTGGCCACTCCTTGAACCTGTGTGTCTGCCAATGTTCTGACCAGGT
GTGTGCCCATTGTTGAACCTTCATTAGACCCTTTCCTCGTACCCCTCCCATACCCATTTCTTGAAAATAGACATTGTTTA
GAACTAAAAAGTCCCACCTCAGTTTCCCCAAATGACCGAGAAATACCCCAAGCCTTATTCGAACTAACCAACCAGCTCGC
TTCTCGCTTCTGTAACCGCGCTTTTTGCTCCCCAGCCCCAGCCCTATAAAAAGGGTAAAAACTCCACACTCGGCGCGCCA
GTCCTCCGATAGACTGCGTCGCCCGGGTACCCGTGCTCCCAATAAAGCCTCTTGCTGTTTGCATCCGAATCGTGGTCTCG
CTGGTCCTTGAGAGGGTCTCCTCAGATTGATTGACTACCCACGTCGGGGGTCTTTCATTTGGAGGTCCCACCGAGATTTG
GAGACCCCTGCCCAGGGACCACCGACCCCCCCGCCGGGAGGTAAGCTGGCCAGCGGTCGTTTCGTGTCTGTCTCTGTCTT
CGTGCGTGTTTGTGCCGGCATCCAATGTTTGCGCCTGCGTCTGTACTAGTTAGCTAACTAGATCTGTATCTGGCGGTTCC
GCGGAAGAACTGACGAGTTCGTATTCCCGGCCGCAGCCCCTGGGAGACGTCCCAGCGGCCTCGGGGGCCCGTTTTGTGGC
CCATTCTGTATCAGTTAACCTACCCGAGTCGGACTTTTTGGAGCTCCGCCACTGTACGTGGCTTTGTTGGGGGACGAGAG
ACAGAGACACTTCCCGCCCCCGTCTGAATTTTTGCTTTCGGTTTTACGCCGAAGCCGCGCCGCGCGTCTGATTTGTTTGT
TGTTCTTTTGTTCTTCGTTAGTTTTCTTCTGTCTTTAAGTGTTTTCGAGATCATGGGACAGACCGTAACTACCCCTCTGA
GTTTAACCTTGCAGCACTGGGGAGATGTCCAGCGCATTGCATCCAACCAGTCTGTGGATGTCAAGAAGAGGCGCTGGATT
ACCTTCTGTTCCGCTGAATGGCCAACTTTCAATGTGGGATGGCCTCAGGATGGTACTTTCAATTTAAGTATTATCTCTCA
GGTTAAGTCTAGAGTGTTTTGTCCTGGTCCCCACGGACACCCGGATCAGGTCCCATATATCGTCACCTGGGAGGCACTTG
CCTATGACCCCCCTCCGTGGGTCAAACCGTTTGTGTCTCCTAAACTTCCTCCCTTGCCGACAGCTCCCGTCCTCCCGCCC
GGTCCTTCTGCGCAACCTCCGTCCCGATCTGCCCTTTACCCTGCCCTTACCCCCTCTATAAAGTCCAAACCTCCTAAGCC
CCAGGTTCTCCCTGATAGCGGCGGACCCCTCATTGACCTTCTCACAGAGGACCCCCCGCCGTACAGAGCACAACCCTCCT
CCTCTGCCAGGGAGAACGACGAAGAAGAGGCGGCCACCACCTCCGAGGTTTCCCCCCCTTCTCCCATGGTGTCTCGACTG
CGGGGAAGGAGAGACCCTCCCGCAGTGGACTCCACCACCTCCCAGGCATTTCCACTCCGCATGGGGGGAGATGGCCAGCT

TCAGTATTGGCCGTTTTCCTCTTCGGATTTATACAATTGGAAAAATAATAACCCTTCCTTTTCTGAAGATCCAGGTAAAT
TGACGGCCTTGATTGAGTCCGTCCTCATCACCCACCAGCCCACCTGGGACGACTGTCAGCAGTTGTTGGGGACCCTGCTG
ACCGGAGAAGAAAAGCAGCGGGTGCTCCTAGAGGCTAGAAAGGCAGTCCGGGGCAATGATGGACGCCCCACTCAGTTGCC
TAATGAAGTCAATGCTGCTTTTCCCCTTGAACGCCCCGGTTGGGATTACACCACTACAGAAGGTAGGAACCACCTAGTCC
TCTATCGCCAGTTGCTCTTAGCGGGTCTCCAGAACGCGGGCAGAAGTCCCACCAATTTGGCCAAGGTAAAAGGGATAACC
CAGGGACCTAATGAGTCTCCCTCAGCCTTTTTAGAGAGACTCAAAGAGGCCTATCGCAGGTACACTCCTTATGACCCTGA
GGACCCAGGGCAAGAAACCAATGTGTCTATGTCATTCATCTGGCAGTCTGCCCCGGATATCGGGCGAAAGTTAGAGCGGT
TAGAAGATTTAAAGAGCAAGACCTTAGGAGACTTAGTGAGGGAAGCTGAAAAGATCTTTAATAAGCGAGAAACCCCGGAA
GAAAGAGAGGAACGTATCAAGAGAGAAACAGAGGAAAAAGAAGAACGCCGTAGGGCAGAGGATGAGCAGAGAGAGAAAGA
AAGGGACCGCAGAAGACATAGAGAGATGAGCAAGCTCTTGGCCACTGTAGTTATTGGTCAGAGACAGGATAGACAGGGGG
GAGAGCGGAGGAGGCCCCAACTTGATAAGGACCAATGCGCCTACTGCAAAGAAAAGGGACACTGGGCTAAGGACTGCCCA
AAGAAGCCACGAGGGCCCCGAGGACTGAGGCCCCAGACCTCCCTCCTAACCTTAGGTGACTAGGGAGGTCAGGGTCAGGA
GCCCCCCCCTGAACCCAGGATAACCCTCAAAGTCGGGGGGCAACCCGTCACCTTCCTGGTAGATACTGGGGCCCAACACT
CCGTGCTGACCCAAAATCCTGGACCCCTAAGTGACAAGGCTGCCTGGGTCCAAGGGGCTACTGGAGGAAAGCGGTATCGC
TGGACCACGGATCGCAAAGTACATCTAGCTACCGGTAAGGTCACCCACTCTTTCCTCCATGTACCAGACTGCCCCTATCC
TCTGCTAGGAAGAGACTTGCTGACTAAACTAAAAGCCCAAATCCACTTCGAGGGATCAGGAGCTCAGGTTATGGGACCAA
TGGGACTGCCCCTGCAAGTGCTGACCCTAAACATAGAAGATGAGTATCGGCTACATGAGACCTCAAAAGAGCCGGATGTT
TCTCTAGGGTCCACCTGGCTTTCTGATTTTCCCCAGGTCTGGGCGGAAACCGGGGGCATGGGACTGGCAGTTCGCCAAGC
TCCTCTGATCATACCTCTGAAGGCAACCTCTACCCCCGTGTCCATAAAACAATACCCCATGTCACAAGAAGCCAGACTGG
GGATCAAGCCCCACATACAGAGACTGTTGGACCAGGGAATACTGGTACCCTGCCAGTCCCCCTGGAACACGCCCCTGCTA
CCCGTTAAGAAACCAGGGACTAATGATTATAGGCCTGTCCAGGATCTGAGAGAAGTCAACAAGCGGGTGGAAGACATCCA
CCCCACCGTGCCCAACCCTTACAACCTCTTGAGCGGGCTCCCACCGTCCCACCAGTGGTACACTGTGCTTGATTTAAAGG
ATGCCTTTTTCTGCCTGAGACTCCACCCCACCAGTCAGCCTCTCTTCGCCTTTGAGTGGAGAGATCCAGAGATGGGAATC
TCAGGACAATTGACCTGGACCAGACTCCCACAGGGTTTCAAAAACAGTCCCACCCTGTTTGATGAGGCACTGCACAGAGA
CCTAGCAGACTTCCGGATCCAGCACCCAGACTTGATCCTGCTACAGTACGTGGATGACTTACTGCTGGCCGCCACTTCTG
AGCTCGACTGCCAACAAGGTACTCGGGCCCTGTTACAAACCCTAGGGGACCTCGGGTATCGGGCCTCGGCCAAGAAAGCC
CAAATTTGCCAGAAACAGGTCAAGTATCTGGGGTATCTTCTAAAAGAGGGTCAGAGATGGCTGACTGAGGCCAGAAAAGA
GACTGTGATGGGGCAGCCTACTCCGAAGACCCCTCGACAACTAAGGGAGTTCCTAGGGACGGCAGGCTTCTGTCGCCTCT
GGATCCCTGGGTTTGCAGAAATGGCAGCCCCCTTGTACCCTCTCACCAAAACGGGGACTCTGTTTAATTGGGGCCCAGAC
CAGCAAAAGGCCTATCAAGAAATCAAACAGGCTCTTCTAACTGCCCCAGCCCTGGGATTGCCAGATTTGACTAAGCCCTT
TGAACTCTTTGTTGACGAGAAGCAGGGCTACGCCAAAGGCGTCCTAACGCAAAAACTGGGACCTTGGCGTCGGCCGGTGG
CCTACCTGTCCAAAAAGCTAGACCCAGTGGCAGCTGGGTGGCCCCCTTGCCTACGGATGGTAGCAGCCATTGCCGTTCTG
ACAAAAGATGCAGGCAAGCTAACCATGGGACAGCCGCTAGTCATCCTGGCCCCCCATGCAGTAGAGGCACTAGTCAAGCA
ACCCCCTGACCGCTGGCTATCCAACGCCCGCATGACCCACTATCAGGCGATGCTCCTAGATACGGACCGGGTCCAGTTCG
GACCGGTGGTAGCCCTAAACCCGGCTACGTTGCTCCCCCTACCGGGGAAAGAGCCTCACCATGACTGCCTCGAGATCTTG
GCCGAGACACACGGAACCAGACCAGACCTCACGGACCAGCCCCTCCCAGACGCCGACCACACCTGGTATACAGATGGAAG
CAGCTTCCTGCAAGAGGGACAACGTAGGGCTGGAGCAGCGGTGACCACCGAGACCGAGGTAATCTGGGCCAAGGCGTTGC
CAGCCGGGACATCCGCCCAGCGAGCTGAACTAATAGCACTCACCCAGGCCCTAAAGATGGCAGAAGGTAAGAAGCTAAAT
GTTTATACTGATAGCCGCTATGCCTTTGCTACCGCCCATATCCATGGAGAAATATATAGGAGACGTGGGTTGCTCACCTC
AGAAGGCAAGGAGATCAAGAACAAGGGCGAAATCTTGGCCTTACTGAAAGCTCTCTTTCTGCCCAAAAGACTCAGTATAA
TTCACTGCCCAGGACATCAGAAAGGCAATAGTGCTGAAGCTAAAGGCAACCGAATGGCGGACCAGGCAGCCCGGGAAGCG
GCCATGGGGACTGACACAAAGGCCTCCTCACTTCTCATAGAGACCTCAACCCCGTACACTCCAGACTTCTTCCATTATAC
TGAGACAGATATAAAGAACCTACAAGAGTTGGGAGCCACATATGATAGAGAGAAAAAATATTGGGTCCTGCAAGGTAAAC
CTGTGATGCCTGACCAGTTCACCTTTGAATTATTAGACTTCCTTCACCAGCTCACCCACCTTAGCTATCAGAAGATGAGG
GCACTTCTAGACAGGAAAGAAAGCCCCTATTACATGCTAAATAAAGATAAGATCCTCCACGAGGTGGCGGAATCATGCCA
AGCCTGTGTCCAAGTAAATGCCAGTAAGACTAAGATCAGGGCCGGAACACGAGTAAGAGGACATCGACCAGGCACCCATT
GGGAAATTGACTTTACTGAAGTGAAGCCCGGACTGTATGGGTACAAGTATCTCCTGGTATTCGTGGACACGTTCTCTGGC
TGGGTTGAAGCCTTCCCAACCAAACATGAGACTGCCAAAATAGTGACCAAGAAACTTCTGGAAGAAATATTTCCAAGGTT
TGGAATGCCCCAAGTGTTGGGGACTGATAATGGGCCTGCCTTCGTCTCCCAGGTAAGTCAGTCGGTGGCCAAGCTACTGG
GGATTGATTGGAAACTACATTGTGCTTACAGACCCCAGAGTTCAGGTCAGGTAGAAAGAATGAATAGGACAATCAAGGAG
ACTTTGACCAAATTAACGCTTGCAACTGGCACTAGAGACTGGGTACTCCTACTTCCCTTAGCCCTCTACCGAGCCCGCAA
CACTCCGGGCCCCCATGGACTCACTCCGTATGAAATCCTGTATGGGGCGCCCCCGCCCCTTGTTAATTTCCATGATCCTG
AAATGTCAAAGTTTACTAATAGCCCCTCTCTCCAAGCTCACTTACAGGCCCTCCAAGCAGTACAACGAGAGGTCTGGAAG
CCACTGGCCGCTGCCTATCAGGACCAGCTGGACCAGCCAGTGATACCACACCCCTTCCGTGTCGGCGACACCGTGTGGGT
ACGCCGGCACCAGACTAAGAACTTGGAACCTCGCTGGAAAGGACCCTACACCGTCCTGCTGACCACCCCCACCGCTCTCA
AAGTAGACGGCATCGCTGCGTGGATCCACGCCGCTCACGTAAAAGCGGCGACAACCCCTCCGGCCGGAACAGCATCAGGA
CCGACATGGAAGGTCCAGCGTTCTCAAAACCCCTTAAAGATAAGATTAACCCGTGGGGCCCCCTAATAATCCTGGGGATC

TTAATAAGAGCAGGAGTATCAGTACAACATGACAGCCCTCATCAGGTCTTCAATGTTACTTGGAGAGTTACCAACTTAAT
GACAGGACAAACAGCTAATGCTACCTCCCTCCTGGGGACAATGACCGATGCCTTTCCTAAACTGTACTTTGACTTGTGCG
ATTTAATAGGGGACGACTGGGATGAGACTGGACTCGGGTGTCGCACTCCCGGGGGAAGAAAAAGGGCAAGAACATTTGAC
TTCTATGTTTGCCCCGGGCATACTGTACCAACAGGGTGTGGAGGGCCGAGAGAGGGCTACTGTGGCAAATGGGGCTGTGA
GACCACTGGACAGGCATACTGGAAGCCATCATCATCATGGGACCTAATTTCCCTTAAGCGAGGAAACACCCCTCGGAATC
AGGGCCCCTGTTATGATTCCTCAGCGGTCTCCAGTGACATCAAGGGCGCCACACCGGGGGGTCGATGCAATCCCCTAGTC
CTGGAATTCACTGACGCGGGCAAAAAGGCCAGCTGGGATGGCCCCAAAGTATGGGGACTAAGACTGTACCGATCCACAGG
GACCGACCCGGTGACCCGGTTCTCTTTGACCCGCCAGGTCCTCAATATAGGGCCCCGCGTCCCCATTGGGCCTAATCCCG
TGATCACTGACCAGTTACCCCCCTCCCGACCCGTGCAGATCATGCTCCCCAGGCCTCCTCAGCCTCCTCCTCCAGGCGCA
GCCTCTATAGTCCCTGAGACTGCCCCACCTTCTCAACAACCTGGGACGGGAGACAGGCTGCTAAACCTGGTAGATGGAGC
CTACCAAGCTCTCAACCTCACCAGTCCTGACAAAACCCAAGAGTGCTGGTTGTGTCTGGTAGCGGGACCCCCCTACTACG
AAGGGGTTGCCGTCCTAGGTACTTATTCCAACCATACCTCTGCCCCAGCTAACTGCTCCGTGGCCTCCCAACACAAGCTG
ACCCTGTCCGAAGTGACCGGACAGGGACTCTGCGTAGGAGCAGTTCCCAAAACCCATCAGGCCCTGTGTAATACCACCCA
GAAGACGAGCGACGGGTCCTACTATCTGGCTGCTCCCGCCGGGACCATTTGGGCTTGCAACACCGGGCTCACTCCCTGCC
TATCTACCACTGTACTCGACCTCACCACCGATTACTGTGTCCTGGTTGAGCTCTGGCCAAAGGTGACCTACCACTCCCCT
GGTTATGTTTATGGCCAGTTTGAGAAAAAAACCAAATATAAAAGAGAGCCGGTGTCATTAACTCTGGCCCTGCTGTTGGG
AGGACTTACTATGGGCGGCATAGCTGCAGGAGTAGGAACCGGGACTACAGCCCTAGTGGCCACCAAACAATTCGAGCAGC
TCCAGGCAGCCATACATACAGACCTTGGGGCTTTAGAAAAGTCAGTCAGTGCCCTAGAAAAGTCTCTGACCTCGTTGTCT
GAGGTGGTCCTACAGAACCGGAGGGGATTAGATCTACTGTTCCTAAAAGAAGGAGGATTATGTGCTGCCCTAAAAGAAGA
ATGCTGTTTCTACGCGGACCACACTGGCGTAGTAAGAGATAGCATGGCAAAGCTAAGAGAAAGGTTGAACCAGAGACAAA
AATTGTTCGAATCAGGACAAGGGTGGTTTGAGGGACTGTTTAACAGGTCCCCATGGTTCACGACCTTAATATCTACCATT
ATGGGCCCCTTGATAATACTTTTATTAATCCTACTCTTCGGACCCTGTATTCTCAACCGCTTGGTCCAGTTTGTAAAAGA
CAGAATTTCGGTAGTGCAGGCCCTGGTTTTGACCCAACAGTATCACCAACTCAAATCAATAGATCCAGAAGAAGTGGAAT
CACGTGAATAAAAGATTTTATTCAGTTTCCAGAAAGAGGGGGGAATGAAAGACCCCACCATCAGGCTTAGCAAGCTAGCT
GCAGTAACGCCATTTTGCAAGGCATGAAAAAGTACCAGAGCTGAGTTCTCAAAAGTTACAAGAAAGTTCAGTTAAAGATT
AACAGTTAAAGATTAAGGCTGAATAATACTGGGACAGGGGCCAAATATCGGTGGTCAAGCACCTGGGCCCCGGCTCAGGG
CCAAGAACAGATGGCTCTCAGACGTCAGTGTTAGCAGAACTAGCTTCACTGATTTAGAAAAATAGAGGTGCACAGTGCTC
TGGCCACTCCTTGAACCTGTGTGTCTGCCAATGTTCTGACCAGGTGTGTGCCCATTGTTGAACCTTCATTAGACCCTTTC
CTCGTACCCCTCCCATACCCATTTCTTGAAAATAGACATTGTTTAGAACTAAAAAGTCCCACCTCAGTTTCCCCAAATGA
CCGAGAAATACCCCAAGCCTTATTCGAACTAACCAACCAGCTCGCTTCTCGCTTCTGTAACCGCGCTTTTTGCTCCCCAG
CCCCAGCCCTATAAAAAGGGTAAAAACTCCACACTCGGCGCGCCAGTCCTCCGATAGACTGCGTCGCCCGGGTACCCGTG
CTCCCAATAAAGCCTCTTGCTGTTTGCATCCGAATCGTGGTCTCGCTGGTCCTTGAGAGGGTCTCCTCAGATTGATTGAC
TACCCACGTCGGGGGTCTTTCA
>Pmv12
TGAAAGACCCCACCATCAGGCTTAGCAAGCTAGCTGCAGTAACGCCATTTTGCAAGGCATGAAAAAGTACCAGAGCTGAG
TTCTCAAAAGTTACAAGAAAGTTCAGTTAAAGATTAACAGTTAAAGATTAAGGCTGAATAATACTGGGACAGGGGCCAAA
TATCGGTGGTCAAGCACCTGGGCCCCGGCTCAGGGCCAAGAACAGATGGCTCTCAGACGTCAGTGTTAGCAGAACTAGCT
TCACTGATTTAGAAAAATAGAGGTGCACAGTGCTCTGGCCACTCCTTAAACCTGTGTGTCTGCCAATGTTCTGACCAGGT
GTGTGCCCATTGTTGAACCTTCATTAGACCCTTTCCTCGTACCCCTCCCATACCCATTTCTTGAAAATAGACATTGTTTA
GAACTAAAAAGTCCCACCTCAGTTTCCCCAAATGACCGAGAAATACCCCAAGCCTTATTCGAACTAACCAACCAGCTCGC
TTCTCGCTTCTGTAACCGCGCTTTTTGCTCCCCAGCCCCAGCCCTATAAAAAGGGTAAAAACTCCACACTCGGCGCGCCA
GTCCTCCGATAGACTGCGTCGCCCGGGTACCCGTGTTCCCAATAAAGCCTCTTGCTGTTTGCATCCGAATCGTGGTCTCG
CTGGTCCTTGAGAGGGTCTCCTCAGATTGATTGACTACCCACGTCGGGGGTCTTTCATTTGGAGGTTCCACCGAGATTTG
GAGACCCCTGCCCAGGGACCACCGACCCCCCGCCGGGAGGTAAGCTGGCCAGCGGTCGTTTCGTGTCTGTCTCTGTCTTC
GTGCGTGTTTGTGCCGGCATCCAATGTTTGCGCCTGCGTCTGTACTAGTTAGCTAACTAGATCTGTATCTGGCGGTTCCG
CGGAAGAACTGACGAGTTCGTATTCCCGGCCGCAGCCCCTGGGAGACGTCCCAGCGGCCTCGGGGGCCCGTTTTGTGGCC
CATTCTGTATCAGTTAACCTACCCGAGTCGGACTTTTTGGAGCTCCGCCACTGTACGTGGCTTTGTTGGGGGACGAGAGA
CAGAGACACTTCCCGCCCCCGTCTGAATTTTTGCTTTCGGTTTTACGCCGAAGCCGCGCCGCGCGTCTGATTTGTTTGTT
GTTCTTTTGTTCTTCGTTAGTTTTCTTCTGTCTTTAAGTGTTTTCGAGATCATGGGACAGACCGTAACTACCCCTCTGAG
TTTAACCTTGCAGCACTGGGGAGATGTCCAGCGCATTGCATCCAACCAGTCTGTGGATGTCAGGAAGAGGCGCTAGATTA
CCTTCTGTTCCGCCGAATGGCCAACTTTCAATGTGGGATGGCCTCAGGATGGTACTTTCAATTTAAGTATTATCTCTCAG
GTTAAGTCTAGAGTGTTTTGTCCTGGTCCCCACGGACACCCGGATCAGGTCCCATATATCGTCACCTGGGAGGCACTTGC
CTATGACCCCCCTCCGTGGGTCAAACCGTTTGTGTCTCCTAAACTTCCTCCCTTGCCGACAGCTCCCGTCCTCCCGCCCG
GTCCTTCTGCGCAACCTCCGTCCCGATCTGCCCTTTACCCTGCCCTTACCCCCTCTATAAAGTCCAAACCTCCTAAGCCC
CAGGTTCTCCCTGATAGCGGCGGACCCCTCATTGACCTTCTCACAGAGGACCCCCCGCCGTACAGAGCACAACCCTCCTC
CTCTGCCAGGGAGAACGACGAAGAAGAGGCGGCCACCACCTCCGAGGTTTCCCCCCCTTCTCCCATGGTGTCTCGACTGC
GGGGAAGGAGAGACCCTCCCGCAGTGGACTCCACCACCTCCCAGGCATTTCCACTCCGCATGGGGGGAGATGGCCAGCTT

CAGTATTGGCCGTTTTCCTCTTCGGATTTATACAATTGGAAAAATAATAACCCTTCCTTTTCTGAAGATCCAGGTAAATT
GACGGCCTTGATTGAGTCCGTCCTCATCACCCACCAGCCCACCTGGGACGACTGTCAGCAGTTGTTGGGGACCCTGCTGA
CCGGAGAAGAAAAGCAGCGGGTGCTCCTAGAGGCTAGAAAGGCAGTCCGGGGCAATGATGGACGCCCCACTCAGTTGCCT
AATGAAGTCAATGCTGCTTTTCCCCTTGAACGCCCCGGTTGGGATTACACCACTACAGAAGGTAGGAACCACCTAGTCCT
CTATCGCCAGTTGCTCTTAGCGGGTCTCCAGAACGCGGGCAGAAGTCCCACCAATTTGGCCAAGGTAAAAGGGATAACCC
AGGGACCTAATGAGTCTCCCTCAGCCTTTTTAGAGAGACTCAAAGAGGCCTATCGCAGGTACACTCCTTATGACCCTGAG
GACCCAGGGCAAGAAACCAATGTGTCTATGTCATTCATCTGGCAGTCTGCCCCGGATATCGGGCGAAAGTTAGAGCGGTT
AGAAGATTTAAAGAGCAAGACCTTAGGAGACTTAGTGAGGGAAGCTGAAAAGATCTTTAATAAGCGAGAAACCCCGGAAG
AAAGAGAGGAACGTATCAAGAGAGAAACAGAGGAAAAAGAAGAACGCCGTAGGGCAGAGGATGAGCAGAGAGAGAAAGAA
AGGGACCGCAGAAGACATAGAGAGATGAGCAAGCTCTTGGCCACTGTAGTTATTGGTCAGAGACAGGATAGACAGGGGGG
AGAGCGGAGGAGGCCCCAACTTGATAAGGACCAATGCGCCTACTGCAAAGAAAAGGGACACTGGGCTAAGGACTGCCCAA
AGAAGCCACGAGGGCCCCGAGGACTGAGGCCCCAGACCTCCCTCCTAACCTTAGGTGACTAGGGAGGTCAGGGTCAGGAG
CCCCCCCCTGAACCCAGGATAACCCTCAAAGTCGGGGGGCAACCCGTCACCTTCCTGGTAGATACTGGGGCCCAACACTC
CGTGCTGACCCAAAATCCTGGACCCCTAAGTGACAAGGCTGCCTGGGTCCAAGGGGCTACTGGAGGAAAGCGGTATCGCT
GGACCACGGATCGCAAAGTACATCTAGCTACCGGTAAGGTCACCCACTCTTTCCTCCATGTACCAGACTGCCCCTATCCT
CTGCTAGGAAGAGACTTGCTGACTAAACTAAAAGCCCAAATCCACTTCGAGGGATCAGGAGCCCAGGTTATGGGACCAAT
GGGACTGCCCCTGCAAGTGCTGACCCTAAACATAGAAGATGAGTATCGGCTACATGAGACCTCAAAAGAGCCGGATGTTT
CTCTAGGGTCCACCTGGCTTTCTGATTTTCCCCAGGTCTGGGCGGAAACCGGGGGCATGGGACTGGCAGTTCGCCAAGCT
CCTCTGATCATACCTCTGAAGGCAACCTCTACCCCCGTGTCCATAAAACAATACCCCATGTCACAAGAAGCCAGACTGGG
GATCAAGCCCCACATACAGAGACTGTTGGACCAGGGAATACTGGTACCCTGCCAGTCCCCCTGGAACACGCCCCTGCTAC
CCGTTAAGAAACCAGGGACTAATGATTATAGGCCTGTCCAGGATCTGAGAGAAGTCAACAAGCGGGTGGAAGACATCCAC
CCCACCGTGCCCAACCCTTACAACCTCTTGAGCGGGCTCCCACCGTCCCACCAGTGGTACACTGTGCTTGATTTAAAGGA
TGCCTTTTTCTGCCTGAGACTCCACCCCACCAGTCAGCCTCTCTTCGCCTTTGAGTGGAGAGATCCAGAGATGGGAATCT
CAGGACAATTGACCTGGACCAGACTCCCACAGGGTTTCAAAAACAGTCCCACCCTGTTTGATGAGGCACTGCACAGAGAC
CTAGCAGACTTCCGGATCCAGCACCCAGACTTGATCCTGCTACAGTACGTGGATGACTTACTGCTGGCCGCCACTTCTGA
GCTCGACTGCCAACAAGGTACTCGGGCCCTGTTACAAACCCTAGGGAACCTCGGGTATCGGGCCTCGGCCAAGAAAGCCC
AAATTTGCCAGAAACAGGTCAAGTATCTGGGGTATCTTCTAAAAGAGGGTCAGAGATGGCTGACTGAGGCCAGAAAAGAG
ACTGTGATGGGACAGCCTACTCCGAAGACCCCTCGACAACTAAGAGAGTTCCTAGGGACGGCAGGCTTCTGTCGCCTCTG
GATCCCTGGGTTTGCAGAAATGGCAGCCCCCTTGTACCCTCTCACCAAAACGGGGACTCTGTTTAATTGGGGCCCAGACC
AGCAAAAGGCCTATCAAGAAATCAAACAGGCTCTTCTAACTGCCCCAGCCCTGGGATTGCCAGATTTGACTAAGCCCTTT
GAACTCTTTGTTGACGAGAAGCAGGGCTACGCCAAAGGCGTCCTAACGCAAAAACTGGGACCTTGGCGTCGGCCGGTGGC
CTACCTGTCCAAAAAGCTAGACCCAGTGGCAGCTGGGTGGCCCCCTTGCCTACGGATGGTAGCAGCCATTGCCGTTCTGA
CAAAAGATGCAGGCAAGCTAACCATGGGACAGCCGCTAGTCATCCTGGCCCCCCATGCAGTAGAGGCACTAGTCAAGCAA
CCCCCTGACCGCTGGCTATCCAACGCCCGCATGACCCACTATCAGGCGATGCTCCTAGATACGGACCGGGTCCAGTTCGG
ACCGGTGGTAGCCCTAAACCCGGCTACGTTGCTCCCCCTACCGGGGAAAGAGCCTCACCATGACTGCCTCGAGATCTTGG
CCGAGACACACGGAACCAGACCAGACCTCACGGACCAGCCCCTCCCAGACGCCGACCATACCTGGTATACAGATGGAAGC
AGCTTCCTGCAAGAGGGACAACGTAGGGCTGGAGCAGCGGTGACCACCGAGACCGAGGTAATCTGGGCCAAGGCGTTGCC
AGCCGGGACATCCGCCCAGCGAGCTGAACTAATAGCACTCACCCAGGCCCTAAAGATGGCAGAAGGTAAGAAGCTAAATG
TTTATACTGATAGCCGCTATGCCTTTGCTACCGCCCATGTCCATGGAGAAATATATAGGAGACGTGGGTTGCTCACCTCA
GAAGGCAAGGAGATCAAGAACAAGGGCGAAATCTTGGCCTTACTGAAAGCTCTCTTTCTGCCCAAAAGACTCAGTATAAT
TCACTGCCCAGGACATCAGAAAGGCAATAGTGCTGAAGCTAAAGGCAACCGAATGGCGGACCAGGCAGCCCGGGAAGCAG
CCATGGGGACTGACACAAAGGCCTCCTCACTTCTCATAGAGACCTCAACCCCGTACACTCCAGACTTCTTCCATTATACT
GAGACAGATATAAAGAACCTACAAGAGTTGGGAGCCACATATGATAGAGAAAAAAAATATTGGGTCCTGCAAGGTAAACC
TGTGATGCCTGACCAGTTCACCTTTGAATTATTAGACTTCCTTCACCAGCTCACCCACCTTAGCTATCAGAAGATGAGGG
CACTTCTAGACAGGAAAGAAAGCCCCTATTACATGCTAAATAAAGATAAGATCCTCCACGAGGTGGCGGAATCATGCCAA
GCCTGTGTCCAAGTAAATGCCAGTAAGACTAAGATCAGGGCCGGAACACGAGTAAGAGGACATCGACCAGGCACCCATTG
GGAAATTGACTTTACTGAAGTGAAGCCCGGACTGTATGGGTACAAGTATCTCCTGGTATTCGTGGACACGTTCTCTGGCT
GGGTTGAAGCCTTCCCAACCAAACATGAGACTGCCAAAATAGTGACCAAGAAACTTCTGGAAGAAATATTTCCAAGGTTT
GGAATGCCCCAAGTGTTGGGGACTGATAATGGGCCTGCCTTCGTCTCCCAGGTAAGTCAGTCGGTGGCCAAGCTACTGGG
GATTGATTGGAAACTACATTGTGCTTACAGACCCCAGAGTTCAGGTCAGGTAGAAAGAATGAATAGGACAATCAAGGAGA
CTTTGACCAAATTAACGCTTGCAACTGGCACTAGAGACTGGGTACTCCTACTTCCCTTAGCCCTCTACCGAGCCCGCAAC
ACTCCGGGCCCCCATGGACTCACTCCGTATGAAATCCTGTATGGGGCGCCCCCGCCCCTTGTTAATTTCCATGATCCTGA
AATGTCAAAGTTTACTAATAGCCCCTCTCTCCAAGCTCACTTACAGGCCCTCCAAGCAGTACAACGAGAGGTCTGGAAGC
CACTGGCCGCTGCCTATCAGGACCAGCTGGACCAGCCAGTGATACCACACCCCTTCCGTGTCGGCGACACCGTGTGGGTA
CGCCGGCACCAGACTAAGAACTTGGAACCTCGCTGGAAAGGACCCTACACCGTCCTGCTGACCACCCCCACCGCTCTCAA
AGTAGACGGCATCGCTGCGTGGATCCACGCCGCTCACGTAAAAGCGGCGACAACCCCTCCGGCCGGAACAGCATCAGGAC
CGACATGGAAGGTCCAGCGCTCTCAAAACCCCTTAAAGATAAGATTAACCCGTGGGGCCCCCTAATAGTCCTGGGGATCT

TAATAAGGGCAGGAGTATCAGTACAACATGACAGCCCTCATCAGGTCTTCAATGTTACTTGGAGAGTTACCAACTTAATG
ACAGGACAAACAGCTAATGCTACCTCCCTCCTGGGGACAATGACCGATGCCTTTCCTAAACTGTACTTTGACTTGTGCGA
TTTAATAGGGGACGACTGGGATGAGACTGGACTCGGGTGTCGCACTCCCGGGGGAAGAAAAAGGGCAAGAACATTTGACT
TCTATGTTTGCCCCGGGCATACTGTACCAACAGGGTGTGGAGGGCCGAGAGAGGGCTACTGTGGCAAATGGGGCTGTGAG
ACCACTGGACAGGCATACTGGAAGCCATCATCATCATGGGACCTAATTTCCCTTAAGCGAGGAAACACCCCTCGGAATCA
GGGCCCCTGTTATGATTCCTCAGCGGTCTCCAGTGACATCAAGGGCGCCACACCGGGGGGTCGATGCAATCCCCTAGTCC
TGGAATTCACTGACGCGGGCAAAAAGGCCAGCTGGGATGGCCCCAAAGTATGGGGACTAAGACTGTACCGATCCACAGGG
ACCGACCCGGTGACCCGGTTCTCTTTGACCCGCCAGGTCCTCAATATAGGGCCCCGCGTCCCCATTGGGCCTAATCCCGT
GATCACTGACCAGTTACCCCCCTCCCGACCCGTGCAGATCATGCTCCCCAGGCCTCCTCAGCCTCCTCCTCCAGGCGCAG
CCTCTATAGTCCCTGAGACTGCCCCACCTTCTCAACAACCTGGGACGGGAGACAGGCTGCTAAACCTGGTAGATGGAGCC
TACCAAGCTCTCAACCTCACCAGTCCTGACAAAACCCAAGAGTGCTGGTTGTGTCTGGTAGCGGGACCCCCCTACTACGA
AGGGGTTGCCGTCCTAGGTACTTATTCCAACCATACCTCTGCCCCAGCTAACTGCTCCGTGGCCTCCCAACACAAGCTGA
CCCTGTCCGAAGTGACCGGACAGGGACTCTGCGTAGGAGCAGTTCCCAAAACCCATCAGGCCCTGTGTAATACCACCCAG
AAGACGAGCGACGGGTCCTACTATCTGGCTGCTCCCGCCGGGACCATTTGGGCTTGCAACACCGGGCTCACTCCCTGCCT
ATCTACCACTGTACTCGACCTCACCACCGATTACTGTGTCCTGGTTGAGCTCTGGCCAAAGGTGACCTACCACTCCCCTG
GTTATGTTTATGGCCAGTTTGAGAAAAAAACCAAATATAAAAGAGAGCCGGTGTCATTAACTCTGGCCCTGCTGTTGGGA
GGACTTACTATGGGCGGCATAGCTGCAGGAGTAGGAACCGGGACTACAGCCCTAGTGGCCACCAAACAATTCGAGCAGCT
CCAGGCAGCCATACATACAGACCTTGGGGCTTTAGAAAAGTCAGTCAGTGCCCTAGAAAAGTCTCTGACCTCGTTGTCTG
AGGTGGTCCTACAGAATCGGAGGGGATTAGATCTACTGTTCCTAAAAGAAGGAGGATTATGTGCTGCCCTAAAAGAAGAA
TGCTGTTTCTACGCGGACCACACTGGCGTAGTAAGAGATAGCATGGCAAAGCTAAGAGAAAGGTTGAACCAGAGACAAAA
ATTGTTCGAATCAGGACAAGGGTGGTTTGAGGGACTGTTTAACAGGTCCCCATGGTTCACGACCTTAATATCCACCATTA
TGGGCCCCTTAATAATACTTTTATTAATCCTACTCTTCGGACCCTGTATTCTCAACCGCTTGGTCCAGTTTGTAAAAGAC
AGAATTTCGGTAGTGCAGGCCCTGGTTTTGACCCAACAGTATCACCAACTCAAATCAATAGATCCAGAAGAAGTGGAATC
ACGTGAATAAAAGATTTTATTCAGTTTCCAGAAAGAGGGGGGAATGAAAGACCCCACCATCAGGCTTAGCAAGCTAGCTG
CAGTAACGCCATTTTGCAAGGCATGAAAAAGTACCAGAGCTGAGTTCTCAAAAGTTACAAGAAAGTTCAGTTAAAGATTA
ACAGTTAAAGATTAAGGCTGAATAATACTGGGACAGGGGCCAAATATCGGTGGTCAAGCACCTGGGCCCCGGCTCAGGGC
CAAGAACAGATGGCTCTCAGACGTCAGTGTTAGCAGAACTAGCTTCACTGATTTAGAAAAATAGAGGTGCACAGTGCTCT
GGCCACTCCTTAAACCTGTGTGTCTGCCAATGTTCTGACCAGGTGTGTGCCCATTGTTGAACCTTCATTAGACCCTTTCC
TCGTACCCCTCCCATACCCATTTCTTGAAAATAGACATTGTTTAGAACTAAAAAGTCCCACCTCAGTTTCCCCAAATGAC
CGAGAAATACCCCAAGCCTTATTCGAACTAACCAACCAGCTCGCTTCTCGCTTCTGTAACCGCGCTTTTTGCTCCCCAGC
CCCAGCCCTATAAAAAGGGTAAAAACTCCACACTCGGCGCGCCAGTCCTCCGATAGACTGCGTCGCCCGGGTACCCGTGT
TCCCAATAAAGCCTCTTGCTGTTTGCATCCGAATCGTGGTCTCGCTGGTCCTTGAGAGGGTCTCCTCAGATTGATTGACT
ACCCACGTCGGGGGTCTTTCA
>Pmv13
TGAAAGACCCCACCATCAGGCTTAGCAAGCTAGCTGCAGTAACGCCATTTTGCAAGGCATGAAAAAGTACCAGAGCTAAG
TTCTCAAAAGTTACAAGAAAGTTCAGTTAAAGATTAACAGTTAAAGATTAAGGCTGAATAATACCGGGACAGGGGCCAAA
TATCGGTGGTCAAGCACCTGGGCCCCGGCTCAGGGCCAAGAACAGATGGCTCTCAGACGTCAGTGTTAGCAGAACTAGCT
TCACTGATTTAGAAAAATAGAGGTGCACAGAGCTCTGGCCACTCCTTGAACCTGTGTGTCTGCCAATGTTCTGACCAGGT
GTGTGCCCATTGTTGAACCTTCATTAGACCCTCTCCTCGTACCCCTCCCATACCCATTTCTTGAAAATAGACATTGTTTA
GAACTAAAAAGTCCCACCTCAGTTTCCCCAAATGACCGAGAAATACCCCAAGCCTTATTCGAACTAACCAACCAGCTCGC
TTCTCGCTTCTGTAACCGCGCTTTTTGCTCCCCAGCCCCAGCCCTATAAAAAGGGTAAAAACTCCACACTCGGCGCGCCA
GTCCTCCGATAGACTGCGTCGCCCGGGTACCCGTGTTCCCAATAAAGCCTCTTGCTGTTTGCATCCGAATCGTGGTCTCG
CTGGTCCTTGAGAGGGTCTCCTCAGATTGATTGACTACCCACGTCGGGGGTCTTTCATTTGGAGGTCCCACCGAGATTTG
GAGACCCCTGCCCAGGGACCACCGACCCCCCGCCGGGAGGTAAGCTGGCCAGCGGTCGTTTCGTGTCTGTCTCTGTCTTC
GTGCGTGTTTGTGCCGGCATCCAATGTTTGCGCCTGCGTCTGTACTAGTTAGCTAACTAGATCTGTATCTGGCGGTTCCG
CGGAAGAACTGACGAGTTCGTATTCCCGGCCGCAGCCCCTGGGAGACGTCCCAGCGGCCTCGGGGGCCCGTTTTGTGGCC
CATTCTGTATCAGTTAACCTACCCGAGTCGGACTTTTTGGAGCTCCGCCACTGTACGTGGCTTTGTTGGGGGACGAGAAA
CAGAGACACTTCCCGCCCCCGTCTGAATTTTTGCTTTCGGTTTTACGCCGAAGCCGCGCCGCGCGTCTGATTTGTTTGTT
GTTCTTTTGTTCTTCGTTAGTTTTCTTCTGTCTTTAAGTGTTTTCGAGATCATGGGACAGACCGTAACTACCCCTCTGAG
TTTAACCTTGCAGCACTGGGGAGATGTCCAGCGCATTGCATCCAACCAGTCTGTGGATGTCAGGAAGAGGCGCTGGATTA
CCTTCTGTTCCGCTGAATGGCCAACTTTCAATGTGGGATGGCCTCAGGATGGTACTTTCAATTTAAGTATTATCTCTCAG
GTTAAGTCTAGAGTGTTTTGTCCTGGTCCCCACGGACACCCGGATCAGGTCCCATATATCGTCACCTGGGAGGCACTTGC
CTATGACCCCCCTCCGTGGGTCAAACCGTTTGTGTCTCCTAAACTTCCTCCCTTGCCGACAGCTCCCGTCCTCCCGCCCG
GTCCTTCTGCGCAACCTCCGTCCCGATCTGCCCTTTACCCTGCCCTTACCCCCTCTATAAAGTCCAAACCTCCTAAGCCC
CAGGTTCTCCCTGATAGCGGCGGACCCCTCATTGACCTTCTCACAGAGGACCCCCCGCCGTACAGAGCACAACCCTCCTC
CTCTGCCAGGGAGAACGACGAAGAAGAGGCGGCCACCACCTCCGAGGTTTCCCCCCCTTCTCCCATGGTGTCTCGACTGC
GGGGAAGGAGAGACCCTCCCGCAGTGGACTCCACCACCTCCCAGGCATTTCCACTCCGCATGGGGGGAGATGGCCAGCTT

CAGTATTGGCCGTTTTCCTCTTCGGATTTATACAATTGGAAAAATAATAACCCTTCCTTTTCTGAAGATCCAGGTAAATT
GACGGCCTTGATTGAGTCCGTCCTCATCACCCACCAGCCCACCTGGGACGACTGTCAGCAGTTGTTGGGGACCCTGCTGA
CCGGAGAAGAAAAGCAGCGGGTGCTCCTAGAGGCTAGAAAGGCAGTCCGGGGCGATGATGGACGCCCCACTCAGTTGCCT
AATGAAGTCAATGCTGCTTTTCCCCTTGAACGCCCCGGTTGGGATTACACCACTACAGAAGGTAGGAACCACCTAGTCCT
CTATCGCCAGTTGCTCTTAGCGGGTCTCCAGAACGCGGGCAGAAGTCCCACCAATTTGGCCAAGGTAAAAGGGATAACCC
AGGGACCTAATGAGTCTCCCTCAGCCTTTTTAGAGAGACTCAAAGAGGCCTATCGCAGGTACACTCCTTATGACCCTGAG
GACCCAGGGCAAGAAACCAATGTGTCTATGTCATTCATCTGGCAGTCTGCCCCGGATATCGGGCGAAAGTTAGAGCGGTT
AGAAGATTTAAAGAGCAAGACCTTAGGAGACTTAGTGAGGGAAGCTGAAAAGATCTTTAATAAGCGAGAAACCCCGGAAG
AAAGAGAGGAACGTATCAAGAGAGAAACAGAGGAAAAAGAAGAACGCCGTAGGGCAGAGGATGAGCAGAGAGAGAAAGAA
AGGGACCGCAGAAGACATAGAGAGATGAGCAAGCTCTTGGCCACTGTAGTTATTGGTCAGAGACAGGATAGACAGGGGGG
AGAGCGGAGGAGGCCCCAACTTGATAAGGACCAATGCGCCTACTGCAAAGAAAAGGGACACTGGGCTAAGGACTGCCCAA
AGAAGCCACGAGGGCCCCGAGGACTGAGGCCCCAGACCTCCCTCCTAACCTTAGGTGACTAGGGAGGTCAGGGTCAGGAG
CCCCCCCCTGAACCCAGGATAACCCTCAAAGTCGGGGGGCAACCCGTCACCTTCCTGGTAGATACTGGGGCCCAACACTC
CGTGCTGACCCAAAATCCTGGACCCCTAAGTGATAAGGCTGCCTGGGTCCAAGGGGCTACTGGAGGAAAGCGGTATCGCT
GGACCACGGATCGCAAAGTACATCTAGCTACCGGTAAGGTCACCCACTCTTTCCTCCATGTACCAGACTGCCCCTATCCT
CTGCTAGGAAGAGACTTGCTGACTAAACTAAAAGCTCAAATCCACTTCGAGGGATCAGGAGCTCAGGTTATGGGACCAAT
GGGACTGCCCCTGCAAGTGCTGACCCTAAACATAGAAGATGAGTATCGGCTACATGAGACCTCAAAAGAGCCGGATGTTT
CTCTAGGGTCCACCTGGCTTTCTGATTTTCCCCAGGTCTGGGCGGAAACCGGGGGCATGGGACTGGCAGTTCGCCAAGCT
CCTCTGATCATACCTCTGAAGGCAACCTCTACCCCCGTGTCCATAAAACAATACCCCATGTCACAAGAAGCCAGACTGGG
GATCAAGCCCCACATACAGAGACTGTTGGACCAGGGAATACTGGTACCCTGCCAGTCCCCCTGGAACACGCCCCTGCTAC
CCGTTAAGAAACCAGGGACTAATGATTATAGGCCTGTCCAGGATCTGAGAGAAGTCAACAAGCGGGTGGAAGACATCCAC
CCCACCGTGCCCAACCCTTACAACCTCTTGAGCGGGCTCCCACCGTCCCACCAGTGGTACACTGTGCTTGATTTAAAGGA
TGCCTTTTTCTGCCTGAGACTCCACCCCACCAGTCAGCCTCTCTTCGCCTTTGAGTGGAGAGATCCAGAGATGGGAATCT
CAGGACAATTGACCTGGACCAGACTCCCACAGGGTTTCAAAAACAGTCCCACCCTGTTTGATGAGGCACTGCACAGAGAC
CTAGCAGACTTCCGGATCCAGCACCCAGACTTGATCCTGCTACAGTACGTGGATGACTTACTGCTGGCCGCCACTTCTGA
GCTAGACTGCCAACAAGGTACTCGGGCCCTGTTACAAACCCTAGGGAACCTCGGGTATCGGGCCTCGGCCAAGAAAGCCC
AAATTTGCCAGAAACAGGTCAAGTATCTGGGGTATCTTCTAAAAGAGGGTCAGAGATGGCTGACTGAGGCCAGAAAAGAG
ACTGTGATGGGGCAGCCTACTCCGAAGACCCCTCGACAACTAAGGGAGTTCCTAGGGACGGCAGGCTTCTGTCGCCTCTG
GATCCCTGGGTTTGCAGAAATGGCAGCCCCCTTGTACCCTCTCACCAAAACGGGGACTCTGTTTAATTGGGGCCCAGACC
AGCAAAAGGCCTATCAAGAAATCAAACAGGCTCTTCTAACTGCCCCAGCCCTGGGATTGCCAGATTTGACTAAGCCCTTT
GAACTCTTTGTCGACGAGAAGCAGGGCTACGCCAAAGGCGTCCTAACGCAAAAACTGGGACCTTGGCGTCGGCCGGTGGC
CTACCTGTCCAAAAAGCTAGACCCAGTGGCAGCTGGGTGGCCCCCTTGCCTACGGATGGTAGCAGCCATTGCCGTTCTGA
CAAAAGATGCAGGCAAGCTAACCATGGGACAGCCGCTAGTCATCCTGGCCCCCCATGCAGTAGAGGCACTAGTCAAGCAA
CCCCCTGACCGCTGGCTATCCAACGCCCGCATGACCCACTATCAGGCGATGCTCCTAGATACGGACCGGGTCCAGTTCGG
ACCGGTGGTAGCCCTAAACCCGGCTACGTTGCTCCCCCTACCGGGGAAAGAGCCTCACCATGACTGCCTCGAGATCTTGG
CCGAGACACACGGAACCAGACCAGACCTCACGGACCAGCCCCTCCCAGACGCCGACCACACCTGGTATACAGATGGAAGC
AGCTTCCTGCAAGAGGGACAACGTAGGGCTGGAGCAGCGGTGACCACCGAGACCGAGGTAATCTGGGCCAAGGCGTTGCC
AGCCGGGACATCCGCCCAGCGAGCTGAACTAATAGCACTCACCCAGGCCCTAAAGATGGCAGAAGGTAAGAAGCTAAATG
TTTATACTGATAGCCGCTATGCCTTTGCTACCGCCCATGTCCATGGAGAAATATATAGGAGACGTGGGTTGCTCACCTCA
GAAGGCAAGGAGATCAAGAACAAGGGCGAAATCTTGGCCTTACTGAAAGCTCTCTTTCTGCCCAAAAGACTCAGTATAAT
TCACTGCCCAGGACATCAGAAAGGCAATAGTGCTGAAGCTAAAGGCAACCGAATGGCGGACCAGGCAGCCCGGGAAGCAG
CCATGGGGACTGACACAAAGGCCTCCTCACTTCTCATAGAGACCTCAACCCCGTACACTCCAGACTTCTTCCATTATACT
GAGACAGATATAAAGAACCTACAAGAGTTGGGAGCCACATATGATAGAGAGAAAAAATATTGGGTCCTGCAAGGTAAACC
TGTGATGCCTGACCAGTTCACCTTTGAATTATTAGACTTCCTTCACCAGCTCACCCACCTTAGCTATCAGAAGATGAGGG
CACTTCTAGACAGGAAAGAAAGCCCCTATTACATGCTAAATAAAGATAAGATCCTCCACGAGGTGGCGGAATCATGCCAA
GCCTGTGTCCAAGTAAATGCCAGTAAGACTAAGATCAGGGCCGGAACACGAGTAAGAGGACATCGACCAGGCACCCATTG
GGAAATTGACTTTACTGAAGTGAAGCCCGGACTGTATGGGTACAAGTATCTCCTGGTATTCGTGGACACGTTCTCTGGCT
GGGTTGAAGCCTTCCCAACCAAACATGAAACTGCCAAAATAGTGACCAAGAAACTTCTGGAAGAAATATTTCCAAGGTTT
GGAATGCCCCAAGTGTTGGGGACTGATAATGGGCCTGCCTTCGTCTCCCAGGTAAGTCAGTCGGTGGCCAAGCTACTGGG
GATTGATTGGAAACTACATTGTGCTTACAGACCCCAGAGTTCAGGTCAGGTAGAAAGAATGAATAGGACAATCAAGGAGA
CTTTGACCAAATTAACGCTTGCAACTGGCACTAGAGACTGGGTACTCCTACTTCCCTTAGCCCTCTACCGAGCCCGCAAC
ACTCCGGGCCCCCATGGACTCACTCCGTATGAAATCCTGTATGGGGCGCCCCCGCCCCTTGTTAATTTCCATGATCCTGA
AATGTCAAAGTTTACTAATAGCCCCTCTCTCCAAGCTCACTTACAGGCCCTCCAAGCAGTACAACGAGAGGTCTGGAAGC
CACTGGCCGCTGCCTATCAGGACCAGCTGGACCAGCCAGTGATACCACACCCCTTCCGTGTCGGCGACACCGTGTGGGTA
CGCCGGCACCAGACTAAGAACTTGGAACCTCGCTGGAAAGGACCCTACACCGTCCTGCTGACCACCCCCACCGCTCTCAA
AGTAGACGGCATCGCTGCGTGGATCCACGCCGCTCACGTAAAAGCGGCGACAACCCCTCCGGCCGGAACAGCATCAGGAC
CGACATGGAAGGTCCAGCGTTCTCAAAACCCCTTAAAGATAAGATTAACCCGTGGGGCCCCCTAATAGTCCTGGGAATCT

TAATAAGGGCAGGAGTATCAGTACAACATGACAGCCCTCATCAGGTCTTCAATGTTACTTGGAGAGTTACCAACTTAATG
ACAGGACAAACAGCTAATGCTACCTCCCTCCTGGGGACAATGACCGATGCCTTTCCTAAACTGTACTTTGACTTGTGCGA
TTTAATAGGGGACGACTGGGATGAGACTGGACTCGGGTGTCGCACTCCCGGGGGAAGAAAAAGGGCAAGAACATTTGACT
TCTATGTTTGCCCCGGGCATACTGTACCAACAGGGTGTGGAGGGCCGAGAGAGGGCTACTGTGGCAAATGGGGCTGTGAG
ACCACTGGACAGGCATACTGGAAGCCATCATCATCATGGGACCTAATTTCCCTTAAGCGAAGAAACACCCCTCAGAATCA
GGGCCCCTGTTATGATTCCTCAGCGGTCTCCAGTGACATCAAGGGCGCCACACCGGGGGGTCGATGCAATCCCCTAGTCC
TGGAATTCACTGACGCGGGCAAAAAGGCCAGCTGGGATGGCCCCAAAGTATGGGGACTAAGACTGTACCGATCCACAGGG
ACCGACCCGGTGACCCGGTTCTCTTTGACCCGCCAGGTCCTCAATATAGGGCCCCGCGTCCCCATTGGGCCTAATCCCGT
GATCACTGACCAGTTACCCCCCTCCCGACCCGTGCAGATCATGCTCCCCAGGCCTCCTCAGCCTCCTCCTCCAGGCGCAG
CCTCTACAGTCCCTGAGACTGCCCCACCTTCTCAACAACCTGGGACGGGAGACAGGCTGCTAAACCTGGTAGATGGAGCC
TACCAAGCTCTCAACCTCACCAGTCCTGACAAAACCCAAGAGTGCTGGTTGTGTCTGGTAGCGGGACCCCCCTACTACGA
AGGGGTTGCCGTCCTAGGTACTTATTCCAACCATACCTCTGCCCCAGCTAACTGCTCCGTGGCCTCCCAACACAAGCTGA
CCCTGTCCGAAGTGACCGGACAGGGACTCTGCGTAGGAGCAGTTCCCAAAACCCATCAGGCCCTGTGTAATACCACCCAG
AAGACGAGCGACGGGTCCTACTATCTGGCTGCTCCCGCCGGGACCATTTGGGCTTGCAACACCGGGCTCACTCCCTGCCT
ATCTACCACTGTGCTCGACCTCACCACCGATTACTGTGTCCTGGTTGAGCTCTGGCCAAAGGTGACCTACCACTCCCCTG
GTTATGTTTATGGCCAGTTTGAGAGAAAAACCAAATATAAAAGAGAGCCGGTGTCATTAACTCTGGCCCTGCTGTTGGGA
GGACTTACTATGGGCGGCATAGCTGCAGGAGTAGGAACCGGGACTACAGCCCTAGTGGCCACCAAACAATTCGAGCAGCT
CCAGGCAGCCATACATACAGACCTTGGGGCTTTAGAAAAGTCAGTCAGTGCCCTAGAAAAGTCTCTGACCTCGTTGTCTG
AGGTGGTCCTACAGAACCGGAGGGGATTAGATCTACTGTTCCTAAAAGAAGGAGGATTATGTGCTGCCCTAAAAGAAGAA
TGCTGTTTCTACGCGGACCACACTGGCGTAGTAAGAGATAGCATGGCAAAGCTAAGAGAAAGGTTGAACCAGAGACAAAA
ATTGTTCGAATCAGGACAAGGGTGGTTTGAGGGACTGTTTAACAGGTCCCCATGGTTCACGACCTTAATATCCACCATTA
TGGGCCCCTTGATAATACTTTTATTAATCCTACTCTTCGGACCCTGTATTCTCAACCGCTTGGTCCAGTTTGTAAAAGAC
AGAATTTCGGTAGTGCAGGCCCTGGTTTTGACCCAACAGTATCACCAACTCAAATCAATAGATCCAGAAGAAGTGGAATC
ACGTGAATAAAAGATTTTATTCAGTTTCCAGAAAGAGGGGGGAATGAAAGACCCCACCATCAGGCTTAGCAAGCTAGCTG
CAGTAACGCCATTTTGCAAGGCATGAAAAAGTACCAGAGCTAAGTTCTCAAAAGTTACAAGAAAGTTCAGTTAAAGATTA
ACAGTTAAAGATTAAGGCTGAATAATACCGGGACAGGGGCCAAATATCGGTGGTCAAGCACCTGGGCCCCGGCTCAGGGC
CAAGAACAGATGGCTCTCAGACGTCAGTGTTAGCAGAACTAGCTTCACTGATTTAGAAAAATAGAGGTGCACAGAGCTCT
GGCCACTCCTTGAACCTGTGTGTCTGCCAATGTTCTGACCAGGTGTGTGCCCATTGTTGAACCTTCATTAGACCCTCTCC
TCGTACCCCTCCCATACCCATTTCTTGAAAATAGACATTGTTTAGAACTAAAAAGTCCCACCTCAGTTTCCCCAAATGAC
CGAGAAATACCCCAAGCCTTATTCGAACTAACCAACCAGCTCGCTTCTCGCTTCTGTAACCGCGCTTTTTGCTCCCCAGC
CCCAGCCCTATAAAAAGGGTAAAAACTCCACACTCGGCGCGCCAGTCCTCCGATAGACTGCGTCGCCCGGGTACCCGTGT
TCCCAATAAAGCCTCTTGCTGTTTGCATCCGAATCGTGGTCTCGCTGGTCCTTGAGAGGGTCTCCTCAGATTGATTGACT
ACCCACGTCGGGGGTCTTTCA
>Pmv14
TGAAAGACCCCACCATCAGGCTTAGCAAGCTAGCTGCAGTAACGCCATTTTGCAAGGCATGAAAAAGTACCAGAGCTGAG
TTCTCAAAAGTTACAAGAAAGTTCAGTTAAAGATTAACAGTTAAAGATTAAGGCTGAATAATACTGGGACAGGGGCCAAA
TATCGGTGGTCAAGCACCTGGGCCCCGGCTCAGGGCCAAGAACAGATGGCTCTCAGACGTCAGTGTTAGCAGAACTAGCT
TCACTGATTTAAAAAAATAGAGGTGCACAGTGCTCTGGCCACTCCTTGAACCTGTGTATCTGCCAATGTTCTGACCAGGT
GTGTGCCCATTGTTAAACCTTCATTAGACCCTTTCCTCGTACCCCTCCCATACCCATTTCTTAAAAATAGACATTGTTTA
AAACTAAAAAGTCCCACCTCAGTTTCCCCAAATGACCGAGAAATACCCCAAGCCTTATTCGAACTAACCAACCAGCTCGC
TTCTCGCTTCTGTAACCGCGCTTTTTGCTCCCCAGCCCCAGCCCTATAAAAAGGGTAAAAACTCCACACTCGGCGCGCCA
GTCCTCCGATAGACTGCGTCGCCCGGGTACCCGTGTTCCCAATAAAGCCTCTTGCTGTTTGCATCCGAATCGTGGTCTCG
CTGGTCCTTGAGAGGGTCTCCTCAGATTGATTGACTACCCACGTCGGGGGTCTTTCATTTGGAGGTTCCACCGAGATTTG
GAGACCCCTGCCCAGGGACCACCGACCCCCCCGCCGGGAGGTAAGCTGGCCAGCGGTCGTTTCGTGTCTGTCTCTGTCTT
CGTGCGTGTTTGTGCCGGCATCCAATGTTTGCGCCTGCGTCTGTACTAGTTAGCTAACTAGATCTGTATCTGGCGGTTCC
GCGGAAGAACTGACGAGTTCGTATTCCCGGCCGCAGCCCCTGGGAGACGTCCCAGCGGCCTCGGGGGCCCGTTTTGTGGC
CCATTCTGTATCAGTTAACCTACCCGAGTCGGACTTTTTGGAGCTCCGCCACTGTACGTGGCTTTGTTGGGGGACGAGAG
ACAGAGACACTTCCCGCCCCCGTCTGAATTTTTGCTTTCGGTTTTACGCCGAAGCCGCGCCGCGCGTCTGACTTGTTTGT
TGTTCTTTTGTTCTTCGTTAGTTTTCTTCTGTCTTTAAGTGTTTTCGAGATCATGGGACAGACCGTAACTACCCCTCTGA
GTTTAACCTTGCAGCACTGGGGAGATGTCCAGCGCATTGCATCCAACCAGTCTGTGGATGTCAGGAAGAGGCGCTGGATT
ACCTTCTGTTCCGCTGAATGGCCAACTTTCAATGTGGGATGGCCTCAGGATGGTACTTTCAATTTAAGTATTATCTCTCA
GGTTAAGTCTAGAGTGTTTTGTCCTGGTCCCCACGGACACCCAGATCAGGTCCCATATATCGTCACCTGGGAGGCACTTG
CCTATGACCCCCCTCCGTGGGTCAAACCGTTTGTGTCTCCTAAACTTCCTCCCTTGCCGACAGCTCCCGTCCTCCCGCCC
GGTCCTTCTGCGCAACCTCCGTCCCGATCTGCCCTTTACCCTGCCCTTACCCCCTCTATAAAGTCCAAACCTCCTAAGCC
CCAGGTTCTCCCTGATAGCGGCGGACCCCTCATTGACCTTCTCACAGAGGACCCCCCGCCGTACAGAGCACAACCCTCCT
CCTCTGCCAGGGAGAACGACGAAGAAGAGGCGGCCACCACCTCCGAGGTTTCCCCCCCTTCTCCCATGGTGTCTCGACTG
CGGGGAAGGAGAGACCCTCCCGCAGTGGACTCCACCACCTCCCAGGCATTTCCACTCCGCATGGGGGGAGATGGCCAGCT

TCAGTATTGGCCGTTTTCCTCTTCGGATTTATACAATTGGAAAAATAATAACCCTTCCTTTTCTGAAGATCCAGGTAAAT
TGACGGCCTTGATTGAGTCCGTCCTCATCACCCACCAGCCCACCTGGGACGACTGTCAGCAGTTGTTGGGGACCCTGCTG
ACCGGAGAAGAAAAGCAGCGGGTGCTCCTAGAGGCTAGAAAGGCAGTCCGGGGCAATGATGGACGCCCCACTCAGTTGCC
TAATGAAGTCAATGCTGCTTTTCCCCTTGAACGCCCCGGTTGGGATTACACCACTACAGAAGGTAGGAACCACCTAGTCC
TCTATCGCCAGTTGCTCTTAGCGGGTCTCCAGAACGCGGGCAGAAGTCCCACCAATTTGGCCAAGGTAAAAGGGATAACC
CAGGGACCTAATGAGTCTCCCTCAGCCTTTTTAGAGAGACTCAAAGAGGCCTATCGCAGGTACACTCCTTATGACCCTGA
GGACCCAGGGCAAGAAACCAATGTGTCTATGTCATTCATCTGGCAGTCTGCCCCGGATATCGGGCGAAAGTTAGAGCGGT
TAGAAGATTTAAAGAGCAAGACCTTAGGAGACTTAGTGAGGGAAGCTGAAAAGATCTTTAATAAGCGAGAAACCCCGGAA
GAAAGAGAGGAACGTATCAAGAGAGAAACAGAGGAAAAAGAAGAACGCCGTAGGGCAGAGGATGAGCAGAGAGAGAAAGA
AAGGGACCGCAGAAGACATAGAGAGATGAGCAAGCTCTTGGCCACTGTAGTTATTGGTCAGAGACAGGATAGACAGGGGG
GAGAGCGGAGGAGGCCCCAACTTGATAAGGACCAATGCGCCTACTGCAAAGAAAAGGGACACTGGGCTAAGGACTGCCCA
AAGAAGCCACGAGGGCCCCGAGGACTGAGGCCCCAGACCTCCCTCCTAACCTTAGGTGACTAGGGAGGTCAGGGTCAGGA
GCCCCCCCCTGAACCCAGGATAACCCTCAAAGTCGGGGGGCAACCCGTCACCTTCCTGGTAGATACTGGGGCCCAACACT
CCGTGCTGACCCAAAATCCTGGACCCCTAAGTGACAAGGCTGCCTGGGTCCAAGGGGCTACTGGAGGAAAGCGGTATCGC
TGGACCACGGATCGCAAAGTACATCTAGCTACCGGTAAGGTCACCCACTCTTTCCTCCATGTACCAGACTGCCCCTATCC
TCTGCTAGGAAGAGACTTGCTGACTAAACTAAAAGCCCAAATCCACTTCGAGGGATCAGGAGCTCAGGTTATGGGACCAA
TGGGACTGCCCCTGCAAGTGCTGACCCTAAACATAGAAGATGAGTATCAGCTACATGAGACCTCAAAAGAGCCGGATGTT
TCTCTAGGGTCCACCTGGCTTTCTGATTTTCCCCAGGTCTGGGCGGAAACCGGGGGCATGGGACTGGCAGTTCGCCAAGC
TCCTCTGATCATACCTCTGAAGGCAACCTCTACCCCCGTGTCCATAAAACAATACCCCATGTCACAAGAAGCCAGACTGG
GGATCAAGCCCCACATACAGAGACTGTTGGACCAGGGAATACTGGTACCCTGCCAGTCCCCCTGGAACACGCCCCTGCTA
CCCGTTAAGAAACCAGGGACTAATGATTATAGGCCTGTCCAGGATCTGAGAGAAGTCAACAAGCGGGTGGAAGACATCCA
CCCCACCGTGCCCAACCCTTACAACCTCTTGAGCGGGCTCCCACCGTCCCACCAGTGGTACACTGTGCTTGATTTAAAGG
ATGCCTTTTTCTGCCTGAGACTCCACCCCACCAGTCAGCCTCTCTTCGCCTTTGAGTGGAGAGATCCAGAGATGGGAATC
TCAGGACAATTGACCTGGACCAGACTCCCACAGGGTTTCAAAAACAGTCCCACCCTGTTTGATGAGGCACTGCACAGAGA
CCTAGCAGACTTCCGGATCCAGCACCCAGACTTGATCCTGCTACAGTACGTGGATGACTTACTGCTGGCCGCCACTTCTG
AGCTAGACTGCCAACAAGGTACTCGGGCCCTGTTACAAACCCTAGGGAACCTCGGGTATCGGGCCTCGGCCAAGAAAGCC
CAAATTTGCCAGAAACAGGTCAAGTATCTGGGGTATCTTCTAAAAGAGGGTCAGAGATGGCTGACTGAGGCCAGAAAAGA
GACTGTGATGGGGCAGCCTACTCCGAAGACCCCTCGACAACTAAGGGAGTTCCTAGGGACGGCAGGCTTCTGTCGCCTCT
GGATCCCTGGGTTTGCAGAAATGGCAGCCCCCTTGTACCCTCTCACCAAAACGGGGACTCTGTTTAATTGGGGCCCAGAC
CAGCAAAAGGCCTATCAAGAAATCAAACAGGCTCTTCTAACTGCCCCAGCCCTGGGATTGCCAGATTTGACTAAGCCCTT
TGAACTCTTTGTCGACGAGAAGCAGGGCTACGCCAAAGGCGTCCTAACGCAAAAACTGGGACCTTGGCGTCGGTCGGTGG
CCTACCTGTCCAAAAAGCTAGACCCAGTGGCAGCTGGGTGGCCCCCTTGCCTACGGATGGTAGCAGCCATTGCCGTTCTG
ACAAAAGATGCAGGCAAGCTAACCATGGGACAGCCGCTAGTCATCCTGGCCCCCCATGCAGTAGAGGCACTAGTCAAGCA
ACCCCCTGACCGCTGGCTATCCAACGCCCGCATGACCCACTATCAGGCGATGCTCCTAGATACGGACCGGGTCCAGTTCG
GACCGGTGGTAGCCCTAAACCCGGCTACGTTGCTCCCCCTACCGGGGAAAGAGCCTCACCATGACTGCCTCGAGATCTTG
GCCGAGACACACGGAACCAGACCAGACCTCACGGACCAGCCCCTCCCAGACGCCGACCACACCTGGTATACAGATGGAAG
CAGCTTCCTGCAAGAGGGACAACGTAGGGCTGGAGCAGCGGTGACCACCGAGACCGAGGTAATCTGGGCCAAGGCGTTGC
CAGCCGGGACATCCGCCCAGCGAGCTGAACTAATAGCACTCACCCAGGCCCTAAAGATGGCAGAAGGTAAGAAGCTAAAT
GTTTATACTGATAGCCGCTATGCCTTTGCTACCGCCCATGTCCATAAAAAAATATATAGGAGACGTGGGTTACTCACCTC
AGAAGGCAAAGAGATCAAGAACAAGGGCGAAATCTTGGCCTTACTGAAAGCTCTCTTTCTGCCCAAAAGACTCAGTATAA
TTCACTGCCCAGGACATCAGAAAGGCAATAGTGCTGAAGCTAAAGGCAACCGAATGGCGGACCAGGCAGCCCGGGAAGCA
GCCATGGGGACTGACACAAAGACCTCCTCACTTCTCATAGAGACCTCAACCCCGTACACTCCAGACTTCTTCCATTATAC
TGAGACAGATATAAAGAACCTACAAGAGTTGGGAGCCACATATGATAGAGAGAAAAAATATTGGGTCCTGCAAGGTAAAC
CTGTGATGCCTGACCAGTTCACCTTTAAATTATTAGACTTCCTTCACCAGCTCACCCACCTTAGCTATCAGAAGATGAGG
GCACTTCTAGACAGAAAAGAAAGCCCCTATTACATGCTAAATAAAGATAAGATCCTCCACGAGGTGGCGGAATCATGCCA
AGCCTGTGTCCAAGTAAATGCCAGTAAGACTAAGATCAGGGCCGGAACACGAGTAAGAGGACATCGACCAGGCACCCATT
GGGAAATTGACTTTACTAAAGTGAAGCCCGGACTGTATGGGTACAAGTATCTCCTGGTATTCGTGGACACGTTCTCTGGC
TGGGTTGAAGCCTTCCCAACCAAACATGAGACTGCCAAAATAGTGACCAAGAAACTTCTGGAAAAAATATTTCCAAGGTT
TGGAATGCCCCAAGTGTTGGGGACTGATAATGGGCCTGCCTTCGTCTCCCAGGTAAGTCAGTCGGTGGCCAAGCTACTGG
GGATTGATTGAAAACTACATTGTGCTTACAGACCCCAGAGTTCAGGTCAGGTAGAAAGAATGAATAGGACAATCAAGGAG
ACTTTGACCAAATTAACGCTTGCAACTGGCACTAGAGACTGGGTACTCCTACTTCCCTTAGCCCTCTACCGAGCCCGCAA
CACTCCAGGCCCCCATGGACTCACTCCGTATGAAATCCTGTATGGGGCGCCCCCGCCCCTTGTTAATTTCCATGATCCTG
AAATGTCAAAGTTTACTAATAGCCCCTCTCTCCAAGCTCACTTACAGGCCCTCCAAGCAGTACAACGAGAGGTCTGGAAG
CCACTGGCCGCTGCCTATCAGGACCAGCTAGACCAGCCAGTGATACCACACCCCTTCCGTGTCGGCGACACCGTGTGGGT
ACGCCGGCACCAGACTAAAAACTTGGAACCTCGCTGGAAAGGACCCTACACCGTCCTGCTGACCACCCCCACCGCTCTCA
AAGTAGACGGCATCGCTGCGTGGATCCACGCCGCTCACATAAAAGCGGCGACAACCCCTCCGGCCGGAACAGCATCAGGA
CCGACATGGAAGGTCCAGCGTTCTCAAAACCCCTTAAAGATAAGATTAACCCGTGGGGCCCCCTAATAGTCCTGGGGATC

TTAATAAGGGCAGGAGTATCAGTACAACATGACAGCCCTCATCAGGTCTTCAATGTTACTTAGAGAGTTACCAACTTAAT
GACAGGACAAACAGCTAATGCTACCTCCCTCCTGGGGACAATGACCGATGCCTTTCCTAAACTGTACTTTGACTTGTGCG
ATTTAATAGGGGACGACTGGGATGAGACTGGACTCGGGTGTCGCACTCCCGGGGGAAGAAAAAGGGCAAGAACATTTGAC
TTCTATGTTTGCCCCGGGCATACTGTACCAACAGGGTGTGGAGGGCCGAGAGAGGGCTACTGTGGCAAATGGGGCTGTGA
GACCACTGGACAGGCATACTGGAAGCCATCATCATCATGGGACCTAATTTCCCTTAAGCGAGGAAACACCCCTCGGAATC
AGGGCCCCTGTTATGATTCCTCAGCGGTCTCCAGTGACATCAAGGGCGCCACACCGGGGGGTCGATGCAATCCCCTAGTC
CTGGAATTCACTGACGCGGGCAAAAAGGCCAGCTGGGATGGCCCCAAAGTATGGGGACTAAGACTGTACCGATCCACAGG
GACCGACCCGGTGACCCGGTTCTCTTTGACCCGCCAGGTCCTCAATATAGGGCCCCGCGTCCCCATTGGGCCTAATCCCG
TGATCACTGACCAGTTACCCCCCTCCCGACCCGTGCAGATCATGCTCCCCAGGCCTCCTCAGCCTCCTCCTCCAGGCGCA
GCCTCTATAGTCCCTGAGACTGCCCCACCTTCTCAACAACCTGGGACGGGAGACAGGCTGCTAAACCTGGTAGATGGAGC
CTACCAAGCTCTCAACCTCACCAGTCCTGACAAAACCCAAGAGTGCTGGTTGTGTCTGGTAGCGGGACCCCCCTACTACG
AAGGGGTTGCCGTCCTAGGTACTTATTCCAACCATACCTCTGCCCCAGCTAACTGTTCCGTGGCCTCCCAACACAAGCTG
ACCCTGTCCAAAGTGACCGGACAGGGACTCTGCGTAGGAGCAGTTCCCAAAACCCATCAGGCCCTGTGTAATACCACCCA
GAAGACGAGCGACGGGTCCTACTATCTGGCTGCTCCCGCCGGGACCATTTGGGCTTGCAACACCGGGCTCACTCCCTGCC
TATCTACCACTGTACTCGACCTCACCACCGATTACTGTGTCCTGGTTGAGCTCTGGCCAAAGGTGACCTACCACTCCCCT
GGTTATGTTTATGGCCAGTTTGAGAGAAAAACCAAATATAAAAGAGAGCCGGTGTCATTAACTCTGGCCCTGCTGTTGGG
AGGACTTACTATGGGCGGCATAGCTGCAGGAGTAGGAACCGGGACTACAGCCCTAGTGGCCACCAAACAATTCGAGCAGC
TCCAGGCAGCCATACATACAGACCTTGGGGCTTTAAAAAAGTCAGTCAGTGCCCTAGAAAAGTCTCTGACCTCGTTGTCT
GAGGTGGTCCTACAGAACCGGAGGGGATTAGATCTACTGTTCCTAAAAGAAGGAGGTTTATGTGCTGCCCTAAAAGAAGA
ATGCTGTTTCTACGCAGACCACACTGGCGTAGTAAGAGATAACATGGCAAAGCTAAGAGAAAGGTTGAACCAGAGACAAA
AATTGTTCGAATCAGGACAAGGGTGGTTTGAGGGACTGTTTAACAGGTCCCCATGGTTCACGACCTTAATATCCACCATT
ATGGGCCCCTTAATAATACTTTTATTAATCCTACTCTTCGGACCCTGTATTCTCAACCGCTTGGTCCAGTTTGTAAAAGA
CAGAATTTCGGTAGTGCAGGCCCTGGTTTTGACCCAACAGTATCACCAACTCAAATCAATAGATCCAGAAAAAGTGGAAT
CACGTGAATAAAAGATTTTATTCAGTTTCCAGAAAGAGGGGGGAATGAAAGACCCCACCATCAGGCTTAGCAAGCTAGCT
GCAGTAACGCCATTTTGCAAGGCATGAAAAAGTACCAGAGCTGAGTTCTCAAAAGTTACAAGAAAGTTCAGTTAAAGATT
AACAGTTAAAGATTAAGGCTGAATAATACTGGGACAGGGGCCAAATATCGGTGGTCAAGCACCTGGGCCCCGGCTCAGGG
CCAAGAACAGATGGCTCTCAGACGTCAGTGTTAGCAGAACTAGCTTCACTGATTTAAAAAAATAGAGGTGCACAGTGCTC
TGGCCACTCCTTGAACCTGTGTATCTGCCAATGTTCTGACCAGGTGTGTGCCCATTGTTAAACCTTCATTAGACCCTTTC
CTCGTACCCCTCCCATACCCATTTCTTAAAAATAGACATTGTTTAAAACTAAAAAGTCCCACCTCAGTTTCCCCAAATGA
CCGAGAAATACCCCAAGCCTTATTCGAACTAACCAACCAGCTCGCTTCTCGCTTCTGTAACCGCGCTTTTTGCTCCCCAG
CCCCAGCCCTATAAAAAGGGTAAAAACTCCACACTCGGCGCGCCAGTCCTCCGATAGACTGCGTCGCCCGGGTACCCGTG
TTCCCAATAAAGCCTCTTGCTGTTTGCATCCGAATCGTGGTCTCGCTGGTCCTTGAGAGGGTCTCCTCAGATTGATTGAC
TACCCACGTCGGGGGTCTTTCA
>Pmv15
TGAAAGACCCCACCATCAGGCTTAGCAAGCTAGCTGCAGTAACGCCATTTTGCAAGGCATGAAAAAGTACCAGAGCTGAG
TTCTCAAAAGTTACAAGAAAGTTCAGTTAAAGATTAACAGTTAAAGATTAAGGCTGAATAATACTGGGACAGGGGCCAAA
TATCGGTGGTCAAGCACCTGGGCCCCGGCTCAGGGCCAAGAACAGATGGCTCTCAGACGTCAGTGTTAGCAGAACTAGCT
TCACTGATTTAAAAAAATAGAGGTGCACAGTGCTCTGGCCACTCCTTGAACCTGTGTGTCTGCCAATGTTCTGACCAGGT
GTGTGCCCATTGTTGAACCTTCATTAGACCCTTTCCTCGTACCCCTCCCATACCCATTTCTTAAAAATAGACATTGTTTA
GAACTAAAAAGTCCCACCTCAGTTTCCCCAAATGACCGAGAAATACCCCAAGCCTTATTCGAACTAACCAACCAGCTCGC
TTCTCGCTTCTGTAACCGCGCTTTTTGCTCCCCAGCCCCAGCCCTATAAAAAGGGTAAAAACTCCACACTCGGCGCGCCA
GTCCTCCGATAGACTGCGTCGCCCGGGTACCCGTGTTCCCAATAAAGCCTCTTGCTGTTTGCATCCGAATCGTGGTCTCG
CTGGTCCTTGAGAGGGTCTCCTCAGATTGATTGACTACCCACGTCGGGGGTCTTTCATTTGGAGGTTCCACCGAGATTTG
GAGACCCCTGCCCAGGGACCACCGACCCCCCGCCGGGAGGTAAGCTGGCCAGCGGTCGTTTCGTGTCTGTCTCTGTCTTC
GTGCGTGTTTGTGCCGGCATCCAATGTTTGCGCCTGCGTCTGTACTAGTTAGCTAACTAGATCTGTATCTGGCGGTTCCG
CGGAAGAACTGACGAGTTCGTATTCCCGGCCGCAGCCCCTGGGAGACGTCCCAGCGGCCTCGGGGGCCCGTTTTGTGGCC
CATTCTGTATCAGTTAACCTACCCGAGTCGGACTTTTTGGAGCTCCGCCACTGTACGTGGCTTTGTTGGGGGACGAGAGA
CAGAGACACTTCCCGCCCCCGTCTGAATTTTTGCTTTCGGTTTTACGCCGAAGCCGCGCCGCGCGTCTGACTTGTTTGTT
GTTCTTTTGTTCTTCGTTAGTTTTCTTCTGTCTTTAAGTGTTTTCGAGATCATGGGACAGACCGTAACTACCCCTCTGAG
TTTAACCTTGCAGCACTGGGGAGATGTCCAGCGCATTGCATCCAACCAGTCTGTGGATGTCAGGAAGAGGCGCTGGATTA
CCTTCTGTTCCGCTGAATGGCCAACTTTCAATGTGGGATGGCCTCAGGATGGTACTTTCAATTTAAGTATTATCTCTCAG
GTTAAGTCTAGAGTGTTTTGTCCTGGTCCCCACGGACACCCGGATCAGGTCCCATATATCGTCACCTGGGAGGCACTTGC
CTATGACCCCCCTCCGTGGGTCAAACCGTTTGTGTCTCCTAAACTTCCTCCCTTGCCGACAGCTCCCGTCCTCCCGCCCG
GTCCTTCTGCGCAACCTCCGTCCCGATCTGCCCTTTACCCTGCCCTTACCCCCTCTATAAAGTCCAAACCTCCTAAGCCC
CAGGTTCTCCCTGATAGCGGCGGACCCCTCATTGACCTTCTCACAGAGGACCCCCCGCCGTACAGAGCACAACCCTCCTC
CTCTGCCAGGGAGAACGACGAAGAAGAGGCGGCCACCACCTCCGAGGTTTCCCCCCCTTCTCCCATGGTGTCTCGACTGC
GGGGAAGGAGAGACCCTCCCGCAGTGGACTCCACCACCTCCCAGGCATTTCCACTCCGCATGGGGGGAGATGGCCAGCTT

CAGTATTGGCCGTTTTCCTCTTCGGATTTATACAATTGGAAAAATAATAACCCTTCCTTTTCTGAAGATCCAGGTAAATT
GACGGCCTTGATTGAGTCCGTCCTCATCACCCACCAGCCCACCTGGGACGACTGTCAGCAGTTGTTGGGGACCCTGCTGA
CCGGAGAAGAAAAGCAGCGGGTGCTCCTAGAGGCTAGAAAGGCAGTCCGGGGCAATGATGGACGCCCCACTCAGTTGCCT
AATAAAGTCAATGCTGCTTTTCCCCTTGAACGCCCCGGTTGGGATTACACCACTACAGAAGGTAGGAACCACCTAGTCCT
CTATCGCCAGTTGCTCTTAGCGGGTCTCCAGAACGCGGGCAGAAGTCCCACCAATTTGGCCAAGGTAAAAGGGATAACCC
AGGGACCTAATGAGTCTCCCTCAGCCTTTTTAGAGAGACTCAAAGAGGCCTATCGCAGGTACACTCCTTATGACCCTGAG
GACCCAGGGCAAGAAACCAATGTGTCTATGTCATTCATCTGGCAGTCTGCCCCGGATATCGGGCGAAAGTTAGAGCGGTT
AGAAGATTTAAAGAGCAAGACCTTAGGAGACTTAGTGAGGGAAGCTGAAAAGATCTTTAATAAGCGAGAAACCCCGGAAG
AAAGAGAGGAACGTATCAAGAGAGAAATAGAGGAAAAAGAAGAACGCCGTAGGGCAGAGGATGAGCAGAGAGAGAAAGAA
AGGGACCGCAGAAGACATAGAGAGATGAGCAAGCTCTTGGCCACTGTAGTTATTGGTCAGAGACAGGATAGACAGGGGGG
AGAGCGGAGGAGGCCCCAACTTGATAAGGACCAATGCGCCTACTGCAAAGAAAAGGGACACTGGGCTAAGGACTGCCCAA
AGAAGCCACGAGGGCCCCGAGGACTGAGGCCCCAGACCTCCCTCCTAACCTTAGGTGACTAGGGAGGTCAGGGTCAGGAG
CCCCCCCCTGAACCCAGGATAACCCTCAAAGTCGGGGGGCAACCCGTCACCTTCCTGGTAGATACTGGGGCCCAACACTC
CGTGCTGACCCAAAATCCTGGACCCCTAAGTGACAAGGCTGCCTGGGTCCAAGGGGCTACTGGAAGAAAGCGGTATCGCT
GGACCACGGATCGCAAAGTACATCTAGCTACCGGTAAGGTCACCCACTCTTTCCTCCATGTACCAGACTGCCCCTATCCT
CTGCTAGGAAGAGACTTGCTGACTAAACTAAAAGCCCAAATCCACTTCGAGGGATCAGGAGCTCAGGTTATGGGACCAAT
GGGACTGCCCCTGCAAGTGCTGACCCTAAACATAGAAGATGAGTATCGGCTACATGAGACCTCAAAAGAGCCGGATGTTT
CTCTAGGGTCCACCTGGCTTTCTGATTTTCCCCAGGTCTGGGCGGAAACCGGGGGCATGGGACTGGCAGTTCGCCAAGCT
CCTCTGATCATACCTCTGAAGGCAACCTCTACCCCCGTGTCCATAAAACAATACCCCATGTCACAAGAAGCCAGACTGGG
GATCAAGCCCCACATACAGAGACTGTTGGACCAGGGAATACTGGTACCCTGCCAGTCCCCCTGGAACACGCCCCTGCTAC
CCGTTAAGAAACCAGGGACTAATGATTATAGGCCTGTCCAGGATCTGAGAGAAGTCAACAAGCGGGTGGAAGACATCCAC
CCCACCGTGCCCAACCCTTACAACCTCTTGAGCGGGCTCCCACCGTCCCACCAGTGGTACACTGTGCTTGATTTAAAGGA
TGCCTTTTTCTGCCTGAGACTCCACCCCACCAGTCAGCCTCTCTTCGCCTTTGAGTGGAGAGATCCAGAGATGGGAATCT
CAGGACAATTGACCTGGACCAGACTCCCACAGGGTTTCAAAAACAGTCCCACCCTGTTTGATGAGGCACTGCACAGAGAC
CTAGCAGACTTCCGGATCCAGCACCCAGACTTGATCCTGCTACAGTACGTGGATGACTTACTGCTGGCCGCCACTTCTGA
GCTAGACTGCCAACAAGGTACTCGGGCCCTGTTACAAACCCTAGAGAACCTCGGGTATCGGGCCTCGGCCAAGAAAGCCC
AAATTTGCCAGAAACAGGTCAAGTATCTGGGGTATCTTCTAAAAGAGGGTCAGAGATGGCTGACTGAGGCCAGAAAAGAG
ACTGTGATGGGGCAGCCTACTCCGAAGACCCCTCGACAACTAAGGGAGTTCCTAGGGACGGCAGGCTTCTGTCGCCTCTG
GATCCCTGGGTTTGCAGAAATGGCAGCCCCCTTGTACCCTCTCACCAAAACGGGGACTCTGTTTAATTGGGGCCCAGACC
AGCAAAAGGCCTATCAAGAAATCAAACAGGCTCTTCTAACTGCCCCAGCCCTGGGATTGCCAGATTTGACTAAGCCCTTT
GAACTCTTTGTCGACGAGAAGCAGGGCTACGCCAAAGGCGTCCTAACGCAAAAACTGGGACCTTGGCGTCGGCCGGTGGC
CTACCTGTCCAAAAAGCTAGACCCAGTGGCAGCTGGGTGGCCCCCTTGCCTACGGATGGTAGCAGCCATTGCCGTTCTGA
CAAAAGATGCAGGCAAGCTAACCATGGGACAGCCGCTAGTCATCCTGGCCCCCCATGCAGTAGAGGCACTAGTCAAGCAA
CCCCCTGACCGCTGGCTATCCAACGCCCGCATGACCCACTATCAGGCGATGCTCCTAGATACGGACCGGGTCCAGTTCGG
ACCGGTGGTAGCCCTAAACCCGGCTACGTTGCTCCCCCTACCGGGGAAAGAGCCTCACCATGACTGCCTCGAGATCTTGG
CCGAGACACACGGAACCAGACCAGACCTCACGGACCAGCCCCTCCCAGACGCCGACCACACCTGGTATACAGATGAAAGC
AGCTTCCTGCAAGAGGGACAACGTAGGGCTGGAGCAGCGGTGACCACCGAGACCGAGGTAATCTGGGCCAAGGCGTTGCC
AGCCGGGACATCCGCCCAGCGAGCTGAACTAATAGCACTCACCCAGGCCCTAAAGATGGCAGAAGGTAAGAAGCTAAATG
TTTATACTGATAGCCGCTATGCCTTTGCTACCGCCCATGTCCATGGAGAAATATATAGGAGACGTGGGTTGCTCACCTCA
GAAGGCAAGGAGATCAAGAACAAGGGCGAAATCTTAGCCTTACTAAAAGCTCTCTTTCTGCCCAAAAGACTCAGTATAAT
TCACTGCCCAGGACATCAGAAAGGCAATAGTGCTGAAGCTAAAGGCAACCGAATGGCGGACCAGGCAGCCCGGGAAGCAG
CCATGGGGACTGACACAAAGACCTCCTCACTTCTCATAGAGACCTCAACCCCGTACACTCCAGACTTCTTCCATTATACT
GAGACAGATATAAAGAACCTACAAGAGTTGGGAGCCACATATGATAGAGAAAAAAAATATTGGGTCCTGCAAGGTAAACC
TGTGATGCCTGACCAGTTCACCTTTAAATTATTAGACTTCCTTCACCAGCTCACCCACCTTAGCTATCAGAAGATGAGGG
CACTTCTAGACAGGAAAGAAAGCCCCTATTACATGCTAAATAAAGATAAGATCCTCCACGAGGTGGCGGAATCATGCCAA
GCCTGTGTCCAAGTAAATGCCAGTAAGACTAAGATCAGGGCCGGAACACGAGTAAGAGGACATCGACCAGGCACCCATTG
GGAAATTGACTTTACTGAAGTGAAGCCCGGACTGTATGGGTACAAGTATCTCCTGGTATTCGTGGACACGTTCTCTGGCT
GGGTTGAAGCCTTCCCAACCAAACATGAGACTGCCAAAATAGTGACCAAGAAACTTCTGGAAGAAATATTTCCAAGGTTT
GGAATGCCCCAAGTGTTGGGGACTGATAATGGGCCTGCCTTCGTCTCCCAGGTAAGTCAGTCGGTGGCCAAGCTACTGGG
GATTGATTGGAAACTACATTGTGCTTACAGACCCCAGAGTTCAGGTCAGGTAGAAAGAATGAATAGGACAATCAAGGAGA
CTTTGACCAAATTAACGCTTGCAACTGGCACTAGAGACTGGGTACTCCTACTTCCCTTAGCCCTCTACCGAGCCCGCAAC
ACTCCGGGCCCCCATGGACTCACTCCGTATGAAATCCTGTATGGGGCGCCCCCGCCCCTTGTTAATTTCCATGATCCTGA
AATGTCAAAGTTTACTAATAGCCCCTCTCTCCAAGCTCACTTACAGGCCCTCCAAGCAGTACAACGAGAGGTCTGGAAGC
CACTGGCCGCTGCCTATCAGGACCAGCTGGACCAGCCAGTGATACCACACCCCTTCCGTGTCGGCGACACCGTGTGGGTA
CGCCGGCACCAGACTAAGAACTTGGAACCTCGCTGGAAAGGACCCTACACCGTCCTGCTGACCACCCCCACCGCTCTCAA
AGTAGACGGCATCGCTGCGTGGATCCACGCCGCTCACGTAAAAGCGGCGACAACCCCTCCGGCCGGAACAGCATCAGGAC
CGACATAGAAGGTCCAGCGTTCTCAAAACCCCTTAAAGATAAGATTAACCCGTGGGGCCCCCTAATAGTCCTGGGGATCT

TAATAAGGGCAGGAGTATCAGTACAACATGACAGCCCTCATCAGGTCTTCAATGTTACTTGGAGAGTTACCAACTTAATG
ACAGGACAAACAGCTAATGCTACCTCCCTCCTGGGGACAATGACCGATGCCTTTCCTAAACTGTACTTTGACTTGTGCGA
TTTAATAGGGGACGACTGGGATGAGACTGGACTCGGGTGTCGCACTCCCGGGGGAAGAAAAAGGGCAAGAACATTTGACT
TCTATGTTTGCCCCGGGCATACTGTACCAACAGGGTGTGGAGGGCCGAGAGAGGGCTACTGTGGCAAATGGGGCTGTGAG
ACCACTGGACAGGCATACTGGAAGCCATCATCATCATGGGACCTAATTTCCCTTAAGCGAGGAAACACCCCTCGGAATCA
GGGCCCCTGTTATGATTCCTCAGCGGTCTCCAGTGACATCAAGGGCGCCACACCGGGGGGTCGATGCAATCCCCTAGTCC
TGGAATTCACTGACGCGGGCAAAAAGGCCAGCTGGGATGGCCCCAAAGTATGGGGACTAAGACTGTACCGATCCACAGGG
ACCGACCCGGTGACCCGGTTCTCTTTGACCCGCCAGGTCCTCAATATAGGGCCCCGCGTCCCCATTGGGCCTAATCCCGT
GATCACTGACCAGTTACCCCCCTCCCGACCCGTGCAGATCATGCTCCCCAGGCCTCCTCAGCCTCCTCCTCCAGGCGCAG
CCTCTATAGTCCCTGAGACTGCCCCACCTTCTCAACAACCTGGGACGGGAGACAGGCTGCTAAACCTGGTAGATGGAGCC
TACCAAGCTCTCAACCTCACCAGTCCTGACAAAACCCAAGAGTGCTGGTTGTGTCTGGTAGCGGGACCCCCCTACTACGA
AGGGGTTGCCGTCCTAGGTACTTATTCCAACCATACCTCTGCCCCAGCTAACTGCTCCGTGGCCTCCCAACACAAGCTGA
CCCTGTCCGAAGTGACCGGACAGGGACTCTGCGTAGGAGCAGTTCCCAAAACCCATCAGGCCCTGTGTAATACCACCCAG
AAGACGAGCAACGGGTCCTACTATCTGGCTGCTCCCGCCGGGACCATTTGGGCTTGCAACACCGGGCTCACTCCCTGCCT
ATCTACCACTGTACTCGACCTCACCACCGATTACTGTGTCCTGGTTGAGCTCTGGCCAAAGGTGACCTACCACTCCCCTG
GTTATGTTTATGGCCAGTTTGAGAGAAAAACCAGATATAAAAGAGAGCCGGTGTCATTAACTCTGGCCCTGCTGTTGGGA
GGACTTACTATGGGCGGCATAGCTGCAGGAGTAGGAACCGGGACTACAGCCCTAGTGGCCACCAAACAATTCGAGCAGCT
CCAGGCAGCCATACATACAGACCTTGGGGCTTTAAAAAAGTCAGTCAGTGCCCTAAAAAAGTCTCTGACCTCGTTGTCTG
AGGTGGTCCTACAGAACCGGAGGGGATTAGATCTACTGTTCCTAAAAGAAGGAGGATTATGTGCTGCCCTAAAAGAAGAA
TGCTGTTTCTACGCGGACCACACTGGCGTAGTAAGAGATAGCATGGCAAAGCTAAGAGAAAGGTTGAACCAGAGACAAAA
ATTGTTCAAATCAGGACAAGGGTGGTTTGAGGGACTGTTTAACAGGTCCCCATGGTTCACGACCTTAATATCCACCATTA
TGGGCCCCTTGATAATACTTTTATTAATCCTACTCTTCGGACCCTGTATTCTCAACCGCTTGGTCCAGTTTGTAAAAGAC
AGAATTTCGGTAGTGCAGGCCCTAGTTTTGACCCAACAGTATCACCAACTCAAATCAATAGATCCAGAAGAAGTGGAATC
ACGTGAATAAAAGATTTTATTCAGTTTCCAGAAAGAGGGGGGAATGAAAGACCCCACCATCAGGCTTAGCAAGCTAGCTG
CAGTAACGCCATTTTGCAAGGCATGAAAAAGTACCAGAGCTGAGTTCTCAAAAGTTACAAGAAAGTTCAGTTAAAGATTA
ACAGTTAAAGATTAAGGCTGAATAATACTGGGACAGGGGCCAAATATCGGTGGTCAAGCACCTGGGCCCCGGCTCAGGGC
CAAGAACAGATGGCTCTCAGACGTCAGTGTTAGCAGAACTAGCTTCACTGATTTAAAAAAATAGAGGTGCACAGTGCTCT
GGCCACTCCTTGAACCTGTGTGTCTGCCAATGTTCTGACCAGGTGTGTGCCCATTGTTGAACCTTCATTAGACCCTTTCC
TCGTACCCCTCCCATACCCATTTCTTAAAAATAGACATTGTTTAGAACTAAAAAGTCCCACCTCAGTTTCCCCAAATGAC
CGAGAAATACCCCAAGCCTTATTCGAACTAACCAACCAGCTCGCTTCTCGCTTCTGTAACCGCGCTTTTTGCTCCCCAGC
CCCAGCCCTATAAAAAGGGTAAAAACTCCACACTCGGCGCGCCAGTCCTCCGATAGACTGCGTCGCCCGGGTACCCGTGT
TCCCAATAAAGCCTCTTGCTGTTTGCATCCGAATCGTGGTCTCGCTGGTCCTTGAGAGGGTCTCCTCAGATTGATTGACT
ACCCACGTCGGGGGTCTTTCA
>Pmv16
TGAAAGACCCCACCATCAGGCTTAGCAAGCTAGCTGCAGTAACGCCATTTTGCAAGGCATGAAAAAGTACCAGAGCTGAG
TTCTCAAAAGTTACAAGAAAGTTCAGTTAAAGATTAACAGTTAAAGATTAAGGCTGAATAATACTGGGACAGGGGCCAAA
TATCGGTGGTCAAGCACCTGGGCCCCGGCTCAGGGCCAAGAACAGATGGCTCTCAGACGTCAGTGTTAGCAGAACTAGCT
TCACTGATTTAGAAAAATAGAGGTGCACAGTGCTCTGGCCACTCCTTGAACCTGTGTGTCTGCCAATGTTCTGACCAGGT
GTGTGCCCATTGTTGAACCTTCATTAGACCCTTTCCTCGTACCCCTCCCATACCCATTTCTTGAAAATAGACATTGTTTA
GAACTAAAAAGTCCCACCTCAGTTTCCCCAAATGACCGAGAAATACCCCAAGCCTTATTCGAACTAACCAACCAGCTCGC
TTCTCGCTTCTGTAACCGCGCTTTTTGCTCCCCAGCCCCAGCCCTATAAAAAGGGTAAAAACTCCACACTCGGCGCGCCA
GTCCTCCGATAGACTGCGTCGCCCGGGTACCCGTGTTCTCAATAAAGCCTCTTGCTGTTTGCATCCGAATCGTGGTCTCG
CTGGTCCTTGAGAGGGTCTCCTCAGATTGATTGACTACCCACGTCGGGGGTCTTTCATTTGGAGGTCCCACCGAGATTTG
GAGACCCCTGCCCAGGGACCACCGACCCCCCGCCGGGAGGTAAGCTGGCCAGCGGTCGTTTCGTGTCTGTCTCTGTCTTC
GTGCGTGTTTGTGCCGGCATCCAATGTTTGCGCCTGCGTCTGTACTAGTTAGCTAACTAGATCTGTATCTGGCGGTTCCG
CGGAAGAACTGACGAGTTCGTATTCCCGGCCGCAGCCCCTGGGAGACGTCCCAGCGGCCTCGGGGGCCCGTTTTGTGGCC
CATTCTGTATCAGTTAACCTACCCGAGTCGGACTTTTTGGAGCTCCGCCACTGTACGTGGCTTTGTTGGGGGACGAGAGA
CAGAGACACTTCCCGCCCCCGTCTGAATTTTTGCTTTCGGTTTTACGCCGAAGCCGCGCCGCGCGTCTGATTTGTTTGTT
GTTCTTTTGTTCTTCGTTAGTTTTCTTCTGTCTTTAAGTGTTTTCGAGATCATGGGACAGACCGTAACTACCCCTCTGAG
TTTAACCTTGCAGCACTGGGGAGATGTCCAGCGCATTGCATCCAACCAGTCTGTGGATGTCAAGAAGAGGCGCTAGATTA
CCTTCTGTTCCGCTGAATGGCCAACTTTCAATGTGGGATGGCCTCAGGATGGTACTTTCAATTTAAGTATTATCTCTCAG
GTTAAGTCTAGAGTGTTTTGTCCTGGTCCCCACGGACACCCGGATCAGGTCCCATATATCGTCACCTGGGAGGCACTTGC
CTATGACCCCCCTCCGTGGGTCAAACCGTTTGTGTCTCCTAAACTTCCTCCCTTGCCGACAGCTCCCGTCCTCCCGCCCG
GTCCTTCTGCGCAACCTCCGTCCCGATCTGCCCTTTACCCTGCCCTTACCCCCTCTATAAAGTCCAAACCTCCTAAGCCC
CAGGTTCTCCCTGATAGCGGCGGACCCCTCATTGACCTTCTCACAGAGGACCCCCCGCCGTACAGAGCACAACCCTCCTC
CTCTGCCAGGGAGAACGACGAAGAAGAGGCGGCCACCACCTCCGAGGTTTCCCCCCCTTCTCCCATGGTGTCTCGACTGC
GGGGAAGGAGAGACCCTCCCGCAGTGGACTCCACCACCTCCCAGGCATTTCCACTCCGCATGGGGGGAGATGGCCAGCTT

CAGTATTGGCCGTTTTCCTCTTCGGATTTATACAATTGGAAAAATAATAACCCTTCCTTTTCTGAAGATCCAGGTAAATT
GACGGCCTTGATTGAGTCCGTCCTCATCACCCACCAGCCCACCTGGGACGACTGTCAGCAGTTGTTGGGGACCCTGCTGA
CCGGAGAAGAAAAGCAGCGGGTGCTCCTAGAGGCTAGAAAGGCAGTCCGGGGCAATGATGGACGCCCCACTCAGTTGCCT
AATGAAGTCAATGCTGCTTTTCCCCTTGAACGCCCCGGTTGGGATTACACCACTACAGAAGGTAGGAACCACCTAGTCCT
CTATCGCCAGTTGCTCTTAGCGGGTCTCCAGAACGCGGGCAGAAGTCCCACCAATTTGGCCAAGGTAAAAGGGATAACCC
AGGGACCTAATGAGTCTCCCTCAGCCTTTTTAGAGAGACTCAAAGAGGCCTATCGCAGGTACACTCCTTATGACCCTGAG
GACCCAGGGCAAGAAACCAATGTGTCTATGTCATTCATCTGGCAGTCTGCCCCGGATATCGGGCGAAAGTTAGAGCGGTT
AGAAGATTTAAAGAGCAAGACCTTAGGAGACTTAGTGAGGGAAGCTGAAAAGATCTTTAATAAGCGAGAAACCCCGGAAG
AAAGAGAGGAACGTATCAAGAGAGAAACAGAGGAAAAAGAAGAACGCCGTAGGGCAGAGGATGAGCAGAGAGAGAAAGAA
AGGGACCGCAGAAGACATAGAGAGATGAGCAAGCTCTTGGCCACTGTAGTTATTGGTCAGAGACAGGATAGACAGGGGGG
AGAGCGGAGGAGGCCCCAACTTGATAAGGACCAATGCGCCTACTGCAAAAAAAAGGGACACTGGGCTAAGGACTGCCCAA
AGAAGCCACGAGGGCCCCGAGGACTGAGGCCCCAGACCTCCCTCCTAACCTTAGGTGACTAGGGAGGTCAGGGTCAGGAG
CCCCCCCCTGAACCCAGGATAACCCTCAAAGTCGGGGGGCAACCCGTCACCTTCCTGGTAGATACTGGGGCCCAACACTC
CGTGCTGACCCAAAATCCTGGACCCCTAAGTGACAAGGCTGCCTGGGTCCAAGGGGCTACTGGAGGAAAGCGGTATCGCT
GGACCACGGATCGCAAAGTACATCTAGCTACCGGTAAGGTCACCCACTCTTTCCTCCATGTACCAGACTGCCCCTATCCT
CTGCTAGGAAGAGACTTGCTGACTAAACTAAAAGCCCAAATCCACTTCGAGGGATCAGGAGCTCAGGTTATGGGACCAAT
GGGACTGCCCCTGCAAGTGCTGACCCTAAACATAGAAGATGAGTATCGGCTACATGAGACCTCAAAAGAGCCGGATGTTT
CTCTAGGGTCCACCTGGCTTTCTGATTTTCCCCAGGTCTGGGCGGAAACCGGGGGCATGGGACTGGCAGTTCGCCAAGCT
CCTCTGATCATACCTCTGAAGGCAACCTCTACCCCCGTGTCCATAAAACAATACCCCATGTCACAAGAAGCCAGACTGGG
GATCAAGCCCCACATACAGAGACTGTTGGACCAGGGAATACTGGTACCCTGCCAGTCCCCCTGGAACACGCCCCTGCTAC
CCGTTAAGAAACCAGGGACTAATGATTATAGGCCTGTCCAGGATCTGAGAGAAGTCAACAAGCGGGTGGAAGACATCCAC
CCCACCGTGCCCAACCCTTACAACCTCTTGAGCGGGCTCCCACCGTCCCACCAGTGGTACACTGTGCTTGATTTAAAGGA
TGCCTTTTTCTGCCTGAGACTCCACCCCACCAGCCAGCCTCTCTTCGCCTTTGAGTGGAGAGATCCAGAGATGGGAATCT
CAGGACAATTGACCTGGACCAGACTCCCACAGGGTTTCAAAAACAGTCCCACCCTGTTTGATGAGGCACTGCACAGAGAC
CTAGCAGACTTCCGGATCCAGCACCCAGACTTGATCCTGCTACAGTACGTGGATGACTTACTGCTGGCCGCCACTTCTGA
GCTCGACTGCCAACAAGGTACTCGGGCCCTGTTACAAACCCTAGGGAACCTCGGGTATCGGGCCTCAGCCAAGAAAGCCC
AAATTTGCCAGAAACAGGTCAAGTATCTGGGGTATCTTCTAAAAGAGGGTCAGAGATGGCTGACTGAGGCCAGAAAAGAG
ACTGTGATGGGGCAGCCTACTCCGAAGACCCCTCGACAACTAAGGGAGTTCCTAGGGACGGCAGGCTTCTGTCGCCTCTG
GATCCCTGGGTTTGCAGAAATGGCAGCCCCCTTGTACCCTCTCACCAAAACGGGGACTCTGTTTAATTGGGGCCCAGACC
AGCAAAAGGCCTATCAAGAAATCAAACAGGCTCTTCTAACTGCCCCAGCCCTGGGATTGCCAGATTTGACTAAGCCCTTT
GAACTCTTTGTTGACGAGAAGCAGGGCTACGCCAAAGGCGTCCTAACGCAAAAACTGGGACCTTGGCGTCGGCCGGTGGC
CTACCTGTCCAAAAAGCTAGACCCAGTGGCAGCTGGGTGGCCCCCTTGCCTACGGATGGTAGCAGCCATTGCCGTTCTGA
CAAAAGATGCAGGCAAGCTAACCATGGGACAGCCGCTAGTCATCCTGGCCCCCCATGCAGTAGAGGCACTAGTCAAGCAA
CCCCCTGACCGCTGGCTATCCAACGCCCGCATGACCCACTATCAGGCGATGCTCCTAGATACGGACCGGGTCCAGTTCGG
ACCGGTGGTAGCCCTAAACCCGGCTACGTTGCTCCCCCTACCGGGGAAAGAGCCTCACCATGACTGCCTCGAGATCTTGG
CCGAGACACACGGAACCAGACCAGACCTCACGGACCAGCCCCTCCCAGACGCCGACCACACCTGGTATACAGATGGAAGC
AGCTTCCTGCAAGAGGGACAACGTAGGGCTGGAGCAGCGGTGACCACCGAGACCGAGGTAATCTGGGCCAAGGCGTTGCC
AGCCGGGACATCCGCCCAGCGAGCTGAACTAATAGCACTCACCCAGGCCCTAAAGATGGCAGAAGGTAAGAAGCTAAATG
TTTATACTGATAGCCGCTATGCCTTTGCTACCGCCCATATCCATGGAGAAATATATAGGAGACGTGGGTTGCTCACCTCA
GAAGGCAAGGAGATCAAGAACAAGGGCGAAATCTTGGCCTTACTGAAAGCTCTCTTTCTGCCCAAAAGACTCAGTATAAT
TCACTGCCCAGGACATCAGAAAGGCAATAGTGCTGAAGCTAAAGGCAACCGAATGGCGGACCAGGCAGCCCGGGAAGCGG
CCATGGGGACTGACACAAAGGCCTCCTCACTTCTCATAGAGACCTCAACCCCGTACACTCCAGACTTCTTCCATTATACT
GAGACAGATATAAAGAACCTACAAGAGTTGGGAGCCACATATGATAGAGAGAAAAAATATTGGGTCCTGCAAGGTAAACC
TGTGATGCCTGACCAGTTCACCTTTAAATTATTAGACTTCCTTCACCAGCTCACCCACCTTAGCTATCAGAAGATGAGGG
CACTTCTAGACAGGAAAGAAAGCCCCTATTACATGCTAAATAAAGATAAGATCCTCCACGAGGTGGCGGAATCATGCCAA
GCCTGTGTCCAAGTAAATGCCAGTAAGACTAAGATCAGGGCCGGAACACGAGTAAGAGGACATCGACCAGGCACCCATTG
GGAAATTGACTTTACTGAAGTGAAGCCCGGACTGTATGGGTACAAGTATCTCCTGGTATTCGTGGACACGTTCTCTGGCT
GGGTTGAAGCCTTCCCAACCAAACATGAGACTGCCAAAATAGTGACCAAGAAACTTCTGGAAGAAATATTTCCAAGGTTT
GGAATGCCCCAAGTGTTGGGGACTGATAATGGGCCTGCCTTCGTCTCCCAGGTAAGTCAGTCGGTGGCCAAGCTACTGGG
GATTGATTGGAAACTACATTGTGCTTACAGACCCCAGAGTTCAGGTCAGGTAGAAAGAATGAATAGGACAATCAAGGAGA
CTTTGACCAAATTAACGCTTGCAACTGGCACTAGAGACTGGGTACTCCTACTTCCCTTAGCCCTCTACCGAGCCCGCAAC
ACTCCGGGCCCCCATGGACTCACTCCGTATGAAATCCTGTATGGGGCGCCCCCGCCCCTTGTTAATTTCCATGATCCTGA
AATGTCAAAGTTTACTAATAGCCCCTCTCTCCAAGCTCACTTACAGGCCCTCCAAGCAGTACAACGAGAGGTCTGGAAGC
CACTGGCCGCTGCCTATCAGGACCAGCTGGACCAGCCAGTGATACCACACCCCTTCCGTGTCGGCGACACCGTGTGGGTA
CGCCGGCACCAGACTAAGAACTTGGAACCTCGCTGGAAAGGACCCTACACCGTCCTGCTGACCACCCCCACCGCTCTCAA
AGTAGACGGCATCGCTGCGTGGATCCACGCCGCTCACGTAAAAGCGGCGACAACCCCTCCGGCCGGAACAGCATCAGGAC
CGACATGGAAGGTCCAGCGTTCTCAAAACCCCTTAAAGATAAGATTAACCCGTGGGGCCCCCTAATAATCCTGGGGATCT

TAATAAGAGCAGGAGTATCAGTACAACATGACAGCCCTCATCAGGTCTTCAATGTTACTTGGAGAGTTACCAACTTAATG
ACAGGACAAACAGCTAATGCTACCTCCCTCCTGGGGACAATGACCGATGCCTTTCCTAAACTGTACTTTGACTTGTGCGA
TTTAATAGGGGACGACTGGGATGAGACTGGACTCGGGTGTCGCACTCCCGGGGGAAGAAAAAGGGCAAGAACATTTGACT
TCTATGTTTGCCCCGGGCATACTGTACCAACAGGGTGTGGAGGGCCGAGAGAGGGCTACTGTGGCAAATGGGGCTGTGAG
ACCACTGGACAGGCATACTGGAAGCCATCATCATCATGGGACCTAATTTCCCTTAAGCGAGGAAACACCCCTCGGAATCA
GGGCCCCTGTTATGATTCCTCAGCGGTCTCCAGTGACATCAAGGGCGCCACACCGGGGGGTCGATGCAATCCCCTAGTCC
TGGAATTCACTGACGCGGGCAAAAAGGCCAGCTGGGATGGCCCCAAAGTATGGGGACTAAGACTGTACCGATCCACAGGG
ACCGACCCGGTGACCCGGTTCTCTTTGACCCGCCAGGTCCTCAATATAGGGCCCCGCGTCCCCATTGGGCCTAATCCCGT
GATCACTGACCAGTTACCCCCCTCCCGACCCGTGCAGATCATGCTCCCCAGGCCTCCTCAGCCTCCTCCTCCAGGCGCAG
CCTCTATAGTCCCTGAGACTGCCCCACCTTCTCAACAACCTGGGACGGGAGACAGGCTGCTAAACCTGGTAGATGGAGCC
TACCAAGCTCTCAACCTCACCAGTCCTGACAAAACCCAAGAGTGCTGGTTGTGTCTGGTAGCGGGACCCCCCTACTACGA
AGGGGTTGCCGTCCTAGGTACTTATTCCAACCATACCTCTGCCCCAGCTAACTGCTCCGTGGCCTCCCAACACAAGCTGA
CCCTGTCCGAAGTGACCGGACAGGGACTCTGCGTAGGAGCAGTTCCCAAAACCCATCAGGCCCTGTGTAATACCACCCAG
AAGACGAGCGACGGGTCCTACTATCTGGCTGCTCCCGCCGGGACCATTTGGGCTTGCAACACCGGGCTCACTCCCTGCCT
ATCTACCACTGTACTCGACCTCACCACCGATTACTGTGTCCTGGTTGAGCTCTGGCCAAAGGTGACCTACCACTCCCCTG
GTTATGTTTATGGCCAGTTTGAGAAAAAAACCAAATATAAAAGAGAGCCGGTGTCATTAACTCTGGCCCTGCTGTTGGGA
GGACTTACTATGGGCGGCATAGCTGCAGGAGTAGGAACCGGGACTACAGCCCTAGTGGCCACCAAACAATTCGAGCAGCT
CCAGGCAGCCATACATACAGACCTTGGGGCTTTAGAAAAGTCAGTCAGTGCCCTAGAAAAGTCTCTGACCTCGTTGTCTG
AGGTGGTCCTACAGAACCGGAGGGGATTAGATCTACTGTTCCTAAAAGAAGGAGGATTATGTGCTGCCCTAAAAGAAGAA
TGCTGTTTCTACGCGGACCACACTGGCGTAGTAAGAGATAGCATGGCAAAGCTAAGAGAAAGGTTGAACCAGAGACAAAA
ATTGTTCGAATCAGGACAAGGGTGGTTTGAGGGACTGTTTAACAGGTCCCCATGGTTCACGACCTTAATATCCACCATTA
TGGGCCCCTTGATAATACTTTTATTAATCCTACTCTTCGGACCCTGTATTCTCAACCGCTTGGTCCAGTTTGTAAAAGAC
AGAATTTCGGTAGTGCAGGCCCTGGTTTTGACCCAACAGTATCACCAACTCAAATCAATAGATCCAGAAGAAGTGGAATC
ACGTGAATAAAAGATTTTATTCAGTTTCCAGAAAGAGGGGGGAATGAAAGACCCCACCATCAGGCTTAGCAAGCTAGCTG
CAGTAACGCCATTTTGCAAGGCATGAAAAAGTACCAGAGCTGAGTTCTCAAAAGTTACAAGAAAGTTCAGTTAAAGATTA
ACAGTTAAAGATTAAGGCTGAATAATACTGGGACAGGGGCCAAATATCGGTGGTCAAGCACCTGGGCCCCGGCTCAGGGC
CAAGAACAGATGGCTCTCAGACGTCAGTGTTAGCAGAACTAGCTTCACTGATTTAGAAAAATAGAGGTGCACAGTGCTCT
GGCCACTCCTTGAACCTGTGTGTCTGCCAATGTTCTGACCAGGTGTGTGCCCATTGTTGAACCTTCATTAGACCCTTTCC
TCGTACCCCTCCCATACCCATTTCTTGAAAATAGACATTGTTTAGAACTAAAAAGTCCCACCTCAGTTTCCCCAAATGAC
CGAGAAATACCCCAAGCCTTATTCGAACTAACCAACCAGCTCGCTTCTCGCTTCTGTAACCGCGCTTTTTGCTCCCCAGC
CCCAGCCCTATAAAAAGGGTAAAAACTCCACACTCGGCGCGCCAGTCCTCCGATAGACTGCGTCGCCCGGGTACCCGTGT
TCTCAATAAAGCCTCTTGCTGTTTGCATCCGAATCGTGGTCTCGCTGGTCCTTGAGAGGGTCTCCTCAGATTGATTGACT
ACCCACGTCGGGGGTCTTTCA
>Pmv17
TGAAAGACCCCACCATCAGGCTTAGCAAGCTAGCTGCAGTAACGCCATTTTGCAAGGCATGAAAAAGTACCAGAGCTGAG
TTCTCAAAAGTTACAAGAAAGTTCAGTTAAAGATTAACAGTTAAAGATTAAGGCTGAATAATACTGGGACAGGGGCCAAA
TATCGGTGGTCAAGCACCTGGGCCCCGGCTCAGGGCCAAGAACAGATGGCTCTCAGACGTCAGTGTTAGCAGAACTAGCT
TCACTGATTTAGAAAAATAGAGGTGCACAGTGCTCTGGCCACTCCTTGAACCTGTGTGTCTGCCAATGTTCTGACCAGGT
GTGTGCCCATTGTTGAACCTTCATTAGACCCTTTCCTCGTACCCCTCCCATACCCATTTCTTGAAAATAGACATTGTTTA
GAACTAAAAAGTCCCACCTCAGTTTCCCCAAATGACCGAGAAATACCCCAAGCCTTATTCGAACTAACCAACCAGCTCGC
TTCTCGCTTCTGTAACCGCGCTTTTTGCTCCCCAGCCCCAGCCCTATAAAAAGGGTAAAAACTCCACACTCGGCGCGCCA
GTCCTCCGATAGACTGCGTCGCCCGGGTACCCGTGTTCCCAATAAAGCCTCTTGCTGTTTGCATCCGAATCGTGGTCTCG
CTGGTCCTTGAGAGGGTCTCCTCAGATTGATTGACTACCCACGTCGGGGGTCTTTCATTTGGAGGTTCCACCGAGATTTG
GAGACCCCTGCCCAGGGACCACCGACCCCCCGCCGGGAGGTAAGCTGGCCAGCGGTCGTTTCGTGTCTGTCTCTGTCTTC
GTGCGTGTTTGTGCCGGCATCCAATGTTTGCGCCTGCGTCTGTACTAGTTAGCTAACTAGATCTGTATCTGGCGGTTCCG
CGGAAGAACTGACGAGTTCGTATTCCCGGCCGCAGCCCCTGGGAGACGTCCCAGCGGCCTCGGGGGCCCGTTTTGTGGCC
CATTCTGTATCAGTTAACCTACCCGAGTCGGACTTTTTGGAGCTCCGCCACTGTACGTGGCTTTGTTGGGGGACGAGAGA
CAGAGACACTTCCCGCCCCCGTCTGAATTTTTGCTTTCGGTTTTACGCCGAAGCCGCGCCGCGCGTCTGATTTGTTTGTT
GTTCTTTTGTTCTTCGTTAGTTTTCTTCTGTCTTTAAGTGTTTTCGAGATCATGGGACAGACCGTAACTACCCCTCTGAG
TTTAACCTTGCAGCACTGGGGAGATGTCCAGCGCATTGCATCCAACCAGTCTGTGGATGTCAGGAAGAGGCGCTGGATTA
CCTTCTGTTCCGCTGAATGGCCAACTTTCAATGTGGGATGGCCTCAGGATGGTACTTTCAATTTAAGTATTATCTCTCAG
GTTAAGTCTAGAGTGTTTTGTCCTGGTCCCCACGGACACCCGGATCAGGTCCCATATATCGTCACCTGGGAGGCACTTGC
CTATGACCCCCCTCCGTGGGTCAAACCGTTTGTGTCTCCTAAACTTCCTCCCTTGCCGACAGCTCCCGTCCTCCCGCCCG
GTCCTTCTGCGCAACCTCCGTCCCGATCTGCCCTTTACCCTGCCCTTACCCCCTCTATAAAGTCCAAACCTCCTAAGCCC
CAGGTTCTCCCTGATAGCGGCGGACCCCTCATTGACCTTCTCACAGAGGACCCCCCGCCGTACAGAGCACAACCCTCCT
CCTCTGCCAGGGAGAACGACGAAGAAGAGGCGGCCACCACCTCCGAGGTTTCCCCCCCCCCTTCTCCCATGGTGTCTCGA
CTGCGGGGAAGGAGAGACCCTCCCGCAGTGGACTCCACCACCTCCCAGGCATTTCCACTCCGCATGGGGGGAGATGGCCA

GCTTCAGTATTGGCCGTTTTCCTCTTCGGATTTATACAATTGGAAAAATAATAACCCTTCCTTTTCTGAAGATCCAGGTA
AATTGACGGCCTTGATTGAGTCCGTCCTCATCACCCACCAGCCCACCTGGGACGACTGTCAGCAGTTGTTGGGGACCCTG
CTGACCGGAGAAGAAAAGCAGCGGGTGCTCCTAGAGGCTAAAAAGGCAGTCCGGGGCAATGATGGACGCCCCACTCAGTT
GCCTAATGAAGTCAATGCTGCTTTTCCCCTTGAACGCCCCGGTTGGGATTACACCACTACAGAAGGTAGGAACCACCTAG
TCCTCTATCGCCAGTTGCTCTTAGCGGGTCTCCAGAACGCGGGCAGAAGTCCCACCAATTTGGCCAAGGTAAAAGGGATA
ACCCAGGGACCTAATGAGTCTCCCTCAGCCTTTTTAGAGAGACTCAAAGAGGCCTATCGCAGGTACACTCCTTATGACCC
TGAGGACCCAGGGCAAGAAACCAATGTGTCTATGTCATTCATCTGGCAGTCTGCCCCGGATATCGGGCGAAAGTTAGAGC
GGTTAGAAGATTTAAAGAGCAAGACCTTAGGAGACTTAGTGAGGGAAGCTGAAAAGATCTTTAATAAGCGAGAAACCCCG
GAAGAAAGAGAGGAACGTATCAAGAGAAAAACAGAGGAAAAAGAAGAACGCCGTAGGGCAGAGGATGAGCAGAGAGAGAA
AAAAAGGGACCGCAAAAGACATAGAGAGATGAGCAAGCTCTTGGCCACTGTAGTTATTGGTCAGAGACAGGATAGACAGG
GGGGAGAGCGGAGGAGGCCCCAACTTGATAAGGACCAATGCGCCTACTGCAAAGAAAAGGGACACTGGGCTAAGGACTGC
CCAAAGAAGCCACGAGGGCCCCGAGGACTGAGGCCCCAGACCTCCCTCCTAACCTTAGGTGACTAGGGAGGTCAGGGTCA
GGAGCCCCCCCCTGAACCCAGGATAACCCTCAAAGTCGGGGGGCAACCCGTCACCTTCCTGGTAGATACTGGGGCCCAAC
ACTCCGTGCTGACCCAAAATCCTGGACCCCTAAGTGACAAGGCTGCCTGGGTCCAAGGGGCTACTGGAGGAAAGCGGTAT
CGCTGGACCACGGATCGCAAAGTACATCTGGGCCAAGGCGTTGCCAGCCGGGACATCCGCCCAGCGAGCTGAACTAATAG
CACTCACCCAGGCCCTAAAGATGGCAGAAGGTAAGAAGCTAAACGTTTATACTGATAGCCGCTATGCCTTTGCTACCGCC
CATGTCCATGGAGAAATATATAGGAGACGTGGGTTGCTCACCTCAGAAGGCAAGGAGATCAAAAACAAGGGCGAAATCTT
GGCCTTACTGAAAGCTCTCTTTTTGCCCAAAAGACTCAGTATAATTCACTGCCCAGGACATCAGAAAGGCAATAGTGCTG
AAGCTAAAGGCAACCAAATGGCGGACCAGGCAGCCCGGGAAGCAGCCATGGGGACTGACACAAAGGCCTCCTCACTTCTC
ATAGAGACCTCAACCCCGTACACTCCAGACTTCTTCCATTATACTGAGACAGATATAAAGAACCTACAAGAGTTGGGAGC
CACATATGATAGAGAGAAAAAATATTGGGTCCTGCAAGGTAAACCTGTGATGCCTGACCAGTTCACCTTTGAATTATTAG
ACTTCCTTCACCAGCTCACCCACCTTAGCTATCAGAAGATGAGGGCACTTCTAGACAGGAAAGAAAGCCCCTATTACATG
CTAAATAAAGATAAGATCCTCCACGAGGTGGCGGAATCATGCCAAGCCTGTGTCCAAGTAAATGCCAGTAAGACTAAGAT
CAGGGCCGGAACACGAGTAAGAGGACATCGACCAGGCACCCATTGGGAAATTGACTTTACTGAAGTAAAGCCCGGACTGT
ATGGGTACAAGTATCTCCTGGTATTCGTGGACACGTTCTCTGGCTGGGTTGAAGCCTTCCCAACCAAACATGAGACTGCC
AAAATAGTGACCAAGAAACTTCTGAAAGAAATATTTCCAAGGTTTGGAATGCCCCAAGTGTTGGGGACTGATAATGGGCC
TGCCTTCGTCTCCCAGGTAAGTCAGTCGGTGGCCAAGCTACTGGGGATTGATTGGAAACTACATTGTGCTTACAGACCCC
AGAGTTCAGGTCAGGTAGAAAGAATGAATAGGACAATCAAGGAGACTTTGACCAAATTAACGCTTGCAACTGGCACTAGA
GACTGGGTACTCCTACTTCCCTTAGCCCTCTACCGAGCCCGCAACACTCCGGGCCCCCATGGACTCACTCCGTATGAAAT
CCTGTATGGGGCGCCCCCGCCCCTTGTTAATTTCCATGATCCTGAAATGTCAAAGTTTACTAATAGCCCCTCTCTCCAAG
CTCACTTACAGGCCCTCCAAGCAGTACAACGAGAGGTCTGGAAGCCACTGGCCGCTGCCTATCAGGACCAGCAGGACCAG
CCAGTGATACCACACCCCTTCCGTGTAGGCGACACCGTGTGGGTACGCCGGCACCAGACTAAGAACTTGGAACCTCGCTG
GAAAGGACCCTACACCGTCCTGCTGACCACCCCCACCGCTCTCAAAGTAGACGGCATCGCTGCGTGGATCCACGCCGCTC
ACGTAAAAGCGGCGACAACCCCTCCGGCCGGAACAGCATCAGGACCGACATGGAAGGTCCAGCGTTCTCAAAACCCCTTA
AAGATAAGATTAACCCGTGGGGCCCCCTAATAGTCCTGGGGATCTTAATAAGGGCAGGAGTATCAGTACAACATGACAGC
CCTCATCAGGTCTTCAATGTTACTTGGAGAGTTACCAACTTAATGACAGGACAAACAGCTAATGCTACCTCCCTCCTGGG
GACAATGACCGATGCCTTTCCTAAACTGTACTTTGACTTGTGCGATTTAATAGGGGACGATTGGGATGAGACTGGACTCG
GGTGTCGCACTCCCGGGGAAAGAAAAAGGGCAAGAACATTTGACTTCTATGTTTGCCCCGGGCATACTGTACCAACAGGG
TGTGGAGGGCCGAGAGAGGGCTACTGTGGCAAATGGGGCTGTGAGACCACTGGACAGGCATACTGGAAGCCATCATCATC
ATGGGACCTAATTTCCCTTAAGCGAGAAAACACCCCTCGGAATCAGGGCCCCTGTTATGATTCCTCAGCGGTCTCCAGTG
ACATCAAGGGCGCCACACCGGGGGGTCGATGCAATCCCCTAGTCCTGGAATTCACTGACGCGGGCAAAAAGGCCAGCTGG
GATGGCCCCAAAGTATGGGGACTAAGACTGTACCGATCCACAGGGACCGACCCGGTGACCCGGTTCTCTTTGACCCGCCA
GGTCCTCAATATAGGGCCCCGCGTCCCCATTGGGCCTAATCCCGTGATCACTGACCAGTTACCCCCCTCCCGACCCGTGC
AGATCATGCTCCCCAGGCCTCCTCAGCCTCCTCCTCCAGGCGCAGCCTCTATAGTCCCTGAGACTGCCCCACCTTCTCAA
CAACCTGGGACGGGAGACAGGCTGCTAAACCTGGTAGATGGAGCCTACCAAGCTCTCAACCTCACCAGTCCTGACAAAAC
CCAAGAGTGCTGGTTGTGTCTGGTAGCGGGACCCCCCTACTACGAAGGGGTTGCCGTCCTAGGTACTTATTCCAACCATA
CCTCTGCCCCAGCTAACTGCTCCGTGGCCTCCCAACACAAGCTGACCCTGTCCGAAGTGACCGGACAGGGACTCTGCGTA
GGAGCAGTTCCCAAAACCCATCAGGCCCTGTGTAATACCACCCAGAAGGCGAGCGACGGGTCCTACTATCTGGCTGCTCC
CGCCGGGACCATTTGGGCTTGCAACACCGGGCTCACTCCCTGCCTATCTACCACTGTACTCGACCTCACCACCGATTACT
GTGTCCTGGTTGAGCTCTGGCCAAAGGTGACCTACCACTCCCCTGGTTATGTTTATGGCCAGTTTGAGAGAAAAACCAAA
TATAAAAGAGAGCCGGTGTCATTAACTCTGGCCCTGCTGTTGGGAGGACTTACTATGGGCGGCATAGCTGCAGGAGTAGG
AACCGGGACTACAGCCCTAGTGGCCACCAAACAATTCGAGCAGCTCCAGGCAGCCATACATACAGACCTTGGGGCTTTAG
AAAAGTCAGTCAGTGCCCTAGAAAAGTCTCTGACCTCGTTGTCTGAGGTGGTCCTACAGAACCGGAGGGGATTAGATCTA
CTGTTCCTAAAAGAAGGAGGATTATGTGCTGCCCTAAAAGAAGAATGCTGTTTCTACGCGGACCACACTGGCGTAGTAAG
AGATAGCATGGCAAAGCTAAGAGAAAGGTTGAACCAGAGACAAAAATTGTTCGAATCAGGACAAGGGTGGTTTGAGGGAC
TGTTTAACAGGTCCCCATGGTTCACGACCTTAATATCCACCATTATGGGCCCCTTAATAATACTTTTATTAATCCTACTC
TTCGGACCCTGTATTCTCAACCGCTTGGTCCAGTTTGTAAAAGACAGAATTTCGGTAGTGCAAGCCCTAGTTTTGACCCA

ACAGTATCACCAACTCAAATCAATAGATCCAGAAAAAGTGGAATCACGTGAATAAAAGATTTTATTCAGTTTCCAGAAAG
AGGGGGGAATGAAAGACCCCACCATCAGGCTTAGCAAGCTAGCTGCAGTAACGCCATTTTGCAAGGCATGAAAAAGTACC
AGAGCTGAGTTCTCAAAAGTTACAAGAAAGTTCAGTTAAAGATTAACAGTTAAAGATTAAGGCTGAATAATACTGGGACA
GGGGCCAAATATCGGTGGTCAAGCACCTGGGCCCCGGCTCAGGGCCAAGAACAGATGGCTCTCAGACGTCAGTGTTAGCA
GAACTAGCTTCACTGATTTAGAAAAATAGAGGTGCACAGTGCTCTGGCCACTCCTTGAACCTGTGTGTCTGCCAATGTTC
TGACCAGGTGTGTGCCCATTGTTGAACCTTCATTAGACCCTTTCCTCGTACCCCTCCCATACCCATTTCTTGAAAATAGA
CATTGTTTAGAACTAAAAAGTCCCACCTCAGTTTCCCCAAATGACCGAGAAATACCCCAAGCCTTATTCGAACTAACCAA
CCAGCTCGCTTCTCGCTTCTGTAACCGCGCTTTTTGCTCCCCAGCCCCAGCCCTATAAAAAGGGTAAAAACTCCACACTC
GGCGCGCCAGTCCTCCGATAGACTGCGTCGCCCGGGTACCCGTGTTCCCAATAAAGCCTCTTGCTGTTTGCATCCGAATC
GTGGTCTCGCTGGTCCTTGAGAGGGTCTCCTCAGATTGATTGACTACCCACGTCGGGGGTCTTTCA
>Pmv18
TGAAAGACCCCACCATCAGGCTTAGCAAGCTAGCTGCAGTAACGCCATTTTGCAAGGCATGAAAAAGTACCAGAGCTGAG
TTCTCAAAAGTTACAAGAAAGTTCAGTTAAAGATTAACAGTTAAAGATTAAGGCTGAATAATACTGGGACAGGGGCCAAA
TATCGGTGGTCAAGCACCTGGGCCCCGGCTCAGGGCCAAGAACAGATGGCTCTCAGACGTCAGTGTTAGCAGAACTAGCT
TCACTGATTTAGAAAAATAGAGGTGCACAGTGCTCTGGCCACTCCTTGAACCTGTGTGTCTGCCAATGTTCTGACCAGGT
GTGTGCCCATTGTTGAACCTTCATTAGACCCTTTCCTCGTACCCCTCCCATACCCATTTCTTGAAAATAGACATTGTTTA
AAACTAAAAAGTCCCACCTCAGTTTCCCCAAATGACCGAGAAATACCCCAAGCCTTATTCGAACTAACCAACCAGCTCGC
TTCTCGCTTCTGTAACCGCGCTTTTTGCTCCCCAGCCCCAGCCCTATAAAAAGGGTAAAAACTCCACACTCGGCGCGCCA
GTCCTCCAATAGACTGCGTCGCCCGGGTACCCGTGTTCTCAATAAAGCCTCTTGCTGTTTGCATCCGAATCGTGGTCTCG
CTGGTCCTTGAGAGGGTCTCCTCAGATTGATTGACTACCCACCTCGGGGGTCTTTCATTTGGAGGTTCCACCGAGATTTG
GAGACCCCTGCCCAGGGACCACCGACCCCCCGCCGGGAGGTAAGCTGGCCAGCGGTCGTTTCGTGTCTGTCTCTGTCTTC
GTGCGTGTTTGTGCCGGCATCCAATGTTTGCGCCTGCGTCTGTACTAGTTAGCTAACTAGATCTGTATCTGGCGGTTCCG
CGGAAGAACTGACGAGTTCGTATTCCCGGCCGCAGCCCCTGGGAGACGTCCCAGCGGCCTCGGGGGCCCGTTTTGTGGCC
CATTCTGTATCAGTTAACCTACCCGAGTCGGACTTTTTGGAGCTCCGCCACTGTACGTGGCTTTGTTGGGGGACGAGAGA
CAGAGACACTTCCCGCCCCCGTCTGAATTTTTGCTTTCGGTTTTACGCCGAAGCCGCGCCGCGCGTCTGACTTGTTTGTT
GTTCTTTTGTTCTTCGTTAGTTTTCTTCTGTCTTTAAGTGTTTTCGAGATCATGGGACAGACCGTAACTACCCCTCTGAG
TTTAACCTTGCAGCACTGGGGAGATGTCCAGCGCATTGCATCCAACCAGTCTGTGGATGTCAGGAAGAGGCGCTGGATTA
CCTTCTGTTCCGCTGAATGGCCAACTTTCAATGTGGGATGGCCTCAGGATGGTACTTTCAATTTAAGTATTATCTCTCAG
GTTAAGTCTAGAGTGTTTTGTCCTGGTCCCCACGGACACCCGGATCAGGTCCCATATATCGTCACCTGGGAGGCACTTGC
CTATGACCCCCCTCCGTGGGTCAAACCGTTTGTGTCTCCTAAACTTCCTCCCTTGCCGACAGCTCCCGTCCTCCCGCCCG
GTCCTTCTGCGCAACCTCCGTCCCGATCTGCCCTTTACCCTGCCCTTACCCCCTCTATAAAGTCCAAACCTCCTAAGCCC
CAGGTTCTCCCTGATAGCGGCGGACCCCTCATTGACCTTCTCACAGAGGACCCCCCGCCGTACAGAGCACAACCCTCCTC
CTCTGCCAGGGAGAACGACGAAGAAGAGGCGGCCACCACCTCCGAGGTTTCCCCCCCTTCTCCCATGGTGTCTCGACTGC
GGGGAAGGAGAGACCCTCCCGCAGTGGACTCCACCACCTCCCAGGCATTTCCACTCCGCATGGGGGGAGATGGCCAGCTT
CAGTATTGGCCGTTTTCCTCTTCGGATTTATACAATTGGAAAAATAATAACCCTTCCTTTTCTGAAGATCCAGGTAAATT
GACGGCCTTGATTGAGTCCGTCCTCATCACCCACCAGCCCACCTGGGACGACTGTCAGCAGTTGTTGGGGACCCTGCTGA
CCGGAGAAGAAAAGCAGCGGGTGCTCCTAGAGGCTAGAAAGGCAGTCCGGGGCAATGATGGACGCCCCACTCAGTTGCCT
AATGAAGTCAATGCTGCTTTTCCCCTTGAACGCCCCGGTTGGGATTACACCACTACAGAAGGTAGGAACCACCTAGTCCT
CTATCGCCAGTTGCTCTTAGCGGGTCTCCAGAACGCGGGCAGAAGTCCCACCAATTTGGCCAAGGTAAAAGGGATAACCC
AGGGACCTAATGAGTCTCCCTCAGCCTTTTTAGAGAGACTCAAAGAGGCCTATCGCAGGTACACTCCTTATGACCCTGAG
GACCCAGGGCAAGAAACCAATGTGTCTATGTCATTCATCTGGCAGTCTGCCCCGGATATCGGGCGAAAGTTAGAGCGGTT
AGAAGATTTAAAGAGCAAGACCTTAGGAGACTTAGTGAGGGAAGCTGAAAAGATCTTTAATAAGCGAGAAACCCCGGAAG
AAAGAGAGGAACGTATCAAGAGAGAAACAGAGGAAAAAGAAGAACGCCGTAGGGCAGAGGATGAGCAGAGAGAGAAAGAA
AGGGACCGCAGAAGACATAGAGAGATGAGCAAGCTCTTGGCCACTGTAGTTATTGGTCAGAGACAGGATAGACAGGGGGG
AGAGCGGAGGAGGCCCCAACTTGATAAGGACCAATGCGCCTACTGCAAAGAAAAGGGACACTGGGCTAAGGACTGCCCAA
AGAAGCCACGAGGGCCCCGAGGACTGAGGCCCCAGACCTCCCTCCTAACCTTAGGTGACTAGGGAGGTCAGGGTCAGGAG
CCCCCCCCTGAACCCAGGATAACCCTCAAAGTCGGGGGGCAACCCGTCACCTTCCTGGTAGATACTGGGGCCCAACACTC
CGTGCTGACCCAAAATCCTGGACCCCTAAGTGACAAGGCTGCCTGGGTCCAAGGGGCTACTGGAGGAAAGCGGTATCGCT
GGACCACGGATCGCAAAGTACATCTAGCTACCGGTAAGGTCACCCACTCTTTCCTCCATGTACCAGACTGCCCCTATCCT
CTGCTAGGAAGAGACTTGCTGACTAAACTAAAAGCCCAAATCCACTTCGAGGGATCAGGAGCTCAGGTTATGGGACCAAT
GGGACTGCCCCTGCAAGTGCTGACCCTAAACATAGAAGATGAATATCGGCTACATGAGACCTCAAAAGAGCCGGATGTTT
CTCTAGGGTCCACCTGGCTTTCTGATTTTCCCCAGGTCTGGGCGGAAACCGGGGGCATGGGACTGGCAGTTCGCCAAGCT
CCTCTGATCATACCTCTGAAGGCAACCTCTACCCCCGTGTCCATAAAACAATACCCCATGTCACAAGAAGCCAGACTGGG
GATCAAGCCCCACATACAGAGACTGTTGGACCAGGGAATACTGGTACCCTGCCAGTCCCCCTGGAACACGCCCCTGCTAC
CCGTTAAGAAACCAGGGACTAATGATTATAGGCCTGTCCAGGATCTGAGAGAAGTCAACAAGCGGGTGGAAGACATCCAC
CCCACCGTGCCCAACCCTTACAACCTCTTGAGCGGGCTCCCACCGTCCCACCAGTGGTACACTGTGCTTGATTTAAAGGA
TGCCTTTTTCTGCCTGAGACTCCACCCCACCAGTCAGCCTCTCTTCACCTTTGAGTGGAGAGATCCAGAGATGAGAATCT

CAGGACAATTGACCTGGACCAGACTCCCACAGGGTTTCAAAAACAGTCCCACCCTGTTTGATGAGGCACTGCACAGAGAC
CTAGCAGACTTCCGGATCCAGCACCCAGACTTGATCCTGCTACAGTACGTGGATGACTTACTGCTGGCCGCCACTTCTGA
GCTCGACTGCCAACAAGGTACTCGGGCCCTGTTACAAACCCTAGGGAACCTCGGGTATCGGGCCTCGGCCAAGAAAGCCC
AAATTTGCCAGAAACAGGTCAAGTATCTGGGGTATCTTCTAAAAGAGGGTCAGAGATGGCTGACTGAGGCCAGAAAAGAG
ACTGTGATGGGGCAGCCTACTCCGAAGACCCCTCGACAACTAAGGGAGTTCCTAGGGACGGCAGGCTTCTGTCGCCTCTG
GATCCCTGGGTTTGCAGAAATGGCAGCCCCCTTGTACCCTCTCACCAAAACGGGGACTCTGTTTAATTGGGGCCCAGACC
AGCAAAAGGCCTATCAAGAAATCAAACAGGCTCTTCTAACTGCCCCAGCCCTGGGATTGCCAGATTTGACTAAGCCCTTT
GAACTCTTTGTTGACGAGAAGCAGGGCTACGCCAAAGGCGTCCTAACGCAAAAACTGGGACCTTGGCGTCGGCCGGTGGC
CTACCTGTCCAAAAAGCTAGACCCAGTGGCAGCTGGGTGGCCCCCTTGCCTACGGATGGTAGCAGCCATTGCCGTTCTGA
CAAAAGATGCAGGCAAGCTAACCATGGGACAGCCGCTAGTCATCCTGGCCCCCCATGCAGTAGAGGCACTAGTCAAGCAA
CCCCCTGACCGCTGGCTATCCAACGCCCGCATGACCCACTATCAGGCGATGCTCCTAGATACGGACCGGGTCCAGTTCGG
ACCGGTGGTAGCCCTAAACCCGGCTACGTTGCTCCCCCTACCGGGGAAAGAGCCTCACCATGACTGCCTCGAGATCTTGG
CCGAGACACACGGAACCAGACCAGACCTCACGGACCAGCCCCTCCCAGACGCCGACCACACCTGGTATACAGATGGAAGC
AGCTTCCTGCAAGAGGGACAACGTAGGGCTGGAGCAGCGGTGACCACCGAGACCGAGGTAATCTGGGCCAAGGCGTTGCC
AGCCGGGACATCCGCCCAGCGAGCTGAACTAATAGCACTCACCCAGGCCCTAAAGATGGCAGAAGGTAAGAAGCTAAATG
TTTATACTGATAGCCGCTATGCCTTTGCTACCGCCCATGTCCATGGAGAAATATATAGGAGACGTGGGTTGCTCACCTCA
GAAGGCAAGGAGATCAAGAACAAGGGCGAAATCTTGGCCTTACTGAAAGCTCTCTTTCTGCCCAAAAGACTCAGTATAAT
TCACTGCCCAGGACATCAGAAAGGCAATAGTGCTGAAGCTAAAGGCAACCGAATGGCGGACCAGGCAGCCCGGGAAGCAG
CCATGGGGACTGACACAAAGGCCTCCTCACTTCTCATAGAGACCTCAACCCCGTACACTCCAGACTTCTTCCATTATACT
GAGACAGATATAAAGAACCTACAAGAGTTGGGAGCCACATATGATAGAGAGAAAAAATATTGGGTCCTGCAAGGTAAACC
TGTGATGCCTGACCAGTTCACCTTTGAATTATTAGACTTCCTTCACCAGCTCACCCACCTTAGCTATCAGAAGATGAGGG
CACTTCTAGACAGGAAAAAAAAGCCCCTATTACATGCTAAATAAAGATAAGATCCTCCACGAGGTGGCGGAATCATGCCAA
GCCTGTGTCCAAGTAAATGCCAGTAAGACTAAGATCAGGGCCGGAACACGAGTAAGAGGACATCGACCAGGCACCCATTG
GGAAATTGACTTTACTGAAGTGAAGCCCGGACTGTATGGGTACAAGTATCTCCTGGTATTCGTGGACACGTTCTCTGGCT
GGGTTGAAGCCTTCCCAACCAAACATGAGACTGCCAAAATAGTGACCAAGAAACTTCTGGAAGAAATATTTCCAAGGTTT
AGAATGCCCCAAGTGTTGGGGACTGATAATGGGCCTGCCTTCGTCTCCCAGGTAAGTCAGTCGGTGGCCAAGCTACTGGG
GATTGATTGGAAACTACATTGTGCTTACAGACCCCAGAGTTCAGGTCAGGTAAAAAGAATGAATAGGACAATCAAGGAGA
CTTTGACCAAATTAACGCTTGCAACTGGCACTAGAGACTGGGTACTCCTACTTCCCTTAGCCCTCTACCGAGCCCGCAAC
ACTCCGGGCCCCCATGGACTCACTCCGTATGAAATCCTGTATGGGGCGCCCCCGCCCCTTGTTAATTTCCATGATCCTGA
AATGTCAAAGTTTACTAATAGCCCCTCTCTCCAAGCTCACTTACAGGCCCTCCAAGCAGTACAACGAGAGGTCTGGAAGC
CACTGGCCGCTGCCTATCAGGACCAGCTGGACCAGCCAGTGATACCACACCCCTTCCGTGTCGGCGACACCGTGTGGGTA
CGCCGGCACCAGACTAAGAACTTGGAACCTCGCTGGAAAGGACCCTACACCGTCCTGCTGACCACCCCCACCGCTCTCAA
AGTAGACGGCATCGCTGCGTGGATCCACGCCGCTCACGTAAAAGCGGCGACAACCCCTCCGGCCGGAACAGCATCAGGAC
CGACATGGAAGGTCCAGCGTTCTCAAAACCCCTTAAAGATAAGATTAACCCGTGGGGCCCCCTAATAATCCTGGGGATCT
TAATAAGGGCAGGAGTATCAGTACAACATGACAGCCCTCATCAGGTCTTCAATGTTACTTGGAGAGTTACCAACTTAATG
ACAGGACAAACAGCTAATGCTACCTCCCTCCTGGGGACAATGACCGATGCCTTTCCTAAACTGTACTTTGACTTGTGCGA
TTTAATAGGGGACGACTGGGATGAGACTGGACTCGGGTGTCGCACTCCCGGGGGAAGAAAAAAGGCCAGCTGGGATGGCC
CCAAAGTATGGGGACTAAGACTGTACCGATCCACAGGGACCGACCCGGTGACCCGGTTCTCTTTGACCCGCCAGGTCCTC
AATATAGGGCCCCGCGTCCCCATTGGGCCTAATCCCGTGATCACTGACCAGTTACCCCCCTCCCGACCCGTGCAGATCAT
GCTCCCCAGGCCTCCTCAGCCTCCTCCTCCAGGCGCAGCCTCTATAGTCCCTGAGACTGCCCCACCTTCTCAACAACCTG
GGACGGGAGACAGGCTGCTAAACCTGGTAGATGGAGCCTACCAAGCTCTCAACCTCACCAGTCCTGACAAAACCCAAGAG
TGCTGGTTGTGTCTGGTAGCGGGACCCCCCTACTACGAAGGGGTTGCCGTCCTAGGTACTTATTCCAACCATACCTCTGC
CCCAGCTAACTGCTCCGTGGCCTCCCAACACAAGCTGACCCTGTCCGAAGTGACCGGACAGGGACTCTGCGTAAGAGCAG
TTCCCAAAACCCATCAGGCCCTGTGTAATACCACCCAGAAGACGAGCGACGGGTCCTACTATCTGGCTGCTCCCGCCGGG
ACCATTTGGGCCTGCAACACCGGGCTCACTCCCTGCCTATCTACCACTGTACTCGACCTCACCACCGATTACTGTGTCCT
GGTTGAGCTCTGGCCAAAGGTGACCTACCACTCCCCTGGTTATGTTTATGGCCAGTTTGAGAGAAAAACCAAATATAAAA
GAGAGCCGGTGTCATTAACTCTGGCCCTGCTGTTGGGAGGACTTACTATGGGCGGCATAGCTGCAGGAGTAGGAACCGGG
ACTACAGCCCTAGTGGCCACCAAACAATTCGAGCAGCTCCAGGCAGCCATACATACAGACCTTGGGGCTTTAAAAAAGTC
AGTCAGTGCCCTAGAAAAGTCTCTGACCTCGTTGTCTGAGGTGGTCCTACAGAACCGGAGGGGATTAGATCTACTGTTCC
TAAAAGAAGGAGGATTATGTGCTGCCCTAAAAGAAGAATGCTGTTTCTACGCGGACCACACTGGCGTAGTAAGAGATAGC
ATGGCAAAGCTAAGAGAAAGGTTGAACCAGAGACAAAAATTGTTCAAATCAGGACAAGGGTGGTTTGAGGGACTGTTTAA
CAGGTCCCCATGGTTCACGACCTTAATATCCACCATTATGGGCCCCTTAATAATACTTTTATTAATCCTACTCTTCGGAC
CCTGTATTCTCAACCGCTTGGTCCAGTTTGTAAAAGACAGAATTTCGGTAGTGCAGGCCCTGGTTTTGACCCAACAGTAT
CACCAACTCAAATCAATAGATCCAGAAGAAGTGGAATCACGTGAATAAAAGATTTTATTCAGTTTCCAGAAAGAGGGGGG
AATGAAAGACCCCACCATCAGGCTTAGCAAGCTAGCTGCAGTAACGCCATTTTGCAAGGCATGAAAAAGTACCAGAGCTG
AGTTCTCAAAAGTTACAAGAAAGTTCAGTTAAAGATTAACAGTTAAAGATTAAGGCTGAATAATACTGGGACAGGGGCCA
AATATCGGTGGTCAAGCACCTGGGCCCCGGCTCAGGGCCAAGAACAGATGGCTCTCAGACGTCAGTGTTAGCAGAACTAG

CTTCACTGATTTAGAAAAATAGAGGTGCACAGTGCTCTGGCCACTCCTTGAACCTGTGTGTCTGCCAATGTTCTGACCAG
GTGTGTGCCCATTGTTGAACCTTCATTAGACCCTTTCCTCGTACCCCTCCCATACCCATTTCTTGAAAATAGACATTGTT
TAAAACTAAAAAGTCCCACCTCAGTTTCCCCAAATGACCGAGAAATACCCCAAGCCTTATTCGAACTAACCAACCAGCTC
GCTTCTCGCTTCTGTAACCGCGCTTTTTGCTCCCCAGCCCCAGCCCTATAAAAAGGGTAAAAACTCCACACTCGGCGCGC
CAGTCCTCCAATAGACTGCGTCGCCCGGGTACCCGTGTTCTCAATAAAGCCTCTTGCTGTTTGCATCCGAATCGTGGTCT
CGCTGGTCCTTGAGAGGGTCTCCTCAGATTGATTGACTACCCACCTCGGGGGTCTTTCA
>Pmv19
TGAAAGACCCCACCATCAGGCTTAGCAAGCTAGCTGCAGTAACGCCATTTTGCAAGGCATGAAAAAGTACCAGAGCTGAG
TTCTCAAAAGTTACAAGAAAGTTCAGTTAAAGATTAACAGTTAAAGATTAAGGCTGAATAATACTGGGACAGGGGCCAAA
TATCGGTGGTCAAGCACCTGGGCCCCGGCTCAGGGCCAAGAACAGATGGCTCTCAGACGTCAGTGTTAGCAGAACTAGCT
TCACTGATTTAGAAAAATAGAGGTGCACAGTGCTCTGGCCACTCCTTGAACCTGTGTGTCTGCCAATGTTCTGACCAGGT
GTGTGCCCATTGTTGAACCTTCATTAGACTCTTTCCTCGTACCCCTCCCATACCCATTTCTTGAAAATAGACATTGTTTA
GAACTAAAAAGTCCCACCTCAGTTTCCCCAAATGACCGAGAAAATACCCCAAGCCTTATTCGAACTAACCAACCAGCTCG
CTTCTCGCTTCTGTAACCGCGCTTTTTGCTCCCCAGCCCCAGCCCTATAAAAAGGGTAAAAACTCCACACTCGGCGCGCC
AGTCCTCCGATAGACTGCGTCGCCCGGGTACCCGTGTTCTCAATAAAGCCTCTTGCTGTTTGCATCCGAATCGTGGTCTC
GCTGGTCCTTGAGAGGGTCTCCTCAGATTGATTGACTACCCACGTCGGGGGTCTTTCATTTGGAGGTTCCACCGAGATTT
GGAGACCCCTGCCCAGGGACCACCGACCCCCCGCCGGGAGGTAAGCTGGCCAGCGGTCGTTTCGTGTCTGTCTCTGTCTT
CGTGCGTGTTTGTGCCGGCATCCAATGTTTGCGCCTGCGTCTGTACTAGTTAGCTAACTAGATCTGTATCTGGCGGTTCC
GCGGAAGAACTGACGAGTTCGTATTCCCGGCCGCAGCCCCTGGGAGACGTCCCAGCGGCCTCGGGGGCCCGTTTTGTGGC
CCATTCTGTATCAGTTAACCTACCCGAGTCGGACTTTTTGGAGCTCCGCCACTGTACGTGGCTTTGTTGGGGGACGAGAG
ACAGAGACACTTCCCGCCCCCGTCTGAATTTTTGCTTTCGGTTTTACGCCGAAGCCGCGCCGCGCGTCTGATTTGTTTGT
TGTTCTTTTGTTCTTCGTTAGTTTTCTTCTGTCTTTAAGTGTTTTCGAGATCATGGGACAGACCGTAACTACCCCTCTGA
GTTTAACCTTGCAGCACTGGGGAGATGTCCAGCGCATTGCATCCAACCAGTCTGTGGATGTCAGGAAGAGGCGCTGGATT
ACCTTCTGTTCCGCTGAATGGCCAACTTTCAATGTGGGATGGCCTCAGGATGGTACTTTCAATTTAAGTATTATCTCTCA
GGTTAAGTCTAGAGTGTTTTGTCCTGGTCCCCACGGACACCCGGATCAGGTCCCATATATCGTCACCTGGGAGGCACTTG
CCTATGACCCCCCTCCGTGGGTCAAACCGTTTGTGTCTCCTAAACTTCCTCCCTTGCCGACAGCTCCCGTCCTCCCGCCC
GGTCCTTCTGCGCAACCTCCGTCCCGATCTGCCCTTTACCCTGCCCTTACCCCCTCTATAAAGTCCAAACCTCCTAAGCC
CCAGGTTCTCCCTGATAGCGGCGGACCCCTCATTGACCTTCTCACAGAGGACCCCCCGCCGTACAGAGCACAACCCTCCT
CCTCTGCCAGGGAGAACGACGAAGAAGAGGCGGCCACCACCTCCGAGGTTTCCCCCCCTTCTCCCATGGTGTCTCGACTG
CGGGGAAGGAGAGACCCTCCCGCAGTGGACTCCACCACCTCCCAGGCATTTCCACTCCGCATGGGGGGAGATGGCCAGCT
TCAGTATTGGCCGTTTTCCTCTTCGGATTTATACAATTGGAAAAATAATAACCCTTCCTTTTCTGAAGATCCAGGTAAAT
TGACGGCCTTGATTGAGTCCGTCCTCATCACCCACCAGCCCACCTGGGACGACTGTCAGCAGTTGTTGGGGACCCTGCTG
ACCGGAGAAGAAAAGCAGCGGGTGCTCCTAGAGGCTAGAAAGGCAGTCCGGGGCAATGATGGACGCCCCACTCAGTTGCC
TAATGAAGTCAATGCTGCTTTTCCCCTTGAACGCCCCGGTTGGGATTACACCACTACAGAAGGTAGGAACCACCTAGTCC
TCTATCGCCAGTTGCTCTTAGCGGGTCTCCAGAACGCGGGCAGAAGTCCCACCAATTTGGCCAAGGTAAAAGGGATAACC
CAGGGACCTAATGAGTCTCCCTCAGCCTTTTTAGAGAGACTCAAAGAGGCCTATCGCAGGTACACTCCTTATGACCCTGA
GGACCCAGGGCAAGAAACCAATGTGTCTATGTCATTCATCTGGCAGTCTGCCCCGGATATCGGGCGAAAGTTAGAGCGGT
TAGAAGATTTAAAGAGCAAGACCTTAGGAGACTTAGTGAGGGAAGCTGAAAAGATCTTTAATAAGCGAGAAACCCCGGAA
GAAAGAGAGGAACGTATCAAGAGAGAAACAGAGGAAAAAGAAGAACGCCGTAGGGCAGAGGATGAGCAGAGAGAGAAAGA
AAGGGACCGCAGAAGACATAGAGAGATGAGCAAGCTCTTGGCCACTGTAGTTATTGGTCAGAGACAGGATAGACAGGGGG
GAGAGCGGAGGAGGCCCCAACTTGATAAGGACCAATGCGCCTACTGCAAAGAAAAGGGACACTGGGCTAAGGACTGCCCA
AAGAAGCCACGAGGGCCCCGAGGACTGAGGCCCCAGACCTCCCTCCTAACCTTAGGTGACTAGGGAGGTCAGGGTCAGGA
GCCCCCCCCTGAACCCAGGATAACCCTCAAAGTCGGGGGGCAACCCGTCACCTTCCTGGTAGATACTGGGGCCCAACACT
CCGTGCTGACCCAAAATCCTGGACCCCTAAGTGACAAGGCTGCCTGGGTCCAAGGGGCTACTGGAGGAAAGCGGTATCGC
TGGACCACGGATCGCAAAGTACATCTAGCTACCGGTAAGGTCACCCACTCTTTCCTCCATGTACCAGACTGCCCCTATCC
TCTGCTAGGAAGAGACTTGCTGACTAAACTAAAAGCCCAAATCCACTTCGAGGGATCAGGAGCTCAGGTTATGGGACCAA
TGGGACTGCCCCTGCAAGTGCTGACCCTAAACATAGAAGATGAGTATCGGCTACATGAGACCTCAAAAGAGCCGGATGTT
TCTCTAGGGTCCACCTGGCTTTCTGATTTTCCCCAGGTCTGGGCGGAAACCGGGGGCATGGGACTGGCAGTTCGCCAAGC
TCCTCTGATCATACCTCTGAAGGCAACCTCTACCCCCGTGTCCATAAAACAATACCCCATGTCACAAGAAGCCAGACTGG
GGATCAAGCCCCACATACAGAGACTGTTGGACCAGGGAATACTGGTACCCTGCCAGTCCCCCTGGAACACGCCCCTGCTA
CCCGTTAAGAAACCAGGGACTAATGATTATAGGCCTGTCCAGGATCTGAGAGAAGTCAACAAGCGGGTGGAAGACATCCA
CCCCACCGTGCCCAACCCTTACAACCTCTTGAGCGGGCTCCCACCGTCCCACCAGTGGTACACTGTGCTTGATTTAAAGG
ATGCCTTTTTCTGCCTGAGACTCCACCCCACCAGTCAGCCTCTCTTCGCCTTTGAGTGGAGAGATCCAGAGATGGGAATC
TCAGGACAATTGACCTGGACCAGACTCCCACAGGGTTTCAAAAACAGTCCCACCCTGTTTGATGAGGCACTGCACAGAGA
CCTAGCAGACTTCCGGATCCAGCACCCAGACTTGATCCTGCTACAGTACGTGGATGACTTACTGCTGGCCGCCACTTCTG
AGCTCGACTGCCAACAAGGTACTCGGGCCCTGTTACAAACCCTAGGGAACCTCGGGTATCGGGCCTCGGCCAAGAAAGCC
CAAATTTGCCAGAAACAGGTCAAGTATCTGGGGTATCTTCTAAAAGAGGGTCAGAGATGGCTGACTGAGGCCAGAAAAGA

GACTGTGATGGGGCAGCCTACTCCGAAGACCCCTCGACAACTAAGGGAGTTCCTAGGGACGGCAGGCTTCTGTCGCCTCT
GGATCCCTGGGTTTGCAGAAATGGCAGCCCCCTTGTACCCTCTCACCAAAACGGGGACTCTGTTTAATTGGGGCCCAGAC
CAGCAAAAGGCCTATCAAGAAATCAAACAGGCTCTTCTAACTGCCCCAGCCCTGGGATTGCCAGATTTGACTAAGCCCTT
TGAACTCTTTGTTGACGAGAAGCAGGGCTACGCCAAAGGCGTCCTAACGCAAAAACTGGGACCTTGGCGTCGGCCGGTGG
CCTACCTGTCCAAAAAGCTAGACCCAGTGGCAGCTGGGTGGCCCCCTTGCCTACGGATGGTAGCAGCCATTGCCGTTCTG
ACAAAAGATGCAGGCAAGCTAACCATGGGACAGCCGCTAGTCATCCTGGCCCCCCATGCAGTAGAGGCACTAGTCAAGCA
ACCCCCTGACCGCTGGCTATCCAACGCCCGCATGACCCACTATCAGGCGATGCTCCTAGATACGGACCGGGTCCAGTTCG
GACCGGTGGTAGCCCTAAACCCGGCTACGTTGCTCCCCCTACCGGGGAAAGAGCCTCACCATGACTGCCTCGAGATCTTG
GCCGAGACACACGGAACCAGACCAGACCTCACGGACCAGCCCCTCCCAGACGCCGACCACACCTGGTATACAGATGGAAG
CAGCTTCCTGCAAGAGGGACAACGTAGGGCTGGAGCAGCGGTGACCACCGAGACCGAGGTAATCTGGGCCAAGGCGTTGC
CAGCCGGGACATCCGCCCAGCGAGCTGAACTAATAGCACTCACCCAGGCCCTAAAGATGGCAGAAGGTAAGAAGCTAAAT
GTTTATACTGATAGCCGCTATGCCTTTGCTACCGCCCATGTCCATGGAGAAATATATAGGAGACGTGGGTTGCTCACCTC
AGAAGGCAAGGAGATCAAGAACAAGGGCGAAATCTTGGCCTTACTGAAAGCTCTCTTTCTGCCCAAAAGACTCAGTATAA
TTCACTGCCCAGGACATCAGAAAGGCAATAGTGCTGAAGCTAAAGGCAACCGAATGGCGGACCAGGCAGCCCGGGAAGCA
GCCATGGGGACTGACACAAAGGCCTCCTCACTTCTCATAGAGACCTCAACCCCGTACACTCCAGACTTCTTCCATTATAC
TGAGACAGATATAAAGAACCTACAAGAGTTGGGAGCCACATATGATAGAGAGAAAAAATATTGGGTCCTGCAAGGTAAAC
CTGTGATGCCTGACCAGTTCACCTTTGAATTATTAGACTTCCTTCACCAGCTCACCCACCTTAGCTATCAGAAGATGAGG
GCACTTCTAGACAGGAAAGAAAGCCCCTATTACATGCTAAATAAAGATAAGATCCTCCACGAGGTGGCGGAATCATGCCA
AGCCTGTGTCCAAGTAAATGCCAGTAAGACTAAGATCAGGGCCGGAACACGAGTAAGAGGACATCGACCAGGCACCCATT
GGGAAATTGACTTTACTGAAGTGAAGCCCGGACTGTATGGGTACAAGTATCTCCTGGTATTCGTGGACACGTTCTCTGGC
TGGGTTGAAGCCTTCCCAACCAAACATGAGACTGCCAAAATAGTGACCAAGAAACTTCTGGAAGAAATATTTCCAAGGTT
TGGAATGCCCCAAGTGTTGGGGACTGATAATGGGCCTGCCTTCGTCTCCCAGGTAAGTCAGTCGGTGGCCAAGCTACTGG
GGATTGATTGGAAACTACATTGTGCTTACAGACCCCAGAGTTCAGGTCAGGTAGAAAGAATGAATAGGACAATCAAGGAG
ACTTTGACCAAATTAACGCTTGCAACTGGCACTAGAGACTGGGTACTCCTACTTCCCTTAGCCCTCTACCGAGCCCGCAA
CACTCCGGGCCCCCATGGACTCACTCCGTATGAAATCCTGTATGGGGCGCCCCCGCCCCTTGTTAATTTCCATGATCCTG
AAATGTCAAAGTTTACTAATAGCCCCTCTCTCCAAGCTCACTTACAGGCCCTCCAAGCAGTACAACGAGAGGTCTGGAAG
CCACTGGCCGCTGCCTATCAGGACCAGCTGGACCAGCCAGTGATACCACACCCCTTCCGTGTCGGCGACACCGTGTGGGT
ACGCCGGCACCAGACTAAGAACTTGGAACCTCGCTGGAAAGGACCCTACACCGTCCTGCTGACCACCCCCACCGCTCTCA
AAGTAGACGGCATCGCTGCGTGGATCCACGCCGCTCACGTAAAAGCGGCGACAACCCCTCCGGCCGGAACAGCATCAGGA
CCGACATGGAAGGTCCAGCGTTCTCAAAACCCCTTAAAGATAAGATTAACCCGTGGGGCCCCCTAATAATCCTGGGGATC
TTAATAAGGGCAGGAGTATCAGTACAACATGACAGCCCTCATCAGGTCTTCAATGTTACTTGGAGAGTTACCAACTTAAT
GACAGGACAAACAGCTAATGCTACCTCCCTCCTGGGGACAATGACCGATGCCTTTCCTAAACTGTACTTTGACTTGTGCG
ATTTAATAGGGGACGACTGGGATGAGACTGGACTCGGGTGTCGCACTCCCGGGGGAAGAAAAAGGGCAAGAACATTTGAC
TTCTATGTTTGCCCCGGGCATACTGTACCAACAGGGTGTGGAGGGCCGAGAGAGGGCTACTGTGGCAAATGGGGCTGTGA
GACCACTGGACAGGCATACTGGAAGCCATCATCATCATGGGACCTAATTTCCCTTAAGCGAGGAAACACCCCTCGGAATC
AGGGCCCCTGTTATGATTCCTCAGCGGTCTCCAGTGACATCAAGGGCGCCACACCGGGGGGTCGATGCAATCCCCTAGTC
CTGGAATTCACTGACGCGGGCAAAAAGGCCAGCTGGGATGGCCCCAAAGTATGGGGACTAAGACTGTACCGATCCACAGG
GACCGACCCGGTGACCCGGTTCTCTTTGACCCGCCAGGTCCTCAATATAGGGCCCCGCGTCCCCATTGGGCCTAATCCCG
TGATCACTGACCAGTTACCCCCCTCCCGACCCGTGCAGATCATGCTCCCCAGGCCTCCTCAGCCTCCTCCTCCAGGCGCA
GCCTCTATAGTCCCTGAGACTGCCCCACCTTCTCAACAACCTGGGACGGGAGACAGGCTGCTAAACCTGGTAGATGGAGC
CTACCAAGCTCTCAACCTCACCAGTCCTGACAAAACCCAAGAGTGCTGGTTGTGTCTGGTAGCGGGACCCCCCTACTACG
AAGGGGTTGCCGTCCTAGGTACTTATTCCAACCATACCTCTGCCCCAGCTAACTGCTCCGTGGCCTCCCAACACAAGCTG
ACCCTGTCCGAAGTGACCGGACAGGGACTCTGCGTAGGAGCAGTTCCCAAAACCCATCAGGCCCTGTGTAATACCACCCA
GAAGACGAGCGACGGGTCCTACTATCTGGCTGCTCCCGCCGGGACCATTTGGGCTTGCAACACCGGGCTCACTCCCTGCC
TATCTACCACTGTACTCGACCTCACCACCGATTACTGTGTCCTGGTTGAGCTCTGGCCAAAGGTGACCTACCACTCCCCT
GGTTATGTTTATGGCCAGTTTGAGAGAAAAACCAAATATAAAAGAGAGCCGGTGTCATTAACTCTGGCCCTGCTGTTGGG
AGGACTTACTATGGGCGGCATAGCTGCAGGAGTAGGAACCGGGACTACAGCCCTAGTGGCCACCAAACAATTCGAGCAGC
TCCAGGCAGCCATACATACAGACCTTGGGGCTTTAGAAAAGTCAGTCAGTGCCCTAGAAAAGTCTCTGACCTCGTTGTCT
GAGGTGGTCCTACAGAACCGGAGGGGATTAGATCTACTGTTCCTAAAAGAAGGAGGATTATGTGCTGCCCTAAAAGAAGA
ATGCTGTTTCTACGCGGACCACACTGGCGTAGTAAGAGATAGCATGGCAAAGCTAAGAGAAAGGTTGAACCAGAGACAAA
AATTGTTCGAATCAGGACAAGGGTGGTTTGAGGGACTGTTTAACAGGTCCCCATGGTTCACGACCTTAATATCCACCATT
ATGGGCCCCTTGATAATACTTTTATTAATCCTACTCTTCGGACCCTGTATTCTCAACCGCTTGGTCCAGTTTGTAAAAGA
CAGAATTTCGGTAGTGCAGGCCCTGGTTTTGACCCAACAGTATCACCAACTCAAATCAATAGATCCAGAAGAAGTGGAAT
CACGTGAATAAAAGATTTTATTCAGTTTCCAGAAAGAGGGGGGAATGAAAGACCCCACCATCAGGCTTAGCAAGCTAGCT
GCAGTAACGCCATTTTGCAAGGCATGAAAAAGTACCAGAGCTGAGTTCTCAAAAGTTACAAGAAAGTTCAGTTAAAGATT
AACAGTTAAAGATTAAGGCTGAATAATACTGGGACAGGGGCCAAATATCGGTGGTCAAGCACCTGGGCCCCGGCTCAGGG
CCAAGAACAGATGGCTCTCAGACGTCAGTGTTAGCAGAACTAGCTTCACTGATTTAGAAAAATAGAGGTGCACAGTGCTC

TGGCCACTCCTTGAACCTGTGTGTCTGCCAATGTTCTGACCAGGTGTGTGCCCATTGTTGAACCTTCATTAGACCCTTTC
CTCGTACCCCTCCCATACCCATTTCTTGAAAATAGACATTGTTTAGAACTAAAAAGTCCCACCTCAGTTTCCCCAAATGA
CCGAGAAAATACCCCAAGCCTTATTCGAACTAACCAACCAGCTCGCTTCTCGCTTCTGTAACCGCGCTTTTTGCTCCCCA
GCCCCAGCCCTATAAAAAGGGTAAAAACTCCACACTCGGCGCGCCAGTCCTCCGATAGACTGCGTCGCCCGGGTACCCGT
GTTCTCAATAAAGCCTCTTGCTGTTTGCATCCGAATCGTGGTCTCGCTGGTCCTTGAGAGGGTCTCCTCAGATTGATTGA
CTACCCACGTCGGGGGTCTTTCA
>Pmv2
TGAAAGACCCCACCATCAGGCTTAGCAAGCTAGCTGCAGTAACGCCATTTTGCAAGGCATGAAAAAGTACCAGAGCTGAG
TTCTCAAAAGTTACAAGAAAGTTCAGTTAAAGATTAACAGTTAAAGATTAAGGCTGAATAATACTGGGACAGGGGCCAAA
TATCGGTGGTCAAGCACCTGGGCCCCGGCTCAGGGCCAAGAACAGATGGCTCTCAGACGTCAGTGTTAGCAGAACTAGCT
TCACTGATTTAGAAAAATAGAGGTGCACAGTGCTCTGGCCACTCCTTAAACCTGTGTGTCTGCCAATGTTCTGACCAGGT
GTGTGCCCATTGTTGAACCTTCATTAGACCCTTTCCTCGTACCCCTCCCATACCCATTTCTTGAAAATAGACATTGTTTA
GAACTAAAAAGTCCCACCTCAGTTTCCCCAAATGACCGAGAAATACCCCAAGCCTTATTCGAACTAACCAACCAGCTCGC
TTCTCGCTTCTGTAACCGCGCTTTTTGCTCCCCAGCCCCAGCCCTATAAAAAGGGTAAAAACTCCACACTCGGCGCGCCA
GTCCTCCGATAGACTGCGTCGCCCGGGTACCCGTGTTCTCAATAAAGCCTCTTGCTGTTTGCATCCGAATCGTGGTCTCG
CTGGTCCTTGAGAGGGTCTCCTCAGATTGATTGACTACCCACGTCGGGGGTCTTTCATTTGGAGGTTCCACCGAGATTTG
GAGACCCCTGCCCAGGGACCACCGACCCCCCGCCGGGAGGTAAGCTGGCCAGCGGTCGTTTCGTGTCTGTCTCTGTCTTC
GTGCGTGTTTGTGCCGGCATCCAATGTTTGCGCCTGCGTCTGTACTAGTTAGCTAACTAGATCTGTATCTGGCGGTTCCG
CGGAAGAACTGACGAGTTCGTATTCCCGGCCGCAGCCCCTGGGAGACGTCCCAGCGGCCTCGGGGGCCCGTTTTGTGGCC
CATTCTGTATCAGTTAACCTACCCGAGTCGGACTTTTTGGAGCTCCGCCACTGTACGTGGCTTTGTTGGGGGACGAGAGA
CAGAGACACTTCCCGCCCCCGTCTGAATTTTTGCTTTCGGTTTTACGCCGAAGCCGCGCCGCGCGTCTGATTTGTTTGTT
GTTCTTTTGTTCTTCGTTAGTTTTCTTCTGTCTTTAAGTGTTTTCGAGATCATGGGACAGACCGTAACTACCCCTCTGAG
TTTAACCTTGCAGCACTGGGGAGATGTCCAGCGCATTGCATCCAACCAGTCTGTGGATGTCAGGAAGAGGCGCTGGATTA
CCTTCTGTTCCGCTGAATGGCCAACTTTCAATGTGGGATGGCCTCAGGATGGTACTTTCAATTTAAGTATTATCTCTCAG
GTTAAGTCTAGAGTGTTTTGTCCTGGTCCCCACGGACACCCGGATCAGGTCCCATATATCGTCACCTGGGAGGCACTTGC
CTATGACCCCCCTCCGTGGGTCAAACCGTTTGTGTCTCCTAAACTTCCTCCCTTGCCGACAGCTCCCGTCCTCCCGCCCG
GTCCTTCTGCGCAACCTCCGTCCCGATCTGCCCTTTACCCTGCCCTTACCCCCTCTATAAAGTCCAAACCTCCTAAGCCC
CAGGTTCTCCCTAATAGCGGCGGACCCCTCATTGACCTTCTCACAGAGGACCCCCCGCCGTACAGAGCACAACCCTCCTC
CTCTGCCAGGGAGAACGACGAAGAAGAGGCGGCCACCACCTCCGAGGTTTCCCCCCCTTCTCCCATGGTGTCTCGACTGC
GGGGAAGGAGAGACCCTCCCGCAGTGGACTCCACCACCTCCCAGGCATTTCCACTCCGCATGGGGGGAGATGGCCAGCTT
CAGTATTGGCCGTTTTCCTCTTCGGATTTATACAATTGGAAAAATAATAACCCTTCCTTTTCTGAAGATCCAGGTAAATT
GACGGCCTTGATTGAGTCCGTCCTCATCACCCACCAGCCCACCTGGGACGACTGTCAGCAGTTGTTGGGGACCCTGCTGA
CCGGAGAAGAAAAGCAGCGGGTGCTCCTAGAGGCTAGAAAGGCAGTCCGGGGCAATGATGGACGCCCCACTCAGTTGCCT
AATGAAGTCAATGCTGCTTTTCCCCTTGAACGCCCCGGTTGGGATTACACCACTACAGAAGGTAGGAACCACCTAGTCCT
CTATCGCCAGTTGCTCTTAGCGGGTCTCCAGAACGCGGGCAGAAGTCCCACCAATTTGGCCAAGGTAAAAGGGATAACCC
AGGGACCTAATGAGTCTCCCTCAGCCTTTTTAGAGAGACTCAAAGAGGCCTATCGCAGGTACACTCCTTATGACCCTGAG
GACCCAGGGCAAGAAACCAATGTGTCTATGTCATTCATCTGGCAGTCTGCCCCGGATATCGGGCGAAAGTTAGAGCGGTT
AGAAGATTTAAAGAGCAAGACCTTAGGAGACTTAGTGAGGGAAGCTGAAAAGATCTTTAATAAGCGAGAAACCCCGGAAG
AAAGAGAGGAACGTATCAAGAGAGAAACAGAGGAAAAAGAAGAACGCCGTAGGGCAGAGGATGAGCAGAGAGAAAAAGAA
AGGGACCGCAGAAGACATAGAGAGATGAGCAAGCTCTTGGCCACTGTAGTTATTGGTCAGAGACAGGATAGACAGGGGGG
AGAGCGGAGGAGGCCCCAACTTGATAAGGACCAATGCGCCTACTGCAAAGAAAAGGGACACTGGGCTAAGGACTGCCCAA
AGAAGCCACGAGGGCCCCGAGGACTGAGGCCCCAGACCTCCCTCCTAACCTTAGGTGACTAGGGAGGTCAGGGTCAGGAG
CCCCCCCCTGAACCCAGGATAACCCTCAAAGTCGGGGGGCAACCCGTCACCTTCCTGGTAGATACTGGGGCCCAACACTC
CGTGCTGACCCAAAATCCTGGACCCCTAAGTGACAAGGCTGCCTGGGTCCAAGGGGCTACTGGAGAAAAGCGGTATCGCT
GGACCACGGATCGCAAAGTACATCTAGCTACCGGTAAGGTCACCCACTCTTTCCTCCATGTACCAGACTGCCCCTATCCT
CTGCTAGGAAGAGACTTGCTGACTAAACTAAAAGCCCAAATCCACTTCGAGGGATCAGGAGCTCAGGTTATGGGACCAAT
GGGACTGCCCCTGCAAGTGCTGACCCTAAACATAGAAGATGAGTATCGGCTACATGAGACCTCAAAAGAGCCGGATGTTT
CTCTAGGGTCCACCTGGCTTTCTGATTTTCCCCAGGTCTGGGCGGAAACCGGGGGCATGGGACTGGCAGTTCGCCAAGCT
CCTCTGATCATACCTCTGAAGGCAACCTCTACCCCCGTGTCCATAAAACAATACCCCATGTCACAAGAAGCCAGACTGGG
GATCAAGCCCCACATACAGAGACTGTTGGACCAGGGAATACTGGTACCCTGCCAGTCCCCCTGGAACACGCCCCTGCTAC
CCGTTAAGAAACCAGGGACTAATGATTATAGGCCTGTCCAGGATCTGAGAGAAGTCAACAAGCGGGTGGAAGACATCCAC
CCCACCGTGCCCAACCCTTACAACCTCTTGAGCGGGCTCCCACCGTCCCACCAGTGGTACACTGTGCTTGATTTAAAGGA
TGCCTTTTTCTGCCTGAGACTCCACCCCACCAGTCAGCCTCTCTTCGCCTTTGAGTAGAGAGATCCAGAGATGGGAATCT
CAGGACAATTGACCTGGACCAGACTCCCACAGGGTTTCAAAAACAGTCCCACCCTGTTTGATGAGGCACTGCACAGAGAC
CTAGCAGACTTCCGGATCCAGCACCCAGACTTGATCCTGCTACAGTACGTGGATGACTTACTGCTGGCCGCCACTTCTGA
GCTCGACTGCCAACAAGGTACTCGGGCCCTGTTACAAACCCTAGGGAACCTCGGGTATCGGGCCTCGGCCAAGAAAGCCC
AAATTTGCCAGAAACAGGTCAAGTATCTGGGGTATCTTCTAAAAGAGGGTCAGAGATGGCTGACTGAGGCCAGAAAAGAG

ACTGTGATGGGGCAGCCTACTCCGAAGACCCCTCGACAACTAAGGGAGTTCCTAGGGACGGCAGGCTTCTGTCGCCTCTG
GATCCCAGGGTTTGCAGAAATGGCAGCCCCCTTGTACCCTCTCACCAAAACGGGGACTCTGTTTAATTGGGGCCCAGACC
AGCAAAAGGCCTATCAAGAAATCAAACAGGCTCTTCTAACTGCCCCAGCCCTGGGATTGCCAGATTTGACTAAGCCTTTT
GAACTCTTTGTTGACGAGAAGCAGGGCTACGCCAAAGGCGTCCTAACGCAAAAACTGGGACCTTGGCGTCGGCCGGTGGC
CTACCTGTCCAAAAAGCTAGACCCAGTGGCAGCTGGGTGGCCCCCTTGCCTACGGATGGTAGCAGCCATTGCCGTTCTGA
CAAAAGATGCAGGCAAGCTAACCATGGGACAGCCGCTAGTCATCCTGGCCCCCCATGCAGTAGAGGCACTAGTCAAGCAA
CCCCCTGACCGCTGGCTATCCAACGCCCGCATGACCCACTATCAGGCGATGCTCCTAGATACGGACCGGGTCCAGTTCGG
ACCGGTGGTAGCCCTAAACCCGGCTACGTTGCTCCCCCTACCGGGGAAAGAGCCTCATCATGACTGCCTCGAGATCTTGG
CCGAGACACACGGAACCAGACCAGACCTCACGGACCAGCCCCTCCCAGACGCCGACCACACCTGGTATACAGATGGAAGC
AGCTTCCTGCAAGAGGGACAACGTAGGGCTGGAGCAGCGGTGACCACCGAGACCGAGGTAATCTGGGCCAAGGCGTTGCC
AGCCGGGACATCCGCCCAGCGAGCTGAACTAATAGCACTCACCCAGGCCCTAAAGATGGCAGAAGGTAAGAAGCTAAATG
TTTATACTGATAGCCGCTATGCCTTTGCTACCGCCCATGTCCATGGAGAAATATATAGGAGACGTGGGTTGCTCACCTCA
GAAGGCAAGGAGATCAAGAACAAGGGCGAAATCTTGGCCTTACTGAAAGCTCTCTTTCTGCCCAAAAGACTCAGTATAAT
TCACTGCCCAGGACATCAGAAAGGCAATAGTGCTGAAGCTAAAGGCAACCGAATGGCGGACCAGGCAGCCCGGGAAGCAG
CCATGGGGACTGACACAAAGGCCTCCTCACTTCTCATAGAGACCTCAACCCCGTACACTCCAGACTTCTTCCATTATACT
GAGACAGATATAAAGAACCTACAAGAGTTGGGAGCCACATATGATAGAGAGAAAAAATATTGGGTCCTGCAAGGTAAACC
TGTGATGCCTGACCAGTTCACCTTTAAATTATTAGACTTCCTTCACCAGCTCACCCACCTTAGCTATCAGAAGATGAGGG
CACTTCTAGACAGGAAAGAAAGCCCCTATTACATGCTAAATAAAGATAAGATCCTCCACGAGGTGGCGGAATCATGCCAA
GCCTGTGTCCAAGTAAATGCCAGTAAGACTAAGATCAGGGCCGGAACACGAGTAAGAGGACATCGACCAGGCACCCATTG
GGAAATTGACTTTACTGAAGTGAAGCCCGGACTGTATGGGTACAAGTATCTCCTGGTATTCGTGGACACGTTCTCTGGCT
GGGTTGAAGCCTTCCCAACCAAACATGAGACTGCCAAAATAGTGACCAAGAAACTTCTGGAAGAAATATTTCCAAGGTTT
GGAATGCCCCAAGTGTTGGGGACTGATAATGGGCCTGCCTTCGTCTCCCAGGTAAGTCAGTCGGTGGCCAAGCTACTGGG
GATTGATTGGAAACTACAGACCCCAGAGTTCAGGTCAGGTAGAAAGAATGAATAGGACAATCAAGGAGACTTTGACCAAA
TTAACGCTTGCAACTGGCACTAGAGACTGGGTACTCCTACTTCCCTTAGCCCTCTACCGAGCCCGCAACACTCCGGGCCC
CCATGGACTCACTCCGTATGAAATCCTGTATGGGGCGCCCCCGCCCCTTGTTAATTTCCATGATCCTGAAATGTCAAAGT
TTACTAATAGCCCCTCTCTCCAAGCTCACTTACAGGCCCTCCAAGCAGTACAACGAGAGGTCTGGAAGCCACTGGCCGCT
GCCTATCAGGACCAGCTGGACCAGCCAGTGATACCACACCCCTTCCGTGTCGGCGACACCGTGTGGGTACGCCGGCACCA
GACTAAGAACTTGGAACCTCGCTGGAAAGGACCCTACACCGTCCTGCTGACCACCCCCACCGCTCTCAAAGTAGACGGCA
TCGCTGCGTGGATCCACGCCGCTCACGTAAAAGCGGCGACAACCCCTCCGGCCGGAACAGCATCAGGACCGACATGGAAG
GTCCAGCGTTCTCAAAACCCCTTAAAGATAAGATTAACCCGTGGGGCCCCCTAATAATCCTGGGGATCTTAATAAGGGCA
GGAGTATCAGTACAACATGACAGCCCTCATCAGGTCTTCAATGTTACTTGGAGAGTTACCAACTTAATGACAGGACAAAC
AGCTAATGCTACCTCCCTCCTGGGGACAATGACCGATGCCTTTCCTAAACTGTACTTTGACTTGTGCGATTTAATAGGGG
ACGACTGGGATGAGACTGGACTCGGGTGTCGCACTCCCGGGGGAAGAAAAAGGGCAAGAACATTTGACTTCTATGTTTGC
CCCGGGCATACTGTACCAACAGGGTGTGGAGGGCCGAGAGAGGGCTACTGTGGCAAATGGGGCTGTGAGACCACTGGACA
GGCATACTGGAAGCCATCATCATCATGGGACCTAATTTCCCTTAAGCGAGGAAACACCCCTCAGAATCAGGGCCCCTGTT
ATGATTCCTCAGCGGTCTCCAGTGACATCAAGGGCGCCACACCGGGGGGTCGATGCAATCCCCTAGTCCTGGAATTCACT
GACGCGGGCAAAAAGGCCAGCTGGGATGGCCCCAAAGTATGGGGACTAAGACTGTACCGATCCACAGGGACCGACCCGGT
GACCCGGTTCTCTTTGACCCGCCAGGTCCTCAATATAGGGCCCCGCGTCCCCATTGGGCCTAATCCCGTGATCACTGACC
AGTTACCCCCCTCCCGACCCGTGCAGATCATGCTCCCCAGGCCTCCTCAGCCTCCTCCTCCAGGCGCAGCCTCTATAGTC
CCTGAGACTGCCCCACCTTCTCAACAACCTGGGACGGGAGACAGGCTGCTAAACCTGGTAGATGGAGCCTACCAAGCTCT
CAACCTCACCAGTCCCGACAAAACCCAAGAGTGCTGGTTGTGTCTGGTAGCGGGACCCCCCTACTACGAAGGGGTTGCCG
TCCTAGGTACTTATTCCAACCATACCTCTGCCCCAGCTAACTGCTCCGTGGCCTCCCAACACAAGCTGACCCTGTCCGAA
GTGACCGGACAGGGACTCTGCGTAGGAGCAGTTCCCAAAACCCATCAGGCCCTGTGTAATACCACCCAGAAGACGAGCGA
CGGGTCCTACTATCTGGCTGCTCCCGCCGGGACCATTTGGGCTTGCAACACCGGGCTCACTCCCTGCCTATCTACCACTG
TACTCGACCTCACCACCGATTACTGTGTCCTGGTTGAGCTCTGGCCAAAGGTGACCTACCACTCCCCTGGTTATGTTTAT
GGCCAGTTTGAGAGAAAAACCAAATATAAAAGAGAGCCGGTGTCATTAACTCTGGCCCTGCTGTTGGGAGGACTTACTAT
GGGCGGCATAGCTGCAGGAGTAGGAACCGGGACTACAGCCCTAGTGGCCACCAAACAATTCGAGCAGCTCCAGGCAGCCA
TACATACAGACCTTGGGGCTTTAGAAAAGTCAGTCAGTGCCCTAGAAAAGTCTCTGACCTCGTTGTCTGAGGTGGTCCTA
CAGAACCGGAGGGGATTAGATCTACTGTTCCTAAAAGAAGGAGGATTATGTGCTGCCCTAAAAGAAGAATGCTGTTTCTA
CGCGGACCACACTGGCGTAGTAAGAGATAGCATGGCAAAGCTAAGAGAAAGGTTGAACCAGAGACAAAAATTGTTCGAAT
CAGGACAAGGGTGGTTTGAGGGACTGTTTAACAGGTCCCCATGGTTCACGACCTTAATATCCACCATTATGGGCCCCTTG
ATAATACTTTTATTAATCCTACTCTTCGGACCCTGTATTCTCAACCGCTTGGTCCAGTTTGTAAAAGACAGAATTTCGGT
AGTGCAGGCCCTGGTTTTGACCCAACAGTATCACCAACTCAAATCAATAGATCCAGAAGAAGTGGAATCACGTGAATAAA
AGATTTTATTCAGTTTCCAGAAAGAGGGGGGAATGAAAGACCCCACCATCAGGCTTAGCAAGCTAGCTGCAGTAACGCCA
TTTTGCAAGGCATGAAAAAGTACCAGAGCTGAGTTCTCAAAAGTTACAAGAAAGTTCAGTTAAAGATTAACAGTTAAAGA
TTAAGGCTGAATAATACTGGGACAGGGGCCAAATATCGGTGGTCAAGCACCTGGGCCCCGGCTCAGGGCCAAGAACAGAT
GGCTCTCAGACGTCAGTGTTAGCAGAACTAGCTTCACTGATTTAGAAAAATAGAGGTGCACAGTGCTCTGGCCACTCCTT

AAACCTGTGTGTCTGCCAATGTTCTGACCAGGTGTGTGCCCATTGTTGAACCTTCATTAGACCCTTTCCTCGTACCCCTC
CCATACCCATTTCTTGAAAATAGACATTGTTTAGAACTAAAAAGTCCCACCTCAGTTTCCCCAAATGACCGAGAAATACC
CCAAGCCTTATTCGAACTAACCAACCAGCTCGCTTCTCGCTTCTGTAACCGCGCTTTTTGCTCCCCAGCCCCAGCCCTAT
AAAAAGGGTAAAAACTCCACACTCGGCGCGCCAGTCCTCCGATAGACTGCGTCGCCCGGGTACCCGTGTTCTCAATAAAG
CCTCTTGCTGTTTGCATCCGAATCGTGGTCTCGCTGGTCCTTGAGAGGGTCTCCTCAGATTGATTGACTACCCACGTCGG
GGGTCTTTCA
>Pmv20
TGAAAGACCCCACCATCAGGCTTAGCAAGCTAGCTGCAGTAACGCCATTTTGCAAGGCATGAAAAAGTACCAGAGCTGAG
TTCTCAAAAGTTACAAGAAAGTTCAGTTAAAGATTAACAGTTAAAGATTAAGGCTGAATAATACTGGGACAGGGGCCAAA
TATCGGTGGTCAAGCACCTGGGCCCCGGCTCAGGGCCAAGAACAGATGGCTCTCAGACGTCAGTGTTAGCAGAACTAGCT
TCACTGATTTAGAAAAATAGAGGTGCACAGTGCTCTGGCCACTCCTTAAACCTGTGTGTCTGCCAATGTTCTGACCAGGT
GTGTGCCCATTGTTGAACCTTCATTAGACCCTTTCCTCGTACCCCTCCCATACCCATTTCTTGAAAATAGACATTGTTTA
GAACTAAAAAGTCCCACCTCAGTTTCCCCAAATGACCGAGAAATACCCCAAGCCTTATTCGAACTAACCAACCAGCTCGC
TTCTCGCTTCTGTAACCGCGCTTTTTGCTCCCCAGCCCCAGCCCTATAAAAAGGGTAAAAACTCCACACTCGGCGCGCCA
GTCCTCCGATAGACTGCGTCGCCCGGGTACCCGTGTTCCCAATAAAGCCTCTTGCTGTTTGCATCCGAATCGTGGTCTCG
CTGGTCCTTGAGAGGGTCTCCTCAGATTGATTGACTACCCACGTCGGGGGTCTTTCATTTGGAGGTCCCACCGAGATTTG
GAGACCCCTGCCCAGGGACCACCGATCCCCCGCCGGGAGGTAAGCTGGCCAGCGGTCGTTTCGTGTCTGTCTCTGTCTTC
GTGCGTGTTTGTGCCGGCATCCAATGTTTGCGCCTGCGTCTGTACTAGTTAGCTAACTAGATCTGTATCTGGCGGTTCCG
CGGAAGAACTGACGAGTTCGTATTCCCGGCCGCAGCCCCTGGGAGACGTCCCAGCGGCCTCGGGGGCCCGTTTTGTGGCC
CATTCTGTATCAGTTAACCTACCCGAGTCGGACTTTTTGGAGCTCCGCCACTGTACGTGGCTTTGTTGGGGGACGAGAGA
CAGAGACACTTCCCGCCCCCGTCTGAATTTTTGCTTTCGGTTTTACGCCGAAGCCGCGCCGCGCGTCTGATTTGTTTGTT
GTTCTTTTGTTCTTCGTTAGTTTTCTTCTGTCTTTAAGTGTTTTCGAGATCATGGGACAGACCGTAACTACCCCTCTGAG
TTTAACCTTGCAGCACTGGGGGGATGTCCAGCGCATTGCATCCAACCAGTCTGTGGATGTCAGGAAGAGGCGCTGGATTA
CCTTCTGTTCCGCTGAATGGCCAACTTTCAATGTGGGATGGCCTCAGGATGGTACTTTCAATTTAAGTATTATCTCTCAG
GTTAAGTCTAGAGTGTTTTGTCCTGGTCCCCACGGACACCCGGATCAGGTCCCATATATCGTCACCTGGGAGGCACTTGC
CTATGACCCCCCTCCGTGGGTCAAACCGTTTGTGTCTCCTAAACTTCCTCCCTTGCCGACAGCTCCCGTCCTCCCGCCCG
GTCCTTCTGCGCAACCTCCGTCCCGATCTGCCCTTTACCCTGCCCTTACCCCCTCTATAAAGTCCAAACCTCCTAAGCCC
CAGGTTCTCCCTGATAGCGGCGGACCCCTCATTGACCTTCTCACAGAGGACCCCCCGCCGTACAGAGCACAACCCTCCCG
CAGTGGACTCCACCACCTCCCAGGCATTTCCACTCCGCATGGGGGGAGATGGCCAGCTTCAGTATTGGCCGTTTTCCTCT
TCGGATTTATACAATTGGAAAAATAATAACCCTTCCTTTTCTGAAGATCCAGGTAAATTGACGGCCTTGATTGAGTCCGT
CCTCATCACCCACCAGCCCACCTGGGACGACTGTCAGCAGTTGTTGGGGACCCTGCTGACCGGAGAAGAAAAGCAGCGGG
TGCTCCTAGAGGCTAGAAAGGCAGTCCGGGGCAATGATGGACGCCCCACTCAGTTACCTAATGAAGTCAATGCTGCTTTT
CCCCTTGAACGCCCCGGTTGGGATTACACCACTACAGAAGGTAGGAACCACCTAGTCCTCTATCGCCAGTTGCTCTTAGC
GGGTCTCCAGAACGCGGGCAGAAGTCCCACCAATTTGGCCAAGGTAAAAGGGATAACCCAGGGACCTAATGAGTCTCCCT
CAGCCTTTTTAGAGAGACTCAAAGAGGCCTATCGCAGGTACACTCCTTATGACCCTGAGGACCCAGGGCAAGAAACCAAT
GTGTCTATGTCATTCATCTGGCAGTCTGCCCCGGATATCGGGCGAAAGTTAGAGCGGTTAGAAGATTTAAAGAGCAAGAC
CTTAGGAGACTTAGTGAGGGAAGCTGAAAAGATCTTTAATAAGCGAGAAACCCCGGAAGAAAGAGAGGAACGTATCAAGA
GAGAAACAGAGGAAAAAGAAGAACGCCGTAGGGCAGAGGATGAGCAGAGAGAGAAAGAAAGGGACCGCAGAAGACATAGA
GAGATGAGCAAGCTCTTGGCCACTGTAGTTATTGGTCAGAGACAGGATAGACAGGGGGGAGAGCGGAGGAGGCCCCAACT
TGATAAGGACCAATGCGCCTACTGCAAAGAAAAGGGACACTGGGCTAAGGACTGCCCAAAGAAGCCACGAGGGCCCCGAG
GACTGAGGCCCCAGACCTCCCTCCTAACCTTAGGTGACTAGGGAGGTCAGGGTCAGGAGCCCCCCCCTGAACCCAGGATA
ACCCTCAAAGTCGGGGGGCAACCCGTCACCTTCCTGGTAGATACTGGGGCCCAACACTCCGTGCTGACCCAAAATCCTGG
ACCCCTAAGTGACAAGGCTGCCTGGGTCCAAGGGGCTACTGGAGGAAAGCGGTATCGCTGGACCACGGATCGCAAAGTAC
ATCTAGCTACCGGTAAGGTCACCCACTCTTTCCTCCATGTACCAGACTGCCCCTATCCTCTGCTAGGAAGAGACTTGCTG
ACTAAACTAAAAGCCCAAATCCACTTCGAGGGATCAGGAGCTCAGGTTATGGGACCAATGGGACTGCCCCTGCAAGTGCT
GACCCTAAACATAGAAGATGAGTATCGGCTACATGAGACCTCAAAAGAGCCGGATGTTTCTCTAGGGTCCACCTGGCTTT
CTGATTTTCCCCAGGTCTGGGCGGAAACCGGGGGCATGGGACTGGCAGTTCGCCAAGCTCCTCTGATCATACCTCTGAAG
GCAACCTCTACCCCCGTGTCCATAAAACAATACCCCATGTCACAAGAAGCCAGACTGGGGATCAAGCCCCACATACAGAG
ACTGTTGGACCAGGGAATACTGGTACCCTGCCAGTCCCCCTGGAACACGCCCCTGCTACCCGTTAAGAAACCAGGGACTA
ATGATTATAGGCCTGTCCAGGATCTGAGAGAAGTCAACAAGCGGGTGGAAGACATCCACCCCACCGTGCCCAACCCTTAC
AACCTCTTGAGCGGGCTCCCACCGTCCCACCAGTGGTACACTGTGCTTGATTTAAAGGATGCCTTTTTCTGCCTGAGACT
CCACCCCACCAGTCAGCCTCTCTTCGCCTTTGAGTGGAGAGATCCAGAGATGGGAATCTCAGGACAATTGACCTGGACCA
GACTCCCACAGGGTTTCAAAAACAGTCCCACCCTGTTTGATGAGGCACTGCACAGAGACCTAGCAGACTTCCGGATCCAG
CACCCAGACTTGATCCTGCTACAGTACGTGGATGACTTACTGCTGGCCGCCACTTCTGAGCTCGACTGCCAACAAGGTAC
TCGGGCCCTGTTACAAACCCTAGGGAACCTCGGGTATCGGGCCTCGGCCAAGAAAGCCCAAATTTGCCAGAAACAGGTCA
AGTATCTGGGGTATCTTCTAAAAGAGGGTCAGAGATGGCTGACTGGGGCCAGAAAAGAGACTGTGATGGGGCAGCCTACT
CCGAAGACCCCTCGACAACTAAGGGAGTTCCTAGGGACGGCAGGCTTCTGTCGCCTCTGGATCCCTGGGTTTGCAGAAAT

GGCAGCCCCCTTGTACCCTCTCACCAAAACGGGGACTCTGTTTAATTGGGGCCCAGACCAGCAAAAGGCCTATCAAGAAA
TTAAACAGGCTCTTCTAACTGCCCCAGCCCTGGGATTGCCAGATTTGACTAAGCCCTTTGAACTCTTTGTTGACGAGAAG
CAGGGCTACGCCAAAGGCGTCCTAACGCAAAAACTGGGACCTTGGCGTCGGCCGGTGGCCTACCTGTCCAAAAAGCTAGA
CCCAGTGGCAGCTGGGTGGCCCCCTTGCCTACGGATGGTAGCAGCCATTGCCGTTCTGACAAAAGATGCAGGCAAGCTAA
CCATGGGACAGCCGCTAGTCATCCTGGCCCCCCATGCAGTAGAGGCACTAGTCAAGCAACCCCCTGACCGCTGGCTATCC
AACGCCCGCATGACCCACTATCAGGCGATGCTCCTAGATACGGACCGGGTCCAGTTCGGACCGGTGGTAGCCCTAAACCC
GGCTACGTTGCTCCCCCTACCGGGGAAAGAGCCTCACCATGACTGCCTCGAGATCTTGGCCGAGACACACGGAACCAGAC
CAGACCTCACGGACCAGCCCCTCCCAGACGCCGACCACACCTGGTATACAGATGGAAGCAGCTTCCTGCAAGAGGGACAA
CGTAGGGCTGGAGCAGCGGTGACCACCGAGACCGAGGTAATCTGGGCCAAGGCGTTGCCAGCCGGGACATCCGCCCAGCG
AGCTGAACTAATAGCACTCACCCAGGCCCTAAAGATGGCAGAAGGTAAGAAGCTAAATGTTTATACTGATAGCCGCTATG
CCTTTGCTACCGCCCATGTCCATGGAGAAATATATAGGAGACGTGGGTTGCTCACCTCAGAAGGCAAAGAGATCAAGAAC
AAGGGCGAAATCTTGGCCTTACTGAAAGCTCTCTTTCTGCCCAAAAGACTCAGTATAATTCACTGCCCAGGACATCAGAA
AGGCAATAGTGCTGAAGCTAAAGGCAACCGAATGGCGGACCAGGCAGCCCGAGAAGCAGCCATGGGGACTGACACAAAGG
CCTCCTCACTTCTCATAGAGACCTCAACCCCGTACACTCCAGACTTCTTCCATTACACTGAGACAGATATAAAGAACCTA
CAAGAGTTGGGAGCCACATATGATAGAGAGAAAAAATATTGGGTCCTGCAAGGTAAACCTGTGATGCCTGACCAGTTCAC
CTTTGAATTATTAGACTTCCTTCACCAGCTCACCCACCTTAGCTATCAGAAGATGAGGGCACTTCTAGACAGGAAAGAAA
GCCCCTATTACATGCTAAATAAAGATAAGATCCTCCACGAGGTGGCGGAATCATGCCAAGCCTGTGTCCAAGTAAATGCC
AGTAAGACTAAGATCAGGGCCGGAACACGAGTAAGAGGACATCGACCAGGCACCCATTGGGAAATTGACTTTACTAAAGT
GAAGCCCGGACTGTATGGGTACAAGTATCTCCTGGTATTCGTGGACACGTTCTCTGGCTGGGTTGAAGCCTTCCCAACCA
AACATGAGACTGCCAAAATAGTGACCAAGAAACTTCTGGAAGAAATATTTCCAAGGTTTGGAATGCCCCAAGTGTTGGGG
ACTGATAATGGGCCTGCCTTCGTCTCCCAGGTAAGTCAGTCGGTGGCCAAGCTACTGGGGATTGATTGGAAACTACATTG
TGCTTACAGACCCCAGAGTTCAGGTCAGGTAGAAAGAATGAATAGGACAATCAAGGAGACTTTGACCAAATTAACGCTTG
CAACTGGCACTAGAGACTGGGTACTCCTACTTCCCTTAGCCCTCTACCGAGCCCGCAACACTCCGGGCCCCCATGGACTC
ACTCCGTATGAAATCCTGTATGGGGCGCCCCCGCCCCTTGTTAATTTCCATGATCCTGAAATGTCAAAGTTTACTAATAG
CCCCTCTCTCCAAGCTCACTTACAGGCCCTCCAAGCAGTACAACGAGAGGTCTGGAAGCCACTGGCCGCTGCCTATCAGG
ACCAGCTGGACCAGCCAGTGATACCACACCCCTTCCGTGTCGGCGACACCGTGTGGGTACGCCGGCACCAGACTAAGAAC
TTGGAACCTCGCTGGAAAGGACCCTACACCGTCCTGCTGACCACCCCCACCGCTCTCAAAGTAGACGGCATCGCTGCGTG
GATCCACGCCGCTCACGTAAAAGCGGCGACAACCCCTCCGGCCGGAACAGCATCAGGACCGACATGGAAGGTCCAGCGTT
CTCAAAACCCCTTAAAGATAAGATTAACCCGTGGGGCCCCCTAATAATCCTGGGGATCTTAATAAGGGCAGGAGTATCAG
TACAACATGACAGCCCTCATCAGGTCTTCAATGTTACTTGGAGAGTTACCAACTTAATGACAGGACAAACAGCTAATGCT
ACCTCCCTCCTGGGGACAATGACCGATGCCTTTCCTAAACTGTACTTTGACTTGTGCGATTTAATAGGGGACGACTGGGA
TGAGACTGGACTCGGGTGTCGCACTCCCGGGGGAAGAAAAAGGGCAAGAACATTTGACTTCTATGTTTGCCCCGGGCATA
CTGTACCAACAGGGTGTGGAGGGCCGAGAGAGGGCTACTGTGGCAAATGGGGCTGTGAGACCACTGGACAGGCATACTGG
AAGCCATCATCATCATGGGACCTAATTTCCCTTAAGCGAGGAAACACCCCTCGGAATCAGGGCCCCTGTTATGATTCCTC
AGCGGTCTCCAGTGACATCAAGGGCGCCACACCGGGGGGTCGATGCAATCCCCTAGTCCTGGAATTCACTGACGCGGGCA
AAAAGGCCAGCTGGGATGGCCCCAAAGTATGGGGACTAAGACTGTACCGATCCACAGGGACCGACCCGGTGACCCGGTTC
TCTTTGACCCGCCAGGTCCTCAATATAGGGCCCCGCGTCCCCATTGGGCCTAATCCCGTGATCACTGACCAGTTACCCCC
CTCCCGACCCGTGCAGATCATGCTCCCCAGGCCTCCTCAGCCTCCTCCTCCAGGCGCAGCCTCTATAGTCCCTGAGACTG
CCCCACCTTCTCAACAACCTGGGACGGGAGACAGGCTGCTAAACCTGGTAGATGGAGCCTACCAAGCTCTCAACCTCACC
AGTCCCGACAAAACCCAAGAGTGCTGGTTGTGTCTGGTAGCGGGACCCCCCTACTACGAAGGGGTTGCCGTCCTAGGTAC
TTATTCCAACCATACCTCTGCCCCAGCTAACTGCTCCGTGGCCTCCCAACACAAGCTGACCCTGTCCGAAGTGACCGGAC
AGGGACTCTGCGTAGGAGCAGTTCCCAAAACCCATCAGGCCCTGTGTAATACCACCCAAAAGACGAGCGACGGGTCCTAC
TATCTGGCTGCTCCCGCCGGGACCATTTGGGCTTGCAACACCGGGCTCACTCCCTGCCTATCTACCACTGTACTCGACCT
CACCACCGATTACTGTGTCCTGGTTGAGCTCTGGCCAAAGGTGACCTACCACTCCCCTGGTTATGTTTATGGCCAGTTTG
AGAGAAAAACCAAATATAAAAGAGAGCCGGTGTCATTAACTCTGGCCCTGCTGTTGGGAGGACTTACTATGGGCGGCATA
GCTGCAGGAGTAGGAACCGGGACTACAGCCCTAGTGGCCACCAAACAATTCGAGCAGCTCCAGGCAGCCATACATACAGA
CCTTGGGGCTTTAGAAAAGTCAGTCAGTGCCCTAGAAAAGTCTCTGACCTCGTTGTCTGAGGTGGTCCTACAGAACCGGA
GGAGATTAGATCTACTGTTCCTAAAAGAAGGAAGATTATGTGCTGCCCTAAAAAAAGAATGCTGTTTCTACGCGGACCAC
ACTGGCGTAGTAAGAGATAGCATGGCAAAGCTAAGAGAAAGGTTGAACCAGAGACAAAAATTGTTCGAATCAGGACAAGG
GTGGTTTGAGGGACTGTTTAACAGGTCCCCATGGTTCACGACCTTAATATCCACCATTATGGGCCCCTTGATAATACTTT
TATTAATCCTACTCTTCGGACCCTGTATTCTCAACCGCTTGGTCCAGTTTGTAAAAGACAGAATTTCGGTAGTGCAGGCC
CTGGTTTTGACCCAACAGTATCACCAACTCAAATCAATAGATCCAGAAGAAGTGGAATCACGTGAATAAAAGATTTTATT
CAGTTTCCAGAAAGAGGGGGGAATGAAAGACCCCACCATCAGGCTTAGCAAGCTAGCTGCAGTAACGCCATTTTGCAAGG
CATGAAAAAGTACCAGAGCTGAGTTCTCAAAAGTTACAAGAAAGTTCAGTTAAAGATTAACAGTTAAAGATTAAGGCTGA
ATAATACTGGGACAGGGGCCAAATATCGGTGGTCAAGCACCTGGGCCCCGGCTCAGGGCCAAGAACAGATGGCTCTCAGA
CGTCAGTGTTAGCAGAACTAGCTTCACTGATTTAGAAAAATAGAGGTGCACAGTGCTCTGGCCACTCCTTAAACCTGTGT
GTCTGCCAATGTTCTGACCAGGTGTGTGCCCATTGTTGAACCTTCATTAGACCCTTTCCTCGTACCCCTCCCATACCCAT

TTCTTGAAAATAGACATTGTTTAGAACTAAAAAGTCCCACCTCAGTTTCCCCAAATGACCGAGAAATACCCCAAGCCTTA
TTCGAACTAACCAACCAGCTCGCTTCTCGCTTCTGTAACCGCGCTTTTTGCTCCCCAGCCCCAGCCCTATAAAAAGGGTA
AAAACTCCACACTCGGCGCGCCAGTCCTCCGATAGACTGCGTCGCCCGGGTACCCGTGTTCCCAATAAAGCCTCTTGCTG
TTTGCATCCGAATCGTGGTCTCGCTGGTCCTTGAGAGGGTCTCCTCAGATTGATTGACTACCCACGTCGGGGGTCTTTCA
>Pmv21
TGAAAGACCCCACCATCAGGCTTAGCAAGCTAGCTGCAGTAACGCCATTTTGCAAGGCATGAAAAAGTACCAGAGCTGAG
TTCTCAAAAGTTACAAGAAAGTTCAGTTAAAGATTAACAGTTAAAGATTAAGGCTGAATAATACTGAGACAGGGGCCAAA
TATCGGTGGTCAAGCACCTGGGCCCCGGCTCAGGGCCAAGAACAGATGGCTCTCAGACGTCAGTGTTAGCAGAACTAGCT
TCACTGATTTAGAAAAATAGAGGTGCACAGTGCTCTGGCCACTCCTTAAACCTGTGTGTCTGCCAATGTTCTGACCAGGT
GTGTGCCCATTGTTGAACCTTCATTAGACCCTTTCCTCGTACCCCTCCCATACCCATTTCTTGAAAATAGACATTGTTTA
GAACTAAAAAGTCCCACCTCAGTTTCCCCAAATGACCGAGAAATACCCCAAGCCTTATTCGAACTAACCAACCAGCTCGC
TTCTCGCTTCTGTAACCGCGCTTTTTGCTCCCCAGCCCCAGCCCTATAAAAAGGGTAAAAACTCCACACTCGGCGCGCCA
GTCCTCCGATAGACTGCGTCGCCCGGGTACCCGTGTTCCCAATAAAGCCTCTTGCTGTTTGCATCCGAATCGTGGTCTCG
CTGGTCCTTGAGAGGGTCTCCTCAGATTGATTGACTACCCACGTCGGGGGTCTTTCATTTGGAGGTCCCACCGAGATTTG
GAGACCCCTGCCCAGGGACCACCGACCCCCCGCCGGGAGGTAAGCTGGCCAGCGGTCGTTTCGTGTCTGTCTCTGTCTTC
GTGCGTGTTTGTGCCGGCATCCAATGTTTGCGCCTGCGTCTGTACTAGTTAGCTAACTAGATCTGTATCTGGCGGTTCCG
CGGAAGAACTGACGAGTTCGTATTCCCGGCCGCAGCCCCTGGGAGACGTCCCAGCGGCCTCGGGGGCCCGTTTTGTGGCC
CATTCTGTATCAGTTAACCTACCCGAGTCGGACTTTTTGGAGCTCCGCCACTGTACGTGGCTTTGTTGGGGGACGAGAGA
CAGAGACACTTCCCGCCCCCGTCTGAATTTTTGCTTTCGGTTTTACGCCGAAGCCGCGCCGCGCGTCTGATTTGTTTGTT
GTTCTTTTGTTCTTCGTTAGTTTTCTTCTGTCTTTAAGTGTTTTCGAGATCATGGGACAGACCGTAACTACCCCTCTGAG
TTTAACCTTGCAGCACTGGGGCGCTGTCCAGCGCATTGCATCCAACCAGTCTGTGGATGTCAGGAAGAGGCGCTGGATTA
CCTTCTGTTCCGCTGAATGGCCAACTTTCAATGTGGGATGGCCTCAGGATGGTACTTTCAATTTAAGTATTATCTCTCAG
GTTAAGTCTAGAGTGTTTTGTCCTGGTCCCCACGGACACCCGGATCAGGTCCCATATATCGTCACCTGGGAGGCACTTGC
CTATGACCCCCCTCCGTGGGTCAAACCGTTTGTGTCTCCTAAACTTCCTCCCTTGCCGACAGCTCCCGTCCTCCCGCCCG
GTCCTTCTGCGCAACCTCCGTCCCGATCTGCCCTTTACCCTGCCCTTACCCCCTCTATAAAGTCCAAACCTCCTAAGCCC
CAGGTTCTCCCTGATAGCGGCGGACCCCTCATTGACCTTCTCACAGAGGACCCCCCGCCGTACAGAGCACAACCCTCCTC
CTCTGCCAGGGAAAACGACGAAGAAGAGGCGGCCACCACCTCCGAGGTTTCCCCCCCTTCTCCCATGGTGTCTCGACTGC
GGGGAAGGAGAGACCCTCCCGCAGTGGACTCCACCACCTCCCAGGCATTTCCACTCCGCATGGGGGGAGATGGCCAGCTT
CAGTATTGGCCGTTTTCCTCTTCGGATTTATACAATTGGAAAAATAATAACCCTTCCTTTTCTGAAGATCCAGGTAAATT
GACGGCCTTGATTGAGTCCGTCCTCATCACCCACCAGCCCACCTGGGACGACTGTCAGCAGTTGTTGGGGACCCTGCTGA
CCGGAGAAGAAAAGCAGCGGGTGCTCCTAGAGGCTAGAAAGGCAGTCCGGGGCAATGATGGACGCCCCACTCAGTTGCCT
AATGAAGTCAATGCTGCTTTTCCCCTTGAACGCCCCGGTTGGGATTACACCACTACAGAAGGTAGGAACCACCTAGTCCT
CTATCGCCAGTTGCTCTTAGCGGGTCTCCAGAACGCGGGCAGAAGTCCCACCAATTTGGCCAAGGTAAAAGGGATAACCC
AGGGACCTAATGAGTCTCCCTCAGCCTTTTTAGAGAGACTCAAAGAGGCCTATCGCAGGTACACTCCTTATGACCCTGAG
GACCCAGGGCAAGAAACCAATGTGTCTATGTCATTCATCTGGCAGTCTGCCCCGGATATCGGGCGAAAGTTAGAGCGGTT
AGAAGGTTTAAAGAGCAAGACCTTAGGAGACTTAGTAAGGGAAGCTGAAAAGATCTTTAATAAGCGAGAAACCCCGGAAG
AAAGAAAGGAACGTATCAAGAGAGAAACAGAGGAAAAAGAAGAACGCCGTAGGGCAGAGGATGAGCAGAGAGAGAAAGAA
AGGGACCGCAGAAGACATAGAGAGATGAGCAAGCTCTTGGCCACTGTAGTTATTGGTCAGAGACAGGATAGACAGGGGGG
AGAGCGGAGGAGGCCCCAACTTGATAAGGACCAATGCGCCTACTGCAAAGAAAAGGGACACTGGGCTAAGGACTGCCCAA
AGAAGCCACGAGGGCCCCGAGGACTGAGGCCCCAGACCTCCCTCCTAACCTTAGGTGACTAGGGAGGTCAGGGTCAGGAG
CCCCCCCCTGAACCCAGGATAACCCTCAAAGTCGGGGGGCAACCCGTCACCTTCCTGGTAGATACTGGGGCCCAACACTC
CGTGCTGACCCAAAATCCTGGACCCCTAAGTGACAAGGCTGCCTGGGTCCAAGGGGCTACTGGAGGAAAGCGGTATCGCT
GGACCACGGATCGCAAAGTACATCTAGCTACCGGTAAGGTCACCCACTCTTTCCTCCATGTACCAGACTGCCCCTATCCT
CTGCTAGGAAGAGACTTGCTGACTAAACTAAAAGCCCAAATCCACTTCGAGGGATCAGGAGCTCAGGTTATGGGACCAAT
GGGACTGCCCCTGCAAGTGCTGACCCTAAACATAGAAGATGAGTATCGGCTACATGAGACCTCAAAAGAGCCGGATGTTT
CTCTAGGGTCCACCTGGCTTTCTGATTTTCCCCAGGTCTGGGCGGAAACCGGGGGCATGGGACTGGCAGTTCGCCAAGCT
CCTCTGATCATACCTCTGAAGGCAACCTCTACCCCCGTGTCCATAAAACAATACCCCATGTCACAAGAAGCCAGACTGGG
GATCAAGCCCCACATACAGAGACTGTTGGACCAGGGAATACTGGTACCCTGCCAGTCCCCCTGGAACACGCCCCTGCTAC
CCGTTAAGAAACCAGGGACTAATGATTATAGGCCTGTCCAGGATCTGAGAGAAGTCAACAAGCGGGTGGAAGACATCCAC
CCCACCGTGCCCAACCCTTACAACCTCTTGAGCGGGCTCCCACCGTCCCACCAGTGGTACACTGTGCTTGATTTAAAGGA
TGCCTTTTTCTGCCTGAGACTCCACCCCACCAGTCAGCCTCTCTTCGCCTTTGAGTGGAGAGATCCAGAGATGGGAATCT
CAGGACAATTGACCTGGACCAGACTCCCACAGGGTTTCAAAAACAGTCCCACCCTGTTTGATGAGGCACTGCACAGAGAC
CTAGCAGACTTCCGGATCCAGCACCCAGACTTGATCCTGCTACAGTACGTGGATGACTTACTGCTGGCCGCCACTTCTGA
GCTCGACTGCCAACAAGGTACTCGGGCCCTGTTACAAACCCTAGGGAACCTCGGGTATCGGGCCTCGGCCAAGAAAGCCC
AAATTTGCCAGAAACAGGTCAAGTATCTGGGGTATCTTCTAAAAGAGGGTCAGAGATGGCTGACTGAGGCCAGAAAAGAG
ACTGTGATGGGACAGCCTACTCCGAAGACCCCTCGACAACTAAGAGAGTTCCTAGGGACGGCAGGCTTCTGTCGCCTCTG
GATCCCTGGGTTTGCAGAAATGGCAGCCCCCTTGTACCCTCTCACCAAAACGGGGACTCTGTTTAATTGGGGCCCAGACC

AGCAAAAGGCCTATCAAGAAATCAAACAGGCTCTTCTAACTGCCCCAGCCCTGGGATTGCCAGATTTGACTAAGCCCTTT
GAACTCTTTGTTGACGAGAAGCAGGGCTACGCCAAAGGCGTCCTAACGCAAAAACTGGGACCTTGGCGTCGGCCGGTGGC
CTACCTGTCCAAAAAGCTAGACCCAGTGGCAGCTGGGTGGCCCCCTTGCCTACGGATGGTAGCAGCCATTGCCGTTCTGA
CAAAAGATGCAGGCAAGCTAACCATGGGACAGCCGCTAGTCATCCTGGCCCCCCATGCAGTAGAGGCACTAGTCAAGCAA
CCCCCTGACCGCTGGCTATCCAACGCCCGCATGACCCACTATCAGGCGATGCTCCTAGATACGGACCGGGTCCAGTTCGG
ACCGGTGGTAGCCCTAAACCCGGCTACGTTGCTCCCCCTACCGGGGAAAGAGCCTCACCATGACTGCCTCGAGATCTTGG
CCGAGACACACGGAACCAGACCAGACCTCACGGACCAGCCCCTCCCAGACGCCGACCACACCTGGTATACAGATGGAAGC
AGCTTCCTGCAAGAGGGACAACGTAGGGCTGGAGCAGCGGTGACCACCGAGACCGAGGTAATCTGGGCCAAGGCGTTGCC
AGCCGGGACATCCGCCCAGCGAGCTGAACTAATAGCACTCACCCAGGCCCTAAAGATGGCAGAAGGTAAGAAGCTAAATG
TTTATACTGATAGCCGCTATGCCTTTGCTACCGCCCATGTCCATGAAAAAATATATAGGAGACGTGGGTTGCTCACCTCA
GAAGGCAAGGAGATCAAGAACAAGGGCGAAATCTTGGCCTTACTGAAAGCTCTCTTTCTGCCCAAAAGACTCAGTATAAT
TCACTGCCCAGGACATCAGAAAGGCAATAGTGCTGAAGCTAAAGGCAACCAAATGGCGGACCAGGCAGCCCGGGAAGCAG
CCATGGGGACTGACACAAAGGCCTCCTCACTTCTCATAGAGACCTCAACCCCGTACACTCCAGACTTCTTCCATTATACT
GAGACAGATATAAAGAACCTACAAGAGTTGGGAGCCACATATGATAGAGAGAAAAAATATTGGGTCCTGCAAGGTAAACC
TGTGATGCCTGACCAGTTCACCTTTAAATTATTAGACTTCCTTCACCAGCTCACCCACCTTAGCTATCAGAAGATGAGGG
CACTTCTAGACAGGAAAGAAAGCCCCTATTACATGCTAAATAAAGATAAGATCCTCCACGAGGTGGCGGAATCATGCCAA
GCCTGTGTCCAAGTAAATGCCAGTAAGACTAAGATCAGGGCCGGAACACGAGTAAGAGGACATCGACCAGGCACCCATTG
GAAAATTGACTTTACTGAAGTGAAGCCCGGACTGTATGGGTACAAGTATCTCCTGGTATTCGTGGACACGTTCTCTGGCT
GGGTTAAAGCCTTCCCAACCAAACATGAGACTGCCAAAATAGTGACCAAGAAACTTCTGGAAGAAATATTTCCAAGGTTT
GGAATGCCCCAAGTGTTGGGGACTGATAATGGGCCTGCCTTCGTCTCCCAGGTAAGTCAGTCGGTGGCCAAGCTACTGGG
GATTGATTGGAAACTACATTGTGCTTACAGACCCCAGAGTTCAGGTCAGGTAGAAAGAATAAATAGGACAATCAAGGAGA
CTTTGACCAAATTAACGCTTGCAACTGGCACTAGAGACTGGGTACTCCTACTTCCCTTAGCCCTCTACCGAGCCCGCAAC
ACTCCGGGCCCCCATGGACTCACTCCGTATGAAATCCTGTATGGGGCGCCCCCGCCCCTTGTTAATTTCCATGATCCTGA
AATGTCAAAGTTTACTAATAGCCCCTCTCTCCAAGCTCACTTACAGGCCCTCCAAGCAGTACAACGAGAGGTCTGGAAGC
CACTGGCCGCTGCCTATCAGGACCAGCTGGACCAGCCAGTGATACCACACCCCTTCCGTGTCGGCGACACCGTGTGGGTA
CGCCGGCACCAGACTAAGAACTTGGAACCTCGCTAGAAAGGACCCTACACCGTCCTGCTGACCACCCCCACCGCTCTCAA
AGTAGACGGCATCGCTGCGTGGATCCACGCCGCTCACGTAAAAGCGGCGACAACCCCTCCGGCCGGAACAGCATCAGGAC
CGACATGAAAGGTCCAGCGCTCTCAAAACCCCTTAAAGATAAGATTAACCCGTGGGGCCCCCTAATAGTCCTGGGGATCT
TAATAAGGGCAGGAGTATCAGTACAACATGACAGCCCTCATCAGGTCTTCAATGTTACTTGGAGAGTTACCAACTTAATG
ACAGGACAAACAGCTAATGCTACCTCCCTCCTGGGGACAATGACCGATGCCTTTCCTAAACTGTACTTTGACTTGTGCGA
TTTAATAGGGGACGACTGGGATGAGACTGGACTCGGGTGTCGCACTCCCGGGGGAAGAAAAAGGGCAAGAACATTTGACT
TCTATGTTTGCCCCGGGCATACTGTACCAACAGGGTGTAGAGGGCCGAGAGAGGGCTACTGTGGCAAATGGGGCTGTGAG
ACCACTGGACAGGCATACTGGAAGCCATCATCATCATGGGACCTAATTTCCCTTAAGCGAGGAAACACCCCTCGGAATCA
GGGCCCCTGTTATGATTCCTCAGCGGTCTCCAGTGACATCAAGGGCGCCACACCGGGGGGTCGATGCAATCCCCTAGTCC
TGAAATTCACTGACGCGGGCAAAAAGGCCAGCTGAGATGGCCCCAAAGTATGGGGACTAAGACTGTACCGATCCACAGGG
ACCGACCCGGTGACCCGGTTCTCTTTGACCCGCCAGGTCCTCAATATAGGGCCCCGCGTCCCCATTGGGCCTAATCCCGT
GATCACTGACCAGTTACCCCCCTCCCGACCCGTGCAGATCATGCTCCCCAGGCCTCCTCAGCCTCCTCCTCCAGGCGCAG
CCTCTATAGTCCCTGAGACTGCCCCACCTTCTCAACAACCTGGGACGGGAGACAGGCTGCTAAACCTGGTAAATAGAGCC
TACCAAGCTCTCAACCTCACCAGTCCTGACAAAACCCAAGAGTGCTGGTTGTGTCTGGTAGCGGGACCCCCCTACTACGA
AGGGGTTGCCGTCCTAGGTACTTATTCCAACCATACCTCTGCCCCAGCTAACTGCTCCGTGGCCTCCCAACACAAGCTGA
CCCTGTCCGAAGTGACCGGACAGGGACTCTGCGTAGGAGCAGTTCCCAAAACCCATCAGGCCCTGTGTAATACCACCCAG
AAGACGAGCGACGGGTCCTACTATCTGGCTGCTCCCGCCGGGACCATTTGGGCTTGCAACACCGGGCTCACTCCCTGCCT
ATCTACCACTGTACTCGACCTCACCACCGATTACTGTGTCCTGGTTGAGCTCTGGCCAAAGGTGACCTACCACTCCCCTG
GTTATGTTTATGGCCAGTTTGAGAAAAAAACCAAATATAAAAGAGAGCCGGTGTCATTAACTCTGGCCCTGCTGTTGGGA
GGACTTACTATGGGCGGCATAGCTGCAGGAGTAGGAACCGGGACTACAGCCCTAGTGGCCACCAAACAATTCGAGCAGCT
CCAGGCAGCCATACATACAGACCTTGGGGCTTTAAAAAAGTCAGTCAGTGCCCTAGAAAAGTCTCTGACCTCGTTGTCTG
AGGTGGTCCTACAGAACCGGAGGGGATTAGATCTACTGTTCCTAAAAGAAGGAGGATTATGTGCTGCCCTAAAAGAAGAA
TGCTGTTTCTACGCGGACCACACTGGCGTAGTAAGAGATAGCATGGCAAAGCTAAGAAAAAGGTTGAACCAGAGACAAAA
ATTGTTCGAATCAGGACAAGGGTGGTTTGAAGGACTGTTTAACAGGTCCCCATGGTTCACGACCTTAATATCCACCATTA
TGGGCCCCTTGATAATACTTTTATTAATCCTACTCTTCGGACCCTGTATTCTCAACCGCTTGGTCCAGTTTGTAAAAGAC
AGAATTTCGGTAGTGCAGGCCCTGGTTTTGACCCAACAGTATCACCAACTCAAATCAATAGATCCAGAAAAAGTGGAATC
ACGTGAATAAAAGATTTTATTCAGTTTCCAGAAAGAGGGGGGAATGAAAGACCCCACCATCAGGCTTAGCAAGCTAGCTG
CAGTAACGCCATTTTGCAAGGCATGAAAAAGTACCAGAGCTGAGTTCTCAAAAGTTACAAGAAAGTTCAGTTAAAGATTA
ACAGTTAAAGATTAAGGCTGAATAATACTGAGACAGGGGCCAAATATCGGTGGTCAAGCACCTGGGCCCCGGCTCAGGGC
CAAGAACAGATGGCTCTCAGACGTCAGTGTTAGCAGAACTAGCTTCACTGATTTAGAAAAATAGAGGTGCACAGTGCTCT
GGCCACTCCTTAAACCTGTGTGTCTGCCAATGTTCTGACCAGGTGTGTGCCCATTGTTGAACCTTCATTAGACCCTTTCC
TCGTACCCCTCCCATACCCATTTCTTGAAAATAGACATTGTTTAGAACTAAAAAGTCCCACCTCAGTTTCCCCAAATGAC

CGAGAAATACCCCAAGCCTTATTCGAACTAACCAACCAGCTCGCTTCTCGCTTCTGTAACCGCGCTTTTTGCTCCCCAGC
CCCAGCCCTATAAAAAGGGTAAAAACTCCACACTCGGCGCGCCAGTCCTCCGATAGACTGCGTCGCCCGGGTACCCGTGT
TCCCAATAAAGCCTCTTGCTGTTTGCATCCGAATCGTGGTCTCGCTGGTCCTTGAGAGGGTCTCCTCAGATTGATTGACT
ACCCACGTCGGGGGTCTTTCA
>Pmv22
TGAAAGACCCCACCATCAGGCTTAGCAAGCTAGCTGCAGTAACGCCATTTTGCAAGGCATGAAAAAGTACCAGAGCTGAG
TTCTCAAAAGTTACAAGAAAGTTCAGTTAAAGATTAACAGTTAAAGATTAAGGCTGAATAATACTGGGACAGGGGCCAAA
TATCGGTGGTCAAGCACCTGGGCCCCGGCTCAGGGCCAAGAACAGATGGCTCTCAGACGTCAGTGTTAGCAGAACTAGCT
TCACTGATTTAGAAAAATAGAGGTGCACAGTGCTCTGGCCACTCCTTGAACCTGTGTGTCCGCCAATGTTCTGACCAGGT
GTGTGCCCATTGTTGAACCTTCATTAGACCCTTTCCTCGTACCCCTCCCATACCCATTTCTTGAAAATAGACATTGTTTA
GAACTAAAAAGTCCCACCTCAGTTTCCCCAAATGACCGAGAAATACCCCAAGCCTTATTCGAACTAACCAACCAGCTCGC
TTCTCGCTTCTGTAACCGCGCTTTTTGCTCCCCAGCCCCAGCCCTATAAAAAGGGTAAAAACTCCACACTCGGCGCGCCA
GTCCTCCGATAGACTGCGTCGCCCGGGTACCCGTGTTCTCAATAAAGCCTCTTGCTGTTTGCATCCGAATCGTGGTCTCG
CTGGTCCTTGAGAGGGTCTCCTCAGATTGATTGACTACCCACCTCGGGGGTCTTTCATTTGGAGGTTCCACCGAGATTTG
GAGACCCCTGCCCAGGGACCACCGACCCCCCGCCGGGAGGTAAGCTGGCCAGCGGTCGTTTCGTGTCTGTCTCTGTCTTC
GTGCGTGTTTGTGCCGGCATCCAATGTTTGCGCCTGCGTCTGTACTAGTTAGCTAACTAGATCTGTATCTGGCGGTTCCG
CGGAAGAACTGACGAGTTCGTATTCCCGGCCGCAGCCCCTGGGAGACGTCCCAGCGGCCTCGGGGGCCCGTTTTGTGGCC
CATTCTGTATCAGTTAACCTACCCGAGTCGGACTTTTTGGAGCTCCGCCACTGTACGTGGCTTTGTTGGGGGACGAGAGA
CAGAGACACTTCCCGCCCCCGTCTGAATTTTTGCTTTCGGTTTTACGCCGAAGCCGCGCCGCGCGTCTGATTTGTTTGTT
GTTCTTTTGTTCTTCGTTAGTTTTCTTCTGTCTTTAAGTGTTTTCGAGATCATGGGACAGACCGTAACTACCCCTCTGAG
TTTAACCTTGCAGCACTGGGGAGATGTCCAGCGCATTGCATCCAACCAGTCTGTGGATGTCAGGAAGAGGCGCTGGATTA
CCTTCTGTTCCGCTGAATGGCCAACTTTCAATGTGGGATGGCCTCAGGATGGTACTTTCAATTTAAGTATTATCTCTCAG
GTTAAGTCTAGAGTGTTTTGTCCTGGTCCCCACGGACACCCGGATCAGGTCCCATATATCGTCACCTGGGAGGCACTTGC
CTATGACCCCCCTCCGTGGGTCAAACCGTTTGTGTCTCCTAAACTTCCTCCCTTGCCGACAGCTCCCGTCCTCCCGCCCG
GTCCTTCTGCGCAACCTCCGTCCCGATCTGCCCTTTACCCTGCCCTTACCCCCTCTATAAAGTCCAAACCTCCTAAGCCC
CAGGTTCTCCCTGATAGCGGCGGACCCCTCATTGACCTTCTCACAGAGGACCCCCCGCCGTACAGAGCACAACCCTCCTC
CTCTGCCAGGGAGAACGACGAAGAAGAGGCGGCCACCACCTCCGAGGTTTCCCCCCCTTCTCCCATGGTGTCTCGACTGC
GGGGAAGGAGAGACCCTCCCGCAGTGGACTCCACCACCTCCCAGGCATTTCCACTCCGCATGGGGGGAGATGGCCAGCTT
CAGTATTGGCCGTTTTCCTCTTCGGATTTATACAATTGGAAAAATAATAACCCTTCCTTTTCTGAAGATCCAGGTAAATT
GACGGCCTTGATTGAGTCCGTCCTCATCACCCACCAGCCCACCTGGGACGACTGTCAGCAGTTGTTGGGGACCCTGCTGA
CCGGAGAAGAAAAGCAGCGGGTGCTCCTAGAGGCTAGAAAGGCAGTCCGGGGCAATGATGGACGCCCCACTCAGTTGCCT
AATGAAGTCAATGCTGCTTTTCCCCTTGAACGCCCCGGTTGGGATTACACCACTACAGAAGGTAGGAACCACCTAGTCCT
CTATCGCCAGTTGCTCTTAGCGGGTCTCCAGAACGCGGGCAGAAGTCCCACCAATTTGGCCAAGGTAAAAGGGATAACCC
AGGGACCTAATGAGTCTCCCTCAGCCTTTTTAGAGAGACTCAAAGAGGCCTATCGCAGGTACACTCCTTATGACCCTGAG
GACCCAGGGCAAGAAACCAATGTGTCTATGTCATTCATCTGGCAGTCTGCCCCGGATATCGGGCGAAAGTTAGAGCGGTT
AGAAGATTTAAAGAGCAAGACCTTAGGAGACTTAGTGAGGGAAGCTGAAAAGATCTTTAATAAGCGAGAAACCCCGGAAG
AAAGAGAGGAACGTATCAAGAGAGAAACAGAGGAAAAAGAAGAACGCCGTAGGGCAGAGGATGAGCAGAGAGAGAAAGAA
AGGGACCGCAGAAGACATAGAGAGATGAGCAAGCTCTTGGCCACTGTAGTTATTGGTCAGAGACAGGATAGACAGGGGGG
AGAGCGGAGGAGGCCCCAACTTGATAAGGACCAATGCGCCTACTGCAAAGAAAAGGGACACTGGGCTAAGGACTGCCCAA
AGAAGCCACGAGGGCCCCGAGGACTGAGGCCCCAGACCTCCCTCCTAACCTTAGGTGACTAGGGAGGTCAGGGTCAGGAG
CCCCCCCCGAACCCAGGATAACCCTCAAAGTCGGGGGGCAACCCGTCACCTTCCTGGTAGATACTGGGGCCCAACACTCC
GTGCTGACCCAAAATCCTGGACCCCTAAGTGACAAGGCTGCCTGGGTCCAAGGGGCTACTGGAGGAAAGCGGTATCGCTG
GACCACGGATCGCAAAGTACATCTAGCTACCGGTAAGGTCACCCACTCTTTCCTCCATGTACCAGACTGCCCCTATCCTC
TGCTAGGAAGAGACTTGCTGACTAAACTAAAAGCCCAAATCCACTTCGAGGGATCAGGAGCTCAGGTTATGGGACCAATG
GGACTGCCCCTGCAAGTGCTGACCCTAAACATAGAAGATGAGTATCGGCTACATGAGACCTCAAAAGAGCCGGATGTTTC
TCTAGGGTCCACCTGGCTTTCTGATTTTCCCCAGGTCTGGGCGGAAACCGGAGGCATGGGACTGGCAGTTCGCCAAGCTC
CTCTGATCATACCTCTGAAGGCAACCTCTACCCCCGTGTCCATAAAACAATACCCCATGTCACAAGAAGCCAGACTGGGG
ATCAAGCCCCACATACAGAGACTGTTGGACCAGGGAATACTGGTACCCTGCCAGTCCCCCTGGAACACGCCCCTGCTACC
CGTTAAGAAACCAGGGACTAATGATTATAGGCCTGTCCAGGATCTGAGAGAAGTCAACAAGCGGGTGGAAGACATCCACC
CCACCGTGCCCAACCCTTACAACCTCTTGAGCGGGCTCCCACCGTCCCACCAGTGGTACACTGTGCTTGATTTAAAGGAT
GCCTTTTTCTGCCTGAGACTCCACCCCACCAGTCAGCCTCTCTTCGCCTTTGAGTGGAGAGATCCAGAGATGGGAATCTC
AGGACAATTGACCTGGACCAGACTCCCACAGGGTTTCAAAAACAGTCCCACCCTGTTTGATGAGGCACTGCACAGAGACC
TAGCAGACTTCCGGATCCAGCACCCAGACTTGATCCTGCTACAGTACGTGGATGACTTACTGCTGGCCGCCACTTCTGAG
CTCGACTGCCAACAAGGTACTCGGGCCCTGTTACAAACCCTAGGGAACCTCGGGTATCGGGCCTCGGCCAAGAAAGCCCA
AATTTGCCAGAAACAGGTCAAGTATCTGGGGTATCTTCTAAAAGAGGGTCAGAGATGGCTGACTGAGGCCAGAAAAGAGA
CTGTGATGGGGCAGCCTACTCCGAAGACCCCTCGACAACTAAGGGAGTTCCTAGGGACGGCAGGCTTCTGTCGCCTCTGG
ATCCCTGGGTTTGCAGAAATGGCAGCCCCCTTGTACCCTCTCACCAAAACGGGGACTCTGTTTAATTGGGGCCCAGACCA

GCAAAAGGCCTATCAAGAAATCAAACAGGCTCTTCTAACTGCCCCAGCCCTGGGATTGCCAGATTTGACTAAGCCCTTTG
AACTCTTTGTTGACGAGAAGCAGGGCTACGCCAAAGGCGTCCTAACGCAAAAACTGGGACCTTGGCGTCGGCCGGTGGCC
TACCTGTCCAAAAAGCTAGACCCAGTGGCAGCTGGGTGGCCCCCTTGCCTACGGATGGTAGCAGCCATTGCCGTTCTGAC
AAAAGATGCAGGCAAGCTAACCATGGGACAGCCGCTAGTCATCCTGGCCCCCCATGCAGTAGAGGCACTAGTCAAGCAAC
CCCCTGACCGCTGGCTATCCAACGCCCGCATGACCCACTATCAGGCGATGCTCCTAGATACGGACCGGGTCCAGTTCGGA
CCGGTGGTAGCCCTAAACCCGGCTACGTTGCTCCCCCTACCGGGGAAAGAGCCTCACCATGACTGCCTCGAGATCTTGGC
CGAGACACACGGAACCAGACCAGACCTCACGGACCAGCCCCTCCCAGACGCCGACCACACCTGGTATACAGATGGAAGCA
GCTTCCTGCAAGAGGGACAACGTAGGGCTGGAGCAGCGGTGACCACCGAGACCGAGGTAATCTGGGCCAAGGCGTTGCCA
GCCGGGACATCCGCCCAGCGAGCTGAACTAATAGCACTCACCCAGGCCCTAAAGATGGCAGAAGGTAAGAAGCTAAATGT
TTATACTGATAGCCGCTATGCCTTTGCTACCGCCCATGTCCATGGAGAAATATATAGGAGACGTGGGTTGCTCACCTCAG
AAGGCAAGGAGATCAAGAACAAGGGCGAAATCTTGGCCTTACTGAAAGCTCTCTTTCTGCCCAAAAGACTCAGTATAATT
CACTGCCCAGGACATCAGAAAGGCAATAGTGCTGAAGCTAAAGGCAACCGAATGGCGGACCAGGCAGCCCGGGAAGCAGC
CATGGGGACTGACACAAAGGCCTCCTCACTTCTCATAGAGACCTCAACCCCGTACACTCCAGACTTCTTCCATTATACTG
AGACAGATATAAAGAACCTACAAGAGTTGGGAGCCACATATGATAGAGAGAAAAAATATTGGGTCCTGCAAGGTAAACCT
GTGATGCCTGACCAGTTCACCTTTGAATTATTAGACTTCCTTCACCAGCTCACCCACCTTAGCTATCAGAAGATGAGGGC
ACTTCTAGACAGGAAAGAAAGCCCCTATTACATGCTAAATAAAGATAAGATCCTCCACGAGGTGGCGGAATCATGCCAAG
CCTGTGTCCAAGTAAATGCCAGTAAGACTAAGATCAGGGCCGGAACACGAGTAAGAGGACATCGACCAGGCACCCATTGG
GAAATTGACTTTACTGAAGTGAAGCCCGGACTGTATGGGTACAAGTATCTCCTGGTATTCGTGGACACGTTCTCTGGCTG
GGTTGAAGCCTTCCCAACCAAACATGAGACTGCCAAAATAGTGACCAAGAAACTTCTGGAAGAAATATTTCCAAGGTTTG
GAATGCCCCAAGTGTTGGGGACTGATAATGGGCCTGCCTTCGTCTCCCAGGTAAGTCAGTCGGTGGCCAAGCTACTGGGG
ATTGATTGGAAACTACATTGTGCTTACAGACCCCAGAGTTCAGGTCAGGTAGAAAGAATGAATAGGACAATCAAGGAGAC
TTTGACCAAATTAACGCTTGCAACTGGCACTAGAGACTGGGTACTCCTACTTCCCTTAGCCCTCTACCGAGCCCGCAACA
CTCCGGGCCCCCATGGACTCACTCCGTATGAAATCCTGTATGGGGCGCCCCCGCCCCTTGTTAATTTCCATGATCCTGAA
ATGTCAAAGTTTACTAATAGCCCCTCTCTCCAAGCTCACTTACAGGCCCTCCAAGCAGTACAACGAGAGGCCTGGAAGCC
ACTGGCCGCTGCCTATCAGGACCAGCTGGACCAGCCAGTGATACCACACCCCTTCCGTGTCGGCGACACCGTGTGGGTAC
GCCGGCACCAGACTAAGAACTTGGAACCTCGCTGGAAAGGACCCTACACCGTCCTGCTGACCACCCCCACCGCTCTCAAA
GTAGACGGCATCGCTGCGTGGATCCACGCCGCTCACGTAAAAGCGGCGACAACCCCTCCGGCCGGAACAGCATCAGGACC
GACATGGAAGGTCCAGCGTTCTCAAAATCCCTTAAAGATAAGATTAACCCGTGGGGCCCCCTAATAATCCTGGGGATCTT
AATAAGGGCAGGAGTATCAGTACAACATGACAGCCCTCATCAGGTCTTCAATGTTACTTGGAGAGTTACCAACTTAATGA
CAGGACAAACAGCTAATGCTACCTCCCTCCTGGGGACAATGACCGATGCCTTTCCTAAACTGTACTTTGACTTGTGCGAT
TTAATAGGGGACGACTGGGATGAGACTGGACTCGGGTGTCGCACTCCCGGGGGAAGAAAAAGGGCAAGAACATTTGACTT
CTATGTTTGCCCCGGGCATACTGTACCAACAGGGTGTGGAGGGCCGAGAGAGGGCTACTGTGGCAAATGGGGCTGTGAGA
CCACTGGACAGGCATACTGGAAGCCATCATCATCATGGGACCTAATTTCCCTTAAGCGAGGAAACACCCCTCGGAATCAG
GGCCCCTGTTATGATTCCTCAGCGGTCTCCAGTGACATCAAGGGCGCCACACCGGGGGGTCGATGCAATCCCCTAGTCCT
GGAATTCACTGACGCGGGCAAAAAGGCCAGCTGGGATGGCCCCAAAGTATGGGGACTAAGACTGTACCGATCCACAGGGA
CCGACCCGGTGACCCGGTTCTCTTTGACCCGCCAGGTCCTCAATATAGGGCCCCGCGTCCCCATTGGGCCTAATCCCGTG
ATCACTGACCAGTTACCCCCCTCCCGACCCGTGCAGATCATGCTCCCCAGGCCTCCTCAGCCTCCTCCTCCAGGCGCAGC
CTCTATAGTCCCTGAGACTGCCCCACCTTCTCAACAACCTGGGACGGGAGACAGGCTGCTAAACCTGGTAGATGGAGCCT
ACCAAGCTCTCAACCTCACCAGTCCTGACAAAACCCAAGAGTGCTGGTTGTGTCTGGTAGCGGGACCCCCCTACTACGAA
GGGGTTGCCGTCCTAGGTACTTATTCCAACCATACCTCTGCCCCAGCTAACTGCTCCGTGGCCTCCCAACACAAGCTGAC
CCTGTCCGAAGTGACCGGACAGGGACTCTGCGTAGGAGCAGTTCCCAAAACCCATCAGGCCCTGTGTAATACCACCCAGA
AGACGAGCGACGGGTCCTACTATCTGGCTGCTCCCGCCGGGACCATTTGGGCTTGCAACACCGGGCTCACTCCCTGCCTA
TCTACCACTGTACTCGACCTCACCACCGATTACTGTGTCCTGGTTGAGCTCTGGCCAAAGGTGACCTACCACTCCCCTGG
TTATGTTTATGGCCAGTTTGAGAGAAAAACCAAATATAAAAGAGAGCCGGTGTCATTAACTCTGGCCCTGCTGTTGGGAG
GACTTACTATGGGCGGCATAGCTGCAGGAGTAGGAACCGGGACTACAGCCCTAGTGGCCACCAAACAATTCGAGCAGCTC
CAGGCAGCCATACATACAGACCTTGGGGCTTTAGAAAAGTCAGTCAGTGCCCTAGAAAAGTCTCTGACCTCGTTGTCTGA
GGTGGTCCTACAGAACCGGAGGGGATTAGATCTACTGTTCCTAAAAGAAGGAGGATTATGTGCTGCCCTAAAAGAAGAAT
GCTGTTTCTACGCGGACCACACTGGCGTAGTAAGAGATAGCATGGCAAAGCTAAGAGAAAGGTTGAACCAGAGACAAAAA
TTGTTCAAATCAGGACAAGGGTGGTTTGAGGGACTGTTTAACAGGTCCCCATGGTTCACGACCTTAATATCCACCATTAT
GGGCCCCTTGATAATACTTTTATTAATCCTACTCTTCGGACCCTGTATTCTCAACCGCTTGGTCCAGTTTGTAAAAGACA
GAATTTCGGTAGTGCAGGCCCTGGTTTTGACCCAACAGTATCACCAACTCAAATCAATAGATCCAGAAGAAGTGGAATCA
CGTGAATAAAAGATTTTATTCAGTTTCCAGAAAGAGGGGGGAATGAAAGACCCCACCATCAGGCTTAGCAAGCTAGCTGC
AGTAACGCCATTTTGCAAGGCATGAAAAAGTACCAGAGCTGAGTTCTCAAAAGTTACAAGAAAGTTCAGTTAAAGATTAA
CAGTTAAAGATTAAGGCTGAATAATACTGGGACAGGGGCCAAATATCGGTGGTCAAGCACCTGGGCCCCGGCTCAGGGCC
AAGAACAGATGGCTCTCAGACGTCAGTGTTAGCAGAACTAGCTTCACTGATTTAGAAAAATAGAGGTGCACAGTGCTCTG
GCCACTCCTTGAACCTGTGTGTCCGCCAATGTTCTGACCAGGTGTGTGCCCATTGTTGAACCTTCATTAGACCCTTTCCT
CGTACCCCTCCCATACCCATTTCTTGAAAATAGACATTGTTTAGAACTAAAAAGTCCCACCTCAGTTTCCCCAAATGACC

GAGAAATACCCCAAGCCTTATTCGAACTAACCAACCAGCTCGCTTCTCGCTTCTGTAACCGCGCTTTTTGCTCCCCAGCC
CCAGCCCTATAAAAAGGGTAAAAACTCCACACTCGGCGCGCCAGTCCTCCGATAGACTGCGTCGCCCGGGTACCCGTGTT
CTCAATAAAGCCTCTTGCTGTTTGCATCCGAATCGTGGTCTCGCTGGTCCTTGAGAGGGTCTCCTCAGATTGATTGACTA
CCCACCTCGGGGGTCTTTCA
>Pmv23
TGAAAGACCCCACCATCAGGCTTAGCAAGCTAGCTGCAGTAACGCCATTTTGCAAGGCATAAAAAAGTACCAGAGCTGAG
TTCTCAAAAGTTACAAGAAAGTTCAGTTAAAGATTAACAGTTAAAAATTAAGGCTGAATAATACTGGGACAGGGGCCAAA
TATCGGTGGTCAAGCACCTGGGCCCCGGCTCAGGGCCAAGAACAGATGGCTCTCAGACGTCAGTGTTAGCAGAACTAGCT
TCACTGATTTAGAAAAATAGAGGTGCACAGAGCTCTGGCCACTCCTTGAACCTGTGTGTCTGCCAATGTTCTGACCAGGT
GTGTGCCCATTGTTGAACCTTCATTAGACCCTCTCCTCGTACCCCTCCCATACCCATTTCTTAAAAATAGACATTGTTTA
GAACTAAAAAGTCCCACCTCAGTTTCCCCAAATGACCGAGAAATACCCCAAGCCTTATTCGAACTAACCAACCAGCTCGC
TTCTCGCTTCTGTAACCGCGCTTTTTGCTCCCCAGCCCCAGCCCTATAAAAAAGATAAAAACTCCACACTCGGCGCGCCA
GTCCTCCGATAGACTGCGTCGCCCGGGTACCCGTGTTCCCAATAAAGCCTCTTGCTGTTTGCATCCGAATCGTGGTCTCG
CTGGTCCTTGAGAGGGTCTCCTCAGATTGATTGACTACCCACGTCGGGGGTCTTTCATTTGGAGGTCCCACCGAGATTTG
GAGACCCCTGCCCAGGGACCACCGACCCCCCCGCCGGGAGGTAAGCTGGCCAGCGGTCGTTTCGTGTCTGTCTCTGTCTT
CGTGCGTGTTTGTGCCGGCATCCAATGTTTGCGCCTGCGTCTGTACTAGTTAGCTAACTAGATCTGTATCTGGCGGTTCC
GCGGAAGAACTGACGAGTTCGTATTCCCGGCCGCAGCCCCTGGGAGACGTCCCAGCGGCCTCGGGGGCCCGTTTTGTGGC
CCATTCTGTATCAGTTAACCTACCCGAGTCGGACTTTTTGGAGCTCCGCCACTGTACGTGGCTTTGTTGGGGGACGAGAA
ACAGAGACACTTCCCGCCCCCGTCTGAATTTTTGCTTTCGGTTTTACGCCGAAGCCGCGCCGCGCGTCTGATTTGTTTGT
TGTTCTTTTGTTCTTCGTTAGTTTTCTTCTGTCTTTAAGTGTTTTCGAGATCATGGGACAGACCGTAACTACCCCTCTGA
GTTTAACCTTGCAGCACTGGGGAGATGTCCAGCGCATTGCATCCAACCAGTCTGTGGATGTCAGGAAGAGGCGCTGGATT
ACCTTCTGTTCCGCTGAATGGCCAACTTTCAATGTGGGATGGCCTCAGGATGGTACTTTCAATTTAAGTATTATCTCTCA
GGTTAAGTCTAGAGTGTTTTGTCCTGGTCCCCACGGACACCCGGATCAGGTCCCATATATCGTCACCTGGGAGGCACTTG
CCTATGACCCCCCTCCGTGGGTCAAACCGTTTGTGTCTCCTAAACTCCCTCCCTTGCCGACAGCTCCCGTCCTCCCGCCC
GGTCCTTCTGCGCAACCTCCGTCCCGATCTGCCCTTTACCCTGCCCTTACCCCCTCTATAAAGTCCAAACCTCCTAAGCC
CCAGGTTCTCCCTGATAGCGGCGGACCCCTCATTGACCTTCTCACAGAGGACCCCCCGCCGTACAGAGCACAACCCTCCT
CCTCTGCCAGGGAGAACGACGAAGAAGAGGCGGCCACCACCTCCGAGGTTTCCCCCCCTTCTCCCATGGTGTCTCGACTG
CGGGGAAGGAGAGACCCTCCCGCAGTGGACTCCACCACCTCCCAGGCATTTCCACTCCGCATGGGGGGAGATGGCCAGCT
TCAGTATTGGCCGTTTTCCTCTTCGGATTTATACAATTGGAAAAATAATAACCCTTCCTTTTCTGAAGATCCAGGTAAAT
TGACGGCCTTGATTGAGTCCGTCCTCATCACCCACCAGCCCACCTGGGACGACTGTCAGCAGTTGTTGGGGACCCTGCTG
ACCGGAGAAGAAAAGCAGCGGGTGCTCCTAGAGGCTAGAAAGGCAGTCCGGGGCGATGATGGACGCCCCACTCAGTTGCC
TAATGAAGTCAATGCTGCTTTTCCCCTTGAACGCCCCGGTTGGGATTACACCACTACAGAAGGTAGGAACCACCTAGTCC
TCTATCGCCAGTTGCTCTTAGCGGGTCTCCAGAACGCGGGCAGAAGTCCCACCAATTTGGCCAAGGTAAAAGGGATAACC
CAGGGACCTAATGAGTCTCCCTCAGCCTTTTTAGAGAGACTCAAAGAGGCCTATCGCAGGTACACTCCTTATGACCCTGA
GGACCCAGGGCAAGAAACCAATGTGTCTATGTCATTCATCTGGCAGTCTGCCCCGGATATCGGGCGAAAGTTAGAGCGGT
TAGAAGATTTAAAGAGCAAGACCTTAGGAGACTTAGTGAGGGAAGCTGAAAAGATCTTTAATAAGCGAGAAACCCCGGAA
GAAAGAGAGGAACGTATCAAGAGAGAAACAGAGGAAAAAGAAGAACGCCGTAGGGCAGAGGATGAGCAGAGAGAGAAAGA
AAGGGACCGCAGAAGACATAGAGAGATGAGCAAGCTCTTGGCCACTGTAGTTATTGGTCAGAGACAGGATAGACAGGGGG
GAGAGCGGAGGAGGCCCCAACTTGATAAGGACCAATGCGCCTACTGCAAAGAAAAGGGACACTGGGCTAAGGACTGCCCA
AAAAAGCCACGAGGGCCCCGAGGACTGAGGCCCCAGACCTCCCTCCTAACCTTAGGTGACTAGGGAGGTCAGGGTCAGGA
GCCCCCCCCTGAACCCAGGATAACCCTCAAAGTCGGGGGGCAACCCGTCACCTTCCTGGTAGATACTGGGGCCCAACACT
CCGTGCTGACCCAAAATCCTGGACCCCTAAGTGATAAGGCTGCCTGGGTCCAAGGGGCTACTGGAGGAAAGCGGTATCGC
TGGACCACGGATCGCAAAGTACATCTAGCTACCGGTAAGGTCACCCACTCTTTCCTCCATGTACCAGACTGCCCCTATCC
TCTGCTAGGAAGAGACTTGCTGACTAAACTAAAAGCTCAAATCCACTTCGAGGGATCAGGAGCTCAGGTTATGGGACCAA
TGGGACTGCCCCTGCAAGTGCTGACCCTAAACATAGAAGATGAGTATCGGCTACATGAGACCTCAAAAGAGCCGGATGTT
TCTCTAGGGCCCACCTGGCTTTCTGATTTTCCCCAGGTCTGGGCGGAAACCGGGGGCATGGGACTGGCAGTTCGCCAAGC
TCCTCTGATCATACCTCTGAAGGCAACCTCTACCCCCGTGTCCATAAAACAATACCCCATGTCACAAGAAGCCAGACTGG
GGATCAAGCCCCACATACAGAGACTGTTGGACCAGGGAATACTGGTACCCTGCCAGTCCCCCTGGAACACGCCCCTGCTA
CCCGTTAAGAAACCAGGGACTAATGATTATAGGCCTGTCCAGGATCTGAGAGAAGTCAACAAGCGGGTGGAAGACATCCA
CCCCACCGTGCCCAACCCTTACAACCTCTTGAGCGGGCTCCCACCGTCCCACCAGTGGTACACTGTGCTTGATTTAAAGG
ATGCCTTTTTCTGCCTGAGACTCCACCCCACCAGTCAGCCTCTCTTCGCCTTTGAGTGGAGAGATCCAGAGATGGGAATC
TCAGGACAATTGACCTGGACCAGACTCCCACAGGGTTTCAAAAACAGTCCCACCCTGTTTGATGAGGCACTGCACAGAGA
CCTAGCAGACTTCCAGATCCAGCACCCAGACTTGATCCTGCTACAGTACGTGGATGACTTACTGCTGGCCGCCACTTCTG
AGCTAGACTGCCAACAAGGTACTCGGGCCCTGTTACAAACCCTAGGGAACCTCGGGTATCGGGCCTCGGCCAAGAAAGCC
CAAATTTGCCAGAAACAGGTCAAGTATCTGGGGTATCTTCTAAAAGAGGGTCAGAGATGGCTGACTGAGGCCAGAAAAGA
GACTGTGATGGGGCAGCCTACTCCGAAGACCCCTCGACAACTAAGGGAGTTCCTAGGGACGGCAGGCTTCTGTCGCCTCT
GGATCCCTGGGTTTGCAGAAATGGCAGCCCCCTTGTACCCTCTCACCAAAACGGGGACTCTGTTTAATTGGGGCCCAGAC

CAGCAAAAGGCCTATCAAGAAATCAAACAGGCTCTTCTAACTGCCCCAGCCCTGAGATTGCCAGATTTGACTAAGCCCTT
TGAACTCTTTGTCGACGAGAAGCAGGGCTACGCCAAAGGCGTCCTAACGCAAAAACTGGGACCTTGGCGTCGGCCGGTGG
TCTACCTGTCCAAAAAGCTAGACCCAGTGGCAGCTGGGTGGCCCCCTTGCCTACGGATGGTAGCAGCCATTGCCGTTCTG
ACAAAAGATGCAGGCAAGCTAACCATGGGACAGCCGCTAGTCATCCTGGCCCCCCATGCAGTAGAGGCACTAGTCAAGCA
ACCCCCTGACCGCTGGCTATCCAACGCCCGCATGACCCACTATCAGGCGATGCTCCTAGATACGGACCGGGTCCAGTTCG
GACCGGTGGTAGCCCTAAACCCGGCTACGTTGCTCCCCCTACCGGGGAAAGAGCCTCACCATGACTGCCTCGAGATCTTG
GCCGAGACACACGGAACCAGACCAGACCTCACGGACCAGCCCCTCCCAGACGCCGACCACACCTGGTATACAGATGGAAG
CAGCTTCCTGCAAGAGGGACAACGTAGGGCTGGAGCAGCGGTGACCACCGAGACCGAGGTAATCTGGGCCAAGGCGTTGC
CAGCCGGGACATCCGCCCAGCGAGCTGAACTAATAGCACTCACCCAGGCCCTAAAGATGGCAGAAGGTAAGAAGCTAAAT
GTTTATACTGATAGCCGCTATGCCTTTGCTACCGCCCATGTCCATGGAAAAATATATAGGAGACGTGGGTTGCTCACCTC
AGAAGGCAAGGAGATCAAGAACAAGGGCGAAATCTTGGCCTTACTGAAAGCTCTCTTTCTGCCCAAAAGACTCAGTATAA
TTCACTGCCCAGGACATCAGAAAGGCAATAGTGCTGAAGCTAAAGGCAACCGAATGGCGGACCAGGCAGCCCGGGAAGCA
GCCATGGGGACTGACACAAAGGCCTCCTCACTTCTCATAGAGACCTCAACCCCGTACACTCCAGACTTCTTCCATTATAC
TGAGACAGATATAAAGAACCTACAAGAGTTGGGAGCCACATATGATAGAGAGAAAAAATATTGGGTCCTGCAAGGTAAAC
CTGTGATGCCTGACCAGTTCACCTTTGAATTATTAGACTTCCTTCACCAGCTCACCCACCTTAGCTATCAGAAGATGAGG
GCACTTCTAGACAGGAAAGAAAGCCCCTATTACATGCTAAATAAAGATAAGATCCTCCACGAGGTGGCGGAATCATGCCA
AGCCTGTGTCCAAGTAAATGCCAGTAAGACTAAGATCAGGGCCGGAACACGAGTAAGAGGACATCGACCAGGCACCCATT
GGAAAATTGACTTTACTGAAGTAAAGCCCGGACTGTATGGGTACAAGTATCTCCTGGTATTCGTGGACACGTTCTCTGGC
TGGGTTGAAGCCTTCCCAACCAAACATGAAACTGCCAAAATAGTGACCAAGAAACTTCTGGAAAAAATATTTCCAAGGTT
TAGAATGCCCCAAGTGTTGGGGACTGATAATGGGCCTGCCTTCGTCTCCCAGGTAAGTCAGTCGGTGGCCAAGCTACTGG
GGATTGATTGAAAACTACATTGTGCTTACAGACCCCAGAGTTCAGGTCAGGTAAAAAAAATGAATAGGACAATCAAGGAG
ACTTTGACCAAATTAACGCTTGCAACTGGCACTAGAGACTGGGTACTCCTACTTCCCTTAGCCCTCTACCGAGCCCGCAA
CACTCCGGGCCCCCATGGACTCACTCCGTATGAAATCCTGTATGGGGCGCCCCCGCCCCTTGTTAATTTCCATGATCCTG
AAATGTCAAAGTTTACTAATAGCCCCTCTCTCCAAGCTCACTTACAGGCCCTCCAAGCAGTACAACGAGAGGTCTGGAAG
CCACTGGCCGCTGCCTATCAGGACCAGCTGGACCAGCCAGTGATACCACACCCCTTCCGTGTCGGCGACACCGTGTGGGT
ACGCCGGCACCAGACTAAGAACTTGGAACCTCGCTGGAAAGGACCCTACACCGTCCTGCTGACCACCCCCACCGCTCTCA
AAGTAGACGGCATCGCTGCGTGGATCCACGCCGCTCACGTAAAAGCGGCGACAACCCCTCCGGCCGGAACAGCATCAGGA
CCGACATGGAAGGTCCAGCGTTCTCAAAACCCCTTAAAGATAAGATTAACCCGTGGGGCCCCCTAATAGTCCTGGGAATC
TTAATAAGGGCAGGAGTATCAGTACAACATGACAGCCCTCATCAGGTCTTCAATGTTACTTGGAGAGTTACCAACTTAAT
GACAGGACAAACAGCTAATGCTACCTCCCTCCTGGGGACAATGACCGATGCCTTTCCTAAACTGTACTTTGACTTGTGCG
ATTTAATAGGGGACGACTGGGATGAGACTGGACTCGGGTGTCGCACTCCCGGGGGAAGAAAAAGGGCAAAAACATTTGAC
TTCTATGTTTGCCCCGGGCATACTGTACCAACAGGGTGTGGAGGGCCGAGAGAGGGCTACTGTGGCAAATGGGGCTGTGA
GACCACTGGACAGGCATACTGGAAGCCATCATCATCATGGGACCTAATTTCCCTTAAGCGAAGAAACACCCCTCAGAATC
AGGGCCCCTGTTATGATTCCTCAGCGGTCTCCAGTGACATCAAGGGCGCCACACCGGGGGGTCGATGCAATCCCCTAGTC
CTGGAATTCACTGACGCGGGCAAAAAGGCCAGCTGGGATGGCCCCAAAGTATGGGGACTAAGACTGTACCGATCCACAGG
GACCGACCCGGTGACCCGGTTCTCTTTGACCCGCCAGGTCCTCAATATAGGGCCCCGCGTCCCCATTGGGCCTAATCCCG
TGATCACTGACCAGTTACCCCCCTCCCGACCCGTGCAGATCATGCTCCCCAGGCCTCCTCAGCCTCCTCCTCCAGGCGCA
GCCTCTATAGTCCCTGAGACTGCCCCACCTTCTCAACAACCTGGGACGGGAGACAGGCTGCTAAACCTGGTAGATGGAGC
CTACCAAGCTCTCAACCTCACCAGTCCTGACAAAACCCAAGAGTGCTGGTTGTGTCTGGTAGCGGGACCCCCCTACTACG
AAGGGGTTGCCGTCCTAGGTACTTATTCCAACCATACCTCTGCCCCAGCTAACTGCTCCGTGGCCTCCCAACACAAGCTG
ACCCTGTCCGAAGTGACCGGACAGGGACTCTGCGTAGGAGCAGTTCCCAAAACCCATCAGGCCCTGTGTAATACCACCCA
GAAGACGAGCGACGGGTCCTACTATCTGGCTGCTCCCGCCGGGACCATTTGGGCTTGCAACACCGGGCTCACTCCCTGCC
TATCTACCACTGTGCTCGACCTCACCACCGATTACTGTGTCCTGGTTGAGCTCTGGCCAAAGGTGACCTACCACTCCCCT
GGTTATGTTTATGGCCAGTTTGAGAGAAAAACCAAATATAAAAGAGAGCCGGTGTCATTAACTCTGGCCCTGCTGTTGGG
AGGACTTACTATGGGCGGCATAGCTGCAGGAGTAGGAACCGGGACTACAGCCCTAGTGGCCACCAAACAATTCGAGCAGC
TCCAGGCAGCCATACATACAGACCTTGGGGCTTTAGAAAAGTCAGTCAGTGCCCTAAAAAAGTCTCTGACCTCGTTGTCT
GAGGTGGTCCTACAGAACCGGAGGAGATTAGATCTACTGTTCCTAAAAAAAGGAGGATTATGTGCTGCCCTAAAAGAAGA
ATGCTGTTTCTACGCGGACCACACTGGCGTAGTAAGAGATAGCATGGCAAAGCTAAAAAAAAGGTTGAACCAGAGACAAA
AATTGTTCGAATCAGGACAAGGGTGGTTTGAGGGACTGTTTAACAGGTCCCCATGGTTCACGACCTTAATATCCACCATT
ATGGGCCCCTTGATAATACTTTTATTAATCCTACTCTTCGGACCCTGTATTCTCAACCGCTTGGTCCAGTTTGTAAAAGA
CAGAATTTCGGTAGTGCAGGCCCTGGTTTTGACCCAACAGTATCACCAACTCAAATCAATAGATCCAGAAGAAGTGGAAT
CACGTGAATAAAAGATTTTATTCAGTTTCCAGAAAGAGGGGGGAATGAAAGACCCCACCATCAGGCTTAGCAAGCTAGCT
GCAGTAACGCCATTTTGCAAGGCATAAAAAAGTACCAGAGCTGAGTTCTCAAAAGTTACAAGAAAGTTCAGTTAAAGATT
AACAGTTAAAAATTAAGGCTGAATAATACTGGGACAGGGGCCAAATATCGGTGGTCAAGCACCTGGGCCCCGGCTCAGGG
CCAAGAACAGATGGCTCTCAGACGTCAGTGTTAGCAGAACTAGCTTCACTAATTTAGAAAAATAGAGGTGCACAGAGCTC
TGGCCACTCCTTGAACCTGTGTGTCTGCCAATGTTCTGACCAGGTGTGTGCCCATTGTTGAACCTTCATTAGACCCTCTC
CTCGTACCCCTCCCATACCCATTTCTTAAAAATAGACATTGTTTAGAACTAAAAAGTCCCACCTCAGTTTCCCCAAATGA

CCGAGAAATACCCCAAGCCTTATTCGAACTAACCAACCAGCTCGCTTCTCGCTTCTGTAACCGCGCTTTTTGCTCCCCAG
CCCCAGCCCTATAAAAAAGATAAAAACTCCACACTCGGCGCGCCAGTCCTCCGATAGACTGCGTCGCCCGGGTACCCGTG
TTCCCAATAAAGCCTCTTGCTGTTTGCATCCGAATCGTGGTCTCGCTGGTCCTTGAGAGGGTCTCCTCAGATTGATTGAC
TACCCACGTCGGGGGTCTTTCA
>Pmv24
TGAAAGACCCCACCATCAGGCTTAGCAAGCTAGCTGCAGTAACGCCATTTTGCAAGGCATGAAAAAGTACCAGAGCTGAG
TTCTCAAAAGTTACAAGAAAGTTCAGTTAAAGATTAACAGTTAAAGATTAAGGCTGAATAATACTGGGACAGGGGCCAAA
TATCGGTGGTCAAGCACCTGGGCCCCGGCTCAGGGCCAAGAACAGATGGCTCTCAGACGTCAGTGTTAGCAGAACTAGCT
TCACTGATTTAGAAAAATAGAGGTGCACAGTGCTCTGGCCACTCCTTAAACCTGTGTGTCTGCCAATGTTCTGACCAGGT
GTGTGCCCATTGTTGAACCTTCATTAGACCCTTTCCTCGTACCCCTCCCATACCCATTTCTTGAAAATAGACATTGTTTA
GAACTAAAAAGTCCCACCTCAGTTTCCCCAAATGACCGAGAAATACCCCAAGCCTTATTCGAACTAACCAACCAGCTCGC
TTCTCGCTTCTGTAACCGCGCTTTTTGCTCCCCAGCCCCAGCCCTATAAAAAGGGTAAAAACTCCACACTCGGCGCGCCA
GTCCTCCGATAGACTGCGTCGCCCGGGTACCCGTGTTCCCAATAAAGCCTCTTGCTGTTTGCATCCGAATCGTGGTCTCG
CTGGTCCTTGAGAGGGTCTCCTCAGATTGATTGACTACCCACGTCGGGGGTCTTTCATTTGGAGGTCCCACCGAGATTTG
GAGACCCCTGCCCAGGGACCACCGATCCCCCGCCGGGAGGTAAGCTGGCCAGCGGTCGTTTCGTGTCTGTCTCTGTCTTC
GTGCGTGTTTGTGCCGGCATCCAATGTTTGCGCCTGCGTCTGTACTAGTTAGCTAACTAGATCTGTATCTGGCGGTTCCG
CGGAAGAACTGACGAGTTCGTATTCCCGGCCGCAGCCCCTGGGAGACGTCCCAGCGGCCTCGGGGGCCCGTTTTGTGGCC
CATTCTGTATCAGTTAACCTACCCGAGTCGGACTTTTTGGAGCTCCGCCACTGTACGTGGCTTTGTTGGGGGACGAGAGA
CAGAGACACTTCCCGCCCCCGTCTGAATTTTTGCTTTCGGTTTTATGCCGAAGCCGCGCCGCGCGTCTGATTTGTTTGTT
GTTCTTTTGTTCTTCGTTAGTTTTCTTCTGTCTTTAAGTGTTTTCGAGATCATGGGACAGACCGTAACTACCCCTCTGAG
TTTAACCTTGCAGCACTGGGGGGATGTCCAGCGCATTGCATCCAACCAGTCTGTGGATGTCAGGAAGAGGCGCTGGATTA
CCTTCTGTTCCGCTGAATGGCCAACTTTCAATGTGGGATGGCCTCAGGATGGTACTTTCAATTTAAGTATTATCTCTCAG
GTTAAGTCTAGAGTGTTTTGTCCTGGTCCCCACGGACACCCGGATCAGGTCCCATATATCGTCACCTGGGAGGCACTTGC
CTATGACCCCCCTCCGTGGGTCAAACCGTTCGTGTCTCCTAAACTTCCTCCCTTGCCGACAGCTCCCGTCCTCCCGCCCG
GTCCTTCTGCGCAACCTCCGTCCCGATCTGCCCTTTACCCTGCCCTTACCCCCTCTATAAAGTCCAAACCTCCTAAGCCC
CAGGTTCTCCCTGATAGCGGCGGACCCCTCATTGACCTTCTCACAGAGGACCCCCCGCCGTACAGAGCACAACCCTCCTC
CTCTGCCAGGGAGAACGACGAAGAAGAGGCGGCCACCACCTCCGAGGTTTCCCCCCCTTCTCCCATGGTGTCTCGACTGC
GGGGAAGGAGAGACCCTCCCGCAGTGGACTCCACCACCTCCCAGGCATTTCCACTCCGCATGGGGGGAGATGGCCAGCTT
CAGTATTGGCCGTTTTCCTCTTCGGATTTATACAATTGGAAAAATAATAACCCTTCCTTTTCTGAAGATCCAGGTAAATT
GACGGCCTTGATTGAGTCCGTCCTCATCACCCACCAGCCCACCTGGGACGACTGTCAGCAGTTGTTGGGGACCCTGCTGA
CCGGAGAAGAAAAGCAGCGGGTGCTCCTAGAGGCTAGAAAGGCAGTCCGGGGCAATGATGGACGCCCCACTCAGTTGCCT
AATGAAGTCAATGCTGCTTTTCCCCTTGAACGCCCCGGTTGGGATTACACCACTACAGAAGGTAGGAACCACCTAGTCCT
CTATCGCCAGTTGCTCTTAGCGGGTCTCCAGAACGCGGGCAGAAGTCCCACCAATTTGGCCAAGGTAAAAGGGATAACCC
AGGGACCTAATGAGTCTCCCTCAGCCTTTTTAGAGAGACTCAAAGAGGCCTATCGCAGGTACACTCCTTATGACCCTGAG
GACCCAGGGCAAGAAACCAATGTGTCTATGTCATTCATCTGGCAGTCTGCCCCGGATATCGGGCGAAAGTTAGAGCGGTT
AGAAGATTTAAAGAGCAAGACCTTAGGAGACTTAGTGAGGGAAGCTGAAAAGATCTTTAATAAGCGAGAAACCCCGGAAG
AAAGAGAGGAACGTATCAAGAGAGAAACAGAGGAAAAAGAAGAACGCCGTAGGGCAGAGGATGAGCAGAGAGAGAAAGAA
AGGGACCGCAGAAGATATAGAGAGATGAGCAAGCTCTTGGCCACTGTAGTTATTGGTCAGAGACAGGATAGACAGGGGGG
AGAGCGGAGGAGGCCCCAACTTGATAAGGACCAATGCGCCTACTGCAAAGAAAAGGGACACTGGGCTAAGGACTGCCCAA
AGAAGCCACGAGGGCCCCGAGGACTGAGGCCCCAGACCTCCCTCCTAACCTTAGGTGACTAGGGAGGTCAGGGTCAGGAG
CCCCCCCCTGAACCCAGGATAACCCTCAAAGTCGGGGGGCAACCCGTCACCTTCCTGGTAGATACTGGGGCCCAACACTC
CGTGCTGACCCAAAATCCTGGACCCCTAAGTGACAAGGCTGCCTGGGTCCAAGGGGCTACTGGAGGAAAGCGGTATCGCT
GGACCACGGATCGCAAAGTACATCTAGCTACCGGTAAGGTCACCCACTCTTTCCTCCATGTACCAGACTGCCCCTATCCT
CTGCTAGGAAGAGACTTGCTGACTAAACTAAAAGCCCAAATCCACTTCGAGGGATCAGGAGCTCAGGTTATGGGACCAAT
GGGACTGCCCCTGCAAGTGCTGACCCTAAACATAGAAGATGAGTATCGGCTACATGAGACCTCAAAAGAGCCGGATGTTT
CTCTAGGGTCCACCTGGCTTTCTGATTTTCCCCAGGTCTGGGCGGAAACCGGGGGCATGGGACTGGCAGTTCGCCAAGCT
CCTCTGATCATACCTCTGAAGGCAACCTCTACCCCCGTGTCCATAAAACAATACCCCATGTCACAAGAAGCCAGACTGGG
GATCAAGCCCCACATACAGAGACTGTTGGACCAGGGAATACTGGTACCCTGCCAGTCCCCCTGGAACACGCCCCTGCTAC
CCGTTAAGAAACCAGGGACTAATGATTATAGGCCTGTCCAGGATCTGAGAGAAGTCAACAAGCGGGTGGAAGACATCCAC
CCCACCGTGCCCAACCCTTACAACCTCTTGAGCGGGCTCCCACCGTCCCACCAGTGGTACACTGTGCTTGATTTAAAGGA
TGCCTTTTTCTGCCTGAGACTCCACCCCACCAGTCAGCCTCTCTTCGCCTTTGAGTGGAGAGATCCAGAGATGGGAATCT
CAGGACAATTGACCTGGACCAGACTCCCACAGGGTTTCAAAAACAGTCCCACCCTGTTTGATGAGGCACTGCACAGAGAC
CTAGCAGACTTCCGGATCCAGCACCCAGACTTGATCCTGCTACAGTACGTGGATGACTTACTGCTGGCCGCCACTTCTGA
GCTCGACTGCCAACAAGGTACTCGGGCCCTGTTACAAACCCTAGGGAACCTCGGGTATCGGGCCTCGGCCAAGAAAGCCC
AAATTTGCCAGAAACAGGTCAAGTATCTGGGGTATCTTCTAAAAGAGGGTCAGAGATGGCTGACTGAGGCCAGAAAAGAG
ACTGTGATGGGGCAGCCTACTCCGAAGACCCCTCGACAACTAAGGGAGTTCCTAGGGACGGCAGGCTTCTGTCGCCTCTG
GATCCCTGGGTTTGCAGAAATGGCAGCCCCCTTGTACCCTCTCACCAAAACGGGGACTCTGTTTAATTGGGGCCCAGACC

AGCAAAAGGCCTATCAAGAAATTAAACAGGCTCTTCTAACTGCCCCAGCCCTGGGATTGCCAGATTTGACTAAGCCCTTT
GAACTCTTTGTTGACGAGAAGCAGGGCTACGCCAAAGGCGTCCTAACGCAAAAACTGGGACCTTGGCGTCGGCCGGTGGC
CTACCTGTCCAAAAAGCTAGACCCAGTGGCAGCTGGGTGGCCCCCTTGCCTACGGATGGTAGCAGCCATTGCCGTTCTGA
CAAAAGATGCAGGCAAGCTAACCATGGGACAGCCGCTAGTCATCCTGGCCCCCCATGCAGTAGAGGCACTAGTCAAGCAA
CCCCCTGACCGCTGGCTATCCAACGCCCGCATGACCCACTATCAGGCGATGCTCCTAGATACGGACCGGGTCCAGTTCGG
ACCGGTGGTAGCCCTAAACCCGGCTACGTTGCTCCCCCTACCGGGGAAAGAGCCTCACCATGACTGCCTCGAGATCTTGG
CCGAGACACACGGAACCAGACCAGACCTCACGGACCAGCCCCTCCCAGACGCCGACCACACCTGGTATACAGATGGAAGC
AGCTTCCTGCAAGAGGGACAACGTAGGGCTGGAGCAGCGGTGACCACCGAGACCGAGGTAATCTGGGCCAAGGCGTTGCC
AGCCGGGACATCCGCCCAGCGAGCTGAACTAATAGCACTCACCCAGGCCCTAAAGATGGCAGAAGGTAAGAAGCTAAATG
TTTATACTGATAGCCGCTATGCCTTTGCTACCGCCCATGTCCATGGAGAAATATATAGGAGACGTGGGTTGCTCACCTCA
GAAGGCAAAGAGATCAAGAACAAGGGCGAAATCTTGGCCTTACTGAAAGCTCTCTTTCTGCCCAAAAGACTCAGTATAAT
TCACTGCCCAGGACATCAGAAAGGCAATAGTGCTGAAGCTAAAGGCAACCGAATGGCGGACCAGGCAGCCCGGGAAGCAG
CCATGGGGACTGACACAAAGGCCTCCTCACTTCTCATAGAGACCTCAACCCCGTACACTCCAGACTTCTTCCATTACACT
GAGACAGATATAAAGAACCTACAAGAGTTGGGAGCCACATATGATAGAGAGAAAAAATATTGGGTCCTGCAAGGTAAACC
TGTGATGCCTGACCAGTTCACCTTTGAATTATTAGACTTCCTTCACCAGCTCACCCACCTTAGCTATCAGAAGATGAGGG
CACTTCTAGACAGGAAAGAAAGCCCCTATTACATGCTAAATAAAGATAAGATCCTCCACGAGGTGGCGGAATCATGCCAA
GCCTGTGTCCAAGTAAATGCCAGTAAGACTAAGATCAGGGCCGGAACACGAGTAAGAGGACATCGACCAGGCACCCATTG
GAAAATTGACTTTACTGAAGTGAAGCCCGGACTGTATGGGTACAAGTATCTCCTGGTATTCGTGGACACGTTCTCTGGCT
GGGTTGAAGCCTTCCCAACCAAACATGAGACTGCCAAAATAGTGACCAAGAAACTTCTGGAAAAAATATTTCCAAGGTTT
GGAATGCCCCAAGTGTTGGGGACTGATAATGGGCCTGCCTTCGTCTCCCAGGTAAGTCAGTCGGTGGCCAAGCTACTGGG
GATTGATTGGAAACTACATTGTGCTTACAGACCCCAGAGTTCAGGTCAGGTAAAAAGAATGAATAGGACAATCAAGGAGA
CTTTGACCAAATTAACGCTTGCAACTGGCACTAGAGACTGGGTACTCCTACTTCCCTTAGCCCTCTACCGAGCCCGCAAC
ACTCCGGGCCCCCATGGACTCACTCCGTATGAAATCCTGTATGGGGCGCCCCCGCCCCTTGTTAATTTCCATGATCCTGA
AATGTCAAAGTTTACTAATAGCCCCTCTCTCCAAGCTCACTTACAGGCCCTCCAAGCAGTACAACGAGAGGTCTGGAAGC
CACTGGCCGCTGCCTATCAGGACCAGCTGGACCAGCCAGTGATACCACACCCCTTCCGTGTCGGCGACACCGTGTGGGTA
CGCCGGCACCAGACTAAGAACTTGGAACCTCGCTGGAAAGGACCCTACACCGTCCTGCTGACCACCCCCACCGCTCTCAA
AGTAGACGGCATCGCTGCGTGGATCCACGCCGCTCACGTAAAAGCGGCGACAACCCCTCCGGCCGGAACAGCATCAGGAC
CGACATGAAAGGTCCAGCGTTCTCAAAACCCCTTAAAGATAAGATTAACCCGTGGGGCCCCCTAATAATCCTGGGGATCT
TAATAAGGGCAGGAGTATCAGTACAACATGACAGCCCTCATCAGGTCTTCAATGTTACTTGGAGAGTTACCAACTTAATG
ACAGGACAAACAGCTAATGCTACCTCCCTCCTGGGGACAATGACCGATGCCTTTCCTAAACTGTACTTTGACTTGTGCGA
TTTAATAGGGGACGACTGGGATGAGACTGGACTCGGGTGTCGCACTCCCGGGGGAAGAAAAAGGGCAAGAACATTTGACT
TCTATGTTTGCCCCGGGCATACTGTACCAACAGGGTGTGGAGGGCCGAGAGAGGGCTACTGTGGCAAATGGGGCTGTGAG
ACCACTGGACAGGCATACTGGAAGCCATCATCATCATGGGACCTAATTTCCCTTAAGCGAGGAAACACCCCTCGGAATCA
GGGCCCCTGTTATGATTCCTCAGCGGTCTCCAGTGACATCAAGGGCGCCACACCGGGGGGTCGATGCAATCCCCTAGTCC
TGGAATTCACTGACACGGGCAAAAAGGCCAGCTGGGATGGCCCCAAAGTATGGGGACTAAGACTGTACCGATCCACAGGG
ACCGACCCGGTGACCCGGTTCTCTTTGACCCGCCAGGTCCTCAATATAGGGCCCCGCGTCCCCATTGGGCCTAATCCCGT
GATCACTGACCAGTTACCCCCCTCCCGACCCGTGCAGATCATGCTCCCCAGGCCTCCTCAGCCTCCTCCTCCAGGCGCAG
CCTCTATAGTCCCTGAGACTGCCCCACCTTCTCAACAACCTGGGACGGGAGACAGGCTGCTAAACCTGGTAGATGGAGCC
TACCAAGCTCTCAACCTCACCAGTCCCGACAAAACCCAAGAGTGCTGGTTGTGTCTGGTAGCGGGACCCCCCTACTACGA
AGGGGTTGCCGTCCTAGGTACTTATTCCAACCATACCTCTGCCCCAGCTAACTGCTCCGTGGCCTCCCAACACAAGCTGA
CCCTGTCCGAAGTGACCGGACAGGGACTCTGCGTAGGAGCAGTTCCCAAAACCCATCAGGCCCTGTGTAATACCACCCAG
AAGACGAGCGACGGGTCCTACTATCTGGCTGCTCCCGCCGGGACCATTTGGGCTTGCAACACCGGGCTCACTCCCTGCCT
ATCTACCACTGTACTCGACCTCACCACCGATTACTGTGTCCTGGTTGAGCTCTGGCCAAAGGTGACCTACCACTCCCCTG
GTTATGTTTATGGCCAGTTTGAGAGAAAAACCAAATATAAAAGAGAGCCGGTGTCATTAACTCTGGCCCTGCTGTTGGGA
GGACTTACTATGGGCGGCATAGCTGCAGGAGTAGGAACCGGGACTACAGCCCTAGTGGCCACCAAACAATTCGAGCAGCT
CCAGGCAGCCATACATACAGACCTTGGGGCTTTAGAAAAGTCAGTCAGTGCCCTAGAAAAGTCTCTGACCTCGTTGTCTG
AGGTGGTCCTACAGAACCGGAGGGGATTAGATCTACTGTTCCTAAAAGAAGGAGGATTATGTGCTGCCCTAAAAGAAGAA
TGCTGTTTCTACGCGGACCACACTGGCGTAGTAAGAGATAGCATGGCAAAGCTAAGAGAAAGGTTGAACCAGAGACAAAA
ATTGTTCGAATCAGGACAAGGGTGGTTTGAGGGACTGTTTAACAGGTCCCCATGGTTCACGACCTTAATATCCACCATTA
TGGGCCCCTTGATAATACTTTTATTAATCCTACTCTTCGGACCCTGTATTCTCAACCGCTTGGTCCAGTTTGTAAAAGAC
AGAATTTCGGTAGTGCAGGCCCTGGTTTTGACCCAACAGTATCACCAACTCAAATCAATAGATCCAGAAGAAGTGGAATC
ACGTGAATAAAAGATTTTATTCAGTTTCCAGAAAGAGGGGGGAATGAAAGACCCCACCATCAGGCTTAGCAAGCTAGCTG
CAGTAACGCCATTTTGCAAGGCATGAAAAAGTACCAGAGCTGAGTTCTCAAAAGTTACAAGAAAGTTCAGTTAAAGATTA
ACAGTTAAAGATTAAGGCTGAATAATACTGGGACAGGGGCCAAATATCGGTGGTCAAGCACCTGGGCCCCGGCTCAGGGC
CAAGAACAGATGGCTCTCAGACGTCAGTGTTAGCAGAACTAGCTTCACTGATTTAGAAAAATAGAGGTGCACAGTGCTCT
GGCCACTCCTTAAACCTGTGTGTCTGCCAATGTTCTGACCAGGTGTGTGCCCATTGTTGAACCTTCATTAGACCCTTTCC
TCGTACCCCTCCCATACCCATTTCTTGAAAATAGACATTGTTTAGAACTAAAAAGTCCCACCTCAGTTTCCCCAAATGAC

CGAGAAATACCCCAAGCCTTATTCGAACTAACCAACCAGCTCGCTTCTCGCTTCTGTAACCGCGCTTTTTGCTCCCCAGC
CCCAGCCCTATAAAAAGGGTAAAAACTCCACACTCGGCGCGCCAGTCCTCCGATAGACTGCGTCGCCCGGGTACCCGTGT
TCCCAATAAAGCCTCTTGCTGTTTGCATCCGAATCGTGGTCTCGCTGGTCCTTGAGAGGGTCTCCTCAGATTGATTGACT
ACCCACGTCGGGGGTCTTTCA
>Pmv4
TGAAAGACCCCACCATCAGGCTTAGCAAGCTAGCTGCAGTAACGCCATTTTGCAAGGCATGAAAAAGTACCAGAGCTGAG
TTCTCAAAAGTTACAAGAAAGTTCAGTTAAAGATTAACAGTTAAAGATTAAGGCTGAATAATACTGGGACAGGGGCCAAA
TATCGGTGGTCAAGCACCTGGGCCCCGGCTCAGGGCCAAGAACAGATGGCTCTCAGACGTCAGTGTTAGCAGAACTAGCT
TCACTGATTTAAAAAAATAGAGGTGCACAGTGCTCTGGCCACTCTTTAAACCTGTGTGTCTGCCAATGTTCTGACCAGGT
GTGTGCCCATTGTTGAACCTTCATTAGACCCTTTCCTCGTACCCCTCCCATACCCATTTCTTGAAAATAGACATTGTTTA
GAACTAAAAAGTCCCACCTCAGTTTCCCCAAATGACCGAGAAATACCCCAAGCCTTATTTAAACTAACCAACCAGCTCGC
TTCTCGCTTCTGTAACCGCGCTTTTTGCTCCCCAGCCCCAGCCCTATAAAAAGGGTAAAAACTCCACACTCGGCGCGCCA
GTCCTCCGATAGACTGCGTCGCCCGGGTACCCGTGTTCCCAATAAAGCCTCTTGCTGTTTACATCCGAATCGTGGTCTCG
CTGGTCCTTGAGAGGGTCTCCTCAGATTGATTGACTACCCACGTCGGGGGTCTTTCATTTGGAGGTCCCACCGAGATTTA
GAGACCCCTGCCCAGGGACCACCGACCCCCCGCCGGGAGGTAAGCTGGCCAGCGGTCGTTTCGTGTCTGTCTCTGTCTTC
GTGCGTGTTTGTGCCGGCATCCAATGTTTGCGCCTGCGTCTGTACTAGTTAGCTAACTAGATCTGTATCTGGCGGTTCCG
CGGAAGAACTGACGAGTTCGTATTCCCGGCCGCAGCCCCTGGGAGACGTCCCAGCGGCCTCGGGGGCCCGTTTTGTGGCC
CATTCTGTATCAGTTAACCTACCCGAGTCGGACTTTTTGGAGCTCCGCCACTGTACGTGGCTTTGTTGGGGGACGAGAGA
CAGAGACACTTCCCGCCCCCGTCTGAATTTTTGCTTTCGGTTTTACGCCGAAGCCGCGCCGCGCGTCTGATTTGTTTGTT
GTTCTTTTGTTCTTCGTTAGTTTTCTTCTGTCTTTAAGTGTTTTCGAGATCATGGGACAGACCGTAACTACCCCTCTGAG
TTTAACCTTGCAGCACTGGGGAGATGTCCAGCGCATTGCATCCAACCAGTCTGTGGATGTCAGGAAGAGGCGCTGGATTA
CCTTCTGTTCCGCTGAATGGCCAATTTTCAATGTGGGATGGCCTCAGGATGGTACTTTCAATTTAAGTATTATCTCTCAG
GTTAAGTCTAGAGTGTTTTGTCCTGGTCCCCACGGACACCCGGATCAGGTCCCATATATCGTCACCTGGGAGGCACTTGC
CTATGACCCCCCTCCGTGGGTCAAACCGTTTGTGTCTCCTAAACTTCCTCCCTTGCCGACAGCTCCCGTCCTCCCGCCCG
GTCCTTCTGCGCAACCTCCGTCCCGATCTGCCCTTTACCCTGCCCTTACCCCCTCTATAAAGTCCAAACCTCCTAAGCCC
CAGGTTCTCCCTGATAGCGGCGGACCCCTCATTGACCTTCTCACAGAGGACCCCCCGCCGTACAGAGCACAACCCTCCTC
CTCTGCCAGGGAGAACGACGAAGAAGAGGCGGCCACCACCTCCGAGGTTTCCCCCCCTTCTCCCATGGTGTCTCGACTGC
GGGGAAGGAGAGACCCTCCCGCAGTGGACTCCACCACCTCCCAGGCATTTCCACTCCGCATGGGGGGAGATGGCCAGCTT
CAGTATTGGCCGTTTTCCTCTTCGGATTTATACAATTGGAAAAATAATAACCCTTCCTTTTCTGAAGATCCAGGTAAATT
GACGGCCTTAATTGAGTCCGTCCTCATCACCCACCAGCCCACCTGGGACGACTGTCAGCAGTTGTTGGGGACCCTGCTGA
CCGGAAAAGAAAAGCAGCGGGTGCTCCTAGAGGCTAGAAAGGCAGTCCGGGACAATGATGGACGCCCCACTCAGTTGCCT
AATGAAGTCAATGCTGCTTTTCCCCTTAAACGCCCCGGTTGGGATTACACCACTACAGAAGGTAGGAACCACCTAGTCCT
CTATCGCCAGTTGCTCTTAGCGGGTCTCCAGAACGCGGGCAGAAGTCCCACCAATTTGGCCAAGGTAAAAGGGATAACCC
AGGGACCTAATGAGTCTCCCTCAGCCTTTTTAGAGAGACTCAAAGAGGCCTATCGCAGGTACACTCCTTATGACCCTGAG
GACCCAGGGCAAGAAACCAATGTGTCTATGTCATTCATCTGGCAGTCTGCCCCGGATATCGGGCAAAAGTTAGAGCGGTT
AAAAGATTTAAAGAGCAAGACCTTAGGAGACTTAGTGAGGAAAGCTGAAAAGATCTTTAATAAGCGAAAAACCCCGGAAG
AAAGAGAGGAACGTATCAAGAGAAAAACAGAGGAAAAAGAAGAACGCCGTAGGGCAGAGGATGAGCAGAGAAAAAAAAAA
AAGGGACCGCAGAAGACATAGAGAAATGAGCAAGCTCTTGGCCACTGTAGTTATTGGTCAGAGACAGGATAGACAGGGGG
GAGAGCGGAGGAGGCCCCAACTTGATAAGGACCAATGCGCCTACTGCAAAGAAAAGGGACACTGGGCTAAGGACTGCCCA
AAGAAGCCACGAGGGCCCCGAGGACTGAGGCCCCAGACCTCCCTCCTAACCTTAGGTGACTAGGAAGGTCAGGGTCAGGA
GCCCCCCCCTGAACCCAGGATAACCCTCAAAGTCGGGGGGCAACCCGTCACCTTCCTGGTAGATACTGGGGCCCAACACT
CCGTGCTGACCCAAAATCCTGGACCCCTAAGTGACAAGGCTGCCTGGGTCCAAGGGGCTACTGGAAGAAAGCGGTATCGC
TGGACCACGGATCGCAAAGTACATCTAGCTACCGGTAAGGTCACCCACTCTTTCCTCCATGTACCAGACTGCCCCTATCC
TCTGCTAAAAAGAGACTTGCTGACTAAACTAAAAGCCCAAATCCACTTCGAGGGATCAGGAGCTCAGGTTATGGGACCAA
TGAGACTGCCCCTGCAAGTGCTGACCCTAAACATAGAAGATGAGTATCGGCTACATGAGACCTCAAAAGAGCCGGATGTT
TCTCTAGGGTCCACCTGGCTTTCTGATTTTCCCCAGGTCTGGGCAAAAACCGGGGGCATGGGACTGGCAGTTCGCCAAGC
TCCTCTGATCATACCTCTAAAGGCAACCTCTACCCCCGTGTCCATAAAACAATACCCCATGTCACAAGAAGCCAGACTGG
GGATCAAGCCCCACATACAGAGACTGTTGGACCAGGGAATACTGGTACCCTGCCAGTCCCCCTGAAACACGCCCCTGCTA
CCCGTTAAAAAACCAGGGACTAATGATTATAGGCCTGTCCAGGATCTGAGAAAAGTCAACAAGCGGGTAAAAGACATCCA
CCCCACCGTGCCCAACCCTTACAACCTCTTGAGCGGGCTCCCACCGTCCCACCAGTAGTACACTGTGCTTAATTTAAAGG
ATGCCTTTTTCTGCCTGAGACTCCACCCCACCAGTCAGCCTCTCTTCGCCTTTGAGTGGAGAGATCCAGAGATGGGAATC
TCAGGACAATTGACCTGGACCAGACTCCCACAGGGTTTCAAAAACAGTCCCACCCTGTTTGATGAGGCACTGCACAGAGA
CCTAGCAGACTTCCGGATCCAGCACCCAGACTTGATCCTGCTACAGTACGTGGATGACTTACTGCTGGCCGCCACTTCTG
AGCTAGACTGCCAACAAGGTACTCGGGCCCTGTTGCAAACCCTAGAGAACCTCGGGTATCGGGCCTCGGCCAAGAAAGCC
CAAATTTGCCAGAAACAGGTCAAGTATCTGGGGTATCTTCTAAAAGAGGGTCAGAGATGGCTGACTGAGGCCAGAAAAGA
GACTGTGATGGGGCAGCCTACTCCGAAGACCCCTCGACAACTAAGGGAGTTCCTAGGGACGGCAGGCTTCTGTCGCCTCT
GGATCCCTGGGTTTGCAGAAATGGCAGCCCCCTTGTACCCTCTCACCAAAACGGGGACTCTGTTTAATTGGGGCCCAGAC

CAGCAAAAGGCCTATCAAAAAATCAAACAGGCTCTTCTAACTGCCCCAGCCCTGAGATTGCCAGATTTGACTAAGCCCTT
TAAACTCTTTGTCGACGAGAAGCAGGGCTACGCCAAAGGCGTCCTAACGCAAAAACTGGGACCTTGGCGTCGGCCGGTGG
CCTACCTGTCCAAAAAGCTAGACCCAGTGGCAGCTGGGTGGCCCCCTTGCCTACGGATGGTAGCAGCCATTGCCGTTCTG
ACAAAAGATGCAGGCAAGCTAACCATGGGACAGCCGCTAGTCATCCTGGCCCCCCATGCAGTAGAGGCACTAGTCAAGCA
ACCCCCGGATCGCTGGCTATCCAACGCCCGCATGACCCACTATCAGGCGATGCTCCTAGATACGGACCGGGTCCAGTTCG
GACCGGTGGTAGCCCTAAACCCGGCTACGTTGCTCCCCCTACCGGGAAAAGAGCCTCACCATGACTGCCTCGAGATCTTG
GCCGAGACACACGGAACCAGACCAGACCTCACGGACCAGCCCCTCCCAGACGCCGACCACACCTGGTATACAGATAAAAG
CAGCTTCCTGCAAGAGGGACAACGTAGGGCTAGAGCAGCGGTGACCACCGAGACCGAGGTAATCTGGGCCAAGGCATTGC
CAGCCGGGACATCCGCCCAGCGAGCTGAACTAATAGCACTCACCCAGGCCCTAAAGATGGCAGAAGGTAAAAAGCTAAAT
GTTTATACTGATAGCCGCTATGCCTTTGCTACCGCCCATGTCCATGGAGAAATATATAAGAGACGTGGGTTGCTCACCTC
AGAAGGCAAGGAGATCAAGAACAAGGGCGAAATCTTGGCCTTACTGAAAGCTCTCTTTCTGCCCAAAAGACTCAGTATAA
TTCACTGCCCAGGACATCAGAAAGGCAATAGTGCTGAAGCTAAAGGCAACCGAATGGCGGACCAGGCAGCCCGGGAAGCA
GCCATGGGGACTAACACAAAGGCCTCCTCACTTCTCATAGAGACCTCAACCCCGTACACTCCAGACTTCTTCCATTATAC
TGAGACAGATATAAAGAACCTACAGGAGTTGGGAGCCACATATGATAGAGAAAAAAAATATTGGGTCCTGCAAAGTAAAC
CTGTGATGCCTGACCAGTTCACCTTTAAATTATTAGACTTCCTTCACCAGCTCACCCACCTTAGCTATCAGAAGATAAGG
GCACTTCTAGACAAGAAAGAAAGCCCCTATTACATGCTAAATAAAGATAAGATCCTCCACGAAGTGGCAGAATCATGCCA
AGCCTGTGTCCAAGTAAATGCCAGTAAGACTAAGATCAGGGCCGGAACACGAATAAGAGGACATCGACCAGGCACCCATT
AAAAAATTGACTTTACTGAAGTAAAGCCCGGACTGTATGGGTACAAGTATCTCCTGGTATTCGTGAACACGTTCTCTGGC
TGGGTTAAAGCCTTCCCAACCAAACATGAGACTGCCAAAATAGTGACCAAGAAACTTCTGAAAAAAATATTTCCAAAGTT
TAAAATGCCCCAAGTGTTGGGGACTGATAATGGGCCTGCCTTCGTCTCCCAGGTAAGTCAGTCGGTGGCCAAGCTACTGG
GGATTGATTAAAAACTACATTGTGCTTACAGACCCCAGAGTTCAGGTCAGGTAAAAAAAATAAATAAGACAATCAAGGAG
ACTTTGACCAAATTAACGCTTGCAACTGGCACTAGAGACTGGGTACTCCTACTTCCCTTAGCCCTCTACCGAGCCCGCAA
CACTCCGGGCCCCCATAGACTCACTCCGTATAAAATCCTGTATGGGGCGCCCCCGCCCCTTGTTAATTTCCATGATCCTG
AAATGTCAAAGTTTACTAATAGCCCCTCTCTCCAAGCTCACTTACAGGCCCTCCAAGCAGTACAACGAGAGGTCTAGAAG
CCACTGGCCGCTGCCTATCAGGACCAGCTGGACCAGCCAGTAATACCACACCCCTTCCGTGTCGGCGACACCGTGTGGGT
ACGCCGGCACCAGACTAAGAACTTAGAACCTCGCTAAAAAGGACCCTACACCGTCCTGCTGACCACCCCCACCGCTCTCA
AAGTAGACGGCATCGCTGCGTGGATCCACGCCGCTCACGTAAAAGCGGCGACAACCCCTCCGGCCGGAACAGCATCAGGA
CCGACATAAAAAGTCCAGCGTTCTCAAAACCCCTTAAAGATAAGATTAACCCGTGGGGCCCCCTAATAGTCCTGGGGATC
TTAATAAGGGCAGGAGTATCAGTACAACATGACAGCCCTCATCAGGTCTTCAATGTTACTTAAAAAGTTACCAACTTAAT
GACAGGACAAACAGCTAATGCTACCTCCCTCCTGGGGACAATGACCGATGCCTTTCCTAAACTGTACTTTGACTTGTGCG
ATTTAATAGGGGACGACTGAGATGAGACTGGACTCGGGTGTCGCACTCCCGGGGAAAAAAAAAGGGCAAGAACATTTGAC
TTCTATGTTTGCCCCGGGCATACTGTACCAACAGGGTGTAGAGGGCCGAAAAAGGGCTACTGTGGCAAATGGGGCTGTGA
GACCACTGGACAGGCATACTGAAAGCCATCATCATCATGGGACCTAATTTCCCTTAAGCGAAGAAACACCCCTCAGAATC
AGGGCCCCTGTTATGATTCCTCAGCGGTCTCCAGTGACATCAAGGGCGCCACACCGGGGGGTCGATGCAATCCCCTAGTC
CTAAAATTCACTGACGCGGGCAAAAAGGCCAGCTGGGATGGCCCCAAAGTATAGAGACTAAGACTGTACCGATCCACAGG
GACCGACCCGGTGACCCGGTTCTCTTTGACCCGCCAGGTCCTCAATATAGGGCCCCGCGTCCCCATTGGGCCTAATCCCG
TGATCACTGACCAGTTACCCCCCTCCCGACCCGTGCAGATCATGCTCCCCAGGCCTCCTCAGCCTCCTCCTCCAGGCGCA
GCCTCTATAGTCCCTGAGACTGCCCCACCTTCTCAACAACCTGGGACGAGAGACAGGCTGCTAAACCTGGTAAATAGAGC
CTACCAAGCTCTCAACCTCACCAGTCCTGACAAAACCCAAGAGTGCTGGTTGTGTCTGGTAGCGGACCCCCCTACTACG
AAGGGGTTGCCGTCCTAGGTACTTATTCCAACCATACCTCTGCCCCAGCTAACTGCTCCGTGGCCTCCCAACACAAGCTG
ACCCTGTCCAAAGTGACCGGACAGGGACTCTGCGTAAGAGCAGTTCCCAAAACCCATCAGGCCCTGTGTAATACCACCCA
AAAGACGAGCGACGGGTCCTACTATCTGGCTGCTCCCGCCGAGACCATTTAGGCTTGCAACACCGGGCTCACTCCCTGCC
TATCTACCACTGTACTCGACCTCACCACCGATTACTGTGTCCTGGTTGAGCTCTGGCCAAAGGTGACCTACCACTCCCCT
AGTTATGTTTATGGCCAGTTTGAAAAAAAAACCAAATATAAAAGAGAGCCGGTGTCATTAACTCTGGCCCTGCTGTTAAG
AAGACTTACTATGGGCGGCATAGCTGCAGGAGTAAAAACCGGGACTACAGCCCTAGTGGCCACCAAACAATTCGAGCAGC
TCCAGGCAGCCATACATACAGACCTTGGGGCTTTAAAAAAGTCAGTCAGTGCCCTAAAAAAGTCTCTGACCTCGTTGTCT
GAGGTGGTCCTACAGAACCGGAGGGGATTAGATCTACTGTTCCTAAAAAAAAGGAAGATTATGTGCTGCCCTAAAAAAAAA
ATGCTGTTTCTACGCGGACCACACTGGCGTAGTAAGAGATAGCATGGCAAAGCTAAAAAAAAAGGTTAAACCAGAGACAAA
AATTGTTCGAATCAGGACAAGGGTGGTTTAAGGGACTGTTTAACAGGTCCCCATGGTTCACGACCTTAATATCCACCATT
ATGGGCCCCTTGATAATACTTTTATTAATCCTACTCTTCGGACCCTGTATTCTCAACCGCTTGGTCCAGTTTGTAAAAGA
CAAAATTTCGGTAGTGCAGGCCCTGGTTTTGACCCAACAGTATCACCAACTCAAATCAATAGATCCAGAAAAAGTAAAAT
CACGTAAATAAAAGATTTTATTCAGTTTCCAGAAAGAGGGGGGAATGAAAGACCCCACCATCAGGCTTAGCAAGCTAGCT
GCAGTAACGCCATTTTGCAAGGCATGAAAAAGTACCAGAGCTGAGTTCTCAAAAGTTACAAGAAAGTTCAGTTAAAGATT
AACAGTTAAAGATTAAGGCTGAATAATACTGGGACAGGGGCCAAATATCGGTGGTCAAGCACCTGGGCCCCGGCTCAGGG
CCAAGAACAGATGGCTCTCAGACGTCAGTGTTAGCAGAACTAGCTTCACTGATTTAAAAAAATAGAGGTGCACAGTGCTC
TGGCCACTCCTTAAACCTGTGTGTCTGCCAATGTTCTGACCAGGTGTGTGCCCATTGTTGAACCTTCATTAGACCCTTTC
CTCGTACCCCTCCCATACCCATTTCTTGAAAATAGACATTGTTTAGAACTAAAAAGTCCCACCTCAGTTTCCCCAAATGA

CCGAGAAATACCCCAAGCCTTATTCAAACTAACCAACCAGCTCGCTTCTCGCTTCTGTAACCGCGCTTTTTGCTCCCCAG
CCCCAGCCCTATAAAAAGGGTAAAAACTCCACACTCGGCGCGCCAGTCCTCCGATAGACTGCGTCGCCCGGGTACCCGTG
TTCCCAATAAAGCCTCTTGCTGTTTACATCCGAATCGTGGTCTCGCTGGTCCTTGAGAGGGTCTCCTCAGATTGATTGAC
TACCCACGTCGGGGGTCTTTCA
>Pmv5
TGAAAGACCCCACCATCAGGCTTAGCAAGCTAGCTGCAGTAACGCCATTTTGCAAGGCATGAAAAAGTACCAGAGCTGAG
TTCTCAAAAGTTACAAGAAAGTTCAGTTAAAGATTAACAGTTAAAGATTAAGGCTGAATAATACTGGGACAGGGGCCAAA
TATCGGTGGTCAAGCACCTGGGCCCCGGCTCAGGGCCAAGAACAGATGGCTCTCAGACGTCAGTGTTAGCAGAACTAGCT
TCACTGATTTAGAAAAATAGAGGTGCACAGTGCTCTGGCCACTCCTTGAACCTGTGTGTCTGCCAATGTTCTGACCAGGT
GTGTGCCCATTGTTGAACCTTCATTAGACCCTTTCCTCGTACCCCTCCCATACCCATTTCTTGAAAATAGACATTGTTTA
GAACTAAAAAGTCCCACCTCAGTTTCCCCAAATGACCGAGAAATACCCCAAGCCTTATTCGAACTAACCAACCAGCTCGC
TTCTCGCTTCTGTAACCGCGCTTTTTGCTCCCCAGCCCCAGCCCTATAAAAAGGGTAAAAACTCCACACTCGGCGCGCCA
GTCCTCCGATAGACTGCGTCGCCCGGGTACCCGTGCTCCCAATAAAGCCTCTTGCTGTTTGCATCCGAATCGTGGTCTCG
CTGGTCCTTGAGAGGGTCTCCTCAGATTGATTGACTACCCACGTCGGGGGTCTTTCATTTGGAGGTCCCACCGAGATTTG
GAGACCCCTGCCCAGGGACCACCGACCCCCCGCCGGGAGGTAAGCTGGCCAGCGGTCGTTTCGTGTCTGTCTCTGTCTTC
GTGCGTGTTTGTGCCGGCATCCAATGTTTGCGCCTGCGTCTGTACTAGTTAGCTAACTAGATCTGTATCTGGCGGTTCCG
CGGAAGAACTGACGAGTTCGTATTCCCGGCCGCAGCCCCTGGGAGACGTCCCAGCGGCCTCGGGGGCCCGTTTTGTGGCC
CATTCTGTATCAGTTAACCTACCCGAGTCGGACTTTTTGGAGCTCCGCCACTGTACGTGGCTTTGTTGGGGGACGAGAGA
CAGAGACACTTCCCGCCCCCGTCTGAATTTTTGCTTTCGGTTTTACGCCGAAGCCGCGCCGCGCGTCTGATTTGTTTGTT
GTTCTTTTGTTCTTCGTTAGTTTTCTTCTGTCTTTAAGTGTTTTCGAGATCATGGGACAGACCGTAACTACCCCTCTGAG
TTTAACCTTGCAGCACTGGGGAGATGTCCAGCGCATTGCATCCAACCAGTCTGTGGATGTCAGGAAGAGGCGCTGGATTA
CCTTCTGTTCCGCTGAATGGCCAACTTTCAATGTGGGATGGCCTCAGGATGGTACTTTCAATTTAAGTATTATCTCTCAG
GTTAAGTCTAGAGTGTTTTGTCCTGGTCCCCACGGACACCCGGATCAGGTCCCATATATCGTCACCTGGGAGGCACTTGC
CTATGACCCCCCTCCGTGGGTCAAACCGTTTGTGTCTCCTAAACTTCCTCCCTTGCCGACAGCTCCCGTCCTCCCGCCCG
GTCCTTCTGCGCAACCTCCGTCCCGATCTGCCCTTTACCCTGCCCTTACCCCCTCTATAAAGTCCAAACCTCCTAAGCCC
CAGGTTCTCCCTGATAGCGGCGGACCCCTCATTGACCTTCTCACAGAGGACCCCCCGCCGTACAGAGCACAACCCTCCTC
CTCTGCCAGGGAGAACGACGAAGAAGAGGCGGCCACCACCTCCGAGGTTTCCCCCCCCTTCTCCCATGGTGTCTCGACTG
CGGGGAAGGAGAGACCCTCCCGCAGTGGACTCCACCACCTCCCAGGCATTTCCACTCCGCATGGGGGGAGATGGCCAGCT
TCAGTATTGGCCGTTTTCCTCTTCGGATTTATACAATTGGAAAAATAATAACCCTTCCTTTTCTGAAGATCCAGGTAAAT
TGACGGCCTTGATTGAGTCCGTCCTCATCACCCACCAGCCCACCTGGGACGACTGTCAGCAGTTGTTGGGGACCCTGCTG
ACCGGAGAAGAAAAGCAGCGGGTGCTCCTAGAGGCTAGAAAGGCAGTCCGGGGCAATGATGGACGCCCCACTCAGTTGCC
TAATGAAGTCAATGCTGCTTTTCCCCTTGAACGCCCCGGTTGGGATTACACCACTACAGAAGGTAGGAACCACCTAGTCC
TCTATCGCCAGTTGCTCTTAGCGGGTCTCCAGAACGCGGGCAGAAGTCCCACCAATTTGGCCAAGGTAAAAGGGATAACC
CAGGGACCTAATGAGTCTCCCTCAGCCTTTTTAGAGAGACTCAAAGAGGCCTATCGCAGGTACACTCCTTATGACCCTGA
GGACCCAGGGCAAGAAACCAATGTGTCTATGTCATTCATCTGGCAGTCTGCCCCGGATATCGGGCGAAAGTTAGAGCGGT
TAGAAGATTTAAAGAGCAAGACCTTAGGAGACTTAGTGAGGGAAGCTGAAAAGATCTTTAATAAGCGAGAAACCCCGGAA
GAAAGAGAGGAACGTATCAAGAGAGAAACAGAGGAAAAAGAAGAACGCCGTAGGGCAGAGGATGAGCAGAGAGAGAAAGA
AAGGGACCGCAGAAGACATAGAGAGATGAGCAAGCTCTTGGCCACTGTAGTTATTGGTCAGAGACAGGATAGACAGGGGG
GAGAGCGGAGGAGGCCCCAACTTGATAAGGACCAATGCGCCTACTGCAAAGAAAAGGGACACTGGGCTAAGGACTGCCCA
AAGAAGCCACGAGGGCCCCGAGGACTGAGGCCCCAGACCTCCCTCCTAACCTTAGGTGACTAGGGAGGTCAGGGTCAGGA
GCCCCCCCCTGAACCCAGGATAACCCTCAAAGTCGGGGGGCAACCCGTCACCTTCCTGGTAGATACTGGGGCCCAACACT
CCGTGCTGACCCAAAATCCTGGACCCCTAAGTGACAAGGCTGCCTGGGTCCAAGGGGCTACTGGAGGAAAGCGGTATCGC
TGGACCACGGATCGCAAAGTACATCTAGCTACCGGTAAGGTCACCCACTCTTTCCTCCATGTACCAGACTGCCCCTATCC
TCTGCTAGGAAGAGACTTGCTGACTAAACTAAAAGCCCAAATCCACTTCGAGGGATCAGGAGCTCAGGTTATGGGACCAA
TGGGACTGCCCCTGCAAGTGCTGACCCTAAACATAGAAGATGAGTATCGGCTACATGAGACCTCAAAAGAGCCGGATGTT
TCTCTAGGGTCCACCTGGCTTTCTGATTTTCCCCAGGTCTGGGCGGAAACCGGGGGCATGGGACTGGCAGTTCGCCAAGC
TCCTCTGATCATACCTCTGAAGGCAACCTCTACCCCCGTGTCCATAAAACAATACCCCATGTCACAAGAAGCCAGACTGG
GGATCAAGCCCCACATACAGAGACTGTTGGACCAGGGAATACTGGTACCCTGCCAGTCCCCCTGGAACACGCCCCTGCTA
CCCGTTAAGAAACCAGGGACTAATGATTATAGGCCTGTCCAGGATCTGAGAGAAGTCAACAAGCGGGTGGAAGACATCCA
CCCCACCGTGCCCAACCCTTACAACCTCTTGAGCGGGCTCCCACCGTCCCACCAGTGGTACACTGTGCTTGATTTAAAGG
ATGCCTTTTTCTGCCTGAGACTCCACCCCACCAGTCAGCCTCTCTTCGCCTTTGAGTGGAGAGATCCAGAGATGGGAATC
TCAGGACAATTGACCTGGACCAGACTCCCACAGGGTTTCAAAAACAGTCCCACCCTGTTTGATGAGGCACTGCACAGAGA
CCTAGCAGACTTCCGGATCCAGCACCCAGACTTGATCCTGCTACAGTACGTGGATGACTTACTGCTGGCCGCCACTTCTG
AGCTCGACTGCCAACAAGGTACTCGGGCCCTGTTACAAACCCTAGGGGACCTCGGGTATCGGGCCTCGGCCAAGAAAGCC
CAAATTTGCCAGAAACAGGTCAAGTATCTGGGGTATCTTCTAAAAGAGGGTCAGAGATGGCTGACTGAGGCCAGAAAAGA
GACTGTGATGGGGCAGCCTACTCCGAAGACCCCTCGACAACTAAGGGAGTTCCTAGGGACGGCAGGCTTCTGTCGCCTCT
GGATCCCTGGGTTTGCAGAAATGGCAGCCCCCTTGTACCCTCTCACCAAAACGGGGACTCTGTTTAATTGGGGCCCAGAC

CAGCAAAAGGCCTATCAAGAAATCAAACAGGCTCTTCTAACTGCCCCAGCCCTGGGATTGCCAGATTTGACTAAGCCCTT
TGAACTCTTTGTTGACGAGAAGCAGGGCTACGCCAAAGGCGTCCTAACGCAAAAACTGGGACCTTGGCGTCGGCCGGTGG
CCTACCTGTCCAAAAAGCTAGACCCAGTGGCAGCTGGGTGGCCCCCTTGCCTACGGATGGTAGCAGCCATTGCCGTTCTG
ACAAAAGATGCAGGCAAGCTAACCATGGGACAGCCGCTAGTCATCCTGGCCCCCCATGCAGTAGAGGCACTAGTCAAGCA
ACCCCCTGACCGCTGGCTATCCAACGCCCGCATGACCCACTATCAGGCGATGCTCCTAGATACGGACCGGGTCCAGTTCG
GACCGGTGGTAGCCCTAAACCCGGCTACGTTGCTCCCCCTACCGGGGAAAGAGCCTCACCATGACTGCCTCGAGATCTTG
GCCGAGACACACGGAACCAGACCAGACCTCACGGACCAGCCCCTCCCAGACGCCGACCACACCTGGTATACAGATGGAAG
CAGCTTCCTGCAAGAGGGACAACGTAGGGCTGGAGCAGCGGTGACCACCGAGACCGAGGTAATCTGGGCCAAGGCGTTGC
CAGCCGGGACATCCGCCCAGCGAGCTGAACTAATAGCACTCACCCAGGCCCTAAAGATGGCAGAAGGTAAGAAGCTAAAT
GTTTATACTGATAGCCGCTATGCCTTTGCTACCGCCCATATCCATGGAGAAATATATAGGAGACGTGGGTTGCTCACCTC
AGAAGGCAAGGAGATCAAGAACAAGGGCGAAATCTTGGCCTTACTGAAAGCTCTCTTTCTGCCCAAAAGACTCAGTATAA
TTCACTGCCCAGGACATCAGAAAGGCAATAGTGCTGAAGCTAAAGGCAACCGAATGGCGGACCAGGCAGCCCGGGAAGCG
GCCATGGGGACTGACACAAAGGCCTCCTCACTTCTCATAGAGACCTCAACCCCGTACACTCCAGACTTCTTCCATTATAC
TGAGACAGATATAAAGAACCTACAAGAGTTGGGAGCCACATATGATAGAGAGAAAAAATATTGGGTCCTGCAAGGTAAAC
CTGTGATGCCTGACCAGTTCACCTTTGAATTATTAGACTTCCTTCACCAGCTCACCCACCTTAGCTATCAGAAGATGAGG
GCACTTCTAGACAGGAAAGAAAGCCCCTATTACATGCTAAATAAAGATAAGATCCTCCACGAGGTGGCGGAATCATGCCA
AGCCTGTGTCCAAGTAAATGCCAGTAAGACTAAGATCAGGGCCGGAACACGAGTAAGAGGACATCGACCAGGCACCCATT
GGGAAATTGACTTTACTGAAGTGAAGCCCGGACTGTATGGGTACAAGTATCTCCTGGTATTCGTGGACACGTTCTCTGGC
TGGGTTGAAGCCTTCCCAACCAAACATGAGACTGCCAAAATAGTGACCAAGAAACTTCTGGAAGAAATATTTCCAAGGTT
TGGAATGCCCCAAGTGTTGGGGACTGATAATGGGCCTGCCTTCGTCTCCCAGGTAAGTCAGTCGGTGGCCAAGCTACTGG
GGATTGATTGGAAACTACATTGTGCTTACAGACCCCAGAGTTCAGGTCAGGTAGAAAGAATGAATAGGACAATCAAGGAG
ACTTTGACCAAATTAACGCTTGCAACTGGCACTAGAGACTGGGTACTCCTACTTCCCTTAGCCCTCTACCGAGCCCGCAA
CACTCCGGGCCCCCATGGACTCACTCCGTATGAAATCCTGTATGGGGCGCCCCCGCCCCTTGTTAATTTCCATGATCCTG
AAATGTCAAAGTTTACTAATAGCCCCTCTCTCCAAGCTCACTTACAGGCCCTCCAAGCAGTACAACGAGAGGTCTGGAAG
CCACTGGCCGCTGCCTATCAGGACCAGCTGGACCAGCCAGTGATACCACACCCCTTCCGTGTCGGCGACACCGTGTGGGT
ACGCCGGCACCAGACTAAGAACTTGGAACCTCGCTGGAAAGGACCCTACACCGTCCTGCTGACCACCCCCACCGCTCTCA
AAGTAGACGGCATCGCTGCGTGGATCCACGCCGCTCACGTAAAAGCGGCGACAACCCCTCCGGCCGGAACAGCATCAGGA
CCGACATGGAAGGTCCAGCGTTCTCAAAACCCCTTAAAGATAAGATTAACCCGTGGGGCCCCCTAATAATCCTGGGGATC
TTAATAAGAGCAGGAGTATCAGTACAACATGACAGCCCTCATCAGGTCTTCAATGTTACTTGGAGAGTTACCAACTTAAT
GACAGGACAAACAGCTAATGCTACCTCCCTCCTGGGGACAATGACCGATGCCTTTCCTAAACTGTACTTTGACTTGTGCG
ATTTAATAGGGGACGACTGGGATGAGACTGGACTCGGGTGTCGCACTCCCGGGGGAAGAAAAAGGGCAAGAACATTTGAC
TTCTATGTTTGCCCCGGGCATACTGTACCAACAGGGTGTGGAGGGCCGAGAGAGGGCTACTGTGGCAAATGGGGCTGTGA
GACCACTGGACAGGCATACTGGAAGCCATCATCATCATGGGACCTAATTTCCCTTAAGCGAGGAAACACCCCTCGGAATC
AGGGCCCCTGTTATGATTCCTCAGCGGTCTCCAGTGACATCAAGGGCGCCACACCGGGGGGTCGATGCAATCCCCTAGTC
CTGGAATTCACTGACGCGGGCAAAAAGGCCAGCTGGGATGGCCCCAAAGTATGGGGACTAAGACTGTACCGATCCACAGG
GACCGACCCGGTGACCCGGTTCTCTTTGACCCGCCAGGTCCTCAATATAGGGCCCCGCGTCCCCATTGGGCCTAATCCCG
TGATCACTGACCAGTTACCCCCCTCCCGACCCGTGCAGATCATGCTCCCCAGGCCTCCTCAGCCTCCTCCTCCAGGCGCA
GCCTCTATAGTCCCTGAGACTGCCCCACCTTCTCAACAACCTGGGACGGGAGACAGGCTGCTAAACCTGGTAGATGGAGC
CTACCAAGCTCTCAACCTCACCAGTCCTGACAAAACCCAAGAGTGCTGGTTGTGTCTGGTAGCGGGACCCCCCTACTACG
AAGGGGTTGCCGTCCTAGGTACTTATTCCAACTATACCTCTGCCCCAGCTAACTGCTCCGTGGCCTCCCAACACAAGCTG
ACCCTGTCCGAAGTGACCGGACAGGGACTCTGCGTAGGAGCAGTTCCCAAAACCCATCAGGCCCTGTGTAATACCACCCA
GAAGACGAGCGACGGGTCCTACTATCTGGCTGCTCCCGCCGGGACCATTTGGGCTTGCAACACCGGGCTCACTCCCTGCC
TATCTACCACTGTACTCGACCTCACCACCGATTACTGTGTCCTGGTTGAGCTCTGGCCAAAGGTGACCTACCACTCCCCT
GGTTATGTTTATGGCCAGTTTGAGAAAAAAACCAAATATAAAAGAGAGCCGGTGTCATTAACTCTGGCCCTGCTGTTGGG
AGGACTTACTATGGGCGGCATAGCTGCAGGAGTAGGAACCGGGACTACAGCCCTAGTGGCCACCAAACAATTCGAGCAGC
TCCAGGCAGCCATACATACAGACCTTGGGGCTTTAGAAAAGTCAGTCAGTGCCCTAGAAAAGTCTCTGACCTCGTTGTCT
GAGGTGGTCCTACAGAACCGGAGGGGATTAGATCTACTGTTCCTAAAAGAAGGAGGATTATGTGCTGCCCTAAAAGAAGA
ATGCTGTTTCTACGCGGACCACACTGGCGTAGTAAGAGATAGCATGGCAAAGCTAAGAGAAAGGTTGAACCAGAGACAAA
AATTGTTCGAATCAGGACAAGGGTGGTTTGAGGGACTGTTTAACAGGTCCCCATGGTTCACGACCTTAATATCTACCATT
ATGGGCCCCTTGATAATACTTTTATTAATCCTACTCTTCGGACCCTGTATTCTCAACCGCTTGGTCCAGTTTGTAAAAGA
CAGAATTTCGGTAGTGCAGGCCCTGGTTTTGACCCAACAGTATCACCAACTCAAATCAATAGATCCAGAAGAAGTGGAAT
CACGTGAATAAAAGATTTTATTCAGTTTCCAGAAAGAGGGGGGAATGAAAGACCCCACCATCAGGCTTAGCAAGCTAGCT
GCAGTAACGCCATTTTGCAAGGCATGAAAAAGTACCAGAGCTGAGTTCTCAAAAGTTACAAGAAAGTTCAGTTAAAGATT
AACAGTTAAAGATTAAGGCTGAATAATACTGGGACAGGGGCCAAATATCGGTGGTCAAGCACCTGGGCCCCGGCTCAGGG
CCAAGAACAGATGGCTCTCAGACGTCAGTGTTAGCAGAACTAGCTTCACTGATTTAGAAAAATAGAGGTGCACAGTGCTC
TGGCCACTCCTTGAACCTGTGTGTCTGCCAATGTTCTGACCAGGTGTGTGCCCATTGTTGAACCTTCATTAGACCCTTTC
CTCGTACCCCTCCCATACCCATTTCTTGAAAATAGACATTGTTTAGAACTAAAAAGTCCCACCTCAGTTTCCCCAAATGA

CCGAGAAATACCCCAAGCCTTATTCGAACTAACCAACCAGCTCGCTTCTCGCTTCTGTAACCGCGCTTTTTGCTCCCCAG
CCCCAGCCCTATAAAAAGGGTAAAAACTCCACACTCGGCGCGCCAGTCCTCCGATAGACTGCGTCGCCCGGGTACCCGTG
CTCCCAATAAAGCCTCTTGCTGTTTGCATCCGAATCGTGGTCTCGCTGGTCCTTGAGAGGGTCTCCTCAGATTGATTGAC
TACCCACGTCGGGGGTCTTTCA
>Pmv6
TGAAAGACCCCACCATCAGGCTTAGCAAGCTAGCTGCAGTAACGCCATTTTGCAAGGCATGAAAAAGTACCAGAGCTGAG
TTCTCAAAAGTTACAAGAAAGTTCAGTTAAAGATTAACAGTTAAAGATTAAGGCTGAATAATACTGGGACAGGGGCCAAA
TATCGGTGGTCAAGCACCTGGGCCCCGGCTCAGGGCCAAGAACAGATGGCTCTCAGACGTCAGTGTTAGCAGAACTAGCT
TCACTGATTTAGAAAAATAGAGGTGCACAGAGCTCTGGCCACTCCTTGAACCTGTGTGTCTGCCAATGTTCTGACCAGGT
GTGTGCCCATTGTTGAACCTTCATTAGACCCTCTCCTCGTACCCCTCCCATACCCATTTCTTGAAAATAGACATTGTTTA
GAACTAAAAAGTCCCACCTCAGTTTCCCCAAATGACCGAGAAATACCCCAAGCCTTATTCGAACTAACCAACCAGCTCGC
TTCTCGCTTCTGTAACCGCGCTTTTTGCTCCCCAGCCCCAGCCCTATAAAAAGGGTAAAAACTCCACACTCGGCGCGCCA
GTCCTCCGATAGACTGCGTCGCCCGGGTACCCGTGTTCTCAATAAAGCCTCTTGCTGTTTGCATCCGAATCGTGGTCTCG
CTGGTCCTTGAGAGGGTCTCCTCAGATTGATTGACTACCCACCTCGGGGGTCTTTCATTTGGAGGTCCCACCGAGATTTG
GAGACCCCTGCCCAGGGACCACCGACCCCCCGCCGGGAGGTAAGCTGGCCAGCGGTCGTTTCGTGTCTGTCTCTGTCTTC
GTGCGTGTTTGTGCCGGCATCCAATGTTTGCGCCTGCGTCTGTACTAGTTAGCTAACTAGATCTGTATCTGGCGGTTCCG
CGGAAGAACTGACGAGTTCGTATTCCCGGCCGCAGCCCCTGGGAGACGTCCCAGCGGCCTCGGGGGCCCGTTTTGTGGCC
CATTCTGTATCAGTTAACCTACCCGAGTCGGACTTTTTGGAGCTCCGCCACTGTACGTGGCTTTGTTGGGGGACGAGAGA
CAGAGACACTTCCCGCCCCCGTCTGAATTTTTGCTTTCGGTTTTACGCCGAAGCCGCGCCGCGCGTCTGATTTGTTTGTT
GTTCTTTTGTTCTTCGTTAGTTTTCTTCTGTCTTTAAGTGTTTTCGAGATCATGGGACAGACCGTAACTACCCCTCTGAG
TTTAACCTTGCAGCACTGGGGAGATGTCCAGCGCATTGCATCCAACCAGTCTGTGGATGTCAGGAAGAGGCGCTGGATTA
CCTTCTGTTCCGCTGAATGGCCAACTTTCAATGTGGGATGGCCTCAGGATGGTACTTTCAATTTAAGTATTATCTCTCAG
GTTAAGTCTAGAGTGTTTTGTCCTGGTCCCCACGGACACCCGGATCAGGTCCCATATATCGTCACCTGGGAGGCACTTGC
CTATGACCCCCCTCCGTGGGTCAAACCGTTTGTGTCTCCTAAACTTCCTCCCTTGCCGACAGCTCCCGTCCTCCCGCCCG
GTCCTTCTGCGCAACCTCCGTCCCGATCTGCCCTTTACCCTGCCCTTACCCCCTCTATAAAGTCCAAACCTCCTAAGCCC
CAGGTTCTCCCTGATAGCGGCGGACCCCTCATTGACCTTCTCACAGAGGACCCCCCGCCGTACAGAGCACAACCCTCCTC
CTCTGCCAGGGAGAACGACGAAGAAGAGGCGGCCACCACCTCCGAGGTTTCCCCCCCTTCTCCCATGGTGTCTCGACTGC
GGGGAAGGAGAGACCCTCCCGCAGTGGACTCCACCACCTCCCAGGCATTTCCACTCCGCATGGGGGGAGATGGCCAGCTT
CAGTATTGGCCGTTTTCCTCTTCGGATTTATACAATTGGAAAAATAATAACCCTTCCTTTTCTGAAGATCCAGGTAAATT
GATGGCCTTGATTGAGTCCGTCCTCATCACCCACCAGCCCACCTGGGACGACTGTCAGCAGTTGTTGGGGACCCTGCTGA
CCGGAGAAGAAAAGCAGCGGGTGCTCCTAGAGGCTAGAAAGGCAGTCCGGGGCAATGATGGACGCCCCACTCAGTTGCCT
AATGAAGTCAATGCTGCTTTTCCCCTTGAACGCCCCGGTTGGGATTACACCACTACAGAAGGTAGGAACCACCTAGTCCT
CTATCGCCAGTTGCTCTTAGCGGGTCTCCAGAACGCGGGCAGAAGTCCCACCAATTTGGCCAAGGTAAAAGGGATAACCC
AGGGACCTAATGAGTCTCCCTCAGCCTTTTTAGAGAGACTCAAAGAGGCCTATCGCAGGTACACTCCTTATGACCCTGAG
GACCCAGGGCAAGAAACCAATGTGTCTATGTCATTCATCTGGCAGTCTGCCCCGGATATCGGGCGAAAGTTAGAGCGGTT
AGAAGATTTAAAGAGCAAGACCTTAGGAGACTTAGTGAGGGAAGCTGAAAAGATCTTTAATAAGCGAGAAACCCCGGAAG
AAAGAGAGGAACGTATCAAGAGAGAAACAGAGGAAAAAGAAGAACGCCGTAGGGCAGAGGATGAGCAGAGAGAGAAAGAA
AGGGACCGCAGAAGACATAGAGAGATGAGCAAGCTCTTGGCCACTGTAGTTATTGGTCAGAGACAGGATAGACAGGGGGG
AGAGCGGAGGAGGCCCCAACTTGATAAGGACCAATGCGCCTACTGCAAAGAAAAGGGACACTGGGCTAAGGACTGCCCAA
AGAAGCCACGAGGGCCCCGAGGACTGAGGCCCCAGACCTCCCTCCTAACCTTAGGTGACTAGGGAGGTCAGGGTCAGGAG
CCCCCCCCTGAACCCAGGATAACCCTCAAAGTCGGGGGGCAACCCGTCACCTTCCTGGTAGATACTGGGGCCCAACACTC
CGTGCTGACCCAAAATCCTGGACCCCTAAGTGACAAGGCTGCCTGGGTCCAAGGGGCTACTGGAGGAAAGCGGTATCGCT
GGACCACGGATCGCAAAGTACATCTAGCTACCGGTAAGGTCACCCACTCTTTCCTCCATGTACCAGACTGCCCCTATCCT
CTGCTAGGAAGAGACTTGCTGACTAAACTAAAAGCCCAAATCCACTTCGAGGGATCAGGAGCTCAGGTTATGGGACCAAT
GGGACTGCCCCTGCAAGTGCTGACCCTAAACATAGAAGATGAATATCGGCTACATGAGACCTCAAAAGAGCCGGATGTTT
CTCTAGGGTCCACCTGGCTTTCTGATTTTCCCCAGGTCTGGGCGGAAACCGGGGGCATGGGACTGGCAGTTCGCCAAGCT
CCTCTGATCATACCTCTGAAGGCAACCTCTACCCCCGTGTCCATAAAACAATACCCCATGTCACAAGAAGCCAGACTGGG
GATCAAGCCCCACATACAGAGACTGTTGGACCAGGGAATACTGGTACCCTGCCAGTCCCCCTGGAACACGCCCCTGCTAC
CCGTTAAGAAACCAGGGACTAATGATTATAGGCCTGTCCAGGATCTGAGAGAAGTCAACAAGCGGGTGGAAGACATCCAC
CCCACCGTGCCCAACCCTTACAACCTCTTGAGCGGGCTCCCACCGTCCCACCAGTGGTACACTGTGCTTGATTTAAAGGA
TGCCTTTTTCTGCCTGAGACTCCACCCCACCAGTCAGCCTCTCTTCGCCTTTGAGTGGAGAGATCCAGAGATGGGAATCT
CAGGACAATTGACCTGGACCAGACTCCCACAGGGTTTCAAAAACAGTCCCACCCTGTTTGATGAGGCACTGCACAGAGAC
CTAGCAGACTTCCGGATCCAGCACCCAGACTTGATCCTGCTACAGTACGTGGATGACTTACTGCTGGCCGCCACTTCTGA
GCTCGACTGCCAACAAGGTACTCGGGCCCTGTTACAAACCCTAGGGAACCTCGGGTATCGGGCCTCGGCCAAGAAAGCCC
AAATTTGCCAGAAACAGGTCAAGTATCTGGGGTATCTTCTAAAAGAGGGTCAGAGATGGCTGACTGAGGCCAGAAAAGAG
ACTGTGATGGGGCAGCCTACTCCGAAGACCCCTCGACAACTAAGGGAGTTCCTAGGGACGGCAGGCTTCTGTCGCCTCTG
GATCCCTGGGTTTGCAGAAATGGCAGCCCCCTTGTACCCTCTCACCAAAACGGGGACTCTGTTTAATTGGGGCCCAGACC

AGCAAAAGGCCTATCAAGAAATCAAACAGGCTCTTCTAACTGCCCCAGCCCTGGGATTGCCAGATTTGACTAAGCCCTTT
GAACTCTTTGTTGACGAGAAGCAGGGCTACGCCAAAGGCGTCCTAACGCAAAAACTGGGACCTTGGCGTCGGCCGGTGGC
CTACCTGTCCAAAAAGCTAGACCCAGTGGCAGCTGGGTGGCCCCCTTGCCTACGGATGGTAGCAGCCATTGCCGTTCTGA
CAAAAGATGCAGGCAAGCTAACCATGGGACAGCCGCTAGTCATCCTGGCCCCCCATGCAGTAGAGGCACTAGTCAAGCAA
CCCCCTGACCGCTGGCTATCCAACGCCCGCATGACCCACTATCAGGCGATGCTCCTAGATACGGACCGGGTCCAGTTCGG
ACCGGTGGTAGCCCTAAACCCGGCTACGTTGCTCCCCCTACCGGGGAAAGAGCCTCACCATGACTGCCTCGAGATCTTGG
CCGAGACACACGGAACCAGACCAGACCTCACGGACCAGCCCCTCCCAGACGCCGACCACACCTGGTATACAGATGGAAGC
AGCTTCCTGCAAGAGGGACAACGTAGGGCTGGAGCAGCGGTGACCACCGAGACCGAGGTAATCTGGGCCAAGGCGTTGCC
AGCCGGGACATCCGCCCAGCGAGCTGAACTAATAGCACTCACCCAGGCCCTAAAGATGGCAGAAGGTAAGAAGCTAAATG
TTTATACTGATAGCCGCTATGCCTTTGCTACCGCCCATGTCCATGGAGAAATATATAGGAGACGTGGGTTGCTCACCTCA
GAAGGCAAGGAGATCAAGAACAAGGGCGAAATCTTGGCCTTACTGAAAGCTCTCTTTCTGCCCAAAAGACTCAGTATAAT
TCACTGCCCAGGACATCAGAAAGGCAATAGTGCTGAAGCTAAAGGCAACCGAATGGCGGACCAGGCAGCCCGGGAAGCAG
CCATGGGGACTGACACAAAGGCCTCCTCACTTCTCATAGAGACCTCAACCCCGTACACTCCAGACTTCTTCCATTATACT
GAGACAGATATAAAGAACCTACAAGAGTTGGGAGCCACATATGATAGAGAGAAAAAATATTGGGTCCTGCAAGGTAAACC
TGTGATGCCTGACCAGTTCACCTTTGAATTATTAGACTTCCTTCACCAGCTCACCCACCTTAGCTATCAGAAGATGAGGG
CACTTCTAGACAGGAAAGAAAGCCCCTATTACATGCTAAATAAAGATAAGATCCTCCACGAGGTGGCGGAATCATGCCAA
GCCTGTGTCCAAGTAAATGCCAGTAAGACTAAGATCAGGGCCGGAACACGAGTAAGAGGACATCGACCAGGCACCCATTG
GGAAATTGACTTTACTGAAGTGAAGCCCGGACTGTATGGGTACAAGTATCTCCTGGTATTCGTGGACACGTTCTCTGGCT
GGGTTGAAGCCTTCCCAACCAAACATGAAACTGCCAAAATAGTGACCAAGAAACTTCTGGAAGAAATATTTCCAAGGTTT
GGAATGCCCCAAGTGTTGGGGACTGATAATGGGCCTGCCTTCGTCTCCCAGGTAAGTCAGTCGGTGGCCAAGCTACTGGG
GATTGATTGGAAACTACATTGTGCTTACAGACCCCAGAGTTCAGGTCAGGTAGAAAGAATGAATAGGACAATCAAGGAGA
CTTTGACCAAATTAACGCTTGCAACTGGCACTAGAGACTGGGTACTCCTACTTCCCTTAGCCCTCTACCGAGCCCGCAAC
ACTCCGGGCCCCCATGGACTCACTCCGTATGAAATCCTGTATGGGGCGCCCCCGCCCCTTGTTAATTTCCATGATCCTGA
AATGTCAAAGTTTACTAATAGCCCCTCTCTCCAAGCTCACTTACAGGCCCTCCAAGCAGTACAACGAGAGGTCTGGAAGC
CACTGGCCGCTGCCTATCAGGACCAGCTGGACCAGCCAGTGATACCACACCCCTTCCGTGTCGGCGACACCGTGTGGGTA
CGCCGGCACCAGACTAAGAACTTGGAACCTCGCTGGAAAGGACCCTACACCGTCCTGCTGACCACCCCCACCGCTCTCAA
AGTAGACGGCATCGCTGCGTGGATCCACGCCGCTCACGTAAAAGCGGCGACAACCCCTCCGGCCGGAACAGCATCAGGAC
CGACATGGAAGGTCCAGCGTTCTCAAAACCCCTTAAAGATAAGATTAACCCGTGGGGCCCCCTAATAGTCCTGGGAATCT
TAATAAGGGCAGGAGTATCAGTACAACATGACAGCCCTCATCAGGTCTTCAATGTTACTTGGAGAGTTACCAACTTAATG
ACAGGACAAACAGCTAATGCTACCTCCCTCCTGGGGACAATGACCGATGCCTTTCCTAAACTGTACTTTGACTTGTGCGA
TTTAATAGGGGACGACTGGGATGAGACTGGACTCGGGTGTCGCACTCCCGGGGGAAGAAAAAGGGCAAGAACATTTGACT
TCTATGTTTGCCCCGGGCATACTGTACCAACAGGGTGTGGAGGGCCGAGAGAGGGCTACTGTGGCAAATGGGGCTGTGAG
ACCACTGGACAGGCATACTGGAAGCCATCATCATCATGGGACCTAATTTCCCTTAAGCGAGGAAACACCCCTCGGAATCA
GGGCCCCTGTTATGATTCCTCAGCGGTCTCCAGTGACATCAAGGGCGCCACACCGGGGGGTCGATGCAATCCCCTAGTCC
TGGAATTCACTAACGCGGGCAAAAAGGCCAGCTGGGATGGCCCCAAAGTATGGGGACTAAGACTGTACCGATCCACAGGG
ACCGACCCGGTGACCCGGTTCTCTTTGACCCGCCAGGTCCTCAATATAGGGCCCCGCGTCCCCATTGGGCCTAATCCCGT
GATCACTGACCAGTTACCCCCCTCCCGACCCGTGCAGATCATGCTCCCCAGGCCTCCTCAGCCTCCTCCTCCAGGCGCAG
CCTCTATAGTCCCTGAGACTGCCCCACCTTCTCAACAACCTGGGACGGGAGACAGGCTGCTAAACCTGGTAGATGGAGCC
TACCAAGCTCTCAACCTCACCAGTCCTGACAAAACCCAAGAGTGCTGGTTGTGTCTGGTAGCGGGACCCCCCTACTACGA
AGGGGTTGCCGTCCTAGGTACTTATTCCAACCATACCTCTGCCCCAGCTAACTGCTCCGTGGCCTCCCAACACAAGCTGA
CCCTGTCCGAAGTGACCGGACAGGGACTCTGCGTAGGAGCAGTTCCCAAAACCCATCAGGCCCTGTGTAATACCACCCAG
AAGACGAGCGACGGGTCCTACTATCTGGCTGCTCCCGCCGGGACCATTTGGGCCTGCAACACCGGGCTCACTCCCTGCCT
ATCTACCACTGTACTCGACCTCACCACCGATTACTGTGTCCTGGTTGAGCTCTGGCCAAAGGTGACCTACCACTCCCCTG
GTTATGTTTATGGCCAGTTTGAGAGAAAAACCAAATATAAAAGAGAGCCGGTGTCATTAACTCTGGCCCTGCTGTTGGGA
GGACTTACTATGGGCGGCATAGCTGCAGGAGTAGGAACCGGGACTACAGCCCTAGTGGCCACCAAACAATTCGAGCAGCT
CCAGGCAGCCATACATACAGACCTTGGGGCTTTAGAAAAGTCAGTCAGTGCCCTAGAAAAGTCTCTGACCTCGTTGTCTG
AGGTGGTCCTACAGAACCGGAGGGGATTAGATCTACTGTTCCTAAAAGAAGGAGGATTATGTGCTGCCCTAAAAGAAGAA
TGCTGTTTCTACGCGGACCACACTGGCGTAGTAAGAGATAGCATGGCAAAGCTAAGAGAAAGGTTGAACCAGAGACAAAA
ATTGTTCGAATCAGGACAAGGGTGGTTTGAGGGACTGTTTAACAGGTCCCCATGGTTCACGACCTTAATATCCACCATTA
TGGGCCCCTTGATAATACTTTTATTAATCCTACTCTTCGGACCCTGTATTCTCAACCGCTTGGTCCAGTTTGTAAAAGAC
AGAATTTCGGTAGTGCAGGCCCTGGTTTTGACCCAACAGTATCACCAACTCAAATCAATAGATCCAGAAGAAGTGGAATC
ACGTGAATAAAAGATTTTATTCAGTTTCCAGAAAGAGGGGGGAATGAAAGACCCCACCATCAGGCTTAGCAAGCTAGCTG
CAGTAACGCCATTTTGCAAGGCATGAAAAAGTACCAGAGCTGAGTTCTCAAAAGTTACAAGAAAGTTCAGTTAAAGATTA
ACAGTTAAAGATTAAGGCTGAATAATACTGGGACAGGGGCCAAATATCGGTGGTCAAGCACCTGGGCCCCGGCTCAGGGC
CAAGAACAGATGGCTCTCAGACGTCAGTGTTAGCAGAACTAGCTTCACTGATTTAGAAAAATAGAGGTGCACAGAGCTCT
GGCCACTCCTTGAACCTGTGTGTCTGCCAATGTTCTGACCAGGTGTGTGCCCATTGTTGAACCTTCATTAGACCCTCTCC
TCGTACCCCTCCCATACCCATTTCTTGAAAATAGACATTGTTTAGAACTAAAAGTCCCACCTCAGTTTCCCCAAATGAC

CGAGAAATACCCCAAGCCTTATTCGAACTAACCAACCAGCTCGCTTCTCGCTTCTGTAACCGCGCTTTTTGCTCCCCAGC
CCCAGCCCTATAAAAAGGGTAAAAACTCCACACTCGGCGCGCCAGTCCTCCGATAGACTGCGTCGCCCGGGTACCCGTGT
TCTCAATAAAGCCTCTTGCTGTTTGCATCCGAATCGTGGTCTCGCTGGTCCTTGAGAGGGTCTCCTCAGATTGATTGACT
ACCCACCTCGGGGGTCTTTCA
>Pmv7
TGAAAGACCCCACCATCAGGCTTAGCAAGCTAGCTGCAGTAACGCCATTTTGCAAGGCATGAAAAAGTACCAGAGCTGAG
TTCTCAAAAGTTACAAGAAAGTTCAGTTAAAGATTAACAGTTAAAGATTAAGGCTGAATAATACTGGGACAGGGGCCAAA
TATCGGTGGTCAAACACCTGGGCCCCGGCTCAGGGCCAAGAACAGATGGCTCTCAGACGTCAGTGTTAGCAGAACTAGCT
TCACTGATTTAAAAAAATAGAGGTGCACAGTGCTCTGGCCACTCCTTAAACCTGTGTGTCTGCCAATGTTCTGACCAGGT
GTGTGCCCATTGTTGAACCTTCATTAGACCCTTTCCTCGTACCCCTCCCATACCCATTTCTTGAAAATAGACATTGTTTA
GAACTAAAAAGTCCCACCTCAGTTTCCCCAAATGACCGAGAAATACCCCAAGCCTTATTCGAACTAACCAACCAGCTCGC
TTCTCGCTTCTGTAACCGCGCTTTTTGCTCCCCAGCCCCAGCCCTATAAAAAGGGTAAAAACTCCACACTCGGCGCGCCA
GTCCTCCGATAGACTGCGTCGCCCGGGTACCCGTGTTCTCAATAAAGCCTCTTGCTGTTTGCATCCGAATCGTGGTCTCG
CTGGTCCTTGAGAGGGTCTCCTCAGATTGATTGACTACCCACGTCGGGGGTCTTTCATTTGGAGGTTCCACCGAGATTTG
GAGACCCCTGCCCAGGGACCACCGACCCCCCGCCGGGAGGTAAGCTGGCCAGCGGTCGTTTCGTGTCTGTCTCTGTCTTC
GTGCGTGTTTGTGCCGGCATCCAATGTTTGCGCCTGCGTCTGTACTAGTTAGCTAACTAGATCTGTATCTGGCGGTTCCG
CGGAAGAACTGACGAGTTCGTATTCCCGGCCGCAGCCCCTGGGAGACGTCCCAGCGGCCTCGGGGGCCCGTTTTGTGGCC
CATTCTGTATCAGTTAACCTACCCGAGTCGGACTTTTTGGAGCTCCGCCACTGTACGTGGCTTTGTTGGGGGACGAGAGA
CAGAGACACTTCCCGCCCCCGTCTGAATTTTTGCTTTCGGTTTTACGCCGAAGCCGCGCCGCGCGTCTGATTTGTTTGTT
GTTCTTTTGTTCTTCGTTAGTTTTCTTCTGTCTTTAAGTGTTTTCGAGATCATGGGACAGACCGTAACTACCCCTCTGAG
TTTAACCTTGCAGCACTGGGGAGATGTCCAGCGCATTGCATCCAACCAGTCTGTGGATGTCAGGAAGAGGCGCTGGATTA
CCTTCTGTTCCGCTGAATGGCCAACTTTCAATGTGGGATGGCCTCAGGATGGTACTTTCAATTTAAGTATTATCTCTCAG
GTTAAGTCTAGAGTGTTTTGTCCTGGTCCCCACGGACACCCGGATCAGGTCCCATATATCGTCACCTGGGAGGCACTTGC
CTATGACCCCCCTCCGTGGGTCAAACCGTTTGTGTCTCCTAAACTTCCTCCCTTGCCGACAGCTCCCGTCCTCCCGCCCG
GTCCTTCTGCGCAACCTCCGTCCCGATCTGCCCTTTACCCTGCCCTTACCCCCTCTATAAAGTCCAAACCTCCTAAGCCC
CAGGTTCTCCCTGATAGCGGCGGACCCCTCATTGACCTTCTCACAGAGGACCCCCCGCCGTACAGAGCACAACCCTCCTC
CTCTGCCAGGGAGAACGACGAAGAAGAGGCGGCCACCACCTCCGAGGTTTCCCCCCCTTCTCCCATGGTGTCTCGACTGC
GGGGAAGGAGAGACCCTCCCGCAGTGGACTCCACCACCTCCCAGGCATTTCCACTCCGCATGGGGGGAGATGGCCAGCTT
CAGTATTGGCCGTTTTCCTCTTCGGATTTATACAATTGGAAAAATAATAACCCTTCCTTTTCTGAAGATCCAGGTAAATT
GACGGCCTTGATTGAGTCCGTCCTCATCACCCACCAGCCCACCTGGGACGACTGTCAGCAGTTGTTGGGGACCCTGCTGA
CCGGAGAAGAAAAGCAGCGGGTGCTCCTAGAGGCTAGAAAGGCAGTCCGGGGCAATGATGGACGCCCCACTCAGTTGCCT
AATGAAGTCAATGCTGCTTTTCCCCTTGAACGCCCCGGTTGGGATTACACCACTACAGAAGGTAGGAACCACCTAGTCCT
CTATCGCCAGTTGCTCTTAGCGGGTCTCCAGAACGCGGGCAGAAGTCCCACCAATTTGGCCAAGGTAAAAGGGATAACCC
AGGGACCTAATGAGTCTCCCTCAGCCTTTTTAGAGAGACTCAAAGAGGCCTATCGCAGGTACACTCCTTATGACCCTGAG
GACCCAGGGCAAGAAACCAATGTGTCTATGTCATTCATCTGGCAGTCTGCCCCGGATATCGGGCGAAAGTTAGAGCGGTT
AGAAGATTTAAAGAGCAAGACCTTAGGAGACTTAGTGAGGGAAGCTGAAAAGATCTTTAATAAGCGAGAAACCCCGGAAG
AAAGAGAGGAACGTATCAAGAGAGAAACAGAGGAAAAAGAAGAACGCCGTAGGGCAGAGGATGAGCAGAGAGAGAAAGAA
AGGGACCGCAGGAGACATAGAGAGATGAGCAAGCTCTTGGCCACTGTAGTTATTGGTCAGAGACAGGATAGACAGGGGGG
AGAGCGGAGGAGGCCCCAACTTGATAAGGACCAATGCGCCTACTGCAAAGAAAAGGGACACTGGGCTAAGGACTGCCCAA
AGAAGCCACGAGGGCCCCGAGGACTGAGGCCCCAGACCTCCCTCCTAACCTTAGGTGACTAGGGAGGTCAGGGTCAGGAG
CCCCCCCCTGAACCCAGGATAACCCTCAAAGTCGGGGGGCAACCCGTCACCTTCCTGGTAGATACTGGGGCCCAACACTC
CGTGCTGACCCAAAATCCTGGACCCCTAAGTGACAAGGCTGCCTGGGTCCAAGGGGCTACTGGAGGAAAGCGGTATCGCT
GGACCACGGATCGCAAAGTACATCTAGCTACCGGTAAGGTCACCCACTCTTTCCTCCATGTACCAGACTGCCCCTATCCT
CTGCTAGGAAGAGACTTGCTGACTAAACTAAAAGCCCAAATCCACTTCGAGGGATCAGGAGCTCAGGTTATGGGACCAAT
GGGACTGCCCCTGCAAGTGCTGACCCTAAACATAGAAGATGAGTATCGGCTACATGAGACCTCAAAAGAGCCGGATGTTT
CTCTAGGGTCCACCTGGCTTTCTGATTTTCCCCAGGTCTGGGCGGAAACCGGGGGCATGGGACTGGCAGTTCGCCAAGCT
CCTCTGATCATACCTCTGAAGGCAACCTCTACCCCCGTGTCCATAAAACAATACCCCATGTCACAAGAAGCCAGACTGGG
GATCAAGCCCCACATACAGAGACTGTTGGACCAGGGAATACTGGTACCCTGCCAGTCCCCCTGGAACACGCCCCTGCTAC
CCGTTAAGAAACCAGGGACTAATGATTATAGGCCTGTCCAGGATCTGAGAGAAGTCAACAAGCGGGTGGAAGACATCCAC
CCCACCGTGCCCAACCCTTACAACCTCTTGAGCGGGCTCCCACCGTCCCACCAGTGGTACACTGTGCTTGATTTAAAGGA
TGCCTTTTTCTGCCTGAGACTCCACCCCACCAGTCAGCCTCTCTTCGCCTTTGAGTGGAGAGATCCAGAGATGGGAATCT
CAGGACAATTGACCTGGACCAGACTCCCACAGGGTTTCAAAAACAGTCCCACCCTGTTTGATGAGGCACTGCACAGAGAC
CTAGCAGACTTCCGGATCCAGCACCCAGACTTGATCCTGCTACAGTACGTGGATGACTTACTGCTGGCCGCCACTTCTGA
GCTCGACTGCCAACAAGGTACTCGGGCCCTGTTACAAACCCTAGGGAACCTCGGGTATCGGGCCTCGGCCAAGAAAGCCC
AAATTTGCCAGAAACAGGTCAAGTATCTGGGGTATCTTCTAAAAGAGGGTCAGAGATGGCTGACTGAGGCCAGAAAAGAG
ACTGTGATGGGGCAGCCTACTCCGAAGACCCCTCGACAACTAAGGGAGTTCCTAGGGACGGCAGGCTTCTGTCGCCTCTG
GATCCCTGGGTTTGCAGAAATGGCAGCCCCCTTGTACCCTCTCACCAAAACGGGGACTCTGTTTAATTGGGGCCCAGACC

AGCAAAAGGCCTATCAAGAAATCAAACAGGCTCTTCTAACTGCCCCAGCCCTGGGATTGCCAGATTTGACTAAGCCTTTT
GAACTCTTTGTTGACGAGAAGCAGGGCTACGCCAAAGGCGTCCTAACGCAAAAACTGGGACCTTGGCGTCGGCCGGTGGC
CTACCTGTCCAAAAAGCTAGACCCAGTGGCAGCTGGGTGGCCCCCTTGCCTACGGATGGTAGCAGCCATTGCCGTTCTGA
CAAAAGATGCAGGCAAGCTAACCATGGGACAGCCGCTAGTCATCCTGGCCCCCCATGCAGTAGAGGCACTAGTCAAGCAA
CCCCCTGACCGCTGGCTATCCAACGCCCGCATGACCCACTATCAGGCGATGCTCCTAGATACGGACCGGGTCCAGTTCGG
ACCGGTGGTAGCCCTAAACCCGGCTACGTTGCTCCCCCTACCGGGGAAAGAGCCTCACCATGACTGCCTCGAGATCTTGG
CCGAGACACACGGAACCAGACCAGACCTCACGGACCAGCCCCTCCCAGACGCCGACCACACCTGGTATACAGATGGAAGC
AGCTTCCTGCAAGAGGGACAACGTAGGGCTGGAGCAGCGGTGACCACCGAGACCGAGGTAATCTGGGCCAAGGCGTTGCC
AGCCGGGACATCCGCCCAGCGAGCTGAACTAATAGCACTCACCCAGGCCCTAAAGATGGCAGAAGGTAAGAAGCTAAATG
TTTATACTGATAGCCGCTATGCCTTTGCTACCGCCCATGTCCATGGAAAAATATATAGGAGACGTGGGTTGCTCACCTCA
GAAGGCAAGGAGATCAAGAACAAGGGCGAAATCTTGGCCTTACTGAAAGCTCTCTTTCTGCCCAAAAGACTCAGTATAAT
TCACTGCCCAGGACATCAGAAAGGCAATAGTGCTGAAGCTAAAGGCAACCGAATGGCGGACCAGGCAGCCCGGGAAGCAG
CCATGGGGACTGACACAAAGGCCTCCTCACTTCTCATAGAGACCTCAACCCCGTACACTCCAGACTTCTTCCATTATACT
GAGACAGATATAAAGAACCTACAAGAGTTGGGAGCCACATATGATAGAGAGAAAAAATATTGGGTCCTGCAAGGTAAACC
TGTGATGCCTGACCAGTTCACCTTTGAATTATTAGACTTCCTTCACCAGCTCACCCACCTTAGCTATCAGAAGATGAGGG
CACTTCTAGACAGGAAAGAAAGCCCCTATTACATGCTAAATAAAGATAAGATCCTCCACGAGGTGGCGGAATCATGCCAA
GCCTGTGTCCAAGTAAATGCCAGTAAGACTAAGATCAGGGCCGGAACACGAGTAAGAGGACATCGACCAGGCACCCATTG
GGAAATTGACTTTACTGAAGTGAAGCCCGGACTGTATGGGTACAAGTATCTCCTGGTATTCGTGGACACGTTCTCTGGCT
GGGTTGAAGCCTTCCCAACCAAACATGAGACTGCCAAAATAGTGACCAAGAAACTTCTGGAAGAAATATTTCCAAGGTTT
GGAATGCCCCAAGTGTTGGGGACTGATAATGGGCCTGCCTTCGTCTCCCAGGTAAGTCAGTCGGTGGCCAAGCTACTGGG
GATTGATTGGAAACTACATTGTGCTTACAGACCCCAGAGTTCAGGTCAGGTAGAAAGAATGAATAGGACAATCAAGGAGA
CTTTGACCAAATTAACGCTTGCAACTGGCACTAGAGACTGGGTACTCCTACTTCCCTTAGCCCTCTACCGAGCCCGCAAC
ACTCCGGGCCCCCATGGACTCACTCCGTATGAAATCCTGTATGGGGCGCCCCCGCCCCTTGTTAATTTCCATGATCCTGA
AATGTCAAAGTTTACTAATAGCCCCTCTCTCCAAGCTCACTTACAGGCCCTCCAAGCAGTACAACGAGAGGTCTGGAAGC
CACTGGCCGCTGCCTATCAGGACCAGCTGGACCAGCCAGTGATACCACACCCCTTCCGTGTCGGCGACACCGTGTGGGTA
CGCCGGCACCAGACTAAGAACTTGGAACCTCGCTGGAAAGGACCCTACACCGTCCTGCTGACCACCCCCACCGCTCTCAA
AGTAGACGGCATCGCTGCGTGGATCCACGCCGCTCACGTAAAAGCGGCGACAACCCCTCCGGCCGGAACAGCATCAGGAC
CGACATGGAAGGTCCAGCGTTCTCAAAACCCCTTAAAGATAAGATTAACCCGTGGGGCCCCCTAATAATCCTGGGGATCT
TAATAAGGGCAGGAGTATCAGTACAACATGACAGCCCTCATCAGGTCTTCAATGTTACTTGGAGAGTTACCAACTTAATG
ACAGGACAAACAGCTAATGCTACCTCCCTCCTGGGGACAATGACCGATGCCTTTCCTAAACTGTACTTTGACTTGTGCGA
TTTAATAGGGGACGACTGGGATGAGACTGGACTCGGGTGTCGCACTCCCGGGGAAAGAAAAAGGGCAAGAACATTTGACT
TCTATGTTTGCCCCGGGCATACTGTACCAACAGGGTGTGGAGGGCCGAGAGAGGGCTACTGTGGCAAATGGGGCTGTGAG
ACCACTGGACAGGCATACTGGAAGCCATCATCATCATGGGACCTAATTTCCCTTAAGCGAGGAAACACCCCTCGGAATCA
GGGCCCCTGTTATGATTCCTCAGCGGTCTCCAGTGACATCAAGGGCGCCACACCGGGGGGTCGATGCAATCCCCTAGTCC
TGGAATTCACTGACGCGGGCAAAAAGGCCAGCTGGGATGGCCCCAAAGTATGGGGACTAAGACTGTACCGATCCACAGGG
ACCGACCCGGTGACCTGGTTCTCTTTGACCCGCCAGGTCCTCAATATAGGGCCCCGCGTCCCCATTGGGCCTAATCCCGT
GATCACTGACCAGTTACCCCCCTCCCGACCCGTGCAGATCATGCTCCCCAGGCCTCCTCAGCCTCCTCCTCCAGGCGCAG
CCTCTATAGTCCCTGAGACTGCCCCACCTTCTCAACAACCTGGGACGGGAGACAGGCTGCTAAACCTGGTAGATGGAGCC
TACCAAGCTCTCAACCTCACCAGTCCCGACAAAACCCAAGAGTGCTGGTTGTGTCTGGTAGCGGGACCCCCCTACTACGA
AGGGGTTGCCGTCCTAGGTACTTATTCCAACCATACCTCTGCCCCAGCTAACTGCTCCGTGGCCTCCCAACACAAGCTGA
CCCTGTCCGAAGTGACCGGACAGGGACTCTGCGTAGGAGCAGTTCCCAAAACCCATCAGGCCCTGTGTAATACCACCCAG
AAGACGAGCGACGGGTCCTACTATCTGGCTGCTCCCGCCGGGACCATTTGGGCTTGCAACACCGGGCTCACTCCCTGCCT
ATCTACCACTGTACTCGACCTCACCACCGATTACTGTGTCCTGGTTGAGCTCTGGCCAAAGGTGACCTACCACTCCCCTG
GTTATGTTTATGGCCAGTTTGAGAAAAAAACCAAATATAAAAGAGAGCCGGTGTCATTAACTCTGGCCCTGCTGTTGGGA
GGACTTACTATGGGCGGCATAGCTGCAGGAGTAGGAACCGGGACTACAGCCCTAGTGGCCACCAAACAATTCGAGCAGCT
CCAGGCAGCCATACATACAGACCTTGGGGCTTTAAAAAAGTCAGTCAGTGCCCTAAAAAAGTCTCTGACCTCGTTGTCTG
AGGTGGTCCTACAGAACCGGAGGGGATTAGATCTACTGTTCCTAAAAGAAGGAGGATTATGTGCTGCCCTAAAAGAAGAA
TGCTGTTTCTACGCGGACCACACTGGCGTAGTAAGAGATAGCATGGCAAAGCTAAGAGAAAGGTTGAACCAGAGACAAAA
ATTGTTCGAATCAGGACAAGGGTGGTTTGAGGGACTGTTTAACAGGTCCCCATGGTTCACGACCTTAATATCCACCATTA
TGGGCCCCTTGATAATACTTTTATTAATCCTACTCTTCGGACCCTGTATTCTCAACCGCTTGGTCCAGTTTGTAAAAGAC
AGAATTTCGGTAGTGCAGGCCCTGGTTTTGACCCAACAGTATCACCAACTCAAATCAATAGATCCAGAAGAAGTGGAATC
ACGTGAATAAAAGATTTTATTCAGTTTCCAGAAAGAGGGGGGAATGAAAGACCCCACCATCAGGCTTAGCAAGCTAGCTG
CAGTAACGCCATTTTGCAAGGCATGAAAAAGTACCAGAGCTGAGTTCTCAAAAGTTACAAGAAAGTTCAGTTAAAGATTA
ACAGTTAAAGATTAAGGCTGAATAATACTGGGACAGGGGCCAAATATCGGTGGTCAAACACCTGGGCCCCGGCTCAGGGC
CAAGAACAGATGGCTCTCAGACGTCAGTGTTAGCAGAACTAGCTTCACTGATTTAAAAAAATAGAGGTGCACAGTGCTCT
GGCCACTCCTTAAACCTGTGTGTCTGCCAATGTTCTGACCAGGTGTGTGCCCATTGTTGAACCTTCATTAGACCCTTTCC
TCGTACCCCTCCCATACCCATTTCTTGAAAATAGACATTGTTTAGAACTAAAAAGTCCCACCTCAGTTTCCCCAAATGAC

CGAGAAATACCCCAAGCCTTATTCGAACTAACCAACCAGCTCGCTTCTCGCTTCTGTAACCGCGCTTTTTGCTCCCCAGC
CCCAGCCCTATAAAAAGGGTAAAAACTCCACACTCGGCGCGCCAGTCCTCCGATAGACTGCGTCGCCCGGGTACCCGTGT
TCTCAATAAAGCCTCTTGCTGTTTGCATCCGAATCGTGGTCTCGCTGGTCCTTGAGAGGGTCTCCTCAGATTGATTGACT
ACCCACGTCGGGGGTCTTTCA
>Pmv8
TGAAAGACCCCACCATCAGGCTTAGCAAGCTAGCTGCAGTAACGCCATTTTGCAAGGCATGAAAAAGTACCAGAGCTGAG
TTCTCAAAAGTTACAAGAAAGTTCAGTTAAAGATTAAGGCTGAATAATACTGGGACAGGGGCCAAATATCGGTGGTCAAG
CACCTGGGCCCCGGCTCAGGGCCAAGAACAGATGGCTCTCAGACGTCAGTGTTAGCAGAACTAGCTTCACTGATTTAGAA
AAATAGAGGTGCACAGTGCTCTGGCCACTCCTTGAACCTGTGTGTCTGCCAATGTTCTGACCAGGTGTGTGCCCATTGTT
GAACCTTCATTAGACCCTTTCCTCGTACCCCTCCCACACCCATTTCTTGAAAATAGACATTGTTTAGAACTAAAAAGTCC
CACCTCAGTTTCCCCAAATGACCGAGAAATACCCCAAGCCTTATTCGAACTAACCAACCAGCTCGCTTCTCGCTTCTGTA
ACCGCGCTTTTTGCTCCCCAGCCCCAGCCCTATAAAAAGGGTAAAAACTCCACACTCGGCGCGCCAGTCCTCCGATAGAC
TGCGTCGCCCGGGTACCCGTGTTCTCAATAAAGCCTCTTGCTGTTTGCATCCGAATCGTGGTCTCGCTGGTCCTTGAGAG
GGTCTCCTCAGATTGATTGACTACCCACGTCGGGGGTCTTTCATTTGGAGGTTCCACCGAGATTTGGAGACCCCTGCCCA
GGGACCACCGACCCCCCGCCGGGAGGTAAGCTGGCCAGCGGTCGTTTCGTGTCTGTCTCTGTCTTCGTGCGTGTTTGTGC
CGGCATCCAATGTTTGCGCCTGCGTCTGTACTAGTTAGCTAACTAGATCTGTATCTGGCGGTTCCGCGGAAGAACTGACG
AGTTCGTATTCCCGGCCGCAGCCCCTGGGAGACGTCCCAGCGGCCTCGGGGGCCCGTTTTGTGGCCCATTCTGTATCAGT
TAACCTACCCGAGTCGGACTTTTTGGAGCTCCGCCACTGTACGTGGCTTTGTTGGGGGACGAGAGACAGAGACACTTCCC
GCCCCCGTCTGAATTTTTGCTTTCGGTTTTACGCCGAAGCCGCGCCGCGCGTCTGATTTGTTTGTTGTTCTTTTGTTCTT
CGTTAGTTTTCTTCTGTCTTTAAGTGTTTTCGAGATCATGGGACAGACCGTAACTACCCCTCTGAGTTTAACCTTGCAGC
ACTGGGGAGATGTCCAGCGCATTGCATCCAACCAGTCTGTGGATGTCAAGAAGAGGCGCTGGATTACCTTCTGTTCCGCT
GAATGGCCAACTTTCAATGTGGGATGGCCTCAGGATGGTACTTTCAATTTAAGTATTATCTCTCAGGTTAAGTCTAGAGT
GTTTTGTCCTGGTCCCCACGGACACCCGGATCAGGTCCCATATATCGTCACCTGGGAGGCACTTGCCTATGACCCCCCTC
CGTGGGTCAAACCGTTTGTGTCTCCTAAACTTCCTCCCTTGCCGACAGCTCCCGTCCTCCCGCCCGGTCCTTCTGCGCAA
CCTCCGTCCCGATCTGCCCTTTACCCTGCCCTTACCCCCTCTATAAAGTCCAAACCTCCTAAGCCCCAGGTTCTCCCTGA
TAGCGGCGGACCCCTCATTGACCTTCTCACAGAGGACCCCCGCCGTACAGAGCACAACCCTCCTCCTCTGCCAGGGAGA
ACGACGAAGAAGAGGCGGCCACCACCTCCGAGGTTTCCCCCCCTTCTCCCATGGTGTCTCGACTGCGGGAAAGAGAGAC
CCTCCCGCAGTGGACTCCACCACCTCCCAGGCATTTCCACTCCGCATGGGGGGAGATGGCCAGCTTCAGTATTGGCCGTT
TTCCTCTTCGGATTTATACAATTGGAAAAATAATAACCCTTCCTTTTCTGAAGATCCAGGTAAATTGACGGCCTTGATTG
AGTCCGTCCTCATCACCCACCAGCCCACCTGGGACGACTGTCAGCAGTTGTTGGGGACCCTGCTGACCGGAGAAGAAAAG
CAGCGGGTGCTCCTAGAGGCTAGAAAGGCAGTCCGGGGCAATGATGGACGCCCCACTCAGTTGCCTAATGAAGTCAATGC
TGCTTTTCCCCTTGAACGCCCCGGTTGGGATTACACCACTACAGAAGGTAGGAACCACCTAGTCCTCTATCGCCAGTTGC
TCTTAGCGGGTCTCCAGAACGCGGGCAGAAGTCCCACCAATTTGGCCAAGGTAAAAGGGATAACCCAGGGACCTAATGAG
TCTCCCTCAGCCTTTTTAGAGAGACTCAAAGAGGCCTATCGCAGGTACACTCCTTATGACCCTGAGGACCCAGGGCAAGA
AACCAATGTGTCTATGTCATTCATCTGGCAGTCTGCCCCGGATATCGGGCGAAAGTTAGAGCGGTTAGAAGATTTAAAGA
GCAAGACCTTAGGAGACTTAGTGAGGGAAGCTGAAAAGATCTTTAATAAGCGAGAAACCCCGGAAGAAAGAGAGGAACGT
ATCAAGAGAGAAACAGAGGAAAAAGAAGAACGCCGTAGGGCAGAGGATGAGCAGAGAGAGAAAGAAAGGGACCGCAGAAG
ACATAGAGAGATGAGCAAGCTCTTGGCCACTGTAGTTATTGGTCAGAGACAGGATAGACAGGGGGGAGAGCGGAGGAGGC
CCCAACTTGATAAGGACCAATGCGCCTACTGCAAAGAAAAGGGACACTGGGCTAAGGACTGCCCAAAGAAGCCACGAGGG
CCCCGAGGACTGAGGCCCCAGACCTCCCTCCTAACCTTAGGTGACTAGGGAGGTCAGGGTCAGGAGCCCCCCCCTGAACC
CAGGATAACCCTCAAAGTCGGGGGGCAACCCGTCACCTTCCTGGTAGATACTGGGGCCCAACACTCCGTGCTGACCCAAA
ATCCTGGACCCCTAAGTGACAAGGCTGCCTGGGTCCAAGGGGCTACTGGAGGAAAGCGGTATCGCTGGACCACGGATCGC
AAAGTACATCTAGCTACCGGTAAGGTCACCCACTCTTTCCTCCATGTACCAGACTGCCCCTATCCTCTGCTAGGAAGAGA
CTTGCTGACTAAACTAAAAGCCCAAATCCACTTCGAGGGATCAGGAGCTCAGGTTATGGGACCAATGGGACTGCCCCTGC
AAGTGCTGACCCTAAACATAGAAGATGAGTATCGGCTACATGAGACCTCAAAAGAGCCGGATGTTTCTCTAGGGTCCACC
TGGCTTTCTGATTTTCCCCAGGTCTGGGCGGAAACCGGGGGCATGGGACTGGCAGTTCGCCAAGCTCCTCTGATCATACC
TCTGAAGGCAACCTCTACCCCCGTGTCCATAAAACAATACCCCATGTCACAAGAAGCCAGACTGGGGATCAAGCCCCACA
TACAGAGACTGTTGGACCAGGGAATACTGGTACCCTGCCAGTCCCCCTGGAACACGCCCCTGCTACCCGTTAAGAAACCA
GGGACTAATGATTATAGGCCTGTCCAGGATCTGAGAGAAGTCAACAAGCGGGTGGAAGACATCCACCCCACCGTGCCCAA
CCCTTACAACCTCTTGAGCGGGCTCCCACCGTCCCACCAGTGGTACACTGTGCTTGATTTAAAGGATGCCTTTTTCTGCC
TGAGACTCCACCCCACCAGCCAGCCTCTCTTCGCCTTTGAGTGGAGAGATCCAGAGATGGGAATCTCAGGACAATTGACC
TGGACCAGACTCCCACAGGGTTTCAAAAACAGTCCCACCCTGTTTGATGAGGCACTGCACAGAGACCTAGCAGACTTCCG
GATCCAGCACCCAGACTTGATCCTGCTACAGTACGTGGATGACTTACTGCTGGCCGCCACTTCTGAGCTCGACTGCCAAC
AAGGTACTCGGGCCCTGTTACAAACCCTAGGGAACCTCGGGTATCGGGCCTCGGCCAAGAAAGCCCAAATTTGCCAGAAA
CAGGTCAAGTATCTGGGGTATCTTCTAAAAGAGGGTCAGAGATGGCTGACTGAGGCCAGAAAAGAGACTGTGATGGGGCA
GCCTACTCCGAAGACCCCTCGACAACTAAGGGAGTTCCTAGGGACGGCAGGCTTCTGTCGCCTCTGGATCCCTGGGTTTG
CAGAAATGGCAGCCCCCTTGTACCCTCTCACCAAAACGGGGACTCTGTTTAATTGGGGCCCAGACCAGCAAAAGGCCTAT

CAAGAAATCAAACAGGCTCTTCTAACTGCCCCAGCCCTGGGATTGCCAGATTTGACTAAGCCCTTTGAACTCTTTGTTGA
CGAGAAGCAGGGCTACGCCAAAGGCGTCCTAACGCAAAAACTGGGACCTTGGCGTCGGCCGGTGGCCTACCTGTCCAAAA
AGCTAGACCCAGTGGCAGCTGGGTGGCCCCCTTGCCTACGGATGGTAGCAGCCATTGCCGTTCTGACAAAAGATGCAGGC
AAGCTAACCATGGGACAGCCGCTAGTCATCCTGGCCCCCCATGCAGTAGAGGCACTAGTCAAGCAACCCCCTGACCGCTG
GCTATCCAACGCCCGCATGACCCACTATCAGGCGATGCTCCTAGATACGGACCGGGTCCAGTTCGGACCGGTGGTAGCCC
TAAACCCGGCTACGTTGCTCCCCCTACCGGGGAAAGAGCCTCACCATGACTGCCTCGAGATCTTGGCCGAGACACACGGA
ACCAGACCAGACCTCACGGACCAGCCCCTCCCAGACGCCGACCACACCTGGTATACAGATGGAAGCAGCTTCCTGCAAGA
GGGACAACGTAGGGCTGGAGCAGCGGTGACCACCGAGACCGAGGTAATCTGGGCCTGCCTTCGTCTCCCAGGTAAGTCAG
TCGGTGGCCAAGCTACTGGGGATTGATTGGAAACTACATTGTGCTTACAGACCCCAGAGTTCAGGTCAGGTAGAAAGAAT
GAATAGGACAATCAAGGAGACTTTGACCAAATTAACGCTTGCAACTGGCACTAGAGACTGGGTACTCCTACTTCCCTTAG
CCCTCTACCGAGCCCGCAACACTCCGGGCCCCCATGGACTCACTCCGTATGAAATCCTGTATGGGGCGCCCCCGCCCCTT
GTTAATTTCCATGATCCTGAAATGTCAAAGTTTACTAATAGCCCCTCTCTCCAAGCTCACTTACAGGCCCTCCAAGCAGT
ACAACGAGAGGTCTGGAAGCCACTGGCCGCTGCCTATCAGGACCAGCTGGACCAGCCAGTGATACCACACCCCTTCCGTG
TCGGCGACACCGTGTGGGTACGCCGGCACCAGACTAAAAACTTGGAACCTCGCTGGAAAGGACCCTACACCGTCCTGCTG
ACCACCCCCACCGCTCTCAAAGTAGACGGCATCGCTGCGTGGATCCACGCCGCTCACGTAAAAGCGGCGACAACCCCTCC
GGCCGGAACAGCATCAGGACCGACATGAAAGGTCCAGCGTTCTCAAAACCCCTTAAAGATAAGATTAACCCGTGGGGCCC
CCTAATAATCCTGGGGATCTTAATAAGAGCAGGAGTATCAGTACAACATGACAGCCCTCATCAGGTCTTCAATGTTACTT
AGAGAGTTACCAACTTAATGACAGGACAAACAGCTAATGCTACCTCCCTCCTGGGGACAATGACCGATGCCTTTCCTAAA
CTGTACTTTGACTTGTGCGATTTAATAGGGGACGACTGGGATGAGACTGGACTCGGGTGTCGCACTCCCGGGGGAAGAAA
AAGGGCAAGAACATTTGACTTCTATGTTTGCCCCGGGCATACTGTACCAACAGGGTGTGGAGGGCCGAGAGAGGGCTACT
GTGGCAAATGGGGCTGTGAGACCACTGGACAGGCATACTGGAAGCCATCATCATCATGGGACCTAATTTCCCTTAAGCGA
GGAAACACCCCTCGGAATCAGGGCCCCTGTTATGATTCCTCAGCGGTCTCCAGTGACATCAAGGGCGCCACACCGGGGGG
TCGGTGCAATCCCCTAGTCCTGGAATTCACTGACGCGGGCAAAAAGGCCAGCTGGGATGGCCCCAAAGTATGGGGACTAA
GACTGTACCGATCCACAGGGACCGACCCGGTGACCCGGTTCTCTTTGACCCGCCAGGTCCTCAATATAGGGCCCCGCGTC
CCCATTGGGCCTAATCCCGTGATCACTGACCAGTTACCCCCCTCCCGACCCGTGCAGATCATGCTCCCCAGGCCTCCTCA
GCCTCCTCCTCCAGGCGCAGCCTCTATAGTCCCTGAGACTGCCCCACCTTCTCAACAACCTGGGACGGGAGACAGGCTGC
TAAACCTGGTAGATGGAGCCTACCAAGCTCTCAACCTCACCAGTCCTGACAAAACCCAAGAGTGCTGGTTGTGTCTGGTA
GCGGGACCCCCCTACTACGAAGGGGTTGCCGTCCTAGGTACTTATTCCAACCATACCTCTGCCCCAGCTAACTGCTCCGT
GGCCTCCCAACACAAGCTGACCCTGTCCAAAGTGACCGGACAGGGACTCTGCGTAGGAGCAGTTCCCAAAACCCATCAGG
CCCTGTGTAATACCACCCAGAAGACGAGCGACGGGTCCTACTATCTGGCTGCTCCCGCCGGGACCATTTGGGCTTGCAAC
ACCGGGCTCACTCCCTGCCTATCTACCACTGTACTCGACCTCACCACCGATTACTGTGTCCTGGTTGAGCTCTGGCCAAA
GGTGACCTACCACTCCCCTGGTTATGTTTATGGCCAGTTTGAGAGAAAAACCAAATATAAAAGAGAGCCGGTGTCATTAA
CTCTGGCCCTGCTGTTGGGAGGACTTACTATGGGCGGCATAGCTGCAGGAGTAGGAACCGGGACTACAGCCCTAGTGGCC
ACCAAACAATTCGAGCAGCTCCAGGCAGCCATACATACAGACCTTGGGGCTTTAGAAAAGTCAGTCAGTGCCCTAGAAAA
GTCTCTGACCTCGTTGTCTGAGGTGGTCCTACAGAACCGGAGGGGATTAGATCTACTGTTCCTAAAAGAAGGAGGATTAT
GTGCTGCCCTAAAAGAAGAATGCTGTTTCTACGCGGACCACACTGGCGTAGTAAGAGATAGCATGGCAAAGCTAAGAGAA
AGGTTGAACCAGAGACAAAAATTGTTCGAATCAGGACAAGGGTGGTTTGAGGGACTGTTTAACAGGTCCCCATGGTTCAC
GACCTTAATATCCACCATTATGGGCCCCTTGATAATACTTTTATTAAACCTACTCTTCGGACCCTGTATTCTCAACCGCT
TGGTCCAGTTTGTAAAAGACAGAATTTCGGTAGTGCAGGCCCTGGTTTTGACCCAACAGTATCACCAACTCAAATCAATA
GATCCAGAAGAAGTGGAATCACGTGAATAAAAGATTTTATTCAGTTTCCAGAAAGAGGGGGGAATGAAAGACCCCACCAT
CAGGCTTAGCAAGCTAGCTGCAGTAACGCCATTTTGCAAGGCATGAAAAAGTACCAGAGCTGAGTTCTCAAAAGTTACAA
GAAAGTTCAGTTAAAGATTAAGGCTGAATAATACTGGGACAGGGGCCAAATATCGGTGGTCAAGCACCTGGGCCCCGGCT
CAGGGCCAAGAACAGATGGCTCTCAGACGTCAGTGTTAGCAGAACTAGCTTCACTGATTTAGAAAAATAGAGGTGCACAG
TGCTCTGGCCACTCCTTGAACCTGTGTGTCTGCCAATGTTCTGACCAGGTGTGTGCCCATTGTTGAACCTTCATTAGACC
CTTTCCTCGTACCCCTCCCACACCCATTTCTTGAAAATAGACATTGTTTAGAACTAAAAAGTCCCACCTCAGTTTCCCCA
AATGACCGAGAAATACCCCAAGCCTTATTCGAACTAACCAACCAGCTCGCTTCTCGCTTCTGTAACCGCGCTTTTTGCTC
CCCAGCCCCAGCCCTATAAAAAGGGTAAAAACTCCACACTCGGCGCGCCAGTCCTCCGATAGACTGCGTCGCCCGGGTAC
CCGTGTTCTCAATAAAGCCTCTTGCTGTTTGCATCCGAATCGTGGTCTCGCTGGTCCTTGAGAGGGTCTCCTCAGATTGA
TTGACTACCCACGTCGGGGGTCTTTCA
>Pmv9
TGAAAGACCCCACCATCAGGCTTAGCAAGCTAGCTGCAGTAACGCCATTTTGCAAGGCATGAAAAAGTACCAGAGCTGAG
TTCTCAAAAGTTACAAGAAAGTTCAGTTAAAGATTAACAGTTAAAGATTAAGGCTGAATAATACTGGGACAGGGGCCAAA
TATCGGTGGTCAAGCACCTGGGCCCCGGCTCAGGGCCAAGAACAGATGGCTCTCAGACGTCAGTGTTAGCAGAACTAGCT
TCACTGATTTAGAAAAATAGAGGTGCACAGTGCTCTGGCCACTCCTTGAACCTGTGTGTCTGCCAATGTTCTGACCAGGT
GTGTGCCCATTGTTGAACCTTCATTAGACCCTTTCCTCGTACCCCTCCCATACCCATTTCTTGAAAATAGACATTGTTTA
GAACTAAAAAGTCCCACCTCAGTTTCCCCAAATGACCGAGAAATACCCCAAGCCTTATTCAAACTAACCAACCAGCTCGC
TTCTCGCTTCTGTAACCGCGCTTTTTGCTCCCCAGCCCCAGCCCTATAAAAAGGGTAAAAACTCCACACTCGGCGCGCCA

GTCCTCCGATAGACTGCGTCGCCCGGGTACCCGTGTTCCCAATAAAGCCTCTTGCTGTTTGCATCCGAATCGTGGTCTCG
CTGGTCCTTGAGAGGGTCTCCTCAGATTGATTGACTACCCACGTCGGGGGTCTTTCATTTGGAGGTCCCACCGAGATTTG
GAGACCCCTGCCCAGGGACCACCGACCCCCCGCCGGGAGGTAAGCTGGCCAGCGGTCGTTTCGTGTCTGTCTCTGTCTCC
GTGCGTGTTTGTGCCGGCCTCTAATGTTTGCGCCTGCGTCTGTACTAGTTGGCTAACTAGATCTGTATCTGGCGGTTCCG
CGGAAGAACTGACGAGTTCGTATTCCCGGCCGCAGCCCCTGGGAGACGTCCCAGCGGCCTCGGGGGCCCGTTTTGTGGCC
CATTCTGTATCAGTTAACCTACCCGAGTCGGACTTTTTGGAGCTCCGCCACTGTACGTGGCTTTGTTGGGGGACGAGAGA
CAGAGACACTTCCCGCCCCCGTCTAAATTTTTGCTTTCGGTTTTACGCCGAAGCCGCGCCGCGCGTCTGATTTGTTTGTT
GTTCTTTTGTTCTTCGTTAGTTTTCTTCTGTCTTTAAGTGTTTTCGAGATCATGGGACAGACCGTAACTACCCCTCTGAG
TTTAACCTTGCAGCACTGGGGAGATGTCCAGCGCATTGCATCCAACCAGTCTGTGGATGTCAGGAAGAGGCGCTGGATTA
CCTTCTGTTCCGCTGAATGGCCAACTTTCAATGTGGGATGGCCTCAGGATGGTACTTTCAATTTAAGTATTATCTCTCAG
GTTAAGTCTAGAGTGTTTTGTCCTGGTCCCCACGGACACCCGGATCAGGTCCCATATATCGTCACCTGGGAGGCACTTGC
CTATGACCCCCCTCCGTGGGTCAAACCGTTTGTGTCTCCTAAACTTCCTCCCTTGCCGACAGCTCCCGTCCTCCCGCCCG
GTCCTTCTGCGCAACCTCCGTCCCGATCTGCCCTTTACCCTGCCCTTACCCCCTCTATAAAGTCCAAACCTCCTAAGCCC
CAGGTTCTCCCTGATAGCGGCGGACCCCTCATTGACCTTCTCACAGAGGACCCCCCGCCGTACAGAGCACAACCCTCCTC
CTCTGCCAGGGAGAACGACGAAGAAGAGGCGGCCACCACCTCCGAGGTTTCCCCCCCTTCTCCCATGGTGTCTCGACTGC
GGGGAAGGAGAGACCCTCCCGCAGTGGACTCCACCACCTCCCAGGCATTTCCACTCCGCATGGGGGGAGATGGCCAGCTT
CAGTATTGGCCGTTTTCCTCTTCGGATTTATACAATTGGAAAAATAATAACCCTTCCTTTTCTGAAGATCCAGGTAAATT
GACGGCCTTGATTGAGTCCGTCCTCATCACCCACCAGCCCACCTGGGACGACTGTCAGCAGTTGTTGGGGACCCTGCTGA
CCGGAGAAGAAAAGCAGCGGGTGCTCCTAGAGGCTAGAAAGGCAGTCCGGGGCAATGATGGACGCCCCACTCAGTTGCCT
AATGAAGTCAATGCTGCTTTTCCCCTTAAACGCCCCGGTTGGGATTACACCACTACAGAAGGTAGGAACCACCTAGTCCT
CTATCGCCAGTTGCTCTTAGCGGGTCTCCAGAACGCGGGCAAAAGTCCCACCAATTTGGCCAAGGTAAAAGGGATAACCC
AGGGACCTAATGAGTCTCCCTCAGCCTTTTTAGAGAGACTCAAAGAGGCCTATCGCAGGTACACTCCTTATGACCCTGAG
GACCCAGGGCAAGAAACCAATGTGTCTATGTCATTCATCTGGCAGTCTGCCCCGGATATCGGGCGAAAGTTAGAGCGGTT
AGAAGATTTAAAGAGCAAGACCTTAGGAGACTTAGTGAGGGAAGCTGAAAAGATCTTTAATAAGCGAGAAACCCCGGAAG
AAAGAGAGGAACGTATCAAGAGAGAAACAGAGGAAAAAGAAGAACGCCGTAGGGCAGAGGATGAGCAGAGAGAGAAAGAA
AGGGACCGCAGAAGACATAGAGAGATGAGCAAGCTCTTGGCCACTGTAGTTATTGGTCAGAGACAGGATAGACAGGGGGG
AGAGCGGAGGAGGCCCCAACTTGATAAGGACCAATGCGCCTACTGCAAAGAAAAGGGACACTGGGCTAAGGACTGCCCAA
AGAAGCCACGAGGGCCCCGAGGACTGAGGCCCCAGACCTCCCTCCTAACCTTAGGTGACTAGGGAGGTCAGGGTCAGGAG
CCCCCCCCTGAACCCAGGATAACCCTCAAAGTCGGGGGGCAACCCGTCACCTTCCTGGTAGATACTGGGGCCCAACACTC
CGTGCTGACCCAAAATCCTGGACCCCTAAGTGACAAGTCTGCCTGGGTCCAAGGGGCTACTGGAGGAAAGCGGTATCGCT
GGACCACGGATCGCAAAGTGCATCTAGCTACCGGTAAGGTCACCCACTCTTTCCTCCATGTACCAGACTGCCCCTATCCT
CTGCTAGGAAGAGATTTGCTGACTAAACTAAAAGCCCAAATCCACTTTGAGGGATCAGGAGCTCAGGTTGTGGGACCAAT
GGGACAGCCCCTGCAAGTGCTGACCCTAAACATAGAAGATGAGTATCGGCTACATGAGACCTCAAAAGAGCCGGATGTTT
CTCTAGGGTCCACATGGCTTTCTGATTTTCCCCAGGCCTGGGCGGAAACCGGGGGCATGGGACTGGCAGTTCGCCAAGCT
CCTCTGATCATACCTCTGAAGGCAACCTCTACCCCCGTGTCCATAAAACAATACCCCATGTCACAAGAAGCCAGACTGGG
GATCAAGCCCCACATACAGAGACTGTTGGACCAGGGAATACTGGTACCCTGCCAGTCCCCCTGGAACACGCCCCTGCTAC
CCGTTAAGAAACCAGGGACTAATGATTATAGGCCTGTCCAGGATCTGAGAGAAGTCAACAAGCGGGTGGAAGACATCCAC
CCCACCGTGCCCAACCCTTACAACCTCTTGAGCGGGCTCCCACCGTCCCACCAGTGGTACACTGTGCTTGACTTAAAGGA
TGCCTTTTTCTGCCTGAGACTCCACCCCACCAGTCAGCCTCTCTTCGCCTTTGAGTGGAGAGACCCAGAGATGGGAATCT
CAGGACAATTAACCTGGACCAGACTCCCACAGGGTTTCAAAAACAGTCCCACCCTGTTTGATGAGGCACTGCACAGAGAC
CTAGCAGGCTTCCGGATCCAGCACCCAGACTTGATCCTGCTACAGTACGTGGATGACTTACTGCTGGCCGCCACTTCTGA
GCTCGACTGCCAACAAGGTACTCGGGCCCTGTTACAAACCCTAGGGGACCTCGGGTATCGGGCCTCGGCCAAGAAGGCCC
AAATTTGCCAGAAACAGGTCAAGTATCTGGGGTATCTTCTAAAAGAGGGTCAGAGATGGCTGACTGAGGCCAGAAAAGAG
ACTGTGATGGGGCAGCCTACTCCGAAGACCCCTCGACAACTAAGGGAGTTCCTAGGGACGGCAGGCTTCTGTCGCCTCTG
GATCCCTGGGTTTGCAGAAATGGCAGCCCCCTTGTACCCTCTCACCAAAACGGGGACTCTGTTTAATTGGGGCCCAGACC
AGCAAAAGGCCTATCAAGAAATCAAACAGGCTCTTCTAACTGCCCCAGCCCTGGGATTGCCAGATTTGACTAAGCCCTTT
GAACTCTTTGTCGACGAGAAGCAGGGCTACGCCAAAGGCGTCCTAACGCAAAAACTGGGACCTTGGCGTCGGCCGGTGGC
CTACCTGTCCAAAAAGCTAGACCCAGTGGCAGCTGGGTGGCCCCCTTGCCTACGGATGGTAGCAGCCATTGCCGTTCTGA
CAAAAGATGCAGGCAAGCTAACCATGGGACAGCCGCTAGTCATCCTGGCCCCCCATGCAGTAGAGGCACTAGTCAAGCAA
CCCCCGGATCGCTGGCTATCCAACGCCCGCATGACCCACTATCAGGCGATGCTCCTAGATACGGACCGGGTCCAGTTCGG
ACCGGTGGTAGCCCTAAACCCGGCTACGTTGCTCCCCCTACCGGGGAAAGAGCCTCACCATGACTGCCTCGAGATCTTGG
CCGAGACACACGGAACCAGACCAGACCTCACGGACCAGCCCCTCCCAGACGCCGACCACACCTGGTATACAGATGGAAGC
AGCTTCCTGCAAGAGGGACAACGTAGGGCTGGAGCAGCGGTGACCACCGAGACCGAGGTAATCTGGGCCAAGGCATTGCC
AGCCGGGACATCCGCCCAGCGAGCTGAACTAATAGCACTCACCCAGGCCCTAAAGATGGCAGAAGGTAAGAAGCTAAATG
TTTATACTGATAGCCGCTATGCCTTTGCTACCGCCCATGTCCATGAAAAAATATATAGGAGACGTGGGTTGCTCACCTCA
GAAGGCAAGGAGATCAAGAACAAGGGCGAAATCTTGGCCTTACTGAAAGCTCTCTTTCTGCCCAAAAGACTCAGTATAAT
TCACTGCCCAGGACATCAGAAAGGCAATAGTGCTGAAGCTAAAGGCAACCGAATGGCGGACCAGGCAGCCCGGGAAGCAG

CCATGGGGACTGACACAAAGGCCTCCTCACTTCTCATAGAGACCTCAACCCCGTACACTCCAGACTTCTTCCATTATACT
GAGACAGATATAAAGAACCTACAAGAGTTGGGAGCCACATATGATAGAGAGAAAAAATATTGGGTCCTGCAAGGTAAACC
TGTGATGCCTGACCAGTTCACCTTTGAATTATTAGACTTCCTTCACCAGCTCACCCACCTTAGCTATCAGAAGATGAGGG
CACTTCTAGACAGGAAAGAAAGCCCCTATTACATGCTAAATAAAGATAAGATCCTCCACGAGGTGGCGGAATCATGCCAA
GCCTGTGTCCAAGTAAATGCCAGTAAGACTAAGATCAGGGCCGGAACACGAGTAAGAGGACATCGACCAGGCACCCATTG
GGAAATTGACTTTACTGAAGTGAAGCCCGGACTGTATGGGTACAAGTATCTCCTGGTATTCGTGGACACGTTCTCTGGCT
GGGTTGAAGCCTTCCCAACCAAACATGAGACTGCCAAAATAGTGACCAAGAAACTTCTGGAAAAAATATTTCCAAGGTTT
GGAATGCCCCAAGTGTTGGGGACTGATAATGGGCCTGCCTTCGTCTCCCAGGTAAGTCAGTCGGTGGCCAAGCTACTGGG
GATTGATTGGAAACTACATTGTGCTTACAGACCCCAGAGTTCAGGTCAGGTAGAAAGAATGAATAGGACAATCAAGGAGA
CTTTGACCAAATTAACGCTTGCAACTGGCACTAGAGACTGGGTACTCCTACTTCCCTTAGCCCTCTACCGAGCCCGCAAC
ACTCCGGGCCCCCATGGACTCACTCCGTATGAAATCCTGTATGGGGCGCCCCCGCCCCTTGTTAATTTCCATGATCCTGA
AATGTCAAAGTTTACTAATAGCCCCTCTCTCCAAGCTCACTTACAGGCCCTCCAAGCAGTACAACGAGAGGTCTGGAAGC
CACTGGCCGCTGCCTATCAGGACCAGCTGGACCAGCCAGTGATACCACACCCCTTCCGTGTCGGCGACACCGTGTGGGTA
CGCCGGCACCAGACTAAGAACTTGGAACCTCGCTGGAAAGGACCCTACACCGTCCTGCTGACCACCCCCACCGCTCTCAA
AGTAGACGGCATCGCTGCGTGGATCCACGCCGCTCACGTAAAAGCGGCGACAACCCCTCCGGCCGGAACAGCATCAGGAC
CGACATGAAAGGTCCAGCGTTCTCAAAACCCCTTAAAGATAAGATTAACCCGTGGGGCCCCCTAATAGTCCTGGGGATCT
TAATAAGGGCAGGAGTATCAGTACAACATGACAGCCCTCATCAGGTCTTCAATGTTACTTGGAGAGTTACCAACTTAATG
ACAGGACAAACAGCTAATGCTACCTCCCTCCTGGGGACAATGACCGATGCCTTTCCTAAACTGTACTTTGACTTGTGCGA
TTTAATAGGGGACGACTGGGATGAGACTGGACTCGGGTGTCGCACTCCCGGGGGAAGAAAAAGGGCAAGAACATTTGACT
TCTATGTTTGCCCCGGGCATACTGTACCAACAGGGTGTGGAGGGCCGAGAGAGGGCTACTGTGGCAAATGGGGCTGTGAG
ACCACTGGACAGGCATACTGGAAGCCATCATCATCATGGGACCTAATTTCCCTTAAGCGAGGAAACACCCCTCGGAATCA
GGGCCCCTGTTATGATTCCTCAGCGGTCTCCAGTGACATCAAGGGCGCCACACCGGGGGGTCGATGCAATCCCCTAGTCC
TGGAATTCACTGACGCGGGCAAAAAGGCCAGCTGGGATGGCCCCAAAGTATGGGGACTAAGACTGTACCGATCCACAGGG
ACCGACCCGGTGACCCGGTTCTCTTTGACCCGCCAGGTCCTCAATATAGGGCCCCGCGTCCCCATTGGGCCTAATCCCGT
GATCACTGACCAGTTACCCCCCTCCCGACCCGTGCAGATCATGCTCCCCAGGCCTCCTCAGCCTCCTCCTCCAGGCGCAG
CCTCTATAGTCCCTGAGACTGCCCCACCTTCTCAACAACCTGGGACGGGAGACAGGCTGCTAAACCTGGTAGATGGAGCC
TACCAAGCTCTCAACCTCACCAGTCCTGACAAAACCCAAGAGTGCTGGTTGTGTCTGGTAGCGGGACCCCCCTACTACGA
AGGGGTTGCCGTCCTAGGTACTTATTCCAACCATACCTCTGCCCCAGCTAACTGCTCCGTGGCCTCCCAACACAAGCTGA
CCCTGTCCGAAGTGACCGGACAGGGACTCTGCGTAGGAGCAGTTCCCAAAACCCATCAGGCCCTGTGTAATACCACCCAG
AAGACGAGCGACGGGTCCTACTATCTGGCTGCTCCCGCCGGGACCATTTGGGCTTGCAACACCGGGCTCACTCCCTGCCT
ATCTACCACTGTACTCGACCTCACCACCGATTACTGTGTCCTGGTTGAGCTCTGGCCAAAGGTGACCTACCACTCCCCTG
GTTATGTTTATGGCCAGTTTGAGAGAAAAACCAAATATAAAAGAGAGCCGGTGTCATTAACTCTGGCCCTGCTGTTGGGA
GGACTTACTATGGGCGGCATAGCTGCAGGAGTAGAAACCGGGACTACAGCCCTAGTGGCCACCAAACAATTCGAGCAGCT
CCAGGCAGCCATACATACAGACCTTGGGGCTTTAGAAAAGTCAGTCAGTGCCCTAGAAAAGTCTCTGACCTCGTTGTCTG
AGGTGGTCCTACAGAACCGGAGGGGATTAGATCTACTGTTCCTAAAAGAAGGAGGATTATGTGCTGCCCTAAAAGAAAAA
TGCTGTTTCTACGCGGACCACACTGGCGTAGTAAGAGATAGCATGGCAAAGCTAAGAGAAAGGTTAAACCAGAGACAAAA
ATTGTTCGAATCAGGACAAGGGTGGTTTGAGGGACTGTTTAACAGGTCCCCATGGTTCACGACCTTAATATCCACCATTA
TGGGCCCCTTGATAATACTTTTATTAATCCTACTCTTCGGACCCTGTATTCTCAACCGCTTGGTCCAGTTTGTAAAAGAC
AGAATTTCGGTAGTGCAGGCCCTGGTTTTGACCCAACAGTATCACCAACTCAAATCAATAGATCCAGAAGAAGTGGAATC
ACGTGAATAAAAGATTTTATTCAGTTTCCAGAAAGAGGGGGGAATGAAAGACCCCACCATCAGGCTTAGCAAGCTAGCTG
CAGTAACGCCATTTTGCAAGGCATGAAAAAGTACCAGAGCTGAGTTCTCAAAAGTTACAAGAAAGTTCAGTTAAAGATTA
ACAGTTAAAGATTAAGGCTGAATAATACTGGGACAGGGGCCAAATATCGGTGGTCAAGCACCTGGGCCCCGGCTCAGGGC
CAAGAACAGATGGCTCTCAGACGTCAGTGTTAGCAGAACTAGCTTCACTGATTTAGAAAAATAGAGGTGCACAGTGCTCT
GGCCACTCCTTGAACCTGTGTGTCTGCCAATGTTCTGACCAGGTGTGTGCCCATTGTTGAACCTTCATTAGACCCTTTCC
TCGTACCCCTCCCATACCCATTTCTTGAAAATAGACATTGTTTAGAACTAAAAAGTCCCACCTCAGTTTCCCCAAATGAC
CGAGAAATACCCCAAGCCTTATTCAAACTAACCAACCAGCTCGCTTCTCGCTTCTGTAACCGCGCTTTTTGCTCCCCAGC
CCCAGCCCTATAAAAAGGGTAAAAACTCCACACTCGGCGCGCCAGTCCTCCGATAGACTGCGTCGCCCGGGTACCCGTGT
TCCCAATAAAGCCTCTTGCTGTTTGCATCCGAATCGTGGTCTCGCTGGTCCTTGAGAGGGTCTCCTCAGATTGATTGACT
ACCCACGTCGGGGGTCTTTCA
>Xmv10
TGAAAGACCCCACCATAAGGCTTAGCAAGCTAGCTGCAGTAACGCCATTTTGCAAGGCATGAAAAAGTACCAGAGCTGAG
TTCTCAAAAGTTACAAGGAAGTTCAGTTAAAGATTAACAGTTACAAATCAAGGCTGAATAATACTAGGACAAGGGCCAAG
AACCGATGGTACCCAGATAAAGCGGAACCAGCAACAGTTTCTGAAAAAGTCCCACCTCAGTTTCAGGTTCCCCAAATGAC
CAGGAAATACCCCAAGCCTTGATTTGAACTAACCACTCAGCTCGCTTCTCGCTTCTGTACCCGCGCTTTTTGCTCCCCAG
CCCCAGCCCTATAAAAAGGGTAAGAACTCCACACTCGGCGCGCCAGTCCTCCGACAGACTGAGTCGCCCGGGTACCCGTG
TTCCCAATAAAGCCTCTTGCTGATTACATCCGAATCGTGGTCTCGCTGATCCTTGGGAGGGTCTCCTCAGATTGATTGAC
CACCCACCTCGGGGGTCTTTCATTTGGAGGTTCCACCGAGATTAGGAGACCCCTGCCCAGGGACCACCGACCCCCGCCGG

GAGGTAAGCTGGCCAGCGGTCGTTTCGTGTCTGTCTCTGTCTCCGTGCGTGTTCGTGCCGGCATCTAATGTTTGCGCCTG
CGTCTGTACTAGTTGGCTAACTAGATCTGAATCTGGCGGTTCCGTGGAAGAACTGACGAGTTCATATTCCCGGCCGCAGC
CCTGGGAGACGTCTCAGAGGCATCGGGGGCCATCTTTGTGGCCCAATCTGTATCTGAGAACCCGACCCGTCTCGGACTCT
TTGGAGCCTCTCCTTTGACCGAGGGATACGTGGTTCTGTTGGGCGGCGAGGGGCCGAAACGCTCCTCTCCCCCATCTGAA
TTTTTGCTTTCGGTTTTCCGCCGAAACCGCGCCGCGCGTCTTGTCTGTCTCTGTGTTGTTTTGTCATTTGTCGGTTCGTT
ATTGTTTTGGACCGTTTCTAAAAATATGGGACAGACCGTAACCACTCCTTTGAGTCTGACCCTAGAACACTGGGGAGACG
TCCAGCGCATTGCGTCCAACCAGTCCGTGGACGTCAAGAAGAGACGTTGGGTCACCTTCTGCTCTGCCGAGTGGCCAACT
TTCGATGTGGGGTGGCCGCAAGATGGTACTTTTAATTTGGACATTATTTTACAGGTTAAATCTAAGGTGTTCTCTCCCGG
TCCCCACGGACACCCGGATCAGGTCCCATACATTGTCACCTGGGAGGCACTTGCCTATGACCCCCCTCCGTGGGTCAAAC
CGTTTGTCTCTCCAAAACCCCCTCCTTTACCGACAGCTCCCGTCCTCCCGCCCGGTCCTTCTGCGCAACCTCCGTCCCGA
TCTGCCCTTTACCCTGCCCTTACCCCCTCTATAAAGACCAAACCTCCTAAGCCCCAGGTTCTCCCTGATAACGGCGGACC
GCTCATTGACCTTCTCACAGAGGACCCCCCGCCGTACGGAGCACAACCTTCCTCCTCTGCCAGAGGGAACGATGAAGAAG
AGGCGGCCGCCACCTCCGAGGTTTCCCCCCCTTCTCCCATGGTGTCTCGACTGCGGGGAAGGAGGGACCCTCCCGCAGCG
GACTCCACCTCCTCCCAGGCATTCCCACTCCGCATGGGGGGAGATGGCCAGCTTCAGTATTGGCCGTTTTCCTCCTCGGA
CTTATACAATTGGAAAAATAATAACCCTTCCTTTTCTGAAGACCCAGGTAAATTGACGGCCTTGATTGAGTCCGTCCTCA
TCACCCACCAGCCCACCTGGGACGACTGTCAGCAGTTGTTGGGGACCCTGCTGACCGGAGAAGAAAAGCAGCGGGTGCTC
CTAGAGGCTAGAAAGGCAGTCCGGGGCAATGATGGACGCCCCACTCAGTTGCCTAATGAAGTCAATGCTGCTTTTCCCCT
TGAACGCCCCGATTGGGATTACACCACTACAGAAGGTAGGAACCACCTAGTCCTCTATCGCCAGTTGCTCTTAGCGGGTC
TCCAAAACGCGGGCAGAAGCCCCACCAATTTGGCCAAGGTAAAAGGGATAACCCAGGGACCTAATGAGTCTCCCTCAGCC
TTTTTAGAGAGACTCAAGGAGGCCTATCGCAGGTACACTCCTTATGACCCTGAGGACCCAGGGCAAGAAACCAATGTGTC
TATGTCATTCATCTGGCAGTCTGCCCCGGATATCGGGCGAAAGTTAGAGCGGTTAGAAGATTTAAAGAGCAAGACCTTAG
GAGACTTAGTGAGGGAAGCTGAAAGGATCTTTAATAAGCGAGAAACCCCGGAAGAAAGAGAGGAACGTATCAGGAGAGAA
ACAGAGGAAAAAGAAGAACGCCGTAGGGCAGAGGATGAGCAGAGAGAGAAAGAAAGGGACCGCAGAAGACATAGAGAGAT
GAGCAAGCTCTTGGCCACTGTAGTTATTGGTCAGAGACAGGATAGACAGGGGGGAGAGCGGAGGAGGCCCCAACTTGATA
AGGACCAATGCGCCTACTGCAAAGAAAAGGGACACTGGGCTAAGGACTGCCCAAAGAAGCCACGAGGGCCCCGAGGACCG
AGGCCCCAGACCTCCCTCCTGACCTTAGGTGACTAGGGAGGTCAGGGTCAGGAGCCCCCCCTGAACCCAGGATAACCCT
CAAAGTCGGGGGGCAACCCGTCACCTTCCTGGTAGATACTGGGGCCCAACACTCCGTGCTGACCCAAAATCCTGGCCCCC
CATGCAGTAGAGGCACTGGTCAAGCAACCCCCTGACCGCTGGCTATCCAATGCCCGCATGACCCACTACCAGGCAATGCT
CCTAGACACTGACCGAGTTCAGTTCGGACCAGTGGTGGCCCTCAATCCTGCCACCTTGCTCCCTCTACCGGAGAAAGGAG
CCCCCCATGATTGCCTCGAGATCTTGGCTGAAACGCATGGAACCAGACCGGACCTCACCGACCAGCCCATCCCAGACGCC
GACCACACCTGGTATACCGATGGGAGCAGCTTTTTGCAAGAAGGACAGCGAAAGGCTGGGGCAGCAGTGACGACTGAGAC
CGAGGTAATCTGGGCGAGGGCCCTGCCAGCTGGAACGTCAGCCCAGCGAGCCGAACTGATCGCACTCACCCAAGCCCTGA
AAATGGCAGAAGGTAAGAAGCTAAATGTTTATACTGACAGCCGATATGCTTTCGCCACGGCCCATGTCCATGGAGAAATC
TATAGGAGGCGAGGGTTGCTGACCTCAGAGGGCAGAGAAATCAAAAACAAGAGCGAGATCCTGGCTTTACTGAAAGCTCT
TTTCCTGCCTAAAAGACTCAGTATAATTCACTGCCCCGGGCATCAAAAGGGAAACAGTGCTGAAGCCAGGGGCAACCGTA
TGGCAGACCAAGCGGCCCGAGAGGCAGCCATAAGGACATCTCCAGAAACTTCCACCCTCCTCATAGAGGACTCGACCCCG
TATACGCCCTCCCATTTCCACTACACTGAAACAGATCTAAAGAGATTACGAGAACTGGGAGCCACCTATAATCAGATAAA
AGGATATTGGGTCCTACAAGGCAAGCCGGTAATGCCCGATCAGTTTGTGTTTGAACTATTAGACTCCTTACACAGGCTCA
CTCACCTCAGCCCTCAAAAGATGAAGGCACTCCTTGACAGAGAAGAAAGCCCCTACTACATGTTAAACAGAGACAGAACT
CTCCAGTATGTGGCAGAATCATGCACAGCTTGTGCTCAAGTGAATGCTAGTAAAGCCAAGATCGGGGCAGGGGTACGAGT
ACGCGGACATCGACCAGGTACCCATTGGGAAATTGACTTTACTGAAGTTAAGCCAGGACTGTACGGGTACAAGTACCTCC
TAGTGTTCGTGGACACCTTCTCTGGCTGGGTAGAAGCCTTCCCAACTAAACGTGAAACCGCCAAGGTGGTAACCAAGAAG
CTATTAGAAGAAATATTCCCAAGATTCGGGATGCCACAGGTACTGGGTTCTGACAATGGGCCTGCCTTCGTCTCCCAGGT
AAGTCATTCGGTGGCCGATTTACTGGGGATTGATTGGAAGTTACATTGTGCTTATAGACCCCAGAGTTCAGGTCAGGTAG
AGAGAATGAATAGAACCATCAAGGAGACTTTAACCAAATTAACGCTGGCAGCTGGCACTAGAGACTGGGTACTCCTACTC
CCCCTGGCCCTCTACCGAGCCCGGAATACTCCGGGCCCCCATGGACTTACTCCGTATGAAATCCTGTATGGGGCACCCCC
GCCCCTCGTCAATTTTCATGATCCTGAAATGTCAAAGTTAACTAATAGTCCCTCTCTCCAAGCTCACTTACAGGCCCTCC
AAGCAGTACAACGAGAGGTCTGGAAGCCACTGGCCGCTGCTTATCAGGACCAGCTGGATCAGCCAGTGATACCACACCCC
TTCCGTGTCGGTGACGCCGTGTGGGTACGCCGGCACCAGACTAAGAACTTAGAACCCCGCTGGAAAGGACCCTACACCGT
CCTGCTGACCACCCCCACCGCTCTCAAAGTAGACGGCATCTCTGCGTGGATACACGCCGCTCACGTAAAGGCGGCGACAA
CTCCTCCAGCCGGAACAGCATGAAAGGTCCAGCGTTCTCAAAACCCCTTAAAGATAAGATTAACCCGTGGGGCCCCCTAA
TAGTTATGGGGATCTTGGTGAGGGCAGGAGCCTCGGTACAACGTGACAGCCCTCACCAGATCTTCAATGTTACTTGGAGA
GTTACCAACCTAATGACAGGACAAACAGCTAACGCCACCTCCCTCCTGGGGACGATGACTGACACCTTCCCTAAACTATA
TTTTGACCTGTGTGATTTAGTAGGAGACTACTGGGATGACCCAGAACCCGATATTGGGGATGGTTGCCGCACTCCCGGGG
GAAGAAGAAGGACAAGACTGTATGACTTCTATGTTTGCCCCGGTCATACTGTACCAATAGGGTGTGGAGGGCCGGGAGAG
GGCTACTGTGGCAAATGGGGATGTGAAACCACTGGACAGGCATACTGGAAGCCATCATCATCATGGGACCTAATTTCCCT
TAAGCGAGGAAACACTCCTAAGGATCAGGGCCCCTGTTATGATTCCTCGGTCTCCAGTGGCGTCCAGGGTGCCACACCGG

GGGGTCGATGCAACCCCCTGGTCTTAGAATTCACTGACGCGGGTAGAAAGGCCAGCTGGGATGCCCCCAAAGTTTGGGGA
CTAAGACTCTATCGATCCACAGGGGCCGACCCGGTGACCCGGTTCTCTTTGACCCGCCAGGTCCTCAATGTAGGACCCCG
CGTCCCCATTGGGCCTAATCCCGTGATCACTGACCAGCTACCCCCATCCCAACCCGTGCAGATCATGCTCCCCAGGCCTC
CTCATCCTCCTCCTTCAGGCACGGTCTCTATGGTACCTGGGGCTCCCCCGCCTTCTCAACAACCTGGGACGGGAGACAGG
CTGCTAAATCTGGTAGAAGGAGCCTACCAAGCACTCAACCTCACCAGTCCTGACAAAACCCAAGAGTGCTGGTTGTGTCT
GGTATCGGGACCCCCCTACTACGAAGGGGTTGCCGTCCTAGGTACCTACTCCAACCATACCTCTGCCCCAGCTAACTGCT
CCGTGGCCTCCCAACACAAGCTGACCCTGTCCGAAGTGACCGGACAGGGACTCTGCGTAGGAGCAGTTCCCAAAACCCAT
CAGGCCCTGTGTAATACCACCCAGAAGACGAGCGACGGGTCCTACTATCTGGCTGCTCCCGCCGGGACCATCTGGGCTTG
CAACACCGGGCTCACTCCCTGCCTATCTACTACTGTACTCAACCTCACCACCGATTACTGTGTCCTGGTTGAGCTCTGGC
CAAAGGTGACCTACCACTCCCCTGGTTATGTTTATGGCCAGTTTGAGAAAAAAACCAAATATAAAAGGGAGCCGGTGTCA
TTAACTCTGGCCCTGCTGTTGGGAGGACTTACTATGGGCGGCATAGCTGCAGGAGTAGGAACAGGGACTACAGCCCTAGT
GGCCACCAAACAATTCGAGCAGCTCCAGGCAGCCATACATACAGACCTTGGGGCCTTAGAAAAATCAGTCAGTGCCCTAG
AAAAGTCTCTGACCTCGTTGTCTGAGGTGGTCCTACAGAACCGGAGAGGATTAGATCTGCTGTTCCTAAAAGAGGGAGGA
TTATGTGCTGCCCTAAAAGAAGAATGCTGTTTCTACGCGGACCACACTGGCGTAGTAAGGGATAGCATGGCTAAGCTAAG
AGAGAGACTAAACCAGAGACAAAAATTGTTCGAATCAGGACAAGGGTGGTTTGAGGGACTGTTTAACAGGTCCCCATGGT
TCACGACCCTGATATCCACCATTATGGGCCCTCTGATAGTACTTTTATTAATCCTACTCTTCGGACCCTGTATTCTCAAC
CGCTTGGTCCAGTTTGTAAAAGACAGAATTTCGGTGGTGCAGGCCCTGGTTCTGACTCAACAGTATCACCAACTCAAATC
AATAGATCCAGAAGCAGTGGAATCACGTGAATAAAAGATTTTATTCAGTTTCCAGAAAGAGGGGGGAATGAAAGACCCCA
CCATAAGGCTTAGCAAGCTAGCTGCAGTAACGCCATTTTGCAAGGCATGAAAAAGTACCAGAGCTGAGTTCTCAAAAGTT
ACAAGGAAGTTCAGTTAAAGATTAACAGTTACAAATCAAGGCTGAATAATACTAGGACAAGGGCCAAGAACCGATGGTAC
CCAGATAAAGCGGAACCAGCAACAGTTTCTGAAAAAGTCCCACCTCAGTTTCAGGTTCCCCAAATGACCAGGAAATACCC
CAAGCCTTGATTTGAACTAACCACTCAGCTCGCTTCTCGCTTCTGTACCCGCGCTTTTTGCTCCCCAGCCCCAGCCCTAT
AAAAAGGGTAAGAACTCCACACTCGGCGCGCCAGTCCTCCGACAGACTGAGTCGCCCGGGTACCCGTGTTCCCAATAAAG
CCTCTTGCTGATTACATCCGAATCGTGGTCTCGCTGATCCTTGGGAGGGTCTCCTCAGATTGATTGACCACCCACCTCGG
GGGTCTTTCA
>Xmv12
TGAAAGACCCCACCATAAGGCTTAGCAAGCTAGCTGCAGTAACGCCATTTTGCAAGGCATGAAAAAGTACCAGAGCTGAG
TTCTCAAAAGTTACAAGAAAGTTCAGTTAAAGATTAACAGTTAAAATCAAGGCTGAATAATACTAGGACAAGGGCCAAGA
ACCGATGGTACCCACCGGGGCCCCGGCTCAAGGCCAAGAACCGATGGTACCCACCTGGGCCCCGGCTCAGGGCCAAGAAC
AGATGATACCCAGATAAGGCGGAACCAGCAACAGTTTCTGAAAAAGTCCCACCTCAGTTTCAGGTTCCCCAAATGACCAG
GAAATACCCCAAGCCTTGATTTGAACTAACCACTCAGCTCGCTTCTCGCTTCTGTACCCGCGCTTTTTGCTCCCCAGCCC
TATAAAAAGGGTAAAAACCCCACACTCGGCGCGCCAGTCCTCCGAAAGACTGAGTCGCCCGGGTACCCGTGTTCCCAATA
AAGCCTCTTGCTGATTACATCCGAATCGTGGTCTCGCTGATCCTTGGGAGGGTCTCCTCAGATTGATTGACTACCCACCT
CGGGGGTCTTTCATTTGGAGGTTCCACCGAGATTAGGAGACCCCTGCCCAGGGACCACCGACCCCCGCCGGGAGGTAAGC
TGGCCAGCGGTCGTTTCGTGTCTGTCTCTGTCTCCGTGCGTGTTTGTGCCGGCATCTAATGTTTGCGCCTGCGTCTGTAC
TAGTTGGCTAACTAGATCTGTATCTGGCGGTTCCGCGGAAGAACTGACGAGTTCGTATTCCCGGCCGCAGCCCTGGGAGA
CGTCCCAGCGGCCTCGGGGGCCCGTTTTGTGGCCCATTCTGTATCAGTTAACCTGCCCGAGTCGGACTTTTTGGAGCTCC
TCCACTGTCCGAGGGGTACGTGGCTTTGTCGGGGGACGAGAGGCAGAGACACTTCCCTCCCCCGTCTGAATTTTTGCTTT
CGGTTTTACGCCGAAACCGCGCCGCGCGTCTGATTTGTTTGTTGTTCTTTTGTTCTTCGTTAGTTTTCTCCTGTCTTTAA
GTGTTTTTGAGATCATGGGACAGACCGTAACTACCCCTCTGAGTCTAACCTTGCAGCACTGGGGAGATGTCCAGCGCATT
GCATCCAATCAGTCTGTGGATGTCAGGAAGAGGCGCTGGGTTACCTTCTGTTCCGCCGAATGGCCAACTTTCAATGTGGG
ATGGCCTCAGGATGGTACTTTTAATTTAGGTATTATCTCTCAGGTCAAGTCTAGAGTGTTTTGTCCTGGTCCCCACGGAC
ACCCGGATCAGGTCCCATATATCGTCACCTGGGAGGCACTTGCCTATGACCCCCCTCCGTGGGTCAAACCGTTTGTCTCC
CCAAAACCCCCTCCTTTACCGACAGCTCCCGTCCTCCCGCCCGGTCCTTCTGCGCAACCTCCGTCCCGATCTGCCCTTTA
CCCTGCCCTTACCCCCTCTATAAAGCCCAAACCTCCTAAGCCCCAGGTTCTCCCTGATAACGGCGGACCTCTCATTGACC
TTCTCACAGAGGACCCCCCGCCGTACGGAGCACAACCTTCCTCCTCTGCCAGAGGGAACGATAAAGAAGAGGCGGCCACC
ACCTCCGAGGTTTCCCCCCCTTCTCCCATGGTGTCTCGACTGCGGGGAAGGAGAGACCCTCCCGCAGCGGACTCCACCTC
CTCCCAGGCATTCCCACTCCGCATGGGGGGAGATGGCCAGCTTCAGTACTGGCCGTTTTCCTCCTCAGACTTATATAATT
GGAAAAATAATAACCCTTCCTTTTCTGAAGACCCAGGTAAATTGACGGCCTTGATTGAGTCCGTCCTCATCACCCACCAG
CCCACCTGGGACGACTGTCAGCAGTTGTTGGGGACCCTGCTGACCGGAGAAGAAAAGCAGCGGGTGCTCCTAGAGGCTAG
AAAGGCAGTCCGGGGCAATGATGGACGCCCCACTCAGTTGCCTAATGAAGTCAATGCTGCTTTTCCCCTTGAACGCCCCG
ATTGGGATTACACCACTACAGAAGGTAGGAACCACCTAGTCCTCTATCGCCAGTTGCTCTTAGCGGGTCTCCAAAACGCG
GGCAGGAGCCCCACCAATTTGGCCAAGGTAAAAGGGATAACCCAGGGACCTAATGAGTCTCCCTCAGCCTTTTTAGAGAG
ACTCAAGGAGGCCTATCGCAGGTACACTCCTTATGACCCTGAGGACCCAGGGCAAGAAACCAATGTGTCTATGTCATTCA
TCTGGCAGTCTGCCCCGGATATCGGGCGAAAGTTAGAGCGGTTAGAAGACTTAAAGAGCAAGACCTTAGGAGACTTAGTG
AGGGAAGCTGAAAGGATCTTTAATAAGCGAGAAACCCCGGAAGAAAGAGAGGAACGTATCAGGAGAGAAACAGAGGAAAA
AGAAGAACGCCGTAGGGCAGAGGATGAGCAGAGAGAGAAAGAAAGGGACCGCAGAAGACATAGAGAGATGAGCAAGCTCT

TGGCCACTGTAGTTATTGGTCAGAGACAGGATAGACAGGGGGGAGAGCGGAGGAGGCCCCAACTTGATAAGGACCAATGC
GCCTACTGCAAAGAAAAGGGACACTGGGCTAAGGACTGCCCAAAGAAGCCACGAGGGCCCCGAGGACCGAGGCCCCAGAC
CTCCCTCCTGACCTTAGGTGACTAGGGAGGTCAGGGTCAGGAGCCCCCCCTGAACCCAGGATAACCCTCAAAGTCGGGG
GGCAACCCGTCACCTTCCTGGTAGATACTGGGGCCCAACACTCCGTGCTGACCCAAAATCCTGGACCCCTAAGTGACAAG
TCTGCCTGGGTCCAAGGGGCTACTGGAGGAAAGCGGTATCGCTGGACCACGGATCGCAAAGTACATCTAGCTACCGGTAA
GGTCACCCACTCTTTCCTCCATGTACCAGACTGCCCCTATCCTCTGCTAGGAAGAGACTTGCTGACTAAACTAAAAGCCC
AGATCCACTTCGAGGGATCAGGAGCTCAGGTTGTGGGACCAATGGGACAGCCCCTGCAAGTGCTGACCCTAAACATAGAA
GATGAGTATCGGCTACATGAGACCTCAAAAGAGCCGGATGTTTCTCTAGGGTCCACATGGCTTTCTGATTTTCCCCAGGC
CTGGGCAGAAACCGGGGGCATGGGACTGGCAGTTCGCCAAGCGCCTCTGATTATACCTCTAAAGGCAACCTCTACCCCCG
TGTCCATAAAACAATACCCCATGTCACAGGAAGCCAGACTGGGGATCAAGCCCCACATACAGAGGCTGTTGGACCAGGGA
ATACTGGTACCCTGCCAGTCCCCCTGGAACACGCCCCTGCTACCCGTTAAGAAACCAGGGACTAATGATTACAGGCCTGT
CCAGGATCTGAGAGAAGTCAACAAGCGGGTGGAAGACATCCACCCCACCGTGCCCAACCCTTACAACCTCTTGAGCGGGC
TCCCACCGTCCCACCAGTGGTACACTGTGCTTGATTTAAAGGATGCCTTTTTCTGCCTGAGACTCCACCCCACCAGTCAG
CCTCTCTTCGCCTTTGAGTGGAGAGATCCAGGGATGGGAATCTCAGGACAATTGACCTGGACCAGACTCCCACAGGGTTT
CAAAAACAGTCCCACCCTGTTTGATGAGGCACTGCACAGAGACCTAGCAGACTTCCGGATCCAGCACCCAGACTTGATCC
TGCTACAGTACGTGGATGACATACTACTGGCCGCCACTTCTGAGCTCGACTGCCAACAAGGTACTCGGGCCCTGTTACAA
ACCCTAGGAAACCTCGGGTATCGGGCCTCGGCCAAGAAAGCCCAACTTTGCCAGAAACAGGTCAAGTATCTGGGGTATCT
TCTAAAAGAGGGTCAGAGATGGCTGACTGAGGCCAGAAAAGAGACTGTGATGGGGCAGCCTACTCCGAAGACCCCTCGAC
AACTAAGGGAGTTCCTAGGGACGGCAGGCTTCTGTCGCCTCTGGATCCCTGGGTTTGCGGAAATGGCAGCCCCCTTGTAT
CCTCTTACCAAAACGGGGACTCTGTTTAATTGGGGCCCAGACCAGCAAAAGGCCTATCAAGAAATCAAACAGGCCCTTCT
AACTGCCCCCGCCCTGGGATTGCCAGATTTGACTAAGCCCTTTGAACTCTTTGTCGACGAGAAGCAGGGCTACGCCAAAG
GCGTCCTAACGCAAAAACTGGGACCTTGGCGTCGGCCTGTGGCCTACCTGTCCAAAAAGCTAGACCCAGTGGCAGCTGGG
TGGCCCCCTTGCCTACGGATGGTAGCAGCCATTGCCGTTCTGACAAAAGATGCAGGCAAGCTAACTATGGGACAGCCGCT
AGTCATCCTGGCCCCCCATGCAGTAGAGGCACTGGTCAAGCAACCCCCTGACCGCTGGCTATCCAACGCCCGCATGACCC
ACTACCAGGCAATGCTCCTAGACACTGACCGAGTTCAGTTCGGACCAGTGGTGGCCCTCAATCCTGCCACCTTGCTCCCT
CTACCGGAAAAAGGAGCCCCCCATGATTGCCTCGAGATCTTGGCTGAAACGCATGGAACCAGACCGGATCTCACCGACCA
GCCCATCCCAGACGCCGACCACACCTGGTATACCGATGGGAGCAGCTTTTTGCAAGAAGGACAGCGAAAGGCTGGGGCAG
CAGTGACGACTGAAACCGAGGTAATCTGGGCGAGGGCCCTGCCAGCTGGAACGTCAGCCCAGCGAGCCGAACTGGTCGCA
CTCACCCAAGCCCTGAAAATGGCAGAAGGTAAGAAGCTAAATGTTTACACTGATAGCCGCTATGCCTTCGCTACGGCCCA
TGTTCATGGGGAAATATATAGGAGACGGGGGTTGCTGACCTCAGAAGGCAAGGAAATCAAGAACAAAAGCGAGATCCTAG
CCTTGCTGAAAGCCCTCTTTTTGCCAAAGAGGCTCAGTATTATCCATTGCCCGGGACACCAAAAAGGGAACAGTGCTGAA
GCCAGGGGCAACCGAATGGCGGATCAAGCAGCCAGGGAGGCAGCCATGAGAGAGATCACAGAAACTTCCACGCTCTTAAT
AGAGGATTCGTCCCCGTATACTCCCAGTTACTTCCACTACACAGAGACTGACAGAAAAGACCTTCTGAGACTGGGAGCCG
TATACGATGAGGACAAAAGGTATTGGGTCCTGCAGGGAAAACCTGTAATGCCTGACCAATTCACCTTTAAGCTCTTAGAC
TCCCTACACCGGCTCACCCACCTCAACTATCAGAAGATGAAGGCACTCCTTGACCGGGAAGAGAGCCCCTACTACATGTT
AAATCGGGATAAAACTCTCCAGCACGTAACAGAATCCTGTACAGCTTGTGCTCAAGTAAATGCTAGTAAAGCCAAAATCG
GAGCAAGGGTGCGAGCGCGGGGACACCGACCCGGCACCCATTGGGAAATCGATTTTACCGAAGTTAAGCCAGGGCTGTAT
GGACATAAGTATCTCCTGGTGTTTGTGGACACATTCTCTGGCTGGGTAGAAGCATTTCCAACCAAGCGTGAAACTGCCAG
GGTTGTGACCAAGAAGCTGCTTGAAGAAATTTTTCCAAGATTCGGGATGCCCCAGGTATTGGGAACAGATAATGGGCCTG
CCTTCATCTCCCAGGTAAGTCAGTCGGTGGCCAAGTTGCTGGGGATTGATTGGAAACTGCATTGTGCTTACAGACCCCAG
AGTTCAGGTCAGGTAGAAAGAATGAACAGGACAATCAAGGAGACTTTAACCAAATTAACGCTTGCAGCTGGCACTAGAGA
CTGGGTACTCCTACTCCCCCTGGCCCTCTACCGAGCCCGGAATACTCCGGGCCCCCATGGACTTACTCCGTATGAAATTC
TGTATGGGGCACCCCCGCCCCTTGTCAATTTTCATGATCCTGAAATGTCAAAGTTAACTAATAGTCCCTCTCTCCAAGCT
CACTTACAGGCCCTCCAAGCAGTACAACGAGAGGTCTGGAAGCCACTGGCCGCTGCTTATCAGGACCAGCTGGATCAGCC
AGTGATACCACACCCCTTCCGCGTCGGTGACGCCGTGTGGGTACGCCGGCACCAGACTAAGAACTTAGAACCCCGCTGGA
AAGGACCCTACACCGTCCTGCTGACCACCCCCACCGCTCTCAAAGTAGACGGCATCTCTGCGTGGATACACGCCGCTCAC
GTAAAGGCGGCGACAACTCCTCCGGCCGGAACAGCATGGAAGGTCCAGCGTTCTCAAAATCCCTTAAAGATAAGATTAAC
CCGTGGGGCCCCCTGATAGTTATGGGGATCTTAGTGAGGGCAGGAGCCTCGGTACAACGTGACAGCCCTCACCAGGTCTT
CAATGTTACTTGGAGAGTTACCAACCTAATGACAGGACAAACAGCTAACGCCACCTCCCTCCTGGGGACGATGACAGACA
CCTTCCCTAAACTATATTTTGACCTGTGTGATTTAGTAGGAGACTACTGGGATTGGGATGACCCAGATGGAGATGGTTGC
CGCTCTCCCGGGGGAAGAAAAAGGACAAGACTGTATGACTTCTATGTTTGCCCCGGTCATACTGTACCAATAGGGTGTGG
AGGACCGGGAGAGGGCTACTGTGGCAAATGGGGATGTGAGACCACTGGACAGGCATACTGGAAGCCATCATCATCATGGG
ACCTAATTTCCCTTAAGCGAGGAAACACTCCTAAGGATCAGGGCCCCTGTTATGATTCCTCGGTCTCCAGTGGCGTCCAG
GGTGCCACACCGGGGGGTCGATGCAACCCCCTAGTCTTAGAATTCACTGACGCGGGTAAAAAGGCCAGCTGGGATGCCCC
CAAAGTTTGGGGACTAAGACTCTACCGATCCACAGGGGCCGACCCGGTGACCCGGTTCTCTTTGACCCGCCAGGTCCTCA
ATGTAGGACCCCGCGTCCCCATTGGGCCTAATCCCGTGATCACTGACCAGCTACCCCCATCCCAACCCGTGCAGATCATG
CTCCCCAGGCCTCCTCATCCTCCTCCTTCAGGCACGGTCTCTATGGTACCTGGGGCTCCCCCGCCTTCTCAACAGCCTGG

GACGGGAGACAGGCTGCTAAATCTGGTAGACGGAGCCTACCAAGCACTCAACCTCACCAGTCCTGACAAAACCCAAGAGT
GCTGGTTGTGTCTGGTATCGGGACCCCCCTACTACGAAGGGGTTGCCGTCCTAGGTACCTACTCCAACCATACCTCTGCC
CCAGCTAACTGCTCCGTGGCCTCCCAACACAAGCTGACCCTGTCCGAAGTGACCGGACAGGGACTCTGCGTAGGAGCAGT
TCCCAAAACCCATCAGGCCCTGTGTAATACCACCCAGAAGACGAGCGACGGGTCCTACTATCTGGCTGCTCCCGCCGGGA
CCATCTGGGCTTGCAACACCGGGCTCACTCCCTGCCTATCTACTACTGTACTCAACCTCACCACCGATTACTGTGTCCTG
GTTGAGCTCTGGCCAAAGGTGACCTACCACTCCCCTGGTTATGTTTATGGCCAGTTTGAGAGAAAAACCAAATATAAAAG
AGAGCCGGTGTCATTAACTCTGGCCCTGCTGTTGGGAGGACTTACTATGGGCGGCATAGCTGCAGGAGTAGGAACAGGGA
CTACAGCCCTAGTGGCCACCAAACAATTCGAGCAGCTCCAGGCAGCCATACATACAGACCTTGGGGCCTTAGAAAAATCA
GTCAGTGCCCTAGAAAAGTCTCTGACCTCGTTGTCTGAGGTGGTCCTACAGAACCGGAGAGGATTAGATCTGCTGTTCCT
AAAAGAAGGAGGATTATGTGCTGCCCTAAAAGAAGAATGCTGTTTCTACGCGGACCACACTGGCGTAGTGAGAGATAGCA
TGGCAAAGCTAAGAGAAAGGTTAAACCAGAGACAAAAATTGTTCGAATCAGGACAAGGGTGGTTTGAGGGACTGTTTAAC
AGGTCCCCATGGTTCACGACCTTGATATCCACCATTATGGGCCCCTTGATAATACTTTTATTAATCCTACTCCTCGGACC
CTGTATTCTCAACCGCTTGGTCCAGTTTGTAAAAGACAGAATTTCGGTGGTGCAGGCCCTGGTTCTGACCCAACAGTATC
ACCAACTCAAATCAATAGATCCAGAAGAAGTGGAATCACGTGAATAAAAGATTTTATTCAGTTTCCAGAAAGAGGGGGGA
ATGAAAGACCCCACCATAAGGCTTAGCAAGCTAGCTGCAGTAACGCCATTTTGCAAGGCATGAAAAAGTACCAGAGCTGA
GTTCTCAAAAGTTACAAGGAAGTTCAGTTAAAGATTAACAGTTAAAATCAAGGCTGAATAATACTAGGACAAGGGCCAAG
AACCGATGGTACCCACCGGGGCCCCGGCTCAAGGCCAAGAACCGATGGTACCCACCTGGGCCCCGGCTCAGGGCCAAGAA
CAGATGATACCCAGATAAGGCGGAACCAGCAACAGTTTCTGAAAAAGTCCCACCTCAGTTTCAGGTTCCCCAAATGACCA
GGAAATACCCCAAGCCTTGATTTGAACTAACCACTCAGCTCGCTTCTCGCTTCTGTACCCGCGCTTTTTGCTCCCCAGCC
CTATAAAAAGGGTAAAAACCCCACACTCGGCGCGCCAGTCCTCCGAAAGACTGAGTCGCCCGGGTACCCGTGTTCCCAAT
AAAGCCTCTTGCTGATTACATCCGAATCGTGGTCTCGCTGATCCTTGGGAGGGTCTCCTCAGATTGATTGACTACCCACC
TCGGGGGTCTTTCA
>Xmv13
TGAAAGACCCCACCATAAGGCTTAGCAAGCTAGCTGCAGTAACGCCATTTTGCAAGGCATGAAAAAGTACCAGAGCTGAG
TTCTCAAAAGTTACAAGGAAGTTCAGTTAAAGATTAACAGTTAAAAATCAAGGCTGAATAATACTAGGACAAGGGCCAAA
CAGAATATCGGTGGTCAAGCGCCTGGGCCCCGGCTCAGGGCCAAGAACAGATGGTACCCAGATAAAGCGGAACCAGCAAC
AGTTTCTGAAAAAGTCCCACCTCAGTTTCAGGTTCCCCAAATGACCAGGAAATACCCCAAGCCTTGATTTGAACTAACCA
CTCAGCTCGCTTCTCGCTTCTGTACCCACGCTTTTTGCTCCCCAGCCCCAGCCCTATAAAAAGGGTAAGAACTCCACACT
CGGCGCGCCAGTCCTCCGATAGACTGAGTCGCCCGGGTACCCGTGTTCCCAATAAAGCCTCTTGCTGATTACATCCGAAT
CGTGGTCTCGCTGATCCTTGGGAGGGTCTCCTCAGATTGATTGACCACCCACCTCGGGGGTCTTTCATTTGGAGGTCCCA
CCGAGATTAGGAGACCCCTGCCCAGGGACCACCGACCCCCGCCGGGAGGTAAGCTGGCCAGCGGTCGTTTCGTGTCTGTC
TCTGTCTCCGTGCGTGTCTGTGCCGGCATCTAATGTTTGCGCCTGCGTCTGTACTAGTTGGCTAACTAGATCTGAATCTG
GCGGTTCCGTGGAAGAACTGACGAGTTCATATTCCCGGCCGCAGCCCTGGGAGACGTCTCAGAGGCATCGGGGGCCATCT
TTGTGGCCCAATCTGTATCTGAGAACCCGACCCGTCTCGGACTCTTTGGAGCCTCTCCTTTGACCGAGGGATACGTGGTT
CTGTTGGGCGGCGAGGGGCCGAAACGCTCCCCTCCCCCATCTGAATTTTTGCTTTCGGTTTTCCGCCGAAACCGCGCCGC
GCGTCTTGTCTGTCTCTGTGTTGTTTTGTCATTTGTCGGTTCGTTATTGTTTTGGACCGTTTCTAAAAATATGGGACAGA
CCGTAACCACTCCTTTGAGTCTGACCCTAGAACACTGGGGAGACGTCCAGCGCATTGCGTCCAACCAGTCCGTGGACGTC
AAGAAGAGACGTTGGGTCACCTTCTGCTCTGCCGAATGGCCAACTTTCAATGTGGGATGGCCTCAGGATGGTACTTTTAA
TTTGGACATTATTTTACAGGTTAAATCTAAGGTGTTCTCTCCCGGTCCCCACGGACACCCGGATCAGGTCCCATACATTG
TCACCTGGGAGGCACTTGCCTATGACCCCCCTCCGTGGGTCAAACCGTTTGTCTCTCCAAAACCCCCTCCCTTACCGACA
GCTCCCGTCCTCCCGCCCGGTCCTTCTGCGCAACCTCCGTCCCGATCTGCCCTTTACCCTGCCCTTACCCCCTCTATAAA
GTCCAAACCTCCTAAGCCCCAGGTTCTCCCTGATAGCGGCGGACCTCTCATTGACCTTCTCACAGAGGACCCCCCGCCGT
ACGGAGCACAACCTTCCTCCTCTGCCAGAGAAAACAATGAAGAAGAGGCGGCCGCCACCTCCGAGGTTTCCCCCCCTTCT
CCCATGGTGTCTCGACTGCGGGGAAGGAGGGACCCTCCCGCAGCGGACTCCACCTCCTCCCAGGCATTCCCACTCCGCAT
GGGGGGAGATGGCCAGCTTCAGTATTGGCCGTTTTCCTCCTCGGACTTATACAATTGGAAAAATAATAACCCTTCCTTTT
CTGAAGACCCAGGTAAATTGACGGCCTTGATTGAGTCCGTCCTCATCACCCACCAGCCCACCTGGGACGACTGTCAGCAG
TTGTTAGGGACCCTGCTGACCGGAGAAGAAAAGCAGCGGGTGCTCCTAGAGGCTAGAAAGGCAGTCCGGGGCAATGATGG
ACGCCCCACTCAGTTGCCTAATGAAGTCAATGCTGCTTTTCCCCTTGAACGCCCCGATTGGGATTACACCACTACAGAAG
GTAGGAACCACCTAGTCCTCTATCGCCAGTTGCTCTTAGCGGGTCTCCAAAACGCGGGCAGAAGCCCCACCAATTTGGCC
AAGGTAAAAGGGATAACCCAGGGACCTAATGAGTCTCCCTCAGCCTTTTTAGAGAGACTCAAGGAGGCCTATCGCAGGTA
CACTCCTTATGACCCTGAGGACCCAGGGCAAGAAACCAATGTGTCTATGTCATTCATCTGGCAGTCTGCCCCGGATATCG
GGCGAAAGTTAGAGCGGTTAGAAGATTTAAAGAGCAAGACCTTAGGAGATTTAGTGAGGGAAGCTGAAAAGATCTTTAAT
AAGCGAGAAACCCCGGAAGAAAGAGAGGAACGTATCAGGAGAGAAACAGAGGAAAAAGAAGAACGCCGTAGGGCAGAGGA
TGAGCAGAGAGAGAAAGAAAGGGACCGCAGGAGACATAGAGAGATGAGCAAGCTCTTGGCCACTGTAGTTAGTGGACAGA
GACAGGATAGACAGGGGGGAGAGCGAAGGAGGCCCCAACTTGATAAGGACCAATGCGCCTACTGCAAAGAAAAGGGACAC
TGGGCTAAGGACTGCCCAAAGAAGCCACGAGGGCCCCGAGGACCGAGGCCCCAGACCTCCCTCCTGACCTTAGGTGACTA
GGGAGGTCAGGGTCAGGAGCCCCCCCTGAACCCAGGATAACCCTCAAAGTCGGGGGGCAACCCGTCACCTTCCTGGTAG

ATACTGGGGCCCAACACTCCGTGCTGACCCAAAATCCTGGACCCCTAAGTGACAAGTCTGCCTGGGTCCAAGGGGCTACT
GGAGGAAAGCGGTATCGCTGGACCACGGATCGCAAAGTACATCTAGCTACCGGTAAGGTCACCCACTCTTTCCTCCATGT
ACCAGACTGCCCCTATCCTCTGCTAGGAAGAGACTTGCTGACTAAACTCAAAGCCCAGATCCACTTCGAGGGATCAGGAG
CTCAGGTTGTGGGACCAATGGGACAGCCCCTGCAAGTGCTGACCCTAAACATAGAGGATGAGTATCGGCTACATGAGACC
TCAACAGAGCCGGATGTTTCTCTAGGGTCCACCTGGCTTTCTGATTTTCCCCAGGCCTGGGCGGAAACCGGGGGCATGGG
ACTGGCAGTTCGCCAAGCGCCTCTGATTATACCTCTAAAGGCAACCTCCACCCCTGTGTCCATAAAACAATACCCCATGT
CGCAAGAAGCCAGACTGGGGATCAAGCCCCACATACAGAGGCTGTTGGACCAGGGAATATTGGTACCCTGCCAGTCCCCC
TGGAATACACCCCTGCTACCCGTTAAGAAACCAGGGACTAATGATTACAGGCCTGTCCAGGATCTGAGAGAAGTTAACAA
GCGGGTAGAAGACATCCACCCCACCGTGCCCAACCCTTACAACCTCTTAAGTGGGCTCCCACCGTCCCACCAGTGGTACA
CTGTGCTTGATTTAAAGGATGCCTTTTTCTGCCTGAGACTCCACCCCACCAGTCAGCCTCTCTTCGCCTTTGAGTGGAGA
GATCCAGGGATGGGAATCTCAGGACAATTGACCTGGACCAGACTCCCACAGGGTTTCAAAAACAGTCCCACCCTGTTTGA
TGAGGCACTGCACAGAGACCTAGCAGACTTCCGGATCCAGCACCCAGACTTGATCCTGCTACAGTACGTGGATGACATAC
TACTGGCCGCCACTTCTGAGCTCGACTGCCAACAAGGTACTCGGGCCCTGTTACTAACCCTAGGAAACCTCGGGTATCGG
GCCTCGGCCAAGAAAGCCCAACTTTGCCAGAAGCAGGTCAAGTATCTGGGGTATCTTCTAAAAGAGGGTCAGAGATGGCT
GACTGAGGCCAGAAAAGAGACTGTGATGGGGCAGCCTACTCCGAAGACCCCTCGACAACTAAGGGAGTTCCTAGGGACGG
CAGGCTTCTGTCGCCTCTGGATCCCTGGGTTTGCGGAAATGGCAGCCCCCTTGTATCCTCTTACCAAAACGGGGACTCTG
TTTAATTGGGGCCCAGACCAGCAAAAGGCCTATCAAGAAATCAAACAGGCCCTTCTAACTGCCCCCGCCCTGGGATTGCC
AGATTTGACTAAGCCCTTTGAACTCTTTGTCGACGAGAAGCAGGGCTACGCCAAAGGCGTCCTAACGCAAAAACTGGGAC
CTTGGCGTCGGCCTGTGGCCTACCTGTCCAAAAAGCTAGACCCAGTGGCAGCTGGGTGGCCCCCTTGCCTACGGATGGTA
GCAGCCATTGCCATTCTGACAAAAGATGCAGGCAAGCTAACTATGGGACAGCCGCTAGTCATCCTGGCCCCCCATGCAGT
AGAGGCACTGGTCAAGCAACCCCCTGACCGCTGGCTATCCAATGCCCGCATGACCCACTACCAGGCAATGCTCCTAGACA
CTGACCGAGTTCAGTTCGGACCAGTGGTGGCCCTCAATCCTGCCACCTTGCTCCCTCTACCGGAGAAAGGAGCCCCCCAT
GATTGCCTCGAGATCTTGGCTGAAACGCATGGAACCAGACCGGACCTCACCGACCAGCCCATCCCAGACGCCGACCACAC
CTGGTATACCGATGGGAGCAGCTTTTTGCAAGAAGGACAGCGAAAAGCTGGGGCAGCAGTGACGACAGAGACCGAGGTAA
TCTGGGCGAGGGCCCTGCCAGCTGGAACGTCAGCCCAGCGAGCCGAACTGATCGCACTCACCCAAGCCCTGAAAATGGCA
GAAGGTAAGAAGCTAAATGTTTATACTGACAGCCGATATGCTTTCGCCACGGCCCATGTCCATGGAGAAATCTATAGGAG
GCGAGGGTTGCTGACCTCAGAGGGCAGAGAAATCAAAAACAAGAGCGAGATCCTGGCTTTACTGAAAGCTCTTTTCCTGC
CTAAAAGACTCAGTATAATTCACTGCCCCGGGCATCAAAAGGGAAACAGTGCTGAAGCCAGGGGCAACCGTATGGCAGAC
CAAGCGGCCCGAGAGGCAGCCATAAGGACATCTCCAGAAACTTCCACCCTCCTCATAGAGGACTCGACCCCGTATACGCC
CTCCCATTTCCACTACACTGAAACAGATCTAAAGAGATTACGAGAACTGGGAGCCACCTATAATCAGATAAAAGGATATT
GGGTCCTACAAGGCAAGCCGGTAATGCCCGATCAGTTTGTGTTTGAACTATTAGACTCCTTACACAGGCTCACTCACCTC
AGCCCTCAAAAGATGAAGGCACTCCTTGACAGAGAAGAAAGCCCCTACTACATGTTAAACAGAGACAGAACTCTCCAGTA
TGTGGCAGAATCATGCACAGCTTGTGCTCAAGTGAATGCTAGTAAAGCCAAGATCGGGGCAGGGGTACGAGTACGCGGAC
ATCGACCAGGTACCCATTGGGAAATTGACTTTACTGAAGTTAAGCCAGGACTGTACGGGTACAAGTACCTCCTAGTGTTC
GTGGACACCTTCTCTGGCTGGGTAGAAGCCTTCCCAACTAAACGTGAAACCGCCAAAGTGGTAACCAAGAAGCTATTAGA
AGAAATATTCCCAAGATTCGGGATGCCACAGGTACTGGGTTCTGACAATGGGCCTGCCTTCGTCTCCCAGGTAAGTCATT
CGGTGGCCGATTTACTGGGGATTGATTGGAAGTTACATTGCGCTTATAGACCCCAGAGTTCAGGTCAGGTAGAGAGAATG
AATAGAACCATCAAGGAGACTTTAACCAAATTAACGCTTGCAGCTGGCACTAGAGACTGGGTACTCCTACTCCCCTTGGC
CCTCTACCGAGCCCGGAATACTCCGGGCCCACATGGACTTACTCCGTATGAAATTCTGTATGGGGCACCCCCGCCCCTTG
TCAATTTTCATGATCCTGAAATGTCAAAGTTAACTAATAGTCCCTCTCTCCAAGCTCACTTACAGGCCCTCCAAGCAGTA
CAACGAGAGGTCTGGAAGCCACTGGCCGCTGCTTATCAGGACCAGCTGGATCAGCCAGTGATACCACACCCCTTCCGTGT
CGGTGACGCCGTGTGGGTACGCCGGCACCAGACTAAGAACTTAGAACCCCGCTGGAAAGGACCCTACACCGTCCTGCTGA
CCACCCCCACCGCTCTCAAAGTAGACGGCATCTCTGCGTGGATACACGCCGCTCACGTAAAGGCGGCGACAACTCCTCCA
GCCGGAACAGCATGGAAGGTCCAGCGTTCTCAAAACCCCTTAAAGATAAGATTAACCCGTGGGGCCCCCTAATAGTTATG
GGGATCTTGGTGAGGGCAGGAGCCTCGGTACAACGTGACAGCCCTCACCAGATCTTCAATGTTACTTGGAGAGTTACCAA
CCTAATGACAGGACAAACAGCTAACGCCACCTCCCTCCTGGGGACGATGACAGACACCTTCCCTAAACTATATTTTGACC
TGTGTGATTTAGTAGGAGACTACTGGGATGACCCAGAACCCGATATTGGGGATGGTTGCCGCACTCCCGGGGGAAGAAGA
AGGACAAGACTGTATGACTTCTATGTTTGCCCCGGTCATACTGTACCAATAGGGTGTGGAGGGCCGGGAGAGGGCTACTG
TGGCAAATGGGGATGTGAGACCACTGGACAGGCATACTGGAAGCCATCATCATCATGGGACCTAATTTCCCTTAAGCGAG
GAAACACTCCTAAGGATCAGGGCCCCTGTTATGATTCCTCGGTCTCCAGTGGCGTCCAGGGTGCCACACCGGGGGGTCGA
TGCAACCCCCTGGTCTTAGAATTCACTGACGCGGGTAGAAAGGCCAGCTGGGATGCCCCCAAAGTTTGGGGACTAAGACT
CTATCGATCCACAGGGGCCGACCCGGTGACCCGGTTCTCTTTGACCCGCCAGGTCCTCAATGTAGGACCCCGCGTCCCCA
TTGGGCCTAATCCCGTGATCACTGACCAGCTACCCCCATCCCAACCCGTGCAGATCATGCTCCCCAGGCCTCCTCATCCT
CCTCCTTCAGGCACGGTCTCTATGGTACCTGGGGCTCCCCCGCCTTCTCAACAACCTGGGACGGGAGACAGGCTGCTAAA
TCTGGTAGAAGGAGCCTACCAAGCACTCAACCTCACCAGTCCTGACAAAACCCAAGAGTGCTGGTTGTGTCTGGTATCGG
GACCCCCCTACTACGAAGGGGTTGCCGTCCTAGGCACCTACTCCAACCATACCTCTGCCCCAGCTAACTGCTCCGTGGCC
TCCCAACACAAGCTGACCCTGTCCGAAGTAACCGGACAGGGACTCTGCGTAGGAGCAGTTCCCAAAACCCATCAGGCCCT

GTGTAATACCACCCAAAAGACGAGCGACGGGTCCTACTATCTGGCTGCTCCCGCCGGGACCATCTGGGCTTGCAACACCG
GGCTCACTCCCTGCCTATCTACTACTGTACTCAACCTCACCACCGATTACTGTGTCCTGGTTGAGCTCTGGCCAAAGGTG
ACCTACCACTCCCCTGGTTATGTTTATGGCCAGTTTGAGAGAAAAACCAAATATAAAAGAGAGCCGGTGTCATTAACTCT
GGCCCTGCTGTTAGGAGGACTTACTATGGGCGGCATAGCTGCAGGAGTAGGAACAGGGACTACAGCCCTAGTGGCCACCA
AACAATTCGAGCAACTCCAGGCAGCCATACATACAGACCTTGGGGCCTTAGAAAAATCAGTCAGTGCCCTAGAAAAGTCT
CTGACCTCGTTGTCTGAGGTGGTCCTACAGAACCGGAGAGGATTAGATCTGCTGTTCCTAAAAGAAGGAGGATTATGTGC
TGCCCTAAAAGAAGAATGCTGTTTCTACGCGGACCACACTGGCGTAGTAAGGGATAGCATGGCTAAGCTAAGAGAGAGAC
TAAACCAGAGACAAAAATTGTTCGAATCAGGACAAGGGTGGTTTGAGGGACTGTTTAACAGGTCCCCATGGTTCACGACC
CTGACATCCACCATTATGGGCCCTCTGATAGTACTTTTATTAATCCTACTCTTCGGACCCTGTATTCTCAACCGCTTGGT
CCAGTTTGTAAAAGACAGAATTTCGGTGGTGCAGGCCCTGGTCCTGACCCAACAGTATCACCAACTCAAATCAATAGATC
CAGAAGCAGTGGAAACACGTGAATAAAAGATTTTATTCAGTTTCCAGAAAGAGGGGGGAATGAAAGACCCCACCATAAGG
CTTAGCAAGCTAGCTGCAGTAACGCCATTTTGCAAGGCATGAAAAAGTACCAGAGCTGAGTTCTCAAAAGTTACAAGGAA
GTTCAGTTAAAGATTAACAGTTAAAAATCAAGGCTGAATAATACTAGGACAAGGGCCAAACAGAATATCGGTGGTCAAGC
GCCTGGGCCCCGGCTCAGGGCCAAGAACAGATGGTACCCAGATAAAGCGGAACCAGCAACAGTTTCTGAAAAAGTCCCAC
CTCAGTTTCAGGTTCCCCAAATGACCAGGAAATACCCCAAGCCTTGATTTGAACTAACCACTCAGCTCGCTTCTCGCTTC
TGTACCCACGCTTTTTGCTCCCCAGCCCCAGCCCTATAAAAAGGGTAAGAACTCCACACTCGGCGCGCCAGTCCTCCGAT
AGACTGAGTCGCCCGGGTACCCGTGTTCCCAATAAAGCCTCTTGCTGATTACATCCGAATCGTGGTCTCGCTGATCCTTG
GGAGGGTCTCCTCAGATTGATTGACCACCCACCTCGGGGGTCTTTCA
>Xmv15
TGAAAGACCCCACCATAAGGCTTAGCAAGCTAGCTGCAGTAACGCCATTTTGCAAGGCATGAAAAAGTACCAGAGCTGAG
TTCTCAAAAGTTACAAGGAAGTTCAGTTAAAGATTAACAGTTAAAAATCAAGGCTGAATAATACTAGGACAAGGGCCAAG
AACCGATGGTACCCACCTGGGCCCCGGCTCAAGGCCAAGAACCGATGGTACCCACCTGGGCCCCGGCTCAGGGCCAAGAA
CAGATGGTACCCAGATAAGGCGGAACCAGCAACAGTTTCTGAAAAAGTCCCACCTCAGTTTCAGGTTCCCCAAATGACCA
GGAAATACCCCAAGCCTTGATTTGAACTAACCACTCAGCTCGCTTCTCGCTTCTGTACCCGCGCTTTTTGCTCCCCAGCC
CTATAAAAAGGGTAAAAACCCCACACTCGGCGCGCCAGTCCTCCGATAGACTGAGTCGCCCGGGTACCCGTGTTCCCAAT
AAAGCCTCTTGCTGATTACATCCGAATCGTGGTCTCGCTGATCCTTGGGAGGGTCTCCTCAGATTGATTGACTACCCACC
TCGGGGGTCTTTCATTTGGAGGTCCCACCGAGATTAGGAGACCCCTGCCCAGGGACCACCGACCCCCGCCGGGAGGTAAG
CTGGCCAGCGGTCGTTTCGTGTCTGTCTCTGTCTCCGTGCGTGTTTGTGCCGGCATCTAATGTTTGCGCCTGCGTCTGTA
CTAGTTGGCTAACTAGATCTGTATCTGGCGGTTCCGCGGAAGAACTGACGAGTTCGTATTCCCGGCCGCAGCCCTGGGAG
ACGTCCCAGCGGCCTCGGGGGCCCGTTTTGTGGCCCATTCTGTATCAGTTAACCTACCCGAGTCGGACTTTTTGGAGCTC
CTCCACTGTACGTGGCTTTGTCGGGGGACGAGAGGCAGAGACACTTCCCTCCCCCGTCTGAATTTTTGCTTTCGGTTTTA
CGCCGAAACCGCGCCGCGCGTCTGATTTGTTTGTTGTTCTTTTGTTCTTCGTTAGTTTTCTCCTGTCTTTAAGTGTTTTT
GAGATCATGGGACAGACCGTAACTACCCCTCTGAGTCTAACCTTGCAGCACTGGGGAGATGTCCAGCGCATTGCATCCAA
TCAGTCTGTGGATGTCAGGAAGAGGCGCTGGGTTACCTTCTGTTCCGCCGAATGGCCAACTTTCAATGTGGGATGGCCTC
AGGATGGTACTTTTAATTTAGGTATTATCTCTCAGGTCAAGTCTAGAGTGTTTTGTCCTGGTCCCCACGGACACCCGGAT
CAGGTCCCATATATCGTCACCTGGGAGGCACTTGCCTATGACCCCCCTCCGTGGGTCAAACCGTTTGTCTCTCCAAAACC
CCCTCCTTTACCGACAGCTCCCGTCCTCCCGCCCGGTCCTTCTGCGCAACCTCCGTCCCGATCTGCCCTTTACCCTGCCC
TTACCCCCTCTATAAAGCCCAAACCTCCTAAGCCCCAGGTTCTCCCTGATAACGGCGGACCTCTCATTGACCTTCTCACA
GAGGACCCCCCGCCGTACGGAGCACAACCTTCCTCCTCTGCCAGAGGGAACGATGAAGAAGAGGCGGCCACCACCTCCGA
GGTTTCCCCCCCTTCTCCCATGGTGTCTCGACTGCGGGGAAGGAGAGACCCTCCCGCAGCGGACTCCACCTCCTCCCAGG
CATTCCCACTCCGCATGGGGGGAGATGGCCAGCTTCAGTACTGGCCGTTTTCCTCCTCTGACTTATATAATTGGAAAAAT
AATAACCCTTCCTTTTCTGAAGATCCAGGTAAATTGACGGCCTTGATTGAGTCCGTCCTCATCACCCACCAGCCCACCTG
GGACGACTGTCAGCAGTTGTTGGGGACCCTGCTGACCGGAGAAGAAAAGCAGCGGGTGCTCCTAGAGGCTAGAAAGGCAG
TCCGGGGCAATGATGGACGCCCCACTCAGTTGCCTAATGAAGTCAATGCTGCTTTTCCCCTTGAACGCCCCGATTGGGAT
TACACCACTACAGAAGGTAGGAACCACCTAGTCCTCTATCGCCAGTTGCTCTTAGCGGGTCTCCAAAACGCGGGCAGGAG
CCCCACCAATTTGGCCAAGGTAAAAGGGATAACCCAGGGACCTAATGAGTCTCCCTCAGCCTTTTTAGAGAGACTCAAGG
AGGCCTATCGCAGGTACACTCCTTATGACCCTGAGGACCCAGGGCAAGAAACCAATGTGTCTATGTCATTCATCTGGCAG
TCTGCCCCGGATATCGGGCGAAAGTTAGAGCGGTTAGAAGATTTAAAGAGCAAGACCTTAGGAGACTTAGTGAGGGAAGC
TGAAAGGATCTTTAATAAGCGAGAAACCCCGGAAGAAAGAGAGGAACGTATCAGGAGAGAAACAGAGGAAAAAGAAGAAC
GCCGTAGGGCAGAGGATGAGCAGAGAGAGAAAGAAAGGGACCGCAGAAGACATAGAGAGATGAGCAAGCTCTTGGCCACT
GTAGTTATTGGTCAGAGACAGGATAGACAGGGGGGAGAGCGAAGGAGGCCCCAACTTGATAAGGACCAATGCGCCTACTG
CAAAGAAAAGGGACACTGGGCTAAGGACTGCCCAAAGAAGCCACGAGGGCCCCGAGGACCGAGGCCCCAGACCTCCCTCC
TGACCTTAGGTGACTAGGGAGGTCAGGGTCAGGAGCCCCCCCTGAACCCAGGATAACCCTCAAAGTCGGGGGGCAACCCG
TCACCTTCCTGGTAGATACTGGGGCCCAACACTCCGTGCTGACCCAAAATCCTGGACCCCTAAGTGACAAGTCTGCCTGG
GTCCAAGGGGCTACTGGAGGAAAGCGGTATCGCTGGACCACGGATCGCAAAGTACATCTAGCTACCGGTAAGGTCACCCA
CTCTTTCCTCCATGTACCAGACTGCCCCTATCCTCTGCTAGGAAGAGACTTGCTGACTAAACTAAAAGCCCAGATCCACT
TCGAGGGATCAGGAGCTCAGGTTGTGGGACCAATGGGACAGCCCCTGCAAGTGCTGACCCTAAACATAGAAGATGAGTAT

CGGCTACATGAGACCTCAAAAGAGCCGGATGTTTCTCTAGGGTCCACATGGCTTTCTGATTTTCCCCAGGCCTGGGCAGA
AACCGGGGGCATGGGACTGGCAGTTCGCCAAGCGCCTCTGATTATACCTCTAAAGGCAACCTCTACCCCCGTGTCCATAA
AACAATACCCCATGTCACAGGAAGCCAGACTGGGGATCAAGCCCCACATACAGAGGCTGTTGGACCAGGGAATACTGGTA
CCCTGCCAGTCCCCCTGGAACACGCCCCTGCTACCCGTTAAGAAACCAGGGACTAATGATTACAGGCCTGTCCAGGATCT
GAGAGAAGTCAACAAGCGGGTGGAAGACATCCACCCCACCGTGCCCAACCCTTACAACCTCTTGAGCGGGCTCCCACCGT
CCCACCAGTGGTACACTGTGCTTGATTTAAAGGATGCCTTTTTCTGCCTGAGACTCCACCCCACCAGTCAGCCTCTCTTC
GCCTTTGAGTGGAGAGATCCAGGGATGGGAATCTCAGGACAATTGACCTGGACCAGACTCCCACAGGGTTTCAAAAACAG
TCCCACCCTGTTTGATGAGGCACTACACAGAGACCTAGCAGACTTCCGGATCCAGCACCCAGACTTGATCCTGCTACAGT
ACGTGGATGACATACTACTGGCCGCCACTTCTGAGCTCGACTGCCAACAAGGTACTCGGGCCCTGTTACAAACCCTAGGA
AACCTCGGGTATCGGGCCTCGGCCAAGAAAGCCCAACTTTGCCAGAAACAGGTCAAGTATCTGGGGTATCTTCTAAAAGA
GGGTCAGAGATGGCTGACTGAGGCCAGAAAAGAGACTGTGATGGGGCAGCCTACTCCGAAGACCCCTCGACAACTAAGGG
AGTTCCTAGGGACGGCAGGCTTCTGTCGCCTCTGGATCCCTGGGTTTGCGGAAATGGCAGCCCCCTTGTATCCTCTTACC
AAAACGGGGACTCTGTTTAATTGGGGCCCAGACCAGCAAAAGGCCTATCAAGAAATCAAACAGGCCCTTCTAACTGCCCC
CGCCCTGGGATTGCCAGATTTGACTAAGCCCTTTGAACTCTTTGTCGACGAGAAGCAGGGCTACGCCAAAGGCGTCCTAA
CGCAAAAACTGGGACCTTGGCGTCGGCCTGTGGCCTACCTGTCCAAAAAGCTAGACCCAGTGGCAGCTGGGTGGCCCCCT
TGCCTACGGATGGTAGCAGCCATTGCCGTTCTGACAAAAGATGCAGGCAAGCTAACTATGGGACAGCCGCTAGTCATCCT
GGCCCCCCATGCAGTAGAGGCACTGGTCAAGCAACCCCCTGACCGCTGGCTATCCAACGCCCGCATGACCCACTACCAGG
CAATGCTCCTAGACACTGACCGAGTTCAGTTCGGACCAGTGGTGGCCCTCAATCCTGCCACCTTGCTCCCTCTACCGGAA
AAAGGAGCCCCCCATGATTGCCTCGAGATCTTGGCTGAAACGCATGGAACCAGACCGGATCTCACCGACCAGCCCATCCC
AGACGCCGACCACACCTGGTATACCGATGGGAGCAGCTTTTTGCAAGAAGGACAGCGAAAGGCTGGGGCAGCAGTGACGA
CTGAAACCGAGGTAATCTGGGCGAGGGCCCTGCCAGCTGGAACGTCAGCCCAGCGAGCCGAACTGATCGCACTCACCCAA
GCCCTGAAAATGGCAGAAGGTAAGAAGCTAAATGTTTACACTGATAGCCGCTATGCCTTCGCTACGGCCCATGTTCATGG
GGAAATATATAGGAGACGGGGGTTGCTGACCTCAGAAGGCAAGGAAATCAAGAACAAAAGCGAGATCCTAGCCTTGCTGA
AAGCCCTCTTTTTGCCAAAGAGGCTCAGTATTATCCATTGCCCGGGACACCAAAAAGGGAACAGTGCTGAAGCCAGGGGC
AACCGAATGGCGGATCAAGCAGCCAGGGAGGCAGCCATGAGAGAGATCACAGAAACTTCCACGCTCTTAATAGAGGATTC
GTCCCCGTATACTCCCAGTTACTTCCACTACACAGAGACTGACAGAAAAGACCTTCTGAGACTGGGAGCCGTATACGATG
AGGACAAAAGGTATTGGGTCCTGCAGGGAAAACCTGTAATGCCTGACCAATTCACCTTTGAGCTCTTAGACTCCCTACAC
CGGCTCACCCACCTCAGCTATCAGAAGATGAAGGCACTCCTTGACCGGGAAGAGAGCCCCTACTACATGTTAAATCGGGG
TAGAACTCTCCAGCACGTAACAGAATCCTGTACAGCTTGTGCTCAAGTAAATGCTAGTAAAGCCAAAATCGGAGCAAGGG
TGCGAGCGCGGGGACACCGACCCGGCACCCATTGGGAAATCGATTTTACCGAAGTTAAGCCAGGGCTGTATGGACATAAG
TACCTCCTGGTGTTTGTGGACACATTCTCTGGCTGGGTAGAAGCATTTCCAACCAAGCGTGAAACTGCCAGGGTTGTGAC
CAAGAAGCTGCTTGAAGAAATTTTTCCAAGATTCGGGATGCCCCAGGTATTGGGGACAGATAATGGGCCTGCCTTCATCT
CCCAGGTAAGTCAGTCGGTGGCCAAGTTGCTGGGGATTGATTGGAAACTGCATTGTGCTTACAGACCCCAGAGTTCAGGT
CAGGTAGAAAGAATGAACAGGACAATCAAGGAGACTTTAACCAAATTAACGCTTGCAGCTGGCACTAGAGACTGGGTACT
CCTACTCCCCCTGGCCCTCTACCGAGCCCGGAATACTCCGGGCCCCCATGGACTTACTCCGTATGAAATTCTGTATGGGG
CACCCCCGCCCCTTGTCAATTTTCATGATCCTGAAATGTCAAAGTTAACTAATAGTCCCTCTCTCCAAGCTCACTTACAG
GCCCTCCAAGCAGTACAACGAGAGGTCTGGAAGCCACTGGCCGCTGCTTATCAGGACCAGCTGGATCAGCCAGTGATACC
ACACCCCTTCCGCGTCGGTGACGCCGTGTGGGTACGCCGGCACCAGACTAAGAACTTAGAACCCCGCTGGAAAGGACCCT
ACACCGTCCTGCTGACCACCCCCACCGCTCTCAAAGTAGACGGCATCTCTGCGTGGATACACGCCGCTCACGTAAAGGCG
GCGACAACTCCTCCGGCCGGAACAGCATGGAAGGTCCAGCGTTCTCAAAATCCCTTAAAGATAAGATTAACCCGTGGGGC
CCCCTGATAGTTATGGGGATCTTAGTGAGGGCAGGAGCCTCGGTACAACGTGACAGCCCTCACCAGGTCTTCAATGTTAC
TTGGAGAGTTACCAACCTAATGACAGGACAAACAGCTAACGCCACCTCCCTCCTGGGGACGATGACAGACACCTTCCCTA
AACTATATTTTGACCTGTGTGATTTAGTAGGAGACTACTGGGATGACCCAGATGGAGATGGTTGCCGCTCTCCCGGGGGA
AGAAAAAGGACAAGACTGTATGACTTCTATGTTTGCCCCGGTCATACTGTACCAATAGGGTGTGGAGGGCCGGGAGAGGG
CTACTGTGGCAAATGGGGATGTGAGACCACTGGACAGGCATACTGGAAGCCATCATCATCATGGGACCTAATTTCCCTTA
AGCGAGGAAACACTCCTAAGGATCAGGGCCCCTGTTATGATTCCTCGGTCTCCAGTGGCGTCCAGGGTGCCACACCGGGG
GGTCGATGCAACCCCCTAGTCTTAGAATTCACTGACGCGGGTAAAAAGGCCAGCTGGGATGCCCCCAAAGTTTGGGGACT
AAGACTCTACCGATCCACAGGGGCCGACCCGGTGACCCGGTTCTCTTTGACCCGCCGGGTCCTCAATGTAGGACCCCGCG
TCCCCATTGGGCCTAATCCCGTGATCACTGACCAGCTACCCCCATCCCAACCCGTGCAGATCATGCTCCCCAGGCCTCCT
CATCCTCCTCCTTCAGGCACGGTCTCTATGGTACCTGGGGCTCCCCCGCCTTCTCAACAGCCTGGGACGGGAGACAGGCT
GCTAAATCTGGTAGACGGAGCCTACCAAGCACTCAACCTCACCAGTCCTGACAAAACCCAAGAGTGCTGGTTGTGTCTGG
TATCGGGACCCCCCTACTACGAAGGGGTTGCCGTCCTAGGTACCTACTCCAACCATACCTCTGCCCCAGCTAACTGCTCC
GTGGCCTCCCAACACAAGCTGACCCTGTCCGAAGTGACCGGACAGGGACTCTGCGTAGGAGCAGTTCCCAAAACCCATCA
GGCCCTGTGTAATACCACCCAGAAGACGAGCGACGGGTCCTACTATCTGGCTGCTCCCGCCGGGACCATCTGGGCTTGCA
ACACCGGGCTCACTCCCTGCCTATCTACTACTGTACTCAACCTCACCACCGATTACTGTGTCCTGGTTGAGCTCTGGCCA
AAGGTGACCTACCACTCCCCTGGTTATGTTTATGGCCAGTTTGAGAGAAAAACCAAATATAAAAGAGAGCCGGTGTCATT
AACTCTGGCCCTGCTGTTGGGAGGACTTACTATGGGCGGCATAGCTGCAGGAGTAGGAACAGGGACTACAGCCCTAGTGG

CCACCAAACAATTCGAGCAGCTCCAGGCAGCCATACATACAGACCTTGGGGCCTTAGAAAAATCAGTCAGTGCCCTAGAA
AAGTCTCTGACCTCGTTGTCTGAGGTGGTCCTACAGAACCGGAGAGGATTAGATCTGCTGTTCCTAAAAGAAGGAGGATT
ATGTGCTGCCCTAAAAGAAGAATGCTGTTTCTACGCGGACCACACTGGCGTAGTGAGAGATAGCATGGCAAAGCTAAGAG
AAAGGTTAAACCAGAGACAAAAATTGTTCGAATCAGGACAAGGGTGGTTTGAGGGACTGTTTAACAGGTCCCCATGGTTC
ACGACCTTGATATCCACCATTATGGGCCCCTTGATAATACTTTTATTAATCCTACTCCTCGGACCCTGTATTCTCAACCG
CTTGGTCCAGTTTGTAAAAGACAGAATTTCGGTGGTGCAGGCCCTGGTTCTGACCCAACAGTATCACCAACTCAAATCAA
TAGATCCAGAAGAAGTGGAATCACGTGAATAAAAGATTTTATTCAGTTTCCAGAAAGAGGGGGGAATGAAAGACCCCACC
ATAAGGCTTAGCAAGCTAGCTGCAGTAACGCCATTTTGCAAGGCATGAAAAAGTACCAGAGCTGAGTTCTCAAAAGTTAC
AAGGAAGTTCAGTTAAAGATTAACAGTTAAAAATCAAGGCTGAATAATACTAGGACAAGGGCCAAGAACCGATGGTACCC
ACCTGGGCCCCGGCTCAAGGCCAAGAACCGATGGTACCCACCTGGGCCCCGGCTCAAGGCCAAGAACCGATGGTACCCAC
CTGGGCCCCGGCTCAGGGCCAAGAACAGATGGTACCCAGATAAGGCGGAACCAGCAACAGTTTCTGAAAAAGTCCCACCT
CAGTTTCAGGTTCCCCAAATGACCAGGAAATACCCCAAGCCTTGATTTGAACTAACCACTCAGCTCGCTTCTCGCTTCTG
TACCCGCGCTTTTTGCTCCCCAGCCCTATAAAAAGGGTAAAAACCCCACACTCGGCGCGCCAGTCCTCCGATAGACTGAG
TCGCCCGGGTACCCGTGTTCCCAATAAAGCCTCTTGCTGATTACATCCGAATCGTGGTCTCGCTGATCCTTGGGAGGGTC
TCCTCAGATTGATTGACTACCCACCTCGGGGGTCTTTCA
>Xmv16
TGAAAGACCCCACCATAAGGCTTAGCAAGCTAGCTGCAGTAACGCCATTTTGCAAGGCATGAAAAAGTACCAGAGCTGAG
TTCTCAAAAGTTACAAGGAAGTTCAGTTAAAGATTAACAGTTAAAAATCAAGGCTGAATAATACTAGGACAAGGGCCAAG
AACCGATGGTGCCCACCTGGGCCCCGGCTCAAGGCCAAGAACCGGTGGTACCCACCTGGGCCCCGGCTCAGGGCCAAGAA
CAGATGGTACCCAGATAAGGCGGAACCAGCAACAGTTTCTGAAAAAGTCCCACCTCAGTTTCAGGTTCCCCAAATGACCA
GGAAATACCCCCAGCCTTGATTTGAACTAACCACTCAGCTCGCTTCTCGCTTCTGTACCCGCGCTTTTTGCTCCCCAGCC
CTATAAAAAGGGTAAGAACTCCACACTCGGCGCGCCAGTCCTCCGACAGACTGAGTCGCCCGGGTACCCGTGTTCCCAAT
AAAGCCTCATGCTGATTACATCCGAATCGTGGTCTCGCTGATCCTTGGGAGGGTCTCCTCAGATTGATTGACCACCCACC
TCGGAGGTCTTTCATTTGGAGGCCCCAGCGAGATAAGGACCAATGCGCCTACTGCAAAGAAAAGGGACACTGGGCTAAGG
ACTGCCCAAAGAAGCCACGAGGGCCCCGAGGACCGAGGCCCCAGACCTCCCTCCTGACCTTAGGTGACTAGGGAGGTCAG
GGTCAGGAGCCCCCCCCTGAACCCAGGATAACCCTCAAAGTCGGGGGGCAACCCGTCACCTTCCTGGTAGATACTGGGGC
CCAACACTCCGTGCTGACCCAAAATCCTGGACCCCTAAGTGACAAGTCTGCCTGGGTCCAAGGGGCTACTGGAGGAAAGC
GGTATCGCTGGACCACGGATCGCAAAGTACATCTAGCTACCGGTAAGGTCACCCACTCTTTCCTCCATGTACCAGACTGC
CCCTATCCTCTGCTAGGAAGAGACTTGCTGACTAAACTCAAAGCCCAGATCCACTTCGAGGGATCAGGAGCTCAGGTTGT
GGGACCAATGGGACAGCCCCTGCAAGTGCTGACCCTAAACATAGAGGATGAGTATCGGCTACATGAGACCTCAACAGAGC
CGGATGTTTCTCTAGGGTCCACCTGGCTTTCTGATTTTCCCCAGGCCTGGGCGGAAACCGGGGGCATGGGACTGGCAGTT
CGCCAAGCGCCTCTGATTATACCTCTAAAGGCAACCTCCACCCCTGTGTCCATAAAACAATACCCCATGTCGCAAGAAGC
CAGACTGGGGATCAAGCCCCACATACAGAGGCTGTTGGACCAGGGAATATTGGTACCCTGCCAGTCCCCCTGGAATACAC
CCCTGCTACCCGTTAAGAAACCAGGGACTAATGATTACAGGCCTGTCCAGGATCTGAGAGAAGTTAACAAGCGGGTAGAA
GACATCCACCCCACCGTGCCCAACCCTTACAACCTCTTAAGTGGGCTCCCACCGTCCCACCAGTGGTACACTGTGCTTGA
TTTAAAGGATGCCTTTTTCTGCCTGAGACTCCACCCCACCAGTCAGCCTCTCTTCGCCTTTGAGTGGAGAGATCCAGGGA
TGGGAATCTCAGGACAATTGACCTGGACCAGACTCCCACAGGGTTTCAAAAACAGTCCCACCCTGTTTGATGAGGCACTG
CACAGAGACCTAGCAGACTTCCGGATCCAGCACCCAGACTTGATCCTGCTACAGTACGTGGATGACATACTACTGGCCGC
CACTTCTGAGCTCGACTGCCAACAAGGTACTCGGGCCCTGTTACTAACCCTAGGAAACCTCGGGTATCGGGCCTCGGCCA
AGAAAGCCCAACTTTGCCAGAAGCAGGTCAAGTATCTGGGGTATCTTCTAAAAGAGGGTCAGAGATGGCTGACTGAGGCC
AGAAAAGAGACTGTGATGGGGCAGCCTACTCCGAAGACCCCTCGACAACTAAGGGAGTTCCTAGGGACGGCAGGCTTCTG
TCGCCTCTGGATCCCTGGGTTTGCGGAAATGGCAGCCCCCTTGTATCCTCTTACCAAAACGGGGACTCTGTTTAATTGGG
GCCCAGACCAGCAAAAGGCCTATCAAGAAATCAAACAGGCCCTTCTAACTGCCCCCGCCCTGGGATTGCCAGATTTGACT
AAGCCCTTTGAACTCTTTGTCGACGAGAAGCAGGGCTACGCCAAAGGCGTCCTAACGCAAAAACTGGGACCTTGGCGTCG
GCCTGTGGCCTACCTGTCCAAAAAGCTAGACCCAGTGGCAGCTGGGTGGCCCCCTTGCCTACGGATGGTAGCAGCCATTG
CCATTCTGACAAAAGATGCAGGCAAGCTAACTATGGGACAGCCGCTAGTCATCCTGGCCCCCCATGCAGTAGAGGCACTG
GTCAAGCAACCCCCTGACCGCTGGCTATCCAATGCCCGCATGACCCACTACCAGGCAATGCTCCTAGACACTGACCGAGT
TCAGTTCGGACCAGTGGTGGCCCTCAATCCTGCCACCTTGCTCCCTCTACCGGAGAAAGGAGCCCCCCATGATTGCCTCG
AGATCTTGGCTGAAACGCATGGAACCAGACCGGACCTCACCGACCAGCCCATCCCAGACGCCGACCACACCTGGTATACC
GATGGGAGCAGCTTTTTGCAAGAAGGACAGCGAAAAGCTGGGGCAGCAGTGACGACAGAGACCGAGGTAATCTGGGCGAG
GGCCCTGCCAGCTGGAACGTCAGCCCAGCGAGCCGAACTGATCGCACTCACCCAAGCCCTGAAAATGGCAGAAGGTAAGA
AGCTAAATGTTTATACTGACAGCCGATATGCTTTCGCCACGGCCCATGTCCATGGAGAAATCTATAGGAGGCGAGGGTTG
CTGACCTCAGAGGGCAGAGAAATCAAAAACAAGAGCGAGATCCTGGCTTTACTGAAAGCTCTTTTCCTGCCTAAAAGACT
CAGTATAATTCACTGCCCCGGGCATCAAAAGGGAAACAGTGCTGAAGCCAGGGGCAACCGTATGGCAGACCAAGCGGCCC
GAGAGGCAGCCATAAGGACATCTCCAGAAACTTCCACCCTCCTCATAGAGGACTCGACCCCGTATACGCCCTCCCATTTC
CACTACACTGAAACAGATCTAAAGAGATTACGAGAACTGGGAGCCACCTATAATCAGATAAAAGGATATTGGGTCCTACA
AGGCAAGCCGGTAATGCCCGATCAGTTTGTGTTTGAACTATTAGACTCCTTACACAGGCTCACTCACCTCAGCCCTCAAA

AGATGAAGGCACTCCTTGACAGAGAAGAAAGCCCCTACTACATGTTAAACAGAGACAGAACTCTCCAGTATGTGGCAGAA
TCATGCACAGCTTGTGCTCAAGTGAATGCTAGTAAAGCCAAGATCGGGGCAGGGGTACGAGTACGCGGACATCGACCAGG
TACCCATTGGGAAATTGACTTTACTGAAGTTAAGCCAGGACTGTACGGGTACAAATACCTCCTAGTGTTCGTGGACACCT
TCTCTGGCTGGGTAGAAGCCTTCCCAACTAAACGTGAAACCGCCAAGGTGGTAACCAAGAAGCTATTAGAAGAAATATTC
CCAAGATTCGGGATGCCACAGGTACTGGGTTCTGACAATGGGCCTGCCTTCGTCTCCCAGGTAAGTCATTCGGTGGCCGA
TTTACTGGGGATTGATTGGAAGTTACATTGTGCTTATAGACCCCAGAGTTCAGGTCAGGTAGAGAGAATGAATAGAACCA
TCAAGGAGACTTTAACCAAATTAACGCTTGCAGCTGGCACTAGAGACTGGGTACTCCTACTCCCCCTGGCCCTCTACCGA
GCCCGGAATACTCCGGGCCCCCATGGACTTACTCCGTATGAAATCCTGTATGGGGCACCCCCGCCCCTCGTCAATTTTCA
TGAGCCTGAAATGTCAAAGTTAACTAATAGTCCCTCTCTCCAAGCTCACTTACAGGCCCTCCAAGCAGTACAACGAGAGG
TCTGGAAGCCACTGGCCGCTGCTTATCAGGACCAGCTGGATCAGCCAGTGATACCACACCCCTTCCGTGTCGGTGACGCC
GTGTGGGTACGCCGGCACCAGACTAAGAACTTAGAACCCCGCTGGAAAGGACCCTACACCGTCCTGCTGACCACCCCCAC
CGCTCTCAAAGTAGACGGCATCTCCGCGTGGATACACGCCGCTCACGTAAAGGCGGCGACAACTCCTCCAGCCGGAACAG
CATGGAAGGTCCAGCGTTCTCAAAACCCCTTAAAGATAAGATTAACCCGTGGGGCCCCCTAATAGTTATGGGGATCTTGG
TGAGGGCAGGAGCCTCGGTACAACGTGACAGCCCTCACCAGATCTTCAATGTTACTTGGAGAGTTACCAACCTAATGACA
GGACAAACAGCTAACGCCACCTCCCTCCTGGGGACGATGACAGACACCTTCCCTAAACTATATTTTGACCTGTGTGATTT
AGTAGGAGACTACTGGGATGACCCAGAACCCGATATTGGGGATGGTTGCCGCACTCCCGGGGGAAGAAGAAGGACAAGAC
TGTATGACTTCTATGTTTGCCCCGGTCATACTGTACCAATAGGGTGTGGAGGGCCGGGAGAGGGCTACTGTGGCAAATGG
GGATGTGAGACCACTGGACAGGCATACTGGAAGCCATCATCATCATGGGACCTAATTTCCCTTAAGCGAGGAAACACTCC
TAAGGATCAGGGCCCCTGTTATGATTCCTCGGTCTCCAGTGGCGTCCAGGGTGCCACACCGGGGGGTCGATGCAACCCCC
TGGTCTTAGAATTCACTGACGCGGGTAAAAAGGCCAGCTGGGATGCCCCCAAAGTTTGGGGACTGAGACTCTATCGATCC
ACAGGGGCCGACCCGGTGACCCGGTTCTCTTTGACCCGCCAGGTCCTCAATGTAGGACCCCGCGTCCCCATTGGGCCTAA
TCCCGTGATCACTGACCAGCTACCCCCATCCCAACCCGTGCAGATCATGCTCCCCAGGCCTCCTCATCCTCCTCCTTCAG
GCACGGTCTCTATGGTACCTGGGGCTCCCCCGCCTTCTCAACAACCTGGGACGGGAGACAGGCTGCTAAATCTGGTAGAA
GGAGCCTACCAAGCACTCAACCTCACCAGTCCTGACAAAACCCAAGAGTGCTGGTTGTGTCTGGTATCGGGACCCCCCTA
CTACGAAGGGGTTGCCGTCCTAGGTACCTACTCCAACCATACCTCTGCCCCAGCTAACTGCTCCGTGGCCTCCCAACACA
AGCTGACCCTGTCCGAAGTGACCGGACAGGGACTCTGCGTAGGAGCAGTTCCCAAAACCCATCAGGCCCTGTGTAATACC
ACCCAGAAGACGAGCGACGGGTCCTACTATCTGGCTGCTCCCGCCGGGACCATCTGGGCTTGCAACACCGGGCTCACTCC
CTGCCTATCTACTACTGTACTCAACCTCACCACCGATTACTGTGTCCTGGTTGAGCTCTGGCCAAAGGTGACCTACCACT
CCCCTGGTTATGTTTATGGCCAGTTTGAGAGAAAAACCAAATATAGAAGGGAGCCGGTGTCATTAACTCTGGCCCTGCTG
TTGGGAGGACTTACTATGGGCGGCATAGCTGCAGGAGTAGGAACAGGGACTACAGCCCTAGTGGCCACCAAACAATTCGA
GCAGCTCCAGGCAGCCATACATACAGACCTTGGGGCCTTAGAAAAATCAGTCAGTGCCCTAGAAAAGTCTCTGACCTCGT
TGTCTGAGGTGGTCCTACAGAACCGGAGAGGATTAGATCTGCTGTTCCTAAAAGAAGGAGGATTATGTGCTGCCCTAAAA
GAAGAATGCTGTTTCTACGCGGACCACACTGGCGTAGTAAGGGATAGCATGGCTAAGCTAAGAGAGAGACTAAACCAGAG
ACAAAAATTGTTCGAATCAGGACAAGGGTGGTTTGAGGGACTGTTTAACAGGTCCCCATGGTTCACGACCCTGATATCCA
CCATTATGGGCCCTCTGATAGTACTTTTATTAATCCTACTCTTCGGACCCTGTATTCTCAACCGCTTGGTCCAGTTTGTA
AAAGACAGAATTTCGGTGGTGCAGGCCCTGGTTCTGACTCAACAGTATCACCAACTCAAATCAATAGATCCAGAAGCAGT
GGAATCACGTGAATAAAAGATTTTATTCAGTTTCCAGAAAGAGGGGGGAATGAAAGACCCCACCATAAGGCTTAGCAAGC
TAGCTGCAGTAACGCCATTTTGCAAGGCATGAAAAAGTACCAGAGCTGAGTTCTCAAAAGTTACAAGGAAGTTCAGTTAA
AGATTAACAGTTAAAAATCAAGGCTGAATAATACTAGGACAAGGGCCAAGAACCGATGGTGCCCACCTGGGCCCCGGCTC
AAGGCCAAGAACCGGTGGTACCCACCTGGGCCCCGGCTCAGGGCCAAGAACAGATGGTACCCAGATAAGGCGGAACCAGC
AACAGTTTCTGAAAAAGTCCCACCTCAGTTTCAGGTTCCCCAAATGACCAGGAAATACCCCCAGCCTTGATTTGAACTAA
CCACTCAGCTCGCTTCTCGCTTCTGTACCCGCGCTTTTTGCTCCCCAGCCCTATAAAAAGGGTAAGAACTCCACACTCGG
CGCGCCAGTCCTCCGACAGACTGAGTCGCCCGGGTACCCGTGTTCCCAATAAAGCCTCATGCTGATTACATCCGAATCGT
GGTCTCGCTGATCCTTGGGAGGGTCTCCTCAGATTGATTGACCACCCACCTCGGAGGTCTTTCA
>Xmv17
TGAAAGACCCCACCATAAGGCTTAGCAAGCTAGCTGCAGTAACGCCATTTTGCAAGGCATGAAAAAGTACCAGAGCTGAG
TTCTCAAAAGTTACAAGGAAGTTCAGTTAAAGATTAACAGTTAAAAATCAAGGCTGAATAATACTAGGACAAGGGCCAAG
AACCGATGGTACCCACCTGGGCCCCGGCTCAGGGCCAAGAACAGATGGTACCCAGATAAGGCGGAACCAGCAACAGTTTC
TGAAAAAGTCCCACCTCAGTTTCAGGTTCCCCAAATGACCAGGAAATACCCCAAGCCTTGATTTGAACTAACCACTCAGC
TCGCTTCTCGCTTCTGTACCCGCGCTTTTTGCTCCCCAGCCCTATAAAAAGGGTAAAAACCCCACACTCGGCGCGCCAGT
CCTCCGATAGACTGAGTCGCCCGGGTACCCGTGTTCCCAATAAAGCCTCTTGCTGATTACATCCGAATCGTGGTCTCGCT
GATCCTTGGGAGGGTCTCCTCAGATTGATTGACTACCCACCTCGGGGGTCTTTCATTTGGAGGTTCCACCGAGATTAGGA
GACCCCTGCCCAGGGACCACCGACCCCCGCCGGGAGGTAAGCTGGCCAGCGGTCGTTTCGTGTCTGTCTCTGTCTCCGTG
CGTGTTTGTGCCGGCATCTAATGTTTGCGCCTGCGTCTGTACTAGTTGGCTAACTAGATCTGTATCTGGCGGTTCCGTGG
AAGAACTGACGAGTTCGTATTCCCGGCCGCAGCCCTGGGAGACGTCCCAGCGGCCTCGGGGGCCCGTTTTGTGGCCCATT
CTGTATCAGTTAACCTGCCCGAGTCGGACTTTTTTGGAGCTCCTCCACTGTCCGAGGGGTACGTGGCTTTGTCGGGGGAC
GAGAGGCAGAGACACTTCCCTCCCCCGTCTGAATTTTTGCTTTCGGTTTTACGCCGAAACCGCGCCGCGCGTCTGATTTG

TTTGTTGTTCTTTTGTTCTTCGTTAGTTTTCTCCTGTCTTTAAGTGTTTTTGAGATCATGGGACAGACCGTAACTACCCC
TCTGAGTCTAACCTTGCAGCACTGGGGAGATGTCCAGCGCATTGCATCCAATCAGTCTGTGGATGTCAGGAAGAGGCGCT
GGGTTACCTTCTGTTCCGCCGAATGGCCAACTTTCAATGTGGGATGGCCTCAGGATGGTACTTTTAATTTAGGTATTATC
TCTCAGGTCAAGTCTAGAGTGTTTTGTCCTGGTCCCCACGGACACCCGGATCAGGTCCCATATATCGTCACCTGGGAGGC
ACTTGCCTATGACCCCCCTCCGTGGGTCAAACCGTTTGTCTCTCCAAAACCCCCTCCTTTACCGACAGCTCCCGTCCTCC
CGCCCGGTCCTTCTGCGCAACCTCCGTCCCGATCTGCCCTTTACCCTGCCCTTACCCCCTCTATAAAGCCCAAACCTCCT
AAGCCCCAGGTTCTCCCTGATAACGGCGGACCTCTCATTGACCTTCTCACAGAGGACCCCCCGCCGTACGGAGCACAACC
TTCCTCCTCTGCCAGAGGGAACGATAAAGAAGAGGCGGCCACCACCTCCGAGGTTTCCCCCCCTTCTCCCATGGTGTCTC
GACTGCGGGGAAGGAGAGACCCTCCCGCAGCGGACTCCACCTCCTCCCAGGCATTCCCACTCCGCATGGGGGGAGATGGC
CAGCTTCAGTACTGGCCGTTTTCCTCCTCGGACTTATATAATTGGAAAAATAATAACCCTTCCTTTTCTGAAGATCCAGG
TAAATTGACGGCCTTGATTGAGTCCGTCCTCATCACCCACCAGCCCACCTGGGACGACTGTCAGCAGTTGTTGGGGACCC
TGCTGACCGGAGAAGAAAAGCAGCGGGTGCTCCTAGAGGCTAGAAAGGCAGTCCGGGGCAATGATGGACGCCCCACTCAG
TTGCCTAATGAAGTCAATGCTGCTTTTCCCCTTGAACGCCCCGATTGGGATTACACCACTACAGAAGGTAGGAACCACCT
AGTCCTCTATCGCCAGTTGCTCTTAGCGGGTCTCCAAAACGCGGGCAGGAGCCCCACCAATTTGGCCAAGGTAAAAGGGA
TAACCCAGGGACCTAATAAGTCTCCCTCAGCCTTTTTAGAGAGACTCAAGAAGGCCTATCGCAGGTACACTCCTTATGAC
CCTGAGGACCCAGGGCAAGAAACCAATGTGTCTATGTCATTCATCTGGCAGTCTGCCCCGGATATCGGGCGAAAGTTAGA
GCGGTTAAAAGATTTAAAGAGCAAGACCTTAGGAGACTTAGTGAGGGAAGCTGAAAGGATCTTTAATAAGCGAGAAACCC
CGGAAGAAAGAGAGGAACGTATCAGGAGAGAAACAGAGGAAAAAGAAGAACGCCGTAGGGCAGAGGATGAGCAGAGAGAG
AAAGAAAGGGACCGCAGAAGACATAGAGAGATGAGCAAGCTCTTGGCCACTGTAGTTAATGGTCAGAGACAGGATAGACA
GGGGGGAGAGCGGAGGAGGCCCCAACTTGATAAGGACCAATGCGCCTACTGCAAAGAAAAGGGACACTGGGCTAAGGACT
GCCCAAAGAAGCCACGAGGGCCCCGAGGACCGAGGCCCCAGACCTCCCTCCTGACCTTAGGTGACTAGGGAGGTCAGGGT
CAGGAGCCCCCCATGATTGCCTCGAGATCTTGGCTGAAACGCATGGAACCAGACCGGATCTCACCGACCAGCCCATCCCA
GACGCCGACCACACCCGGTATACCGATGGGAGCAGCTTTTTGCAAGAAGGACAGCGAAAGGCTGGGGCAGCAGTGACGAC
TGAAACCGAGGTAATCTGGGCGAGGGCCCTGCCAGCTGGAACGTCAGCCCAGCGAGCCGAACTGATCGCACTCACCCAAG
CCCTGAAAATGGCAGAAGGTAAGAAGCTAAATGTTTACACTGATAGCCGCTATGCCTTCGCTACGGCCCATGTTCATGGG
AAAATATATAGGAGACGGGGGTTGCTGACCTCAGAAGGCAAGGAAATCAAGAACAAAAGCGAGATCCTAGCCTTGCTGAA
AGCCCTCTTTTTGCCAAAGAGGCTCAGTATTATCCATTGCCCGGGACACCAAAAAGGGAACAGTGCTGAAGCCAGGGGCA
ACCGAATGGCGGATCAAGCAGCCAGGGAGGCAGCCATGAGAGAGATCACAGAAACTTCCACGCTCTTAATAGAGGATTCG
TCCCCGTATACTCCCAGTTACTTCCACTACACAGAGACTGACAGAAAAGACCTTCTGAGACTGGGAGCCGTATACGATGA
GGACAAAAGGTATTGGGTCCTGCAGGGAAAACCTGTAATGCCTGACCAATTCACCTTTGAGCTCTTAGACTCCCTACACC
GGCTCACCCACCTCAGCTATCAGAGGATGAAGGCACTCCTTGACCGGGAAGAGAGCCCCTACTACATGTTAAATCGGGAT
AGAACTCTCCAGCACGTAACAGAATCCTGTACAGCTTGTGCTCAAGTAAATGCTAGTAAAGCCAAAATCGGAGCAAGGGT
GCGAGCGCGGGGACACCGACCCGGCACCCATTGAGAAATCGATTTTACCGAAGTTAAGCCAGGGCTGTATGGACATAAGT
ATCTCCTGGTGTTTGTGGACACATTCTCTGGCTGGGTAGAAGCATTTCCAACCAAGCGTGAAACTGCCAGGGTTGTGACC
AAGAAGCTGCTTGAAGAAATTTTTCCAAGATTCGGGATGCCCCAGGTATTGGGGACAGATAATGGGCCTGCCTTCATCTC
CCAGGTAAGTCAGTCGGTGGCCAAGTTGCTGGGGATTGATTGGAAACTGCATTGTGCTTACAGACCCCAGAGTTCAGGTC
AGGTAAAAAAAATGAACAGGACAATCAAGGAGACTTTAACCAAATTAACGCTTGCAGCTGGCACTAGAGACTGGGTACTC
CTACTCCCCCTGGCCCTCTACCGAGCCCGGAATACTCCGGGCCCCCATGGACTTACTCCGTATGAAATTCTGTATGGGGC
ACCCCCGCCCCTTGTCAATTTTCATGATCCTGAAATGTCAAAGTTAACTAATAGTCCCTCTCTCCAAGCTCACTTACAGG
CCCTCCAAGCAGTACAACGAGAGGTCTGGAAGCCACTGGCCGCTGCTTATCAGGACCAGCTGGATCAGCCAGTGATACCA
CACCCCTTCCGCGTCGGTGACGCCGTGTGGGTACGCCGGCACCAGACTAAGAACTTAGAACCCCGCTGAAAAGGACCCTA
CACCGTCCTGCTGACCACCCCCACCGCTCTCAAAGTAGACGGCATCTCTGCGTGGATACACGCCGCTCACGTAAAGGCGG
CGACAACTCCTCCGGCCGGAACAGCATGGAAGGTCCAGCGTTCTCAAAATCCCTTAAAGATAAGATTAACCCGTGGGGCC
CCCTGATAGTTATGGGGATCTTAGTGAGGGCAGGAGCCTCGGTACAACGTGACAGCCCTCACCAGGTCTTCAATGTTACT
TGGAGAGTTACCAACCTAATGACAGGACAAACAGCTAACGCCACCTCCCTCCTGGGGACGATGACAGACACCTTCCCTAA
ACTATATTTTGACCTGTGTGATTTAGTAGGAAACTACTGGGATTGGGATGACCCAGATGGAGATGGTTGCCGCTCTCCCG
GGGGAAGAAAAAGGACAAGACTGTATGACTTCTATGTTTGCCCCGGTCATACTGTACCAATAGGGTGTGGAGGGCCGGGA
GAGGGCTACTGTGGCAAATGGGGATGTGAGACCACTGGACAGGCATACTGGAAGCCATCATCATCATGGGACCTAATTTC
CCTTAAGCGAGGAAACACTCCTAAGGATCAGGGCCCCTGTTATGATTCCTCGGTCTCCAGTGGCGTCCAGGGTGCCACAC
CGGGGGGTCGATGCAACCCCCTAGTCTTAGAATTCACTGACGCGGGTAAAAAGGCCAGCTGGGATGCCCCCAAAGTTTGG
GGACTAAGACTCTACCGATCCACAGGGGCCGACCCGGTGACCCGGTTCTCTTTGACCCGCCAGGTCCTCAATGTAGGACC
CCGCGTCCCCATTGGGCCTAATCCCGTGATCACTGACCAGCTACCCCCATCCCAACCCGTGCAGATCATGCTCCCCAGGC
CTCCTCATCCTCCTCCTTCAGGCACGGTCTCTATGGTACCTGGGGCTCCCCCGCCTTCTCAACAGCCTGGGACGGGAGAC
AGGCTGCTAAATCTGGTAGACGGAGCCTACCAAGCACTCAACCTCACCAGTCCTGACAAAACCCAAGAGTGCTGGTTGTG
TCTGGTATCGGGACCCCCCTACTACGAAGGGGTTGCCGTCCTAGGTACCTACTCCAACCATACCTCTGCCCCAGCTAACT
GCTCCGTGGCTTCCCAACACAAGCTGACCCTGTCCGAAGTGACCGGACAGGGACTCTGCGTAGGAGCAGTTCCCAAAACC
CATCAGGCCCTGTGTAATACCACCCAGAAGACGAGCGACGGGTCCTACTATCTGGCTGCTCCCGCCGGGACCATCTGGGC

TTGCAACACCGGGCTCACTCCCTGCTTATCTACTACTGTACTCAACCTCACCACCGATTACTGTGTCCTGGTTGAGCTCT
GGCCAAAGGTGACCTACCACTCCCCTGGTTATGTTTATGGCCAGTTTGAGAGAAAAACCAAATATATAAGAGAGCCGGTG
TCATTAACTCTGGCCCTGCTGTTGGGAGGACTTACTATGGGCGGCATAGCTGCAGGAGTAGGAACAGGGACTACAGCCCT
AGTGGCCACCAAACAATTCGAGCAGCTCCAGGCAGCCATACATACAGACCTTGGGGCCTTAGAAAAATCAGTCAGTGCCC
TAGAAAAGTCTCTGACCTCGTTGTCTGAGGTGGTCCTACAGAACCGGAGAGGATTAGATCTGCTGTTCCTAAAAGAAGGA
GGATTATGTGCTGCCCTAAAAGAAGAATGCTGTTTCTACGCGGACCACACTGGCGTAGTAAGAGATAGCATGGCAAAGCT
AAGAGAAAGGTTAAACCAGAGACAAAAATTGTTCGAATCAGGACAAGGGTGGTTTGAGGGACTGTTTAACAGGTCCCCAT
GGTTCACGACCTTAATATCCACCATTATGGGCCCCTTGATAATACTTTTATTAATCCTACTCCTCGGACCCTGTATTCTC
AACCGCTTGGTCCAGTTTGTAAAAGACAGAATTTCGGTGGTGCAGGCCCTGGTTCTGACCCAACAGTATCACCAACTCAA
ATCAATAGATCCAGAAGAAGTGGAATCACGTGAATAAAAGATTTTATTCAGTTTCCAGAAAGAGGGGGGAATGAAAGACC
CCACCATAAGGCTTAGCAAGCTAGCTGCAGTAACGCCATTTTGCAAGGCATGAAAAAGTACCAGAGCTGAGTTCTCAAAA
GTTACAAGGAAGTTCAGTTAAAGATTAACAGTTAAAAATCAAGGCTGAATAATACTAGGACAAGGGCCAAGAACCGATGG
TACCCACCTGGGCCCCGGCTCAGGGCCAAGAACAGATGGTACCCAGATAAGGCGGAACCAGCAACAGTTTCTGAAAAAGT
CCCACCTCAGTTTCAGGTTCCCCAAATGACCAGGAAATACCCCAAGCCTTGATTTGAACTAACCACTCAGCTCGCTTCTC
GCTTCTGTACCCGCGCTTTTTGCTCCCCAGCCCTATAAAAAGGGTAAAAACCCCACACTCGGCGCGCCAGTCCTCCGATA
GACTGAGTCGCCCGGGTACCCGTGTTCCCAATAAAGCCTCTTGCTGATTACATCCGAATCGTGGTCTCGCTGATCCTTGG
GAGGGTCTCCTCAGATTGATTGACTACCCACCTCGGGGGTCTTTCA
>Xmv18
TGAAAGACCCCACCATAAGGCTTAGCAAGCTAGCTGCAGTAACGCCATTTTGCAAGGCATGAAAAAGTACCAGAGCTGAG
TTCTCAAAAGTTACAAGGAAGTTCAGTTAAAGATTAACAGTTAAAAATCAAGGCTGAATAATACTAGGACAAGGGCCAAG
AACCGATGGTACCCACCGGGGCCCCGGCTCAGGGCCAAGAACCGATGGTACCCACCTGGGCCCCGGCTCAGGGCCAAGAA
CAGATGATACCCAGATAAGGCGGAACCAGCAACAGTTTCTGAAAAAGTCCCACCTCAGTTTCAGGTTCCCCAAATGACCA
GGAAATACCCCAAGCCTTGATTTGAACTAACCACTCAGCTCGCTTCTCGCTTCTGTACCCGCGCTTTTTGCTCCCCAGCC
CTATAAAAAGGGTAAAAACCCCACACTCGGCGCGCCAGTCCTCCGATAGACTGAGTCGCCCGGGTACCCGTGTTCCCAAT
AAAGCCTCTTGCTGATTACATCCGAATCGTGGTCTCGCTGATCCTTGGGAGGGTCTCCTCAGATTGATTGACTACCCACC
TCGGGGGTCTTTCATTTGGAGGTTCCACCGAGATTAGGAGACCCCTGCCCAGGGACCACCGACCCCCGCCGGGAGGTAAG
CTGGCCAGCGGTCGTTTCGTGTCTGTCTCTGTCTCCGTGCGTGTTTGTGCCGGCATCTAATGTTTGCGCCTGCGTCTGTA
CTAGTTGGCTAACTAGATCTGTATCTGGCGGTTCCGTGGAAGAACTGACGAGTTCGTATTCCCGGCCGCAGCCCTGGGAG
ACGTCCCAGCGGCCTCGGGGGCCCGTTTTGTGGCCCATTCTGTATCAGTTAACCTGCCCGAGTCGGACTTTTTGGAGCTC
CTCCACTGTCCGAGGGGTACGTGGCTTTGTCGGGGGACGAGAGGCAGAGACACTTCCCTCCCCCGTCTGAATTTTTGCTT
TCGGTTTTACGCCGAAACCGCGCCGCGCGTCTGATTTGTTTGTTGTTCTTTTGTTCTTCGTTAGTTTTCTCCTGTCTTTA
AGTGTTTTTGAGATCATGGGACAGACCGTAACTACCCCTCTGAGTCTAACCTTGCAGCACTGGGGAGATGTCCAGCGCAT
TGCATCCAATCAGTCTGTGGATGTCAGGAAGAGGCGCTGGGTTACCTTCTGTTCCGCCGAATGGCCAACTTTCAATGTGG
GATGGCCTCAGGATGGTACTTTTAATTTAGGTATTATCTCTCAGGTCAAGTCTAGAGTGTTTTGTCCTGGTCCCCACGGA
CACCCGGATCAGGTCCCATATATCGTCACCTGGGAGGCACTTGCCTATGACCCCCCTCCGTGGGTCAGACCGTTTGTCTC
TCCAAAACCCCCTCCTTTACCGACAGCTCCCGTCCTCCCGCCCGGTCCTTCTGCGCAACCTCCGTCCCGATCTGCCCTTT
ACCCTGCCCTTACCCCCTCTATAAAGCCCAAACCTCCTAAGCCCCAGGTTCTCCCTGATAACGGCGGACCTCTCATTGAC
CTTCTCACAGAGGACCCCCCGCCATACGGAGCACAACCTTCCTCCTCTGCCAGAGGGAACGATGAAGAAGAGGCGGCCAC
CACCCCCGAGGTTTCCCCCCCTTCTCCCATGGTGTCTCGACTGCGGGGAAGGAGAGACCCTCCCGCAGCGGACTCCACCT
CCTCCCAGGCATTCCCACTCCGCATGGGGGGAGATGGCCAGCTTCAGTACTGGCCGTTTTCCTCCTCTGACTTATATAAT
TGGAAAAATAATAACCCTTCCTTTTCTGAAGACCCAGGTAAATTGACGGCCTTGATTGAGTCCGTCCTCATCACCCACCA
GCCCACCTGGGACGACTGTCAGCAGTTGTTGGGGACCCTGCTGACCGGAGAAGAAAAGCAGCGGGTGCTCCTAGAGGCTA
GAAAGGCAGTCCGGGGCAATGATGGACGCCCCACTCAGTTGCCTAATGAAGTCAATGCTGCTTTTCCCCTTGAACGCCCC
GATTGGGATTACACCACTACAGAAGGTAGGAACCACCTAGTCCTCTATCGCCAGTTGCTCTTAGCGGGTCTCCAAAACGC
GGGCAGGAGCCCCACCAATTTGGCCAAGGTAAAAGGGATAACCCAGGGACCTAATGAGTCTCCCTCAGCCTTTTTAGAGA
GACTCAAGGAGGCCTATCGCAGGTACACTCCTTATGACCCTGAGGACCCAGGGCAAGAAACCAATGTGTCTATGTCATTC
ATCTGGCAGTCTGCCCCGGATATCGGGCGAAAGTTAGAGCGGTTAGAAGACTTAAAGAGCAAGACCTTAGGAGACTTAGT
GAGGGAAGCTGAAAGGATCTTTAATAAGCGAGAAACCCCGGAAGAAAGAGAGGAACGTATCAGGAGAGAAACAGAGGAAA
AAGAAGAACGCCGTAGGGCAGAGGATGAGCAGAGAGAGAAAGAAAGGGACCGCAGAAGACATAGAGAGATGAGCAAGCTC
TTGGCCACTGTAGTTATTGGTCAGAGACAGGATAGACAGGGGGGAGAGCGGAGGAGGCCCCAACTTGATAAGGACCAATG
CGCCTACTGCAAAGAAAAGGGACACTGGGCTAAGGACTGCCCAAAGAAGCCACGAGGGCCCCGAGGACCGAGGCCCCAGA
CCTCCCTCCTGACCTTAGGTGACTAGGGAGGTCAGGGTCAGGAGCCCCCCCCTGAACCCAGGATAACCCTCAAAGTCGGG
GGGCAACCCGTCACCTTCCTGGTAGATACTGGGGCCCAACACTCCGTGCTGACCCAAAATCCTGGACCCCTAAGTGACCA
GTCTGCCTGGGTCCAAGGGGCTACTGGAGGAAAGCGGTATCGCTGGACCACGGATCGCAAAGTACATCTAGCTACCGGTA
AGGTCACCCACTCTTTCCTCCATGTACCAGACTGCCCCTATCCTCTGCTAGGAAGAGACTTGCTGACTAAACTAAAAGCC
CAGATCCACTTCGAGGGATCAGGAGCTCAGGTTGTGGGACCAATGGGACGGCCCCTGCAAGTGCTGACCCTAAACATAGA
AGATGAGTATCGGCTACATGAGACCTCAAAAGAGCCGGATGTTTCTCTAGGGTCCACATGGCTTTCTGATTTTCCCCAGG

CCTGGGCAGAAACCGGGGGCATGGGACTGGCAGTTCGCCAAGCGCCTCTGATTATACCTCTAAAGGCAACCTCTACCCCC
GTGTCCATAAAACAATACCCCATGTCACAGGAAGCCAGACTGGGGATCAAGCCCCACATACAGAGGCTGTTGGACCAGGG
AATACTGGTACCCTGCCAGTCCCCCTGGAACACGCCCCTGCTACCCGTTAAGAAACCAGGGACTAATGATTACAGGCCTG
TCCAGGATCTGAGAGAAGTCAACAAGCGGGTGGAAGACATCCACCCCACCGTGCCCAACCCTTACAACCTCTTGAGCGGG
CTCCCACCGTCCCACCAGTGGTACACTGTGCTTGATTTAAAGGATGCCTTTTTCTGCCTGAGACTCCACCCCACCAGTCA
GCCTCTCTTCGCCTTTGAGTGGAGAGATCCAGGGATGGGAATCTCAGGACAATTGACCTGGACCAGACTCCCACAGGGTT
TCAAAAACAGTCCCACCCTGTTTGATGAGGCACTGCACAGAGACCTAGCAGACTTCCGGATCCAGCACCCAGACTTGATC
CTGCTACAGTACGTGGATGACATACTACTGGCCGCCACTTCTGAGCTCGACTGCCAACAAGGTACTCGGGCCCTGTTACA
AACCCTAGGAAACCTCGGGTATCGGGCCTCGGCCAAGAAAGCCCAACTTTGCCAGAAACAGGTCAAGTATCTGGGGTATC
TTCTAAAAGAGGGTCAGAGATGGCTGACTGAGGCCAGAAAAGAGACTGTGATGGGGCAGCCTACTCCGAAGACCCCTCGA
CAACTAAGGGAGTTCCTAGGGACGGCAGGCTTCTGTCGCCTCTGGATCCCTGGGTTTGCGGAAATGGCAGCCCCCTTGTA
TCCTCTTACCAAAACGGGGACTCTGTTTAATTGGGGCCCAGACCAGCAAAAGGCCTATCAAGAAATCAAACAGGCCCTTC
TAACTGCCCCCGCCCTGGGATTGCCAGATTTGACTAAGCCCTTTGAACTCTTTGTCGACGAGAAGCAGGGCTACGCCAAA
GGCGTCCTAACGCAAAAACTGGGACCTTGGCGTCGGCCTGTGGCCTACCTGTCCAAAAAGCTAGACCCAGTGGCAGCTGG
GTGGCCCCCTTGCCTACGGATGGTAGCAGCCATTGCCGTTCTGACAAAAGATGCAGGCAAGCTAACTATGGGACAGCCGC
TAGTCATCCTGGCCCCCCATGCAGTAGAGGCACTGGTCAAGCAACCCCCTGACCGCTGGCTATCCAACGCCCGCATGACC
CACTACCAGGCAATGCTCCTAGACACTGACCGAGTTCAGTTCGGACCAGTGGTGGCCCTCAATCCTGCCACCTTGCTCCC
TCTACCGGAAAAAGGAGCCCCCCATGATTGCCTCGAGATCTTGGCTGAAACGCATGGAACCAGACCGGATCTCACCGACC
AGCCCATCCCAGACGCCGACCACACCTGGTATACCGATGGGAGCAGCTTTTTGCAAGAAGGACAGCGAAAGGCTGGGGCA
GCAGTGACGACTGAAACCGAGGTAATCTGGGCGAGGGCCCTGCCAGCTGGAACGTCAGCCCAGCGAGCCGAACTGATCGC
ACTCACCCAAGCCCTGAAAATGGCAGAAGGTAAGAAGCTAAATGTTTACACTGATAGCCGCTATGCCTTCGCTACGGCCC
ATGTTCATGGGGAAATATATAGGAGACGGGGGTTGCTGACCTCAGAAGGCAAGGAAATCAAGAACAAAAGCGAGATCCTA
GCCTTGCTGAAAGCCCTCTTTTTGCCAAAGAGGCTCAGTATTATCCATTGCCCGGGACACCAAAAAGGGAACAGTGCTGA
AGCCAGGGGCAACCGAATGGCGGATCAAGCAGCCAGGGAGGCAGCCATGAGAGAGATCACAGAAACTTCCACGCTCTTAA
TAGAGGATTCGTCTCCGTATACTCCCAGTTACTTCCACTACACAGAGACTGACAGAAAAGACCTTCTGAGACTGGGAGCC
GTATACGATGAGGACAAAAGGTATTGGGTCCTGCAGGGAAAACCTGTAATGCCTGACCAATTCACCTTTGAGCTCTTAGA
CTCCCTACACCGGCTCACCCACCTCAGCTATCAGAAGATGAAGGCACTCCTTGACCGGGAAGAGAGCCCCTACTACATGT
TAAATCGGGATAGAACTCTCCAGCACGTAACAGAATCCTGTACAGCTTGTGCTCAAGTAAATGCTAGTAAAGCCAAAATC
GGAGCAAGGGTGCGAGCGCGGGGACACCGACCCGGCACCCATTGGGAAATCGATTTTACCGAAGTTAAGCCAGGGCTGTA
TGGACATAAGTATCTCCTGGTGTTTGTGGACACATTCTCTGGCTGGGTAGAAGCATTTCCAACCAAGCGTGAAACTGCCA
GGGTTGTGACCAAGAAGCTGCTTGAAGAAATTTTTCCAAGATTCGGGATGCCCCAGGTATTGGGGACAGATAATGGGCCT
GCCTTCATCTCCCAGGTAAGTCAGTCGGTGGCCAAGTTGCTGGGGATTGATTGGAAACTGCATTGTGCTTACAGACCCCA
GAGTTCAGGTCAGGTAGAAAGAATGAACAGGACAATCAAGGAGACTTTAACCAAATTAACGCTTGCAGCTGGCACTAGAG
ACTGGGTACTCCTACTCCCCCTGGCCCTCTACCGAGCCCGGAATACTCCGGGCCCCCATGGACTTACTCCGTATGAAATT
CTGTATGGGGCACCCCCGCCCCTTGTCAATTTTCATGATCCTGAAATGTCAAAGTTAACTAATAGTCCCTCTCTCCAAGC
TCACTTACAGGCCCTCCAAGCAGTACAACGAGAGGTCTGGAAGCCACTGGCCGCTGCTTATCAGGACCAGCTGGATCAGC
CAGTGATACCACACCCCTTCCGCGTCGGTGACGCCGTGTGGGTACGCCGGCACCAGACTAAGAACTTAGAACCCCGCTGG
AAAGGACCCTACACCGTCCTGCTGACCACCCCCACCGCTCTCAAAGTAGACGGCATCTCTGCGTGGATACACGCCGCTCA
CGTAAAGGCGGCGACAACTCCTCCGGCCGGAACAGCATGGAAGGTCCAGCGTTCTCAAAATCCCTTAAAGATAAGATTAA
CCCGTGGGGCCCCCTGATAGTTATGGGGATCTTAGTGAGGGCAGGAGCCTCGGTACAACGTGACAGCCCTCACCAGGTCT
TCAATGTTACTTGGAGAGTTACCAACCTAATGACAGGACAAACAGCTAACGCCACCTCCCTCCTGGGGACGATGACAGAC
ACCTTCCCTAAACTATATTTTGATCTGTGTGATTTAGTAGGAGACTACTGGGATGACCCAGATGGAGATGGTTGCCGCTC
TCCCGGGGGAAGAAAAAGGACAAGACTGTATGACTTCTATGTTTGCCCCGGTCATACTGTACCAATAGGGTGTGGAGGGC
CGGGAGAGGGCTACTGTGGCAAATGGGGATGTGAGACCACTGGACAGGCATACTGGAAGCCATCATCATCATGGGACCTA
ATTTCCCTTAAGCGAGGAAACACTCCTAAGGATCAGGGCCCCTGTTATGATTCCTCGGTCTCCAGTGGCGTCCAGGGTGC
CACACCGGGGGGTCGATGCAACCCCCTAGTCTTAGAATTCACTGACGCGGGTAAAAAGGCCAGCTGGGATGCCCCCAAAG
TTTGGGGACTAAGACTCTACCGATCCACAGGGGCCGACCCGGTGACCCGGTTCTCTTTGACCCGCCAGGTCCTCAATGTA
GGACCCCGCGTCCCCATTGGGCCTAATCCCGTGATCACTGACCAGCTACCCCCATCCCAACCCGTGCAGATCATGCTCCC
CAGGCCTCCTCATCCTCCTTCAGGCACGGTCTCTATGGTACCTGGGGCTCCCCCGCCTTCTCAACAGCCTGGGACGGGAG
ACAGGCTGCTAAATCTAGTAGACGGAGCCTACCAAGCACTCAACCTCACCAGTCCTGACAAAACCCAAGAGTGCTGGTTG
TGTCTGGTATCGGGACCCCCCTACTACGAAGGGGTTGCCGTCCTAGGTACCTACTCCAACCATACCTCTGCCCCAGCTAA
CTGCTCCGTGGCCTCCCAACACAAGCTGACCCTGTCCGAAGTGACCGGACAGGGACTCTGCGTAGGAGCAGTTCCCAAAA
CCCATCAGGCCCTGTGTAATACCACCCAGAAGACGAGCGACGGGTCCTACTATCTGGCTGCTCCCGCCGGGACCATCTGG
GCTTGCAACACCGGGCTCACTCCCTGCCTATCTACTACTGTACTCAACCTCACCACCGATTACTGTGTCCTGGTTGAGCT
CTGGCCAAAGGTGACCTACCACTCCCCTGGTTATGTTTATGGCCAGTTTGAGAGAAAAACCAAATATATAAGAGAGCCGG
TGTCATTAACTCTGGCCCTGCTGTTGGGAGGACTTACTATGGGCGGCATAGCTGCAGGAGTAGGAACAGGGACTACAGCC
CTAGTGGCCACCAAACAATTCGAGCAGCTCCAGGCAGCCATACATACAGACCTTGGGGCCTTAGAAAAATCAGTCAGTGC

CCTAGAAAAGTCTCTGACCTCGTTGTCTGAGGTGGTCCTACAGAACCGGAGAGGATTAGATCTGCTGTTCCTAAAAGAAG
GAGGATTATGTGCTGCCCTAAAAGAAGAATGCTGTTTCTACGCGGACCACACTGGCGTAGTGAGAGATAGCATGGCAAAG
CTAAGAGAAAGGTTAAACCAGAGACAAAAATTGTTCGAATCAGGACAAGGGTGGTTTGAGGGACTGTTTAACAGGTCCCC
ATGGTTCACGACCTTGATATCCACCATTATGGGCCCCTTGATAATACTTTTATTAATCCTACTCCTCGGACCCTGTATTC
TCAACCGCTTGGTCCAGTTTGTAAAAGACAGAATTTCGGTGGTGCAGGCCCTGGTTCTGACCCAACAGTATCACCAACTC
AAATCAATAGATCCAGAAGAAGTGGAATCACGTGAATAAAAGATTTTATTCAGTTTCCAGAAAGAGGGGGGAATGAAAGA
CCCCACCATAAGGCTTAGCAAGCTAGCTGCAGTAACGCCATTTTGCAAGGCATGAAAAAGTACCAGAGCTGAGTTCTCAA
AAGTTACAAGGAAGTTCAGTTAAAGATTAACAGTTAAAAATCAAGGCTGAATAATACTAGGACAAGGGCCAAGAACCGAT
GGTACCCACCGGGGCCCCGGCTCAGGGCCAAGAACCGATGGTACCCACCTGGGCCCCGGCTCAGGGCCAAGAACAGATGA
TACCCAGATAAGGCGGAACCAGCAACAGTTTCTGAAAAAGTCCCACCTCAGTTTCAGGTTCCCCAAATGACCAGGAAATA
CCCCAAGCCTTGATTTGAACTAACCACTCAGCTCGCTTCTCGCTTCTGTACCCGCGCTTTTTGCTCCCCAGCCCTATAAA
AAGGGTAAAAACCCCACACTCGGCGCGCCAGTCCTCCGATAGACTGAGTCGCCCGGGTACCCGTGTTCCCAATAAAGCCT
CTTGCTGATTACATCCGAATCGTGGTCTCGCTGATCCTTGGGAGGGTCTCCTCAGATTGATTGACTACCCACCTCGGGGG
TCTTTCA
>Xmv19
TTGAAAGACCCCACCATAAGGCTTAGCAAGCTAGCTGCAGTAACGCCATTTTGCAAGGCATGAAAAAGTACCAGAGCTGA
GTTCTCAAAAGTTACAAGGAAGTTCAGTTAAAGATTAACAGTTAAAAATCAAGGCTGAATAATACTAGGACAAGGGCCAA
GAACCGATGGTGCCCACCTGGGCCCCGGCTCAAGGCCAAGAACCGATGGTACCCACCTGGGCCCCGGCTCAGGGCCAAGA
ACAGATGGTACCCAGATAAGGCGGAACCAGCAACAGTTTCTGAAAAAGTCCCACCTCAGTTTCAGGTTCCCCAAATGACC
AGGAAATACCCCAAGCCTTGATTTGAACTAACCACTCAGCTCGCTTCTCGCTTCTGTACCCGCGCTTTTTGCTCCCCAGC
CCTATAAAAAGGGTAAAAACCCCACACTCGGCGCGCCAGTCCTCCGATAGACTGAGTCGCCCGGGTACCCGTGTTCCCAA
TAAAGCCTCTTGCTGATTACATCCGAATCGTGGTCTCGCTGATCCTTGGGAGGGTCTCCTCAGATTGATTGACTACCCAC
CTCGGGGGTCTTTCATTTGGAGGTTCCACCGAGATTAGGAGACCCCTGCCCAGGGACCACCGACCCCCGCCGTACGGAGC
ACAACCTTCCTCCTCTGCCAGAGGGAACGATGAAGAAGAGGCGGCCACCACCTCCGAGGTTTCCCCCCCTTCTCCCATGG
TGTCTCGACTGCGGGGAAGGAGAGACCCTCCCGCAGCGGACTCCACCTCCTCCCAGGCATTCCCACTCCGCATGGGGGGA
GATGGCCAGCTTCAGTATTGGCCGTTTTCCTCCTCAGACTTATATAATTGGAAAAATAATAACCCTTCCTTTTCTGAAGA
CCCAGGTAAATTGACGGCCTTGATTGAGTCCGTCCTCATCACCCACCAGCCCACCTGGGACGACTGTCAGCAGTTGTTGG
GGACCCTGCTGACCGGAGAAGAAAAGCAGCGGGTGCTCCTAGAGGCTAGAAAGGCAGTCCGGGGCAATGATGGACGCCCC
ACTCAGTTGCCTAATGAAGTCAATGCTGCCTTTCCCCTTGAACGCCCCGATTGGGATTACACCACTACAGAAGGTAGGAA
CCACCTAGTCCTCTATCGCCAGTTGCTCTTAGCGGGTCTCCAAAACGCGGGCAGGAGCCCCACCAATTTGGCCAAGGTAA
AAGGGATAACCCAGGGACCTAATGAGTCTCCCTCAGCCTTTTTAGAGAGACTCAAGGAGGCCTATCGCAGGTACACTCCT
TATGACCCTGAGGACCCAGGGCAAGAAACCAATGTGTCTATGTCATTCATCTGGCAGTCTGCCCCGGATATCGGGCGAAA
GTTAGAGCGGTTAGAAGATTTAAAGAGCAAGACCTTAGGAGACTTAGTGAGGGAAGCTGAAAGGATCTTTAATAAGCGAG
AAACCCCGGAAGAGAGAGAGGAACGTATCAGGAGAGAAACAGAGGAAAAAGAAGAACGCCGTAGGGCAGAGGATGAGCAG
AGAGAGAAAGAAAGGGACCGCAGAAGACATAGAGAGATGAGCAAGCTCTTGGCCACTGTAGTTATTGGTCAGAGGCAGGA
TAGACAGGGGGGAGAGCGGAGGAGGCCCCAACTTGATAAGGACCAATGCGCCTACTGCAAAGAAAAGGGACACTGGGCTA
AGGACTGCCCAAAGAAGCCACGAGGGCCCCGAGGACCGAGGCCCCAGACCTCCCTCCTGACCTTAGGTGACTAGGGAGGT
CAGGGTCAGGAGCCCCCCCCTGAACCCAGGATAACCCTCAAAGTCGGGGGGCAACCCGTCACCTTCCTGGTAGATACTGG
GGCCCAACACTCCGTGCTGACCCAAAATCCTGGACCCCTAAGTGACAAGTCTGCCTGGGTCCAAGGGGCTACTGGAGGAA
AGCGGTACCGCTGGACCACGGATCGCAAAGTACATCTAGCTACCGGTAAGGTCACCCACTCTTTCCTCCATGTACCAGAC
TGCCCCTATCCTCTGCTAGGAAGAGACTTGCTGACTAAACTAAAAGCCCAGATCCACTTCGAGGGATCAGGAGCTCAGGT
TGTGGGACCAATGGGGCAGCCCCTGCACGTGCTGACCCTAAACATAGAAGATGAGTATCGGCTACATGAGACCTCAAAAG
AGCCGGATGTTTCTCTAGGGTCAACATGGCTTTCTGATTTTCCCCAGGCCTGGGCAGAAACCGGGGGCATGGGACTGGCA
GTTCGCCAAGCGCCTCTGATTATACCTCTAAAGGCAACCTCTACCCCCGTGTCCATAAAACAATACCCCATGTCACAGGA
AGCCAGACTGGGGATCAAGCCCCACATACAGAGGCTGTTGGACCAGGGAATACTGATACCCTGCCAGTCCCCCTGGAACA
CGCCCCTGCTACCCGTTAAGAAACCAGGGACTAATGATTACAGGCCTGTCCAGGATCTGAGAGAAGTCAACAAGCGGGTG
GAAGACATCCACCCCACCGTGCCCAACCCTTACAACCTCTTGAGCGGGCTCCCACCGTCCCACCAGTGGTACACTGTGCT
TGATTTAAAGGATGCCTTTTTCTGCCTGAGACTCCACCCCACCAGTCAGCCTCTCTTCGCCTTTGAGTGGAGAGATCCAG
AGATGGGAATCTCAGGACAATTGACCTGGACCAGACTCCCACAGGGTTTCAAAAACAGTCCCACCCTGTTTGATGAGGCA
CTACACAGAGACCTAGCAGACTTCCGGATCCAGCACCCAGACTTGATCCTGCTACAGTACGTGGATGACATACTACTGGC
CGCCACTTCTGAGCTCAACTGCCAACAAGGTACTCGGGCCCTGTTACAAACCCTAGGAAACCTCGGGTATCGGGCCTCGG
CCAAGAAAGCCCAACTTTGCCAGAAACAGGTCAAGTATCTGGGGTATCTTCTGAAAGAGGGTCAGAGATGGCTGACTGAG
GCCAGAAAAGAGACTGTGATGGGGCAGCCTACTCCGAAGACCCCTCGACAACTAAGGGAGTTCCTAGGGACGGCAGGCTT
CTGTCGCCTCTGGATCCCTGGGTTTGCGGAAATGGCAGCCCCCTTGTATCCTCTTACCAAAACGGGGACTCTGTTTAATT
GGGGCCCAGACCAGCAAAAGGCCTATCAAGAAATCAAACAGGCCCTTCTAACTGCCCCCGCCCTGGGATTGCCAGATTTG
ACTAAGCCCTTTGAACTCTTTGTCGACGAGAAGCAGGGCTACGCCAAAGGCGTCCTAACGCAAAAACTGGGACCTTGGCG
TCGGCCTGTGGCCTACCTGTCCAAAAAGCTAGACCCAGTGGCAGCTGGGTGGCCCCCTTGCCTACGGATGGTAGCAGCCA

TTGCCGTTCTGACAAAAGATGCAGGCAAGCTAACTATGGGACAGCCGCTAGTCATCCTGGCCCCCCATGCAGTAGAGGCA
CTGGTCAAGCAACCCCCTGACCGCTGGCTATCCAACGCCCGCATGACCCACTACCAGGCAATGCTCCTAGACACTGACCG
AGTTCAGTTCGGACCAGTGGTGGCCCTCAATCCTGCCACCTTGCTCCCTCTACCGGAAAAAGGAGCCCCCCATGATTGCC
TCGAGATCTTGGCTGAAACGCATGGAACCAGACCGGATCTCACCGACCAGCCCATCCCAGACGCCGACCACACCTGGTAT
ACCGATGGGAGCAGCTTTTTGCAAGAAGGACAGCGAAAGGCTGGGGCAGCAGTGACGACTGAAACCGAGGTAATCTGGGC
GAGGGCCCTGCCAGCTGGAACGTCAGCCCAGCGAGCCGAACTGATCGCACTCACCCAAGCCCTGAAAATGGCAGAAGGTA
AGAAGCTAAATGTTTACACTGATAGCCGCTATGCCTTCGCTACGGCCCATGTTCATGGGGAAATATATAGGAGACGGGGG
TTGCTGACCTCAGAAGGCAAGGAAATCAAGAACAAAAGCGAGATCCTAGCCTTGCTGAAAGCCCTCTTTTTGCCAAAGAG
GCTCAGTATTATCCATTGCCCGGGACACCAAAAAGGGAACAGTGCTGAAGCCAGGGGCAACCGAATGGCGGATCAAGCAG
CCAGGGAGGCAGCCATGAGAGGGATCACAGAAACTTCCACGCTCTTAATAGAGGATTCGTCCCCGTATACTCCCAGTTAC
TTCCACTACACAGAGACTGACAGAAAAGACCTTCTGAGACTGGGAGCCGTATACGATGAGGACAAAAGGTATTGGGTCCT
GCAGGGAAAACCTGTAATGCCTGACCAATTCACCTTTGAGCTCTTAGACTCCCTACACCGGCTCACCCACCTCAGCTATC
AGAAGATGAAGGCACTCCTTGACCGGGAAGAGAGCCCCTACTACATGTTAAATCGGGATAGAACTCTCCAGCACGTAACA
GAATCCTGTACAGCTTGTGCTCAAGTAAATGCTAGTAAAGCCAAAATCGGAGCAAGGGTGCGAGCGCGGGGACACCGACC
CGGCACCCATTGGGAAATCGATTTTACCGAAGTTAAGCCAGGGCTGTATGGACATAAGTATCTCCTGGTGTTTGTGGACA
CATTCTCTGGCTGGGTAGAAGCATTTCCAACCAAGCGTGAAACTGCCAGGGTTGTGACCAAGAAGCTGCTTGAAGAAATT
TTTCCAAGATTCGGGATGCCCCAGGTATTGGGGACAGATAATGGGCCTGCCTTCATCTCCCAGGTAAGTCAGTCGGTGGC
CAAGTTGCTGGGGATTGATTGGAAACTGCATTGTGCTTACAGACCCCAGAGTTCAGGTCAGGTAGAAAGAATGAACAGGA
CAATCAAGGAGACTTTAACCAAATTAACGCTTGCAGCTGGCACTAGAGACTGGGTACTCCTACTCCCCCTGGCCCTCTAC
CGAGCCCGGAATACTCCGGGCCCCCATGGACTTACTCCGTATGAAATTCTGTATGGGGCACCCCCGCCCCTTGTCAATTT
TCATGATCCTGAAATGTCAAAGTTAACTAATAGTCCCTCTCTCCAAGCTCACTTACAGGCCCTCCAAGCAGTACAACGAG
AGGTCTGGAAGCCACTGGCCGCTGCTTATCAGGACCAGCTGGATCAGCCAGTGATACCACACCCCTTCCGCGTCGGTGAC
GCCGTGTGGGTACGCCGACACCAGACTAAGAACTTAGAACCCCGCTGGAAAGGACCCTACACCGTCCTGCTGACCACCCC
CACCGCTCTCAAAGTAGACGGCATCTCTGCGTGGATACACGCCGCTCACGTAAAGGCGGCGACAACTCCTCCGGCCGGAA
CAGCATGGAAGGTCCAGCGTTCTCAAAATCCCTTAAAGATAAGATTAACCCGTGGGGCCCCCTGATAGTTATGGGGATCT
TAGTGAGGGCAGGAGCCTCGGTACAACGTGACAGCCCTCACCAGGTCTTCAATGTTACTTGGAGAGTTACCAACCTAATG
ACAGGACAAACAGCTAACGCCACCTCCCTCCTGGGGACGATGACAGACACCTTCCCTAAACTATATTTTGACCTGTGTGA
TTTAGTAGGAGACTACTGGGATTGGGATGACCCAGATGGAGATGGTTGCCGCTCTCCCGGGGGAAGAAAAAGGACAAGAC
TGTATGACTTCTATGTTTGCCCCGGTCATACTGTACCAATAGGGTGTGGAGGGCCGGGAGAGGGCTACTGTGGCAAATGG
GGATGTGAGACCACTGGACAGGCATACTGGAAGCCATCATCATCATGGGACCTAATTTCCCTTAAGCGAGGAAACACTCC
TAAGGATCAGGGCCCCTGTTATGATTCCTCGGTCTCCAGTGGCGTCCAGGGTGCCACACCGGGGGGTCGATGCAACCCCC
TAGTCTTAGAATTCACTGACGCGGGTAAAAAGGCCAGCTGGGATGCCCCCAAAGTTTGGGGACTAAGACTCTACCGATCC
ACAGGGGCCGACCCGGTGACCCGGTTCTCTTTGACCCGCCAGGTCCTCAATGTAGGACCCCGCGTCCCCATTGGGCCTAA
TCCCGTGATCACTGACCAGCTACCCCCATCCCAACCCGTGCAGATCATGCTCCCCAGGCCTCCTCATCCTCCTCCTTCAG
GCACGGTCTCTATGGTACCTGGGGCTCCCCCGCCTTCTCAACAGCCTGGGACGGGAGACAGGCTGCTAAATCTGGTAGAC
GGAGCCTACCAAGCACTCAACCTCACCAGTCCTGACAAAACCCAAGAGTGCTGGTTGTGTCTGGTATCGGGACCCCCCTA
CTACGAAGGGGTTGCCGTCCTAGGTACCTACTCCAACCATACCTCTGCCCCAGCTAACTGCTCCGTGGCCTCCCAACACA
AGCTGACCCTGTCCGAAGTGACCGGACAGGGACTCTGCGTAGGAGCAGTTCCCAAAACCCATCAGGCCCTGTGTAATACC
ACCCAGAAGACGAGCGACGGGTCCTACTATCTGGCTGCTCCCGCCGGGACCATCTGGGCTTGCAACACCGGGCTCACTCC
CTGCCTATCTACTACTGTACTCAACCTCACCACCGATTACTGTGTCCTGGTTGAGCTCTGGCCAAAGGTGACCTACCACT
CCCCTGGTTATGTTTATGGCCAGTTTGAGAGAAAAACCAAATATAAAAGAGAGCCGGTGTCATTAACTCTGGCCCTGCTG
TTGGGAGGACTTACTATGGGCGGCATAGCTGCAGGAGTAGGAACAGGGACTACAGCCCTAGTGGCCACCAAACAATTCGA
GCAGCTCCAGGCAGCCATACATACAGACCTTGGGGCCTTAGAAAAATCAGTCAGTGCCCTAGAAAAGTCTCTGACCTCGT
TGTCTGAGGTGGTCCTACAGAACCGGAGAGGATTAGATCTGCTGTTCCTAAAAGAAGGAGGATTATGTGCTGCCCTAAAA
GAAGAATGCTGTTTCTACGCGGACCACACTGGCGTAGTGAGAGATAGCATGGCAAAGCTAAGAGAAAGGTTAAACCAGAG
ACAAAAATTGTTCGAATCAGGACAAGGGTGGTTTGAGGGACTGTTTAACAGGTCCCCATGGTTCACGACCTTGATATCCA
CCATTATGGGCCCCTTGATAATACTTTTATTAATCCTACTCCTCGGACCCTGTATTCTCAACCGCTTGGTCCAGTTTGTA
AAAGACAGAATTTCGGTGGTGCAGGCCCTGGTTCTGACCCAACAGTATCACCAACTCAAATCAATAGATCCAGAAGAAGT
GGAGTCACGTGAATAAAAGATTTTATTCAGTTTCCAGAAAGAGGGGGGAATGAAAGACCCCACCATAAGGCTTAGCAAGC
TAGCTGCAGTAACGCCATTTTGCAAGGCATGAAAAAGTACCAGAGCTGAGTTCTCAAAAGTTACAAGGAAGTTCAGTTAA
AGATTAACAGTTAAAAATCAAGGCTGAGTAATACTAGGACAAGGGCCAAGAACCGATGGTGCCCACCTGGGCCCCGGCTC
AAGGCCAAGAACCGATGGTACCCACCTGGGCCCCGGCTCAGGGCCAAGAACAGATGGTACCCAGATAAGGCGGAACCAGC
AACAGTTTCTGAAAAAGTCCCACCTCAGTTTCAGGTTCCCCAAATGACCAGGAAATACCCCAAGCCTTGATTTGAACTAA
CCACTCAGCTCGCTTCTCGCTTCTGTACCCGCGCTTTTTGCTCCCCAGCCCTATAAAAAGGGTAAAAACCCCACACTCGG
CGCGCCAGTCCTCCGATAGACTGAGTCGCCCGGGTACCCGTGTTCCCAATAAAGCCTCTTGCTGATTACATCCGAATCGT
GGTCTCGCTGATCCTTGGGAGGGTCTCCTCAGATTGATTGACTACCCACCTCGGGGGTCTTTCA
>Xmv41

TGAAAGACCCCACCATAAGGCTTAGCAAGCTAGCTGCAGTAACGCCATTTTGCAAGGCATGAAAAAGTACCAGAGCTGAG
TTCTCAAAAGCCACAAGAAAGTTTAGTTAAAAAATAAGGCTGAACAAAACTAGGACAGGGGCCAAACAGGATATCTGTGG
TCGAGCACCTGGGCCCCGGCTCAGGGCCAAAAACAGATGGTACTCAGATAAAGCGAAACTAGCAACAGTTTCTGGAAAGT
CCCACCTCAGTTTCAAGTTCCCCAAAAGACCGAGAAAAACCCCAAGCCTTATTTAAACTAACCAATCAGCTCGCTTCTCG
CTTCTGTAACCGCGCTTTTTGCCCCCCAGCCCAAGCCCTATAAAAAGGGTAAAAACTCCACACTCGGCGCGCCAGTCATC
CGATAGACTGAGTCGCCCGGGTACCCGTGTTCCCAATAAAGCCTTTTGCCGTTTGCATCCGAGTCGTGGTCTCGCTGTTC
CTTGGGAGGGTCTCCTCAGAGTAATTGACTACCCAGCTCGGGGGTCTTTCATTTGGGGGCTCGTCCGGGATCCGGAGACC
CCCGCCCAGGGACCACCGACCCACCGTCGGGAGGTAAGCTGGCCAGCGATCGTTTTGTCTCCGTCTCTGTCTTTGTGCGT
GTGTGTGTGCCGGCATCTAATCTTTGCGCCTGCGTCTGTATCTGTACTAGTTAGCTAACTAGATCTGTATCTGGCGGTTC
CGTGGAAGAACTGATGAGTTCGTATTCCCGACCGCAGCCCTGGGAGACGTCTCAGAGGCATCAGGGGCCCGCTGGGTGGC
CCAATCAGTAAGTCCGAGTCCTGATCGATTCGGGCTATTTGGAGCCCCTCCTTTGTCGGAGGGGTACGTGGTTCTTTTAG
GAGACGAGAGGTCCAAGCCCTCGCCGCCTCCATCTGAATTTTTGCTTTCGGTTTTTCGCCGAAACCGCGCCGCGCGTCTT
GTCTGTCTCAGTGTTGTTCTGTCATTTGTCTGTTCATTGTTGTTTTGGACCGTTTCTAAAAATATGGGACAGACTGTAAC
CACTCCTTTGAGCCTGACCCTTGAACACTGGGGAGACGTCCAGCGCATTGCGTCCAACCAGTCCGTGGACGTCAAGAAGA
GACGCTGGGTCACCTTCTGCTCTGCCGAGTGGCCAACTTTCGATGTGGGGTGGCCACAAGATGGTACTTTTAATTTAGAC
ATCATTTCACAGGTTAAATCTAAGGTGTTCTCTCCCGGTCCCCACGGACACCCGGATCAGGTCCCATACATTGTCACCTG
GGAGGCTATTGCCTATGACCCCCCTCCGTGGGTCAAACCTTTTGTCTCTCCCAAACTCTCTCTCTCTCCAACCGCTCCCA
TCCTCCCATCCGGCCCTTCGACCCAACCTCCGCCCCGATCTGCCCTTTACCCTGCTCTTACCCCCTCTATAAAACCCAGA
CCTTCTAAACCTCAGGTTCTCTCCGATAACGGCGGACCTCTCATTGACCTTCTCACAGAAGACCCTCCGCCGTACGGAGA
ACAGGGACCGTCCTCCTCTGACGGAGATGGCGACAGAGAAGAGGCCACCTCCACTTCTGAGATTCCTGCCCCCTCTCCCA
TGGTGTCTCGCCTGCGGGGCAAAAGAGACCCCCCCGCGGCAGATTCCACCACCTCTCGGGCTTTCCCACTCCGTTTGGGG
GGTAATGGTCAGTTGCAGTACTGGCCGTTTTCCTCCTCTGATCTATATAACTGGAAAAATAATAATCCTTCCTTTTCTGA
AGATCCAGGTAAATTGACTGCCTTAATCGAGTCTGTTCTCATCACCCATCAGCCTACCTGGGATGACTGTCAGCAGTTGC
TGGGGACTCTGCTGACAGGAGAGGAGAAGCAGCGGGTGCTCCTGGAGGCTAGAAAGGCAGTCCGGGGCGACGACGGGCGC
CCCACCCAGTTGCCCAACGAGATCGAGGCCGCCTTTCCCCTCGAACGTCCCGACTGGGACTATACCACCCCTGGAGGTAG
GAACCACCTAGTTCTCTATCGCCAGCTGCTCTTAGCGGGTCTCCAGAATGCGGGCCGGAGCCCCACCAATTTGGCCAAGG
TAAAAGGGATAACTCAGGGACCCAATGAGTCTCCCTCGGCCTTTTTAGAGAGACTCAAAGAGGCCTATCGCAGGTACACT
CCTTATGACCCTGAGGACCCTGGGCAAGAAACCAATGTATCCATGTCGTTCATCTGGCAGTCCGCTCCAGACATTGGTCG
GAAGTTAGAGCGGTTAGAAGACTTAAAAAGCAAAACCTTAGGGGACTTAGTGAGAGAAGCCGAGAGGATCTTTAATAAAC
GAGAAACCCCGGAAGAAAGAGAAGAACGTATCAGGAGAGAAACAGAGGAAAAAGAAGAGCGCCGTAGGGCAGAGGATGAG
CAGAGAGAAAAAGAAAGGGACCGCAGAAGACAAAGAAAAATGAGCAAGCTATTGGCCACTGTAGTTACTGGTCAGAGACA
GGATAGACAGGGGGGAGAGCGAAGGAGGCCCCAACTCGATAAGGACCAATGCGCCTACTGCAAAGAAAAGGGACACTGGG
CTAAGGATTGCCCAAAGAAGCCACGGGGGCCCCGAGGACCGAGGCCCCAGACCTCCCTCCTGACCCTAGATGACTAGGGA
GGTCAGGGTCAGGAGCCCCCCCCTGAACCCAGGATAACCCTCACTGTCGGGGGGCAACCAGTCACCTTCCTGGTGGATAC
TGGGGCCCAACACTCCGTGCTGACCCAAAATCCTGGACCCCTAAGTGACAGGTCTGCCTGGGTCCAAGGGGCTACTGGAG
GAAAGCGGTATCGCTGGACCACAGATCGCAAGGTACACCTGGCTACCGGTAAGGTCACTCACTCTTTCCTCCATGTGCCA
GACTGCCCCTATCCTTTGCTAGGAAGAGACTTGTTGACTAAACTAAAGGCCCAGATCCACTTCGAGGGATCGGGAGCTCA
GGTTGTGGGACCAAAAGGACAGCCCCTACAGGTACTGACCCTAAGCATAGAGGATGAGTATCGGCTACATGAGACCTCAA
CAGAGCCGGATGTTTCTCTAGGGTCCACCTGGCTTTCTGACTTTCCCCAGGCCTGGGCAGAAACCGGGGGCATGAGACTG
GCAGTTCGCCAAGCGCCCCTGATTATACCTCTAAAGGCAACCTCCACCCCTGTGTCCATCAAACAATACCCCATGTCACA
CGAAGCCAGACTGGGGATCAAGCCCCACATACAGAGACTGTTGGACCAGGGAATATTGGTACCTTGCCAGTCCCCCTGGA
ACACACCCCTGCTGCCCGTTAAGAAACCAGGGACTAATGATTACAGGCCTGTCCAGGATCTGAGAGAAGTCAACAAGCGG
GTGGAAGATATCCACCCCACCGTGCCCAATCCTTACAACCTCTTAAGTGGACTCCCTCCGTCCCACCAGTGGTACACTGT
GCTCGATTTAAAAGATGCCTTTTTCTGCCTGAGACTCCACCCCACCAGTCAGCCTCTCTTCGCCTTTGAGTGGAGAGATC
CAGAAATGGGAATCTCTGGGCAATTGACCTAGACCAGACTCCCACAGGGTTTCAAAAACAGTCCCACCCTGTTTAATGAG
GCACTGCACAGAGACCTAGCAGACTTCCGGATCCAGCACCCAGACTTGATCCTGCTACAGTACGTGGATGACTTACTGCT
GGCCGCTACTTCCGAACTAGACTGCCAACAAGGTACTCGGGCCCTACTACAAACCCTAGGGGACCTCGGATACCGGGCCT
CGGCCAAGAAAGCCCAAATCTGCCAGAAACAGGTTAAATACCTGGGGTACCTTCTGAGGGAGGGTCAGAGATGGCTGACT
GAGGCTAGAAAAGAGACTGTGATGGGGCAACCCGTTCCAAAGACTCCTCGACAACTTAGGGAGTTCCTAGGGACGGCAGG
CTTCTGCCGCCTCTGGATCCCTGGGTTTGCGGAAATGGCGGCCCCCTTATACCCTCTCACCAAAACTGGGACTCTGTTTA
ATTGGGGCCCAGATCAGCAGAGGGCCTATCAAGAAATCAAACAGGCCCTCCTGACGGCCCCTGCCCTGGGATTGCCAGAC
TTGACCAAGCCCTTTGAACTCTTTGTCGATGAAAAGCAGGGCTACGCCAAAGGGGTCCTAACGCAGAAACTGGGACCTTG
GCGCCGGCCTGTGGCCTACCTGTCCAAAAAGCTGGACCCAGTGGCAGCCGGGTGGCCCCCCTGCTTACGGATGGTAGCAG
CCATTGCCATTCTGACAAAGGATGCAGGCAAACTAACGATGGGACAGCCGCTAGTCATTCTGGCCCCCCATGCGGTAGAA
GCACTGGTCAAACAACCCCCTGACCGTTGGCTATCCAATGCCCGCATGACCCACTATCAGGCCATGCTCCTGGATACGGA
ACGGGTTCAGTTCGGACCGGTGGTGACCCTCAACCCGGCCACCTTGCTCCCCCTACCGGAAAAGGAGCCCCCCATGACT
GCCTCGAGATCTTGGCTGAGACACATGGAACCAGACCGGACCTCACGGACCAGCCCATCCCAGACGCCGACCACACCTGG

TATACAGATGGAAGCAGCTTCCTACAAGAAGGACAGCGGAGAGCTGGAGCCGCGGTGACCACTGAGACCGAGGTAATCTG
GGCAAGGGCACTGCCGGCTGGAACATCCGCCCAGCGAGCCAAACTGATAGCACTCACCCAAGCCTTAAAAATGGCAGAAG
GTAAGAAGCTAAACGTTTACACTGATAGCCGCTATGCCTTCGCCACGGCCCATGTCCATGGAAAAATATATAGGAGGCGA
GGGTTGCTGACCTCAGAAGGCAGAGAAATTAAAAACAAAAGCGAGATCTTGGCCTTGCTAAAAGCTCTCTTTCTGCCCAA
AAGACTTAGTATAATTCATTGTCCAGGACATCAGAAAAGAGACAGTGCCGAAGCCAGAGGCAACCGTATGGCAGACCAGG
CGGCCCGAGAGGCAGCCACAAAGACAGTTCCAGAAGCCTCTACACTCCTTATAGAGGACTCGACCCCGTACACGCCTGCC
TATTTCCATTACACCGAAACAGATCTAAAAAGATTACGAGAACTGGGAGCCACCTATAACCAGATAAAAGGATATTGGGT
CCTACAAGGCAAGCCGGTGATGCCCGATCAGTTTGTGTTTGAACTATTAGACTCCCTTCACAGACTCACCCATCTCAGCC
CTCAAAAGATGAAGGCACTCCTTGACAGAGAAGAAAGCCCCTACTACATGTTAAACCGAGACAGAACTCTTCAGTATGTG
GCGGAATCCTGCACAACTTGTGCTCAAGTGAATGCTAGTAAAGCCAAAATCGGGGCAGGGGTACGAGTACGCGGACATCG
ACCAGGTACCCATTGGGAAATTGACTTCACTGAAGTTAAACCAGGGCTGTACGGGTACAAGTACCTCCTGGTGTTCGTAG
ACACCTTCTCTGGCTGGGTGAAAGCCTTCCCAACTAAACGTGAAACTGCCAAGGTTGTAACCAAGAAGCTATTAGAAGAA
ATATTCCCAAGATTCGGGATGCCACAGGTATTGGGTTCCGATAATGGGCCTGCCTTCGTCTCCCAGGTAAGTCAGTCGGT
GGCCGATTTACTGGGGATCGATTAGAAATTACATTGTGCTTATAGACCCCAGAGTTCAGGTCAGGTAAAAAGAATAAATA
GAACCATCAAGGAGACTCTAACTAAATTAACGCTTGCAGCTGGCACTAGAGACTGGGTACTCCTACTCCCCTTAGCCCTC
TACCGAGCCCGGAACACTCCGGGCCCCCATGGACTGACCCCGTATGAAATTCTGTATGGGGCACCCCCGCCCCTTGTCAA
TTTTCATGATCCTGAAATGTCAAAGTTAACTAATAGTCCCTCTCTCCAAGCTCACTTACAGGCCCTCCAAGCAGTACAAC
GAGAGGTCTGGAAGCCGCTGGCCGCTGCTTATCGGGACCAGCTAGATCAGCCAGTGATACCACACCCCTTCCGTGTCGGT
GACGCCGTGTGGGTACGCCGGCACCAGACTAAGAACTTGGAACCTCGCTGGAAAGGACCCTACACCGTCCTGCTGACCAC
CCCCACCGCTCTCAAAGTTGACGGCATCTCTGCGTGGATACACGCCGCTCACGTAAAGGCGGCGACAACTCCTCCGGCCG
GAGCAGCATGGAAGGTCCAGCGTTCTCAAAACCCCTTAAAGATAAGATTAACCCGTGGGGCCCCCTAATAGTTATGGGGA
TCTTGGTGAGGGCAGGAGCCTCGGTACAACGTGACAGCCCTCACCAGGTCTTCAATGTCACTTGGAGAGTTACCAACCTA
ATGACAGGACAAACAGCTAACGCTACCTCCCTCCTGGGGACGATGACAGACACCTTCCCTAAACTATATTTTGACTTGTG
TGATTTAGTTGGAGACAACTGGGATGACCCGGAACCCGATATTGGAGATGGTTGCCGCTCTCCGGGGGGAAGAAAAAGGA
CAAGACTATATGATTTCTATGTTTGCCCCGGCCATACTGTGCTAACAGGGTGTGGAGGGCCGAGAGAGGGCTACTGCGGC
AAATGGGGATGTGAGACCACTGGACAGGCATACTGGAAGCCATCATCATCATGGGACCTAATTTCCCTTAAGCGAGGAAA
CACTCCTAAGGGTCAGGGCCCCTGTTATGATTCCTCAGTGGTCTCCAGTAGCGTCCAGGGGGCCACACCGGGGGGTCGAT
GCAACCCCCTAGTCCTAGAATTCACTGACGCAGGTAAAAAGGCCAGCTGGGATGCCCCCAAAGTTTGGGGACTAAGACTC
TACCGATCCACAGGGGCCGACCCGGTGACCCGGTTCTCTTTGACCCGCCAGGTCCTCAATGTAGGACCCCGCGTCCCCAT
TGGGCCTAATCCCGTGATCACTGACCAGCTACCCCCATCCCAACCCGTGCAGATCATGCTCCCCAGGCCTCCTCATCCTC
CTCCTTCAGGCACGGTCTCTATGGTGCCTGGGGCTCCCCCGCCTTCTCAACAACCTGGGACGGGGGACAGGCTGCTAAAC
CTAGTAGAAGGAGCCTACCAAGCACTCAACCTCACCAGTCCCGACAGAACCCAAGAGTGCTGGCTGTGTCTGGTATCGGG
ACCCCCCTACTACGAAGGGGTTGCCGTCCTAGGTACCTACTCCAACCATACCTCTGCCCCAGCTAACTGCTCCGTGGCCT
CCCAACACAAGCTGACCCTGTCCGAAGTGACCGGACAGGGACTCTGCGTAGGAGCAGTTCCCAAAACCCATCAGGCCCTG
TGTAATACCACCCAAAAGACGAGCGACGGGTCCTACTATCTGGCTGCTCCCGCCGGGACCATCTGGGCTTGCAACACCGG
GCTCACTCCCTGCCTATCTACCACTGTACTCAACCTCACCACCGATTACTGTGTCCTGGTTGAGCTCTGGCCAAAGGTGA
CCTACCACTCCCCTGGTTATGTTTATGGCCAGTTTGAGAGAAAAACCAAATATAAAAGAGAGCCGGTGTCATTAACTCTG
GCCCTGCTGTTGGGAGGACTTACTATGGGCGGCATAGCTGCAGGAGTAGAAACAGGGACTACAGCCCTAGTGGCCACCAA
ACAATTCGAGCAGCTCCAGGCAGCCATACATACAGACCTTGGGGCCTTAGAAAAATCAGTCAGTGCCCTAGAAAAGTCTC
TGACCTCGTTGTCTGAAGTGGTCCTACAGAACCGGAGAGGATTAGATCTGCTGTTCCTAAAAGAAGGAGGATTATGTGCT
GCTCTAAAAGAAGAATGCTGTTTCTATGCAGACCACACTGGCGTAGTAAGGGATAGCATGGCTAAGCTGAGAGAAAGGCT
AAACCAGAGACAAAAATTGTTCGAATCAGGACAAGGGTGGTTTGAGGGACTGTTTAACAGGTCCCCATGGTTCACGACCC
TGATATCCACCATTATGGGCCCTCTGATAGTACTTTTATTAATCCTACTCCTCGGACCCTGCATTCTCAACCGCTTGGTC
CAGTTTGTAAAAGACAGAATTTCGGTGGTGCAGGCCCTGGTTCTGACCCAACAGTATCACCAACTCAAATCAATAGATCC
AGAAGAAGTGAAATCACGTGAATAAAAGATTTTATTCAGTTTCCAGAAAGAGGGGGGAATGAAAGACCCCACCATAAGGC
TTAGCAAGCTAGCTGCAGTAACGCCATTTTGCAAGGCATGAAAAAGTACCAGAGCTGAGTTCTCAAAAGCCACAAGAAAG
TTTAGTTAAAAAATAAGGCTGAACAAAACTAGGACAGGGGCCAAACAGGATATCTGTGGTCGAGCACCTGGGCCCCGGCT
CAGGGCCAAAAACAGATGGTACTCAGATAAAGCGAAACTAGCAACAGTTTCTGGAAAGTCCCACCTCAGTTTCAAGTTCC
CCAAAAGACCGAGAAAAACCCCAAGCCTTATTTAAACTAACCAATCAGCTCGCTTCTCGCTTCTGTAACCGCGCTTTTTG
CCCCCCAGCCCAAGCCCTATAAAAAGGGTAAAAACTCCACACTCGGCGCGCCAGTCATCCGATAGACTGAGTCGCCCGGG
TACCCGTGTTCCCAATAAAGCCTTTTGCCGTTTGCATCCGAGTCGTGGTCTCGCTGTTCCTTGGGAGGGTCTCCTCAGAG
TAATTGACTACCCAGCTCGGGGGTCTTTCA
>Xmv42
TGAAAGACCCCATCATAAGGCTTAGCAAGCTAGCTGCAGTAACGCCATTTTGCAAGGCATGAAAAAGTACCAGAGCTGAG
TTCTCAAAAGCTACAAGAAAGTTCAGTAAAAGATTAACAGTTAAAGATTAAGGCTGAATAATACTGGGACAGGGGCCAAA
TATCGGTGGTCAAGCACCTGGGCCCCGGCTCAGGGCCAAGAACAGATGGCTCTCAGACGTCAGTGTTAGCAGAACTAGCT
TCACTGATTTAGAAAAATAGAGGTGCACAGTGCTCTGGCCACTCCTTGAACCTGTGTGTCTGTCAACGTTCTGACCAGGT

GTGTGCCCATTGTTGAACCTTCATTAGACCCTTTCCTCGTACCCCTCCCATACCCATTTCTTGAGAATAGACATTGTTTA
GAACTAAAAAGTCCCACCTCAGTTTCCCCAAATGACCGGGAAATACCCCAAGCCTTATTCGAACTAACCAACCAGCTCGC
TTCTCGCTTCTGTAACCGCGCTTTTTGCTCCCCAGCCCCAGCCCTATAAAAAGGGTAAAAACTCCACACTCGGCGCGCCA
GTCCTCCGATAGACTGAGTCGCCCGGGTACCCGTATTCCCAATAAAGCCTCTTGCTGCTTGCATCCGAATCGTGGTCTCG
CTGGTCCTTGAGAGGGTCTCCTCAAATTGATTGACTTCCCACGTCGGGGGTCTTTCATTTGGAGGTCCCACCGAGATTTG
GAGACCCCTGCCCAGGGACCACCGACCCCCCGCCGGGAGGTAAGCTGGCCAGCGGTCGTTCCGTGTCTGTCTCTGTCTTC
GTGCGTGTTTGTGCCGGCATCCAATGTTTGCGCCTGCGTCTGTACTAGTTAGCTAACTAGATCTGTATCTGGCGGTTCCG
CGGAAGAACTGACGAGTTCGTATTCCCGGCCGCAGCCCTGGGAGACGTCCCAGCGGCCTCGGGGGCCCGTTTTGTGGCCC
ATTCTGTATCAGTTAACCTACCCGAGTCGGACTTTTTGGAGCTCCGCCACTGTACGTGGCTTTGTTGGGGGACGAGAGAC
AGAGACACTTCCCGCCCCCGTCTGAATTTTTGCTTTCGGTTTTACGCCGAAGCCGCGCCGCGCGCCTGATTTGTTTGTTG
TTCTTTTGTTCTTCGTTAGTCTTCTTCTGTCTTTAAGTGTTTTCGAGATCATGGGACAGACCGTAACTACCCCTCTGAGT
CTAACCTTGCAGCACTGGGGAGATGTCCAGCGCATTGCATCCAATCAGTCTGTGGATGTCAGGAAGAGGCGCTGGGTTAC
CTTCTGTTCCGCCGAATGGCCAACTTTCAATGTGGGATGGCCTCAGGATGGTACTTTTAATTTAGGTATTATCTCTCAGG
TCAAGTCTAGAGTGTTTTGTCCTGGTCCCCACGGACACCCGGATCAGGTCCCATATATCGTCACCTGGGAGGCACTTGCC
TATGACCCCCCTCCGTGGGTCAAACCGTTTGTCTCTCCAAAACCCCCTCCTTCACCGACAGCTCCCGTCCTCCCGCCCGA
TCCTTCTGCGCAACCTCCGTCCCGATCTGCCCTTTACCCTGCCCTTACCCCCTCTATAAAGTCCAAACCTCCTAAGCCCC
AGGTTCTCCCTGATAGCGGCGGACCCCTCATTGACCTTCTCACAGAGGACCCCCCGCCGTACAGAGCACAACCCTCCTCC
TCTGCCAGGGAGAACGACGAAGAAGAGGCGGCCACCTCCGAGGTTTCCCCCCCTTCTCCCATGGTGTCTCGACTGCGGGG
AAGGAGAGACCCTCCCGCAGTGGACTCCACCACCTCCCAGGCATTTCCACTCCGCATGGGGGGAGATGGCCAGCTTCAGT
ATTGGCCGTTTTCCTCTTCGGATTTATACAATTGGAAAAATAATAACCCTTCCTTTTCTGAAGATCCAGGTAAATTGACG
GCCTTGATTGAGTCCGTCCTCATCACCCACCAGCCCACCTGGGACGACTGTCAGCAGTTGCTGGGGACCCTGCTGACCGG
AGAAGAAAAGCAGCGGGTGCTCCTAGAGGCTAGAAAGGCAGTCCGGGGCAATGATGGACGCCCCACTCAGTTGCCTAATG
AAGTCAATGCTGCTTTTCCCCTTGAACGCCCCGATTGGGATTACACCACTACAGAAGGTAGGAACCACCTAGTCCTCTAT
CGCCAGTTGCTCTTAGCGGGTCTCCAAAACGCGGGCAGAAGCCCCACCAATTTGGCCAAGGTAAAAGGAATAACCCAGGG
ACCTAATGAGTCTCCCTCAGCCTTTTTAGAGAGACTCAAGGAGGCCTATCGCAGGTACACTCCTTATGACCCTGAGGACC
CAGGGCAAGAAACCAATGTGTCTATGTCATTCATCTGGCAGTCTGCCCCGGATATCGGGCGAAAGTTAGAGCGGTTAGAA
GATTTAAAGAGCAAGACTTTAGGAGACTTAGTGAGGGAAGCTGAAAAGATCTTTAATAAGCGAGAAACCCCGGAAGAAAG
AGAGGAACGTATCAGGAGAGAAACCGAGGAAAGAGAAGAACGCCGTAGGGCAGAAGATGAGCAGAGAGAGAAAGAAAGGG
ACCGCAGAAGACATAGAGAGATGAGCAAGCTCTTGGCCACTGTAGTTATTGGTCAGAGACAGGATAGACAGGGGGGAGAG
CGGAGGAGGCCCCAACTTGATAAGGACCAATGCGCCTACTGCAAAGAAAAGGGACACTGGGCTAAGGACTGCCCAAAGAA
GCCACGAGGGCCCCGAGGACCGAGGCCCCAGACCTCCCTCCTGACCTTAGGTGACTAGGGAGGTCAGGGTCAGGAGCCCC
CCCCTGAACCCAGGATAACCCTTAAAGTCGGGGGGCAACCCGTCACCTTCCTGGTAGATACTGGGGCCCAACACTCCGTG
CTGACCCAAAATCCTGGACCCCTAAGTGACAAGGCTGCCTGGGTCCAAGGGGCTACTGGAGGAAAGCGGTATCGCTGGAC
CACGGATCGCAAAGTACATCTAGCTACCGGTAAGGTCACCCACTCTTTCCTCCATGTACCAGACTGCCCCTATCCTCTGC
TAGGAAGAGACTTGCTGACTAAACTAAAAGCCCAGATCCACTTCGAGGGATCAGGAGCTCAGGTTGTGGGACCAATGGGA
CAGCCCCTGCAAGTGCTGACCCTAAACATAGAAGATGAGTATCGGCTACATGAGACCTCAAAAGAGCCGGATGTTTCTCT
AGGGTCCACATGGCTTTCTGATTTTCCCCAGGCCTGGGCAGAAACCGGGGGCATGGGACTGGCAGTTCGCCAAGCTCCTC
TGATCATACCTCTGAAGGCAACCTCTACCCCCGTGTCCATAAAACAATACCCCATGTCACAGGAAGCCAGACTGGGGATC
AAGCCCCACATACAGAGGCTGTTGGACCAGGGAATACTGGTACCCTGCCAGTCCCCCTGGAACACGCCCCTGCTACCCGT
TAAGAAACCAGGGACTAATGATTACAGGCCTGTCCAGGATCTGAGAGAAGTCAACAAGCGGGTGGAAGACATCCACCCCA
CCGTGCCCAACCCTTACAACCTCTTGAGCGGGCTCCCACCGTCCCACCAGTGGTACACTGTGCTTGATTTAAAGGATGCC
TTTTTCTGCCTGAGACTCCACCCCACCAGTCAGCCTCTCTTCGCCTTTGAGTGGAGAGATCCAGGGATGGGAATCTCAGG
ACAATTGACCTGGACCAGACTCCCACAGGGTTTCAAAAACAGTCCCACCCTGTTTGATGAGGCACTGCACAGAGACCTAG
CAGACTTCCGGATCCAGCACCCAGACTTGATCCTGCTACAGTACGTGGATGACATACTGCTGGCCGCCACTTCTGAGCTC
GACTGCCAACAAGCTCACTTACAGGCCCTCCAAGCAGTACAACGAGAGGTCTGGAGGCCACTGGCTGCTGCTTATCAGGA
CCAGCTGGATCAGCCAGTGATACCACACCCCTTCCGTGTCGGTGACGCCGTGTGGGTACGCCGGCACCAGACTAAGAACT
TAGAACCCCGCTGGAAAGGACCCTACACCGTCCTGCTGACCTCCCCCACCGCTCTCAAAGTAGACGGCATCTCTGCGTGG
ATACACGCCGCTCACGTAAAGGCGGCGACAACTCCTCCGGCCGGAACAGCATGGAAGGTCCAGCGTTCTCAAAATCCCTT
AAAGATAAGATTAACCCGTGGGGCCCCCTGATAGTTATGGGGATCTTAGTGAGGGCAGGAGCCTCGGTACAACGTGACAG
CCCTCACCAGGTCTTCAATGTTACTTGGAGAGTTACCAACCTAATGACAGGACAAACAGCTAACGCCACCTCCCTCCTGG
GGACGATGACAGACACCTTCCCTAAACTATATTTTGACCTGTGTGATTTAGTAGGAGATTACTGGGATGACCCAGATGGA
GATGGTTGCCGCTCTCCCGGGAGAAGAAAAAGGACAAGACTGTATGACTTCTATGTTTGCCCCGGTCATACTGTACCAGT
AGGGTGTGGAGGGCCGAGAGAGGGCTACTGTGGCAAATGGGGATGTGAGACCACTGGACAGGCATACTGGAAGCCATCAT
CATCATGGGACCTAATTTCCCTTAAGCGAGGAAACACTCCTAAGGATCAGGGCCCCTGTTATGATTCCTCGGTCTCCAGT
GACGTCCAGGGTGCCACACCGGGGGGTCGATGCAACCCCCTAGTCTTAGAATTCACTGACGCGGGTAAAAAGGCCAGCTG
GGATGCCCCCAAAGTTTGGGGACTAAGACTCTATCGATCCACAGGGGCCGACCCGGTGACCCGGTTCTCTTTGACCCGCC
AGGTCCTCAATGTAGGACCCCGCGCCCCCATTGGGCCTAATCCCGTGATCGCTGACCAGCTACCCCCATCCCAACCCGTG

CAGATCATGCTCCCCAGGCCTCCTCATCCTCCTCCTTCAGGCACGGTCTCTATAGTACCTGGGGCTCCCCCGCCTTCTCA
ACAACCTGGGACGGGAGACAGGCTGCTAAATCTGGTAAAAGGAGCCTACCAAGCACTCAACCTCACCAGTCCTGACAAAA
CCCAAGAGTGCTGGTTATGTCTGGTATCGGGACCCCCCTACTACGAAGGGGTTGCCGTCCTAGGTACCTACTCCAACCAT
ACCTCTGCCCCAGCTAACTGCTCCGTGGCCTCCCAACACAAGCTGACCCTGTCCGAAGTGACCGGACAGGGACTCTGCGT
AGGAGCAGTTCCCAAAACCCATCAGGCCCTGTGTAATACCACCCAGAAGACAAGCGACGGGTCCTACTATCTGGCTGCTC
CCGCCGGGACCATCTGGGCTTGCAACACCGGGCTCACTCCCTGCCTATCTACTACTGTACTCAACCTCACCACCGATTAC
TGTGTCCTGGTTGAACTCTGGCCAAGGGTGACCTACCACTCCCCTGGTTATGTTTATGGCCAGTTTGAGAGAAAAACCAA
ATATAAAAGAGAGCCGGTGTCATTAACTCTGGCCCTGCTGTTGGGAGGACTTACTATGGGCGGCATAGCTGCAGGAGTAG
GAACAGGGACTACAGCCCTGGTGGCCACCAAACAATTCGAGCAGCTCCAGGCAGCCATACATACAGACCTTGGGGCCTTA
GAAAAATCAGTCAGTGCCCTAGAAAAGTCTCTGACCTCGTTGTCTGAGGTGGTCCTACAGAACCGGAGAGGATTAGATCT
GCTGTTCCTAAAAGAAGGAGGATTATGTGCTGCCCTAAAAGAAGAATGCTGTTTCTACGCGGACCACACTGGCGTAGTGA
GAGATAGCATGGCAAAGCTGAGAGAAAGGTTAAACCAGAGACAAAAATTGTTCGAATCAGGACAAGGGTGGTTCGAGGGA
CTGTTTAACAGGTCCCCATGGTTCACGACCTTGATATCCACCATTATGGGCCCCTTGATAATACTTTTATTAATCCTACT
CTTCGGACCCTGTATTCTCAACCGCTTGGTCCAGTTTGTAAAAGACAGAATTTCGGTGGTGCAGGCCCTGGTTCTGACCC
AACAGTATCACCAACTCAAATCAATAGATCCAGAAGAAGTGGAATCACGTGAATAAAAGATTTTATTCAGTTTCCAGAAA
GAGGGGGGAATGAAAGACCCCATCATAAGGCTTAGCAAGCTAGCTGCAGTAACGCCATTTTGCAAGGCATGAAAAAGTAC
CAGAGCTGAGTTCTCAAAAGCTACAAGAAAGTTCAGTAAAAGATTAACAGTTAAAGATTAAGGCTGAATAATACTGGGAC
AGGGGCCAAATATCGGTGGTCAAGCACCTGGGCCCCGGCTCAGGGCCAAGAACAGATGGCTCTCAGACGTCAGTGTTAGC
AGAACTAGCTTCACTGATTTAGAAAAATAGAGGTGCACAGTGCTCTGGCCACTCCTTGAACCTGTGTGTCTGTCAACGTT
CTGACCAGGTGTGTGCCCATTGTTGAACCTTCATTAGACCCTTTCCTCGTACCCCTCCCATACCCATTTCTTGAGAATAG
ACATTGTTTAGAACTAAAAAGTCCCACCTCAGTTTCCCCAAATGACCGGGAAATACCCCAAGCCTTATTCGAACTAACCA
ACCAGCTCGCTTCTCGCTTCTGTAACCGCGCTTTTTGCTCCCCAGCCCCAGCCCTATAAAAAGGGTAAAAACTCCACACT
CGGCGCGCCAGTCCTCCGATAGACTGAGTCGCCCGGGTACCCGTATTCCCAATAAAGCCTCTTGCTGCTTGCATCCGAAT
CGTGGTCTCGCTGGTCCTTGAGAGGGTCTCCTCAAATTGATTGACTTCCCACGTCGGGGGTCTTTCA
>Xmv43
TGAAAGACCCCACCATAAGGCTTAGCAAGCTAGCTGCAGTAACGCCATTTTGCAAGGCATGAAAAAGTACCAGAGCTGAG
TTCTCAAAAGTCACAAGGAAGTTTAGTTAAAGAATAAGGCTGAACAAAACTGGGACAGGGGCCAAACAGGATATCTGTGG
TCGAGCACCTGGGCCCCGGCTCAGGGCCAAGAACAGATGGTACTCAGATAAAGCGAAACTAGCAACAGTTTCTGGAAAGT
CCCACCTCAGTTTCAAGTTCCCCAAAAGACCGGGAAAAACCCCAAGCCTTATTTAAACTAACCAATCAGCTCGCTTCTCG
CTTCTGTAACCGCGCTTTTTGCTCCCCAGCCCTATAAAAAGGGTAAAAACCCCACACTCGGTGCGCCAGTCATCCGATAG
ACTGAGTCGCCCGGGTACCCGTGTTCCCAATAAAGCCTTTTGCTGTTTGCATCCGAAACGTGGCCTCGCTGTTCCTTGGG
AGGGTCTCCTCAGAGTGATTGACTACCCAGCTCGGGGGTCTTTCATTTGGGGGCTCGTCCGGGATTTGGAGACCCCCGCC
CAGGGACCACCGACCCACCGTCGGGAGGTAAGCTGGCCAGCGATCGTTTTGTCTCCGTCTCTGTCTTTGTGCGTGTGTGT
GTGTGCCGGCATCTACTTTTTGCGCCTGCGTCTGAATCTGTACTAGTTAGCTAACTAGATCTGTATCTGGCGGTTCCGTG
GAAGAACTGACGAGTTCGTATTCCCGACCGCAGCCCTGGGAGACGTCTCAGAGGCATCAGGGGCCCGCTGGGTGGCCCAA
TCAGTAAGTCCGAGTCCTGACCGATTCGGACTATTTGGAGCCCCTCCTTTGTCGGAGGGGTACGTGGTTCTTTTAGGAGA
CGAGAGGTCCAAGCCCTCGCCGCCTCCATCTGAATTTTTGCTTTCGGTTTTTCGCCGAAACCGCGCCGCGCGTCTTGTCT
GTCTCAGTGTTGTTTTGTCATTTGTCTGTTCGTTATTGTTTTGGACCGTTTCTAAAAATATGGGACAGACCGTAACCACC
CCTCTGAGTCTGACCCTAGAACACTGGGGAGACGTCCAGCGCATCGCGTCCAACCAGTCCGTGGACGTCAAGAAGAGACG
CTGGGTCACCTTCTGCTCTGCCGAGTGGCCAACTTTCGGTGTAGGGTGGCCGCAAGATGGTACTTTTAATTTGGACATTA
TTTTACAGGTTAAATCTAAGGTGTTCTCTCCCGGTCCCCACGGACACCCGGATCAGGTCCCATACATTGTCACCTGGGAG
GCTATTGCCTATGAACCCCCTCCGTGGGTCAAACCTTTTGTCTCTCCCAAACTCTCCCTCTCTCCAACCGCTCCCATCCT
CCCATCCGGTCCTTCGACCCAACCTCCGCCCCGATCTGCCCTTTACCCTGCTCTTACCCCCTCTATAAAACCCAGACCTT
CTAAACCTCAGGTTCTCTCCGATAATGGCGGACCTCTCATTGACCTTCTCACAGAAGACCCTCCGCCGTACGGAGAACAG
GGACCGTCCTCCTCTGACGGAGATGGCGACAGAGAAGAGGCCACCTCCACTCCTGAGATTCCTGCCCCCTCTCCCATGGT
GTCTCGCTTGCGGGGCAAAAGAGACCCCCCCGCGGCAGTTTCCACCACCTCTCGGGCTTTCCCACTCCGTTTGGGGGGTA
ATGGTCAGTTGCAGTACTGGCCGTTTTCCTCCTCGGATCTATATAACTGGAAAAATAATAACCCTTCCTTCTCTGAAGAT
CCAGGTAAATTGACTGCCTTAATCGAGTCTGTCCTCACCACCCACCAGCCTACTTGGGATGACTGTCAACAGTTGCTGGG
GACTCTGCTGACAGGAGAAGAAAAGCAGCGGGTGCTCCTGGAAGCCAGAAAGGCAGTCCGGGGCGACGATGGCCGCCCCA
CCCAATTGCCCAATGAGATCGAGGCTGCCTTTCCCCTCGAACGTCCCGACTGGGACTACACCACCCTTAGAGGTAGGAAC
CACCTAGTTCTCTATCGCCAGCTGCTCTTGGCGGGTCTCCAAAATGCGGGCAGGAGCCCCACCAATTTGGCTAAGGTAAA
AGGAATAACCCAGGGGTCCAACGAGTCGCCCTCGGCCTTTCTAGAGAGACTCAAAGAGGCCTATCGCAGATACACTCCTT
ATGACCCTGAGGACCCTGGGCAAGAAACCAATGTATCCATGTCGTTCATCTGGCAGTCTGCTCCAGACATTGGTCGAAAG
TTAGAGCGGTTAGAAGACTTAAAAAATAAGACCTTAGGGGACTTAGTGAGAGAAGCAGAAAGGATCTTTAATAAGAGAGA
GACCCCAGAAGAGAGAGAAGAACGTATTAAGAGAGAAACAGAGGAAAAAGAGGAGCGCCGTAGGGCAGAGGATGAGCAGA
AAGAGAAAGAGAGGGACCGCAGAAGACAGAGAGAAATGAGCAAACTCTTGGCCACCGTAGTTACAGGTCAGAGACAGGAT
AGACAGGGGGGAGAGCGAAGGAGGCCCCAACTCGATAAGGACCAATGCGCCTACTGCAAAGAAAAGGGACACTGGGCTAG

GGATTGCCCAAAGAAGCCACGGGGGCCCCGAGGACCGAGGCCCCAGACCTCCCTCCTGACCCTAGATGACTAGGGAGGTC
AGGGTCAGGAGCCCCCCCCTGAACCCAGGATAACCCTTACTGTCGGGGGGCAACCAGTCACCTTCCTGGTGGATACTGGG
GCCCAACACTCCGTGCTGACCCAGAACCCTGGACCCCTAAGTGACAGGTCTGCCTGGGTCCAAGGGGCTACTGGAGGAAA
GCGGTATCACTGGACCACAGATCGCAAGGTGCACCTGGCTACCGGTAAGGTCACTCACTCTTTCCTCCATGTGCCGGACT
GCCCTTATCCTTTGCTAGGAAGGGACTTGTTGACTAAGTTAAAGGCCCAGATCCACTTCGAGGGATCGGGAGCTCAGGTT
GTGGGACCAAAAGGACAGCCCCTGCAGGTGTTGACCCTTGGCATAGAGGATGAGTATCGGCTACATGAGACCTCAACAGA
GCCGGATGTTTCTCTAGGGTCCACCTGGCTTTCTGACTTTCCCCAGGCCTGGGCAGAAACCGGGGGCATGGGACTGGCAG
TTCGCCAAGCGCCTCTGATTATACCTCTAAAGGCAACCTCCACCCCTGTGTCCATCAAACAGTACCCCATGTCACACGAA
GCCAGACTGGGGATCAAGCCCCACATACAGAGACTGTTGGACCAGGGAATATTGGTACCTTGCCAGTCCCCCTGGAACAC
ACCCCTGCTGCCCGTTAAGAAACCAGGGACTAATGATTACAGGCCTGTCCAGGATCTGAGAGAAGTCAACAAGCGGGTGG
AAGATATCCACCCCACCGTGCCCAATCCTTACAACCTCTTAAGTGGACTCCCTCCGTCCCACCAGTGGTACACTGTGCTT
GATTTAAAAGATGCCTTTTTCTGCCTGAGACTCCACCCCACCAGTCAGCCTCTCTTTGCCTTTGAGTGGAGAGATCCAGA
AATGGGAATCTCTGGACAATTGACCTGGACCAGACTCCCACAGGGTTTCAAAAACAGTCCCACCCTGTTTGATGAGGCAT
TGCACAGAGACCTAGCAGACTTCCGGATCCAGCACCCAGACTTGATCCTGCTACAGTACGTGGATGACTTACTGCTGGCC
GCTACTTCCGAACTAGACTGCCAACAAGGTACTCGGGCCCTTCTACAAACCCTAGGGGACCTCGGATACCGGGCCTCGGC
CAAGAAAGCCCAAATCTGCCAGAAACAGGTTAAATACCTGGGGTACCTTCTGAGGGAGGGTCAGAGATGGCTGACTGAGG
CTAGAAAAGAGACTGTGATGGGGCAACCCGTTCCAAAGACTCCTCGACAACTAAGGGAGTTCCTAGGGACGGCAGGCTTC
TGCCGCCTCTGGATCCCTGGGTTTGCGGAAATGGCGGCCCCCTTGTATCCTCTTACCAAAACGGGGACTCTGTTTAATTG
GGGCCCAGACCAGCAAAAGGCCTATCAAGAAATCAAACAGGCCCTTCTAACTGCCCCCGCCCTGGGATTGCCAGATTTGA
CTAAGCCCTTTGAACTCTTTGTCGACGAGAAGCAGGGCTACGCCAAAGGCGTCCTAACGCAAAAACTGGGACCTTGGCGT
CGGCCTGTGGCCTACCTGTCCAAAAAGCTAGACCCAGTGGCAGCCGGGTGGCCCCCTTGCCTACGGATGGTAGCAGCCAT
TGCCGTTCTGACAAAAGATGCAGGCAAGCTAACTATGGGACAGCCGCTAGTCATCCTGGCCCCCCATGCAGTAGAGGCAC
TGGTCAAGCAACCCCCTGACCGCTGGCTATCCAACGCCCGCATGACCCACTACCAGGCAATGCTCCTAGACACTGACCGA
GTTCAGTTCGGACCAGTGGTGGCCCTCAATCCTGCCACCTTGCTCCCTCTACCGGAAAAAGGAGCCCCCCATGATTGCCT
CGAGATCTTGGCTGAAACGCATGGAACCAGACCGGATCTCACCGACCAGCCCATCCCAGACGCCGACCACACCTGGTATA
CCGATGGGAGCAGCTTTCTGCAAGAAGGACAGCGAAAGGCTGGGGCAGCAGTGACGACTGAAACCGAGGTAATCTGGGCG
AGGGCCCTGCCAGCTGGAACGTCAGCCCAGCGAGCCGAACTGATCGCACTCACCCAAGCCCTGAAAATGGCAGAAGGTAA
GAAGCTAAATGTTTACACTGATAGCCGCTATGCCTTCGCTACGGCCCATGTTCATGGGGAAATATATAGGAGACGGGGGT
TGCTGACCTCAGAAGGCAAGGAAATCAAGAACAAAAGCGAGATCCTAGCCTTGCTGAAAGCCCTCTTTTTGCCAAAGAGA
CTCAGTATTATCCATTGCCCAGGACATCAGAAAGGAGACAGTGCCGAAGCCAGAGGCAACCGTATGGCAGACCAGGCGGC
CCGAGAGGCAGCCACAAAAACAGTTCCAGAAGCCTCTACACTCCTTATAGAGGACTCGACCCCGTACACGCCTGCCTATC
TCCATTACACCGAAACAGATCTAAAAAGATTGCGAGAACTGGGGGCCACCTATAATCAGATAAAAGGATATTGGGTCCTA
CAAGGCAAGCCGGTGATGCCCGATCAGTTTGTGTTTGAATTATTAGACTCCCTTCATAGACTCACCCATCTCAGCCCTCA
AAAGATGAAGGCGCTCCTTGACAGAGAAGAAAGCCCCTACTACATGTTAAACAGGGACAGAACTCTTCAGTATGTGGCAG
AATCCTGCACAGTCTGTGCTCAAGTAAATGCTAGTAAAGCCAAAATCGGGGCAGGGGTACGAGTACGCGGACATCGACCA
GGTACCCATTGGGAAATTGACTTCACTGAAGTTAAACCAGGGCTGTACGGGTACAAGTACCTCCTGGTGTTCGTAGACAC
CTTCTCTGGCTGGGTGGAAGCCTTCCCAACTAAACGTGAAACTGCCAAGGTTGTGACCAAGAAGCTATTAGAAGAAATAT
TCCCAAGATTCGGGATGCCACAGGTATTGGGTTCCGATAATGGGCCTGCCTTCGTCTCCCAGGTAAGTCAGTCGGTGGCC
GATTTACTGGGGATCGATTGGAAATTACATTGTGCTTATAGACCCCAGAGTTCAGGTCAGGTAGAAAGAATGAATAGAAC
CATCAAGGAGACTCTAACTAAATTAACGCTTGCAGCTGGCACTAGAGACTGGGTACTCCTACTCCCCTTAGCCCTCTACC
GAGCCCGGAACACTCCGGGCCCCCATGGACTGACTCCGTATGAAATTCTGTATGGGGCACCCCCGCCCCTTGTCAATTTT
CATGATCCTGAAATGTCAAAGTTAACTAATAGTCCCTCTCTCCAAGCTCACTTACAGGCCCTCCAAGCAGTACAACGAGA
GGTCTGGAAGCCGCTGGCCGCTGCTTATCAGGACCAGCTAGATCAGCCAGTGATACCACACCCCTTCCGTGTCGGTGACG
CCGTGTGGGTACGCCGGCACCAGACTAAGAACTTGGAACCTCGCTGGAAAGGACCCTACACCGTCCTGCTGACCACCCCC
ACCGCTCTCAAAGTTGACGGCATCTCTGCGTGGATACACGCCGCTCACGTAAAGGCGGCGACAACTCCTCCGGCCGGAGC
AGCATGGAAGGTCCAGCGTTCTCAAAACCCCTTAAAGATAAGATTAACCCGTGGGGCCCCCTAATAGTTATAGGGATCTT
GGTGAGGGCAGGAGCCTCGGTACAACGTGACAGCCCTCACCAGGTCTTCAATGTCACTTGGAGAGTTACCAACCTAATGA
CAGGACAAACAGCTAACGCTACCTCCCTCCTGGGGACGATGACAGACACCTTCCCTAAACTATATTTTGACTTGTGTGAT
TTAGTTGGAGACCATTGGGATGACCCAGAACCCGATATTGGAGATGGTTGCCGCTCTCCGGGGGGAAGAAAAAGGACAAG
ACTGTATGACTTCTATGTTTGCCCCGGTCATACTGTACCAATAGGGTGTGGAGGGCCGGGAGAGGGCTACTGTGGCAAAT
GGGGATGTGAGACCACTGGACAGGCATACTGGAAGCCATCATCATCATGGGACCTAATTTCCCTTAAGCGAGGAAACACT
CCTAAGGATCAGGGCCCCTGTTATGATTCCTCGGTCTCCAGTGGCGTCCAGGGTGCCACACCGGGGGGTCGATGCAACCC
CCTAGTCTTAGAATTCACTGACGCGGGTAAAAAGGCCAGCTGGGATGCCCCCAAAGTTTGGGGACTAAGACTCTACCGAT
CCACGGGGGCCGACCCGGTGACCCGGTTCTCTTTGACCCGCCAGGTCCTCAATGTAGGACCCCGCGTCCCCATTGGGCCT
AATCCCGTGATCACTGAACAGCTACCCCCCTCCCAACCCGTGCAGATCATGCTCCCCAGGCCTCCTCATCCTCCTCCTTC
AGGCGCGGCCTCTATGGTGCCTGGGGCTCCCCCGCCTTCTCAACAACCTGGGACGGGGGACAGGCTGCTAAACCTAGTAA
AAGGAGCCTATCAAGCACTCAACCTCACCAGTCCCGACAGAACCCAAGAGTGCTGGCTGTGTCTGGTATCGGGACCCCCC

TACTACGAAGGGGTTGCCGTCCTAGGTACCTACTCCAACCATACCTCTGCCCCAGCTAACTGCTCCGTGGCCTCCCAACA
CAAGCTGACCCTGTCCGAAGTGACCGGGCAGGGACTCTGCGTAGGAGCAGTTCCCAAAACCCATCAGGCCCTGTGTAATA
CCACCCAGAAGGCGAGCGACGGGTCCTACTATCTGGCTGCTCCCGCCGGGACCATCTGGGCTTGCAACACCGGGCTCACT
CCCTGCCTATCTACCACTGTACTCAACCTCACCACCGATTACTGTGTCCTGGTTGAGCTCTGGCCAAAGGTGACCTACCA
CTCCCCTGGTTATGTTTATGACCAGTTTGAGAGAAAAACCAAATATAAAAGAGAGCCGGTGTCATTAACTCTGGCCCTGC
TGTTGGGAGGACTTACTATGGGCGGCATAGCTGCAGGAGTAGGAACAGGGACTACAGCCCTAGTGGCCACCAAACAATTC
GAGCAGCTCCAGGCAGCCATACATACAGACCTTGGGGCCTTAGAAAAATCAGTCAGTGCCCTAGAAAAGTCTCTGACCTC
GTTGTCTGAGGTGGTCCTACAGAACCGGAGAGGATTAGATCTGCTGTTCCTAAAAGAAGGAGGATTATGTGCTGCCCTAA
AAGAAGAATGCTGTTTCTATGCAGACCACACTGGCGTAGTAAGGGATAGCATGGCTAAGCTAAGAGAAAGGCTAAACCAG
AGGCAAAAATTGTTCGAATCAGGACAAGGGTGGTTTGAGGGACTGTTTAACAGGTCCCCATGGTTCACGACCCTGATATC
CACCATTATGGGCCCTCTGATAGTACTTTTATTAATCCTACTCCTCGGACCCTGCATTCTCAACCGCTTGGTCCAGTTTG
TAAAAGACAGAATTTCGGTGGTGCAGGCCCTGGTTCTGACCCAACAGTATCACCAACTCAAATCAATAGATCCAGAAGAA
GTAGAATCGCGTGAATAAAAGATTTTATTCAGTTTCCAGAAAGAGGGGGGAATGAAAGACCCCACCATAAGGCTTAGCAA
GCTAGCTGCAGTAACGCCATTTTGCAAGGCATGAAAAAGTACCAGAGCTGAGTTCTCAAAAGTCACAAGGAAGTTTAGTT
AAAGAATAAGGCTGAACAAAACTGGGACAGGGGCCAAACAGGATATCTGTGGTCGAGCACCTGGGCCCCGGCTCAGGGCC
AAGAACAGATGGTACTCAGATAAAGCGAAACTAGCAACAGTTTCTGGAAAGTCCCACCTCAGTTTCAAGTTCCCCAAAAG
ACCGGGAAAAACCCCAAGCCTTATTTAAACTAACCAATCAGCTCGCTTCTCGCTTCTGTAACCGCGCTTTTTGCTCCCCA
GCCCTATAAAAAGGGTAAAAACCCCACACTCGGTGCGCCAGTCATCCGATAGACTGAGTCGCCCGGGTACCCGTGTTCCC
AATAAAGCCTTTTGCTGTTTGCATCCGAAACGTGGCCTCGCTGTTCCTTGGGAGGGTCTCCTCAGAGTGATTGACTACCC
AGCTCGGGGGTCTTTCA
>Xmv8
TGAAAGACCCCACCATAAGGCTTAGCAAGCTAGCTGCAGTAACGCCATTTTGCAAGGCATGAAAAAGTACCAGAGCTGAG
TTCTCAAAAGTTACAAGGAAGTTCAGTTAAAGATTAACAGTTAAAAATCAAGGCTGAATAATACTAGGACAAGGGCCAAA
AAGGGTATATCGGTGGTCAAGCGCCTGGGCCCCGGCTCAGGGCCAAGAACCGATGGTACCCACCTGGGCCCCGGCTCAGG
GCCAAGAACAGATGGTACCCAGATAAAGCGGAACCAGCAACAGTTTCTGAAAAAGTCCCACCTCAGTTTCAGGTTCCCCA
AATGACCAGGAAATACCCCAAGCCTTGATTTAAACTAACCACTCAGCTCGCTTCTCGCTTCTGTACCCACGCTTTTTGCT
CCCCAGCCCCAGCCCTATAAAAAGGGTAAAAACCCCACACTCGGCGCGCCAGTCCTCCGATAGACTGAGTCGCCCGGGTA
CCCGTGTTCCCAATAAAGCCTCTTGCTGATTACATCCGAATCGTGGTCTCGCTGATCCTTGGGAGGGTCTCCTCAGATTG
ATTGACCACCCACCTCGGGGGTCTTTCATTTGGAGGTTCCACCGAGATTAGGAGACCCCTGCCCAGGGACCACCGACCCC
CGCCGGGAGGTAAGCTGGCCAGCGGTCGTTTCGTGTCTGTCTCTGTCTCCGTGCGTGTTTGTGCCGGCATCTAATGTTTG
CGCCTGCGTCTGTACTAGTTGGCTAACTAGATCTGAATCTGGCGGTTCCGTGGAAGAACTGACGAGTTCATATTCCCGGC
CGCAGCCCTGGGAGACGTCTCAGAGGCATCGGGGGCCATCTTTGTGGCCCAATCTGTATCTGAGAACCCGACCCGTCTCG
GACTCCTTGGAGCCTCTCCTTTGACCGAGGGATACGTGGTTCTGTTGGGCGGCGAGGGGCCGAAACGCTCCTCTCCCCCA
TCTGAATTTTTGCTTTCGGTTTTCCGCCGAAACCGCGCCGCGCGTCTTGTCTGTCTCTGTGTTGTTTTGTCATTTGTCGG
TTCGTTATTGTTTTGGACCGTTTCTAAAAATATGGGACAGACCGTAACCACTCCTTTGAGTCTGACCCTAGAACACTGGG
GAGACGTCCAGCGCATTGCGTCCAACCAGTCCGTGGACGTCAAGAAGAGACGTTGGGTCACCTTCTGCTCTGCCGAGTGG
CCAACTTTCAATGTGGGGTGGCCGCAAGATGGTACTTTTAATTTGGACATTATTTTACAGGTTAAATCTAAGGTATTCTC
TCCCGGTCCCCACGGACACCCGGATCAGGTCCCATACATTGTCACCTGGGAGGCACTTGCCTATGACCCCCCTCCGTGGG
TCAAACCGTTTGTCTCTCCAAAACCCCCTCCTTTACCGACAGCTCCCGTCCTCCCGCCCGGTCCTTCTGCGCAACCTCCG
TCCCGATCTGCCCTTTACCCTGCCCTTACCCCCTCTATAAAGCCCAAACCTCCTAAGCCCCAGGTTCTCCCTGATAGCGG
CGGACCTCTCATTGACCTTCTCACAGAGGACCCCCCGCCGTACGGAGCACAACCTTCCTCCTCTGCCAGAGAAAACAATG
AAGAAGAGGCGGCCGCCACCTCCGAGGTTTCCCCCCCTTCTCCCATGGTGTCTCGACTGCGGGGAAGGAGGGACCCTCCC
GCAGCGGACTCCACCTCCTCCCAGGCATTCCCACTCCGCATGGGGGGAGATGGCCAGCTTCAGTATTGGCCGTTTTCCTC
CTCGGACTTATACAATTGGAAAAATAATAACCCTTCCTTTTCTGAAGATCCAGGTAAATTGACGGCTTTGATTGAGTCCG
TCCTCATCACCCACCAGCCCACCTGGGACGACTGTCAGCAGTTGTTAGGGACCCTGCTGACCGGAGAAGAAAAGCAGCGG
GTGCTCCTAGAGGCTAGAAAGGCAGTCCGGGGCAATGATGGACGCCCCACTCAGTTGCCTAATGAAGTCAATGCTGCTTT
TCCCCTTGAACGCCCCGATTGGGATTACACCACTACAGAAGGTAGGAACCACCTAGTCCTCTATCGCCAGTTGCTCTTAG
CGGGTCTCCAAAACGCGGGCAGAAGCCCCACCAATTTGGCCAAGGTAAAAGGGATAACCCAGGGACCTAATGAGTCTCCC
TCAGCCTTTTTAGAGAGACTCAAGGAGGCCTATCGCAGGTACACTCCTTATGACCCTGAGGACCCAGGGCAAGAAACCAA
TGTGTCTATGTCATTCATCTGGCAGTCTGCCCCGGATATCGGGCGAAAGTTAGAGCGGTTAGAAGATTTAAAGAGCAAGA
CCTTAGGAGATTTAGTGAGGGAAGCTGAAAAGATCTTTAATAAGCGAGAAACCCCGGAAGAAAGAGAGGAACGTATCAGG
AGAGAAACAGAGGAAAAAGAAGAACGCCGTAGGGCAGAGGATGAGCAGAGAGAGAAAGAAAGGGACCGCAGGAGACATAG
AGAAATGAGCAAGCTCTTGGCCACTGTAGTTAGTGGACAGAGACAGGATAGACAGGGGGGAGAGCGAAGGAGGCCCCAAC
TTGATAAGGACCAATGCGCCTACTGCAAGGAAAAGGGACACTGGGCTAAGGACTGCCCAAAGAAGCCACGAGGGCCCCGA
GGACCGAGGCCCCAGACCTCCCTCCTGACCTTAGGTGACTAGGGAGGTCAGGGTCAGGAGCCCCCCCGAACCCAGGATA
ACCCTCAAAGTCGGGGGGCAACCCGTCACCTTCCTGGTAGATACTGGGGCCCAACACTCCGTGCTGACCCAAAATCCTGG
ACCCCTAAGTGACAAGTCTGCCTGGGTCCAAGGGGCTACTGGAGGGAAGCGGTATCGCTGGACCACGGATCGCAAAGTAC

ATCTAGCTACCGGTAAGGTCACCCACTCTTTCCTCCATGTACCAGACTGCCCCTATCCTCTGCTAGGAAGAGACTTGCTG
ACTAAACTCAAAGCCCAGATCCACTTCGAGGGATCAGGAGCTCAGGTTGTGGGACCGATGGGACAGCCCCTGCAAGTGCT
GACCCTAAACATAGAGGATGAGTATCGGCTACATGAGACCTCAACAGAGCCGGATGTTTCTCTAGGGTCCACCTGGCTTT
CTGATTTTCCCCAGGCCTGGGCGGAAACTGGGGGCATGGGACTGGCAGTTCGCCAAGCGCCTCTGATTATACCTCTGAAG
GCAACCTCCACCCCTGTGTCCATAAAACAATACCCCATGTCACAGGAAGCCAGACTGGGGATCAAGCCCCACATACAGAG
GCTGTTGGACCAGGGAATACTGGTACCCTGCCAGTCCCCCTGGAACACGCCCCTGCTACCCGTTAAGAAACCAGGGACTA
ATGATTACAGGCCTGTCCAGGATCTGAGAGAAGTCAACAAGCAGGTGGAAGACATCCACCCCACCGTGCCCAACCCTTAC
AACCTCTTGAGCGGGCTCCCACCGTCCCACCAGTGGTACACTGTGCTTGATTTAAAGGATGCCTTTTTCTGCCTGAGACT
CCACCCCACCAGTCAGCCTCTCTTCGCCTTTGAGTGGAGAGATCCAGGGATGGGAATCTCAGGACAATTGACCTGGACCA
GACTCCCACAGGGTTTCAAAAACAGTCCCACCCTGTTTGACGAGGCTCTGCACAGAGACCTAGCCGACTTCCGGATCCAG
CACCCAGACTTGATCCTGTTGCAGTACGTAGATGACTTACTGCTGGCCGCGACTTCCGAGCCTGACTGCCAACAAGGTAC
TCGAGCCCTATTACAAACCTTAGGGGACCTCGGATACCGGGCCTCGGCCAAGAAAGCCCAACTTTGCCAGAAACAGGTCA
AGTATCTGGGGTATCTTCTAAAAGAGGGTCAGAGATGGCTGACTGAGGCCAGAAAAGAGACTGTGATGGGGCAGCCTACT
CCGAAGACCCCTCGACAACTAAGGGAGTTCCTAGGGACGGCAGGCTTCTGTCGCCTCTGGATCCCTGGGTTTGCGGAAAT
GGCAGCCCCCTTGTATCCTCTTACCAAAACGGGGACTCTGTTTAATTGGGGCCCAGACCAGCAAAAGGCCTATCAAGAAA
TCAAACAGGCCCTTCTAACTGCCCCCGCCCTGGGATTGCCAGATTTGACTAAACCCTTTGAACTCTTTGTCGACGAGAAG
CAGGGCTACGCCAAAGGCGTCCTAACGCAAAAACTGGGACCTTGGCGTCGGCCTGTGGCCTACCTGTCCAAAAAGCTAGA
CCCAGTGGCAGCTGGGTGGCCCCCTTGCCTACGGATGGTAGCAGCCATTGCCGTTTTGATAAAAGATGCAGGCAAACTAA
CTATGGGACAGCCGCTAGTCATCCTGGCCCCCCATGCAGTAGAGGCACTGGTCAAGCAACCCCCTGACCGCTGGCTATCC
AATGCCCGCATGACCCACTACCAGACAATGCTCCTAGACACTGACCGAGTTCAGTTCGGACCAGTGGTGGCCCTCAATCC
TGCCACCTTGCTCCCTCTACCGGAGAAAGGAGCCCCCCATGATTGCCTCGAGATCTTGGCTGAAACGCATGGAACCAGAC
CGGACCTCACCGACCAGCCCATCCCAGACGCCGACCACACCTGGTATACCGATGGGAGCAGCTTTTTGCAAGAAGGACAG
CGAAAAGCTGGGGCAGCAGTGACGACAGAGACCGAGGTAATCTGGGCGAGGGCCCTGCCAGCTGGAACGTCAGCCCAGCG
AGCCGAACTGATCGCACTCACCCAAGCCCTGAAAATGGCAGAAGGTAAGAAGCTAAATGTTTACACTGACAGCCGATATG
CTTTCGCCACGGCCCATGTCCATGGAGAAATCTATAGGAGGCGAGGGTTGCTGACCTCAGAGGGCAGAGAAATCAAAAAC
AAGAGCGAGATCCTGGCTTTACTGAAAGCTCTTTTCCTGCCTAAAAGACTCAGTATAATTCACTGCCCCGGGCATCAAAA
GGGAAACAGTGCTGAAGCCAGGGGCAACCGTATGGCAGACCAAGCGGCCCGAGAGGCAGCCATAAGGACATCTCCAGAAA
CTTCCACCCTCCTCATAGAGGACTCGACCCCGTATACGCCCTCCCATTTCCACTACACTGAAACAGATCTAAAGAGATTA
CGAGAACTGGGAGCCACCTATAATCAGATAAAAGGATATTGGGTTCTACAAGGCAAGCCGGTAATGCCCGATCAGTTTGT
GTTTGAACTATTAGACTCCTTACACAGGCTCACTCACCTCAGCCCTCAAAAGATGAAGGCACTCCTTGACAGAGAAGAAA
GCCCCTACTACATGTTAAACAGAGACAGAACTCTCCAGTATGTGGCAGAATCATGCACAGCTTGTGCTCAAGTGAATGCT
AGTAAAGCCAAGATCGGGGCAGGGGTACGAGTACGCGGACATCGACCAGGTACCCATTGGGAAATTGACTTTACTGAAGT
TAAGCCAGGACTGTACGGGTACAAGTACCTCCTAGTGTTCGTGGACACCTTCTCTGGCTGGGTAGAAGCCTTCCCAACTA
AACGTGAAACCGCCAAGGTGGTAACCAAGAAGCTATTAGAAGAAATATTCCCAAGATTCGGGATGCCACAGGTACTGGGT
TCTGACAATGGGCCTGCCTTCGTCTCCCAAGTAAGTCATTCGGTGGCCGATTTACTGGGGATTGATTGGAAGTTACATTG
TGCTTATAGACCCCAGAGTTCAGGTCAGGTAGAGAGAATGAATAGAACCATCAAGGAGACTTTAACCAAATTAACGCTTG
CAGCTGGCACTAGAGACTGGGTACTCCTACTCCCCCTGGCCCTCTACCGAGCCCGGAATACTCCGGGCCCCCATGGACTT
ACTCCGTATGAAATCCTGTATGGGGCACCCCCGCCCCTCGTCAATTTTCATGATCCTGAAATGTCAAAGTTAACTAATAG
TCCCTCTCTCCAAGCTCACTTACAGGCCCTCCAAGCAGTACAACGAGAGGTCTGGAAGCCACTGGCCGCTGCTTATCAGG
ACCAGCTGGATCAGCCAGTGATACCACACCCCTTCCGTGTCGGTGACGCCGTGTGGGTACGCCGGCACCAGACTAAGAAC
CTAGAACCTCGCTGGAAAGGACCCTACACCGTCCTACTGACCACCCCCACCGCTCTCAAAGTAGACGGCATCTCTGCGTG
GATACACGCCGCTCACGTAAAGGCGGCGACAACTCCTCCGGCCGGAACAGCATGGAAGGTCCAGCGTTCTCAAAATCCCT
TAAAGATAAGATTAACCCGTGGGGCCCCCTGATAGTTATGGGGATCTTAGTGGGGGCAGGAGCCTCGGGACAACGTGACA
GCCCTCACCAGGTCTTCAATGTTACTTGGAGAGTTACCAACCTAATGACAGGACAAACAGCTAACGCCACCTCCCTCCTG
GGGACGATGACAGACACCTTCCCTAAACTATATTTTGACCTGTGTGATTTAGTAGGAGACTACTGGGACGACCCAGATGG
AGATGGTTGCCGCTCTCCCGGAAGAAGAAAAAGGACAAGACTGTATGACTTCTATGTTTGCTCCGGTCATACTGTACCAA
TAGGGTGTGGAGGGCCGGGAGAGGGCTACTGTGGCAAATGGGGATGTGAGACCACTGGACAGGCATACTGGAAGCCCTCA
TCATCATGGGACCTAATTTCCCTTAAGCGAGGAAACACTCCTAAGGATCAGGGCCCCTGTTATGATTCCTCGGTCTCCAG
TGGCGTCCAGGGTGCTACACCGGGGGGTCGATGCAACCCCCTAGTCTTAGAATTCACTGACGCGGGTAAAAAGGCCAGCT
GGGATGCCCCCAAAGTTTGGGGACTAAGACTCTATCGATCCACAAGGGCCGACCCGGTGACCCGGTTCTCTTTGACCCGC
CAGGTCCTCAATGTAGGACCCCGCGTCCCCATTGGGCCTAATCCCGTGATCACTGACCAGCTACCCCCATCCCAACCCGT
GCAGATCATGCTCCCCAGGCCTCCTCATCATCCTCCTTCAGGCACGGTCTCTATGGTACCTGGGGCTCCCCCGCCTTCTC
AACAGCCTGGGACAGGAGACAGGCTACTAAATCTGGTAGACGGAGCCTACCAAGCACTCAACCTCACCAGTCCTGACAAA
ACCCAAGAGTGCTGGTTGTGTCTGGTATCGGGACCCCCCTACTACGAAGGGGTTGCCGTCCTAGGTACCTACTCCAACCA
TACCTCTGCCCCAGCTAACTGCTCCGTGGCCTCCCAACACAAGCTGACCCTGTCCGAAGTGACCGGACAGGGACTCTGCG
TAGGAGCAGTTCCCAAAACCCATCAGGCCCTGTGTAATACCACCCAGAAGACGAGCGACGGGTCCTACTATCTGGCTGCT
CCCGCCGGGACCATCTGGGCTTGCAACACCGGGCTCACTCCCTGCCTATCTACTACTGTACTCAACCTCACCACCGATTA

CTGTGTCCTGGTTGAGCTCTGGCCAAAGGTGACCTACCACTCCCCTGGTTATGTTTATGGCCAGTTTGAGAGAAAAACCA
AATATAAAAGGGAGCCGGTGTCATTAACTCTGGCCCTGCTGTTGGGAGGACTTACTATGGGCGGCATAGCTGCAGGAGTA
GGAACAGGGACTACAGCCCTAGTGGCCACCAAACAATTCGAGCAGCTCCAGGCAGCCATACATACAGACCTTGGGGCCTT
AGAAAAATCAGTCAGTGCCCTAGAAAAGTCTCTGACCTCGTTGTCTGAGGTGGTCCTACAGAACCGGAGAGGATTAGATC
TGCTGTTCCTAAAAGAAGGAGGATTATGTGCTGCCCTAAAAGAAGAATGCTGTTTCTACGCGGACCACACTGGCGTAGTA
AGGGATAGCATGGCTAAGCTAAGAGAGAGACTAAACCAGAGACAAAAATTGTTCGAATCAGGACAAGGGTGGTTTGAGGG
ACTGTTTAACAGGTCCCCATGGTTCACGACCCTGATATCCACCATTATGGGCCCTCTGATAGTACTTTTATTAATCCTAC
TCTTCGGACCCTGTATTCTCAACCGCTTGGTCCAGTTTGTAAAAGACAGAATTTCGGTGGTGCAGGCCCTGGTTCTGACC
CAACAGTATCACCAACTCAAATCAATAGATCCAGAAGCAGTGGAATCACGTGAATAAAAGATTTTATTCAGTTTCCAGAA
AGAGGGGGGAATGAAAGACCCCACCATAAGGCTTAGCAAGCTAGCTGCAGTAACGCCATTTTGCAAGGCATGAAAAAGTA
CCAGAGCTGAGTTCTCAAAAGTTACAAGGAAGTTCAGTTAAAGATTAACAGTTAAAAATCAAGGCTGAATAATACTAGGA
CAAGGGCCAAAAAGGGTATATCGGTGGTCAAGCGCCTGGGCCCCGGCTCAGGGCCAAGAACCGATGGTACCCACCTGGGC
CCCGGCTCAGGGCCAAGAACAGATGGTACCCAGATAAAGCGGAACCAGCAACAGTTTCTGAAAAAGTCCCACCTCAGTTT
CAGGTTCCCCAAATGACCAGGAAATACCCCAAGCCTTGATTTAAACTAACCACTCAGCTCGCTTCTCGCTTCTGTACCCA
CGCTTTTTGCTCCCCAGCCCCAGCCCTATAAAAAGGGTAAAAACCCCACACTCGGCGCGCCAGTCCTCCGATAGACTGAG
TCGCCCGGGTACCCGTGTTCCCAATAAAGCCTCTTGCTGATTACATCCGAATCGTGGTCTCGCTGATCCTTGGGAGGGTC
TCCTCAGATTGATTGACCACCCACCTCGGGGGTCTTTCA
>Xmv9
TGAAAGACCCCACCATAAGGCTTAGCAAGCTAGCTGCAGTAACGCCATTTTGCAAGGCATGAAAAAGTACCAGAGCTGAG
TTCTCAAAAGTTACAAGGAAGTTCAGTTAAAGATTAACAGTTAAAAATCAAGGCTGAATAATACTAGGACAAGGGCCAAA
CAGGATATCGGTGGTCAAGCGCCTGGGCCCCGGCTCAGGGCCAAGAACCGATGGTTCCCACCTGGGCCCCGGCTCAGGGC
CAAGAACAGGTGGTACCCAGATGAAGCGGAACCAGCAACAGTTTCTGAAAAAGTCCCAGGTCCCCAAATGACCAGGAAA
TATCCCAAGCCTTGATTTGAACTAACCACTCAGCTCGCTTCTCGCTTCTGTGCCCACGCTTTTTGCTCCCCAGCCCCAGC
CCTATAAAAAGGGTAAAAATTCCACACTCGGCGCGCCAGTCCTCCGATAGACTGAGTCGCCCGGGTACCCGTGTTCCCAA
TAAAAGCCTCTTGCTGATTGCATCCGAATCGTGGACTCGCTGATTCTTGGGAGGGTCTCCTCAGATTGATTGACTACCCA
CCTCGGGAGTCTTTCATTTGGAGGTCCCACCGAGATTAGGAGACCCCTGCCCAAGGACCACCGACCCCCGCCGGGAGGTA
AGCTGGCCAGCAGTCGTTTCGTGTCTGTCTCCGTTTCCGTGCGTGTTTGTGCCGGCACCTAATGTTTGCGCCTGCGTCTG
TACTAGTTGGCTAACTAGATCTGTATCTGGCGAGTCCGCGGAAGAACTGACGAGTTCGTATTCCCGGCCGCAGCCCTGAG
AGACGTCTCAGAGGCATCGGGGGCCATCTCTGTGGCCCAATCTGTATCTGAGAACCCGGCTCTTTGACCAAGGGATACGT
GGTTCTGTTGGGCGGCGAGGGGCCGAAACGCTCCCCTCCCCCATCTGAATTTTTGCTTTCGGTTTTCCGCCGAAACCGCG
CCGCGCGTCTTGTCTGTCTCTGTGTTGTTTTGTCATTGTTTTGTCATTTGTCGGTTCGTTATTGTTTTAGACCGTTTCTA
AAAATATGGGGCAGACCGTAACCACTCCTTTGAGCCTGACCCTAGAACACTGGGGAGACGTCCAGCGCATTGCGTCCAAC
CAGTCCGTGGACGTCAAGAAGAGACATTGGGTCACCTTCTGCTCTGCCGAGTGGCCAACTTTCGATGTGGGGTGGCCGCA
AGATGGTACTTTTAATTTGGACATTATTTTACAGGTTAAATCTAAGGTGTTCTCTCCCGGTCCCCACGGACACCCGGATC
AGGTCCCATACATTGTCACCTGGGAGGCACTTGCCTATGACCCCCCTCCGTGGGTCAAACCGTTTGTCTCTCCAAAACCC
CCTCCTTTACCGACAGCTCCCGTCCTCCCGCCCGGTCCTTCTGCGCAACCTCCGTCCCGATCTGCCCTTTACCCTGCCCT
TACCCCCTCTATAAAGCCCAAACCTCCTAAGCCCCAGGTTCTCCCTGATGGCGGCGGACCTCTCATTGACCTTCTCACAG
AGGACCCCCCGCCGTACGGAGCACAACCTTCCTCCTCTGCCAGAGGGAACGATGAAAAAGAGGCGGCCGCCACCTCCGAG
GCTTCCCCCCCCTCTCCCATGGTGTCTCGACTGCGGGGAAGGAGGGACCCTCCCGCAGCGGACTCCACCTCCTCCCAGGC
ATTCCCACTCCGCATGGGGGGAGATGGCCAGCTTCAGTATTGGCCGTTTTCCTCTTCGGACTTATACAATTGGAAAAATA
ATAACCCTTCCTTTTCTGAAGACCCAGGTAAATTGACGGCTTTGATTGAGTCCGTCCTCATCACCCACCAGCCCACCTGG
GACGACTGTCAGCAGTTGTTGGGGACCCTGCTGACCGGAGAAGAAAAGCAGCGGGTGCTCCTAGAGGCTAGAAAGGCAGT
CCGGGGCAATGATGGACGCCCCACTCAGTTGCCTAATGAAGTCAATGCTGCTTTTCCCCTTGAACGCCCCGATTGGGATC
ACACCACTACAGAAGGTAGGAACCACCTAGTCCTCTATCGCCAGTTGCTCTTAGCGGGTCTCCAAAACGCGGGCAGAAGC
CCCACCAATTTGGCCAAGGTAAAAGGGATAACCCAGGGACCTAATGAGTCTCCCTCAGCCTTTTTAGAGAGACTCAAGGA
GGCATATTGCAGGTACACTCCTTATGACCCTGAGGACCCAGGGCAAGAAACCAATGTGTCTATGTCATTCATCTGGCAGT
CTGCCCCAGATATCGGGCGAAAGTTAGAGCGGTTAGAAGATTTAAAGAGCAAGACATTAGGAGATTTAGTGAGGGAAGCT
GAAAAGATCTTTAATAAGCGAGAAACCCCGGAAGAAAGAGAGGAACGTATCAGGAGAGAAACAGAGGAAAAGGAAGAACG
CCGTAGGGCAGAGGATGAGCAGAGAGAGAAAGAAAGGGACCGCAGGAGACATAGAGAGATGAGCAAGCTCTTGGCCACTG
TGGTTAGTGGACAGAGACAGGATAGACAGGGGGGAGAGCGAAGGAGGCCCCAACTTGATAAGGACCAATGCGCCTACTGC
AAAGAAAAGGGACACTGGGCTAAGGACTGCCCAAAGAAGCCACGAGGGCCCCGAGGACCGAGGCCCCAGACCTCCCTCCT
GACCTTAGGTGACTAGGGAGGTCAGGGTCAGGAGCCCCCCCCTGAACCCAGGATAACCCTCAAAGTCGGGGGGCAACCCG
TCACCTTCCTGGTAGATACTGGGGCCCAACACTCCGTGCTGACCCAAAATCCTGGACCCCTAAGTGACAAGTCTGCCTGG
GTCCAAGGGGCTACTGGAGGAAAGCGGTATCGCTGGACCACGGATCGCAAAGTACACCTAGCCACCGGTAAGGTCACCCA
CTCTTTCCTCCATGTACCAGACTGCCCCTATCCTCTGCTAGGAAGAGACTTGCTGACTAAACTCAAAGCCCAGATCCACT
TCGAGGGATCAGGAGCTCAGGTTGTGGGACCAATGGGACAGCCCCTGCAAGTGCTGACCCTAAACATAGAGGATGAGTAT
CGGCTACATGAGACCTCAACAGAGCCGGATGTTTCTCTAGGGTCCACCTGGCTTTCTGATTTTCCCCAGGCCTGGGCGGA

AACTGGGGGCATGGGACTGGCAGTTCGCCAAGCGCCTCTGATTATACCTCTGAAGGCAACCTCCACCCCGGTGTCCATAA
AACAATACCCCATGTCACAGGAAGCCAGACTGGGGATCAAGCCCCACATACAGAGGCTGTTGGACCAGGGAATACTGGTA
CCCTGCCAGTCCCCCTGGAATACACCCCTGCTACCCGTTAAGAAACCAGGGACTAACGATTATAGGCCTGTCCAGGATCT
GAGAGAAGTCAACAAGCGGGTGGAAGACATCCACCCCACCGTGCCCAATCCTTACAACCTCTTAAGTGGGCTCCCACCAT
CCCACCAGTGGTATACTGTGCTTGATTTAAAGGATGCCTTTTTCTGCCTGAGACTCCACCCCACCAGTCAGCCTCTCTTC
GCCTTTGAGTGGAGAGATCCAGAAATGGGAATCTCAGGACAATTGACCTGGACCAGACTCCCACAGGGTTTCAAAAACAG
TCCCACCCTGTTTGACGAGGCTCTGCACAGAGACCTAGCCGACTTCCGGATCCAGCACCCAGACTTGATCCTGTTGCAGT
ACGTAGATGACTTACTACTGGCCGCGACTTCCGAGCCTGACTGCCAACAAGGTACTCGAGCCCTATTACAAACCTTAGGG
GACCTCGGATACCGGGCCTCGGCCAAGAAAGCCCAACTTTGCCAGAAACAGGTCAAGTATCTGGGGTATCTTCTAAAAGA
GGGTCAGAGATGGCTGACTGAGGCCAGAAAAGAGACTGTGATGGGGCAGCCTACTCCGAAGACCCCTCGACAACTAAGGG
AGTTCCTAGGGACGGCAGGCTTCTGTCGCCTCTGGATCCCTGGGTTTGCGGAAATGGCAGCCCCCTTGTATCCTCTTACC
AAAACGGGGACTCTGTTTAATTGGGGCCCAGACCAGCAAAAGGCCTATCAAGAAATCAAACAGGCCCTTCTAACTGCCCC
CGCCCTGGGATTGCCAGATTTGACTAAACCCTTTGAACTCTTTGTCGACGAGAAGCAGGGCTACGCCAAAGGCGTCCTAA
CGCAAAAACTGGGACCTTGGCGTCGGCCTGTGGCCTACCTGTCCAAAAAGCTAGACCCAGTGGCAGCTGGGTGGCCCCCT
TGCCTACGGATGGTAGCAGCCATTGCCGTTCTGATAAAAGATGCAGGCAAGCTAACTATGGGACAGCCGCTAGTCATCCT
GGCCCCCCATGCAGTAGAGGCACTGGTCAAGCAACCCCCTGACCGCTGGCTATCCAATGCCCGCATGACCCACTACCAGG
CAATGCTCCTAGACACTGACCGAGTTCAGTTCGGACCAGTGGTGGCCCTCAATCCTGCCACCTTGCTCCCTCTACCGGAG
AAAGGAGCCCCCCATGATTGCCTCGAGGTCTTGGCTGAAACGCATGGAACCAGACCGGACCTCACCGACCAGCCCATCCC
AGACGCCGACCACACCTGGTATACCGATGGGAGCAGCTTTTTGCAAGAAGGACAGCGAAAAGCTGGGGCAGCAGTGACGA
CAGAGACCGAGGTAATCTGGGCGAGGGCCCTGCCAGCTGGAACGTCAGCCCAGCGAGCCGAACTGATCGCACTCACCCAA
GCCCTGAAAATGGCAGAAGGTAAGAAGCTAAATGTTTATACTGATAGCCGCTATGCCTTCGCTACGGCCCATGTTCATGG
GGAAATATATAGGAGACGGGGGTTGCTGACCTCAGAAGGCAAGGAAATCAAGAACAAAAGCGAGATCCTAGCCTTGCTGA
AAGCCCTCTTTTTGCCAAAGAGGCTCAGTATTATCCATTGCCCGGGACACCAAAAAGGGAACAGTGCTGAAGCCAGGGGC
AACCGAATGGCGGATCAAGCAGCCAGGGAGGCAGCCATGAGAGAGAGCGCAGAAACGTCCACGCTCTTAATAGAGGATTC
GTCCCCGTATACTCCCAGCTACTTCCACTACACAGAGACTGACAGAAAAGACCTTCTGAGACTGGGAGCCGTATACGATG
AGGACAAAAAGTATTGGGTCCTGCAGGGAAAACCTGTGATGCCTGACCAATTCACCTTTGAGCTCTTAGATTCCCTACAC
CGGCTCACCCACCTCAGCTATCAGAAGATGAAGGCACTCCTTGACCGGGAAGAGAGCCCCTACTACATGTTAAATCGGGA
TAAAACTCTCCAGCACGTAACGGAATCCTGTACAGCTTGTGCTCAAGTAAATGCTAGTAAAGCCAAAATCGGAGCAAGGG
TGCGAGCGCGGGGACACCGACCCGGCACCCATTGGGAAATCGATTTTACCGAAGTTAAGCCAGGGCTGTATGGATATAAG
TATCTCCTGGTGTTTGTGGACACATTCTCTGGCTGGGTAGAAGCATTTCCAACCAAGCGTGAAACTGCCAGGGTTGTGAC
CAAGAAGCTGCTTGAAGAAATTTTTCCAAGATTCGGGATGCCCCAGGTATTGGGGACAGATAATGGGCCTGCCTTCATCT
CCCAGGTAAGTCAGACAGTGGCCGATTTGTTGGGGATCGATTGGAAACTGCATTGTGCTTATAGACCCCAGAGTTCAGGT
CAGGTAGAAAGAATGAATAGAACAATCAAGGAGACTTTGACCAAATTAACGCTTGCAGCTGGCACTAGAGACTGGGTACT
CCTACTCCCCCTGGCCCTCTACCGAGCCCGGAATACTCCGGGCCCACATGGACTCACTCCGTATGAAATTCTGTATGGGG
CACCCACGCCCCTTGTCAATTTTCATGATCCTGAAATGTCAAAGTTAACTAATAGTCCCTCTCTCCAAGCTCACTTACAG
GCCCTCCAAGCAGTACAACGAGAGGTCTGGAAGCCGCTGGCCGCTGCTTATCAGGACCAGCTGGATCAGCCAGTGATACC
ACACCCCTTCCGTGTCGGTGACGCCGTGTGGGTGCGCCGGCATCAGACTAAGAACCTAGAACCTCGCTGGAAAGGACCCT
ACACCGTCCTACTAACCACCCCCACCGCTCTCAAAGTAGACGGCATCTCTGCGTGGATACACGCCGCTCACGTAAAGGCG
GCGACAACTCCTCCGGCCGGAACAGCATGGAAGGTCCAGCGTTCTCAAAATCCCTTAAAGATAAGACTAACCCGTGGGGC
CCCCTGATAGTCATGGGGATCTTAGTGGGGGCAGGAGCCTCGGGACAACGTGACAGCCCTCACCAGGTCTTCAATGTTAC
TTGGAAAGTTACCAACCTAATGACAGGACAAACAGCTAACGCCACCTCCCTTCTGGGGACGGTGACAGACACCTTCCCTA
AACTATATTTTGACCTGTGTGATTTAGTAGGAGACTACTGGGACGACCCAGATGGAGTTGGTTGCCGCTCTCCCGGAAGA
AGAAAAAGGACAAGACTGTATGACTTCTATGTTTGCCCCGGTCATACTGTACCAATAGGGTGTGGAGGGCCGGGAGAGGG
CTACTGTGGCAAATGGGGATGTGAGACCACTGGACAGGCATACTGGAAGCCCTCATCATCATGGGACCTAATTTCCCTTA
AGCGAGGAAACACTCCTAAGGATCAGGGCCCCTGTTACGATTCCTCGGTCTCCAGTGGCGTCCAGGGTGCTACACCGGGG
GGTCGATGCAACCCCCTAGTCTTAGAATTCACTGACGCGGGTAAAAGGCCAGCTGGGATGCCCCCAAAGTTTGGGGACT
AAGACTCTATCGATCCACAAGGGCCGACCCGGTGACCCGGTTCTCTTTGACCCGCCAGGTCCTCAATGTAGGACCCCGCG
TCCCCATTGGGCCTAATCCCGTGATCACTGACCAGCTACCCCCATCCCAACCCGTGCAGATCATGCTCCCCAGGCCTCCT
CATCATCCTCCTCCAGGCACGGTCTCTATGGTACCTGGGGCTCCCCCGCCTTCTCAACAGCCTGGGACAGGAGACAGGCT
ACTAAGTCTGGTAGACGGAGCCTACCAAGCACTCAACCTCACCAGTCCTGACAAAACTCAAGAGTGCTGGTTGTGTCTGG
TATCGGGACCCCCCTACTACGAAGGGGTTGCCGTCCTAGGTACCTACTCCAACCATACCTCTGCCCCAGCTAACTGCTCC
GTGGCCTCCCAACACAAGCTGACCCTGTCCGAAGTGACCGGACAGGGACTCTGCGTAGGAGCAGTTCCCAAAACCCATCA
GGCCCTGTGTAATACCACCCAGAAGACGAACGACGGGTCCTACTATCTGGCTGCTCCCGCCGGGACCATCTGGGCTTGCA
ACACCGGGCTCACTCCCTGCCTATCTACTACTGTACTTAACCTCACCACCGATTACTGTGTCCTGGTTGAGCTCTGGCCA
AAGGTGACCTACCACTCCCCTGGTTATGTGTATGGCCAGTTTGAGAGAAAAACCAAGTATCAAAGAGAGCCGGTGTCATT
AACTCTGGCCCTACTGTTGGGAGGACTTACTATGGGCGGCATAGCTGCAGGAGTAGGAACAGGGACTACAGCCCTAGTGG
CCACCAAACAATTCGAGCAGCTCCAGGCAGCCATACATACAGACCTTGGGGCCTTAGAAAAATCAGTCAGTGCCCTAGAA

AAGTCTCTGACCTCGTTGTCTGAAGTGGTCCTACAGAACCGGAGAGGATTAGATCTGCTGTTCCTAAAAGAAGGAGGATT
ATGTGCTGCCCTAAAAGAAGAATGCTGTTTCTACGCGGACCACACTGGCGTAGTAAGGGATAGCATGGCTAAGCTAAGAG
AGAGACTAAACCAGAGACAAAAATTGTTCGAATCAGGACAAGGGTGGTTTGAGGGACTGTTTAACAGGTCCCCATGGTTC
ACGACCTTGATATCCACCATTATGGGCCCCTTGATAATACTTTTATTAATCCTACTCCTCGGACCCTGTATTCTCAACCG
ATTGGTCCAGTTTGTAAAAGACAGAATTTCGGTGGTGCAGGCCCTGGTTCTGACCCAACAGTATCACCAACTCAAATCAA
TAGAGCCAGAAGCAGTGGAATCACGTGAATAAAAGATTTTATTCAGTTTCCAGAAAGAGGGGGGAATGAAAGACCCCACC
ATAAGGCTTAGCAAGCTAGCTGCAGTAACGCCATTTTGCAAGGCATGAAAAAGTACCAGAGCTGAGTTCTCAAAAGTTAC
AAGGAAGTTCAGTTAAAGATTAACAGTTAAAAATCAAGGCTGAATAATACTAGGACAAGGGCCAAACAGGATATCGGTGG
TCAAGCGCCTGGGCCCCGGCTCAGGGCCAAGAACCGATGGTTCCCACCTGGGCCCCGGCTCAGGGCCAAGAACAGGTGGT
ACCCAGATGAAGCGGAACCAGCAACAGTTTCTGAAAAAGTCCCAGGTCCCCCAAATGACCAGGAAATATCCCAAGCCTTG
ATTTGAACTAACCACTCAGCTCGCTTCTCGCTTCTGTGCCCACGCTTTTTGCTCCCCAGCCCCAGCCCTATAAAAAGGGT
AAAAATTCCACACTCGGCGCGCCAGTCCTCCGATAGACTGAGTCGCCCGGGTACCCGTGTTCCCAATAAAAGCCTCTTGC
TGATTGCATCCGAATCGTGGACTCGCTGATTCTTGGGAGGGTCTCCTCAGATTGATTGACTACCCACCTCGGGAGTCTTT
CA
