# Supplementary material for: Role of APOBEC3 in Genetic Diversity among Endogenous Murine Leukemia Viruses
Source: PLoS Genet. 2007 Oct 26;3(10):e183. doi: 10.1371/journal.pgen.0030183 (PMC2041998; doi:10.1371/journal.pgen.0030183)
Supplement: Figure S2 — (14 KB PDF) [file pgen.0030183.sg002.pdf]

Figure S2

Proviral junctions and target duplications (TSDs) from http://genome.ucsc.edu/

Mouse genome (mm8, freeze date Feb. 2006)

Locus		5'TSD		3'TSD	
Pmv1	AACACT	**ATAC**	TGAAAGACCCCACCA---//---GTCGGGGGTCTTTCA	**ATAC**	CTACCC
Pmv10	GGTGGG	**AGGT**	TGAAAGACCCCACCA---//---GTCGGGGGTCTTTCA	**AGGT**	GAGCCC
Pmv11	GTTCCA	**CACC**	TGAAAGACCCCACCA---//---GTCGGGGGTCTTTCA	**CACC**	AACGGC
Pmv12	AATACC	**AGGG**	TGAAAGACCCCACCA---//---GTCGGGGGTCTTTCA	**AGGG**	TTAACT
Pmv13	GAGAAG	**GATG**	TGAAAGACCCCACCA---//---GTCGGGGGTCTTTCA	**GATG**	AAGCCA
Pmv14	ATTGTT	**CTGG**	TGAAAGACCCCACCA---//---GTCGGGGGTCTTTCA	**CTGG**	AAGACA
Pmv15	AAAAAC	**CATG**	TGAAAGACCCCACCA---//---GTCGGGGGTCTTTCA	**CATG**	TTCTGG
Pmv16	AAGGGT	**GCCT**	TGAAAGACCCCACCA---//---GTCGGGGGTCTTTCA	**GCCT**	TACAAC
Pmv17	ACAAAC	**AAAC**	TGAAAGACCCCACCA---//---GTCGGGGGTCTTTCA	**AAAC**	AAACAA
Pmv18	GTATTA	**ATTT**	TGAAAGACCCCACCA---//---CTCGGGGGTCTTTCA	**ATTT**	CTTACA
Pmv19	CTGGAA	**AAAC**	TGAAAGACCCCACCA---//---GTCGGGGGTCTTTCA	**AAAC**	AAACAA
Pmv2	TAAATG	**GTTC**	TGAAAGACCCCACCA---//---GTCGGGGGTCTTTCA	**GTTC**	ATGATT
Pmv20	AGCCTA	**CCTG**	TGAAAGACCCCACCA---//---GTCGGGGGTCTTTCA	**CCTG**	GTCTAC
Pmv21	CAGGAT	**GTTG**	TGAAAGACCCCACCA---//---GTCGGGGGTCTTTCA	**GTTG**	CAGAAC
Pmv22	TTATTT	**ATAG**	TGAAAGACCCCACCA---//---CTCGGGGGTCTTTCA	**ATAG**	TTTTTA
Pmv23	TGTTTA	**CACC**	TGAAAGACCCCACCA---//---GTCGGGGGTCTTTCA	**CACC**	CATGTC
Pmv24	CACTAG	**TTTG**	TGAAAGACCCCACCA---//---GTCGGGGGTCTTTCA	**TTTG**	TTCAAC
Pmv4	AAAATT	**ATGA**	TGAAAGACCCCACCA---//---GTCGGGGGTCTTTCA	**ATGA**	TAGATC
Pmv5	GCACAG	**CCAC**	TGAAAGACCCCACCA---//---GTCGGGGGTCTTTCA	**CCAC**	CACAAT
Pmv6	ATCTCA	**TGAA**	TGAAAGACCCCACCA---//---CTCGGGGGTCTTTCA	**GAGT**	ATGCCT
Pmv7	GTGGTG	**GTGT**	TGAAAGACCCCACCA---//---GTCGGGGGTCTTTCA	**GTGT**	AAGGCT
Pmv8	CATGCT	**GTAC**	TGAAAGACCCCACCA---//---GTCGGGGGTCTTTCA	**GTAC**	CAATGA
Pmv9	GTCTTA	**GTGG**	TGAAAGACCCCACCA---//---GTCGGGGGTCTTTCA	**GTGG**	GAGGAA
Mpmv1	GGCATC	**CCTG**	TGAAAGACCCCACCA---//---CTGGGGGGTCTTTCA	**CCTG**	CCTGAC
Mpmv10	TTTATC	**ACTT**	TGAAAGACCCCACCA---//---CTGGGGGGTCTTTCA	**ACTT**	TCAGTT
Mpmv11	CTCTGT	**AGAC**	TGAAAGACCCCACCA---//---CAGGGGGGTCTTTCA	**AGAC**	CAGGCT
Mpmv12	TTATTT	**CTGC**	TGAAAGACCCCACCA---//---GGGGGGGGTCTTTCA	**TTGC**	CAGTGG
Mpmv13	TCACGA	**ATAC**	TGAAAGACCCCACCA---//---CTGGGGGGTCTTTCA	**ATAC**	TAGCGG
Mpmv2	CAAGTC	**AGGG**	TGAAAGACCCCACCA---//---GGGGGGGGTCTTTCA	**TTGG**	AACATT
Mpmv3	CTAAAC	**ATTG**	TGAAAGACCCCACCA---//---CTGGGGGGTCTTTCA	**ATTG**	TTTTTA
Mpmv4	GCACAC	**ACAC**	TGAAAGACCCCACCA---//---CTGGGGGGTCTTTCA	**ACAC**	TCCACC
Mpmv5	TGGATC	**ACAG**	TGAAAGACCCCACCA---//---CTGGGGGGTCTTTCA	**TTAG**	GGCCCC
Mpmv6	TGGGCA	**ACAC**	TGAAAGACCCCACCA---//---GGGGGGGGTCTTTCA	**ACAC**	TATCCC
Mpmv7	ATGTAT	**ATAT**	TAAAAGACCCCACCA---//---CTGGGGGGTCTTTCA	**TTAT**	GTGTGT
Mpmv8	CTAAG	**GAAAC**	TGAAAGACCCCACCA---//---CTGGGGGGTCTTTCA	**GAAAC**	AAAAA
Mpmv9	CTGTTC	**ATGT**	TGAAAGACCCCACCA---//---TGGGGGGGTCTTTCA	**ATGT**	CTCCTG
Xmv10	TGAGTT	**CCAG**	TGAAAGACCCCACCA---//---CTCGGGGGTCTTTCA	**CCAG**	GACAGC
Xmv12	CAGCCT	**GGTC**	TGAAAGACCCCACCA---//---CTCGGGGGTCTTTCA	**GTCT**	ACAGAG
Xmv13	TAAGAT	**GTAC**	TGAAAGACCCCACCA---//---CTCGGGGGTCTTTCA	**GTAC**	ACTATA
Xmv15	GAGCTG	**CTGG**	TGAAAGACCCCACCA---//---CTCGGGGGTCTTTCA	**CTGG**	AGTCTC
Xmv16	GGGGTT	**GGAA**	TGAAAGACCCCACCA---//---CTCGGAGGTCTTTCA	**GGGG**	GTGGGG
Xmv17	GTTGTA	**TTAA**	TGAAAGACCCCACCA---//---CTCGGGGGTCTTTCA	**TTAG**	TCAGGG
Xmv18	AGTCTT	**CTTG**	TGAAAGACCCCACCA---//---CTCGGGGGTCTTTCA	**CTTG**	CTAGTA
Xmv19	GAGAAA	**GTAT**	TGAAAGACCCCACCA---//---CTCGGGGGTCTTTCA	**GTAT**	GTCTCT
Xmv41	GCAGTG	**GTGA**	TGAAAGACCCCACCA---//---CTCGGGGGTCTTTCA	**GTGA**	CACATG
Xmv42	GAGGCA	**GGAG**	TGAAAGACCCCATCA---//---GTCGGGGGTCTTTCA	**GGAG**	GAGGCT
Xmv43	TTCATA	**AACC**	TGAAAGACCCCACCA---//---CTCGGGGGTCTTTCA	**AACC**	TATCCT
Xmv8	GGTCAC	**ATGC**	TGAAAGACCCCACCA---//---CTCGGGGGTCTTTCA	**ATGC**	TACAGG
Xmv9	AGGGTA	**TTGG**	TGAAAGACCCCACCA---//---CTCGGGAGTCTTTCA	**TTGG**	TATTCA
