# Supplementary material for: Role of APOBEC3 in Genetic Diversity among Endogenous Murine Leukemia Viruses
Source: PLoS Genet. 2007 Oct 26;3(10):e183. doi: 10.1371/journal.pgen.0030183 (PMC2041998; doi:10.1371/journal.pgen.0030183)
Supplement: Figure S3 — (142 KB PDF) [file pgen.0030183.sg003.pdf]

Pmv (concatenated *gag, pol* and *env)*

```
PMV_CONS      ATGGGACAGACCGTAACTACCCCTCTGAGTTTAACCTTGCAGCACTGGGGAGATGTCCAGCGCATTGCATCCAACCAGTC
Pmv1          atgggacagaccgtaactacccctctgagtttaaccttgcagcactggggagatgtccagcgcattgcatccaaccagtc
Pmv10         atgggacagaccgtaactacccctctgagtttaaccttgcagcactggggagatgtccagcgcattgcatccaaccagtc
Pmv11         atgggacagaccgtaactacccctctgagtttaaccttgcagcactggggagatgtccagcgcattgcatccaaccagtc
Pmv12         atgggacagaccgtaactacccctctgagtttaaccttgcagcactggggagatgtccagcgcattgcatccaaccagtc
Pmv13         atgggacagaccgtaactacccctctgagtttaaccttgcagcactggggagatgtccagcgcattgcatccaaccagtc
Pmv14         atgggacagaccgtaactacccctctgagtttaaccttgcagcactggggagatgtccagcgcattgcatccaaccagtc
Pmv15         atgggacagaccgtaactacccctctgagtttaaccttgcagcactggggagatgtccagcgcattgcatccaaccagtc
Pmv16         atgggacagaccgtaactacccctctgagtttaaccttgcagcactggggagatgtccagcgcattgcatccaaccagtc
Pmv17         atgggacagaccgtaactacccctctgagtttaaccttgcagcactggggagatgtccagcgcattgcatccaaccagtc
Pmv18         atgggacagaccgtaactacccctctgagtttaaccttgcagcactggggagatgtccagcgcattgcatccaaccagtc
Pmv19         atgggacagaccgtaactacccctctgagtttaaccttgcagcactggggagatgtccagcgcattgcatccaaccagtc
Pmv2          atgggacagaccgtaactacccctctgagtttaaccttgcagcactggggagatgtccagcgcattgcatccaaccagtc
Pmv20         atgggacagaccgtaactacccctctgagtttaaccttgcagcactggggGgatgtccagcgcattgcatccaaccagtc
Pmv21         atgggacagaccgtaactacccctctgagtttaaccttgcagcactggggCgCtgtccagcgcattgcatccaaccagtc
Pmv22         atgggacagaccgtaactacccctctgagtttaaccttgcagcactggggagatgtccagcgcattgcatccaaccagtc
Pmv23         atgggacagaccgtaactacccctctgagtttaaccttgcagcactggggagatgtccagcgcattgcatccaaccagtc
Pmv24         atgggacagaccgtaactacccctctgagtttaaccttgcagcactggggGgatgtccagcgcattgcatccaaccagtc
Pmv4          atgggacagaccgtaactacccctctgagtttaaccttgcagcactggggagatgtccagcgcattgcatccaaccagtc
Pmv5          atgggacagaccgtaactacccctctgagtttaaccttgcagcactggggagatgtccagcgcattgcatccaaccagtc
Pmv6          atgggacagaccgtaactacccctctgagtttaaccttgcagcactggggagatgtccagcgcattgcatccaaccagtc
Pmv7          atgggacagaccgtaactacccctctgagtttaaccttgcagcactggggagatgtccagcgcattgcatccaaccagtc
Pmv8          atgggacagaccgtaactacccctctgagtttaaccttgcagcactggggagatgtccagcgcattgcatccaaccagtc
Pmv9          atgggacagaccgtaactacccctctgagtttaaccttgcagcactggggagatgtccagcgcattgcatccaaccagtc

PMV_CONS      TGTGGATGTCAGGAAGAGGCGCTGGATTACCTTCTGTTCCGCTGAATGGCCAACTTTCAATGTGGGATGGCCTCAGGATG
Pmv1          tgtggatgtcaAgaagaggcgctggattaccttctgttccgctgaatggccaactttcaatgtgggatggcctcaggatg
Pmv10         tgtggatgtcaggaagaggcgctggattaccttctgttccgctgaatggccaactttcaatgtgggatggcctcaggatg
Pmv11         tgtggatgtcaAgaagaggcgctggattaccttctgttccgctgaatggccaactttcaatgtgggatggcctcaggatg
Pmv12         tgtggatgtcaggaagaggcgctAgattaccttctgttccgcCgaatggccaactttcaatgtgggatggcctcaggatg
Pmv13         tgtggatgtcaggaagaggcgctggattaccttctgttccgctgaatggccaactttcaatgtgggatggcctcaggatg
Pmv14         tgtggatgtcaggaagaggcgctggattaccttctgttccgctgaatggccaactttcaatgtgggatggcctcaggatg
Pmv15         tgtggatgtcaggaagaggcgctggattaccttctgttccgctgaatggccaactttcaatgtgggatggcctcaggatg
Pmv16         tgtggatgtcaAgaagaggcgctAgattaccttctgttccgctgaatggccaactttcaatgtgggatggcctcaggatg
Pmv17         tgtggatgtcaggaagaggcgctggattaccttctgttccgctgaatggccaactttcaatgtgggatggcctcaggatg
Pmv18         tgtggatgtcaggaagaggcgctggattaccttctgttccgctgaatggccaactttcaatgtgggatggcctcaggatg
Pmv19         tgtggatgtcaggaagaggcgctggattaccttctgttccgctgaatggccaactttcaatgtgggatggcctcaggatg
Pmv2          tgtggatgtcaggaagaggcgctggattaccttctgttccgctgaatggccaactttcaatgtgggatggcctcaggatg
Pmv20         tgtggatgtcaggaagaggcgctggattaccttctgttccgctgaatggccaactttcaatgtgggatggcctcaggatg
Pmv21         tgtggatgtcaggaagaggcgctggattaccttctgttccgctgaatggccaactttcaatgtgggatggcctcaggatg
Pmv22         tgtggatgtcaggaagaggcgctggattaccttctgttccgctgaatggccaactttcaatgtgggatggcctcaggatg
Pmv23         tgtggatgtcaggaagaggcgctggattaccttctgttccgctgaatggccaactttcaatgtgggatggcctcaggatg
Pmv24         tgtggatgtcaggaagaggcgctggattaccttctgttccgctgaatggccaactttcaatgtgggatggcctcaggatg
Pmv4          tgtggatgtcaggaagaggcgctggattaccttctgttccgctgaatggccaaTtttcaatgtgggatggcctcaggatg
Pmv5          tgtggatgtcaggaagaggcgctggattaccttctgttccgctgaatggccaactttcaatgtgggatggcctcaggatg
Pmv6          tgtggatgtcaggaagaggcgctggattaccttctgttccgctgaatggccaactttcaatgtgggatggcctcaggatg
Pmv7          tgtggatgtcaggaagaggcgctggattaccttctgttccgctgaatggccaactttcaatgtgggatggcctcaggatg
Pmv8          tgtggatgtcaAgaagaggcgctggattaccttctgttccgctgaatggccaactttcaatgtgggatggcctcaggatg
Pmv9          tgtggatgtcaggaagaggcgctggattaccttctgttccgctgaatggccaactttcaatgtgggatggcctcaggatg
```

```
PMV_CONS    GTACTTTCAATTTAAGTATTATCTCTCAGGTTAAGTCTAGAGTGTTTTGTCCTGGTCCCCACGGACACCCGGATCAGGTC
Pmv1        gtactttcaatttaagtattatctctcaggttaagtctagagtgttttgtcctggtccccacggacacccggatcaggtc
Pmv10       gtactttcaatttaagtattatctctcaggttaagtctagagtgttttgtcctggtccccacggacacccggatcaggtc
Pmv11       gtactttcaatttaagtattatctctcaggttaagtctagagtgttttgtcctggtccccacggacacccggatcaggtc
Pmv12       gtactttcaatttaagtattatctctcaggttaagtctagagtgttttgtcctggtccccacggacacccggatcaggtc
Pmv13       gtactttcaatttaagtattatctctcaggttaagtctagagtgttttgtcctggtccccacggacacccggatcaggtc
Pmv14       gtactttcaatttaagtattatctctcaggttaagtctagagtgttttgtcctggtccccacggacacccAgatcaggtc
Pmv15       gtactttcaatttaagtattatctctcaggttaagtctagagtgttttgtcctggtccccacggacacccggatcaggtc
Pmv16       gtactttcaatttaagtattatctctcaggttaagtctagagtgttttgtcctggtccccacggacacccggatcaggtc
Pmv17       gtactttcaatttaagtattatctctcaggttaagtctagagtgttttgtcctggtccccacggacacccggatcaggtc
Pmv18       gtactttcaatttaagtattatctctcaggttaagtctagagtgttttgtcctggtccccacggacacccggatcaggtc
Pmv19       gtactttcaatttaagtattatctctcaggttaagtctagagtgttttgtcctggtccccacggacacccggatcaggtc
Pmv2        gtactttcaatttaagtattatctctcaggttaagtctagagtgttttgtcctggtccccacggacacccggatcaggtc
Pmv20       gtactttcaatttaagtattatctctcaggttaagtctagagtgttttgtcctggtccccacggacacccggatcaggtc
Pmv21       gtactttcaatttaagtattatctctcaggttaagtctagagtgttttgtcctggtccccacggacacccggatcaggtc
Pmv22       gtactttcaatttaagtattatctctcaggttaagtctagagtgttttgtcctggtccccacggacacccggatcaggtc
Pmv23       gtactttcaatttaagtattatctctcaggttaagtctagagtgttttgtcctggtccccacggacacccggatcaggtc
Pmv24       gtactttcaatttaagtattatctctcaggttaagtctagagtgttttgtcctggtccccacggacacccggatcaggtc
Pmv4        gtactttcaatttaagtattatctctcaggttaagtctagagtgttttgtcctggtccccacggacacccggatcaggtc
Pmv5        gtactttcaatttaagtattatctctcaggttaagtctagagtgttttgtcctggtccccacggacacccggatcaggtc
Pmv6        gtactttcaatttaagtattatctctcaggttaagtctagagtgttttgtcctggtccccacggacacccggatcaggtc
Pmv7        gtactttcaatttaagtattatctctcaggttaagtctagagtgttttgtcctggtccccacggacacccggatcaggtc
Pmv8        gtactttcaatttaagtattatctctcaggttaagtctagagtgttttgtcctggtccccacggacacccggatcaggtc
Pmv9        gtactttcaatttaagtattatctctcaggttaagtctagagtgttttgtcctggtccccacggacacccggatcaggtc

PMV_CONS    CCATATATCGTCACCTGGGAGGCACTTGCCTATGACCCCCCTCCGTGGGTCAAACCGTTTGTGTCTCCTAAACTTCCTCC
Pmv1        ccatatatcgtcacctgggaggcacttgcctatgacccccctccgtgggtcaaaccgtttgtgtctcctaaacttcctcc
Pmv10       ccatatatcgtcacctgggaggcacttgcctatgacccccctccgtgggtcaaaccgtttgtgtctcctaaacttcctcc
Pmv11       ccatatatcgtcacctgggaggcacttgcctatgacccccctccgtgggtcaaaccgtttgtgtctcctaaacttcctcc
Pmv12       ccatatatcgtcacctgggaggcacttgcctatgacccccctccgtgggtcaaaccgtttgtgtctcctaaacttcctcc
Pmv13       ccatatatcgtcacctgggaggcacttgcctatgacccccctccgtgggtcaaaccgtttgtgtctcctaaacttcctcc
Pmv14       ccatatatcgtcacctgggaggcacttgcctatgacccccctccgtgggtcaaaccgtttgtgtctcctaaacttcctcc
Pmv15       ccatatatcgtcacctgggaggcacttgcctatgacccccctccgtgggtcaaaccgtttgtgtctcctaaacttcctcc
Pmv16       ccatatatcgtcacctgggaggcacttgcctatgacccccctccgtgggtcaaaccgtttgtgtctcctaaacttcctcc
Pmv17       ccatatatcgtcacctgggaggcacttgcctatgacccccctccgtgggtcaaaccgtttgtgtctcctaaacttcctcc
Pmv18       ccatatatcgtcacctgggaggcacttgcctatgacccccctccgtgggtcaaaccgtttgtgtctcctaaacttcctcc
Pmv19       ccatatatcgtcacctgggaggcacttgcctatgacccccctccgtgggtcaaaccgtttgtgtctcctaaacttcctcc
Pmv2        ccatatatcgtcacctgggaggcacttgcctatgacccccctccgtgggtcaaaccgtttgtgtctcctaaacttcctcc
Pmv20       ccatatatcgtcacctgggaggcacttgcctatgacccccctccgtgggtcaaaccgtttgtgtctcctaaacttcctcc
Pmv21       ccatatatcgtcacctgggaggcacttgcctatgacccccctccgtgggtcaaaccgtttgtgtctcctaaacttcctcc
Pmv22       ccatatatcgtcacctgggaggcacttgcctatgacccccctccgtgggtcaaaccgtttgtgtctcctaaacttcctcc
Pmv23       ccatatatcgtcacctgggaggcacttgcctatgacccccctccgtgggtcaaaccgtttgtgtctcctaaactCcctcc
Pmv24       ccatatatcgtcacctgggaggcacttgcctatgacccccctccgtgggtcaaaccgttCgtgtctcctaaacttcctcc
Pmv4        ccatatatcgtcacctgggaggcacttgcctatgacccccctccgtgggtcaaaccgtttgtgtctcctaaacttcctcc
Pmv5        ccatatatcgtcacctgggaggcacttgcctatgacccccctccgtgggtcaaaccgtttgtgtctcctaaacttcctcc
Pmv6        ccatatatcgtcacctgggaggcacttgcctatgacccccctccgtgggtcaaaccgtttgtgtctcctaaacttcctcc
Pmv7        ccatatatcgtcacctgggaggcacttgcctatgacccccctccgtgggtcaaaccgtttgtgtctcctaaacttcctcc
Pmv8        ccatatatcgtcacctgggaggcacttgcctatgacccccctccgtgggtcaaaccgtttgtgtctcctaaacttcctcc
Pmv9        ccatatatcgtcacctgggaggcacttgcctatgacccccctccgtgggtcaaaccgtttgtgtctcctaaacttcctcc
```

```
PMV_CONS    CTTGCCGACAGCTCCCGTCCTCCCGCCCGGTCCTTCTGCGCAACCTCCGTCCCGATCTGCCCTTTACCCTGCCCTTACCC
Pmv1        cttgccgacagctcccgtcctcccgcccggtccttctgcgcaacctccgtcccgatctgccctttaccctgcccttaccc
Pmv10       cttgccgacagctcccgtcctcccgcccggtccttctgcgcaacctccgtcccgatctgccctttaccctgcccttaccc
Pmv11       cttgccgacagctcccgtcctcccgcccggtccttctgcgcaacctccgtcccgatctgccctttaccctgcccttaccc
Pmv12       cttgccgacagctcccgtcctcccgcccggtccttctgcgcaacctccgtcccgatctgccctttaccctgcccttaccc
Pmv13       cttgccgacagctcccgtcctcccgcccggtccttctgcgcaacctccgtcccgatctgccctttaccctgcccttaccc
Pmv14       cttgccgacagctcccgtcctcccgcccggtccttctgcgcaacctccgtcccgatctgccctttaccctgcccttaccc
Pmv15       cttgccgacagctcccgtcctcccgcccggtccttctgcgcaacctccgtcccgatctgccctttaccctgcccttaccc
Pmv16       cttgccgacagctcccgtcctcccgcccggtccttctgcgcaacctccgtcccgatctgccctttaccctgcccttaccc
Pmv17       cttgccgacagctcccgtcctcccgcccggtccttctgcgcaacctccgtcccgatctgccctttaccctgcccttaccc
Pmv18       cttgccgacagctcccgtcctcccgcccggtccttctgcgcaacctccgtcccgatctgccctttaccctgcccttaccc
Pmv19       cttgccgacagctcccgtcctcccgcccggtccttctgcgcaacctccgtcccgatctgccctttaccctgcccttaccc
Pmv2        cttgccgacagctcccgtcctcccgcccggtccttctgcgcaacctccgtcccgatctgccctttaccctgcccttaccc
Pmv20       cttgccgacagctcccgtcctcccgcccggtccttctgcgcaacctccgtcccgatctgccctttaccctgcccttaccc
Pmv21       cttgccgacagctcccgtcctcccgcccggtccttctgcgcaacctccgtcccgatctgccctttaccctgcccttaccc
Pmv22       cttgccgacagctcccgtcctcccgcccggtccttctgcgcaacctccgtcccgatctgccctttaccctgcccttaccc
Pmv23       cttgccgacagctcccgtcctcccgcccggtccttctgcgcaacctccgtcccgatctgccctttaccctgcccttaccc
Pmv24       cttgccgacagctcccgtcctcccgcccggtccttctgcgcaacctccgtcccgatctgccctttaccctgcccttaccc
Pmv4        cttgccgacagctcccgtcctcccgcccggtccttctgcgcaacctccgtcccgatctgccctttaccctgcccttaccc
Pmv5        cttgccgacagctcccgtcctcccgcccggtccttctgcgcaacctccgtcccgatctgccctttaccctgcccttaccc
Pmv6        cttgccgacagctcccgtcctcccgcccggtccttctgcgcaacctccgtcccgatctgccctttaccctgcccttaccc
Pmv7        cttgccgacagctcccgtcctcccgcccggtccttctgcgcaacctccgtcccgatctgccctttaccctgcccttaccc
Pmv8        cttgccgacagctcccgtcctcccgcccggtccttctgcgcaacctccgtcccgatctgccctttaccctgcccttaccc
Pmv9        cttgccgacagctcccgtcctcccgcccggtccttctgcgcaacctccgtcccgatctgccctttaccctgcccttaccc

PMV_CONS    CCTCTATAAAGTCCAAACCTCCTAAGCCCCAGGTTCTCCCTGATAGCGGCGGACCCCTCATTGACCTTCTCACAGAGGAC
Pmv1        cctctataaagtccaaacctcctaagccccaggttctccctgatagcggcggacccctcattgaccttctcacagaggac
Pmv10       cctctataaagtccaaacctcctaagccccaggttctccctgatagcggcggacccctcattgaccttctcacagaggac
Pmv11       cctctataaagtccaaacctcctaagccccaggttctccctgatagcggcggacccctcattgaccttctcacagaggac
Pmv12       cctctataaagtccaaacctcctaagccccaggttctccctgatagcggcggacccctcattgaccttctcacagaggac
Pmv13       cctctataaagtccaaacctcctaagccccaggttctccctgatagcggcggacccctcattgaccttctcacagaggac
Pmv14       cctctataaagtccaaacctcctaagccccaggttctccctgatagcggcggacccctcattgaccttctcacagaggac
Pmv15       cctctataaagtccaaacctcctaagccccaggttctccctgatagcggcggacccctcattgaccttctcacagaggac
Pmv16       cctctataaagtccaaacctcctaagccccaggttctccctgatagcggcggacccctcattgaccttctcacagaggac
Pmv17       cctctataaagtccaaacctcctaagccccaggttctccctgatagcggcggacccctcattgaccttctcacagaggac
Pmv18       cctctataaagtccaaacctcctaagccccaggttctccctgatagcggcggacccctcattgaccttctcacagaggac
Pmv19       cctctataaagtccaaacctcctaagccccaggttctccctgatagcggcggacccctcattgaccttctcacagaggac
Pmv2        cctctataaagtccaaacctcctaagccccaggttctccctAatagcggcggacccctcattgaccttctcacagaggac
Pmv20       cctctataaagtccaaacctcctaagccccaggttctccctgatagcggcggacccctcattgaccttctcacagaggac
Pmv21       cctctataaagtccaaacctcctaagccccaggttctccctgatagcggcggacccctcattgaccttctcacagaggac
Pmv22       cctctataaagtccaaacctcctaagccccaggttctccctgatagcggcggacccctcattgaccttctcacagaggac
Pmv23       cctctataaagtccaaacctcctaagccccaggttctccctgatagcggcggacccctcattgaccttctcacagaggac
Pmv24       cctctataaagtccaaacctcctaagccccaggttctccctgatagcggcggacccctcattgaccttctcacagaggac
Pmv4        cctctataaagtccaaacctcctaagccccaggttctccctgatagcggcggacccctcattgaccttctcacagaggac
Pmv5        cctctataaagtccaaacctcctaagccccaggttctccctgatagcggcggacccctcattgaccttctcacagaggac
Pmv6        cctctataaagtccaaacctcctaagccccaggttctccctgatagcggcggacccctcattgaccttctcacagaggac
Pmv7        cctctataaagtccaaacctcctaagccccaggttctccctgatagcggcggacccctcattgaccttctcacagaggac
Pmv8        cctctataaagtccaaacctcctaagccccaggttctccctgatagcggcggacccctcattgaccttctcacagaggac
Pmv9        cctctataaagtccaaacctcctaagccccaggttctccctgatagcggcggacccctcattgaccttctcacagaggac
```

```
PMV_CONS      CCCCCGCCGTACAGAGCACAACCCTCCTCCTCTGCCAGGGAGAACGACGAAGAAGAGGCGGCCACCACCTCCGAGGTTTC
Pmv1          cccccgccgtacagagcacaaccctcctcctctgccagggagaacgacgaagaagaggcggccaccacctccgaggtttc
Pmv10         cccccgccgtacagagcacaaccctcctcctctgccagggagaacgacgaagaagaggcggccaccacctccgaggtttc
Pmv11         cccccgccgtacagagcacaaccctcctcctctgccagggagaacgacgaagaagaggcggccaccacctccgaggtttc
Pmv12         cccccgccgtacagagcacaaccctcctcctctgccagggagaacgacgaagaagaggcggccaccacctccgaggtttc
Pmv13         cccccgccgtacagagcacaaccctcctcctctgccagggagaacgacgaagaagaggcggccaccacctccgaggtttc
Pmv14         cccccgccgtacagagcacaaccctcctcctctgccagggagaacgacgaagaagaggcggccaccacctccgaggtttc
Pmv15         cccccgccgtacagagcacaaccctcctcctctgccagggagaacgacgaagaagaggcggccaccacctccgaggtttc
Pmv16         cccccgccgtacagagcacaaccctcctcctctgccagggagaacgacgaagaagaggcggccaccacctccgaggtttc
Pmv17         cccccgccgtacagagcacaaccctcctcctctgccagggagaacgacgaagaagaggcggccaccacctccgaggtttc
Pmv18         cccccgccgtacagagcacaaccctcctcctctgccagggagaacgacgaagaagaggcggccaccacctccgaggtttc
Pmv19         cccccgccgtacagagcacaaccctcctcctctgccagggagaacgacgaagaagaggcggccaccacctccgaggtttc
Pmv2          cccccgccgtacagagcacaaccctcctcctctgccagggagaacgacgaagaagaggcggccaccacctccgaggtttc
Pmv20         cccccgccgtacagagca--------------------------------------------------------------
Pmv21         cccccgccgtacagagcacaaccctcctcctctgccagggaAaacgacgaagaagaggcggccaccacctccgaggtttc
Pmv22         cccccgccgtacagagcacaaccctcctcctctgccagggagaacgacgaagaagaggcggccaccacctccgaggtttc
Pmv23         cccccgccgtacagagcacaaccctcctcctctgccagggagaacgacgaagaagaggcggccaccacctccgaggtttc
Pmv24         cccccgccgtacagagcacaaccctcctcctctgccagggagaacgacgaagaagaggcggccaccacctccgaggtttc
Pmv4          cccccgccgtacagagcacaaccctcctcctctgccagggagaacgacgaagaagaggcggccaccacctccgaggtttc
Pmv5          cccccgccgtacagagcacaaccctcctcctctgccagggagaacgacgaagaagaggcggccaccacctccgaggtttc
Pmv6          cccccgccgtacagagcacaaccctcctcctctgccagggagaacgacgaagaagaggcggccaccacctccgaggtttc
Pmv7          cccccgccgtacagagcacaaccctcctcctctgccagggagaacgacgaagaagaggcggccaccacctccgaggtttc
Pmv8          cccccgccgtacagagcacaaccctcctcctctgccagggagaacgacgaagaagaggcggccaccacctccgaggtttc
Pmv9          cccccgccgtacagagcacaaccctcctcctctgccagggagaacgacgaagaagaggcggccaccacctccgaggtttc

PMV_CONS      C---CCCCCTTCTCCCATGGTGTCTCGACTGCGGGGAAGGAGAGACCCTCCCGCAGTGGACTCCACCACCTCCCAGGCAT
Pmv1          c---cccccttctcccatggtgtctcgactgcggggaaggagagaccctcccgcagtggactccaccacctcccaggcat
Pmv10         c---cccccttctcccatggtgtctcgactgcggggaaggagagaccctcccgcagtggactccaccacctcccaggcat
Pmv11         c---cccccttctcccatggtgtctcgactgcggggaaggagagaccctcccgcagtggactccaccacctcccaggcat
Pmv12         c---cccccttctcccatggtgtctcgactgcggggaaggagagaccctcccgcagtggactccaccacctcccaggcat
Pmv13         c---cccccttctcccatggtgtctcgactgcggggaaggagagaccctcccgcagtggactccaccacctcccaggcat
Pmv14         c---cccccttctcccatggtgtctcgactgcggggaaggagagaccctcccgcagtggactccaccacctcccaggcat
Pmv15         c---cccccttctcccatggtgtctcgactgcggggaaggagagaccctcccgcagtggactccaccacctcccaggcat
Pmv16         c---cccccttctcccatggtgtctcgactgcggggaaggagagaccctcccgcagtggactccaccacctcccaggcat
Pmv17         cCCCcccccttctcccatggtgtctcgactgcggggaaggagagaccctcccgcagtggactccaccacctcccaggcat
Pmv18         c---cccccttctcccatggtgtctcgactgcggggaaggagagaccctcccgcagtggactccaccacctcccaggcat
Pmv19         c---cccccttctcccatggtgtctcgactgcggggaaggagagaccctcccgcagtggactccaccacctcccaggcat
Pmv2          c---cccccttctcccatggtgtctcgactgcggggaaggagagaccctcccgcagtggactccaccacctcccaggcat
Pmv20         -------------------------------------------Aaccctcccgcagtggactccaccacctcccaggcat
Pmv21         c---cccccttctcccatggtgtctcgactgcggggaaggagagaccctcccgcagtggactccaccacctcccaggcat
Pmv22         c---cccccttctcccatggtgtctcgactgcggggaaggagagaccctcccgcagtggactccaccacctcccaggcat
Pmv23         c---cccccttctcccatggtgtctcgactgcggggaaggagagaccctcccgcagtggactccaccacctcccaggcat
Pmv24         c---cccccttctcccatggtgtctcgactgcggggaaggagagaccctcccgcagtggactccaccacctcccaggcat
Pmv4          c---cccccttctcccatggtgtctcgactgcggggaaggagagaccctcccgcagtggactccaccacctcccaggcat
Pmv5          c---cccccttctcccatggtgtctcgactgcggggaaggagagacc-tcccgcagtggactccaccacctcccaggcat
Pmv6          c---cccccttctcccatggtgtctcgactgcggggaaggagagaccctcccgcagtggactccaccacctcccaggcat
Pmv7          c---cccccttctcccatggtgtctcgactgcggggaaggagagaccctcccgcagtggactccaccacctcccaggcat
Pmv8          c---cccccttctcccatggtgtctcgactgcggggaaAgagagaccctcccgcagtggactccaccacctcccaggcat
Pmv9          c---cccccttctcccatggtgtctcgactgcggggaaggagagaccctcccgcagtggactccaccacctcccaggcat
```

```
PMV_CONS      TTCCACTCCGCATGGGGGGAGATGGCCAGCTTCAGTATTGGCCGTTTTCCTCTTCGGATTTATACAATTGGAAAAATAAT
Pmv1          ttccactccgcatggggggagatggccagcttcagtattggccgttttcctcttcggatttatacaattggaaaaataat
Pmv10         ttccactccgcatggggggagatggccagcttcagtattggccgttttcctcttcggatttatacaattggaaaaataat
Pmv11         ttccactccgcatggggggagatggccagcttcagtattggccgttttcctcttcggatttatacaattggaaaaataat
Pmv12         ttccactccgcatggggggagatggccagcttcagtattggccgttttcctcttcggatttatacaattggaaaaataat
Pmv13         ttccactccgcatggggggagatggccagcttcagtattggccgttttcctcttcggatttatacaattggaaaaataat
Pmv14         ttccactccgcatggggggagatggccagcttcagtattggccgttttcctcttcggatttatacaattggaaaaataat
Pmv15         ttccactccgcatggggggagatggccagcttcagtattggccgttttcctcttcggatttatacaattggaaaaataat
Pmv16         ttccactccgcatggggggagatggccagcttcagtattggccgttttcctcttcggatttatacaattggaaaaataat
Pmv17         ttccactccgcatggggggagatggccagcttcagtattggccgttttcctcttcggatttatacaattggaaaaataat
Pmv18         ttccactccgcatggggggagatggccagcttcagtattggccgttttcctcttcggatttatacaattggaaaaataat
Pmv19         ttccactccgcatggggggagatggccagcttcagtattggccgttttcctcttcggatttatacaattggaaaaataat
Pmv2          ttccactccgcatggggggagatggccagcttcagtattggccgttttcctcttcggatttatacaattggaaaaataat
Pmv20         ttccactccgcatggggggagatggccagcttcagtattggccgttttcctcttcggatttatacaattggaaaaataat
Pmv21         ttccactccgcatggggggagatggccagcttcagtattggccgttttcctcttcggatttatacaattggaaaaataat
Pmv22         ttccactccgcatggggggagatggccagcttcagtattggccgttttcctcttcggatttatacaattggaaaaataat
Pmv23         ttccactccgcatggggggagatggccagcttcagtattggccgttttcctcttcggatttatacaattggaaaaataat
Pmv24         ttccactccgcatggggggagatggccagcttcagtattggccgttttcctcttcggatttatacaattggaaaaataat
Pmv4          ttccactccgcatggggggagatggccagcttcagtattggccgttttcctcttcggatttatacaattggaaaaataat
Pmv5          ttccactccgcatggggggagatggccagcttcagtattggccgttttcctcttcggatttatacaattggaaaaataat
Pmv6          ttccactccgcatggggggagatggccagcttcagtattggccgttttcctcttcggatttatacaattggaaaaataat
Pmv7          ttccactccgcatggggggagatggccagcttcagtattggccgttttcctcttcggatttatacaattggaaaaataat
Pmv8          ttccactccgcatggggggagatggccagcttcagtattggccgttttcctcttcggatttatacaattggaaaaataat
Pmv9          ttccactccgcatggggggagatggccagcttcagtattggccgttttcctcttcggatttatacaattggaaaaataat

PMV_CONS      AACCCTTCCTTTTCTGAAGATCCAGGTAAATTGACGGCCTTGATTGAGTCCGTCCTCATCACCCACCAGCCCACCTGGGA
Pmv1          aacccttccttttctgaagatccaggtaaattgacggccttgattgagtccgtcctcatcacccaccagcccacctggga
Pmv10         aacccttccttttctgaagatccaggtaaattgacggccttgattgagtccgtcctcatcacccaccagcccacctggga
Pmv11         aacccttccttttctgaagatccaggtaaattgacggccttgattgagtccgtcctcatcacccaccagcccacctggga
Pmv12         aacccttccttttctgaagatccaggtaaattgacggccttgattgagtccgtcctcatcacccaccagcccacctggga
Pmv13         aacccttccttttctgaagatccaggtaaattgacggccttgattgagtccgtcctcatcacccaccagcccacctggga
Pmv14         aacccttccttttctgaagatccaggtaaattgacggccttgattgagtccgtcctcatcacccaccagcccacctggga
Pmv15         aacccttccttttctgaagatccaggtaaattgacggccttgattgagtccgtcctcatcacccaccagcccacctggga
Pmv16         aacccttccttttctgaagatccaggtaaattgacggccttgattgagtccgtcctcatcacccaccagcccacctggga
Pmv17         aacccttccttttctgaagatccaggtaaattgacggccttgattgagtccgtcctcatcacccaccagcccacctggga
Pmv18         aacccttccttttctgaagatccaggtaaattgacggccttgattgagtccgtcctcatcacccaccagcccacctggga
Pmv19         aacccttccttttctgaagatccaggtaaattgacggccttgattgagtccgtcctcatcacccaccagcccacctggga
Pmv2          aacccttccttttctgaagatccaggtaaattgacggccttgattgagtccgtcctcatcacccaccagcccacctggga
Pmv20         aacccttccttttctgaagatccaggtaaattgacggccttgattgagtccgtcctcatcacccaccagcccacctggga
Pmv21         aacccttccttttctgaagatccaggtaaattgacggccttgattgagtccgtcctcatcacccaccagcccacctggga
Pmv22         aacccttccttttctgaagatccaggtaaattgacggccttgattgagtccgtcctcatcacccaccagcccacctggga
Pmv23         aacccttccttttctgaagatccaggtaaattgacggccttgattgagtccgtcctcatcacccaccagcccacctggga
Pmv24         aacccttccttttctgaagatccaggtaaattgacggccttgattgagtccgtcctcatcacccaccagcccacctggga
Pmv4          aacccttccttttctgaagatccaggtaaattgacggccttAattgagtccgtcctcatcacccaccagcccacctggga
Pmv5          aacccttccttttctgaagatccaggtaaattgacggccttgattgagtccgtcctcatcacccaccagcccacctggga
Pmv6          aacccttccttttctgaagatccaggtaaattgaTggccttgattgagtccgtcctcatcacccaccagcccacctggga
Pmv7          aacccttccttttctgaagatccaggtaaattgacggccttgattgagtccgtcctcatcacccaccagcccacctggga
Pmv8          aacccttccttttctgaagatccaggtaaattgacggccttgattgagtccgtcctcatcacccaccagcccacctggga
Pmv9          aacccttccttttctgaagatccaggtaaattgacggccttgattgagtccgtcctcatcacccaccagcccacctggga
```

```
PMV_CONS      CGACTGTCAGCAGTTGTTGGGGACCCTGCTGACCGGAGAAGAAAAGCAGCGGGTGCTCCTAGAGGCTAGAAAGGCAGTCC
Pmv1          cgactgtcagcagttgttggggaccctgctgaccggagaagaaaagcagcgggtgctcctagaggctagaaaggcagtcc
Pmv10         cgactgtcagcagttgttggggaccctgctgaccggagaagaaaagcagcgggtgctcctagaggctagaaaggcagtcc
Pmv11         cgactgtcagcagttgttggggaccctgctgaccggagaagaaaagcagcgggtgctcctagaggctagaaaggcagtcc
Pmv12         cgactgtcagcagttgttggggaccctgctgaccggagaagaaaagcagcgggtgctcctagaggctagaaaggcagtcc
Pmv13         cgactgtcagcagttgttggggaccctgctgaccggagaagaaaagcagcgggtgctcctagaggctagaaaggcagtcc
Pmv14         cgactgtcagcagttgttggggaccctgctgaccggagaagaaaagcagcgggtgctcctagaggctagaaaggcagtcc
Pmv15         cgactgtcagcagttgttggggaccctgctgaccggagaagaaaagcagcgggtgctcctagaggctagaaaggcagtcc
Pmv16         cgactgtcagcagttgttggggaccctgctgaccggagaagaaaagcagcgggtgctcctagaggctagaaaggcagtcc
Pmv17         cgactgtcagcagttgttggggaccctgctgaccggagaagaaaagcagcgggtgctcctagaggctaAaaaggcagtcc
Pmv18         cgactgtcagcagttgttggggaccctgctgaccggagaagaaaagcagcgggtgctcctagaggctagaaaggcagtcc
Pmv19         cgactgtcagcagttgttggggaccctgctgaccggagaagaaaagcagcgggtgctcctagaggctagaaaggcagtcc
Pmv2          cgactgtcagcagttgttggggaccctgctgaccggagaagaaaagcagcgggtgctcctagaggctagaaaggcagtcc
Pmv20         cgactgtcagcagttgttggggaccctgctgaccggagaagaaaagcagcgggtgctcctagaggctagaaaggcagtcc
Pmv21         cgactgtcagcagttgttggggaccctgctgaccggagaagaaaagcagcgggtgctcctagaggctagaaaggcagtcc
Pmv22         cgactgtcagcagttgttggggaccctgctgaccggagaagaaaagcagcgggtgctcctagaggctagaaaggcagtcc
Pmv23         cgactgtcagcagttgttggggaccctgctgaccggagaagaaaagcagcgggtgctcctagaggctagaaaggcagtcc
Pmv24         cgactgtcagcagttgttggggaccctgctgaccggagaagaaaagcagcgggtgctcctagaggctagaaaggcagtcc
Pmv4          cgactgtcagcagttgttggggaccctgctgaccggaAaagaaaagcagcgggtgctcctagaggctagaaaggcagtcc
Pmv5          cgactgtcagcagttgttggggaccctgctgaccggagaagaaaagcagcgggtgctcctagaggctagaaaggcagtcc
Pmv6          cgactgtcagcagttgttggggaccctgctgaccggagaagaaaagcagcgggtgctcctagaggctagaaaggcagtcc
Pmv7          cgactgtcagcagttgttggggaccctgctgaccggagaagaaaagcagcgggtgctcctagaggctagaaaggcagtcc
Pmv8          cgactgtcagcagttgttggggaccctgctgaccggagaagaaaagcagcgggtgctcctagaggctagaaaggcagtcc
Pmv9          cgactgtcagcagttgttggggaccctgctgaccggagaagaaaagcagcgggtgctcctagaggctagaaaggcagtcc

PMV_CONS      GGGGCAATGATGGACGCCCCACTCAGTTGCCTAATGAAGTCAATGCTGCTTTTCCCCTTGAACGCCCCGGTTGGGATTAC
Pmv1          ggggcaatgatggacgccccactcagttgcctaatgaagtcaatgctgcttttccccttgaacgccccggttgggattac
Pmv10         ggggcGatgatggacgccccactcagttgcctaatgaagtcaatgctgcttttccccttgaacgccccggttgggattac
Pmv11         ggggcaatgatggacgccccactcagttgcctaatgaagtcaatgctgcttttccccttgaacgccccggttgggattac
Pmv12         ggggcaatgatggacgccccactcagttgcctaatgaagtcaatgctgcttttccccttgaacgccccggttgggattac
Pmv13         ggggcGatgatggacgccccactcagttgcctaatgaagtcaatgctgcttttccccttgaacgccccggttgggattac
Pmv14         ggggcaatgatggacgccccactcagttgcctaatgaagtcaatgctgcttttccccttgaacgccccggttgggattac
Pmv15         ggggcaatgatggacgccccactcagttgcctaatAaagtcaatgctgcttttccccttgaacgccccggttgggattac
Pmv16         ggggcaatgatggacgccccactcagttgcctaatgaagtcaatgctgcttttccccttgaacgccccggttgggattac
Pmv17         ggggcaatgatggacgccccactcagttgcctaatgaagtcaatgctgcttttccccttgaacgccccggttgggattac
Pmv18         ggggcaatgatggacgccccactcagttgcctaatgaagtcaatgctgcttttccccttgaacgccccggttgggattac
Pmv19         ggggcaatgatggacgccccactcagttgcctaatgaagtcaatgctgcttttccccttgaacgccccggttgggattac
Pmv2          ggggcaatgatggacgccccactcagttgcctaatgaagtcaatgctgcttttccccttgaacgccccggttgggattac
Pmv20         ggggcaatgatggacgccccactcagttAcctaatgaagtcaatgctgcttttccccttgaacgccccggttgggattac
Pmv21         ggggcaatgatggacgccccactcagttgcctaatgaagtcaatgctgcttttccccttgaacgccccggttgggattac
Pmv22         ggggcaatgatggacgccccactcagttgcctaatgaagtcaatgctgcttttccccttgaacgccccggttgggattac
Pmv23         ggggcGatgatggacgccccactcagttgcctaatgaagtcaatgctgcttttccccttgaacgccccggttgggattac
Pmv24         ggggcaatgatggacgccccactcagttgcctaatgaagtcaatgctgcttttccccttgaacgccccggttgggattac
Pmv4          gggAcaatgatggacgccccactcagttgcctaatgaagtcaatgctgcttttccccttAaacgccccggttgggattac
Pmv5          ggggcaatgatggacgccccactcagttgcctaatgaagtcaatgctgcttttccccttgaacgccccggttgggattac
Pmv6          ggggcaatgatggacgccccactcagttgcctaatgaagtcaatgctgcttttccccttgaacgccccggttgggattac
Pmv7          ggggcaatgatggacgccccactcagttgcctaatgaagtcaatgctgcttttccccttgaacgccccggttgggattac
Pmv8          ggggcaatgatggacgccccactcagttgcctaatgaagtcaatgctgcttttccccttgaacgccccggttgggattac
Pmv9          ggggcaatgatggacgccccactcagttgcctaatgaagtcaatgctgcttttccccttAaacgccccggttgggattac
```

```
PMV_CONS      ACCACTACAGAAGGTAGGAACCACCTAGTCCTCTATCGCCAGTTGCTCTTAGCGGGTCTCCAGAACGCGGGCAGAAGTCC
Pmv1          accactacagaaggtaggaaccacctagtcctctatcgccagttgctcttagcgggtctccagaacgcgggcagaagtcc
Pmv10         accactacagaaggtaggaaccacctagtcctctatcgccagttgctcttagcgggtctccagaacgcgggcagaagtcc
Pmv11         accactacagaaggtaggaaccacctagtcctctatcgccagttgctcttagcgggtctccagaacgcgggcagaagtcc
Pmv12         accactacagaaggtaggaaccacctagtcctctatcgccagttgctcttagcgggtctccagaacgcgggcagaagtcc
Pmv13         accactacagaaggtaggaaccacctagtcctctatcgccagttgctcttagcgggtctccagaacgcgggcagaagtcc
Pmv14         accactacagaaggtaggaaccacctagtcctctatcgccagttgctcttagcgggtctccagaacgcgggcagaagtcc
Pmv15         accactacagaaggtaggaaccacctagtcctctatcgccagttgctcttagcgggtctccagaacgcgggcagaagtcc
Pmv16         accactacagaaggtaggaaccacctagtcctctatcgccagttgctcttagcgggtctccagaacgcgggcagaagtcc
Pmv17         accactacagaaggtaggaaccacctagtcctctatcgccagttgctcttagcgggtctccagaacgcgggcagaagtcc
Pmv18         accactacagaaggtaggaaccacctagtcctctatcgccagttgctcttagcgggtctccagaacgcgggcagaagtcc
Pmv19         accactacagaaggtaggaaccacctagtcctctatcgccagttgctcttagcgggtctccagaacgcgggcagaagtcc
Pmv2          accactacagaaggtaggaaccacctagtcctctatcgccagttgctcttagcgggtctccagaacgcgggcagaagtcc
Pmv20         accactacagaaggtaggaaccacctagtcctctatcgccagttgctcttagcgggtctccagaacgcgggcagaagtcc
Pmv21         accactacagaaggtaggaaccacctagtcctctatcgccagttgctcttagcgggtctccagaacgcgggcagaagtcc
Pmv22         accactacagaaggtaggaaccacctagtcctctatcgccagttgctcttagcgggtctccagaacgcgggcagaagtcc
Pmv23         accactacagaaggtaggaaccacctagtcctctatcgccagttgctcttagcgggtctccagaacgcgggcagaagtcc
Pmv24         accactacagaaggtaggaaccacctagtcctctatcgccagttgctcttagcgggtctccagaacgcgggcagaagtcc
Pmv4          accactacagaaggtaggaaccacctagtcctctatcgccagttgctcttagcgggtctccagaacgcgggcagaagtcc
Pmv5          accactacagaaggtaggaaccacctagtcctctatcgccagttgctcttagcgggtctccagaacgcgggcagaagtcc
Pmv6          accactacagaaggtaggaaccacctagtcctctatcgccagttgctcttagcgggtctccagaacgcgggcagaagtcc
Pmv7          accactacagaaggtaggaaccacctagtcctctatcgccagttgctcttagcgggtctccagaacgcgggcagaagtcc
Pmv8          accactacagaaggtaggaaccacctagtcctctatcgccagttgctcttagcgggtctccagaacgcgggcagaagtcc
Pmv9          accactacagaaggtaggaaccacctagtcctctatcgccagttgctcttagcgggtctccagaacgcgggcaAaagtcc

PMV_CONS      CACCAATTTGGCCAAGGTAAAAGGGATAACCCAGGGACCTAATGAGTCTCCCTCAGCCTTTTTAGAGAGACTCAAAGAGG
Pmv1          caccaatttggccaaggtaaaagggataacccagggacctaatgagtctccctcagcctttttagagagactcaaagagg
Pmv10         caccaatttggccaaggtaaaagggataacccagggacctaatgagtctccctcagcctttttagagagactcaaagagg
Pmv11         caccaatttggccaaggtaaaagggataacccagggacctaatgagtctccctcagcctttttagagagactcaaagagg
Pmv12         caccaatttggccaaggtaaaagggataacccagggacctaatgagtctccctcagcctttttagagagactcaaagagg
Pmv13         caccaatttggccaaggtaaaagggataacccagggacctaatgagtctccctcagcctttttagagagactcaaagagg
Pmv14         caccaatttggccaaggtaaaagggataacccagggacctaatgagtctccctcagcctttttagagagactcaaagagg
Pmv15         caccaatttggccaaggtaaaagggataacccagggacctaatgagtctccctcagcctttttagagagactcaaagagg
Pmv16         caccaatttggccaaggtaaaagggataacccagggacctaatgagtctccctcagcctttttagagagactcaaagagg
Pmv17         caccaatttggccaaggtaaaagggataacccagggacctaatgagtctccctcagcctttttagagagactcaaagagg
Pmv18         caccaatttggccaaggtaaaagggataacccagggacctaatgagtctccctcagcctttttagagagactcaaagagg
Pmv19         caccaatttggccaaggtaaaagggataacccagggacctaatgagtctccctcagcctttttagagagactcaaagagg
Pmv2          caccaatttggccaaggtaaaagggataacccagggacctaatgagtctccctcagcctttttagagagactcaaagagg
Pmv20         caccaatttggccaaggtaaaagggataacccagggacctaatgagtctccctcagcctttttagagagactcaaagagg
Pmv21         caccaatttggccaaggtaaaagggataacccagggacctaatgagtctccctcagcctttttagagagactcaaagagg
Pmv22         caccaatttggccaaggtaaaagggataacccagggacctaatgagtctccctcagcctttttagagagactcaaagagg
Pmv23         caccaatttggccaaggtaaaagggataacccagggacctaatgagtctccctcagcctttttagagagactcaaagagg
Pmv24         caccaatttggccaaggtaaaagggataacccagggacctaatgagtctccctcagcctttttagagagactcaaagagg
Pmv4          caccaatttggccaaggtaaaagggataacccagggacctaatgagtctccctcagcctttttagagagactcaaagagg
Pmv5          caccaatttggccaaggtaaaagggataacccagggacctaatgagtctccctcagcctttttagagagactcaaagagg
Pmv6          caccaatttggccaaggtaaaagggataacccagggacctaatgagtctccctcagcctttttagagagactcaaagagg
Pmv7          caccaatttggccaaggtaaaagggataacccagggacctaatgagtctccctcagcctttttagagagactcaaagagg
Pmv8          caccaatttggccaaggtaaaagggataacccagggacctaatgagtctccctcagcctttttagagagactcaaagagg
Pmv9          caccaatttggccaaggtaaaagggataacccagggacctaatgagtctccctcagcctttttagagagactcaaagagg
```

```
PMV_CONS      CCTATCGCAGGTACACTCCTTATGACCCTGAGGACCCAGGGCAAGAAACCAATGTGTCTATGTCATTCATCTGGCAGTCT
Pmv1          cctatcgcaggtacactccttatgaccctgaggacccagggcaagaaaccaatgtgtctatgtcattcatctggcagtct
Pmv10         cctatcgcaggtacactccttatgaccctgaggacccagggcaagaaaccaatgtgtctatgtcattcatctggcagtct
Pmv11         cctatcgcaggtacactccttatgaccctgaggacccagggcaagaaaccaatgtgtctatgtcattcatctggcagtct
Pmv12         cctatcgcaggtacactccttatgaccctgaggacccagggcaagaaaccaatgtgtctatgtcattcatctggcagtct
Pmv13         cctatcgcaggtacactccttatgaccctgaggacccagggcaagaaaccaatgtgtctatgtcattcatctggcagtct
Pmv14         cctatcgcaggtacactccttatgaccctgaggacccagggcaagaaaccaatgtgtctatgtcattcatctggcagtct
Pmv15         cctatcgcaggtacactccttatgaccctgaggacccagggcaagaaaccaatgtgtctatgtcattcatctggcagtct
Pmv16         cctatcgcaggtacactccttatgaccctgaggacccagggcaagaaaccaatgtgtctatgtcattcatctggcagtct
Pmv17         cctatcgcaggtacactccttatgaccctgaggacccagggcaagaaaccaatgtgtctatgtcattcatctggcagtct
Pmv18         cctatcgcaggtacactccttatgaccctgaggacccagggcaagaaaccaatgtgtctatgtcattcatctggcagtct
Pmv19         cctatcgcaggtacactccttatgaccctgaggacccagggcaagaaaccaatgtgtctatgtcattcatctggcagtct
Pmv2          cctatcgcaggtacactccttatgaccctgaggacccagggcaagaaaccaatgtgtctatgtcattcatctggcagtct
Pmv20         cctatcgcaggtacactccttatgaccctgaggacccagggcaagaaaccaatgtgtctatgtcattcatctggcagtct
Pmv21         cctatcgcaggtacactccttatgaccctgaggacccagggcaagaaaccaatgtgtctatgtcattcatctggcagtct
Pmv22         cctatcgcaggtacactccttatgaccctgaggacccagggcaagaaaccaatgtgtctatgtcattcatctggcagtct
Pmv23         cctatcgcaggtacactccttatgaccctgaggacccagggcaagaaaccaatgtgtctatgtcattcatctggcagtct
Pmv24         cctatcgcaggtacactccttatgaccctgaggacccagggcaagaaaccaatgtgtctatgtcattcatctggcagtct
Pmv4          cctatcgcaggtacactccttatgaccctgaggacccagggcaagaaaccaatgtgtctatgtcattcatctggcagtct
Pmv5          cctatcgcaggtacactccttatgaccctgaggacccagggcaagaaaccaatgtgtctatgtcattcatctggcagtct
Pmv6          cctatcgcaggtacactccttatgaccctgaggacccagggcaagaaaccaatgtgtctatgtcattcatctggcagtct
Pmv7          cctatcgcaggtacactccttatgaccctgaggacccagggcaagaaaccaatgtgtctatgtcattcatctggcagtct
Pmv8          cctatcgcaggtacactccttatgaccctgaggacccagggcaagaaaccaatgtgtctatgtcattcatctggcagtct
Pmv9          cctatcgcaggtacactccttatgaccctgaggacccagggcaagaaaccaatgtgtctatgtcattcatctggcagtct

PMV_CONS      GCCCCGGATATCGGGCGAAAGTTAGAGCGGTTAGAAGATTTAAAGAGCAAGACCTTAGGAGACTTAGTGAGGGAAGCTGA
Pmv1          gccccggatatcgggcgaaagttagagcggttagaagatttaaagagcaagaccttaggagacttagtgagggaagctga
Pmv10         gccccggatatcgggcgaaagttagagcggttagaagatttaaagagcaagaccttaggagacttagtgagggaagctga
Pmv11         gccccggatatcgggcgaaagttagagcggttagaagatttaaagagcaagaccttaggagacttagtgagggaagctga
Pmv12         gccccggatatcgggcgaaagttagagcggttagaagatttaaagagcaagaccttaggagacttagtgagggaagctga
Pmv13         gccccggatatcgggcgaaagttagagcggttagaagatttaaagagcaagaccttaggagacttagtgagggaagctga
Pmv14         gccccggatatcgggcgaaagttagagcggttagaagatttaaagagcaagaccttaggagacttagtgagggaagctga
Pmv15         gccccggatatcgggcgaaagttagagcggttagaagatttaaagagcaagaccttaggagacttagtgagggaagctga
Pmv16         gccccggatatcgggcgaaagttagagcggttagaagatttaaagagcaagaccttaggagacttagtgagggaagctga
Pmv17         gccccggatatcgggcgaaagttagagcggttagaagatttaaagagcaagaccttaggagacttagtgagggaagctga
Pmv18         gccccggatatcgggcgaaagttagagcggttagaagatttaaagagcaagaccttaggagacttagtgagggaagctga
Pmv19         gccccggatatcgggcgaaagttagagcggttagaagatttaaagagcaagaccttaggagacttagtgagggaagctga
Pmv2          gccccggatatcgggcgaaagttagagcggttagaagatttaaagagcaagaccttaggagacttagtgagggaagctga
Pmv20         gccccggatatcgggcgaaagttagagcggttagaagatttaaagagcaagaccttaggagacttagtgagggaagctga
Pmv21         gccccggatatcgggcgaaagttagagcggttagaagGtttaaagagcaagaccttaggagacttagtAagggaagctga
Pmv22         gccccggatatcgggcgaaagttagagcggttagaagatttaaagagcaagaccttaggagacttagtgagggaagctga
Pmv23         gccccggatatcgggcgaaagttagagcggttagaagatttaaagagcaagaccttaggagacttagtgagggaagctga
Pmv24         gccccggatatcgggcgaaagttagagcggttagaagatttaaagagcaagaccttaggagacttagtgagggaagctga
Pmv4          gccccggatatcgggcAaaagttagagcggttaAaagatttaaagagcaagaccttaggagacttagtgaggAaagctga
Pmv5          gccccggatatcgggcgaaagttagagcggttagaagatttaaagagcaagaccttaggagacttagtgagggaagctga
Pmv6          gccccggatatcgggcgaaagttagagcggttagaagatttaaagagcaagaccttaggagacttagtgagggaagctga
Pmv7          gccccggatatcgggcgaaagttagagcggttagaagatttaaagagcaagaccttaggagacttagtgagggaagctga
Pmv8          gccccggatatcgggcgaaagttagagcggttagaagatttaaagagcaagaccttaggagacttagtgagggaagctga
Pmv9          gccccggatatcgggcgaaagttagagcggttagaagatttaaagagcaagaccttaggagacttagtgagggaagctga
```

```
PMV_CONS      AAAGATCTTTAATAAGCGAGAAACCCCGGAAGAAAGAGAGGAACGTATCAAGAGAGAAACAGAGGAAAAAGAAGAACGCC
Pmv1          aaagatctttaataagcgagaaaccccggaagaaagagaggaacgtatcaagagagaaacagaggaaaaagaagaacgcc
Pmv10         aaagatctttaataagcgagaaaccccggaagaaagagaggaacgtatcaagagagaaacagaggaaaaagaagaacgcc
Pmv11         aaagatctttaataagcgagaaaccccggaagaaagagaggaacgtatcaagagagaaacagaggaaaaagaagaacgcc
Pmv12         aaagatctttaataagcgagaaaccccggaagaaagagaggaacgtatcaagagagaaacagaggaaaaagaagaacgcc
Pmv13         aaagatctttaataagcgagaaaccccggaagaaagagaggaacgtatcaagagagaaacagaggaaaaagaagaacgcc
Pmv14         aaagatctttaataagcgagaaaccccggaagaaagagaggaacgtatcaagagagaaacagaggaaaaagaagaacgcc
Pmv15         aaagatctttaataagcgagaaaccccggaagaaagagaggaacgtatcaagagagaaaTagaggaaaaagaagaacgcc
Pmv16         aaagatctttaataagcgagaaaccccggaagaaagagaggaacgtatcaagagagaaacagaggaaaaagaagaacgcc
Pmv17         aaagatctttaataagcgagaaaccccggaagaaagagaggaacgtatcaagagaAaaacagaggaaaaagaagaacgcc
Pmv18         aaagatctttaataagcgagaaaccccggaagaaagagaggaacgtatcaagagagaaacagaggaaaaagaagaacgcc
Pmv19         aaagatctttaataagcgagaaaccccggaagaaagagaggaacgtatcaagagagaaacagaggaaaaagaagaacgcc
Pmv2          aaagatctttaataagcgagaaaccccggaagaaagagaggaacgtatcaagagagaaacagaggaaaaagaagaacgcc
Pmv20         aaagatctttaataagcgagaaaccccggaagaaagagaggaacgtatcaagagagaaacagaggaaaaagaagaacgcc
Pmv21         aaagatctttaataagcgagaaaccccggaagaaagaAaggaacgtatcaagagagaaacagaggaaaaagaagaacgcc
Pmv22         aaagatctttaataagcgagaaaccccggaagaaagagaggaacgtatcaagagagaaacagaggaaaaagaagaacgcc
Pmv23         aaagatctttaataagcgagaaaccccggaagaaagagaggaacgtatcaagagagaaacagaggaaaaagaagaacgcc
Pmv24         aaagatctttaataagcgagaaaccccggaagaaagagaggaacgtatcaagagagaaacagaggaaaaagaagaacgcc
Pmv4          aaagatctttaataagcgaAaaaccccggaagaaagagaggaacgtatcaagagaAaaacagaggaaaaagaagaacgcc
Pmv5          aaagatctttaataagcgagaaaccccggaagaaagagaggaacgtatcaagagagaaacagaggaaaaagaagaacgcc
Pmv6          aaagatctttaataagcgagaaaccccggaagaaagagaggaacgtatcaagagagaaacagaggaaaaagaagaacgcc
Pmv7          aaagatctttaataagcgagaaaccccggaagaaagagaggaacgtatcaagagagaaacagaggaaaaagaagaacgcc
Pmv8          aaagatctttaataagcgagaaaccccggaagaaagagaggaacgtatcaagagagaaacagaggaaaaagaagaacgcc
Pmv9          aaagatctttaataagcgagaaaccccggaagaaagagaggaacgtatcaagagagaaacagaggaaaaagaagaacgcc

PMV_CONS      GTAGGGCAGAGGATGAGCAGAGAGAGAAAGAAAGGGACCGCAGAAGACATAGAGAGATGAGCAAGCTCTTGGCCACTGTA
Pmv1          gtagggcagaggatgagcagagagagaaagaaagggaccgcagaagacatagagagatgagcaagctcttggccactgta
Pmv10         gtagggcagaggatgagcagagagagaaagaaagggaccgcagaagacatagagagatgagcaagctcttggccactgta
Pmv11         gtagggcagaggatgagcagagagagaaagaaagggaccgcagaagacatagagagatgagcaagctcttggccactgta
Pmv12         gtagggcagaggatgagcagagagagaaagaaagggaccgcagaagacatagagagatgagcaagctcttggccactgta
Pmv13         gtagggcagaggatgagcagagagagaaagaaagggaccgcagaagacatagagagatgagcaagctcttggccactgta
Pmv14         gtagggcagaggatgagcagagagagaaagaaagggaccgcagaagacatagagagatgagcaagctcttggccactgta
Pmv15         gtagggcagaggatgagcagagagagaaagaaagggaccgcagaagacatagagagatgagcaagctcttggccactgta
Pmv16         gtagggcagaggatgagcagagagagaaagaaagggaccgcagaagacatagagagatgagcaagctcttggccactgta
Pmv17         gtagggcagaggatgagcagagagagaaaAaaagggaccgcaAaagacatagagagatgagcaagctcttggccactgta
Pmv18         gtagggcagaggatgagcagagagagaaagaaagggaccgcagaagacatagagagatgagcaagctcttggccactgta
Pmv19         gtagggcagaggatgagcagagagagaaagaaagggaccgcagaagacatagagagatgagcaagctcttggccactgta
Pmv2          gtagggcagaggatgagcagagagaAaaagaaagggaccgcagaagacatagagagatgagcaagctcttggccactgta
Pmv20         gtagggcagaggatgagcagagagagaaagaaagggaccgcagaagacatagagagatgagcaagctcttggccactgta
Pmv21         gtagggcagaggatgagcagagagagaaagaaagggaccgcagaagacatagagagatgagcaagctcttggccactgta
Pmv22         gtagggcagaggatgagcagagagagaaagaaagggaccgcagaagacatagagagatgagcaagctcttggccactgta
Pmv23         gtagggcagaggatgagcagagagagaaagaaagggaccgcagaagacatagagagatgagcaagctcttggccactgta
Pmv24         gtagggcagaggatgagcagagagagaaagaaagggaccgcagaagaTatagagagatgagcaagctcttggccactgta
Pmv4          gtagggcagaggatgagcagGAaAaAaaaAaaagggaccgcagaagacatagagaAatgagcaagctcttggccactgta
Pmv5          gtagggcagaggatgagcagagagagaaagaaagggaccgcagaagacatagagagatgagcaagctcttggccactgta
Pmv6          gtagggcagaggatgagcagagagagaaagaaagggaccgcagaagacatagagagatgagcaagctcttggccactgta
Pmv7          gtagggcagaggatgagcagagagagaaagaaagggaccgcagGagacatagagagatgagcaagctcttggccactgta
Pmv8          gtagggcagaggatgagcagagagagaaagaaagggaccgcagaagacatagagagatgagcaagctcttggccactgta
Pmv9          gtagggcagaggatgagcagagagagaaagaaagggaccgcagaagacatagagagatgagcaagctcttggccactgta
```

```
PMV_CONS      GTTATTGGTCAGAGACAGGATAGACAGGGGGGAGAGCGGAGGAGGCCCCAACTTGATAAGGACCAATGCGCCTACTGCAA
Pmv1          gttattggtcagagacaggatagacaggggggagagcggaggaggccccaacttgataaggaccaatgcgcctactgcaa
Pmv10         gttattggtcagagacaggatagacaggggggagagcggaggaggccccaacttgataaggaccaatgcgcctactgcaa
Pmv11         gttattggtcagagacaggatagacaggggggagagcggaggaggccccaacttgataaggaccaatgcgcctactgcaa
Pmv12         gttattggtcagagacaggatagacaggggggagagcggaggaggccccaacttgataaggaccaatgcgcctactgcaa
Pmv13         gttattggtcagagacaggatagacaggggggagagcggaggaggccccaacttgataaggaccaatgcgcctactgcaa
Pmv14         gttattggtcagagacaggatagacaggggggagagcggaggaggccccaacttgataaggaccaatgcgcctactgcaa
Pmv15         gttattggtcagagacaggatagacaggggggagagcggaggaggccccaacttgataaggaccaatgcgcctactgcaa
Pmv16         gttattggtcagagacaggatagacaggggggagagcggaggaggccccaacttgataaggaccaatgcgcctactgcaa
Pmv17         gttattggtcagagacaggatagacaggggggagagcggaggaggccccaacttgataaggaccaatgcgcctactgcaa
Pmv18         gttattggtcagagacaggatagacaggggggagagcggaggaggccccaacttgataaggaccaatgcgcctactgcaa
Pmv19         gttattggtcagagacaggatagacaggggggagagcggaggaggccccaacttgataaggaccaatgcgcctactgcaa
Pmv2          gttattggtcagagacaggatagacaggggggagagcggaggaggccccaacttgataaggaccaatgcgcctactgcaa
Pmv20         gttattggtcagagacaggatagacaggggggagagcggaggaggccccaacttgataaggaccaatgcgcctactgcaa
Pmv21         gttattggtcagagacaggatagacaggggggagagcggaggaggccccaacttgataaggaccaatgcgcctactgcaa
Pmv22         gttattggtcagagacaggatagacaggggggagagcggaggaggccccaacttgataaggaccaatgcgcctactgcaa
Pmv23         gttattggtcagagacaggatagacaggggggagagcggaggaggccccaacttgataaggaccaatgcgcctactgcaa
Pmv24         gttattggtcagagacaggatagacaggggggagagcggaggaggccccaacttgataaggaccaatgcgcctactgcaa
Pmv4          gttattggtcagagacaggatagacaggggggagagcggaggaggccccaacttgataaggaccaatgcgcctactgcaa
Pmv5          gttattggtcagagacaggatagacaggggggagagcggaggaggccccaacttgataaggaccaatgcgcctactgcaa
Pmv6          gttattggtcagagacaggatagacaggggggagagcggaggaggccccaacttgataaggaccaatgcgcctactgcaa
Pmv7          gttattggtcagagacaggatagacaggggggagagcggaggaggccccaacttgataaggaccaatgcgcctactgcaa
Pmv8          gttattggtcagagacaggatagacaggggggagagcggaggaggccccaacttgataaggaccaatgcgcctactgcaa
Pmv9          gttattggtcagagacaggatagacaggggggagagcggaggaggccccaacttgataaggaccaatgcgcctactgcaa

PMV_CONS      AGAAAAGGGACACTGGGCTAAGGACTGCCCAAAGAAGCCACGAGGGCCCCGAGGACTGAGGCCCCAGACCTCCCTCCTAA
Pmv1          agaaaagggacactgggctaaggactgcccaaagaagccacgagggccccgaggactgaggccccagacctccctcctaa
Pmv10         agaaaagggacactgggctaaggactgcccaaagaagccacgagggccccgaggactgaggccccagacctccctcctaa
Pmv11         agaaaagggacactgggctaaggactgcccaaagaagccacgagggccccgaggactgaggccccagacctccctcctaa
Pmv12         agaaaagggacactgggctaaggactgcccaaagaagccacgagggccccgaggactgaggccccagacctccctcctaa
Pmv13         agaaaagggacactgggctaaggactgcccaaagaagccacgagggccccgaggactgaggccccagacctccctcctaa
Pmv14         agaaaagggacactgggctaaggactgcccaaagaagccacgagggccccgaggactgaggccccagacctccctcctaa
Pmv15         agaaaagggacactgggctaaggactgcccaaagaagccacgagggccccgaggactgaggccccagacctccctcctaa
Pmv16         aAaaaagggacactgggctaaggactgcccaaagaagccacgagggccccgaggactgaggccccagacctccctcctaa
Pmv17         agaaaagggacactgggctaaggactgcccaaagaagccacgagggccccgaggactgaggccccagacctccctcctaa
Pmv18         agaaaagggacactgggctaaggactgcccaaagaagccacgagggccccgaggactgaggccccagacctccctcctaa
Pmv19         agaaaagggacactgggctaaggactgcccaaagaagccacgagggccccgaggactgaggccccagacctccctcctaa
Pmv2          agaaaagggacactgggctaaggactgcccaaagaagccacgagggccccgaggactgaggccccagacctccctcctaa
Pmv20         agaaaagggacactgggctaaggactgcccaaagaagccacgagggccccgaggactgaggccccagacctccctcctaa
Pmv21         agaaaagggacactgggctaaggactgcccaaagaagccacgagggccccgaggactgaggccccagacctccctcctaa
Pmv22         agaaaagggacactgggctaaggactgcccaaagaagccacgagggccccgaggactgaggccccagacctccctcctaa
Pmv23         agaaaagggacactgggctaaggactgcccaaaAaagccacgagggccccgaggactgaggccccagacctccctcctaa
Pmv24         agaaaagggacactgggctaaggactgcccaaagaagccacgagggccccgaggactgaggccccagacctccctcctaa
Pmv4          agaaaagggacactgggctaaggactgcccaaagaagccacgagggccccgaggactgaggccccagacctccctcctaa
Pmv5          agaaaagggacactgggctaaggactgcccaaagaagccacgagggccccgaggactgaggccccagacctccctcctaa
Pmv6          agaaaagggacactgggctaaggactgcccaaagaagccacgagggccccgaggactgaggccccagacctccctcctaa
Pmv7          agaaaagggacactgggctaaggactgcccaaagaagccacgagggccccgaggactgaggccccagacctccctcctaa
Pmv8          agaaaagggacactgggctaaggactgcccaaagaagccacgagggccccgaggactgaggccccagacctccctcctaa
Pmv9          agaaaagggacactgggctaaggactgcccaaagaagccacgagggccccgaggactgaggccccagacctccctcctaa
```

```
PMV_CONS     CCTTAGGTGACGCTCAGGTTATGGGACCAATGGGACTGCCCCTGCAAGTGCTGACCCTAAACATAGAAGATGAGTATCGG
Pmv1         ccttaggtgacgctcaggttatgggaccaatgggactgcccctgcaagtgctgaccctaaacatagaagatgagtatcgg
Pmv10        ccttaggtgacgctcaggttatgggaccaatgggactgcccctgcaagtgctgaccctaaacatagaagatgagtatcgg
Pmv11        ccttaggtgacgctcaggttatgggaccaatgggactgcccctgcaagtgctgaccctaaacatagaagatgagtatcgg
Pmv12        ccttaggtgacgcCcaggttatgggaccaatgggactgcccctgcaagtgctgaccctaaacatagaagatgagtatcgg
Pmv13        ccttaggtgacgctcaggttatgggaccaatgggactgcccctgcaagtgctgaccctaaacatagaagatgagtatcgg
Pmv14        ccttaggtgacgctcaggttatgggaccaatgggactgcccctgcaagtgctgaccctaaacatagaagatgagtatcAg
Pmv15        ccttaggtgacgctcaggttatgggaccaatgggactgcccctgcaagtgctgaccctaaacatagaagatgagtatcgg
Pmv16        ccttaggtgacgctcaggttatgggaccaatgggactgcccctgcaagtgctgaccctaaacatagaagatgagtatcgg
Pmv17        ccttaggtgac---------------------------------------------------------------------
Pmv18        ccttaggtgacgctcaggttatgggaccaatgggactgcccctgcaagtgctgaccctaaacatagaagatgaAtatcgg
Pmv19        ccttaggtgacgctcaggttatgggaccaatgggactgcccctgcaagtgctgaccctaaacatagaagatgagtatcgg
Pmv2         ccttaggtgacgctcaggttatgggaccaatgggactgcccctgcaagtgctgaccctaaacatagaagatgagtatcgg
Pmv20        ccttaggtgacgctcaggttatgggaccaatgggactgcccctgcaagtgctgaccctaaacatagaagatgagtatcgg
Pmv21        ccttaggtgacgctcaggttatgggaccaatgggactgcccctgcaagtgctgaccctaaacatagaagatgagtatcgg
Pmv22        ccttaggtgacgctcaggttatgggaccaatgggactgcccctgcaagtgctgaccctaaacatagaagatgagtatcgg
Pmv23        ccttaggtgacgctcaggttatgggaccaatgggactgcccctgcaagtgctgaccctaaacatagaagatgagtatcgg
Pmv24        ccttaggtgacgctcaggttatgggaccaatgggactgcccctgcaagtgctgaccctaaacatagaagatgagtatcgg
Pmv4         ccttaggtgacgctcaggttatgggaccaatgAgactgcccctgcaagtgctgaccctaaacatagaagatgagtatcgg
Pmv5         ccttaggtgacgctcaggttatgggaccaatgggactgcccctgcaagtgctgaccctaaacatagaagatgagtatcgg
Pmv6         ccttaggtgacgctcaggttatgggaccaatgggactgcccctgcaagtgctgaccctaaacatagaagatgaAtatcgg
Pmv7         ccttaggtgacgctcaggttatgggaccaatgggactgcccctgcaagtgctgaccctaaacatagaagatgagtatcgg
Pmv8         ccttaggtgacgctcaggttatgggaccaatgggactgcccctgcaagtgctgaccctaaacatagaagatgagtatcgg
Pmv9         ccttaggtgacgctcaggttGtgggaccaatgggacAgcccctgcaagtgctgaccctaaacatagaagatgagtatcgg
```

```
PMV_CONS      CTACATGAGACCTCAAAAGAGCCGGATGTTTCTCTAGGGTCCACCTGGCTTTCTGATTTTCCCCAGGTCTGGGCGGAAAC
Pmv1          ctacatgagacctcaaaagagccggatgtttctctagggtccacctggctttctgattttccccaggtctgggcggaaac
Pmv10         ctacatgagacctcaaaagagccggatgtttctctagggtccacctggctttctgattttccccaggtctgggcggaaac
Pmv11         ctacatgagacctcaaaagagccggatgtttctctagggtccacctggctttctgattttccccaggtctgggcggaaac
Pmv12         ctacatgagacctcaaaagagccggatgtttctctagggtccacctggctttctgattttccccaggtctgggcggaaac
Pmv13         ctacatgagacctcaaaagagccggatgtttctctagggtccacctggctttctgattttccccaggtctgggcggaaac
Pmv14         ctacatgagacctcaaaagagccggatgtttctctagggtccacctggctttctgattttccccaggtctgggcggaaac
Pmv15         ctacatgagacctcaaaagagccggatgtttctctagggtccacctggctttctgattttccccaggtctgggcggaaac
Pmv16         ctacatgagacctcaaaagagccggatgtttctctagggtccacctggctttctgattttccccaggtctgggcggaaac
Pmv17         --------------------------------------------------------------------------------
Pmv18         ctacatgagacctcaaaagagccggatgtttctctagggtccacctggctttctgattttccccaggtctgggcggaaac
Pmv19         ctacatgagacctcaaaagagccggatgtttctctagggtccacctggctttctgattttccccaggtctgggcggaaac
Pmv2          ctacatgagacctcaaaagagccggatgtttctctagggtccacctggctttctgattttccccaggtctgggcggaaac
Pmv20         ctacatgagacctcaaaagagccggatgtttctctagggtccacctggctttctgattttccccaggtctgggcggaaac
Pmv21         ctacatgagacctcaaaagagccggatgtttctctagggtccacctggctttctgattttccccaggtctgggcggaaac
Pmv22         ctacatgagacctcaaaagagccggatgtttctctagggtccacctggctttctgattttccccaggtctgggcggaaac
Pmv23         ctacatgagacctcaaaagagccggatgtttctctagggCccacctggctttctgattttccccaggtctgggcggaaac
Pmv24         ctacatgagacctcaaaagagccggatgtttctctagggtccacctggctttctgattttccccaggtctgggcggaaac
Pmv4          ctacatgagacctcaaaagagccggatgtttctctagggtccacctggctttctgattttccccaggtctgggcAAaaac
Pmv5          ctacatgagacctcaaaagagccggatgtttctctagggtccacctggctttctgattttccccaggtctgggcggaaac
Pmv6          ctacatgagacctcaaaagagccggatgtttctctagggtccacctggctttctgattttccccaggtctgggcggaaac
Pmv7          ctacatgagacctcaaaagagccggatgtttctctagggtccacctggctttctgattttccccaggtctgggcggaaac
Pmv8          ctacatgagacctcaaaagagccggatgtttctctagggtccacctggctttctgattttccccaggtctgggcggaaac
Pmv9          ctacatgagacctcaaaagagccggatgtttctctagggtccacAtggctttctgattttccccaggCctgggcggaaac

PMV_CONS      CGGGGGCATGGGACTGGCAGTTCGCCAAGCTCCTCTGATCATACCTCTGAAGGCAACCTCTACCCCCGTGTCCATAAAAC
Pmv1          cgggggcatgggactggcagttcgccaagctcctctgatcatacctctgaaggcaacctctacccccgtgtccataaaac
Pmv10         cgggggcatgggactggcagttcgccaagctcctctgatcatacctctgaaggcaacctctacccccgtgtccataaaac
Pmv11         cgggggcatgggactggcagttcgccaagctcctctgatcatacctctgaaggcaacctctacccccgtgtccataaaac
Pmv12         cgggggcatgggactggcagttcgccaagctcctctgatcatacctctgaaggcaacctctacccccgtgtccataaaac
Pmv13         cgggggcatgggactggcagttcgccaagctcctctgatcatacctctgaaggcaacctctacccccgtgtccataaaac
Pmv14         cgggggcatgggactggcagttcgccaagctcctctgatcatacctctgaaggcaacctctacccccgtgtccataaaac
Pmv15         cgggggcatgggactggcagttcgccaagctcctctgatcatacctctgaaggcaacctctacccccgtgtccataaaac
Pmv16         cgggggcatgggactggcagttcgccaagctcctctgatcatacctctgaaggcaacctctacccccgtgtccataaaac
Pmv17         --------------------------------------------------------------------------------
Pmv18         cgggggcatgggactggcagttcgccaagctcctctgatcatacctctgaaggcaacctctacccccgtgtccataaaac
Pmv19         cgggggcatgggactggcagttcgccaagctcctctgatcatacctctgaaggcaacctctacccccgtgtccataaaac
Pmv2          cgggggcatgggactggcagttcgccaagctcctctgatcatacctctgaaggcaacctctacccccgtgtccataaaac
Pmv20         cgggggcatgggactggcagttcgccaagctcctctgatcatacctctgaaggcaacctctacccccgtgtccataaaac
Pmv21         cgggggcatgggactggcagttcgccaagctcctctgatcatacctctgaaggcaacctctacccccgtgtccataaaac
Pmv22         cggAggcatgggactggcagttcgccaagctcctctgatcatacctctgaaggcaacctctacccccgtgtccataaaac
Pmv23         cgggggcatgggactggcagttcgccaagctcctctgatcatacctctgaaggcaacctctacccccgtgtccataaaac
Pmv24         cgggggcatgggactggcagttcgccaagctcctctgatcatacctctgaaggcaacctctacccccgtgtccataaaac
Pmv4          cgggggcatgggactggcagttcgccaagctcctctgatcatacctctAaaggcaacctctacccccgtgtccataaaac
Pmv5          cgggggcatgggactggcagttcgccaagctcctctgatcatacctctgaaggcaacctctacccccgtgtccataaaac
Pmv6          cgggggcatgggactggcagttcgccaagctcctctgatcatacctctgaaggcaacctctacccccgtgtccataaaac
Pmv7          cgggggcatgggactggcagttcgccaagctcctctgatcatacctctgaaggcaacctctacccccgtgtccataaaac
Pmv8          cgggggcatgggactggcagttcgccaagctcctctgatcatacctctgaaggcaacctctacccccgtgtccataaaac
Pmv9          cgggggcatgggactggcagttcgccaagctcctctgatcatacctctgaaggcaacctctacccccgtgtccataaaac
```

```
PMV_CONS    AATACCCCATGTCACAAGAAGCCAGACTGGGGATCAAGCCCCACATACAGAGACTGTTGGACCAGGGAATACTGGTACCC
Pmv1        aataccccatgtcacaagaagccagactggggatcaagccccacatacagagactgttggaccagggaatactggtaccc
Pmv10       aataccccatgtcacaagaagccagactggggatcaagccccacatacagagactgttggaccagggaatactggtaccc
Pmv11       aataccccatgtcacaagaagccagactggggatcaagccccacatacagagactgttggaccagggaatactggtaccc
Pmv12       aataccccatgtcacaagaagccagactggggatcaagccccacatacagagactgttggaccagggaatactggtaccc
Pmv13       aataccccatgtcacaagaagccagactggggatcaagccccacatacagagactgttggaccagggaatactggtaccc
Pmv14       aataccccatgtcacaagaagccagactggggatcaagccccacatacagagactgttggaccagggaatactggtaccc
Pmv15       aataccccatgtcacaagaagccagactggggatcaagccccacatacagagactgttggaccagggaatactggtaccc
Pmv16       aataccccatgtcacaagaagccagactggggatcaagccccacatacagagactgttggaccagggaatactggtaccc
Pmv17       --------------------------------------------------------------------------------
Pmv18       aataccccatgtcacaagaagccagactggggatcaagccccacatacagagactgttggaccagggaatactggtaccc
Pmv19       aataccccatgtcacaagaagccagactggggatcaagccccacatacagagactgttggaccagggaatactggtaccc
Pmv2        aataccccatgtcacaagaagccagactggggatcaagccccacatacagagactgttggaccagggaatactggtaccc
Pmv20       aataccccatgtcacaagaagccagactggggatcaagccccacatacagagactgttggaccagggaatactggtaccc
Pmv21       aataccccatgtcacaagaagccagactggggatcaagccccacatacagagactgttggaccagggaatactggtaccc
Pmv22       aataccccatgtcacaagaagccagactggggatcaagccccacatacagagactgttggaccagggaatactggtaccc
Pmv23       aataccccatgtcacaagaagccagactggggatcaagccccacatacagagactgttggaccagggaatactggtaccc
Pmv24       aataccccatgtcacaagaagccagactggggatcaagccccacatacagagactgttggaccagggaatactggtaccc
Pmv4        aataccccatgtcacaagaagccagactggggatcaagccccacatacagagactgttggaccagggaatactggtaccc
Pmv5        aataccccatgtcacaagaagccagactggggatcaagccccacatacagagactgttggaccagggaatactggtaccc
Pmv6        aataccccatgtcacaagaagccagactggggatcaagccccacatacagagactgttggaccagggaatactggtaccc
Pmv7        aataccccatgtcacaagaagccagactggggatcaagccccacatacagagactgttggaccagggaatactggtaccc
Pmv8        aataccccatgtcacaagaagccagactggggatcaagccccacatacagagactgttggaccagggaatactggtaccc
Pmv9        aataccccatgtcacaagaagccagactggggatcaagccccacatacagagactgttggaccagggaatactggtaccc

PMV_CONS    TGCCAGTCCCCCTGGAACACGCCCCTGCTACCCGTTAAGAAACCAGGGACTAATGATTATAGGCCTGTCCAGGATCTGAG
Pmv1        tgccagtccccctggaacacgcccctgctacccgttaagaaaccagggactaatgattataggcctgtccaggatctgag
Pmv10       tgccagtccccctggaacacgcccctgctacccgttaagaaaccagggactaatgattataggcctgtccaggatctgag
Pmv11       tgccagtccccctggaacacgcccctgctacccgttaagaaaccagggactaatgattataggcctgtccaggatctgag
Pmv12       tgccagtccccctggaacacgcccctgctacccgttaagaaaccagggactaatgattataggcctgtccaggatctgag
Pmv13       tgccagtccccctggaacacgcccctgctacccgttaagaaaccagggactaatgattataggcctgtccaggatctgag
Pmv14       tgccagtccccctggaacacgcccctgctacccgttaagaaaccagggactaatgattataggcctgtccaggatctgag
Pmv15       tgccagtccccctggaacacgcccctgctacccgttaagaaaccagggactaatgattataggcctgtccaggatctgag
Pmv16       tgccagtccccctggaacacgcccctgctacccgttaagaaaccagggactaatgattataggcctgtccaggatctgag
Pmv17       --------------------------------------------------------------------------------
Pmv18       tgccagtccccctggaacacgcccctgctacccgttaagaaaccagggactaatgattataggcctgtccaggatctgag
Pmv19       tgccagtccccctggaacacgcccctgctacccgttaagaaaccagggactaatgattataggcctgtccaggatctgag
Pmv2        tgccagtccccctggaacacgcccctgctacccgttaagaaaccagggactaatgattataggcctgtccaggatctgag
Pmv20       tgccagtccccctggaacacgcccctgctacccgttaagaaaccagggactaatgattataggcctgtccaggatctgag
Pmv21       tgccagtccccctggaacacgcccctgctacccgttaagaaaccagggactaatgattataggcctgtccaggatctgag
Pmv22       tgccagtccccctggaacacgcccctgctacccgttaagaaaccagggactaatgattataggcctgtccaggatctgag
Pmv23       tgccagtccccctggaacacgcccctgctacccgttaagaaaccagggactaatgattataggcctgtccaggatctgag
Pmv24       tgccagtccccctggaacacgcccctgctacccgttaagaaaccagggactaatgattataggcctgtccaggatctgag
Pmv4        tgccagtccccctgAaacacgcccctgctacccgttaaAaaaccagggactaatgattataggcctgtccaggatctgag
Pmv5        tgccagtccccctggaacacgcccctgctacccgttaagaaaccagggactaatgattataggcctgtccaggatctgag
Pmv6        tgccagtccccctggaacacgcccctgctacccgttaagaaaccagggactaatgattataggcctgtccaggatctgag
Pmv7        tgccagtccccctggaacacgcccctgctacccgttaagaaaccagggactaatgattataggcctgtccaggatctgag
Pmv8        tgccagtccccctggaacacgcccctgctacccgttaagaaaccagggactaatgattataggcctgtccaggatctgag
Pmv9        tgccagtccccctggaacacgcccctgctacccgttaagaaaccagggactaatgattataggcctgtccaggatctgag
```

```
PMV_CONS      AGAAGTCAACAAGCGGGTGGAAGACATCCACCCCACCGTGCCCAACCCTTACAACCTCTTGAGCGGGCTCCCACCGTCCC
Pmv1          agaagtcaacaagcgggtggaagacatccaccccaccgtgcccaacccttacaacctcttgagcgggctcccaccgtccc
Pmv10         agaagtcaacaagcgggtggaagacatccaccccaccgtgcccaacccttacaacctcttgagcgggctcccaccgtccc
Pmv11         agaagtcaacaagcgggtggaagacatccaccccaccgtgcccaacccttacaacctcttgagcgggctcccaccgtccc
Pmv12         agaagtcaacaagcgggtggaagacatccaccccaccgtgcccaacccttacaacctcttgagcgggctcccaccgtccc
Pmv13         agaagtcaacaagcgggtggaagacatccaccccaccgtgcccaacccttacaacctcttgagcgggctcccaccgtccc
Pmv14         agaagtcaacaagcgggtggaagacatccaccccaccgtgcccaacccttacaacctcttgagcgggctcccaccgtccc
Pmv15         agaagtcaacaagcgggtggaagacatccaccccaccgtgcccaacccttacaacctcttgagcgggctcccaccgtccc
Pmv16         agaagtcaacaagcgggtggaagacatccaccccaccgtgcccaacccttacaacctcttgagcgggctcccaccgtccc
Pmv17         --------------------------------------------------------------------------------
Pmv18         agaagtcaacaagcgggtggaagacatccaccccaccgtgcccaacccttacaacctcttgagcgggctcccaccgtccc
Pmv19         agaagtcaacaagcgggtggaagacatccaccccaccgtgcccaacccttacaacctcttgagcgggctcccaccgtccc
Pmv2          agaagtcaacaagcgggtggaagacatccaccccaccgtgcccaacccttacaacctcttgagcgggctcccaccgtccc
Pmv20         agaagtcaacaagcgggtggaagacatccaccccaccgtgcccaacccttacaacctcttgagcgggctcccaccgtccc
Pmv21         agaagtcaacaagcgggtggaagacatccaccccaccgtgcccaacccttacaacctcttgagcgggctcccaccgtccc
Pmv22         agaagtcaacaagcgggtggaagacatccaccccaccgtgcccaacccttacaacctcttgagcgggctcccaccgtccc
Pmv23         agaagtcaacaagcgggtggaagacatccaccccaccgtgcccaacccttacaacctcttgagcgggctcccaccgtccc
Pmv24         agaagtcaacaagcgggtggaagacatccaccccaccgtgcccaacccttacaacctcttgagcgggctcccaccgtccc
Pmv4          aAaagtcaacaagcgggtAAaagacatccaccccaccgtgcccaacccttacaacctcttgagcgggctcccaccgtccc
Pmv5          agaagtcaacaagcgggtggaagacatccaccccaccgtgcccaacccttacaacctcttgagcgggctcccaccgtccc
Pmv6          agaagtcaacaagcgggtggaagacatccaccccaccgtgcccaacccttacaacctcttgagcgggctcccaccgtccc
Pmv7          agaagtcaacaagcgggtggaagacatccaccccaccgtgcccaacccttacaacctcttgagcgggctcccaccgtccc
Pmv8          agaagtcaacaagcgggtggaagacatccaccccaccgtgcccaacccttacaacctcttgagcgggctcccaccgtccc
Pmv9          agaagtcaacaagcgggtggaagacatccaccccaccgtgcccaacccttacaacctcttgagcgggctcccaccgtccc

PMV_CONS      ACCAGTGGTACACTGTGCTTGATTTAAAGGATGCCTTTTTCTGCCTGAGACTCCACCCCACCAGTCAGCCTCTCTTCGCC
Pmv1          accagtggtacactgtgcttgatttaaaggatgcctttttctgcctgagactccaccccaccagtcagcctctcttcgcc
Pmv10         accagtggtacactgtgcttgatttaaaggatgcctttttctgcctgagactccaccccaccagtcagcctctcttcgcc
Pmv11         accagtggtacactgtgcttgatttaaaggatgcctttttctgcctgagactccaccccaccagtcagcctctcttcgcc
Pmv12         accagtggtacactgtgcttgatttaaaggatgcctttttctgcctgagactccaccccaccagtcagcctctcttcgcc
Pmv13         accagtggtacactgtgcttgatttaaaggatgcctttttctgcctgagactccaccccaccagtcagcctctcttcgcc
Pmv14         accagtggtacactgtgcttgatttaaaggatgcctttttctgcctgagactccaccccaccagtcagcctctcttcgcc
Pmv15         accagtggtacactgtgcttgatttaaaggatgcctttttctgcctgagactccaccccaccagtcagcctctcttcgcc
Pmv16         accagtggtacactgtgcttgatttaaaggatgcctttttctgcctgagactccaccccaccagCcagcctctcttcgcc
Pmv17         --------------------------------------------------------------------------------
Pmv18         accagtggtacactgtgcttgatttaaaggatgcctttttctgcctgagactccaccccaccagtcagcctctcttcAcc
Pmv19         accagtggtacactgtgcttgatttaaaggatgcctttttctgcctgagactccaccccaccagtcagcctctcttcgcc
Pmv2          accagtggtacactgtgcttgatttaaaggatgcctttttctgcctgagactccaccccaccagtcagcctctcttcgcc
Pmv20         accagtggtacactgtgcttgatttaaaggatgcctttttctgcctgagactccaccccaccagtcagcctctcttcgcc
Pmv21         accagtggtacactgtgcttgatttaaaggatgcctttttctgcctgagactccaccccaccagtcagcctctcttcgcc
Pmv22         accagtggtacactgtgcttgatttaaaggatgcctttttctgcctgagactccaccccaccagtcagcctctcttcgcc
Pmv23         accagtggtacactgtgcttgatttaaaggatgcctttttctgcctgagactccaccccaccagtcagcctctcttcgcc
Pmv24         accagtggtacactgtgcttgatttaaaggatgcctttttctgcctgagactccaccccaccagtcagcctctcttcgcc
Pmv4          accagtAgtacactgtgcttAatttaaaggatgcctttttctgcctgagactccaccccaccagtcagcctctcttcgcc
Pmv5          accagtggtacactgtgcttgatttaaaggatgcctttttctgcctgagactccaccccaccagtcagcctctcttcgcc
Pmv6          accagtggtacactgtgcttgatttaaaggatgcctttttctgcctgagactccaccccaccagtcagcctctcttcgcc
Pmv7          accagtggtacactgtgcttgatttaaaggatgcctttttctgcctgagactccaccccaccagtcagcctctcttcgcc
Pmv8          accagtggtacactgtgcttgatttaaaggatgcctttttctgcctgagactccaccccaccagCcagcctctcttcgcc
Pmv9          accagtggtacactgtgcttgaCttaaaggatgcctttttctgcctgagactccaccccaccagtcagcctctcttcgcc
```

```
PMV_CONS      TTTGAGTGGAGAGATCCAGAGATGGGAATCTCAGGACAATTGACCTGGACCAGACTCCCACAGGGTTTCAAAAACAGTCC
Pmv1          tttgagtggagagatccagagatgggaatctcaggacaattgacctggaccagactcccacagggtttcaaaaacagtcc
Pmv10         tttgagtggagagatccagagatgggaatctcaggacaattgacctggaccagactcccacagggtttcaaaaacagtcc
Pmv11         tttgagtggagagatccagagatgggaatctcaggacaattgacctggaccagactcccacagggtttcaaaaacagtcc
Pmv12         tttgagtggagagatccagagatgggaatctcaggacaattgacctggaccagactcccacagggtttcaaaaacagtcc
Pmv13         tttgagtggagagatccagagatgggaatctcaggacaattgacctggaccagactcccacagggtttcaaaaacagtcc
Pmv14         tttgagtggagagatccagagatgggaatctcaggacaattgacctggaccagactcccacagggtttcaaaaacagtcc
Pmv15         tttgagtggagagatccagagatgggaatctcaggacaattgacctggaccagactcccacagggtttcaaaaacagtcc
Pmv16         tttgagtggagagatccagagatgggaatctcaggacaattgacctggaccagactcccacagggtttcaaaaacagtcc
Pmv17         --------------------------------------------------------------------------------
Pmv18         tttgagtggagagatccagagatgAgaatctcaggacaattgacctggaccagactcccacagggtttcaaaaacagtcc
Pmv19         tttgagtggagagatccagagatgggaatctcaggacaattgacctggaccagactcccacagggtttcaaaaacagtcc
Pmv2          tttgagtAgagagatccagagatgggaatctcaggacaattgacctggaccagactcccacagggtttcaaaaacagtcc
Pmv20         tttgagtggagagatccagagatgggaatctcaggacaattgacctggaccagactcccacagggtttcaaaaacagtcc
Pmv21         tttgagtggagagatccagagatgggaatctcaggacaattgacctggaccagactcccacagggtttcaaaaacagtcc
Pmv22         tttgagtggagagatccagagatgggaatctcaggacaattgacctggaccagactcccacagggtttcaaaaacagtcc
Pmv23         tttgagtggagagatccagagatgggaatctcaggacaattgacctggaccagactcccacagggtttcaaaaacagtcc
Pmv24         tttgagtggagagatccagagatgggaatctcaggacaattgacctggaccagactcccacagggtttcaaaaacagtcc
Pmv4          tttgagtggagagatccagagatgggaatctcaggacaattgacctggaccagactcccacagggtttcaaaaacagtcc
Pmv5          tttgagtggagagatccagagatgggaatctcaggacaattgacctggaccagactcccacagggtttcaaaaacagtcc
Pmv6          tttgagtggagagatccagagatgggaatctcaggacaattgacctggaccagactcccacagggtttcaaaaacagtcc
Pmv7          tttgagtggagagatccagagatgggaatctcaggacaattgacctggaccagactcccacagggtttcaaaaacagtcc
Pmv8          tttgagtggagagatccagagatgggaatctcaggacaattgacctggaccagactcccacagggtttcaaaaacagtcc
Pmv9          tttgagtggagagaCccagagatgggaatctcaggacaattAacctggaccagactcccacagggtttcaaaaacagtcc

PMV_CONS      CACCCTGTTTGATGAGGCACTGCACAGAGACCTAGCAGACTTCCGGATCCAGCACCCAGACTTGATCCTGCTACAGTACG
Pmv1          caccctgtttgatgaggcactgcacagagacctagcagacttccggatccagcacccagacttgatcctgctacagtacg
Pmv10         caccctgtttgatgaggcactgcacagagacctagcagacttccggatccagcacccagacttgatcctgctacagtacg
Pmv11         caccctgtttgatgaggcactgcacagagacctagcagacttccggatccagcacccagacttgatcctgctacagtacg
Pmv12         caccctgtttgatgaggcactgcacagagacctagcagacttccggatccagcacccagacttgatcctgctacagtacg
Pmv13         caccctgtttgatgaggcactgcacagagacctagcagacttccggatccagcacccagacttgatcctgctacagtacg
Pmv14         caccctgtttgatgaggcactgcacagagacctagcagacttccggatccagcacccagacttgatcctgctacagtacg
Pmv15         caccctgtttgatgaggcactgcacagagacctagcagacttccggatccagcacccagacttgatcctgctacagtacg
Pmv16         caccctgtttgatgaggcactgcacagagacctagcagacttccggatccagcacccagacttgatcctgctacagtacg
Pmv17         --------------------------------------------------------------------------------
Pmv18         caccctgtttgatgaggcactgcacagagacctagcagacttccggatccagcacccagacttgatcctgctacagtacg
Pmv19         caccctgtttgatgaggcactgcacagagacctagcagacttccggatccagcacccagacttgatcctgctacagtacg
Pmv2          caccctgtttgatgaggcactgcacagagacctagcagacttccggatccagcacccagacttgatcctgctacagtacg
Pmv20         caccctgtttgatgaggcactgcacagagacctagcagacttccggatccagcacccagacttgatcctgctacagtacg
Pmv21         caccctgtttgatgaggcactgcacagagacctagcagacttccggatccagcacccagacttgatcctgctacagtacg
Pmv22         caccctgtttgatgaggcactgcacagagacctagcagacttccggatccagcacccagacttgatcctgctacagtacg
Pmv23         caccctgtttgatgaggcactgcacagagacctagcagacttccAgatccagcacccagacttgatcctgctacagtacg
Pmv24         caccctgtttgatgaggcactgcacagagacctagcagacttccggatccagcacccagacttgatcctgctacagtacg
Pmv4          caccctgtttgatgaggcactgcacagagacctagcagacttccggatccagcacccagacttgatcctgctacagtacg
Pmv5          caccctgtttgatgaggcactgcacagagacctagcagacttccggatccagcacccagacttgatcctgctacagtacg
Pmv6          caccctgtttgatgaggcactgcacagagacctagcagacttccggatccagcacccagacttgatcctgctacagtacg
Pmv7          caccctgtttgatgaggcactgcacagagacctagcagacttccggatccagcacccagacttgatcctgctacagtacg
Pmv8          caccctgtttgatgaggcactgcacagagacctagcagacttccggatccagcacccagacttgatcctgctacagtacg
Pmv9          caccctgtttgatgaggcactgcacagagacctagcagGcttccggatccagcacccagacttgatcctgctacagtacg
```

```
PMV_CONS    TGGATGACTTACTGCTGGCCGCCACTTCTGAGCTCGACTGCCAACAAGGTACTCGGGCCCTGTTACAAACCCTAGGGAAC
Pmv1        tggatgacttactgctggccgccacttctgagctcgactgccaacaaggtactcgggccctgttacaaaccctagggGac
Pmv10       tggatgacttactgctggccgccacttctgagctAgactgccaacaaggtactcgggccctgttacaaaccctagggaac
Pmv11       tggatgacttactgctggccgccacttctgagctcgactgccaacaaggtactcgggccctgttacaaaccctagggGac
Pmv12       tggatgacttactgctggccgccacttctgagctcgactgccaacaaggtactcgggccctgttacaaaccctagggaac
Pmv13       tggatgacttactgctggccgccacttctgagctAgactgccaacaaggtactcgggccctgttacaaaccctagggaac
Pmv14       tggatgacttactgctggccgccacttctgagctAgactgccaacaaggtactcgggccctgttacaaaccctagggaac
Pmv15       tggatgacttactgctggccgccacttctgagctAgactgccaacaaggtactcgggccctgttacaaaccctagAgaac
Pmv16       tggatgacttactgctggccgccacttctgagctcgactgccaacaaggtactcgggccctgttacaaaccctagggaac
Pmv17       --------------------------------------------------------------------------------
Pmv18       tggatgacttactgctggccgccacttctgagctcgactgccaacaaggtactcgggccctgttacaaaccctagggaac
Pmv19       tggatgacttactgctggccgccacttctgagctcgactgccaacaaggtactcgggccctgttacaaaccctagggaac
Pmv2        tggatgacttactgctggccgccacttctgagctcgactgccaacaaggtactcgggccctgttacaaaccctagggaac
Pmv20       tggatgacttactgctggccgccacttctgagctcgactgccaacaaggtactcgggccctgttacaaaccctagggaac
Pmv21       tggatgacttactgctggccgccacttctgagctcgactgccaacaaggtactcgggccctgttacaaaccctagggaac
Pmv22       tggatgacttactgctggccgccacttctgagctcgactgccaacaaggtactcgggccctgttacaaaccctagggaac
Pmv23       tggatgacttactgctggccgccacttctgagctAgactgccaacaaggtactcgggccctgttacaaaccctagggaac
Pmv24       tggatgacttactgctggccgccacttctgagctcgactgccaacaaggtactcgggccctgttacaaaccctagggaac
Pmv4        tggatgacttactgctggccgccacttctgagctAgactgccaacaaggtactcgggccctgttGcaaaccctagAgaac
Pmv5        tggatgacttactgctggccgccacttctgagctcgactgccaacaaggtactcgggccctgttacaaaccctagggGac
Pmv6        tggatgacttactgctggccgccacttctgagctcgactgccaacaaggtactcgggccctgttacaaaccctagggaac
Pmv7        tggatgacttactgctggccgccacttctgagctcgactgccaacaaggtactcgggccctgttacaaaccctagggaac
Pmv8        tggatgacttactgctggccgccacttctgagctcgactgccaacaaggtactcgggccctgttacaaaccctagggaac
Pmv9        tggatgacttactgctggccgccacttctgagctcgactgccaacaaggtactcgggccctgttacaaaccctagggGac

PMV_CONS    CTCGGGTATCGGGCCTCGGCCAAGAAAGCCCAAATTTGCCAGAAACAGGTCAAGTATCTGGGGTATCTTCTAAAAGAGGG
Pmv1        ctcgggtatcgggcctcggccaagaaagcccaaatttgccagaaacaggtcaagtatctggggtatcttctaaaagaggg
Pmv10       ctcgggtatcgggcctcggccaagaaagcccaaatttgccagaaacaggtcaagtatctggggtatcttctaaaagaggg
Pmv11       ctcgggtatcgggcctcggccaagaaagcccaaatttgccagaaacaggtcaagtatctggggtatcttctaaaagaggg
Pmv12       ctcgggtatcgggcctcggccaagaaagcccaaatttgccagaaacaggtcaagtatctggggtatcttctaaaagaggg
Pmv13       ctcgggtatcgggcctcggccaagaaagcccaaatttgccagaaacaggtcaagtatctggggtatcttctaaaagaggg
Pmv14       ctcgggtatcgggcctcggccaagaaagcccaaatttgccagaaacaggtcaagtatctggggtatcttctaaaagaggg
Pmv15       ctcgggtatcgggcctcggccaagaaagcccaaatttgccagaaacaggtcaagtatctggggtatcttctaaaagaggg
Pmv16       ctcgggtatcgggcctcAgccaagaaagcccaaatttgccagaaacaggtcaagtatctggggtatcttctaaaagaggg
Pmv17       --------------------------------------------------------------------------------
Pmv18       ctcgggtatcgggcctcggccaagaaagcccaaatttgccagaaacaggtcaagtatctggggtatcttctaaaagaggg
Pmv19       ctcgggtatcgggcctcggccaagaaagcccaaatttgccagaaacaggtcaagtatctggggtatcttctaaaagaggg
Pmv2        ctcgggtatcgggcctcggccaagaaagcccaaatttgccagaaacaggtcaagtatctggggtatcttctaaaagaggg
Pmv20       ctcgggtatcgggcctcggccaagaaagcccaaatttgccagaaacaggtcaagtatctggggtatcttctaaaagaggg
Pmv21       ctcgggtatcgggcctcggccaagaaagcccaaatttgccagaaacaggtcaagtatctggggtatcttctaaaagaggg
Pmv22       ctcgggtatcgggcctcggccaagaaagcccaaatttgccagaaacaggtcaagtatctggggtatcttctaaaagaggg
Pmv23       ctcgggtatcgggcctcggccaagaaagcccaaatttgccagaaacaggtcaagtatctggggtatcttctaaaagaggg
Pmv24       ctcgggtatcgggcctcggccaagaaagcccaaatttgccagaaacaggtcaagtatctggggtatcttctaaaagaggg
Pmv4        ctcgggtatcgggcctcggccaagaaagcccaaatttgccagaaacaggtcaagtatctggggtatcttctaaaagaggg
Pmv5        ctcgggtatcgggcctcggccaagaaagcccaaatttgccagaaacaggtcaagtatctggggtatcttctaaaagaggg
Pmv6        ctcgggtatcgggcctcggccaagaaagcccaaatttgccagaaacaggtcaagtatctggggtatcttctaaaagaggg
Pmv7        ctcgggtatcgggcctcggccaagaaagcccaaatttgccagaaacaggtcaagtatctggggtatcttctaaaagaggg
Pmv8        ctcgggtatcgggcctcggccaagaaagcccaaatttgccagaaacaggtcaagtatctggggtatcttctaaaagaggg
Pmv9        ctcgggtatcgggcctcggccaagaaGgcccaaatttgccagaaacaggtcaagtatctggggtatcttctaaaagaggg
```

```
PMV_CONS      TCAGAGATGGCTGACTGAGGCCAGAAAAGAGACTGTGATGGGGCAGCCTACTCCGAAGACCCCTCGACAACTAAGGGAGT
Pmv1          tcagagatggctgactgaggccagaaaagagactgtgatggggcagcctactccgaagacccctcgacaactaagggagt
Pmv10         tcagagatggctgactgaggccagaaaagagactgtgatggggcagcctactccgaagacccctcgacaactaagggagt
Pmv11         tcagagatggctgactgaggccagaaaagagactgtgatggggcagcctactccgaagacccctcgacaactaagggagt
Pmv12         tcagagatggctgactgaggccagaaaagagactgtgatgggAcagcctactccgaagacccctcgacaactaagAgagt
Pmv13         tcagagatggctgactgaggccagaaaagagactgtgatggggcagcctactccgaagacccctcgacaactaagggagt
Pmv14         tcagagatggctgactgaggccagaaaagagactgtgatggggcagcctactccgaagacccctcgacaactaagggagt
Pmv15         tcagagatggctgactgaggccagaaaagagactgtgatggggcagcctactccgaagacccctcgacaactaagggagt
Pmv16         tcagagatggctgactgaggccagaaaagagactgtgatggggcagcctactccgaagacccctcgacaactaagggagt
Pmv17         --------------------------------------------------------------------------------
Pmv18         tcagagatggctgactgaggccagaaaagagactgtgatggggcagcctactccgaagacccctcgacaactaagggagt
Pmv19         tcagagatggctgactgaggccagaaaagagactgtgatggggcagcctactccgaagacccctcgacaactaagggagt
Pmv2          tcagagatggctgactgaggccagaaaagagactgtgatggggcagcctactccgaagacccctcgacaactaagggagt
Pmv20         tcagagatggctgactgGggccagaaaagagactgtgatggggcagcctactccgaagacccctcgacaactaagggagt
Pmv21         tcagagatggctgactgaggccagaaaagagactgtgatgggAcagcctactccgaagacccctcgacaactaagAgagt
Pmv22         tcagagatggctgactgaggccagaaaagagactgtgatggggcagcctactccgaagacccctcgacaactaagggagt
Pmv23         tcagagatggctgactgaggccagaaaagagactgtgatggggcagcctactccgaagacccctcgacaactaagggagt
Pmv24         tcagagatggctgactgaggccagaaaagagactgtgatggggcagcctactccgaagacccctcgacaactaagggagt
Pmv4          tcagagatggctgactgaggccagaaaagagactgtgatggggcagcctactccgaagacccctcgacaactaagggagt
Pmv5          tcagagatggctgactgaggccagaaaagagactgtgatggggcagcctactccgaagacccctcgacaactaagggagt
Pmv6          tcagagatggctgactgaggccagaaaagagactgtgatggggcagcctactccgaagacccctcgacaactaagggagt
Pmv7          tcagagatggctgactgaggccagaaaagagactgtgatggggcagcctactccgaagacccctcgacaactaagggagt
Pmv8          tcagagatggctgactgaggccagaaaagagactgtgatggggcagcctactccgaagacccctcgacaactaagggagt
Pmv9          tcagagatggctgactgaggccagaaaagagactgtgatggggcagcctactccgaagacccctcgacaactaagggagt

PMV_CONS      TCCTAGGGACGGCAGGCTTCTGTCGCCTCTGGATCCCTGGGTTTGCAGAAATGGCAGCCCCCTTGTACCCTCTCACCAAA
Pmv1          tcctagggacggcaggcttctgtcgcctctggatccctgggtttgcagaaatggcagcccccttgtaccctctcaccaaa
Pmv10         tcctagggacggcaggcttctgtcgcctctggatccctgggtttgcagaaatggcagcccccttgtaccctctcaccaaa
Pmv11         tcctagggacggcaggcttctgtcgcctctggatccctgggtttgcagaaatggcagcccccttgtaccctctcaccaaa
Pmv12         tcctagggacggcaggcttctgtcgcctctggatccctgggtttgcagaaatggcagcccccttgtaccctctcaccaaa
Pmv13         tcctagggacggcaggcttctgtcgcctctggatccctgggtttgcagaaatggcagcccccttgtaccctctcaccaaa
Pmv14         tcctagggacggcaggcttctgtcgcctctggatccctgggtttgcagaaatggcagcccccttgtaccctctcaccaaa
Pmv15         tcctagggacggcaggcttctgtcgcctctggatccctgggtttgcagaaatggcagcccccttgtaccctctcaccaaa
Pmv16         tcctagggacggcaggcttctgtcgcctctggatccctgggtttgcagaaatggcagcccccttgtaccctctcaccaaa
Pmv17         --------------------------------------------------------------------------------
Pmv18         tcctagggacggcaggcttctgtcgcctctggatccctgggtttgcagaaatggcagcccccttgtaccctctcaccaaa
Pmv19         tcctagggacggcaggcttctgtcgcctctggatccctgggtttgcagaaatggcagcccccttgtaccctctcaccaaa
Pmv2          tcctagggacggcaggcttctgtcgcctctggatcccAgggtttgcagaaatggcagcccccttgtaccctctcaccaaa
Pmv20         tcctagggacggcaggcttctgtcgcctctggatccctgggtttgcagaaatggcagcccccttgtaccctctcaccaaa
Pmv21         tcctagggacggcaggcttctgtcgcctctggatccctgggtttgcagaaatggcagcccccttgtaccctctcaccaaa
Pmv22         tcctagggacggcaggcttctgtcgcctctggatccctgggtttgcagaaatggcagcccccttgtaccctctcaccaaa
Pmv23         tcctagggacggcaggcttctgtcgcctctggatccctgggtttgcagaaatggcagcccccttgtaccctctcaccaaa
Pmv24         tcctagggacggcaggcttctgtcgcctctggatccctgggtttgcagaaatggcagcccccttgtaccctctcaccaaa
Pmv4          tcctagggacggcaggcttctgtcgcctctggatccctgggtttgcagaaatggcagcccccttgtaccctctcaccaaa
Pmv5          tcctagggacggcaggcttctgtcgcctctggatccctgggtttgcagaaatggcagcccccttgtaccctctcaccaaa
Pmv6          tcctagggacggcaggcttctgtcgcctctggatccctgggtttgcagaaatggcagcccccttgtaccctctcaccaaa
Pmv7          tcctagggacggcaggcttctgtcgcctctggatccctgggtttgcagaaatggcagcccccttgtaccctctcaccaaa
Pmv8          tcctagggacggcaggcttctgtcgcctctggatccctgggtttgcagaaatggcagcccccttgtaccctctcaccaaa
Pmv9          tcctagggacggcaggcttctgtcgcctctggatccctgggtttgcagaaatggcagcccccttgtaccctctcaccaaa
```

```
PMV_CONS      ACGGGGACTCTGTTTAATTGGGGCCCAGACCAGCAAAAGGCCTATCAAGAAATCAAACAGGCTCTTCTAACTGCCCCAGC
Pmv1          acggggactctgtttaattggggcccagaccagcaaaaggcctatcaagaaatcaaacaggctcttctaactgccccagc
Pmv10         acggggactctgtttaattggggcccagaccagcaaaaggcctatcaagaaatcaaacaggctcttctaactgccccagc
Pmv11         acggggactctgtttaattggggcccagaccagcaaaaggcctatcaagaaatcaaacaggctcttctaactgccccagc
Pmv12         acggggactctgtttaattggggcccagaccagcaaaaggcctatcaagaaatcaaacaggctcttctaactgccccagc
Pmv13         acggggactctgtttaattggggcccagaccagcaaaaggcctatcaagaaatcaaacaggctcttctaactgccccagc
Pmv14         acggggactctgtttaattggggcccagaccagcaaaaggcctatcaagaaatcaaacaggctcttctaactgccccagc
Pmv15         acggggactctgtttaattggggcccagaccagcaaaaggcctatcaagaaatcaaacaggctcttctaactgccccagc
Pmv16         acggggactctgtttaattggggcccagaccagcaaaaggcctatcaagaaatcaaacaggctcttctaactgccccagc
Pmv17         --------------------------------------------------------------------------------
Pmv18         acggggactctgtttaattggggcccagaccagcaaaaggcctatcaagaaatcaaacaggctcttctaactgccccagc
Pmv19         acggggactctgtttaattggggcccagaccagcaaaaggcctatcaagaaatcaaacaggctcttctaactgccccagc
Pmv2          acggggactctgtttaattggggcccagaccagcaaaaggcctatcaagaaatcaaacaggctcttctaactgccccagc
Pmv20         acggggactctgtttaattggggcccagaccagcaaaaggcctatcaagaaatTaaacaggctcttctaactgccccagc
Pmv21         acggggactctgtttaattggggcccagaccagcaaaaggcctatcaagaaatcaaacaggctcttctaactgccccagc
Pmv22         acggggactctgtttaattggggcccagaccagcaaaaggcctatcaagaaatcaaacaggctcttctaactgccccagc
Pmv23         acggggactctgtttaattggggcccagaccagcaaaaggcctatcaagaaatcaaacaggctcttctaactgccccagc
Pmv24         acggggactctgtttaattggggcccagaccagcaaaaggcctatcaagaaatTaaacaggctcttctaactgccccagc
Pmv4          acggggactctgtttaattggggcccagaccagcaaaaggcctatcaaAaaatcaaacaggctcttctaactgccccagc
Pmv5          acggggactctgtttaattggggcccagaccagcaaaaggcctatcaagaaatcaaacaggctcttctaactgccccagc
Pmv6          acggggactctgtttaattggggcccagaccagcaaaaggcctatcaagaaatcaaacaggctcttctaactgccccagc
Pmv7          acggggactctgtttaattggggcccagaccagcaaaaggcctatcaagaaatcaaacaggctcttctaactgccccagc
Pmv8          acggggactctgtttaattggggcccagaccagcaaaaggcctatcaagaaatcaaacaggctcttctaactgccccagc
Pmv9          acggggactctgtttaattggggcccagaccagcaaaaggcctatcaagaaatcaaacaggctcttctaactgccccagc

PMV_CONS      CCTGGGATTGCCAGATTTGACTAAGCCCTTTGAACTCTTTGTTGACGAGAAGCAGGGCTACGCCAAAGGCGTCCTAACGC
Pmv1          cctgggattgccagatttgactaagccctttgaactctttgttgacgagaagcagggctacgccaaaggcgtcctaacgc
Pmv10         cctgggattgccagatttgactaagccctttgaactctttgtCgacgagaagcagggctacgccaaaggcgtcctaacgc
Pmv11         cctgggattgccagatttgactaagccctttgaactctttgttgacgagaagcagggctacgccaaaggcgtcctaacgc
Pmv12         cctgggattgccagatttgactaagccctttgaactctttgttgacgagaagcagggctacgccaaaggcgtcctaacgc
Pmv13         cctgggattgccagatttgactaagccctttgaactctttgtCgacgagaagcagggctacgccaaaggcgtcctaacgc
Pmv14         cctgggattgccagatttgactaagccctttgaactctttgtCgacgagaagcagggctacgccaaaggcgtcctaacgc
Pmv15         cctgggattgccagatttgactaagccctttgaactctttgtCgacgagaagcagggctacgccaaaggcgtcctaacgc
Pmv16         cctgggattgccagatttgactaagccctttgaactctttgttgacgagaagcagggctacgccaaaggcgtcctaacgc
Pmv17         --------------------------------------------------------------------------------
Pmv18         cctgggattgccagatttgactaagccctttgaactctttgttgacgagaagcagggctacgccaaaggcgtcctaacgc
Pmv19         cctgggattgccagatttgactaagccctttgaactctttgttgacgagaagcagggctacgccaaaggcgtcctaacgc
Pmv2          cctgggattgccagatttgactaagccTtttgaactctttgttgacgagaagcagggctacgccaaaggcgtcctaacgc
Pmv20         cctgggattgccagatttgactaagccctttgaactctttgttgacgagaagcagggctacgccaaaggcgtcctaacgc
Pmv21         cctgggattgccagatttgactaagccctttgaactctttgttgacgagaagcagggctacgccaaaggcgtcctaacgc
Pmv22         cctgggattgccagatttgactaagccctttgaactctttgttgacgagaagcagggctacgccaaaggcgtcctaacgc
Pmv23         cctgAgattgccagatttgactaagccctttgaactctttgtCgacgagaagcagggctacgccaaaggcgtcctaacgc
Pmv24         cctgggattgccagatttgactaagccctttgaactctttgttgacgagaagcagggctacgccaaaggcgtcctaacgc
Pmv4          cctgAgattgccagatttgactaagccctttAaactctttgtCgacgagaagcagggctacgccaaaggcgtcctaacgc
Pmv5          cctgggattgccagatttgactaagccctttgaactctttgttgacgagaagcagggctacgccaaaggcgtcctaacgc
Pmv6          cctgggattgccagatttgactaagccctttgaactctttgttgacgagaagcagggctacgccaaaggcgtcctaacgc
Pmv7          cctgggattgccagatttgactaagccTtttgaactctttgttgacgagaagcagggctacgccaaaggcgtcctaacgc
Pmv8          cctgggattgccagatttgactaagccctttgaactctttgttgacgagaagcagggctacgccaaaggcgtcctaacgc
Pmv9          cctgggattgccagatttgactaagccctttgaactctttgtCgacgagaagcagggctacgccaaaggcgtcctaacgc
```

```
PMV_CONS      AAAAACTGGGACCTTGGCGTCGGCCGGTGGCCTACCTGTCCAAAAAGCTAGACCCAGTGGCAGCTGGGTGGCCCCCTTGC
Pmv1          aaaaactgggaccttggcgtcggccggtggcctacctgtccaaaaagctagacccagtggcagctgggtggccccccttgc
Pmv10         aaaaactgggaccttggcgtcggccggtggcctacctgtccaaaaagctagacccagtggcagctgggtggccccccttgc
Pmv11         aaaaactgggaccttggcgtcggccggtggcctacctgtccaaaaagctagacccagtggcagctgggtggccccccttgc
Pmv12         aaaaactgggaccttggcgtcggccggtggcctacctgtccaaaaagctagacccagtggcagctgggtggccccccttgc
Pmv13         aaaaactgggaccttggcgtcggccggtggcctacctgtccaaaaagctagacccagtggcagctgggtggccccccttgc
Pmv14         aaaaactgggaccttggcgtcggTcggtggcctacctgtccaaaaagctagacccagtggcagctgggtggccccccttgc
Pmv15         aaaaactgggaccttggcgtcggccggtggcctacctgtccaaaaagctagacccagtggcagctgggtggccccccttgc
Pmv16         aaaaactgggaccttggcgtcggccggtggcctacctgtccaaaaagctagacccagtggcagctgggtggccccccttgc
Pmv17         --------------------------------------------------------------------------------
Pmv18         aaaaactgggaccttggcgtcggccggtggcctacctgtccaaaaagctagacccagtggcagctgggtggccccccttgc
Pmv19         aaaaactgggaccttggcgtcggccggtggcctacctgtccaaaaagctagacccagtggcagctgggtggccccccttgc
Pmv2          aaaaactgggaccttggcgtcggccggtggcctacctgtccaaaaagctagacccagtggcagctgggtggccccccttgc
Pmv20         aaaaactgggaccttggcgtcggccggtggcctacctgtccaaaaagctagacccagtggcagctgggtggccccccttgc
Pmv21         aaaaactgggaccttggcgtcggccggtggcctacctgtccaaaaagctagacccagtggcagctgggtggccccccttgc
Pmv22         aaaaactgggaccttggcgtcggccggtggcctacctgtccaaaaagctagacccagtggcagctgggtggccccccttgc
Pmv23         aaaaactgggaccttggcgtcggccggtggTctacctgtccaaaaagctagacccagtggcagctgggtggccccccttgc
Pmv24         aaaaactgggaccttggcgtcggccggtggcctacctgtccaaaaagctagacccagtggcagctgggtggccccccttgc
Pmv4          aaaaactgggaccttggcgtcggccggtggcctacctgtccaaaaagctagacccagtggcagctgggtggccccccttgc
Pmv5          aaaaactgggaccttggcgtcggccggtggcctacctgtccaaaaagctagacccagtggcagctgggtggccccccttgc
Pmv6          aaaaactgggaccttggcgtcggccggtggcctacctgtccaaaaagctagacccagtggcagctgggtggccccccttgc
Pmv7          aaaaactgggaccttggcgtcggccggtggcctacctgtccaaaaagctagacccagtggcagctgggtggccccccttgc
Pmv8          aaaaactgggaccttggcgtcggccggtggcctacctgtccaaaaagctagacccagtggcagctgggtggccccccttgc
Pmv9          aaaaactgggaccttggcgtcggccggtggcctacctgtccaaaaagctagacccagtggcagctgggtggccccccttgc

PMV_CONS      CTACGGATGGTAGCAGCCATTGCCGTTCTGACAAAAGATGCAGGCAAGCTAACCATGGGACAGCCGCTAGTCATCCTGGC
Pmv1          ctacggatggtagcagccattgccgttctgacaaaagatgcaggcaagctaaccatgggacagccgctagtcatcctggc
Pmv10         ctacggatggtagcagccattgccgttctgacaaaagatgcaggcaagctaaccatgggacagccgctagtcatcctggc
Pmv11         ctacggatggtagcagccattgccgttctgacaaaagatgcaggcaagctaaccatgggacagccgctagtcatcctggc
Pmv12         ctacggatggtagcagccattgccgttctgacaaaagatgcaggcaagctaaccatgggacagccgctagtcatcctggc
Pmv13         ctacggatggtagcagccattgccgttctgacaaaagatgcaggcaagctaaccatgggacagccgctagtcatcctggc
Pmv14         ctacggatggtagcagccattgccgttctgacaaaagatgcaggcaagctaaccatgggacagccgctagtcatcctggc
Pmv15         ctacggatggtagcagccattgccgttctgacaaaagatgcaggcaagctaaccatgggacagccgctagtcatcctggc
Pmv16         ctacggatggtagcagccattgccgttctgacaaaagatgcaggcaagctaaccatgggacagccgctagtcatcctggc
Pmv17         --------------------------------------------------------------------------------
Pmv18         ctacggatggtagcagccattgccgttctgacaaaagatgcaggcaagctaaccatgggacagccgctagtcatcctggc
Pmv19         ctacggatggtagcagccattgccgttctgacaaaagatgcaggcaagctaaccatgggacagccgctagtcatcctggc
Pmv2          ctacggatggtagcagccattgccgttctgacaaaagatgcaggcaagctaaccatgggacagccgctagtcatcctggc
Pmv20         ctacggatggtagcagccattgccgttctgacaaaagatgcaggcaagctaaccatgggacagccgctagtcatcctggc
Pmv21         ctacggatggtagcagccattgccgttctgacaaaagatgcaggcaagctaaccatgggacagccgctagtcatcctggc
Pmv22         ctacggatggtagcagccattgccgttctgacaaaagatgcaggcaagctaaccatgggacagccgctagtcatcctggc
Pmv23         ctacggatggtagcagccattgccgttctgacaaaagatgcaggcaagctaaccatgggacagccgctagtcatcctggc
Pmv24         ctacggatggtagcagccattgccgttctgacaaaagatgcaggcaagctaaccatgggacagccgctagtcatcctggc
Pmv4          ctacggatggtagcagccattgccgttctgacaaaagatgcaggcaagctaaccatgggacagccgctagtcatcctggc
Pmv5          ctacggatggtagcagccattgccgttctgacaaaagatgcaggcaagctaaccatgggacagccgctagtcatcctggc
Pmv6          ctacggatggtagcagccattgccgttctgacaaaagatgcaggcaagctaaccatgggacagccgctagtcatcctggc
Pmv7          ctacggatggtagcagccattgccgttctgacaaaagatgcaggcaagctaaccatgggacagccgctagtcatcctggc
Pmv8          ctacggatggtagcagccattgccgttctgacaaaagatgcaggcaagctaaccatgggacagccgctagtcatcctggc
Pmv9          ctacggatggtagcagccattgccgttctgacaaaagatgcaggcaagctaaccatgggacagccgctagtcatcctggc
```

```
PMV_CONS    CCCCCATGCAGTAGAGGCACTAGTCAAGCAACCCCCTGACCGCTGGCTATCCAACGCCCGCATGACCCACTATCAGGCGA
Pmv1        cccccatgcagtagaggcactagtcaagcaaccccctgaccgctggctatccaacgcccgcatgacccactatcaggcga
Pmv10       cccccatgcagtagaggcactagtcaagcaaccccctgaccgctggctatccaacgcccgcatgacccactatcaggcga
Pmv11       cccccatgcagtagaggcactagtcaagcaaccccctgaccgctggctatccaacgcccgcatgacccactatcaggcga
Pmv12       cccccatgcagtagaggcactagtcaagcaaccccctgaccgctggctatccaacgcccgcatgacccactatcaggcga
Pmv13       cccccatgcagtagaggcactagtcaagcaaccccctgaccgctggctatccaacgcccgcatgacccactatcaggcga
Pmv14       cccccatgcagtagaggcactagtcaagcaaccccctgaccgctggctatccaacgcccgcatgacccactatcaggcga
Pmv15       cccccatgcagtagaggcactagtcaagcaaccccctgaccgctggctatccaacgcccgcatgacccactatcaggcga
Pmv16       cccccatgcagtagaggcactagtcaagcaaccccctgaccgctggctatccaacgcccgcatgacccactatcaggcga
Pmv17       --------------------------------------------------------------------------------
Pmv18       cccccatgcagtagaggcactagtcaagcaaccccctgaccgctggctatccaacgcccgcatgacccactatcaggcga
Pmv19       cccccatgcagtagaggcactagtcaagcaaccccctgaccgctggctatccaacgcccgcatgacccactatcaggcga
Pmv2        cccccatgcagtagaggcactagtcaagcaaccccctgaccgctggctatccaacgcccgcatgacccactatcaggcga
Pmv20       cccccatgcagtagaggcactagtcaagcaaccccctgaccgctggctatccaacgcccgcatgacccactatcaggcga
Pmv21       cccccatgcagtagaggcactagtcaagcaaccccctgaccgctggctatccaacgcccgcatgacccactatcaggcga
Pmv22       cccccatgcagtagaggcactagtcaagcaaccccctgaccgctggctatccaacgcccgcatgacccactatcaggcga
Pmv23       cccccatgcagtagaggcactagtcaagcaaccccctgaccgctggctatccaacgcccgcatgacccactatcaggcga
Pmv24       cccccatgcagtagaggcactagtcaagcaaccccctgaccgctggctatccaacgcccgcatgacccactatcaggcga
Pmv4        cccccatgcagtagaggcactagtcaagcaacccccGgaTcgctggctatccaacgcccgcatgacccactatcaggcga
Pmv5        cccccatgcagtagaggcactagtcaagcaaccccctgaccgctggctatccaacgcccgcatgacccactatcaggcga
Pmv6        cccccatgcagtagaggcactagtcaagcaaccccctgaccgctggctatccaacgcccgcatgacccactatcaggcga
Pmv7        cccccatgcagtagaggcactagtcaagcaaccccctgaccgctggctatccaacgcccgcatgacccactatcaggcga
Pmv8        cccccatgcagtagaggcactagtcaagcaaccccctgaccgctggctatccaacgcccgcatgacccactatcaggcga
Pmv9        cccccatgcagtagaggcactagtcaagcaacccccGgaTcgctggctatccaacgcccgcatgacccactatcaggcga

PMV_CONS    TGCTCCTAGATACGGACCGGGTCCAGTTCGGACCGGTGGTAGCCCTAAACCCGGCTACGTTGCTCCCCCTACCGGGGAAA
Pmv1        tgctcctagatacggaccgggtccagttcggaccggtggtagccctaaacccggctacgttgctccccctaccggggaaa
Pmv10       tgctcctagatacggaccgggtccagttcggaccggtggtagccctaaacccggctacgttgctccccctaccggggaaa
Pmv11       tgctcctagatacggaccgggtccagttcggaccggtggtagccctaaacccggctacgttgctccccctaccggggaaa
Pmv12       tgctcctagatacggaccgggtccagttcggaccggtggtagccctaaacccggctacgttgctccccctaccggggaaa
Pmv13       tgctcctagatacggaccgggtccagttcggaccggtggtagccctaaacccggctacgttgctccccctaccggggaaa
Pmv14       tgctcctagatacggaccgggtccagttcggaccggtggtagccctaaacccggctacgttgctccccctaccggggaaa
Pmv15       tgctcctagatacggaccgggtccagttcggaccggtggtagccctaaacccggctacgttgctccccctaccggggaaa
Pmv16       tgctcctagatacggaccgggtccagttcggaccggtggtagccctaaacccggctacgttgctccccctaccggggaaa
Pmv17       --------------------------------------------------------------------------------
Pmv18       tgctcctagatacggaccgggtccagttcggaccggtggtagccctaaacccggctacgttgctccccctaccggggaaa
Pmv19       tgctcctagatacggaccgggtccagttcggaccggtggtagccctaaacccggctacgttgctccccctaccggggaaa
Pmv2        tgctcctagatacggaccgggtccagttcggaccggtggtagccctaaacccggctacgttgctccccctaccggggaaa
Pmv20       tgctcctagatacggaccgggtccagttcggaccggtggtagccctaaacccggctacgttgctccccctaccggggaaa
Pmv21       tgctcctagatacggaccgggtccagttcggaccggtggtagccctaaacccggctacgttgctccccctaccggggaaa
Pmv22       tgctcctagatacggaccgggtccagttcggaccggtggtagccctaaacccggctacgttgctccccctaccggggaaa
Pmv23       tgctcctagatacggaccgggtccagttcggaccggtggtagccctaaacccggctacgttgctccccctaccggggaaa
Pmv24       tgctcctagatacggaccgggtccagttcggaccggtggtagccctaaacccggctacgttgctccccctaccggggaaa
Pmv4        tgctcctagatacggaccgggtccagttcggaccggtggtagccctaaacccggctacgttgctccccctaccgggAaaa
Pmv5        tgctcctagatacggaccgggtccagttcggaccggtggtagccctaaacccggctacgttgctccccctaccggggaaa
Pmv6        tgctcctagatacggaccgggtccagttcggaccggtggtagccctaaacccggctacgttgctccccctaccggggaaa
Pmv7        tgctcctagatacggaccgggtccagttcggaccggtggtagccctaaacccggctacgttgctccccctaccggggaaa
Pmv8        tgctcctagatacggaccgggtccagttcggaccggtggtagccctaaacccggctacgttgctccccctaccggggaaa
Pmv9        tgctcctagatacggaccgggtccagttcggaccggtggtagccctaaacccggctacgttgctccccctaccggggaaa
```

```
PMV_CONS      GAGCCTCACCATGACTGCCTCGAGATCTTGGCCGAGACACACGGAACCAGACCAGACCTCACGGACCAGCCCCTCCCAGA
Pmv1          gagcctcaccatgactgcctcgagatcttggccgagacacacggaaccagaccagacctcacggaccagcccctcccaga
Pmv10         gagcctcaccatgactgcctcgagatcttggccgagacacacggaaccagaccagacctcacggaccagcccctcccaga
Pmv11         gagcctcaccatgactgcctcgagatcttggccgagacacacggaaccagaccagacctcacggaccagcccctcccaga
Pmv12         gagcctcaccatgactgcctcgagatcttggccgagacacacggaaccagaccagacctcacggaccagcccctcccaga
Pmv13         gagcctcaccatgactgcctcgagatcttggccgagacacacggaaccagaccagacctcacggaccagcccctcccaga
Pmv14         gagcctcaccatgactgcctcgagatcttggccgagacacacggaaccagaccagacctcacggaccagcccctcccaga
Pmv15         gagcctcaccatgactgcctcgagatcttggccgagacacacggaaccagaccagacctcacggaccagcccctcccaga
Pmv16         gagcctcaccatgactgcctcgagatcttggccgagacacacggaaccagaccagacctcacggaccagcccctcccaga
Pmv17         --------------------------------------------------------------------------------
Pmv18         gagcctcaccatgactgcctcgagatcttggccgagacacacggaaccagaccagacctcacggaccagcccctcccaga
Pmv19         gagcctcaccatgactgcctcgagatcttggccgagacacacggaaccagaccagacctcacggaccagcccctcccaga
Pmv2          gagcctcaTcatgactgcctcgagatcttggccgagacacacggaaccagaccagacctcacggaccagcccctcccaga
Pmv20         gagcctcaccatgactgcctcgagatcttggccgagacacacggaaccagaccagacctcacggaccagcccctcccaga
Pmv21         gagcctcaccatgactgcctcgagatcttggccgagacacacggaaccagaccagacctcacggaccagcccctcccaga
Pmv22         gagcctcaccatgactgcctcgagatcttggccgagacacacggaaccagaccagacctcacggaccagcccctcccaga
Pmv23         gagcctcaccatgactgcctcgagatcttggccgagacacacggaaccagaccagacctcacggaccagcccctcccaga
Pmv24         gagcctcaccatgactgcctcgagatcttggccgagacacacggaaccagaccagacctcacggaccagcccctcccaga
Pmv4          gagcctcaccatgactgcctcgagatcttggccgagacacacggaaccagaccagacctcacggaccagcccctcccaga
Pmv5          gagcctcaccatgactgcctcgagatcttggccgagacacacggaaccagaccagacctcacggaccagcccctcccaga
Pmv6          gagcctcaccatgactgcctcgagatcttggccgagacacacggaaccagaccagacctcacggaccagcccctcccaga
Pmv7          gagcctcaccatgactgcctcgagatcttggccgagacacacggaaccagaccagacctcacggaccagcccctcccaga
Pmv8          gagcctcaccatgactgcctcgagatcttggccgagacacacggaaccagaccagacctcacggaccagcccctcccaga
Pmv9          gagcctcaccatgactgcctcgagatcttggccgagacacacggaaccagaccagacctcacggaccagcccctcccaga

PMV_CONS      CGCCGACCACACCTGGTATACAGATGGAAGCAGCTTCCTGCAAGAGGGACAACGTAGGGCTGGAGCAGCGGTGACCACCG
Pmv1          cgccgaccacacctggtatacagatggaagcagcttcctgcaagagggacaacgtagggctggagcagcggtgaccaccg
Pmv10         cgccgaccacacctggtatacagatggaagcagcttcctgcaagagggacaacgtagggctggagcagcggtgaccaccg
Pmv11         cgccgaccacacctggtatacagatggaagcagcttcctgcaagagggacaacgtagggctggagcagcggtgaccaccg
Pmv12         cgccgaccaTacctggtatacagatggaagcagcttcctgcaagagggacaacgtagggctggagcagcggtgaccaccg
Pmv13         cgccgaccacacctggtatacagatggaagcagcttcctgcaagagggacaacgtagggctggagcagcggtgaccaccg
Pmv14         cgccgaccacacctggtatacagatggaagcagcttcctgcaagagggacaacgtagggctggagcagcggtgaccaccg
Pmv15         cgccgaccacacctggtatacagatgAaagcagcttcctgcaagagggacaacgtagggctggagcagcggtgaccaccg
Pmv16         cgccgaccacacctggtatacagatggaagcagcttcctgcaagagggacaacgtagggctggagcagcggtgaccaccg
Pmv17         --------------------------------------------------------------------------------
Pmv18         cgccgaccacacctggtatacagatggaagcagcttcctgcaagagggacaacgtagggctggagcagcggtgaccaccg
Pmv19         cgccgaccacacctggtatacagatggaagcagcttcctgcaagagggacaacgtagggctggagcagcggtgaccaccg
Pmv2          cgccgaccacacctggtatacagatggaagcagcttcctgcaagagggacaacgtagggctggagcagcggtgaccaccg
Pmv20         cgccgaccacacctggtatacagatggaagcagcttcctgcaagagggacaacgtagggctggagcagcggtgaccaccg
Pmv21         cgccgaccacacctggtatacagatggaagcagcttcctgcaagagggacaacgtagggctggagcagcggtgaccaccg
Pmv22         cgccgaccacacctggtatacagatggaagcagcttcctgcaagagggacaacgtagggctggagcagcggtgaccaccg
Pmv23         cgccgaccacacctggtatacagatggaagcagcttcctgcaagagggacaacgtagggctggagcagcggtgaccaccg
Pmv24         cgccgaccacacctggtatacagatggaagcagcttcctgcaagagggacaacgtagggctggagcagcggtgaccaccg
Pmv4          cgccgaccacacctggtatacagatAAaagcagcttcctgcaagagggacaacgtagggctAgagcagcggtgaccaccg
Pmv5          cgccgaccacacctggtatacagatggaagcagcttcctgcaagagggacaacgtagggctggagcagcggtgaccaccg
Pmv6          cgccgaccacacctggtatacagatggaagcagcttcctgcaagagggacaacgtagggctggagcagcggtgaccaccg
Pmv7          cgccgaccacacctggtatacagatggaagcagcttcctgcaagagggacaacgtagggctggagcagcggtgaccaccg
Pmv8          cgccgaccacacctggtatacagatggaagcagcttcctgcaagagggacaacgtagggctggagcagcggtgaccaccg
Pmv9          cgccgaccacacctggtatacagatggaagcagcttcctgcaagagggacaacgtagggctggagcagcggtgaccaccg
```

```
PMV_CONS      AGACCGAGGTAATCTGGGCCAAGGCGTTGCCAGCCGGGACATCCGCCCAGCGAGCTGAACTAATAGCACTCACCCAGGCC
Pmv1          agaccgaggtaatctgggccaaggcgttgccagccgggacatccgcccagcgagctgaactaatagcactcacccaggcc
Pmv10         agaccgaggtaatctgggccaaggcgttgccagccgggacatccgcccagcgagctgaactaatagcactcacccaggcc
Pmv11         agaccgaggtaatctgggccaaggcgttgccagccgggacatccgcccagcgagctgaactaatagcactcacccaggcc
Pmv12         agaccgaggtaatctgggccaaggcgttgccagccgggacatccgcccagcgagctgaactaatagcactcacccaggcc
Pmv13         agaccgaggtaatctgggccaaggcgttgccagccgggacatccgcccagcgagctgaactaatagcactcacccaggcc
Pmv14         agaccgaggtaatctgggccaaggcgttgccagccgggacatccgcccagcgagctgaactaatagcactcacccaggcc
Pmv15         agaccgaggtaatctgggccaaggcgttgccagccgggacatccgcccagcgagctgaactaatagcactcacccaggcc
Pmv16         agaccgaggtaatctgggccaaggcgttgccagccgggacatccgcccagcgagctgaactaatagcactcacccaggcc
Pmv17         -----------atctgggccaaggcgttgccagccgggacatccgcccagcgagctgaactaatagcactcacccaggcc
Pmv18         agaccgaggtaatctgggccaaggcgttgccagccgggacatccgcccagcgagctgaactaatagcactcacccaggcc
Pmv19         agaccgaggtaatctgggccaaggcgttgccagccgggacatccgcccagcgagctgaactaatagcactcacccaggcc
Pmv2          agaccgaggtaatctgggccaaggcgttgccagccgggacatccgcccagcgagctgaactaatagcactcacccaggcc
Pmv20         agaccgaggtaatctgggccaaggcgttgccagccgggacatccgcccagcgagctgaactaatagcactcacccaggcc
Pmv21         agaccgaggtaatctgggccaaggcgttgccagccgggacatccgcccagcgagctgaactaatagcactcacccaggcc
Pmv22         agaccgaggtaatctgggccaaggcgttgccagccgggacatccgcccagcgagctgaactaatagcactcacccaggcc
Pmv23         agaccgaggtaatctgggccaaggcgttgccagccgggacatccgcccagcgagctgaactaatagcactcacccaggcc
Pmv24         agaccgaggtaatctgggccaaggcgttgccagccgggacatccgcccagcgagctgaactaatagcactcacccaggcc
Pmv4          agaccgaggtaatctgggccaaggcAttgccagccgggacatccgcccagcgagctgaactaatagcactcacccaggcc
Pmv5          agaccgaggtaatctgggccaaggcgttgccagccgggacatccgcccagcgagctgaactaatagcactcacccaggcc
Pmv6          agaccgaggtaatctgggccaaggcgttgccagccgggacatccgcccagcgagctgaactaatagcactcacccaggcc
Pmv7          agaccgaggtaatctgggccaaggcgttgccagccgggacatccgcccagcgagctgaactaatagcactcacccaggcc
Pmv8          agaccgaggtaatctgggcc------------------------------------------------------------
Pmv9          agaccgaggtaatctgggccaaggcAttgccagccgggacatccgcccagcgagctgaactaatagcactcacccaggcc

PMV_CONS      CTAAAGATGGCAGAAGGTAAGAAGCTAAATGTTTATACTGATAGCCGCTATGCCTTTGCTACCGCCCATGTCCATGGAGA
Pmv1          ctaaagatggcagaaggtaagaagctaaatgtttatactgatagccgctatgcctttgctaccgcccatAtccatggaga
Pmv10         ctaaagatggcagaaggtaagaagctaaatgtttatactgatagccgctatgcctttgctaccgcccatgtccatggaga
Pmv11         ctaaagatggcagaaggtaagaagctaaatgtttatactgatagccgctatgcctttgctaccgcccatAtccatggaga
Pmv12         ctaaagatggcagaaggtaagaagctaaatgtttatactgatagccgctatgcctttgctaccgcccatgtccatggaga
Pmv13         ctaaagatggcagaaggtaagaagctaaatgtttatactgatagccgctatgcctttgctaccgcccatgtccatggaga
Pmv14         ctaaagatggcagaaggtaagaagctaaatgtttatactgatagccgctatgcctttgctaccgcccatgtccatAAaAa
Pmv15         ctaaagatggcagaaggtaagaagctaaatgtttatactgatagccgctatgcctttgctaccgcccatgtccatggaga
Pmv16         ctaaagatggcagaaggtaagaagctaaatgtttatactgatagccgctatgcctttgctaccgcccatAtccatggaga
Pmv17         ctaaagatggcagaaggtaagaagctaaaCgtttatactgatagccgctatgcctttgctaccgcccatgtccatggaga
Pmv18         ctaaagatggcagaaggtaagaagctaaatgtttatactgatagccgctatgcctttgctaccgcccatgtccatggaga
Pmv19         ctaaagatggcagaaggtaagaagctaaatgtttatactgatagccgctatgcctttgctaccgcccatgtccatggaga
Pmv2          ctaaagatggcagaaggtaagaagctaaatgtttatactgatagccgctatgcctttgctaccgcccatgtccatggaga
Pmv20         ctaaagatggcagaaggtaagaagctaaatgtttatactgatagccgctatgcctttgctaccgcccatgtccatggaga
Pmv21         ctaaagatggcagaaggtaagaagctaaatgtttatactgatagccgctatgcctttgctaccgcccatgtccatgAaAa
Pmv22         ctaaagatggcagaaggtaagaagctaaatgtttatactgatagccgctatgcctttgctaccgcccatgtccatggaga
Pmv23         ctaaagatggcagaaggtaagaagctaaatgtttatactgatagccgctatgcctttgctaccgcccatgtccatggaAa
Pmv24         ctaaagatggcagaaggtaagaagctaaatgtttatactgatagccgctatgcctttgctaccgcccatgtccatggaga
Pmv4          ctaaagatggcagaaggtaaAaagctaaatgtttatactgatagccgctatgcctttgctaccgcccatgtccatggaga
Pmv5          ctaaagatggcagaaggtaagaagctaaatgtttatactgatagccgctatgcctttgctaccgcccatAtccatggaga
Pmv6          ctaaagatggcagaaggtaagaagctaaatgtttatactgatagccgctatgcctttgctaccgcccatgtccatggaga
Pmv7          ctaaagatggcagaaggtaagaagctaaatgtttatactgatagccgctatgcctttgctaccgcccatgtccatggaAa
Pmv8          --------------------------------------------------------------------------------
Pmv9          ctaaagatggcagaaggtaagaagctaaatgtttatactgatagccgctatgcctttgctaccgcccatgtccatgAaAa
```

```
PMV_CONS      AATATATAGGAGACGTGGGTTGCTCACCTCAGAAGGCAAGGAGATCAAGAACAAGGGCGAAATCTTGGCCTTACTGAAAG
Pmv1          aatatataggagacgtgggttgctcacctcagaaggcaaggagatcaagaacaagggcgaaatcttggccttactgaaag
Pmv10         aatatataggagacgtgggttgctcacctcagaaggcaaggagatcaagaacaagggcgaaatcttggccttactgaaag
Pmv11         aatatataggagacgtgggttgctcacctcagaaggcaaggagatcaagaacaagggcgaaatcttggccttactgaaag
Pmv12         aatatataggagacgtgggttgctcacctcagaaggcaaggagatcaagaacaagggcgaaatcttggccttactgaaag
Pmv13         aatatataggagacgtgggttgctcacctcagaaggcaaggagatcaagaacaagggcgaaatcttggccttactgaaag
Pmv14         aatatataggagacgtgggttActcacctcagaaggcaaAgagatcaagaacaagggcgaaatcttggccttactgaaag
Pmv15         aatatataggagacgtgggttgctcacctcagaaggcaaggagatcaagaacaagggcgaaatcttAgccttactAaaag
Pmv16         aatatataggagacgtgggttgctcacctcagaaggcaaggagatcaagaacaagggcgaaatcttggccttactgaaag
Pmv17         aatatataggagacgtgggttgctcacctcagaaggcaaggagatcaaAaacaagggcgaaatcttggccttactgaaag
Pmv18         aatatataggagacgtgggttgctcacctcagaaggcaaggagatcaagaacaagggcgaaatcttggccttactgaaag
Pmv19         aatatataggagacgtgggttgctcacctcagaaggcaaggagatcaagaacaagggcgaaatcttggccttactgaaag
Pmv2          aatatataggagacgtgggttgctcacctcagaaggcaaggagatcaagaacaagggcgaaatcttggccttactgaaag
Pmv20         aatatataggagacgtgggttgctcacctcagaaggcaaAgagatcaagaacaagggcgaaatcttggccttactgaaag
Pmv21         aatatataggagacgtgggttgctcacctcagaaggcaaggagatcaagaacaagggcgaaatcttggccttactgaaag
Pmv22         aatatataggagacgtgggttgctcacctcagaaggcaaggagatcaagaacaagggcgaaatcttggccttactgaaag
Pmv23         aatatataggagacgtgggttgctcacctcagaaggcaaggagatcaagaacaagggcgaaatcttggccttactgaaag
Pmv24         aatatataggagacgtgggttgctcacctcagaaggcaaAgagatcaagaacaagggcgaaatcttggccttactgaaag
Pmv4          aatatataAgagacgtgggttgctcacctcagaaggcaaggagatcaagaacaagggcgaaatcttggccttactgaaag
Pmv5          aatatataggagacgtgggttgctcacctcagaaggcaaggagatcaagaacaagggcgaaatcttggccttactgaaag
Pmv6          aatatataggagacgtgggttgctcacctcagaaggcaaggagatcaagaacaagggcgaaatcttggccttactgaaag
Pmv7          aatatataggagacgtgggttgctcacctcagaaggcaaggagatcaagaacaagggcgaaatcttggccttactgaaag
Pmv8          --------------------------------------------------------------------------------
Pmv9          aatatataggagacgtgggttgctcacctcagaaggcaaggagatcaagaacaagggcgaaatcttggccttactgaaag

PMV_CONS      CTCTCTTTCTGCCCAAAAGACTCAGTATAATTCACTGCCCAGGACATCAGAAAGGCAATAGTGCTGAAGCTAAAGGCAAC
Pmv1          ctctctttctgcccaaaagactcagtataattcactgcccaggacatcagaaaggcaatagtgctgaagctaaaggcaac
Pmv10         ctctctttctgcccaaaagactcagtataattcactgcccaggacatcagaaaggcaatagtgctgaagctaaaggcaac
Pmv11         ctctctttctgcccaaaagactcagtataattcactgcccaggacatcagaaaggcaatagtgctgaagctaaaggcaac
Pmv12         ctctctttctgcccaaaagactcagtataattcactgcccaggacatcagaaaggcaatagtgctgaagctaaaggcaac
Pmv13         ctctctttctgcccaaaagactcagtataattcactgcccaggacatcagaaaggcaatagtgctgaagctaaaggcaac
Pmv14         ctctctttctgcccaaaagactcagtataattcactgcccaggacatcagaaaggcaatagtgctgaagctaaaggcaac
Pmv15         ctctctttctgcccaaaagactcagtataattcactgcccaggacatcagaaaggcaatagtgctgaagctaaaggcaac
Pmv16         ctctctttctgcccaaaagactcagtataattcactgcccaggacatcagaaaggcaatagtgctgaagctaaaggcaac
Pmv17         ctctctttTtgcccaaaagactcagtataattcactgcccaggacatcagaaaggcaatagtgctgaagctaaaggcaac
Pmv18         ctctctttctgcccaaaagactcagtataattcactgcccaggacatcagaaaggcaatagtgctgaagctaaaggcaac
Pmv19         ctctctttctgcccaaaagactcagtataattcactgcccaggacatcagaaaggcaatagtgctgaagctaaaggcaac
Pmv2          ctctctttctgcccaaaagactcagtataattcactgcccaggacatcagaaaggcaatagtgctgaagctaaaggcaac
Pmv20         ctctctttctgcccaaaagactcagtataattcactgcccaggacatcagaaaggcaatagtgctgaagctaaaggcaac
Pmv21         ctctctttctgcccaaaagactcagtataattcactgcccaggacatcagaaaggcaatagtgctgaagctaaaggcaac
Pmv22         ctctctttctgcccaaaagactcagtataattcactgcccaggacatcagaaaggcaatagtgctgaagctaaaggcaac
Pmv23         ctctctttctgcccaaaagactcagtataattcactgcccaggacatcagaaaggcaatagtgctgaagctaaaggcaac
Pmv24         ctctctttctgcccaaaagactcagtataattcactgcccaggacatcagaaaggcaatagtgctgaagctaaaggcaac
Pmv4          ctctctttctgcccaaaagactcagtataattcactgcccaggacatcagaaaggcaatagtgctgaagctaaaggcaac
Pmv5          ctctctttctgcccaaaagactcagtataattcactgcccaggacatcagaaaggcaatagtgctgaagctaaaggcaac
Pmv6          ctctctttctgcccaaaagactcagtataattcactgcccaggacatcagaaaggcaatagtgctgaagctaaaggcaac
Pmv7          ctctctttctgcccaaaagactcagtataattcactgcccaggacatcagaaaggcaatagtgctgaagctaaaggcaac
Pmv8          --------------------------------------------------------------------------------
Pmv9          ctctctttctgcccaaaagactcagtataattcactgcccaggacatcagaaaggcaatagtgctgaagctaaaggcaac
```

```
PMV_CONS      CGAATGGCGGACCAGGCAGCCCGGGAAGCAGCCATGGGGACTGACACAAAGGCCTCCTCACTTCTCATAGAGACCTCAAC
Pmv1          cgaatggcggaccaggcagcccgggaagcGgccatggggactgacacaaaggcctcctcacttctcatagagacctcaac
Pmv10         cgaatggcggaccaggcagcccgggaagcagccatggggactgacacaaaggcctcctcacttctcatagagacctcaac
Pmv11         cgaatggcggaccaggcagcccgggaagcGgccatggggactgacacaaaggcctcctcacttctcatagagacctcaac
Pmv12         cgaatggcggaccaggcagcccgggaagcagccatggggactgacacaaaggcctcctcacttctcatagagacctcaac
Pmv13         cgaatggcggaccaggcagcccgggaagcagccatggggactgacacaaaggcctcctcacttctcatagagacctcaac
Pmv14         cgaatggcggaccaggcagcccgggaagcagccatggggactgacacaaagAcctcctcacttctcatagagacctcaac
Pmv15         cgaatggcggaccaggcagcccgggaagcagccatggggactgacacaaagAcctcctcacttctcatagagacctcaac
Pmv16         cgaatggcggaccaggcagcccgggaagcGgccatggggactgacacaaaggcctcctcacttctcatagagacctcaac
Pmv17         cAaatggcggaccaggcagcccgggaagcagccatggggactgacacaaaggcctcctcacttctcatagagacctcaac
Pmv18         cgaatggcggaccaggcagcccgggaagcagccatggggactgacacaaaggcctcctcacttctcatagagacctcaac
Pmv19         cgaatggcggaccaggcagcccgggaagcagccatggggactgacacaaaggcctcctcacttctcatagagacctcaac
Pmv2          cgaatggcggaccaggcagcccgggaagcagccatggggactgacacaaaggcctcctcacttctcatagagacctcaac
Pmv20         cgaatggcggaccaggcagcccgAgaagcagccatggggactgacacaaaggcctcctcacttctcatagagacctcaac
Pmv21         cAaatggcggaccaggcagcccgggaagcagccatggggactgacacaaaggcctcctcacttctcatagagacctcaac
Pmv22         cgaatggcggaccaggcagcccgggaagcagccatggggactgacacaaaggcctcctcacttctcatagagacctcaac
Pmv23         cgaatggcggaccaggcagcccgggaagcagccatggggactgacacaaaggcctcctcacttctcatagagacctcaac
Pmv24         cgaatggcggaccaggcagcccgggaagcagccatggggactgacacaaaggcctcctcacttctcatagagacctcaac
Pmv4          cgaatggcggaccaggcagcccgggaagcagccatggggactAacacaaaggcctcctcacttctcatagagacctcaac
Pmv5          cgaatggcggaccaggcagcccgggaagcGgccatggggactgacacaaaggcctcctcacttctcatagagacctcaac
Pmv6          cgaatggcggaccaggcagcccgggaagcagccatggggactgacacaaaggcctcctcacttctcatagagacctcaac
Pmv7          cgaatggcggaccaggcagcccgggaagcagccatggggactgacacaaaggcctcctcacttctcatagagacctcaac
Pmv8          --------------------------------------------------------------------------------
Pmv9          cgaatggcggaccaggcagcccgggaagcagccatggggactgacacaaaggcctcctcacttctcatagagacctcaac

PMV_CONS      CCCGTACACTCCAGACTTCTTCCATTATACTGAGACAGATATAAAGAACCTACAAGAGTTGGGAGCCACATATGATAGAG
Pmv1          cccgtacactccagacttcttccattatactgagacagatataaagaacctacaagagttgggagccacatatgatagag
Pmv10         cccgtacactccagacttcttccattatactgagacagatataaagaacctacaagagttgggagccacatatgatagag
Pmv11         cccgtacactccagacttcttccattatactgagacagatataaagaacctacaagagttgggagccacatatgatagag
Pmv12         cccgtacactccagacttcttccattatactgagacagatataaagaacctacaagagttgggagccacatatgatagag
Pmv13         cccgtacactccagacttcttccattatactgagacagatataaagaacctacaagagttgggagccacatatgatagag
Pmv14         cccgtacactccagacttcttccattatactgagacagatataaagaacctacaagagttgggagccacatatgatagag
Pmv15         cccgtacactccagacttcttccattatactgagacagatataaagaacctacaagagttgggagccacatatgatagag
Pmv16         cccgtacactccagacttcttccattatactgagacagatataaagaacctacaagagttgggagccacatatgatagag
Pmv17         cccgtacactccagacttcttccattatactgagacagatataaagaacctacaagagttgggagccacatatgatagag
Pmv18         cccgtacactccagacttcttccattatactgagacagatataaagaacctacaagagttgggagccacatatgatagag
Pmv19         cccgtacactccagacttcttccattatactgagacagatataaagaacctacaagagttgggagccacatatgatagag
Pmv2          cccgtacactccagacttcttccattatactgagacagatataaagaacctacaagagttgggagccacatatgatagag
Pmv20         cccgtacactccagacttcttccattaCactgagacagatataaagaacctacaagagttgggagccacatatgatagag
Pmv21         cccgtacactccagacttcttccattatactgagacagatataaagaacctacaagagttgggagccacatatgatagag
Pmv22         cccgtacactccagacttcttccattatactgagacagatataaagaacctacaagagttgggagccacatatgatagag
Pmv23         cccgtacactccagacttcttccattatactgagacagatataaagaacctacaagagttgggagccacatatgatagag
Pmv24         cccgtacactccagacttcttccattaCactgagacagatataaagaacctacaagagttgggagccacatatgatagag
Pmv4          cccgtacactccagacttcttccattatactgagacagatataaagaacctacaGgagttgggagccacatatgatagag
Pmv5          cccgtacactccagacttcttccattatactgagacagatataaagaacctacaagagttgggagccacatatgatagag
Pmv6          cccgtacactccagacttcttccattatactgagacagatataaagaacctacaagagttgggagccacatatgatagag
Pmv7          cccgtacactccagacttcttccattatactgagacagatataaagaacctacaagagttgggagccacatatgatagag
Pmv8          --------------------------------------------------------------------------------
Pmv9          cccgtacactccagacttcttccattatactgagacagatataaagaacctacaagagttgggagccacatatgatagag
```

```
PMV_CONS    AGAAAAAATATTGGGTCCTGCAAGGTAAACCTGTGATGCCTGACCAGTTCACCTTTGAATTATTAGACTTCCTTCACCAG
Pmv1        agaaaaaatattgggtcctgcaaggtaaacctgtgatgcctgaccagttcacctttgaattattagacttccttcaccag
Pmv10       agaaaaaatattgggtcctgcaaggtaaacctgtgatgcctgaccagttcacctttgaattattagacttccttcaccag
Pmv11       agaaaaaatattgggtcctgcaaggtaaacctgtgatgcctgaccagttcacctttgaattattagacttccttcaccag
Pmv12       aAaaaaaatattgggtcctgcaaggtaaacctgtgatgcctgaccagttcacctttgaattattagacttccttcaccag
Pmv13       agaaaaaatattgggtcctgcaaggtaaacctgtgatgcctgaccagttcacctttgaattattagacttccttcaccag
Pmv14       agaaaaaatattgggtcctgcaaggtaaacctgtgatgcctgaccagttcacctttAaattattagacttccttcaccag
Pmv15       aAaaaaaatattgggtcctgcaaggtaaacctgtgatgcctgaccagttcacctttAaattattagacttccttcaccag
Pmv16       agaaaaaatattgggtcctgcaaggtaaacctgtgatgcctgaccagttcacctttAaattattagacttccttcaccag
Pmv17       agaaaaaatattgggtcctgcaaggtaaacctgtgatgcctgaccagttcacctttgaattattagacttccttcaccag
Pmv18       agaaaaaatattgggtcctgcaaggtaaacctgtgatgcctgaccagttcacctttgaattattagacttccttcaccag
Pmv19       agaaaaaatattgggtcctgcaaggtaaacctgtgatgcctgaccagttcacctttgaattattagacttccttcaccag
Pmv2        agaaaaaatattgggtcctgcaaggtaaacctgtgatgcctgaccagttcacctttAaattattagacttccttcaccag
Pmv20       agaaaaaatattgggtcctgcaaggtaaacctgtgatgcctgaccagttcacctttgaattattagacttccttcaccag
Pmv21       agaaaaaatattgggtcctgcaaggtaaacctgtgatgcctgaccagttcacctttAaattattagacttccttcaccag
Pmv22       agaaaaaatattgggtcctgcaaggtaaacctgtgatgcctgaccagttcacctttgaattattagacttccttcaccag
Pmv23       agaaaaaatattgggtcctgcaaggtaaacctgtgatgcctgaccagttcacctttgaattattagacttccttcaccag
Pmv24       agaaaaaatattgggtcctgcaaggtaaacctgtgatgcctgaccagttcacctttgaattattagacttccttcaccag
Pmv4        aAaaaaaatattgggtcctgcaaAgtaaacctgtgatgcctgaccagttcacctttAaattattagacttccttcaccag
Pmv5        agaaaaaatattgggtcctgcaaggtaaacctgtgatgcctgaccagttcacctttgaattattagacttccttcaccag
Pmv6        agaaaaaatattgggtcctgcaaggtaaacctgtgatgcctgaccagttcacctttgaattattagacttccttcaccag
Pmv7        agaaaaaatattgggtcctgcaaggtaaacctgtgatgcctgaccagttcacctttgaattattagacttccttcaccag
Pmv8        --------------------------------------------------------------------------------
Pmv9        agaaaaaatattgggtcctgcaaggtaaacctgtgatgcctgaccagttcacctttgaattattagacttccttcaccag

PMV_CONS    CTCACCCACCTTAGCTATCAGAAGATGAGGGCACTTCTAGACAGGAAAGAAAGCCCCTATTACATGCTAAATAAAGATAA
Pmv1        ctcacccaccttagctatcagaagatgagggcacttctagacaggaaagaaagcccctattacatgctaaataaagataa
Pmv10       ctcacccaccttagctatcagaagatgagggcacttctagacaggaaagaaagcccctattacatgctaaataaagataa
Pmv11       ctcacccaccttagctatcagaagatgagggcacttctagacaggaaagaaagcccctattacatgctaaataaagataa
Pmv12       ctcacccaccttagctatcagaagatgagggcacttctagacaggaaagaaagcccctattacatgctaaataaagataa
Pmv13       ctcacccaccttagctatcagaagatgagggcacttctagacaggaaagaaagcccctattacatgctaaataaagataa
Pmv14       ctcacccaccttagctatcagaagatgagggcacttctagacagAaaagaaagcccctattacatgctaaataaagataa
Pmv15       ctcacccaccttagctatcagaagatgagggcacttctagacaggaaagaaagcccctattacatgctaaataaagataa
Pmv16       ctcacccaccttagctatcagaagatgagggcacttctagacaggaaagaaagcccctattacatgctaaataaagataa
Pmv17       ctcacccaccttagctatcagaagatgagggcacttctagacaggaaagaaagcccctattacatgctaaataaagataa
Pmv18       ctcacccaccttagctatcagaagatgagggcacttctagacaggaaaAaaagcccctattacatgctaaataaagataa
Pmv19       ctcacccaccttagctatcagaagatgagggcacttctagacaggaaagaaagcccctattacatgctaaataaagataa
Pmv2        ctcacccaccttagctatcagaagatgagggcacttctagacaggaaagaaagcccctattacatgctaaataaagataa
Pmv20       ctcacccaccttagctatcagaagatgagggcacttctagacaggaaagaaagcccctattacatgctaaataaagataa
Pmv21       ctcacccaccttagctatcagaagatgagggcacttctagacaggaaagaaagcccctattacatgctaaataaagataa
Pmv22       ctcacccaccttagctatcagaagatgagggcacttctagacaggaaagaaagcccctattacatgctaaataaagataa
Pmv23       ctcacccaccttagctatcagaagatgagggcacttctagacaggaaagaaagcccctattacatgctaaataaagataa
Pmv24       ctcacccaccttagctatcagaagatgagggcacttctagacaggaaagaaagcccctattacatgctaaataaagataa
Pmv4        ctcacccaccttagctatcagaagatAagggcacttctagacaAgaaagaaagcccctattacatgctaaataaagataa
Pmv5        ctcacccaccttagctatcagaagatgagggcacttctagacaggaaagaaagcccctattacatgctaaataaagataa
Pmv6        ctcacccaccttagctatcagaagatgagggcacttctagacaggaaagaaagcccctattacatgctaaataaagataa
Pmv7        ctcacccaccttagctatcagaagatgagggcacttctagacaggaaagaaagcccctattacatgctaaataaagataa
Pmv8        --------------------------------------------------------------------------------
Pmv9        ctcacccaccttagctatcagaagatgagggcacttctagacaggaaagaaagcccctattacatgctaaataaagataa
```

```
PMV_CONS      GATCCTCCACGAGGTGGCGGAATCATGCCAAGCCTGTGTCCAAGTAAATGCCAGTAAGACTAAGATCAGGGCCGGAACAC
Pmv1          gatcctccacgaggtggcggaatcatgccaagcctgtgtccaagtaaatgccagtaagactaagatcagggccggaacac
Pmv10         gatcctccacgaggtggcggaatcatgccaagcctgtgtccaagtaaatgccagtaagactaagatcagggccggaacac
Pmv11         gatcctccacgaggtggcggaatcatgccaagcctgtgtccaagtaaatgccagtaagactaagatcagggccggaacac
Pmv12         gatcctccacgaggtggcggaatcatgccaagcctgtgtccaagtaaatgccagtaagactaagatcagggccggaacac
Pmv13         gatcctccacgaggtggcggaatcatgccaagcctgtgtccaagtaaatgccagtaagactaagatcagggccggaacac
Pmv14         gatcctccacgaggtggcggaatcatgccaagcctgtgtccaagtaaatgccagtaagactaagatcagggccggaacac
Pmv15         gatcctccacgaggtggcggaatcatgccaagcctgtgtccaagtaaatgccagtaagactaagatcagggccggaacac
Pmv16         gatcctccacgaggtggcggaatcatgccaagcctgtgtccaagtaaatgccagtaagactaagatcagggccggaacac
Pmv17         gatcctccacgaggtggcggaatcatgccaagcctgtgtccaagtaaatgccagtaagactaagatcagggccggaacac
Pmv18         gatcctccacgaggtggcggaatcatgccaagcctgtgtccaagtaaatgccagtaagactaagatcagggccggaacac
Pmv19         gatcctccacgaggtggcggaatcatgccaagcctgtgtccaagtaaatgccagtaagactaagatcagggccggaacac
Pmv2          gatcctccacgaggtggcggaatcatgccaagcctgtgtccaagtaaatgccagtaagactaagatcagggccggaacac
Pmv20         gatcctccacgaggtggcggaatcatgccaagcctgtgtccaagtaaatgccagtaagactaagatcagggccggaacac
Pmv21         gatcctccacgaggtggcggaatcatgccaagcctgtgtccaagtaaatgccagtaagactaagatcagggccggaacac
Pmv22         gatcctccacgaggtggcggaatcatgccaagcctgtgtccaagtaaatgccagtaagactaagatcagggccggaacac
Pmv23         gatcctccacgaggtggcggaatcatgccaagcctgtgtccaagtaaatgccagtaagactaagatcagggccggaacac
Pmv24         gatcctccacgaggtggcggaatcatgccaagcctgtgtccaagtaaatgccagtaagactaagatcagggccggaacac
Pmv4          gatcctccacgaAgtggcAgaatcatgccaagcctgtgtccaagtaaatgccagtaagactaagatcagggccggaacac
Pmv5          gatcctccacgaggtggcggaatcatgccaagcctgtgtccaagtaaatgccagtaagactaagatcagggccggaacac
Pmv6          gatcctccacgaggtggcggaatcatgccaagcctgtgtccaagtaaatgccagtaagactaagatcagggccggaacac
Pmv7          gatcctccacgaggtggcggaatcatgccaagcctgtgtccaagtaaatgccagtaagactaagatcagggccggaacac
Pmv8          --------------------------------------------------------------------------------
Pmv9          gatcctccacgaggtggcggaatcatgccaagcctgtgtccaagtaaatgccagtaagactaagatcagggccggaacac

PMV_CONS      GAGTAAGAGGACATCGACCAGGCACCCATTGGGAAATTGACTTTACTGAAGTGAAGCCCGGACTGTATGGGTACAAGTAT
Pmv1          gagtaagaggacatcgaccaggcacccattgggaaattgactttactgaagtgaagcccggactgtatgggtacaagtat
Pmv10         gagtaagaggacatcgaccaggcacccattgggaaattgactttactgaagtgaagcccggactgtatgggtacaagtat
Pmv11         gagtaagaggacatcgaccaggcacccattgggaaattgactttactgaagtgaagcccggactgtatgggtacaagtat
Pmv12         gagtaagaggacatcgaccaggcacccattgggaaattgactttactgaagtgaagcccggactgtatgggtacaagtat
Pmv13         gagtaagaggacatcgaccaggcacccattgggaaattgactttactgaagtgaagcccggactgtatgggtacaagtat
Pmv14         gagtaagaggacatcgaccaggcacccattgggaaattgactttactAaagtgaagcccggactgtatgggtacaagtat
Pmv15         gagtaagaggacatcgaccaggcacccattgggaaattgactttactgaagtgaagcccggactgtatgggtacaagtat
Pmv16         gagtaagaggacatcgaccaggcacccattgggaaattgactttactgaagtgaagcccggactgtatgggtacaagtat
Pmv17         gagtaagaggacatcgaccaggcacccattgggaaattgactttactgaagtAaagcccggactgtatgggtacaagtat
Pmv18         gagtaagaggacatcgaccaggcacccattgggaaattgactttactgaagtgaagcccggactgtatgggtacaagtat
Pmv19         gagtaagaggacatcgaccaggcacccattgggaaattgactttactgaagtgaagcccggactgtatgggtacaagtat
Pmv2          gagtaagaggacatcgaccaggcacccattgggaaattgactttactgaagtgaagcccggactgtatgggtacaagtat
Pmv20         gagtaagaggacatcgaccaggcacccattgggaaattgactttactAaagtgaagcccggactgtatgggtacaagtat
Pmv21         gagtaagaggacatcgaccaggcacccattggAaaattgactttactgaagtgaagcccggactgtatgggtacaagtat
Pmv22         gagtaagaggacatcgaccaggcacccattgggaaattgactttactgaagtgaagcccggactgtatgggtacaagtat
Pmv23         gagtaagaggacatcgaccaggcacccattggAaaattgactttactgaagtAaagcccggactgtatgggtacaagtat
Pmv24         gagtaagaggacatcgaccaggcacccattggAaaattgactttactgaagtgaagcccggactgtatgggtacaagtat
Pmv4          gaAtaagaggacatcgaccaggcacccattAAAaaattgactttactgaagtAaagcccggactgtatgggtacaagtat
Pmv5          gagtaagaggacatcgaccaggcacccattgggaaattgactttactgaagtgaagcccggactgtatgggtacaagtat
Pmv6          gagtaagaggacatcgaccaggcacccattgggaaattgactttactgaagtgaagcccggactgtatgggtacaagtat
Pmv7          gagtaagaggacatcgaccaggcacccattgggaaattgactttactgaagtgaagcccggactgtatgggtacaagtat
Pmv8          --------------------------------------------------------------------------------
Pmv9          gagtaagaggacatcgaccaggcacccattgggaaattgactttactgaagtgaagcccggactgtatgggtacaagtat
```

```
PMV_CONS      CTCCTGGTATTCGTGGACACGTTCTCTGGCTGGGTTGAAGCCTTCCCAACCAAACATGAGACTGCCAAAATAGTGACCAA
Pmv1          ctcctggtattcgtggacacgttctctggctgggttgaagccttcccaaccaaacatgagactgccaaaatagtgaccaa
Pmv10         ctcctggtattcgtggacacgttctctggctgggttgaagccttcccaaccaaacatgaAactgccaaaatagtgaccaa
Pmv11         ctcctggtattcgtggacacgttctctggctgggttgaagccttcccaaccaaacatgagactgccaaaatagtgaccaa
Pmv12         ctcctggtattcgtggacacgttctctggctgggttgaagccttcccaaccaaacatgagactgccaaaatagtgaccaa
Pmv13         ctcctggtattcgtggacacgttctctggctgggttgaagccttcccaaccaaacatgaAactgccaaaatagtgaccaa
Pmv14         ctcctggtattcgtggacacgttctctggctgggttgaagccttcccaaccaaacatgagactgccaaaatagtgaccaa
Pmv15         ctcctggtattcgtggacacgttctctggctgggttgaagccttcccaaccaaacatgagactgccaaaatagtgaccaa
Pmv16         ctcctggtattcgtggacacgttctctggctgggttgaagccttcccaaccaaacatgagactgccaaaatagtgaccaa
Pmv17         ctcctggtattcgtggacacgttctctggctgggttgaagccttcccaaccaaacatgagactgccaaaatagtgaccaa
Pmv18         ctcctggtattcgtggacacgttctctggctgggttgaagccttcccaaccaaacatgagactgccaaaatagtgaccaa
Pmv19         ctcctggtattcgtggacacgttctctggctgggttgaagccttcccaaccaaacatgagactgccaaaatagtgaccaa
Pmv2          ctcctggtattcgtggacacgttctctggctgggttgaagccttcccaaccaaacatgagactgccaaaatagtgaccaa
Pmv20         ctcctggtattcgtggacacgttctctggctgggttgaagccttcccaaccaaacatgagactgccaaaatagtgaccaa
Pmv21         ctcctggtattcgtggacacgttctctggctgggttAaagccttcccaaccaaacatgagactgccaaaatagtgaccaa
Pmv22         ctcctggtattcgtggacacgttctctggctgggttgaagccttcccaaccaaacatgagactgccaaaatagtgaccaa
Pmv23         ctcctggtattcgtggacacgttctctggctgggttgaagccttcccaaccaaacatgaAactgccaaaatagtgaccaa
Pmv24         ctcctggtattcgtggacacgttctctggctgggttgaagccttcccaaccaaacatgagactgccaaaatagtgaccaa
Pmv4          ctcctggtattcgtgAacacgttctctggctgggttAaagccttcccaaccaaacatgagactgccaaaatagtgaccaa
Pmv5          ctcctggtattcgtggacacgttctctggctgggttgaagccttcccaaccaaacatgagactgccaaaatagtgaccaa
Pmv6          ctcctggtattcgtggacacgttctctggctgggttgaagccttcccaaccaaacatgaAactgccaaaatagtgaccaa
Pmv7          ctcctggtattcgtggacacgttctctggctgggttgaagccttcccaaccaaacatgagactgccaaaatagtgaccaa
Pmv8          --------------------------------------------------------------------------------
Pmv9          ctcctggtattcgtggacacgttctctggctgggttgaagccttcccaaccaaacatgagactgccaaaatagtgaccaa

PMV_CONS      GAAACTTCTGGAAGAAATATTTCCAAGGTTTGGAATGCCCCAAGTGTTGGGGACTGATAATGGGCCTGCCTTCGTCTCCC
Pmv1          gaaacttctggaagaaatatttccaaggtttggaatgccccaagtgttggggactgataatgggcctgccttcgtctccc
Pmv10         gaaacttctggaagaaatatttccaaggtttggaatgccccaagtgttggggactgataatgggcctgccttcgtctccc
Pmv11         gaaacttctggaagaaatatttccaaggtttggaatgccccaagtgttggggactgataatgggcctgccttcgtctccc
Pmv12         gaaacttctggaagaaatatttccaaggtttggaatgccccaagtgttggggactgataatgggcctgccttcgtctccc
Pmv13         gaaacttctggaagaaatatttccaaggtttggaatgccccaagtgttggggactgataatgggcctgccttcgtctccc
Pmv14         gaaacttctggaaAaaatatttccaaggtttggaatgccccaagtgttggggactgataatgggcctgccttcgtctccc
Pmv15         gaaacttctggaagaaatatttccaaggtttggaatgccccaagtgttggggactgataatgggcctgccttcgtctccc
Pmv16         gaaacttctggaagaaatatttccaaggtttggaatgccccaagtgttggggactgataatgggcctgccttcgtctccc
Pmv17         gaaacttctgAaagaaatatttccaaggtttggaatgccccaagtgttggggactgataatgggcctgccttcgtctccc
Pmv18         gaaacttctggaagaaatatttccaaggtttAgaatgccccaagtgttggggactgataatgggcctgccttcgtctccc
Pmv19         gaaacttctggaagaaatatttccaaggtttggaatgccccaagtgttggggactgataatgggcctgccttcgtctccc
Pmv2          gaaacttctggaagaaatatttccaaggtttggaatgccccaagtgttggggactgataatgggcctgccttcgtctccc
Pmv20         gaaacttctggaagaaatatttccaaggtttggaatgccccaagtgttggggactgataatgggcctgccttcgtctccc
Pmv21         gaaacttctggaagaaatatttccaaggtttggaatgccccaagtgttggggactgataatgggcctgccttcgtctccc
Pmv22         gaaacttctggaagaaatatttccaaggtttggaatgccccaagtgttggggactgataatgggcctgccttcgtctccc
Pmv23         gaaacttctggaaAaaatatttccaaggtttAgaatgccccaagtgttggggactgataatgggcctgccttcgtctccc
Pmv24         gaaacttctggaaAaaatatttccaaggtttggaatgccccaagtgttggggactgataatgggcctgccttcgtctccc
Pmv4          gaaacttctgAaaAaaatatttccaaAgtttAAaatgccccaagtgttggggactgataatgggcctgccttcgtctccc
Pmv5          gaaacttctggaagaaatatttccaaggtttggaatgccccaagtgttggggactgataatgggcctgccttcgtctccc
Pmv6          gaaacttctggaagaaatatttccaaggtttggaatgccccaagtgttggggactgataatgggcctgccttcgtctccc
Pmv7          gaaacttctggaagaaatatttccaaggtttggaatgccccaagtgttggggactgataatgggcctgccttcgtctccc
Pmv8          ------------------------------------------------------------------tgccttcgtctccc
Pmv9          gaaacttctggaaAaaatatttccaaggtttggaatgccccaagtgttggggactgataatgggcctgccttcgtctccc
```

```
PMV_CONS      AGGTAAGTCAGTCGGTGGCCAAGCTACTGGGGATTGATTGGAAACTACATTGTGCTTACAGACCCCAGAGTTCAGGTCAG
Pmv1          aggtaagtcagtcggtggccaagctactggggattgattggaaactacattgtgcttacagaccccagagttcaggtcag
Pmv10         aggtaagtcagtcggtggccaagctactggggattgattggaaactacattgtgcttacagaccccagagttcaggtcag
Pmv11         aggtaagtcagtcggtggccaagctactggggattgattggaaactacattgtgcttacagaccccagagttcaggtcag
Pmv12         aggtaagtcagtcggtggccaagctactggggattgattggaaactacattgtgcttacagaccccagagttcaggtcag
Pmv13         aggtaagtcagtcggtggccaagctactggggattgattggaaactacattgtgcttacagaccccagagttcaggtcag
Pmv14         aggtaagtcagtcggtggccaagctactggggattgattgAaaactacattgtgcttacagaccccagagttcaggtcag
Pmv15         aggtaagtcagtcggtggccaagctactggggattgattggaaactacattgtgcttacagaccccagagttcaggtcag
Pmv16         aggtaagtcagtcggtggccaagctactggggattgattggaaactacattgtgcttacagaccccagagttcaggtcag
Pmv17         aggtaagtcagtcggtggccaagctactggggattgattggaaactacattgtgcttacagaccccagagttcaggtcag
Pmv18         aggtaagtcagtcggtggccaagctactggggattgattggaaactacattgtgcttacagaccccagagttcaggtcag
Pmv19         aggtaagtcagtcggtggccaagctactggggattgattggaaactacattgtgcttacagaccccagagttcaggtcag
Pmv2          aggtaagtcagtcggtggccaagctactggggattgattggaa------------Ctacagaccccagagttcaggtcag
Pmv20         aggtaagtcagtcggtggccaagctactggggattgattggaaactacattgtgcttacagaccccagagttcaggtcag
Pmv21         aggtaagtcagtcggtggccaagctactggggattgattggaaactacattgtgcttacagaccccagagttcaggtcag
Pmv22         aggtaagtcagtcggtggccaagctactggggattgattggaaactacattgtgcttacagaccccagagttcaggtcag
Pmv23         aggtaagtcagtcggtggccaagctactggggattgattgAaaactacattgtgcttacagaccccagagttcaggtcag
Pmv24         aggtaagtcagtcggtggccaagctactggggattgattggaaactacattgtgcttacagaccccagagttcaggtcag
Pmv4          aggtaagtcagtcggtggccaagctactggggattgattAAaaactacattgtgcttacagaccccagagttcaggtcag
Pmv5          aggtaagtcagtcggtggccaagctactggggattgattggaaactacattgtgcttacagaccccagagttcaggtcag
Pmv6          aggtaagtcagtcggtggccaagctactggggattgattggaaactacattgtgcttacagaccccagagttcaggtcag
Pmv7          aggtaagtcagtcggtggccaagctactggggattgattggaaactacattgtgcttacagaccccagagttcaggtcag
Pmv8          aggtaagtcagtcggtggccaagctactggg-attgattggaaactacattgtgcttacagaccccagagttcaggtcag
Pmv9          aggtaagtcagtcggtggccaagctactggggattgattggaaactacattgtgcttacagaccccagagttcaggtcag

PMV_CONS      GTAGAAAGAATGAATAGGACAATCAAGGAGACTTTGACCAAATTAACGCTTGCAACTGGCACTAGAGACTGGGTACTCCT
Pmv1          gtagaaagaatgaataggacaatcaaggagactttgaccaaattaacgcttgcaactggcactagagactgggtactcct
Pmv10         gtagaaagaatgaataggacaatcaaggagactttgaccaaattaacgcttgcaactggcactagagactgggtactcct
Pmv11         gtagaaagaatgaataggacaatcaaggagactttgaccaaattaacgcttgcaactggcactagagactgggtactcct
Pmv12         gtagaaagaatgaataggacaatcaaggagactttgaccaaattaacgcttgcaactggcactagagactgggtactcct
Pmv13         gtagaaagaatgaataggacaatcaaggagactttgaccaaattaacgcttgcaactggcactagagactgggtactcct
Pmv14         gtagaaagaatgaataggacaatcaaggagactttgaccaaattaacgcttgcaactggcactagagactgggtactcct
Pmv15         gtagaaagaatgaataggacaatcaaggagactttgaccaaattaacgcttgcaactggcactagagactgggtactcct
Pmv16         gtagaaagaatgaataggacaatcaaggagactttgaccaaattaacgcttgcaactggcactagagactgggtactcct
Pmv17         gtagaaagaatgaataggacaatcaaggagactttgaccaaattaacgcttgcaactggcactagagactgggtactcct
Pmv18         gtaAaaagaatgaataggacaatcaaggagactttgaccaaattaacgcttgcaactggcactagagactgggtactcct
Pmv19         gtagaaagaatgaataggacaatcaaggagactttgaccaaattaacgcttgcaactggcactagagactgggtactcct
Pmv2          gtagaaagaatgaataggacaatcaaggagactttgaccaaattaacgcttgcaactggcactagagactgggtactcct
Pmv20         gtagaaagaatgaataggacaatcaaggagactttgaccaaattaacgcttgcaactggcactagagactgggtactcct
Pmv21         gtagaaagaatAaataggacaatcaaggagactttgaccaaattaacgcttgcaactggcactagagactgggtactcct
Pmv22         gtagaaagaatgaataggacaatcaaggagactttgaccaaattaacgcttgcaactggcactagagactgggtactcct
Pmv23         gtaAaaaAaatgaataggacaatcaaggagactttgaccaaattaacgcttgcaactggcactagagactgggtactcct
Pmv24         gtaAaaagaatgaataggacaatcaaggagactttgaccaaattaacgcttgcaactggcactagagactgggtactcct
Pmv4          gtaAaaaAaatAaataAgacaatcaaggagactttgaccaaattaacgcttgcaactggcactagagactgggtactcct
Pmv5          gtagaaagaatgaataggacaatcaaggagactttgaccaaattaacgcttgcaactggcactagagactgggtactcct
Pmv6          gtagaaagaatgaataggacaatcaaggagactttgaccaaattaacgcttgcaactggcactagagactgggtactcct
Pmv7          gtagaaagaatgaataggacaatcaaggagactttgaccaaattaacgcttgcaactggcactagagactgggtactcct
Pmv8          gtagaaagaatgaataggacaatcaaggagactttgaccaaattaacgcttgcaactggcactagagactgggtactcct
Pmv9          gtagaaagaatgaataggacaatcaaggagactttgaccaaattaacgcttgcaactggcactagagactgggtactcct
```

```
PMV_CONS    ACTTCCCTTAGCCCTCTACCGAGCCCGCAACACTCCGGGCCCCCATGGACTCACTCCGTATGAAATCCTGTATGGGGCGC
Pmv1        acttcccttagccctctaccgagcccgcaacactccgggcccccatggactcactccgtatgaaatcctgtatggggcgc
Pmv10       acttcccttagccctctaccgagcccgcaacactccgggcccccatggactcactccgtatgaaatcctgtatggggcgc
Pmv11       acttcccttagccctctaccgagcccgcaacactccgggcccccatggactcactccgtatgaaatcctgtatggggcgc
Pmv12       acttcccttagccctctaccgagcccgcaacactccgggcccccatggactcactccgtatgaaatcctgtatggggcgc
Pmv13       acttcccttagccctctaccgagcccgcaacactccgggcccccatggactcactccgtatgaaatcctgtatggggcgc
Pmv14       acttcccttagccctctaccgagcccgcaacactccAggcccccatggactcactccgtatgaaatcctgtatggggcgc
Pmv15       acttcccttagccctctaccgagcccgcaacactccgggcccccatggactcactccgtatgaaatcctgtatggggcgc
Pmv16       acttcccttagccctctaccgagcccgcaacactccgggcccccatggactcactccgtatgaaatcctgtatggggcgc
Pmv17       acttcccttagccctctaccgagcccgcaacactccgggcccccatggactcactccgtatgaaatcctgtatggggcgc
Pmv18       acttcccttagccctctaccgagcccgcaacactccgggcccccatggactcactccgtatgaaatcctgtatggggcgc
Pmv19       acttcccttagccctctaccgagcccgcaacactccgggcccccatggactcactccgtatgaaatcctgtatggggcgc
Pmv2        acttcccttagccctctaccgagcccgcaacactccgggcccccatggactcactccgtatgaaatcctgtatggggcgc
Pmv20       acttcccttagccctctaccgagcccgcaacactccgggcccccatggactcactccgtatgaaatcctgtatggggcgc
Pmv21       acttcccttagccctctaccgagcccgcaacactccgggcccccatggactcactccgtatgaaatcctgtatggggcgc
Pmv22       acttcccttagccctctaccgagcccgcaacactccgggcccccatggactcactccgtatgaaatcctgtatggggcgc
Pmv23       acttcccttagccctctaccgagcccgcaacactccgggcccccatggactcactccgtatgaaatcctgtatggggcgc
Pmv24       acttcccttagccctctaccgagcccgcaacactccgggcccccatggactcactccgtatgaaatcctgtatggggcgc
Pmv4        acttcccttagccctctaccgagcccgcaacactccgggcccccatAgactcactccgtatAaaatcctgtatggggcgc
Pmv5        acttcccttagccctctaccgagcccgcaacactccgggcccccatggactcactccgtatgaaatcctgtatggggcgc
Pmv6        acttcccttagccctctaccgagcccgcaacactccgggcccccatggactcactccgtatgaaatcctgtatggggcgc
Pmv7        acttcccttagccctctaccgagcccgcaacactccgggcccccatggactcactccgtatgaaatcctgtatggggcgc
Pmv8        acttcccttagccctctaccgagcccgcaacactccgggcccccatggactcactccgtatgaaatcctgtatggggcgc
Pmv9        acttcccttagccctctaccgagcccgcaacactccgggcccccatggactcactccgtatgaaatcctgtatggggcgc

PMV_CONS    CCCCGCCCCTTGTTAATTTCCATGATCCTGAAATGTCAAAGTTTACTAATAGCCCCTCTCTCCAAGCTCACTTACAGGCC
Pmv1        ccccgccccttgttaatttccatgatcctgaaatgtcaaagtttactaatagcccctctctccaagctcacttacaggcc
Pmv10       ccccgccccttgttaatttccatgatcctgaaatgtcaaagtttactaatagcccctctctccaagctcacttacaggcc
Pmv11       ccccgccccttgttaatttccatgatcctgaaatgtcaaagtttactaatagcccctctctccaagctcacttacaggcc
Pmv12       ccccgccccttgttaatttccatgatcctgaaatgtcaaagtttactaatagcccctctctccaagctcacttacaggcc
Pmv13       ccccgccccttgttaatttccatgatcctgaaatgtcaaagtttactaatagcccctctctccaagctcacttacaggcc
Pmv14       ccccgccccttgttaatttccatgatcctgaaatgtcaaagtttactaatagcccctctctccaagctcacttacaggcc
Pmv15       ccccgccccttgttaatttccatgatcctgaaatgtcaaagtttactaatagcccctctctccaagctcacttacaggcc
Pmv16       ccccgccccttgttaatttccatgatcctgaaatgtcaaagtttactaatagcccctctctccaagctcacttacaggcc
Pmv17       ccccgccccttgttaatttccatgatcctgaaatgtcaaagtttactaatagcccctctctccaagctcacttacaggcc
Pmv18       ccccgccccttgttaatttccatgatcctgaaatgtcaaagtttactaatagcccctctctccaagctcacttacaggcc
Pmv19       ccccgccccttgttaatttccatgatcctgaaatgtcaaagtttactaatagcccctctctccaagctcacttacaggcc
Pmv2        ccccgccccttgttaatttccatgatcctgaaatgtcaaagtttactaatagcccctctctccaagctcacttacaggcc
Pmv20       ccccgccccttgttaatttccatgatcctgaaatgtcaaagtttactaatagcccctctctccaagctcacttacaggcc
Pmv21       ccccgccccttgttaatttccatgatcctgaaatgtcaaagtttactaatagcccctctctccaagctcacttacaggcc
Pmv22       ccccgccccttgttaatttccatgatcctgaaatgtcaaagtttactaatagcccctctctccaagctcacttacaggcc
Pmv23       ccccgccccttgttaatttccatgatcctgaaatgtcaaagtttactaatagcccctctctccaagctcacttacaggcc
Pmv24       ccccgccccttgttaatttccatgatcctgaaatgtcaaagtttactaatagcccctctctccaagctcacttacaggcc
Pmv4        ccccgccccttgttaatttccatgatcctgaaatgtcaaagtttactaatagcccctctctccaagctcacttacaggcc
Pmv5        ccccgccccttgttaatttccatgatcctgaaatgtcaaagtttactaatagcccctctctccaagctcacttacaggcc
Pmv6        ccccgccccttgttaatttccatgatcctgaaatgtcaaagtttactaatagcccctctctccaagctcacttacaggcc
Pmv7        ccccgccccttgttaatttccatgatcctgaaatgtcaaagtttactaatagcccctctctccaagctcacttacaggcc
Pmv8        ccccgccccttgttaatttccatgatcctgaaatgtcaaagtttactaatagcccctctctccaagctcacttacaggcc
Pmv9        ccccgccccttgttaatttccatgatcctgaaatgtcaaagtttactaatagcccctctctccaagctcacttacaggcc
```

```
PMV_CONS    CTCCAAGCAGTACAACGAGAGGTCTGGAAGCCACTGGCCGCTGCCTATCAGGACCAGCTGGACCAGCCAGTGATACCACA
Pmv1        ctccaagcagtacaacgagaggtctggaagccactggccgctgcctatcaggacTagctggaccagccagtgataccaca
Pmv10       ctccaagcagtacaacgagaggtctggaagccactggccgctgcctatcaggaccagctggaccagccagtgataccaca
Pmv11       ctccaagcagtacaacgagaggtctggaagccactggccgctgcctatcaggaccagctggaccagccagtgataccaca
Pmv12       ctccaagcagtacaacgagaggtctggaagccactggccgctgcctatcaggaccagctggaccagccagtgataccaca
Pmv13       ctccaagcagtacaacgagaggtctggaagccactggccgctgcctatcaggaccagctggaccagccagtgataccaca
Pmv14       ctccaagcagtacaacgagaggtctggaagccactggccgctgcctatcaggaccagctAgaccagccagtgataccaca
Pmv15       ctccaagcagtacaacgagaggtctggaagccactggccgctgcctatcaggaccagctggaccagccagtgataccaca
Pmv16       ctccaagcagtacaacgagaggtctggaagccactggccgctgcctatcaggaccagctggaccagccagtgataccaca
Pmv17       ctccaagcagtacaacgagaggtctggaagccactggccgctgcctatcaggaccagcAggaccagccagtgataccaca
Pmv18       ctccaagcagtacaacgagaggtctggaagccactggccgctgcctatcaggaccagctggaccagccagtgataccaca
Pmv19       ctccaagcagtacaacgagaggtctggaagccactggccgctgcctatcaggaccagctggaccagccagtgataccaca
Pmv2        ctccaagcagtacaacgagaggtctggaagccactggccgctgcctatcaggaccagctggaccagccagtgataccaca
Pmv20       ctccaagcagtacaacgagaggtctggaagccactggccgctgcctatcaggaccagctggaccagccagtgataccaca
Pmv21       ctccaagcagtacaacgagaggtctggaagccactggccgctgcctatcaggaccagctggaccagccagtgataccaca
Pmv22       ctccaagcagtacaacgagaggCctggaagccactggccgctgcctatcaggaccagctggaccagccagtgataccaca
Pmv23       ctccaagcagtacaacgagaggtctggaagccactggccgctgcctatcaggaccagctggaccagccagtgataccaca
Pmv24       ctccaagcagtacaacgagaggtctggaagccactggccgctgcctatcaggaccagctggaccagccagtgataccaca
Pmv4        ctccaagcagtacaacgagaggtctAgaagccactggccgctgcctatcaggaccagctggaccagccagtAataccaca
Pmv5        ctccaagcagtacaacgagaggtctggaagccactggccgctgcctatcaggaccagctggaccagccagtgataccaca
Pmv6        ctccaagcagtacaacgagaggtctggaagccactggccgctgcctatcaggaccagctggaccagccagtgataccaca
Pmv7        ctccaagcagtacaacgagaggtctggaagccactggccgctgcctatcaggaccagctggaccagccagtgataccaca
Pmv8        ctccaagcagtacaacgagaggtctggaagccactggccgctgcctatcaggaccagctggaccagccagtgataccaca
Pmv9        ctccaagcagtacaacgagaggtctggaagccactggccgctgcctatcaggaccagctggaccagccagtgataccaca
```

```
PMV_CONS    CCCCTTCCGTGTCGGCGACACCGTGTGGGTACGCCGGCACCAGACTAAGAACTTGGAACCTCGCTGGAAAGGACCCTACA
Pmv1        ccccttccgtgtcggcgacaccgtgtgggtacgccggcaccagactaagaacttggaacctcgctggaaaggaccctaca
Pmv10       ccccttccgtgtcggcgacaccgtgtgggtacgccggcaccagactaagaacttggaacctcgctggaaaggaccctaca
Pmv11       ccccttccgtgtcggcgacaccgtgtgggtacgccggcaccagactaagaacttggaacctcgctggaaaggaccctaca
Pmv12       ccccttccgtgtcggcgacaccgtgtgggtacgccggcaccagactaagaacttggaacctcgctggaaaggaccctaca
Pmv13       ccccttccgtgtcggcgacaccgtgtgggtacgccggcaccagactaagaacttggaacctcgctggaaaggaccctaca
Pmv14       ccccttccgtgtcggcgacaccgtgtgggtacgccggcaccagactaaAaacttggaacctcgctggaaaggaccctaca
Pmv15       ccccttccgtgtcggcgacaccgtgtgggtacgccggcaccagactaagaacttggaacctcgctggaaaggaccctaca
Pmv16       ccccttccgtgtcggcgacaccgtgtgggtacgccggcaccagactaagaacttggaacctcgctggaaaggaccctaca
Pmv17       ccccttccgtgtAggcgacaccgtgtgggtacgccggcaccagactaagaacttggaacctcgctggaaaggaccctaca
Pmv18       ccccttccgtgtcggcgacaccgtgtgggtacgccggcaccagactaagaacttggaacctcgctggaaaggaccctaca
Pmv19       ccccttccgtgtcggcgacaccgtgtgggtacgccggcaccagactaagaacttggaacctcgctggaaaggaccctaca
Pmv2        ccccttccgtgtcggcgacaccgtgtgggtacgccggcaccagactaagaacttggaacctcgctggaaaggaccctaca
Pmv20       ccccttccgtgtcggcgacaccgtgtgggtacgccggcaccagactaagaacttggaacctcgctggaaaggaccctaca
Pmv21       ccccttccgtgtcggcgacaccgtgtgggtacgccggcaccagactaagaacttggaacctcgctAgaaaggaccctaca
Pmv22       ccccttccgtgtcggcgacaccgtgtgggtacgccggcaccagactaagaacttggaacctcgctggaaaggaccctaca
Pmv23       ccccttccgtgtcggcgacaccgtgtgggtacgccggcaccagactaagaacttggaacctcgctggaaaggaccctaca
Pmv24       ccccttccgtgtcggcgacaccgtgtgggtacgccggcaccagactaagaacttggaacctcgctggaaaggaccctaca
Pmv4        ccccttccgtgtcggcgacaccgtgtgggtacgccggcaccagactaagaacttAgaacctcgctAAaaaggaccctaca
Pmv5        ccccttccgtgtcggcgacaccgtgtgggtacgccggcaccagactaagaacttggaacctcgctggaaaggaccctaca
Pmv6        ccccttccgtgtcggcgacaccgtgtgggtacgccggcaccagactaagaacttggaacctcgctggaaaggaccctaca
Pmv7        ccccttccgtgtcggcgacaccgtgtgggtacgccggcaccagactaagaacttggaacctcgctggaaaggaccctaca
Pmv8        ccccttccgtgtcggcgacaccgtgtgggtacgccggcaccagactaaAaacttggaacctcgctggaaaggaccctaca
Pmv9        ccccttccgtgtcggcgacaccgtgtgggtacgccggcaccagactaagaacttggaacctcgctggaaaggaccctaca
```

```
PMV_CONS      CCGTCCTGCTGACCACCCCCACCGCTCTCAAAGTAGACGGCATCGCTGCGTGGATCCACGCCGCTCACGTAAAAGCGGCG
Pmv1          ccgtcctgctgaccacccccaccgctctcaaagtagacggcatcgctgcgtggatccacgccgctcacgtaaaagcggcg
Pmv10         ccgtcctgctgaccacccccaccgctctcaaagtagacggcatcgctgcgtggatccacgccgctcacgtaaaagcggcg
Pmv11         ccgtcctgctgaccacccccaccgctctcaaagtagacggcatcgctgcgtggatccacgccgctcacgtaaaagcggcg
Pmv12         ccgtcctgctgaccacccccaccgctctcaaagtagacggcatcgctgcgtggatccacgccgctcacgtaaaagcggcg
Pmv13         ccgtcctgctgaccacccccaccgctctcaaagtagacggcatcgctgcgtggatccacgccgctcacgtaaaagcggcg
Pmv14         ccgtcctgctgaccacccccaccgctctcaaagtagacggcatcgctgcgtggatccacgccgctcacAtaaaagcggcg
Pmv15         ccgtcctgctgaccacccccaccgctctcaaagtagacggcatcgctgcgtggatccacgccgctcacgtaaaagcggcg
Pmv16         ccgtcctgctgaccacccccaccgctctcaaagtagacggcatcgctgcgtggatccacgccgctcacgtaaaagcggcg
Pmv17         ccgtcctgctgaccacccccaccgctctcaaagtagacggcatcgctgcgtggatccacgccgctcacgtaaaagcggcg
Pmv18         ccgtcctgctgaccacccccaccgctctcaaagtagacggcatcgctgcgtggatccacgccgctcacgtaaaagcggcg
Pmv19         ccgtcctgctgaccacccccaccgctctcaaagtagacggcatcgctgcgtggatccacgccgctcacgtaaaagcggcg
Pmv2          ccgtcctgctgaccacccccaccgctctcaaagtagacggcatcgctgcgtggatccacgccgctcacgtaaaagcggcg
Pmv20         ccgtcctgctgaccacccccaccgctctcaaagtagacggcatcgctgcgtggatccacgccgctcacgtaaaagcggcg
Pmv21         ccgtcctgctgaccacccccaccgctctcaaagtagacggcatcgctgcgtggatccacgccgctcacgtaaaagcggcg
Pmv22         ccgtcctgctgaccacccccaccgctctcaaagtagacggcatcgctgcgtggatccacgccgctcacgtaaaagcggcg
Pmv23         ccgtcctgctgaccacccccaccgctctcaaagtagacggcatcgctgcgtggatccacgccgctcacgtaaaagcggcg
Pmv24         ccgtcctgctgaccacccccaccgctctcaaagtagacggcatcgctgcgtggatccacgccgctcacgtaaaagcggcg
Pmv4          ccgtcctgctgaccacccccaccgctctcaaagtagacggcatcgctgcgtggatccacgccgctcacgtaaaagcggcg
Pmv5          ccgtcctgctgaccacccccaccgctctcaaagtagacggcatcgctgcgtggatccacgccgctcacgtaaaagcggcg
Pmv6          ccgtcctgctgaccacccccaccgctctcaaagtagacggcatcgctgcgtggatccacgccgctcacgtaaaagcggcg
Pmv7          ccgtcctgctgaccacccccaccgctctcaaagtagacggcatcgctgcgtggatccacgccgctcacgtaaaagcggcg
Pmv8          ccgtcctgctgaccacccccaccgctctcaaagtagacggcatcgctgcgtggatccacgccgctcacgtaaaagcggcg
Pmv9          ccgtcctgctgaccacccccaccgctctcaaagtagacggcatcgctgcgtggatccacgccgctcacgtaaaagcggcg

PMV_CONS      ACAACCCCTCCGGCCGGAACAGCATCAGGACCGACATGGAAGGTCCAGCGTTCTCAAAACCCCTTAAAGATAAGATTAAC
Pmv1          acaacccctccggccggaacagcatcaggaccgacatggaaggtccagcgttctcaaaaccccttaaagataagattaac
Pmv10         acaacccctccggccggaacagcatcaggaccgacatggaaggtccagcgttctcaaaaccccttaaagataagattaac
Pmv11         acaacccctccggccggaacagcatcaggaccgacatggaaggtccagcgttctcaaaaccccttaaagataagattaac
Pmv12         acaacccctccggccggaacagcatcaggaccgacatggaaggtccagcgCtctcaaaaccccttaaagataagattaac
Pmv13         acaacccctccggccggaacagcatcaggaccgacatggaaggtccagcgttctcaaaaccccttaaagataagattaac
Pmv14         acaacccctccggccggaacagcatcaggaccgacatggaaggtccagcgttctcaaaaccccttaaagataagattaac
Pmv15         acaacccctccggccggaacagcatcaggaccgacatAgaaggtccagcgttctcaaaaccccttaaagataagattaac
Pmv16         acaacccctccggccggaacagcatcaggaccgacatggaaggtccagcgttctcaaaaccccttaaagataagattaac
Pmv17         acaacccctccggccggaacagcatcaggaccgacatggaaggtccagcgttctcaaaaccccttaaagataagattaac
Pmv18         acaacccctccggccggaacagcatcaggaccgacatggaaggtccagcgttctcaaaaccccttaaagataagattaac
Pmv19         acaacccctccggccggaacagcatcaggaccgacatggaaggtccagcgttctcaaaaccccttaaagataagattaac
Pmv2          acaacccctccggccggaacagcatcaggaccgacatggaaggtccagcgttctcaaaaccccttaaagataagattaac
Pmv20         acaacccctccggccggaacagcatcaggaccgacatggaaggtccagcgttctcaaaaccccttaaagataagattaac
Pmv21         acaacccctccggccggaacagcatcaggaccgacatgAaaggtccagcgCtctcaaaaccccttaaagataagattaac
Pmv22         acaacccctccggccggaacagcatcaggaccgacatggaaggtccagcgttctcaaaaTcccttaaagataagattaac
Pmv23         acaacccctccggccggaacagcatcaggaccgacatggaaggtccagcgttctcaaaaccccttaaagataagattaac
Pmv24         acaacccctccggccggaacagcatcaggaccgacatgAaaggtccagcgttctcaaaaccccttaaagataagattaac
Pmv4          acaacccctccggccggaacagcatcaggaccgacatAAaaAgtccagcgttctcaaaaccccttaaagataagattaac
Pmv5          acaacccctccggccggaacagcatcaggaccgacatggaaggtccagcgttctcaaaaccccttaaagataagattaac
Pmv6          acaacccctccggccggaacagcatcaggaccgacatggaaggtccagcgttctcaaaaccccttaaagataagattaac
Pmv7          acaacccctccggccggaacagcatcaggaccgacatggaaggtccagcgttctcaaaaccccttaaagataagattaac
Pmv8          acaacccctccggccggaacagcatcaggaccgacatgAaaggtccagcgttctcaaaaccccttaaagataagattaac
Pmv9          acaacccctccggccggaacagcatcaggaccgacatgAaaggtccagcgttctcaaaaccccttaaagataagattaac
```

```
PMV_CONS   CCGTGGGGCCCCCATGGAAGGTCCAGCGTTCTCAAAACCCCTTAAAGATAAGATTAACCCGTGGGGCCCCCTAATAATCC
Pmv1       ccgtggggcccccatggaaggtccagcgttctcaaaaccccttaaagataagattaacccgtggggccccctaataatcc
Pmv10      ccgtggggcccccatggaaggtccagcgttctcaaaaccccttaaagataagattaacccgtggggccccctaataGtcc
Pmv11      ccgtggggcccccatggaaggtccagcgttctcaaaaccccttaaagataagattaacccgtggggccccctaataatcc
Pmv12      ccgtggggcccccatggaaggtccagcgCtctcaaaaccccttaaagataagattaacccgtggggccccctaataGtcc
Pmv13      ccgtggggcccccatggaaggtccagcgttctcaaaaccccttaaagataagattaacccgtggggccccctaataGtcc
Pmv14      ccgtggggcccccatggaaggtccagcgttctcaaaaccccttaaagataagattaacccgtggggccccctaataGtcc
Pmv15      ccgtggggcccccatAgaaggtccagcgttctcaaaaccccttaaagataagattaacccgtggggccccctaataGtcc
Pmv16      ccgtggggcccccatggaaggtccagcgttctcaaaaccccttaaagataagattaacccgtggggccccctaataatcc
Pmv17      ccgtggggcccccatggaaggtccagcgttctcaaaaccccttaaagataagattaacccgtggggccccctaataGtcc
Pmv18      ccgtggggcccccatggaaggtccagcgttctcaaaaccccttaaagataagattaacccgtggggccccctaataatcc
Pmv19      ccgtggggcccccatggaaggtccagcgttctcaaaaccccttaaagataagattaacccgtggggccccctaataatcc
Pmv2       ccgtggggcccccatggaaggtccagcgttctcaaaaccccttaaagataagattaacccgtggggccccctaataatcc
Pmv20      ccgtggggcccccatggaaggtccagcgttctcaaaaccccttaaagataagattaacccgtggggccccctaataatcc
Pmv21      ccgtggggcccccatgAaaggtccagcgCtctcaaaaccccttaaagataagattaacccgtggggccccctaataGtcc
Pmv22      ccgtggggcccccatggaaggtccagcgttctcaaaaTcccttaaagataagattaacccgtggggccccctaataatcc
Pmv23      ccgtggggcccccatggaaggtccagcgttctcaaaaccccttaaagataagattaacccgtggggccccctaataGtcc
Pmv24      ccgtggggcccccatgAaaggtccagcgttctcaaaaccccttaaagataagattaacccgtggggccccctaataatcc
Pmv4       ccgtggggcccccatAAaaAgtccagcgttctcaaaaccccttaaagataagattaacccgtggggccccctaataGtcc
Pmv5       ccgtggggcccccatggaaggtccagcgttctcaaaaccccttaaagataagattaacccgtggggccccctaataatcc
Pmv6       ccgtggggcccccatggaaggtccagcgttctcaaaaccccttaaagataagattaacccgtggggccccctaataGtcc
Pmv7       ccgtggggcccccatggaaggtccagcgttctcaaaaccccttaaagataagattaacccgtggggccccctaataatcc
Pmv8       ccgtggggcccccatgAaaggtccagcgttctcaaaaccccttaaagataagattaacccgtggggccccctaataatcc
Pmv9       ccgtggggcccccatgAaaggtccagcgttctcaaaaccccttaaagataagattaacccgtggggccccctaataGtcc

PMV_CONS   TGGGGATCTTAATAAGGGCAGGAGTATCAGTACAACATGACAGCCCTCATCAGGTCTTCAATGTTACTTGGAGAGTTACC
Pmv1       tggggatcttaataagAgcaggagtatcagtacaacatgacagccctcatcaggtcttcaatgttacttggagagttacc
Pmv10      tgAgAatcttaataagggcaggagtatcagtacaacatgacagccctcatcaggtcttcaatgttacttggagagttacc
Pmv11      tggggatcttaataagAgcaggagtatcagtacaacatgacagccctcatcaggtcttcaatgttacttggagagttacc
Pmv12      tggggatcttaataagggcaggagtatcagtacaacatgacagccctcatcaggtcttcaatgttacttggagagttacc
Pmv13      tgggAatcttaataagggcaggagtatcagtacaacatgacagccctcatcaggtcttcaatgttacttggagagttacc
Pmv14      tggggatcttaataagggcaggagtatcagtacaacatgacagccctcatcaggtcttcaatgttacttAgagagttacc
Pmv15      tggggatcttaataagggcaggagtatcagtacaacatgacagccctcatcaggtcttcaatgttacttggagagttacc
Pmv16      tggggatcttaataagAgcaggagtatcagtacaacatgacagccctcatcaggtcttcaatgttacttggagagttacc
Pmv17      tggggatcttaataagggcaggagtatcagtacaacatgacagccctcatcaggtcttcaatgttacttggagagttacc
Pmv18      tggggatcttaataagggcaggagtatcagtacaacatgacagccctcatcaggtcttcaatgttacttggagagttacc
Pmv19      tggggatcttaataagggcaggagtatcagtacaacatgacagccctcatcaggtcttcaatgttacttggagagttacc
Pmv2       tggggatcttaataagggcaggagtatcagtacaacatgacagccctcatcaggtcttcaatgttacttggagagttacc
Pmv20      tggggatcttaataagggcaggagtatcagtacaacatgacagccctcatcaggtcttcaatgttacttggagagttacc
Pmv21      tggggatcttaataagggcaggagtatcagtacaacatgacagccctcatcaggtcttcaatgttacttggagagttacc
Pmv22      tggggatcttaataagggcaggagtatcagtacaacatgacagccctcatcaggtcttcaatgttacttggagagttacc
Pmv23      tgggAatcttaataagggcaggagtatcagtacaacatgacagccctcatcaggtcttcaatgttacttggagagttacc
Pmv24      tggggatcttaataagggcaggagtatcagtacaacatgacagccctcatcaggtcttcaatgttacttggagagttacc
Pmv4       tggggatcttaataagggcaggagtatcagtacaacatgacagccctcatcaggtcttcaatgttacttAAaAagttacc
Pmv5       tggggatcttaataagAgcaggagtatcagtacaacatgacagccctcatcaggtcttcaatgttacttggagagttacc
Pmv6       tgggAatcttaataagggcaggagtatcagtacaacatgacagccctcatcaggtcttcaatgttacttggagagttacc
Pmv7       tggggatcttaataagggcaggagtatcagtacaacatgacagccctcatcaggtcttcaatgttacttggagagttacc
Pmv8       tggggatcttaataagAgcaggagtatcagtacaacatgacagccctcatcaggtcttcaatgttacttAgagagttacc
Pmv9       tggggatcttaataagggcaggagtatcagtacaacatgacagccctcatcaggtcttcaatgttacttggagagttacc
```

```
PMV_CONS      AACTTAATGACAGGACAAACAGCTAATGCTACCTCCCTCCTGGGGACAATGACCGATGCCTTTCCTAAACTGTACTTTGA
Pmv1          aacttaatgacaggacaaacagctaatgctacctccctcctggggacaatgaccgatgcctttcctaaactgtactttga
Pmv10         aacttaatgacaggacaaacagctaatgctacctccctcctggggacaatgaccgatgcctttcctaaactgtactttga
Pmv11         aacttaatgacaggacaaacagctaatgctacctccctcctggggacaatgaccgatgcctttcctaaactgtactttga
Pmv12         aacttaatgacaggacaaacagctaatgctacctccctcctggggacaatgaccgatgcctttcctaaactgtactttga
Pmv13         aacttaatgacaggacaaacagctaatgctacctccctcctggggacaatgaccgatgcctttcctaaactgtactttga
Pmv14         aacttaatgacaggacaaacagctaatgctacctccctcctggggacaatgaccgatgcctttcctaaactgtactttga
Pmv15         aacttaatgacaggacaaacagctaatgctacctccctcctggggacaatgaccgatgcctttcctaaactgtactttga
Pmv16         aacttaatgacaggacaaacagctaatgctacctccctcctggggacaatgaccgatgcctttcctaaactgtactttga
Pmv17         aacttaatgacaggacaaacagctaatgctacctccctcctggggacaatgaccgatgcctttcctaaactgtactttga
Pmv18         aacttaatgacaggacaaacagctaatgctacctccctcctggggacaatgaccgatgcctttcctaaactgtactttga
Pmv19         aacttaatgacaggacaaacagctaatgctacctccctcctggggacaatgaccgatgcctttcctaaactgtactttga
Pmv2          aacttaatgacaggacaaacagctaatgctacctccctcctggggacaatgaccgatgcctttcctaaactgtactttga
Pmv20         aacttaatgacaggacaaacagctaatgctacctccctcctggggacaatgaccgatgcctttcctaaactgtactttga
Pmv21         aacttaatgacaggacaaacagctaatgctacctccctcctggggacaatgaccgatgcctttcctaaactgtactttga
Pmv22         aacttaatgacaggacaaacagctaatgctacctccctcctggggacaatgaccgatgcctttcctaaactgtactttga
Pmv23         aacttaatgacaggacaaacagctaatgctacctccctcctggggacaatgaccgatgcctttcctaaactgtactttga
Pmv24         aacttaatgacaggacaaacagctaatgctacctccctcctggggacaatgaccgatgcctttcctaaactgtactttga
Pmv4          aacttaatgacaggacaaacagctaatgctacctccctcctggggacaatgaccgatgcctttcctaaactgtactttga
Pmv5          aacttaatgacaggacaaacagctaatgctacctccctcctggggacaatgaccgatgcctttcctaaactgtactttga
Pmv6          aacttaatgacaggacaaacagctaatgctacctccctcctggggacaatgaccgatgcctttcctaaactgtactttga
Pmv7          aacttaatgacaggacaaacagctaatgctacctccctcctggggacaatgaccgatgcctttcctaaactgtactttga
Pmv8          aacttaatgacaggacaaacagctaatgctacctccctcctggggacaatgaccgatgcctttcctaaactgtactttga
Pmv9          aacttaatgacaggacaaacagctaatgctacctccctcctggggacaatgaccgatgcctttcctaaactgtactttga

PMV_CONS      CTTGTGCGATTTAATAGGGGACGACTGGGATGAGACTGGACTCGGGTGTCGCACTCCCGGGGGAAGAAAAAGGGCAAGAA
Pmv1          cttgtgcgatttaataggggacgactgggatgagactggactcgggtgtcgcactcccgggggaagaaaaagggcaagaa
Pmv10         cttgtgcgatttaataggggacgactgggatgagactggactcgggtgtcgcactcccgggggaagaaaaagggcaagaa
Pmv11         cttgtgcgatttaataggggacgactgggatgagactggactcgggtgtcgcactcccgggggaagaaaaagggcaagaa
Pmv12         cttgtgcgatttaataggggacgactgggatgagactggactcgggtgtcgcactcccgggggaagaaaaagggcaagaa
Pmv13         cttgtgcgatttaataggggacgactgggatgagactggactcgggtgtcgcactcccgggggaagaaaaagggcaagaa
Pmv14         cttgtgcgatttaataggggacgactgggatgagactggactcgggtgtcgcactcccgggggaagaaaaagggcaagaa
Pmv15         cttgtgcgatttaataggggacgactgggatgagactggactcgggtgtcgcactcccgggggaagaaaaagggcaagaa
Pmv16         cttgtgcgatttaataggggacgactgggatgagactggactcgggtgtcgcactcccgggggaagaaaaagggcaagaa
Pmv17         cttgtgcgatttaataggggacgaTtgggatgagactggactcgggtgtcgcactcccggggAaagaaaaagggcaagaa
Pmv18         cttgtgcgatttaataggggacgactgggatgagactggactcgggtgtcgcactcccgggggaagaaaa----------
Pmv19         cttgtgcgatttaataggggacgactgggatgagactggactcgggtgtcgcactcccgggggaagaaaaagggcaagaa
Pmv2          cttgtgcgatttaataggggacgactgggatgagactggactcgggtgtcgcactcccgggggaagaaaaagggcaagaa
Pmv20         cttgtgcgatttaataggggacgactgggatgagactggactcgggtgtcgcactcccgggggaagaaaaagggcaagaa
Pmv21         cttgtgcgatttaataggggacgactgggatgagactggactcgggtgtcgcactcccgggggaagaaaaagggcaagaa
Pmv22         cttgtgcgatttaataggggacgactgggatgagactggactcgggtgtcgcactcccgggggaagaaaaagggcaagaa
Pmv23         cttgtgcgatttaataggggacgactgggatgagactggactcgggtgtcgcactcccgggggaagaaaaagggcaaAaa
Pmv24         cttgtgcgatttaataggggacgactgggatgagactggactcgggtgtcgcactcccgggggaagaaaaagggcaagaa
Pmv4          cttgtgcgatttaataggggacgactgAgatgagactggactcgggtgtcgcactcccggggAaaAaaaaagggcaagaa
Pmv5          cttgtgcgatttaataggggacgactgggatgagactggactcgggtgtcgcactcccgggggaagaaaaagggcaagaa
Pmv6          cttgtgcgatttaataggggacgactgggatgagactggactcgggtgtcgcactcccgggggaagaaaaagggcaagaa
Pmv7          cttgtgcgatttaataggggacgactgggatgagactggactcgggtgtcgcactcccggggAaagaaaaagggcaagaa
Pmv8          cttgtgcgatttaataggggacgactgggatgagactggactcgggtgtcgcactcccgggggaagaaaaagggcaagaa
Pmv9          cttgtgcgatttaataggggacgactgggatgagactggactcgggtgtcgcactcccgggggaagaaaaagggcaagaa
```

```
PMV_CONS      CATTTGACTTCTATGTTTGCCCCGGGCATACTGTACCAACAGGGTGTGGAGGGCCGAGAGAGGGCTACTGTGGCAAATGG
Pmv1          catttgacttctatgtttgccccgggcatactgtaccaacagggtgtggagggccgagagagggctactgtggcaaatgg
Pmv10         catttgacttctatgtttgccccgggcatactgtaccaacagggtgtggagggccgagagagggctactgtggcaaatgg
Pmv11         catttgacttctatgtttgccccgggcatactgtaccaacagggtgtggagggccgagagagggctactgtggcaaatgg
Pmv12         catttgacttctatgtttgccccgggcatactgtaccaacagggtgtggagggccgagagagggctactgtggcaaatgg
Pmv13         catttgacttctatgtttgccccgggcatactgtaccaacagggtgtggagggccgagagagggctactgtggcaaatgg
Pmv14         catttgacttctatgtttgccccgggcatactgtaccaacagggtgtggagggccgagagagggctactgtggcaaatgg
Pmv15         catttgacttctatgtttgccccgggcatactgtaccaacagggtgtggagggccgagagagggctactgtggcaaatgg
Pmv16         catttgacttctatgtttgccccgggcatactgtaccaacagggtgtggagggccgagagagggctactgtggcaaatgg
Pmv17         catttgacttctatgtttgccccgggcatactgtaccaacagggtgtggagggccgagagagggctactgtggcaaatgg
Pmv18         --------------------------------------------------------------------------------
Pmv19         catttgacttctatgtttgccccgggcatactgtaccaacagggtgtggagggccgagagagggctactgtggcaaatgg
Pmv2          catttgacttctatgtttgccccgggcatactgtaccaacagggtgtggagggccgagagagggctactgtggcaaatgg
Pmv20         catttgacttctatgtttgccccgggcatactgtaccaacagggtgtggagggccgagagagggctactgtggcaaatgg
Pmv21         catttgacttctatgtttgccccgggcatactgtaccaacagggtgtAgagggccgagagagggctactgtggcaaatgg
Pmv22         catttgacttctatgtttgccccgggcatactgtaccaacagggtgtggagggccgagagagggctactgtggcaaatgg
Pmv23         catttgacttctatgtttgccccgggcatactgtaccaacagggtgtggagggccgagagagggctactgtggcaaatgg
Pmv24         catttgacttctatgtttgccccgggcatactgtaccaacagggtgtggagggccgagagagggctactgtggcaaatgg
Pmv4          catttgacttctatgtttgccccgggcatactgtaccaacagggtgtAgagggccgaAaAagggctactgtggcaaatgg
Pmv5          catttgacttctatgtttgccccgggcatactgtaccaacagggtgtggagggccgagagagggctactgtggcaaatgg
Pmv6          catttgacttctatgtttgccccgggcatactgtaccaacagggtgtggagggccgagagagggctactgtggcaaatgg
Pmv7          catttgacttctatgtttgccccgggcatactgtaccaacagggtgtggagggccgagagagggctactgtggcaaatgg
Pmv8          catttgacttctatgtttgccccgggcatactgtaccaacagggtgtggagggccgagagagggctactgtggcaaatgg
Pmv9          catttgacttctatgtttgccccgggcatactgtaccaacagggtgtggagggccgagagagggctactgtggcaaatgg

PMV_CONS      GGCTGTGAGACCACTGGACAGGCATACTGGAAGCCATCATCATCATGGGACCTAATTTCCCTTAAGCGAGGAAACACCCC
Pmv1          ggctgtgagaccactggacaggcatactggaagccatcatcatcatgggacctaatttcccttaagcgaggaaacacccc
Pmv10         ggctgtgagaccactggacaggcatactggaagccatcatcatcatgggacctaatttcccttaagcgaAgaaacacccc
Pmv11         ggctgtgagaccactggacaggcatactggaagccatcatcatcatgggacctaatttcccttaagcgaggaaacacccc
Pmv12         ggctgtgagaccactggacaggcatactggaagccatcatcatcatgggacctaatttcccttaagcgaggaaacacccc
Pmv13         ggctgtgagaccactggacaggcatactggaagccatcatcatcatgggacctaatttcccttaagcgaAgaaacacccc
Pmv14         ggctgtgagaccactggacaggcatactggaagccatcatcatcatgggacctaatttcccttaagcgaggaaacacccc
Pmv15         ggctgtgagaccactggacaggcatactggaagccatcatcatcatgggacctaatttcccttaagcgaggaaacacccc
Pmv16         ggctgtgagaccactggacaggcatactggaagccatcatcatcatgggacctaatttcccttaagcgaggaaacacccc
Pmv17         ggctgtgagaccactggacaggcatactggaagccatcatcatcatgggacctaatttcccttaagcgagAaaacacccc
Pmv18         --------------------------------------------------------------------------------
Pmv19         ggctgtgagaccactggacaggcatactggaagccatcatcatcatgggacctaatttcccttaagcgaggaaacacccc
Pmv2          ggctgtgagaccactggacaggcatactggaagccatcatcatcatgggacctaatttcccttaagcgaggaaacacccc
Pmv20         ggctgtgagaccactggacaggcatactggaagccatcatcatcatgggacctaatttcccttaagcgaggaaacacccc
Pmv21         ggctgtgagaccactggacaggcatactggaagccatcatcatcatgggacctaatttcccttaagcgaggaaacacccc
Pmv22         ggctgtgagaccactggacaggcatactggaagccatcatcatcatgggacctaatttcccttaagcgaggaaacacccc
Pmv23         ggctgtgagaccactggacaggcatactggaagccatcatcatcatgggacctaatttcccttaagcgaAgaaacacccc
Pmv24         ggctgtgagaccactggacaggcatactggaagccatcatcatcatgggacctaatttcccttaagcgaggaaacacccc
Pmv4          ggctgtgagaccactggacaggcatactgAaagccatcatcatcatgggacctaatttcccttaagcgaAgaaacacccc
Pmv5          ggctgtgagaccactggacaggcatactggaagccatcatcatcatgggacctaatttcccttaagcgaggaaacacccc
Pmv6          ggctgtgagaccactggacaggcatactggaagccatcatcatcatgggacctaatttcccttaagcgaggaaacacccc
Pmv7          ggctgtgagaccactggacaggcatactggaagccatcatcatcatgggacctaatttcccttaagcgaggaaacacccc
Pmv8          ggctgtgagaccactggacaggcatactggaagccatcatcatcatgggacctaatttcccttaagcgaggaaacacccc
Pmv9          ggctgtgagaccactggacaggcatactggaagccatcatcatcatgggacctaatttcccttaagcgaggaaacacccc
```

```
PMV_CONS    TCGGAATCAGGGCCCCTGTTATGATTCCTCAGCGGTCTCCAGTGACATCAAGGGCGCCACACCGGGGGGTCGATGCAATC
Pmv1        tcggaatcagggcccctgttatgattcctcagcggtctccagtgacatcaagggcgccacaccggggggtcgatgcaatc
Pmv10       tcAgaatcagggcccctgttatgattcctcagcggtctccagtgacatcaagggcgccacaccggggggtcgatgcaatc
Pmv11       tcggaatcagggcccctgttatgattcctcagcggtctccagtgacatcaagggcgccacaccggggggtcgatgcaatc
Pmv12       tcggaatcagggcccctgttatgattcctcagcggtctccagtgacatcaagggcgccacaccggggggtcgatgcaatc
Pmv13       tcAgaatcagggcccctgttatgattcctcagcggtctccagtgacatcaagggcgccacaccggggggtcgatgcaatc
Pmv14       tcggaatcagggcccctgttatgattcctcagcggtctccagtgacatcaagggcgccacaccggggggtcgatgcaatc
Pmv15       tcggaatcagggcccctgttatgattcctcagcggtctccagtgacatcaagggcgccacaccggggggtcgatgcaatc
Pmv16       tcggaatcagggcccctgttatgattcctcagcggtctccagtgacatcaagggcgccacaccggggggtcgatgcaatc
Pmv17       tcggaatcagggcccctgttatgattcctcagcggtctccagtgacatcaagggcgccacaccggggggtcgatgcaatc
Pmv18       --------------------------------------------------------------------------------
Pmv19       tcggaatcagggcccctgttatgattcctcagcggtctccagtgacatcaagggcgccacaccggggggtcgatgcaatc
Pmv2        tcAgaatcagggcccctgttatgattcctcagcggtctccagtgacatcaagggcgccacaccggggggtcgatgcaatc
Pmv20       tcggaatcagggcccctgttatgattcctcagcggtctccagtgacatcaagggcgccacaccggggggtcgatgcaatc
Pmv21       tcggaatcagggcccctgttatgattcctcagcggtctccagtgacatcaagggcgccacaccggggggtcgatgcaatc
Pmv22       tcggaatcagggcccctgttatgattcctcagcggtctccagtgacatcaagggcgccacaccggggggtcgatgcaatc
Pmv23       tcAgaatcagggcccctgttatgattcctcagcggtctccagtgacatcaagggcgccacaccggggggtcgatgcaatc
Pmv24       tcggaatcagggcccctgttatgattcctcagcggtctccagtgacatcaagggcgccacaccggggggtcgatgcaatc
Pmv4        tcAgaatcagggcccctgttatgattcctcagcggtctccagtgacatcaagggcgccacaccggggggtcgatgcaatc
Pmv5        tcggaatcagggcccctgttatgattcctcagcggtctccagtgacatcaagggcgccacaccggggggtcgatgcaatc
Pmv6        tcggaatcagggcccctgttatgattcctcagcggtctccagtgacatcaagggcgccacaccggggggtcgatgcaatc
Pmv7        tcggaatcagggcccctgttatgattcctcagcggtctccagtgacatcaagggcgccacaccggggggtcgatgcaatc
Pmv8        tcggaatcagggcccctgttatgattcctcagcggtctccagtgacatcaagggcgccacaccggggggtcgGtgcaatc
Pmv9        tcggaatcagggcccctgttatgattcctcagcggtctccagtgacatcaagggcgccacaccggggggtcgatgcaatc

PMV_CONS    CCCTAGTCCTGGAATTCACTGACGCGGGCAAAAAGGCCAGCTGGGATGGCCCCAAAGTATGGGGACTAAGACTGTACCGA
Pmv1        ccctagtcctggaattcactgacgcgggcaaaaaggccagctgggatggccccaaagtatggggactaagactgtaccga
Pmv10       ccctagtcctggaattcactgacgcgggcaaaaaggccagctgggatggccccaaagtatggggactaagactgtaccga
Pmv11       ccctagtcctggaattcactgacgcgggcaaaaaggccagctgggatggccccaaagtatggggactaagactgtaccga
Pmv12       ccctagtcctggaattcactgacgcgggcaaaaaggccagctgggatggccccaaagtatggggactaagactgtaccga
Pmv13       ccctagtcctggaattcactgacgcgggcaaaaaggccagctgggatggccccaaagtatggggactaagactgtaccga
Pmv14       ccctagtcctggaattcactgacgcgggcaaaaaggccagctgggatggccccaaagtatggggactaagactgtaccga
Pmv15       ccctagtcctggaattcactgacgcgggcaaaaaggccagctgggatggccccaaagtatggggactaagactgtaccga
Pmv16       ccctagtcctggaattcactgacgcgggcaaaaaggccagctgggatggccccaaagtatggggactaagactgtaccga
Pmv17       ccctagtcctggaattcactgacgcgggcaaaaaggccagctgggatggccccaaagtatggggactaagactgtaccga
Pmv18       --------------------------------aaggccagctgggatggccccaaagtatggggactaagactgtaccga
Pmv19       ccctagtcctggaattcactgacgcgggcaaaaaggccagctgggatggccccaaagtatggggactaagactgtaccga
Pmv2        ccctagtcctggaattcactgacgcgggcaaaaaggccagctgggatggccccaaagtatggggactaagactgtaccga
Pmv20       ccctagtcctggaattcactgacgcgggcaaaaaggccagctgggatggccccaaagtatggggactaagactgtaccga
Pmv21       ccctagtcctgAaattcactgacgcgggcaaaaaggccagctgAgatggccccaaagtatggggactaagactgtaccga
Pmv22       ccctagtcctggaattcactgacgcgggcaaaaaggccagctgggatggccccaaagtatggggactaagactgtaccga
Pmv23       ccctagtcctggaattcactgacgcgggcaaaaaggccagctgggatggccccaaagtatggggactaagactgtaccga
Pmv24       ccctagtcctggaattcactgacAcgggcaaaaaggccagctgggatggccccaaagtatggggactaagactgtaccga
Pmv4        ccctagtcctAAaattcactgacgcgggcaaaaaggccagctgggatggccccaaagtatAgAgactaagactgtaccga
Pmv5        ccctagtcctggaattcactgacgcgggcaaaaaggccagctgggatggccccaaagtatggggactaagactgtaccga
Pmv6        ccctagtcctggaattcactAacgcgggcaaaaaggccagctgggatggccccaaagtatggggactaagactgtaccga
Pmv7        ccctagtcctggaattcactgacgcgggcaaaaaggccagctgggatggccccaaagtatggggactaagactgtaccga
Pmv8        ccctagtcctggaattcactgacgcgggcaaaaaggccagctgggatggccccaaagtatggggactaagactgtaccga
Pmv9        ccctagtcctggaattcactgacgcgggcaaaaaggccagctgggatggccccaaagtatggggactaagactgtaccga
```

```
PMV_CONS      TCCACAGGGACCGACCCGGTGACCCGGTTCTCTTTGACCCGCCAGGTCCTCAATATAGGGCCCCGCGTCCCCATTGGGCC
Pmv1          tccacagggaccgacccggtgacccggttctctttgacccgccaggtcctcaatatagggccccgcgtccccattgggcc
Pmv10         tccacagggaccgacccggtgacccggttctctttgacccgccaggtcctcaatatagggccccgcgtccccattgggcc
Pmv11         tccacagggaccgacccggtgacccggttctctttgacccgccaggtcctcaatatagggccccgcgtccccattgggcc
Pmv12         tccacagggaccgacccggtgacccggttctctttgacccgccaggtcctcaatatagggccccgcgtccccattgggcc
Pmv13         tccacagggaccgacccggtgacccggttctctttgacccgccaggtcctcaatatagggccccgcgtccccattgggcc
Pmv14         tccacagggaccgacccggtgacccggttctctttgacccgccaggtcctcaatatagggccccgcgtccccattgggcc
Pmv15         tccacagggaccgacccggtgacccggttctctttgacccgccaggtcctcaatatagggccccgcgtccccattgggcc
Pmv16         tccacagggaccgacccggtgacccggttctctttgacccgccaggtcctcaatatagggccccgcgtccccattgggcc
Pmv17         tccacagggaccgacccggtgacccggttctctttgacccgccaggtcctcaatatagggccccgcgtccccattgggcc
Pmv18         tccacagggaccgacccggtgacccggttctctttgacccgccaggtcctcaatatagggccccgcgtccccattgggcc
Pmv19         tccacagggaccgacccggtgacccggttctctttgacccgccaggtcctcaatatagggccccgcgtccccattgggcc
Pmv2          tccacagggaccgacccggtgacccggttctctttgacccgccaggtcctcaatatagggccccgcgtccccattgggcc
Pmv20         tccacagggaccgacccggtgacccggttctctttgacccgccaggtcctcaatatagggccccgcgtccccattgggcc
Pmv21         tccacagggaccgacccggtgacccggttctctttgacccgccaggtcctcaatatagggccccgcgtccccattgggcc
Pmv22         tccacagggaccgacccggtgacccggttctctttgacccgccaggtcctcaatatagggccccgcgtccccattgggcc
Pmv23         tccacagggaccgacccggtgacccggttctctttgacccgccaggtcctcaatatagggccccgcgtccccattgggcc
Pmv24         tccacagggaccgacccggtgacccggttctctttgacccgccaggtcctcaatatagggccccgcgtccccattgggcc
Pmv4          tccacagggaccgacccggtgacccggttctctttgacccgccaggtcctcaatatagggccccgcgtccccattgggcc
Pmv5          tccacagggaccgacccggtgacccggttctctttgacccgccaggtcctcaatatagggccccgcgtccccattgggcc
Pmv6          tccacagggaccgacccggtgacccggttctctttgacccgccaggtcctcaatatagggccccgcgtccccattgggcc
Pmv7          tccacagggaccgacccggtgaccTggttctctttgacccgccaggtcctcaatatagggccccgcgtccccattgggcc
Pmv8          tccacagggaccgacccggtgacccggttctctttgacccgccaggtcctcaatatagggccccgcgtccccattgggcc
Pmv9          tccacagggaccgacccggtgacccggttctctttgacccgccaggtcctcaatatagggccccgcgtccccattgggcc

PMV_CONS      TAATCCCGTGATCACTGACCAGTTACCCCCCTCCCGACCCGTGCAGATCATGCTCCCCAGGCCTCCTCAGCCTCCTCCTC
Pmv1          taatcccgtgatcactgaccagttacccccctcccgacccgtgcagatcatgctccccaggcctcctcagcctcctcctc
Pmv10         taatcccgtgatcactgaccagttacccccctcccgacccgtgcagatcatgctccccaggcctcctcagcctcctcctc
Pmv11         taatcccgtgatcactgaccagttacccccctcccgacccgtgcagatcatgctccccaggcctcctcagcctcctcctc
Pmv12         taatcccgtgatcactgaccagttacccccctcccgacccgtgcagatcatgctccccaggcctcctcagcctcctcctc
Pmv13         taatcccgtgatcactgaccagttacccccctcccgacccgtgcagatcatgctccccaggcctcctcagcctcctcctc
Pmv14         taatcccgtgatcactgaccagttacccccctcccgacccgtgcagatcatgctccccaggcctcctcagcctcctcctc
Pmv15         taatcccgtgatcactgaccagttacccccctcccgacccgtgcagatcatgctccccaggcctcctcagcctcctcctc
Pmv16         taatcccgtgatcactgaccagttacccccctcccgacccgtgcagatcatgctccccaggcctcctcagcctcctcctc
Pmv17         taatcccgtgatcactgaccagttacccccctcccgacccgtgcagatcatgctccccaggcctcctcagcctcctcctc
Pmv18         taatcccgtgatcactgaccagttacccccctcccgacccgtgcagatcatgctccccaggcctcctcagcctcctcctc
Pmv19         taatcccgtgatcactgaccagttacccccctcccgacccgtgcagatcatgctccccaggcctcctcagcctcctcctc
Pmv2          taatcccgtgatcactgaccagttacccccctcccgacccgtgcagatcatgctccccaggcctcctcagcctcctcctc
Pmv20         taatcccgtgatcactgaccagttacccccctcccgacccgtgcagatcatgctccccaggcctcctcagcctcctcctc
Pmv21         taatcccgtgatcactgaccagttacccccctcccgacccgtgcagatcatgctccccaggcctcctcagcctcctcctc
Pmv22         taatcccgtgatcactgaccagttacccccctcccgacccgtgcagatcatgctccccaggcctcctcagcctcctcctc
Pmv23         taatcccgtgatcactgaccagttacccccctcccgacccgtgcagatcatgctccccaggcctcctcagcctcctcctc
Pmv24         taatcccgtgatcactgaccagttacccccctcccgacccgtgcagatcatgctccccaggcctcctcagcctcctcctc
Pmv4          taatcccgtgatcactgaccagttacccccctcccgacccgtgcagatcatgctccccaggcctcctcagcctcctcctc
Pmv5          taatcccgtgatcactgaccagttacccccctcccgacccgtgcagatcatgctccccaggcctcctcagcctcctcctc
Pmv6          taatcccgtgatcactgaccagttacccccctcccgacccgtgcagatcatgctccccaggcctcctcagcctcctcctc
Pmv7          taatcccgtgatcactgaccagttacccccctcccgacccgtgcagatcatgctccccaggcctcctcagcctcctcctc
Pmv8          taatcccgtgatcactgaccagttacccccctcccgacccgtgcagatcatgctccccaggcctcctcagcctcctcctc
Pmv9          taatcccgtgatcactgaccagttacccccctcccgacccgtgcagatcatgctccccaggcctcctcagcctcctcctc
```

```
PMV_CONS    CAGGCGCAGCCTCTATAGTCCCTGAGACTGCCCCACCTTCTCAACAACCTGGGACGGGAGACAGGCTGCTAAACCTGGTA
Pmv1        caggcgcagcctctatagtccctgagactgccccaccttctcaacaacctgggacgggagacaggctgctaaacctggta
Pmv10       caggcgcCgcctctatagtccctgagactgccccaccttctcaacaacctgggacgggagacaggctgctaaacctggta
Pmv11       caggcgcagcctctatagtccctgagactgccccaccttctcaacaacctgggacgggagacaggctgctaaacctggta
Pmv12       caggcgcagcctctatagtccctgagactgccccaccttctcaacaacctgggacgggagacaggctgctaaacctggta
Pmv13       caggcgcagcctctaCagtccctgagactgccccaccttctcaacaacctgggacgggagacaggctgctaaacctggta
Pmv14       caggcgcagcctctatagtccctgagactgccccaccttctcaacaacctgggacgggagacaggctgctaaacctggta
Pmv15       caggcgcagcctctatagtccctgagactgccccaccttctcaacaacctgggacgggagacaggctgctaaacctggta
Pmv16       caggcgcagcctctatagtccctgagactgccccaccttctcaacaacctgggacgggagacaggctgctaaacctggta
Pmv17       caggcgcagcctctatagtccctgagactgccccaccttctcaacaacctgggacgggagacaggctgctaaacctggta
Pmv18       caggcgcagcctctatagtccctgagactgccccaccttctcaacaacctgggacgggagacaggctgctaaacctggta
Pmv19       caggcgcagcctctatagtccctgagactgccccaccttctcaacaacctgggacgggagacaggctgctaaacctggta
Pmv2        caggcgcagcctctatagtccctgagactgccccaccttctcaacaacctgggacgggagacaggctgctaaacctggta
Pmv20       caggcgcagcctctatagtccctgagactgccccaccttctcaacaacctgggacgggagacaggctgctaaacctggta
Pmv21       caggcgcagcctctatagtccctgagactgccccaccttctcaacaacctgggacgggagacaggctgctaaacctggta
Pmv22       caggcgcagcctctatagtccctgagactgccccaccttctcaacaacctgggacgggagacaggctgctaaacctggta
Pmv23       caggcgcagcctctatagtccctgagactgccccaccttctcaacaacctgggacgggagacaggctgctaaacctggta
Pmv24       caggcgcagcctctatagtccctgagactgccccaccttctcaacaacctgggacgggagacaggctgctaaacctggta
Pmv4        caggcgcagcctctatagtccctgagactgccccaccttctcaacaacctgggacgAgagacaggctgctaaacctggta
Pmv5        caggcgcagcctctatagtccctgagactgccccaccttctcaacaacctgggacgggagacaggctgctaaacctggta
Pmv6        caggcgcagcctctatagtccctgagactgccccaccttctcaacaacctgggacgggagacaggctgctaaacctggta
Pmv7        caggcgcagcctctatagtccctgagactgccccaccttctcaacaacctgggacgggagacaggctgctaaacctggta
Pmv8        caggcgcagcctctatagtccctgagactgccccaccttctcaacaacctgggacgggagacaggctgctaaacctggta
Pmv9        caggcgcagcctctatagtccctgagactgccccaccttctcaacaacctgggacgggagacaggctgctaaacctggta

PMV_CONS    GATGGAGCCTACCAAGCTCTCAACCTCACCAGTCCTGACAAAACCCAAGAGTGCTGGTTGTGTCTGGTAGCGGGACCCCC
Pmv1        gatggagcctaccaagctctcaacctcaccagtcctgacaaaacccaagagtgctggttgtgtctggtagcgggaccccc
Pmv10       gatggagcctaccaagctctcaacctcaccagtcctgacaaaacccaagagtgctggttgtgtctggtagcgggaccccc
Pmv11       gatggagcctaccaagctctcaacctcaccagtcctgacaaaacccaagagtgctggttgtgtctggtagcgggaccccc
Pmv12       gatggagcctaccaagctctcaacctcaccagtcctgacaaaacccaagagtgctggttgtgtctggtagcgggaccccc
Pmv13       gatggagcctaccaagctctcaacctcaccagtcctgacaaaacccaagagtgctggttgtgtctggtagcgggaccccc
Pmv14       gatggagcctaccaagctctcaacctcaccagtcctgacaaaacccaagagtgctggttgtgtctggtagcgggaccccc
Pmv15       gatggagcctaccaagctctcaacctcaccagtcctgacaaaacccaagagtgctggttgtgtctggtagcgggaccccc
Pmv16       gatggagcctaccaagctctcaacctcaccagtcctgacaaaacccaagagtgctggttgtgtctggtagcgggaccccc
Pmv17       gatggagcctaccaagctctcaacctcaccagtcctgacaaaacccaagagtgctggttgtgtctggtagcgggaccccc
Pmv18       gatggagcctaccaagctctcaacctcaccagtcctgacaaaacccaagagtgctggttgtgtctggtagcgggaccccc
Pmv19       gatggagcctaccaagctctcaacctcaccagtcctgacaaaacccaagagtgctggttgtgtctggtagcgggaccccc
Pmv2        gatggagcctaccaagctctcaacctcaccagtccCgacaaaacccaagagtgctggttgtgtctggtagcgggaccccc
Pmv20       gatggagcctaccaagctctcaacctcaccagtccCgacaaaacccaagagtgctggttgtgtctggtagcgggaccccc
Pmv21       AatAgagcctaccaagctctcaacctcaccagtcctgacaaaacccaagagtgctggttgtgtctggtagcgggaccccc
Pmv22       gatggagcctaccaagctctcaacctcaccagtcctgacaaaacccaagagtgctggttgtgtctggtagcgggaccccc
Pmv23       gatggagcctaccaagctctcaacctcaccagtcctgacaaaacccaagagtgctggttgtgtctggtagcgggaccccc
Pmv24       gatggagcctaccaagctctcaacctcaccagtccCgacaaaacccaagagtgctggttgtgtctggtagcgggaccccc
Pmv4        AatAgagcctaccaagctctcaacctcaccagtcctgacaaaacccaagagtgctggttgtgtctggtagcgggaccccc
Pmv5        gatggagcctaccaagctctcaacctcaccagtcctgacaaaacccaagagtgctggttgtgtctggtagcgggaccccc
Pmv6        gatggagcctaccaagctctcaacctcaccagtcctgacaaaacccaagagtgctggttgtgtctggtagcgggaccccc
Pmv7        gatggagcctaccaagctctcaacctcaccagtccCgacaaaacccaagagtgctggttgtgtctggtagcgggaccccc
Pmv8        gatggagcctaccaagctctcaacctcaccagtcctgacaaaacccaagagtgctggttgtgtctggtagcgggaccccc
Pmv9        gatggagcctaccaagctctcaacctcaccagtcctgacaaaacccaagagtgctggttgtgtctggtagcgggaccccc
```

```
PMV_CONS    CTACTACGAAGGGGTTGCCGTCCTAGGTACTTATTCCAACCATACCTCTGCCCCAGCTAACTGCTCCGTGGCCTCCCAAC
Pmv1        ctactacgaaggggttgccgtcctaggtacttattccaaccatacctctgccccagctaactgctccgtggcctcccaac
Pmv10       ctactacgaaggggttgccgtcctaggtacttattccaaccatacctctgccccagctaactgctccgtggcctcccaac
Pmv11       ctactacgaaggggttgccgtcctaggtacttattccaaccatacctctgccccagctaactgctccgtggcctcccaac
Pmv12       ctactacgaaggggttgccgtcctaggtacttattccaaccatacctctgccccagctaactgctccgtggcctcccaac
Pmv13       ctactacgaaggggttgccgtcctaggtacttattccaaccatacctctgccccagctaactgctccgtggcctcccaac
Pmv14       ctactacgaaggggttgccgtcctaggtacttattccaaccatacctctgccccagctaactgTtccgtggcctcccaac
Pmv15       ctactacgaaggggttgccgtcctaggtacttattccaaccatacctctgccccagctaactgctccgtggcctcccaac
Pmv16       ctactacgaaggggttgccgtcctaggtacttattccaaccatacctctgccccagctaactgctccgtggcctcccaac
Pmv17       ctactacgaaggggttgccgtcctaggtacttattccaaccatacctctgccccagctaactgctccgtggcctcccaac
Pmv18       ctactacgaaggggttgccgtcctaggtacttattccaaccatacctctgccccagctaactgctccgtggcctcccaac
Pmv19       ctactacgaaggggttgccgtcctaggtacttattccaaccatacctctgccccagctaactgctccgtggcctcccaac
Pmv2        ctactacgaaggggttgccgtcctaggtacttattccaaccatacctctgccccagctaactgctccgtggcctcccaac
Pmv20       ctactacgaaggggttgccgtcctaggtacttattccaaccatacctctgccccagctaactgctccgtggcctcccaac
Pmv21       ctactacgaaggggttgccgtcctaggtacttattccaaccatacctctgccccagctaactgctccgtggcctcccaac
Pmv22       ctactacgaaggggttgccgtcctaggtacttattccaaccatacctctgccccagctaactgctccgtggcctcccaac
Pmv23       ctactacgaaggggttgccgtcctaggtacttattccaaccatacctctgccccagctaactgctccgtggcctcccaac
Pmv24       ctactacgaaggggttgccgtcctaggtacttattccaaccatacctctgccccagctaactgctccgtggcctcccaac
Pmv4        ctactacgaaggggttgccgtcctaggtacttattccaaccatacctctgccccagctaactgctccgtggcctcccaac
Pmv5        ctactacgaaggggttgccgtcctaggtacttattccaacTatacctctgccccagctaactgctccgtggcctcccaac
Pmv6        ctactacgaaggggttgccgtcctaggtacttattccaaccatacctctgccccagctaactgctccgtggcctcccaac
Pmv7        ctactacgaaggggttgccgtcctaggtacttattccaaccatacctctgccccagctaactgctccgtggcctcccaac
Pmv8        ctactacgaaggggttgccgtcctaggtacttattccaaccatacctctgccccagctaactgctccgtggcctcccaac
Pmv9        ctactacgaaggggttgccgtcctaggtacttattccaaccatacctctgccccagctaactgctccgtggcctcccaac

PMV_CONS    ACAAGCTGACCCTGTCCGAAGTGACCGGACAGGGACTCTGCGTAGGAGCAGTTCCCAAAACCCATCAGGCCCTGTGTAAT
Pmv1        acaagctgaccctgtccgaagtgaccggacagggactctgcgtaggagcagttcccaaaacccatcaggccctgtgtaat
Pmv10       acaagctgaccctgtccgaagtgaccggacagggactctgcgtaAgagcagttcccaaaacccatcaggccctgtgtaat
Pmv11       acaagctgaccctgtccgaagtgaccggacagggactctgcgtaggagcagttcccaaaacccatcaggccctgtgtaat
Pmv12       acaagctgaccctgtccgaagtgaccggacagggactctgcgtaggagcagttcccaaaacccatcaggccctgtgtaat
Pmv13       acaagctgaccctgtccgaagtgaccggacagggactctgcgtaggagcagttcccaaaacccatcaggccctgtgtaat
Pmv14       acaagctgaccctgtccAaagtgaccggacagggactctgcgtaggagcagttcccaaaacccatcaggccctgtgtaat
Pmv15       acaagctgaccctgtccgaagtgaccggacagggactctgcgtaggagcagttcccaaaacccatcaggccctgtgtaat
Pmv16       acaagctgaccctgtccgaagtgaccggacagggactctgcgtaggagcagttcccaaaacccatcaggccctgtgtaat
Pmv17       acaagctgaccctgtccgaagtgaccggacagggactctgcgtaggagcagttcccaaaacccatcaggccctgtgtaat
Pmv18       acaagctgaccctgtccgaagtgaccggacagggactctgcgtaAgagcagttcccaaaacccatcaggccctgtgtaat
Pmv19       acaagctgaccctgtccgaagtgaccggacagggactctgcgtaggagcagttcccaaaacccatcaggccctgtgtaat
Pmv2        acaagctgaccctgtccgaagtgaccggacagggactctgcgtaggagcagttcccaaaacccatcaggccctgtgtaat
Pmv20       acaagctgaccctgtccgaagtgaccggacagggactctgcgtaggagcagttcccaaaacccatcaggccctgtgtaat
Pmv21       acaagctgaccctgtccgaagtgaccggacagggactctgcgtaggagcagttcccaaaacccatcaggccctgtgtaat
Pmv22       acaagctgaccctgtccgaagtgaccggacagggactctgcgtaggagcagttcccaaaacccatcaggccctgtgtaat
Pmv23       acaagctgaccctgtccgaagtgaccggacagggactctgcgtaggagcagttcccaaaacccatcaggccctgtgtaat
Pmv24       acaagctgaccctgtccgaagtgaccggacagggactctgcgtaggagcagttcccaaaacccatcaggccctgtgtaat
Pmv4        acaagctgaccctgtccAaagtgaccggacagggactctgcgtaAgagcagttcccaaaacccatcaggccctgtgtaat
Pmv5        acaagctgaccctgtccgaagtgaccggacagggactctgcgtaggagcagttcccaaaacccatcaggccctgtgtaat
Pmv6        acaagctgaccctgtccgaagtgaccggacagggactctgcgtaggagcagttcccaaaacccatcaggccctgtgtaat
Pmv7        acaagctgaccctgtccgaagtgaccggacagggactctgcgtaggagcagttcccaaaacccatcaggccctgtgtaat
Pmv8        acaagctgaccctgtccAaagtgaccggacagggactctgcgtaggagcagttcccaaaacccatcaggccctgtgtaat
Pmv9        acaagctgaccctgtccgaagtgaccggacagggactctgcgtaggagcagttcccaaaacccatcaggccctgtgtaat
```

```
PMV_CONS    ACCACCCAGAAGACGAGCGACGGGTCCTACTATCTGGCTGCTCCCGCCGGGACCATTTGGGCTTGCAACACCGGGCTCAC
Pmv1        accacccagaagacgagcgacgggtcctactatctggctgctcccgccgggaccatttgggcttgcaacaccgggctcac
Pmv10       accacccagaagacgagcgacgggtcctactatctggctgctcccgccgggaccatttgggcttgcaacaccgggctcac
Pmv11       accacccagaagacgagcgacgggtcctactatctggctgctcccgccgggaccatttgggcttgcaacaccgggctcac
Pmv12       accacccagaagacgagcgacgggtcctactatctggctgctcccgccgggaccatttgggcttgcaacaccgggctcac
Pmv13       accacccagaagacgagcgacgggtcctactatctggctgctcccgccgggaccatttgggcttgcaacaccgggctcac
Pmv14       accacccagaagacgagcgacgggtcctactatctggctgctcccgccgggaccatttgggcttgcaacaccgggctcac
Pmv15       accacccagaagacgagcAacgggtcctactatctggctgctcccgccgggaccatttgggcttgcaacaccgggctcac
Pmv16       accacccagaagacgagcgacgggtcctactatctggctgctcccgccgggaccatttgggcttgcaacaccgggctcac
Pmv17       accacccagaagGcgagcgacgggtcctactatctggctgctcccgccgggaccatttgggcttgcaacaccgggctcac
Pmv18       accacccagaagacgagcgacgggtcctactatctggctgctcccgccgggaccatttgggcCtgcaacaccgggctcac
Pmv19       accacccagaagacgagcgacgggtcctactatctggctgctcccgccgggaccatttgggcttgcaacaccgggctcac
Pmv2        accacccagaagacgagcgacgggtcctactatctggctgctcccgccgggaccatttgggcttgcaacaccgggctcac
Pmv20       accacccAaagacgagcgacgggtcctactatctggctgctcccgccgggaccatttgggcttgcaacaccgggctcac
Pmv21       accacccagaagacgagcgacgggtcctactatctggctgctcccgccgggaccatttgggcttgcaacaccgggctcac
Pmv22       accacccagaagacgagcgacgggtcctactatctggctgctcccgccgggaccatttgggcttgcaacaccgggctcac
Pmv23       accacccagaagacgagcgacgggtcctactatctggctgctcccgccgggaccatttgggcttgcaacaccgggctcac
Pmv24       accacccagaagacgagcgacgggtcctactatctggctgctcccgccgggaccatttgggcttgcaacaccgggctcac
Pmv4        accacccAaagacgagcgacgggtcctactatctggctgctcccgccgAgaccatttAggcttgcaacaccgggctcac
Pmv5        accacccagaagacgagcgacgggtcctactatctggctgctcccgccgggaccatttgggcttgcaacaccgggctcac
Pmv6        accacccagaagacgagcgacgggtcctactatctggctgctcccgccgggaccatttgggcCtgcaacaccgggctcac
Pmv7        accacccagaagacgagcgacgggtcctactatctggctgctcccgccgggaccatttgggcttgcaacaccgggctcac
Pmv8        accacccagaagacgagcgacgggtcctactatctggctgctcccgccgggaccatttgggcttgcaacaccgggctcac
Pmv9        accacccagaagacgagcgacgggtcctactatctggctgctcccgccgggaccatttgggcttgcaacaccgggctcac

PMV_CONS    TCCCTGCCTATCTACCACTGTACTCGACCTCACCACCGATTACTGTGTCCTGGTTGAGCTCTGGCCAAAGGTGACCTACC
Pmv1        tccctgcctatctaccactgtactcgacctcaccaccgattactgtgtcctggttgagctctggccaaaggtgacctacc
Pmv10       tccctgcctatctaccactgtGctcgacctcaccaccgattactgtgtcctggttgagctctggccaaaggtgacctacc
Pmv11       tccctgcctatctaccactgtactcgacctcaccaccgattactgtgtcctggttgagctctggccaaaggtgacctacc
Pmv12       tccctgcctatctaccactgtactcgacctcaccaccgattactgtgtcctggttgagctctggccaaaggtgacctacc
Pmv13       tccctgcctatctaccactgtGctcgacctcaccaccgattactgtgtcctggttgagctctggccaaaggtgacctacc
Pmv14       tccctgcctatctaccactgtactcgacctcaccaccgattactgtgtcctggttgagctctggccaaaggtgacctacc
Pmv15       tccctgcctatctaccactgtactcgacctcaccaccgattactgtgtcctggttgagctctggccaaaggtgacctacc
Pmv16       tccctgcctatctaccactgtactcgacctcaccaccgattactgtgtcctggttgagctctggccaaaggtgacctacc
Pmv17       tccctgcctatctaccactgtactcgacctcaccaccgattactgtgtcctggttgagctctggccaaaggtgacctacc
Pmv18       tccctgcctatctaccactgtactcgacctcaccaccgattactgtgtcctggttgagctctggccaaaggtgacctacc
Pmv19       tccctgcctatctaccactgtactcgacctcaccaccgattactgtgtcctggttgagctctggccaaaggtgacctacc
Pmv2        tccctgcctatctaccactgtactcgacctcaccaccgattactgtgtcctggttgagctctggccaaaggtgacctacc
Pmv20       tccctgcctatctaccactgtactcgacctcaccaccgattactgtgtcctggttgagctctggccaaaggtgacctacc
Pmv21       tccctgcctatctaccactgtactcgacctcaccaccgattactgtgtcctggttgagctctggccaaaggtgacctacc
Pmv22       tccctgcctatctaccactgtactcgacctcaccaccgattactgtgtcctggttgagctctggccaaaggtgacctacc
Pmv23       tccctgcctatctaccactgtGctcgacctcaccaccgattactgtgtcctggttgagctctggccaaaggtgacctacc
Pmv24       tccctgcctatctaccactgtactcgacctcaccaccgattactgtgtcctggttgagctctggccaaaggtgacctacc
Pmv4        tccctgcctatctaccactgtactcgacctcaccaccgattactgtgtcctggttgagctctggccaaaggtgacctacc
Pmv5        tccctgcctatctaccactgtactcgacctcaccaccgattactgtgtcctggttgagctctggccaaaggtgacctacc
Pmv6        tccctgcctatctaccactgtactcgacctcaccaccgattactgtgtcctggttgagctctggccaaaggtgacctacc
Pmv7        tccctgcctatctaccactgtactcgacctcaccaccgattactgtgtcctggttgagctctggccaaaggtgacctacc
Pmv8        tccctgcctatctaccactgtactcgacctcaccaccgattactgtgtcctggttgagctctggccaaaggtgacctacc
Pmv9        tccctgcctatctaccactgtactcgacctcaccaccgattactgtgtcctggttgagctctggccaaaggtgacctacc
```

```
PMV_CONS      ACTCCCCTGGTTATGTTTATGGCCAGTTTGAGAGAAAAACCAAATATAAAAGAGAGCCGGTGTCATTAACTCTGGCCCTG
Pmv1          actcccctggttatgtttatggccagtttgagaAaaaaaccaaatataaaagagagccggtgtcattaactctggccctg
Pmv10         actcccctggttatgtttatggccagtttgagagaaaaaccaaatataaaagagagccggtgtcattaactctggccctg
Pmv11         actcccctggttatgtttatggccagtttgagaAaaaaaccaaatataaaagagagccggtgtcattaactctggccctg
Pmv12         actcccctggttatgtttatggccagtttgagaAaaaaaccaaatataaaagagagccggtgtcattaactctggccctg
Pmv13         actcccctggttatgtttatggccagtttgagagaaaaaccaaatataaaagagagccggtgtcattaactctggccctg
Pmv14         actcccctggttatgtttatggccagtttgagagaaaaaccaaatataaaagagagccggtgtcattaactctggccctg
Pmv15         actcccctggttatgtttatggccagtttgagagaaaaaccaGatataaaagagagccggtgtcattaactctggccctg
Pmv16         actcccctggttatgtttatggccagtttgagaAaaaaaccaaatataaaagagagccggtgtcattaactctggccctg
Pmv17         actcccctggttatgtttatggccagtttgagagaaaaaccaaatataaaagagagccggtgtcattaactctggccctg
Pmv18         actcccctggttatgtttatggccagtttgagagaaaaaccaaatataaaagagagccggtgtcattaactctggccctg
Pmv19         actcccctggttatgtttatggccagtttgagagaaaaaccaaatataaaagagagccggtgtcattaactctggccctg
Pmv2          actcccctggttatgtttatggccagtttgagagaaaaaccaaatataaaagagagccggtgtcattaactctggccctg
Pmv20         actcccctggttatgtttatggccagtttgagagaaaaaccaaatataaaagagagccggtgtcattaactctggccctg
Pmv21         actcccctggttatgtttatggccagtttgagaAaaaaaccaaatataaaagagagccggtgtcattaactctggccctg
Pmv22         actcccctggttatgtttatggccagtttgagagaaaaaccaaatataaaagagagccggtgtcattaactctggccctg
Pmv23         actcccctggttatgtttatggccagtttgagagaaaaaccaaatataaaagagagccggtgtcattaactctggccctg
Pmv24         actcccctggttatgtttatggccagtttgagagaaaaaccaaatataaaagagagccggtgtcattaactctggccctg
Pmv4          actcccctAgttatgtttatggccagtttgaAaAaaaaaccaaatataaaagagagccggtgtcattaactctggccctg
Pmv5          actcccctggttatgtttatggccagtttgagaAaaaaaccaaatataaaagagagccggtgtcattaactctggccctg
Pmv6          actcccctggttatgtttatggccagtttgagagaaaaaccaaatataaaagagagccggtgtcattaactctggccctg
Pmv7          actcccctggttatgtttatggccagtttgagaAaaaaaccaaatataaaagagagccggtgtcattaactctggccctg
Pmv8          actcccctggttatgtttatggccagtttgagagaaaaaccaaatataaaagagagccggtgtcattaactctggccctg
Pmv9          actcccctggttatgtttatggccagtttgagagaaaaaccaaatataaaagagagccggtgtcattaactctggccctg

PMV_CONS      CTGTTGGGAGGACTTACTATGGGCGGCATAGCTGCAGGAGTAGGAACCGGGACTACAGCCCTAGTGGCCACCAAACAATT
Pmv1          ctgttgggaggacttactatgggcggcatagctgcaggagtaggaaccgggactacagccctagtggccaccaaacaatt
Pmv10         ctgttgggaggacttactatgggcggcatagctgcaggagtaggaaccgggactacagccctagtggccaccaaacaatt
Pmv11         ctgttgggaggacttactatgggcggcatagctgcaggagtaggaaccgggactacagccctagtggccaccaaacaatt
Pmv12         ctgttgggaggacttactatgggcggcatagctgcaggagtaggaaccgggactacagccctagtggccaccaaacaatt
Pmv13         ctgttgggaggacttactatgggcggcatagctgcaggagtaggaaccgggactacagccctagtggccaccaaacaatt
Pmv14         ctgttgggaggacttactatgggcggcatagctgcaggagtaggaaccgggactacagccctagtggccaccaaacaatt
Pmv15         ctgttgggaggacttactatgggcggcatagctgcaggagtaggaaccgggactacagccctagtggccaccaaacaatt
Pmv16         ctgttgggaggacttactatgggcggcatagctgcaggagtaggaaccgggactacagccctagtggccaccaaacaatt
Pmv17         ctgttgggaggacttactatgggcggcatagctgcaggagtaggaaccgggactacagccctagtggccaccaaacaatt
Pmv18         ctgttgggaggacttactatgggcggcatagctgcaggagtaggaaccgggactacagccctagtggccaccaaacaatt
Pmv19         ctgttgggaggacttactatgggcggcatagctgcaggagtaggaaccgggactacagccctagtggccaccaaacaatt
Pmv2          ctgttgggaggacttactatgggcggcatagctgcaggagtaggaaccgggactacagccctagtggccaccaaacaatt
Pmv20         ctgttgggaggacttactatgggcggcatagctgcaggagtaggaaccgggactacagccctagtggccaccaaacaatt
Pmv21         ctgttgggaggacttactatgggcggcatagctgcaggagtaggaaccgggactacagccctagtggccaccaaacaatt
Pmv22         ctgttgggaggacttactatgggcggcatagctgcaggagtaggaaccgggactacagccctagtggccaccaaacaatt
Pmv23         ctgttgggaggacttactatgggcggcatagctgcaggagtaggaaccgggactacagccctagtggccaccaaacaatt
Pmv24         ctgttgggaggacttactatgggcggcatagctgcaggagtaggaaccgggactacagccctagtggccaccaaacaatt
Pmv4          ctgttAAgaAgacttactatgggcggcatagctgcaggagtaAAaaccgggactacagccctagtggccaccaaacaatt
Pmv5          ctgttgggaggacttactatgggcggcatagctgcaggagtaggaaccgggactacagccctagtggccaccaaacaatt
Pmv6          ctgttgggaggacttactatgggcggcatagctgcaggagtaggaaccgggactacagccctagtggccaccaaacaatt
Pmv7          ctgttgggaggacttactatgggcggcatagctgcaggagtaggaaccgggactacagccctagtggccaccaaacaatt
Pmv8          ctgttgggaggacttactatgggcggcatagctgcaggagtaggaaccgggactacagccctagtggccaccaaacaatt
Pmv9          ctgttgggaggacttactatgggcggcatagctgcaggagtagAaaccgggactacagccctagtggccaccaaacaatt
```

```
PMV_CONS      CGAGCAGCTCCAGGCAGCCATACATACAGACCTTGGGGCTTTAGAAAAGTCAGTCAGTGCCCTAGAAAAGTCTCTGACCT
Pmv1          cgagcagctccaggcagccatacatacagaccttggggctttagaaaagtcagtcagtgccctagaaaagtctctgacct
Pmv10         cgagcagctccaggcagccatacatacagaccttggggctttagaaaagtcagtcagtgccctagaaaagtctctgacct
Pmv11         cgagcagctccaggcagccatacatacagaccttggggctttagaaaagtcagtcagtgccctagaaaagtctctgacct
Pmv12         cgagcagctccaggcagccatacatacagaccttggggctttagaaaagtcagtcagtgccctagaaaagtctctgacct
Pmv13         cgagcagctccaggcagccatacatacagaccttggggctttagaaaagtcagtcagtgccctagaaaagtctctgacct
Pmv14         cgagcagctccaggcagccatacatacagaccttggggctttaAaaaagtcagtcagtgccctagaaaagtctctgacct
Pmv15         cgagcagctccaggcagccatacatacagaccttggggctttaAaaaagtcagtcagtgccctaAaaaagtctctgacct
Pmv16         cgagcagctccaggcagccatacatacagaccttggggctttagaaaagtcagtcagtgccctagaaaagtctctgacct
Pmv17         cgagcagctccaggcagccatacatacagaccttggggctttagaaaagtcagtcagtgccctagaaaagtctctgacct
Pmv18         cgagcagctccaggcagccatacatacagaccttggggctttaAaaaagtcagtcagtgccctagaaaagtctctgacct
Pmv19         cgagcagctccaggcagccatacatacagaccttggggctttagaaaagtcagtcagtgccctagaaaagtctctgacct
Pmv2          cgagcagctccaggcagccatacatacagaccttggggctttagaaaagtcagtcagtgccctagaaaagtctctgacct
Pmv20         cgagcagctccaggcagccatacatacagaccttggggctttagaaaagtcagtcagtgccctagaaaagtctctgacct
Pmv21         cgagcagctccaggcagccatacatacagaccttggggctttaAaaaagtcagtcagtgccctagaaaagtctctgacct
Pmv22         cgagcagctccaggcagccatacatacagaccttggggctttagaaaagtcagtcagtgccctagaaaagtctctgacct
Pmv23         cgagcagctccaggcagccatacatacagaccttggggctttagaaaagtcagtcagtgccctaAaaaagtctctgacct
Pmv24         cgagcagctccaggcagccatacatacagaccttggggctttagaaaagtcagtcagtgccctagaaaagtctctgacct
Pmv4          cgagcagctccaggcagccatacatacagaccttggggctttaAaaaagtcagtcagtgccctaAaaaagtctctgacct
Pmv5          cgagcagctccaggcagccatacatacagaccttggggctttagaaaagtcagtcagtgccctagaaaagtctctgacct
Pmv6          cgagcagctccaggcagccatacatacagaccttggggctttagaaaagtcagtcagtgccctagaaaagtctctgacct
Pmv7          cgagcagctccaggcagccatacatacagaccttggggctttaAaaaagtcagtcagtgccctaAaaaagtctctgacct
Pmv8          cgagcagctccaggcagccatacatacagaccttggggctttagaaaagtcagtcagtgccctagaaaagtctctgacct
Pmv9          cgagcagctccaggcagccatacatacagaccttggggctttagaaaagtcagtcagtgccctagaaaagtctctgacct

PMV_CONS      CGTTGTCTGAGGTGGTCCTACAGAACCGGAGGGGATTAGATCTACTGTTCCTAAAAGAAGGAGGATTATGTGCTGCCCTA
Pmv1          cgttgtctgaggtggtcctacagaaccggaggggattagatctactgttcctaaaagaaggaggattatgtgctgcccta
Pmv10         cgttgtctgaggtggtcctacagaaccggaggggattagatctactgttcctaaaagaaggaggattatgtgctgcccta
Pmv11         cgttgtctgaggtggtcctacagaaccggaggggattagatctactgttcctaaaagaaggaggattatgtgctgcccta
Pmv12         cgttgtctgaggtggtcctacagaaTcggaggggattagatctactgttcctaaaagaaggaggattatgtgctgcccta
Pmv13         cgttgtctgaggtggtcctacagaaccggaggggattagatctactgttcctaaaagaaggaggattatgtgctgcccta
Pmv14         cgttgtctgaggtggtcctacagaaccggaggggattagatctactgttcctaaaagaaggaggTttatgtgctgcccta
Pmv15         cgttgtctgaggtggtcctacagaaccggaggggattagatctactgttcctaaaagaaggaggattatgtgctgcccta
Pmv16         cgttgtctgaggtggtcctacagaaccggaggggattagatctactgttcctaaaagaaggaggattatgtgctgcccta
Pmv17         cgttgtctgaggtggtcctacagaaccggaggggattagatctactgttcctaaaagaaggaggattatgtgctgcccta
Pmv18         cgttgtctgaggtggtcctacagaaccggaggggattagatctactgttcctaaaagaaggaggattatgtgctgcccta
Pmv19         cgttgtctgaggtggtcctacagaaccggaggggattagatctactgttcctaaaagaaggaggattatgtgctgcccta
Pmv2          cgttgtctgaggtggtcctacagaaccggaggggattagatctactgttcctaaaagaaggaggattatgtgctgcccta
Pmv20         cgttgtctgaggtggtcctacagaaccggaggAgattagatctactgttcctaaaagaaggaAgattatgtgctgcccta
Pmv21         cgttgtctgaggtggtcctacagaaccggaggggattagatctactgttcctaaaagaaggaggattatgtgctgcccta
Pmv22         cgttgtctgaggtggtcctacagaaccggaggggattagatctactgttcctaaaagaaggaggattatgtgctgcccta
Pmv23         cgttgtctgaggtggtcctacagaaccggaggAgattagatctactgttcctaaaaAaaggaggattatgtgctgcccta
Pmv24         cgttgtctgaggtggtcctacagaaccggaggggattagatctactgttcctaaaagaaggaggattatgtgctgcccta
Pmv4          cgttgtctgaggtggtcctacagaaccggaggggattagatctactgttcctaaaaAaaggaAgattatgtgctgcccta
Pmv5          cgttgtctgaggtggtcctacagaaccggaggggattagatctactgttcctaaaagaaggaggattatgtgctgcccta
Pmv6          cgttgtctgaggtggtcctacagaaccggaggggattagatctactgttcctaaaagaaggaggattatgtgctgcccta
Pmv7          cgttgtctgaggtggtcctacagaaccggaggggattagatctactgttcctaaaagaaggaggattatgtgctgcccta
Pmv8          cgttgtctgaggtggtcctacagaaccggaggggattagatctactgttcctaaaagaaggaggattatgtgctgcccta
Pmv9          cgttgtctgaggtggtcctacagaaccggaggggattagatctactgttcctaaaagaaggaggattatgtgctgcccta
```

```
PMV_CONS      AAAGAAGAATGCTGTTTCTACGCGGACCACACTGGCGTAGTAAGAGATAGCATGGCAAAGCTAAGAGAAAGGTTGAACCA
Pmv1          aaagaagaatgctgtttctacgcggaccacactggcgtagtaagagatagcatggcaaagctaagagaaaggttgaacca
Pmv10         aaaAaagaatgctgtttctacgcggaccacactggcgtagtaagagatagcatggcaaagctaagagaaaggttgaacca
Pmv11         aaagaagaatgctgtttctacgcggaccacactggcgtagtaagagatagcatggcaaagctaagagaaaggttgaacca
Pmv12         aaagaagaatgctgtttctacgcggaccacactggcgtagtaagagatagcatggcaaagctaagagaaaggttgaacca
Pmv13         aaagaagaatgctgtttctacgcggaccacactggcgtagtaagagatagcatggcaaagctaagagaaaggttgaacca
Pmv14         aaagaagaatgctgtttctacgcAgaccacactggcgtagtaagagataAcatggcaaagctaagagaaaggttgaacca
Pmv15         aaagaagaatgctgtttctacgcggaccacactggcgtagtaagagatagcatggcaaagctaagagaaaggttgaacca
Pmv16         aaagaagaatgctgtttctacgcggaccacactggcgtagtaagagatagcatggcaaagctaagagaaaggttgaacca
Pmv17         aaagaagaatgctgtttctacgcggaccacactggcgtagtaagagatagcatggcaaagctaagagaaaggttgaacca
Pmv18         aaagaagaatgctgtttctacgcggaccacactggcgtagtaagagatagcatggcaaagctaagagaaaggttgaacca
Pmv19         aaagaagaatgctgtttctacgcggaccacactggcgtagtaagagatagcatggcaaagctaagagaaaggttgaacca
Pmv2          aaagaagaatgctgtttctacgcggaccacactggcgtagtaagagatagcatggcaaagctaagagaaaggttgaacca
Pmv20         aaaAaagaatgctgtttctacgcggaccacactggcgtagtaagagatagcatggcaaagctaagagaaaggttgaacca
Pmv21         aaagaagaatgctgtttctacgcggaccacactggcgtagtaagagatagcatggcaaagctaagaAaaaggttgaacca
Pmv22         aaagaagaatgctgtttctacgcggaccacactggcgtagtaagagatagcatggcaaagctaagagaaaggttgaacca
Pmv23         aaagaagaatgctgtttctacgcggaccacactggcgtagtaagagatagcatggcaaagctaaAaAaaaggttgaacca
Pmv24         aaagaagaatgctgtttctacgcggaccacactggcgtagtaagagatagcatggcaaagctaagagaaaggttgaacca
Pmv4          aaaAaaAaatgctgtttctacgcggaccacactggcgtagtaagagatagcatggcaaagctaaAaAaaaggttAaacca
Pmv5          aaagaagaatgctgtttctacgcggaccacactggcgtagtaagagatagcatggcaaagctaagagaaaggttgaacca
Pmv6          aaagaagaatgctgtttctacgcggaccacactggcgtagtaagagatagcatggcaaagctaagagaaaggttgaacca
Pmv7          aaagaagaatgctgtttctacgcggaccacactggcgtagtaagagatagcatggcaaagctaagagaaaggttgaacca
Pmv8          aaagaagaatgctgtttctacgcggaccacactggcgtagtaagagatagcatggcaaagctaagagaaaggttgaacca
Pmv9          aaagaaAaatgctgtttctacgcggaccacactggcgtagtaagagatagcatggcaaagctaagagaaaggttAaacca

PMV_CONS      GAGACAAAAATTGTTCGAATCAGGACAAGGGTGGTTTGAGGGACTGTTTAACAGGTCCCCATGGTTCACGACCTTAATAT
Pmv1          gagacaaaaattgttcgaatcaggacaagggtggtttgagggactgtttaacaggtccccatggttcacgaccttaatat
Pmv10         gagacaaaaattgttcgaatcaggacaagggtggtttgagggactgtttaacaggtccccatggttcacgaccttaatat
Pmv11         gagacaaaaattgttcgaatcaggacaagggtggtttgagggactgtttaacaggtccccatggttcacgaccttaatat
Pmv12         gagacaaaaattgttcgaatcaggacaagggtggtttgagggactgtttaacaggtccccatggttcacgaccttaatat
Pmv13         gagacaaaaattgttcgaatcaggacaagggtggtttgagggactgtttaacaggtccccatggttcacgaccttaatat
Pmv14         gagacaaaaattgttcgaatcaggacaagggtggtttgagggactgtttaacaggtccccatggttcacgaccttaatat
Pmv15         gagacaaaaattgttcAaatcaggacaagggtggtttgagggactgtttaacaggtccccatggttcacgaccttaatat
Pmv16         gagacaaaaattgttcgaatcaggacaagggtggtttgagggactgtttaacaggtccccatggttcacgaccttaatat
Pmv17         gagacaaaaattgttcgaatcaggacaagggtggtttgagggactgtttaacaggtccccatggttcacgaccttaatat
Pmv18         gagacaaaaattgttcAaatcaggacaagggtggtttgagggactgtttaacaggtccccatggttcacgaccttaatat
Pmv19         gagacaaaaattgttcgaatcaggacaagggtggtttgagggactgtttaacaggtccccatggttcacgaccttaatat
Pmv2          gagacaaaaattgttcgaatcaggacaagggtggtttgagggactgtttaacaggtccccatggttcacgaccttaatat
Pmv20         gagacaaaaattgttcgaatcaggacaagggtggtttgagggactgtttaacaggtccccatggttcacgaccttaatat
Pmv21         gagacaaaaattgttcgaatcaggacaagggtggtttgaAggactgtttaacaggtccccatggttcacgaccttaatat
Pmv22         gagacaaaaattgttcAaatcaggacaagggtggtttgagggactgtttaacaggtccccatggttcacgaccttaatat
Pmv23         gagacaaaaattgttcgaatcaggacaagggtggtttgagggactgtttaacaggtccccatggttcacgaccttaatat
Pmv24         gagacaaaaattgttcgaatcaggacaagggtggtttgagggactgtttaacaggtccccatggttcacgaccttaatat
Pmv4          gagacaaaaattgttcgaatcaggacaagggtggtttAagggactgtttaacaggtccccatggttcacgaccttaatat
Pmv5          gagacaaaaattgttcgaatcaggacaagggtggtttgagggactgtttaacaggtccccatggttcacgaccttaatat
Pmv6          gagacaaaaattgttcgaatcaggacaagggtggtttgagggactgtttaacaggtccccatggttcacgaccttaatat
Pmv7          gagacaaaaattgttcgaatcaggacaagggtggtttgagggactgtttaacaggtccccatggttcacgaccttaatat
Pmv8          gagacaaaaattgttcgaatcaggacaagggtggtttgagggactgtttaacaggtccccatggttcacgaccttaatat
Pmv9          gagacaaaaattgttcgaatcaggacaagggtggtttgagggactgtttaacaggtccccatggttcacgaccttaatat
```

```
PMV_CONS      CCACCATTATGGGCCCCTTGATAATACTTTTATTAATCCTACTCTTCGGACCCTGTATTCTCAACCGCTTGGTCCAGTTT
Pmv1          cTaccattatgggccccttgataatacttttattaatcctactcttcggaccctgtattctcaaccgcttggtccagttt
Pmv10         ccaccattatgggccccttgataatacttttattaatcctactcttcggaccctgtattctcaaccgcttggtccagttt
Pmv11         cTaccattatgggccccttgataatacttttattaatcctactcttcggaccctgtattctcaaccgcttggtccagttt
Pmv12         ccaccattatgggcccctt Aataatacttttattaatcctactcttcggaccctgtattctcaaccgcttggtccagttt
Pmv13         ccaccattatgggccccttgataatacttttattaatcctactcttcggaccctgtattctcaaccgcttggtccagttt
Pmv14         ccaccattatgggcccctt Aataatacttttattaatcctactcttcggaccctgtattctcaaccgcttggtccagttt
Pmv15         ccaccattatgggccccttgataatacttttattaatcctactcttcggaccctgtattctcaaccgcttggtccagttt
Pmv16         ccaccattatgggccccttgataatacttttattaatcctactcttcggaccctgtattctcaaccgcttggtccagttt
Pmv17         ccaccattatgggcccctt Aataatacttttattaatcctactcttcggaccctgtattctcaaccgcttggtccagttt
Pmv18         ccaccattatgggcccctt Aataatacttttattaatcctactcttcggaccctgtattctcaaccgcttggtccagttt
Pmv19         ccaccattatgggccccttgataatacttttattaatcctactcttcggaccctgtattctcaaccgcttggtccagttt
Pmv2          ccaccattatgggccccttgataatacttttattaatcctactcttcggaccctgtattctcaaccgcttggtccagttt
Pmv20         ccaccattatgggccccttgataatacttttattaatcctactcttcggaccctgtattctcaaccgcttggtccagttt
Pmv21         ccaccattatgggccccttgataatacttttattaatcctactcttcggaccctgtattctcaaccgcttggtccagttt
Pmv22         ccaccattatgggccccttgataatacttttattaatcctactcttcggaccctgtattctcaaccgcttggtccagttt
Pmv23         ccaccattatgggccccttgataatacttttattaatcctactcttcggaccctgtattctcaaccgcttggtccagttt
Pmv24         ccaccattatgggccccttgataatacttttattaatcctactcttcggaccctgtattctcaaccgcttggtccagttt
Pmv4          ccaccattatgggccccttgataatacttttattaatcctactcttcggaccctgtattctcaaccgcttggtccagttt
Pmv5          cTaccattatgggccccttgataatacttttattaatcctactcttcggaccctgtattctcaaccgcttggtccagttt
Pmv6          ccaccattatgggccccttgataatacttttattaatcctactcttcggaccctgtattctcaaccgcttggtccagttt
Pmv7          ccaccattatgggccccttgataatacttttattaatcctactcttcggaccctgtattctcaaccgcttggtccagttt
Pmv8          ccaccattatgggccccttgataatacttttattaaAcctactcttcggaccctgtattctcaaccgcttggtccagttt
Pmv9          ccaccattatgggccccttgataatacttttattaatcctactcttcggaccctgtattctcaaccgcttggtccagttt

PMV_CONS      GTAAAAGACAGAATTTCGGTAGTG
Pmv1          gtaaaagacagaatttcggtagtg
Pmv10         gtaaaagacagaatttcggtagtg
Pmv11         gtaaaagacagaatttcggtagtg
Pmv12         gtaaaagacagaatttcggtagtg
Pmv13         gtaaaagacagaatttcggtagtg
Pmv14         gtaaaagacagaatttcggtagtg
Pmv15         gtaaaagacagaatttcggtagtg
Pmv16         gtaaaagacagaatttcggtagtg
Pmv17         gtaaaagacagaatttcggtagtg
Pmv18         gtaaaagacagaatttcggtagtg
Pmv19         gtaaaagacagaatttcggtagtg
Pmv2          gtaaaagacagaatttcggtagtg
Pmv20         gtaaaagacagaatttcggtagtg
Pmv21         gtaaaagacagaatttcggtagtg
Pmv22         gtaaaagacagaatttcggtagtg
Pmv23         gtaaaagacagaatttcggtagtg
Pmv24         gtaaaagacagaatttcggtagtg
Pmv4          gtaaaagacaAaatttcggtagtg
Pmv5          gtaaaagacagaatttcggtagtg
Pmv6          gtaaaagacagaatttcggtagtg
Pmv7          gtaaaagacagaatttcggtagtg
Pmv8          gtaaaagacagaatttcggtagtg
Pmv9          gtaaaagacagaatttcggtagtg
```

Mpmv (concatenated *gag*, *pol* and *env*)

```
MPMV_CONS      ATGGGACAGACCGTAACTACCCCTCTGAGTCTAACCTTGCAGCACTGGGGAGATGTCCAGCGCATTGCATCCAACCAGTC
Mpmv1          atgggacagaccgtaactacccctctgagtctaaccttgcagcactggggagatgtccagcgcattgcatccaaccagtc
Mpmv10         atgggacagaccgtaactacccctctgagtctaaccttgcagcactggggagatgtccagcgcattgcatccaaccagtc
Mpmv11         atgggacagaccgtaactacccctctgagtctaaccttgcagcactggggagatgtccagcgcattgcatccaaccagtc
Mpmv12         atgggacagaccgtaactacccctctgagtctaaccttgcagcactggggagatgtccagcgcattgcatccaaccagtc
Mpmv13         atgggacagaccgtaactacccctctgagtctaaccttgcagcactggggagatgtccagcgcattgcatccaaccagtc
Mpmv2          atgggacagaccgtaactacccctctgagtctaaccttgcagcactggggagatgtccagcgcattgcatccaaccagtc
Mpmv3          atgggacagaccgtaactacccctctgagtctaaccttgcagcactggggagatgtccagcgcattgcatccaaccagtc
Mpmv4          atgggacagaccgtaactacccctctgagtctaaccttgcagcactggggagatgtccagcgcattgcatccaaccagtc
Mpmv5          atgggacagaccgtaactacccctctgagtctaaccttgcagcactggggagatgtccagcgcattgcatccaaccagtc
Mpmv6          atgggacagaccgtaactacccctctgagtctaaccttgcagcactggggagatgtccagcgcattgcatccaaccagtc
Mpmv7          atgggacagaccgtaactacccctctgagtctaaccttgcagcactggggagatgtccagcgcattgcatccaaccagtc
Mpmv8          atgggacagaccgtaactacccctctgagtctaaccttgcagcactggggagatgtccagcgcattgcatccaaccagtc
Mpmv9          atgggacagaccgtaactacccctctgagtctaaccttgcagcactggggagatgtccagcgcattgcatccaaccagtc

MPMV_CONS      TGTGGATGTCAGGAAGAGGCGCTGGATTACCTTCTGTTCCGCCGAATGGCCAACTTTCAATGTAGGATGGCCTCAGGATG
Mpmv1          tgtggatgtcaggaagaggcgctggattaccttctgttccgccgaatggccaactttcaatgtaggatggcctcaggatg
Mpmv10         tgtggatgtcaggaagaggcgctggattaccttctgttccgccgaatggccaactttcaatgtaggatggcctcaggatg
Mpmv11         tgtggatgtcaggaagaggcgctggattaccttctgttccgccgaatggccaactttcaatgtaggatggcctcaggatg
Mpmv12         tgtggatgtcaggaagaggcgctggattaccttctgttccgccgaatggccaactttcaatgtaggatggcctcaggatg
Mpmv13         tgtggatgtcaggaagaggcgctggattaccttctgttccgccgaatggccaactttcaatgtaggatggcctcaggatg
Mpmv2          tgtggatgtcaggaagaggcgctggattaccttctgttccgccgaatggccaactttcaatgtaggatggcctcaggatg
Mpmv3          tgtggatgtcaggaagaggcgctggattaccttctgttccgccgaatggccaactttcaatgtaggatggcctcaggatg
Mpmv4          tgtggatgtcaggaagaggcgctggattaccttctgttccgccgaatggccaactttcaatgtaggatggcctcaggatg
Mpmv5          tgtggatgtcaggaagaggcgctggattaccttctgttccgccgaatggccaactttcaatgtaggatggcctcaggatg
Mpmv6          tgtggatgtcaggaagaggcgctggattaccttctgttccgccgaatggccaactttcaatgtaggatggcctcaggatg
Mpmv7          tgtggatgtcaggaagaggcgctggattaccttctgttccgccgaatggccaactttcaatgtaggatggcctcaggatg
Mpmv8          tgtggatgtcaggaagaggcgctggattaccttctgttccgccgaatggccaactttcaatgtaggatggcctcaggatg
Mpmv9          tgtggatgtcaggaagaggcgctggattaccttctgttccgccgaatggccaactttcaatgtGggatggcctcaggatg

MPMV_CONS      GTACTTTCAATTTAAGTATTATCTCTCAGGTTAAGTCTAGAGTGTTTTGTCCTGGTCCCCACGGACACCCGGATCAGGTC
Mpmv1          gtactttcaatttaagtattatctctcaggttaagtctagagtgttttgtcctggtccccacggacacccggatcaggtc
Mpmv10         gtactttcaatttaagtattatctctcaggttaagtctagagtgttttgtcctggtccccacggacacccggatcaggtc
Mpmv11         gtactttcaatttaagtattatctctcaggttaagtctagagtgttttgtcctggtccccacggacacccggatcaggtc
Mpmv12         gtactttcaatttaagtattatctctcaggttaagtctagagtgttttgtcctggtccccacggacacccggatcaggtc
Mpmv13         gtactttcaatttaagtattatctctcaggttaagtctagagtgttttgtcctggtccccacggacacccggatcaggtc
Mpmv2          gtactttcaatttaagtattatctctcaggttaagtctagagtgttttgtcctggtccccacggacacccggatcaggtc
Mpmv3          gtactttcaatttaagtattatctctcaggttaagtctagagtgttttgtcctggtccccacggacacccggatcaggtc
Mpmv4          gtactttcaatttaagtattatctctcaggttaagtctagagtgttttgtcctggtccccacggacacccggatcaggtc
Mpmv5          gtactttcaatttaagtattatctctcaggttaagtctagagtgttttgtcctggtccccacggacacccggatcaggtc
Mpmv6          gtactttcaatttaagtattatctctcaggttaagtctagagtgttttgtcctggtccccacggacacccggatcaggtc
Mpmv7          gtactttcaatttaagtattatctctcaggttaagtctagagtgttttgtcctggtccccacggacacccggatcaggtc
Mpmv8          gtactttcaatttaagtattatctctcaggttaagtctagagtgttttgtcctggtccccacggacacccggatcaggtc
Mpmv9          gtactttcaatttaagtattatctctcaggttaagtctagagtgttttgtcctggtccccacggacacccggatcaggtc

MPMV_CONS      CCATATATCGTTACCTGGGAGGCACTTGCCTATGACCCCCCTCCGTGGGTCAAACCGTTTGTTTCTCCTAAACCTCCTCC
Mpmv1          ccatatatcgttacctgggaggcacttgcctatgacccccctccgtgggtcaaaccgtttgtttctcctaaacctcctcc
Mpmv10         ccatatatcgttacctgggaggcacttgcctatgacccccctccgtgggtcaaaccgtttgtttctcctaaacctcctcc
Mpmv11         ccatatatcgttacctgggaggcacttgcctatgacccccctccgtgggtcaaaccgtttgtttctcctaaacctcctcc
Mpmv12         ccatatatcgttacctgggaggcacttgcctatgacccccctccgtgggtcaaaccgtttgtttctcctaaacctcctcc
Mpmv13         ccatatatcgttacctgggaggcacttgcctatgacccccctccgtgggtcaaaccgtttgtttctcctaaacctcctcc
Mpmv2          ccatatatcgttacctgggaggcacttgcctatgacccccctccgtgggtcaaaccgtttgtttctcctaaacctcctcc
Mpmv3          ccatatatcgttacctgggaggcacttgcctatgacccccctccgtgggtcaaaccgtttgtttctcctaaacctcctcc
Mpmv4          ccatatatcgttacctgggaggcacttgcctatgacccccctccgtgggtcaaaccgtttgtttctcctaaacctcctcc
Mpmv5          ccatatatcgttacctgggaggcacttgcctatgacccccctccgtgggtcaaaccgtttgtttctcctaaacctcctcc
Mpmv6          ccatatatcgttacctgAgaggcacttgcctatgacccccctccgtgggtcaaaccgtttgtttctcctaaacctcctcc
Mpmv7          ccatatatcgttacctgggaggcacttgcctatgacccccctccgtgggtcaaaccgtttgtttctcctaaacctcctcc
Mpmv8          ccatatatcgttacctgggaggcacttgcctatgacccccctccgtgggtcaaaccgtttgtttctcctaaacctcctcc
Mpmv9          ccatatatcgttacctgggaggcacttgcctatgacccccctccgtgggtcaaaccgtttgtttctcctaaacctcctcc
```

```
MPMV_CONS     CTTGCCGACAGCTCCCGTCCTCCCGCCCGGTCCTTCTGCGCAACCTCCGTCCCGATCTGCCCTTTACCCTGCCCTTACCC
Mpmv1         cttgccgacagctcccgtcctcccgcccggtccttctgcgcaacctccgtcccgatctgccctttaccctgcccttaccc
Mpmv10        cttgccgacagctcccgtcctcccgcccggtccttctgcgcaacctccgtcccgatctgccctttaccctgcccttaccc
Mpmv11        cttgccgacagctcccgtcctcccgcccggtccttctgcgcaacctccgtcccgatctgccctttaccctgcccttaccc
Mpmv12        cttgccgacagctcccgtcctcccgcccggtccttctgcgcaacctccgtcccgatctgccctttaccctgcccttaccc
Mpmv13        cttgccgacagctcccgtcctcccgcccggtccttctgcgcaacctccgtcccgatctgccctttaccctgcccttaccc
Mpmv2         cttgccgacagctcccgtcctcccgcccggtccttctgcgcaacctccgtcccgatctgccctttaccctgcccttaccc
Mpmv3         cttgccgacagctcccgtcctcccgcccggtccttctgcgcaacctccgtcccgatctgccctttaccctgcccttaccc
Mpmv4         cttgccgacagctcccgtcctcccgcccggtccttctgcgcaacctccgtcccgatctgccctttaccctgcccttaccc
Mpmv5         cttgccgacagctcccgtcctcccgcccggtccttctgcgcaacctccgtcccgatctgccctttaccctgcccttaccc
Mpmv6         cttgccgacagctcccgtcctcccgcccggtccttctgcgcaacctccgtcccgatctgccctttaccctgcccttaccc
Mpmv7         cttgccgacagctcccgtcctcccgcccggtccttctgcgcaacctccgtcccgatctgccctttaccctgcccttaccc
Mpmv8         cttgccgacagctcccgtcctcccgcccggtccttctgcgcaacctccgtcccgatctgccctttaccctgcccttaccc
Mpmv9         cttgccgacagctcccgtcctcccgcccggtccttctgcgcaacctccgtcccgatctgccctttaccctgcccttaccc

MPMV_CONS     CCTCTATAAAGTCCAAACCTCCTAAGCCCCAGGTTCTCCCTGATAGCGGCGGACCTCTCATTGACCTTCTCACAGAGGAC
Mpmv1         cctctataaagtccaaacctcctaagccccaggttctccctgatagcggcggacctctcattgaccttctcacagaggac
Mpmv10        cctctataaagtccaaacctcctaagccccaggttctccctgatagcggcggacctctcattgaccttctcacagaggac
Mpmv11        cctctataaagtccaaacctcctaagccccaggttctccctgatagcggcggacctctcattgaccttctcacagaggac
Mpmv12        cctctataaagtccaaacctcctaagccccaggttctccctgatagcggcggacctctcattgaccttctcacagaggac
Mpmv13        cctctataaagtccaaacctcctaagccccaggttctccctgatagcggcggacctctcattgaccttctcacagaggac
Mpmv2         cctctataaagtccaaacctcctaagccccaggttctccctgatagcggcggacctctcattgaccttctcacagaggac
Mpmv3         cctctataaagtccaaacctcctaagccccaggttctccctgatagcggcggacctctcattgaTcttctcacagaggac
Mpmv4         cctctataaagtccaaacctcctaagccccaggttctccctgatagcggcggacctctcattgaccttctcacagaggac
Mpmv5         cctctataaagtccaaacctcctaagccccaggttctccctgatagcggcggacctctcattgaccttctcacagaggac
Mpmv6         cctctataaagtccaaacctcctaagccccaggttctccctgatagcggcggacctctcattgaccttctcacagaggac
Mpmv7         cctctataaagtccaaacctcctaagccccaggttctccctgatagcggcggacctctcattgaccttctcacagaggac
Mpmv8         cctctataaagtccaaacctcctaagccccaggttctccctgatagcggcggacctctcattgaccttctcacagaggac
Mpmv9         cctctataaagtccaaacctcctaagccccaggttctccctgatagcggcggacctctcattgaccttctcacagaggac

MPMV_CONS     CCCCCGCCGTACGGAGCACAACCTTCCTCCTCTGCCAGAGAGAACGATGAAGAAGAGGCGGCCACCACCTCCGAGGTTTC
Mpmv1         cccccgccgtacggagcacaaccttcctcctctgccagagagaacgatgaagaagaggcggccaccacctccgaggtttc
Mpmv10        cccccgccgtacggagcacaaccttcctcctctgccagagagaacgatgaagaagaggcggccaccacctccgaggtttc
Mpmv11        cccccgccgtacggagcacaaccttcctcctctgccagagagaacgatgaagaagaggcggccaccacctccgaggtttc
Mpmv12        cccccgccgtacggagcacaaccttcctcctctgccagagagaacgatgaagaagaggcggccaccacctccgaggtttc
Mpmv13        cccccgccgtacggagcacaaccttcctcctctgccagagagaacgatgaagaagaggcggccaccacctccgaggtttc
Mpmv2         cccccgccgtacggagcacaaccttcctcctctgccagagagaacgatgaagaagaggcggccaccacctccgaggtttc
Mpmv3         cccccgccgtacggagcacaaccttcctcctctgccagagagaacgatgaaAaagaggcggccaccacctccgaggtttc
Mpmv4         cccccgccgtacggagcacaaccttcctcctctgccagagagaacgatgaagaagaggcggccaccacctccgaggtttc
Mpmv5         cccccgAcg------gcacaaccttcctcctctgccagagagaacAatgaaAaagaggcggccaccacctccgaggtttc
Mpmv6         cccccgccgtacggagcacaaccttcctcctctgccagagagaacgatgaagaagaggcAgccaccacctccgaggtttc
Mpmv7         cccccgccgtacggagcacaaccttcctcctctgccagagagaacgatgaagaagaggcggccaccacctccgaggtttc
Mpmv8         cccccgccgtacggagcacaaccttcctcctctgccagagagaacgatgaagaagaggcggccaccacctccgaggtttc
Mpmv9         cccccgccgtacggagcacaaccttcctcctctgccagagagaacgatgaagaagaggcggccaccacctccgaggtttc

MPMV_CONS     CCCCCCTTCTCCCATGGCGTCTCGACTGCGGGGAAGGAGAGACCCTCCCGCAGCGGACTCCACCTCCTCCCAGGCATTCC
Mpmv1         ccccccttctcccatggcgtctcgactgcggggaaggagagaccctcccgcagcggactccacctcctcccaggcattcc
Mpmv10        ccccccttctcccatggcgtctcgactgcggggaaggagagaccctcccgcagcggactccacctcctcccaggcattcc
Mpmv11        ccccccttctcccatggcgtctcgactgcggggaaggagagaccctcccgcagcggactccacctcctcccaggcattcc
Mpmv12        ccccccttctcccatggcgtctcgactgcggggaaggagagaccctcccgcagcggactccacctcctcccaggcattcc
Mpmv13        ccccccttctcccatggcgtctcgactgcggggaaggagagaccctcccgcagcggactccacctcctcccaggcattcc
Mpmv2         ccccccttctcccatggcgtctcgactgcggggaaggagagaccctcccgcagcggactccacctcctcccaggcattcc
Mpmv3         ccccccttctcccatggcgtctcgactgcggggaaggagagaccctcccgcagcggactccacctcctcccaggcattcc
Mpmv4         ccccccttctcccatggcgtctcgactgcggggaaggagagaccctcccgcagcggactccacctcctcccaggcattcc
Mpmv5         ccccccttctcccatggcgtctcgactgcggggaaggagagaccctcccgcagcggactccacctcctcccaggcattcc
Mpmv6         ccccccttctcccatggcgtctcgactgcgggAaaggagagaccctcccgcagcggactccacctcctcccaggcattcc
Mpmv7         ccccccttctcccatggcgtctcgactgcggggaaggagagaccctcccgcagcggactccacctcctcccaggcattcc
Mpmv8         ccccccttctcccatggcgtctcgactgcggggaaggagagaccctcccgcagcggactccacctcctcccaggcattcc
Mpmv9         ccccccttctcccatggcgtctcgactgcggggaaggagagaccctcccgcagcggactccacctcctcccaggcattcc
```

```
MPMV_CONS     CACTCCGTATGGGGGGAGATGGCCAGCTTCAGTACTGGCCGTTTTCCTCCTCTGACTTATATAATTGGAAAAATAATAAC
Mpmv1         cactccgtatggggggagatggccagcttcagtactggccgttttcctcctctgacttatataattggaaaaataataac
Mpmv10        cactccgtatggggggagatggccagcttcagtactggccgttttcctcctctgacttatataattggaaaaataataac
Mpmv11        cactccgtatggggggagatggccagcttcagtactggccgttttcctcctctgacttatataattAgaaaaataataac
Mpmv12        cactccgtatggggggagatggccagcttcagtactggccgttttcctcctctgacttatataattggaaaaataataac
Mpmv13        cactccgtatggggggagatggccagcttcagtactggccgttttcctcctctgacttatataattggaaaaataataac
Mpmv2         cactccgtatggggggagatggccagcttcagtactggccgttttcctcctctgacttatataattggaaaaataataac
Mpmv3         cactccgtatggggggagatggccagcttcagtactggccgttttcctcctctgacttatataattggaaaaataataac
Mpmv4         cactccgtatggggggagatggccagcttcagtactggccgttttcctcctctgacttatataattggaaaaataataac
Mpmv5         cactccgtatggggggagatggccagcttcagtactggccgttttcctcctctgacttatataattggaaaaataataac
Mpmv6         cactccgtatggggggagatggccagcttcagtactggccgttttcctcctctgacttatataattggaaaaataataac
Mpmv7         cactccgtatggggggagatggccagcttcagtactggccgttttcctcctctgacttatataattggaaaaataataac
Mpmv8         cactccgtatggggggagatggccagcttcagtactggccgttttcctcctctgacttatataattAAaaaaataataac
Mpmv9         cactccgtatggggggagatggccagcttcagtactggccgttttcctcctctgacttatataattggaaaaataataac

MPMV_CONS     CCTTCCTTTTCTGAAGATCCAGGTAAATTGACGGCCTTGATTGAGTCCGTCCTCATCACCCACCAGCCCACCTGGGACGA
Mpmv1         ccttccttttctgaagatccaggtaaattgacggccttgattgagtccgtcctcatcacccaccagcccacctgggacga
Mpmv10        ccttccttttctgaagatccaggtaaattgacggccttgattgagtccgtcctcatcacccaccagcccacctgggacga
Mpmv11        ccttccttttctgaagatccaggtaaattgacggccttgattgagtccgtcctcatcacccaccagcccacctgggacga
Mpmv12        ccttccttttctgaagatccaggtaaattgacggccttgattgagtccgtcctcatcacccaccagcccacctgggacga
Mpmv13        ccttccttttctgaagatccaggtaaattgacggccttgattgagtccgtcctcatcacccaccagcccacctgggacga
Mpmv2         ccttccttttctgaagatccaggtaaattgacggccttgattgagtccgtcctcatcacccaccagcccacctgggacga
Mpmv3         cctCccttttctgaagatccaggtaaattgacggccttgattgagtccgtcctcatcacccaccagcccacctgggacga
Mpmv4         ccttccttttctgaagatccaggtaaattgacggccttgattgagtccgtcctcatcacccaccagcccacctgggacga
Mpmv5         ccttccttttctgaagatccaggtaaattgacggccttgattgagtccgtcctcatcacccaccagcccacctgggacga
Mpmv6         ccttccttttctgaagatccaggtaaattgacggccttgattgagtccgtcctcatcacccaccagcccacctgggacga
Mpmv7         ccttccttttctgaagatccaggtaaattgacggccttgattgagtccgtcctcatcacccaccagcccacctgggacga
Mpmv8         ccttccttttctgaagatccaggtaaattgacggccttgattgagtccgtcctcatcacccaccagcccacctgggacga
Mpmv9         ccttccttttctgaagatccaggtaaattgacggccttgattgagtccgtcctcatcacccaccagcccacctgggacga

MPMV_CONS     CTGTCAGCAGTTGTTGGGGACCCTGCTGACCGGAGAAGAAAAGCAGCGGGTGCTCCTAGAGGCTAGAAAGGCAGTCCGGG
Mpmv1         ctgtcagcagttgttggggaccctgctgaccggagaagaaaagcagcgggtgctcctagaggctagaaaggcagtccggg
Mpmv10        ctgtcagcagttgttggggaccctgctgaccggagaagaaaagcagcgggtgctcctagaggctagaaaggcagtccggg
Mpmv11        ctgtcagcagttgttggggaccctgctgaccggagaagaaaagcagcgggtgctcctagaggctagaaaggcagtccggg
Mpmv12        ctgtcagcagttgttggggaccctgctgaccggagaagaaaagcagcgggtgctcctagaggctagaaaggcagtccggg
Mpmv13        ctgtcagcagttgttggggaccctgctgaccggagaagaaaagcagcgggtgctcctagaggctagaaaggcagtccggg
Mpmv2         ctgtcagcagttgttggggaccctgctgaccggagaagaaaagcagcgggtgctcctagaggctagaaaggcagtccggg
Mpmv3         ctgtcagcagttgttggggaccctgctgaccggagaagaaaagcagcgggtgctcctagaggctagaaaggcagtccggg
Mpmv4         ctgtcagcagttgttggggaccctgctgaccggagaagaaaagcagcgggtgctcctagaggctagaaaggcagtccggg
Mpmv5         ctgtcagcagttgttggggaccctgctgaccggagaaAaaaagcagcgggtgctcctagaggctagaaaggcagtccggg
Mpmv6         ctgtcagcagttgttggggaccctgctgaccggagaagaaaagcagcgggtgctcctagaggctagaaaggcagtccggg
Mpmv7         ctgtcagcagttgttggggaccctgctgaccggagaagaaaagcagcgggtgctcctagaggctagaaaggcagtccggg
Mpmv8         ctgtcagcagttgttggggaccctgctgaccggagaagaaaagcagcgggtgctcctagaggctagaaaggcagtccggg
Mpmv9         ctgtcagcagttgttggggaccctgctgaccggagaagaaaagcagcgggtgctcctagaggctagaaaggcagtccggg

MPMV_CONS     GCAATGATGGACGCCCCACTCAGTTGCCTAATGAAATCAATGCTGCCTTTCCCCTTGAGCGCCCTGATTGGGATTACACC
Mpmv1         gcaatgatggacgccccactcagttgcctaatgaaatcaatgctgcctttccccttgagcgccctgattgggattacacc
Mpmv10        gcaatgatggacgccccactcagttgcctaatgaaatcaatgctgcctttccccttgagcgccctgattgggattacacc
Mpmv11        gcaatgatggacgccccactcagttgcctaatgaaatcaatgctgcctttcccctCgagcgccctgattgggattacacc
Mpmv12        gcaatgatggacgccccactcagttgcctaatgaaatcaatgctgcctttccccttgagcgccctgattgggattacacc
Mpmv13        gcaatgatggacgccccactcagttgcctaatgaaatcaatgctgcctttccccttgagcgccctgattgggattacacc
Mpmv2         gcaatgatggacgccccactcagttgcctaatgaaatcaatgctgcctttccccttgagcgccctgattgggattacacc
Mpmv3         gcaatgatggacgccccactcagttgcctaatgaaatcaatgctgcctttccccttgagcgccctgattgggattacacc
Mpmv4         gcaatgatggacgccccactcagttgcctaatgaaatcaatgctgcctttccccttgagcgccctgattgggattacacc
Mpmv5         gcaatgatggacgccccactcagttgcctaatgaaatcaatgctgcctttccccttgagcgccctgattgggattacacc
Mpmv6         gcaatgatggacgccccactcagttgcctaatgaaatcaatgctgcctttccccttgagcgccctgattgggattacacc
Mpmv7         gcaatgatggacgccccactcagttgcctaatgaaatcaatgctgcctttccccttgagcgccctgattgggattacacc
Mpmv8         gcaatgatggacgccccactcagttgcctaatgaaatcaatgctgcctttccccttgagcgccctgattgggattacacc
Mpmv9         gcaatgatggacgccccactcagttgcctaatgaaatcaatgctgcctttccccttgagcgccctgattgggattacacc
```

```
MPMV_CONS     ACTACAGAAGGTAGGAACCACCTAGTCCTCTATCGCCAGTTGCTCTTAGCGGGTCTCCAAAACGCGGGCAGAAGCCCCAC
Mpmv1         actacagaaggtaggaaccacctagtcctctatcgccagttgctcttagcgggtctccaaaacgcgggcagaagccccac
Mpmv10        actacagaaggtaggaaccacctagtcctctatcgccagttgctcttagcgggtctccaaaacgcgggcagaagccccac
Mpmv11        actacagaaggtaggaaccacctagtcctctatcgccagttgctcttagcgggtctccaaaacgcgggcagaagccccac
Mpmv12        actacagaaggtaggaaccacctagtcctctatcgccagttgctcttagcgggtctccaaaacgcgggcagaagccccac
Mpmv13        actacagaaggtaggaaccacctagtcctctatcgccagttgctcttagcgggtctccaaaacgcgggcagaagccccac
Mpmv2         actacagaaggtaggaaccacctagtcctctatcgccagttgctcttagcgggtctccaaaacgcgggcagaagccccac
Mpmv3         actacagaaggtaggaaccacctagtcctctatcgccagttgctcttagcgggtctccaaaacgcgggcagaagccccac
Mpmv4         actacagaaggtaggaaccacctagtcctctatcgccagttgctcttagcgggtctccaaaacgcgggcagaagccccac
Mpmv5         actacagaaggtaggaaccacctagtcctctatcgccagttgctcttagcgggtctccaaaacgcgggcagaagTcccac
Mpmv6         actacagaaggtaggaaccacctagtcctctatcgccagttgctcttagcgggtctccaaaacgcgggcagaagccccac
Mpmv7         actacagaaggtaggaaccacctagtcctctatcgccagttgctcttagcgggtctccaaaacgcgggcagaagccccac
Mpmv8         actacagaaggtaggaaccacctagtcctctatcgccagttgctcttagcgggtctccaaaacgcgggcagaagccccac
Mpmv9         actacagaaggtaggaaccacctagtcctctatcgccagttgctcttagcgggtctccaaaacgcgggcagaagccccac

MPMV_CONS     CAATTTGGCCAAGGTAAAAGGGATAACCCAGGGACCTAATGAGTCTCCCTCAGCCTTTTTAGAGAGACTCAAGGAGGCCT
Mpmv1         caatttggccaaggtaaaagggataacccagggacctaatgagtctccctcagcctttttagagagactcaaggaggcct
Mpmv10        caatttggccaaggtaaaagggataacccagggacctaatgagtctccctcagcctttttagagagactcaaggaggcct
Mpmv11        caatttggccaaggtaaaagggataacccagggacctaatgagtctccctcagcctttttagagagactcaaggaggcct
Mpmv12        caatttggccaaggtaaaagggataacccagggacctaatgagtctccctcagcctttttagagagactcaaggaggcct
Mpmv13        caatttggccaaggtaaaagggataacccagggacctaatgagtctccctcagcctttttagagagactcaaggaggcct
Mpmv2         caatttggccaaggtaaaagggataacccagggacctaatgagtctccctcagcctttttagagagactcaaggaggcct
Mpmv3         caatttggccaaggtaaaagggataacccagggacctaatgagtctccctcagcctttttagagagactcaaggaggcct
Mpmv4         caatttggccaaggtaaaagggataacccagggacctaatgagtctccctcagcctttttagagagactcaaggaggcct
Mpmv5         caatttggccaaggtaaaagggataacccagggacctaatgagtctccctcagcctttttagagagactcaaggaggcct
Mpmv6         caatttggccaaggtaaaagggataacccagggacctaatgagtctccctcagcctttttagagagactcaaggaggcct
Mpmv7         caatttggccaaggtaaaagggataacccagggacctaatgagtctccctcagcctttttagagagactcaaggaggcct
Mpmv8         caatttggccaaggtaaaagAgataacccagggacctaatgagtctccctcagcctttttagagagactcaaggaggcct
Mpmv9         caatttggccaaggtaaaagggataacccagggacctaatgagtctccctcagcctttttagagagactcaaggaggcct

MPMV_CONS     ATCGCAGGTACACTCCTTATGACCCTGAGGACCCAGGGCAAGAAACCAATGTGTCTATGTCATTCATCTGGCAGTCTGCC
Mpmv1         atcgcaggtacactccttatgaccctgaggacccagggcaagaaaccaatgtgtctatgtcattcatctggcagtctgcc
Mpmv10        atcgcaggtacactccttatgaccctgaggacccagggcaagaaaccaatgtgtctatgtcattcatctggcagtctgcc
Mpmv11        atcgcaggtacactccttatgaccctgaggacccagggcaagaaaccaatgtgtctatgtcattcatctggcagtctgcc
Mpmv12        atcgcaggtacactccttatgaccctgaggacccagggcaagaaaccaatgtgtctatgtcattcatctggcagtctgcc
Mpmv13        atcgcaggtacactccttatgaccctgaggacccagggcaagaaaccaatgtgtctatgtcattcatctggcagtctgcc
Mpmv2         atcgcaggtacactccttatgaccctgaggacccagggcaagaaaccaatgtgtctatgtcattcatctggcagtctgcc
Mpmv3         atcgcaggtacactccttatgaccctgaggacccagggcaagaaaccaatgtgtctatgtcattcatctggcagtctgcc
Mpmv4         atcgcaggtacactccttatgaccctgaggacccagggcaagaaaccaatgtgtctatgtcattcatctggcagtctgcc
Mpmv5         atcgcaggtacactccttatgaccctgaggacccagggcaagaaaccaatgtgtctatgtcattcatctggcagtctgcc
Mpmv6         atcgcaggtacactccttatgaccctgaggacccagggcaagaaaccaatgtgtctatgtcattcatctggcagtctgcc
Mpmv7         atcgcaggtacactccttatgaccctgaggacccagggcaagaaaccaatgtgtctatgtcattcatctggcagtctgcc
Mpmv8         atcgcaggtacactccttatgaccctgaggacccagggcaagaaaccaatgtgtctatgtcattcatctggcagtctgcc
Mpmv9         atcgcaggtacactccttatgaccctgaggacccagggcaagaaaccaatgtgtctatgtcattcatctggcagtctgcc

MPMV_CONS     CCGGATATCGGGCGAAAGTTAGAGCGGTTAGAAGATTTAAAGAGCAAGACCTTAGGAGACTTAGTGAGGGAAGCTGAAAA
Mpmv1         ccggatatcgggcgaaagttagagcggttagaagatttaaagagcaagaccttaggagacttagtgagggaagctgaaaa
Mpmv10        ccggatatcgggcgaaagttagagcggttagaagatttaaagagcaagaccttaggagacttagtgagggaagctgaaaa
Mpmv11        ccggatatcgggcgaaagttagagcggttagaagatttaaagagcaagaccttaggagacttagtgagggaagctgaaaa
Mpmv12        ccggatatcgggcgaaagttagagcggttagaagatttTaagagcaagaccttaggagacttagtgagggaagctgaaaa
Mpmv13        ccggatatcgggcgaaagttagagcggttagaagatttaaagagcaagaccttaggagacttagtgagggaagctgaaaa
Mpmv2         ccggatatcgggcgaaagttagagcggttagaagatttaaagagcaagaccttaggagacttagtgagggaagctgaaaa
Mpmv3         ccggatatcgggcgaaagttagagcggttagaagatttaaagagcaagaccttaggagacttagtgagggaagctAaaaa
Mpmv4         ccggatatcgggcgaaagttagagcggttagaagatttaaagagcaagaccttaggagacttagtgagggaagctgaaaa
Mpmv5         ccggatatcgggcgaaagttagagcggttaAaagatttaaagagcaagaccttaggagacttagtgagggaagctAaaaa
Mpmv6         ccggatatcgggcgaaagttagagcggttagaagatttaaagagcaagaccttaggagacttagtgagggaagctgaaaa
Mpmv7         ccggatatcgggcgaaagttagagcggttaAaagatttaaagagcaagaccttaggagacttagtgagggaagctgaaaa
Mpmv8         ccggatatcgggcgaaagttagagcggttagaagatttaaagagcaagaccttaggagacttagtgagggaagctgaaaa
Mpmv9         ccggatatcgggcgaaagttagagcggttagaagatttaaagagcaagaccttaggagacttagtgagggaagctgaaaa
```

```
MPMV_CONS     GATCTTTAATAAACGAGAAACCCCGGAAGAAAGAGAGGAACGTATCAGGAGAGAAACAGAGGAAAAAGAAGAACGCCGTA
Mpmv1         gatctttaataaacgagaaaccccggaagaaagagaggaacgtatcaggagagaaacagaggaaaaagaagaacgccgta
Mpmv10        gatctttaataaacgagaaaccccggaagaaagagaggaacgtatcaggagagaaacagaggaaaaagaagaacgccgta
Mpmv11        gatctttaataaacgagaaaccccggaagaaagagaggaacgtatcaggagagaaacagaggaaaaagaagaacgccgta
Mpmv12        gatctttaataaacgagaaaccccggaagaaagagaggaacgtatcaggagagaaacagaggaaaaagaagaacgccgta
Mpmv13        gatctttaataaacgagaaaccccggaagaaagagaggaacgtatcaggagagaaacagaggaaaaagaaAaacgccgta
Mpmv2         gatctttaataGacgagaaaccccggaagaaagagaggaacgtatcaggagagaaacagaggaaaaagaagaacgccgta
Mpmv3         gatctttaataaacgagaaaccccggaagaaagagaggaacgtatcaggagagaaacagaggaaaaagaagaacgccgta
Mpmv4         gatctttaataaacgagaaaccccggaagaaagagaggaacgtatcaggagagaaacagaggaaaaagaagaacgccgta
Mpmv5         gatctttaataaacgagaaaccccggaagaaagagaggaacgtatcaggagagaaacagaggaaaaagaagaacgccgta
Mpmv6         gatctttaataaacgagaaaccccggaagaaagagaggaacgtatcaggagagaaacagaggaaaaagaagaacgccgta
Mpmv7         gatctttaataaacgagaaaccccggaagaaagagaggaacgtatcaggagagaaacagaggaaaGagaagaacgccgta
Mpmv8         gatctttaataaacgagaaaccccggaagaaagagaggaacgtatcaggagagaaacagaggaaaaagaagaacgccgta
Mpmv9         gatctttaataaacgagaaaccccggaagaaagagaggaacgtatcaggagagaaacagaggaaaaagaagaacgccgta

MPMV_CONS     GGGCAGAGGATGAGCAGAAAGAGAAAGAAAGGGACCGCAGAAGACATAGAGAGATGAGCAAGCTCTTGGCCACTGTAGTT
Mpmv1         gggcagaggatgagcagaaagagaaagaaagggaccgcagaagacatagagagatgagcaagctcttggccactgtagtt
Mpmv10        gggcagaggatgagcagaaagagaaagaaagggaccgcagaagacatagagagatgagcaagctcttggccactgtagtt
Mpmv11        gggcagaggatgagcaAaaagagaaagaaagggaccgcagaagacatagagagatgagcaagctcttggccactgtagtt
Mpmv12        gggcagaAgatgagcagaaagagaaagaaagggaccgcagaagacatagagagatgagcaagctcttggccactgtagtt
Mpmv13        gggcagaggatgagcagaaagagaaagaaagggaccgcagaagacatagagagatgagcaagctcttggccactgtagtt
Mpmv2         gggcagaggatgagcagaaagagaaagaaagggaccgcagaagacatagagagatgagcaagctcttggccactgtagtt
Mpmv3         gggcagaggatgagcagaaagagaaagaaagggaccgcagaagacatagagagatgagcaagctcttggccactgtagtt
Mpmv4         gggcagaggatgagcagaaagagaaagaaagggaccgcagaagacatagagagatgagcaagctcttggccactgtagtt
Mpmv5         gggcagaAgatgagcagaaagagaaagaaagggaccgcagaagacatagagagatgagcaagctcttggccactgtagtt
Mpmv6         gggcagaggatgagcagaaagagaaagaaagggaccgcagaagacatagagagatgagcaagctcttggccactgtagtt
Mpmv7         gggcagaggatgagcagaaagagaaagaaagggaccgcagaagacatagagagatgagcaagctcttggccactgtagtt
Mpmv8         gggcagaggatgagcagaaagagaaagaaagggaccgcagaagacatagagagatgagcaagctcttggccactgtagtt
Mpmv9         gggcagaggatgagcagaaagagaaagaaagggaccgcagaagacatagagagatgagcaagctcttggccactgtagtt

MPMV_CONS     ATTGGTCAGAGACAGGATAGACAGGGGGGAGAGCGGAAGAGGCCCCAACTTGATAAGGATCAATGCGCCTACTGCAAAGA
Mpmv1         attggtcagagacaggatagacaggggggagagcggaagaggccccaacttgataaggatcaatgcgcctactgcaaaga
Mpmv10        attggtcagagacaggatagacaggggggagagcggaagaggccccaacttgataaggatcaatgcgcctactgcaaaga
Mpmv11        attggtcagagacaggatagacaggggggagagcggaagaggccccaacttgataaAgatcaatgcgcctactgcaaaga
Mpmv12        attggtcagagacaggatagacaggggggagagcggaagaggccccaacttgataaggatcaatgcgcctactgcaaaga
Mpmv13        attggtcagagacaggatagacaAgggggagagcggaagaggccccaacttgataaggatcaatgcgcctactgcaaaga
Mpmv2         attggtcagagacaggatagacaggggggagagcggaagaggccccaacttgataaggatcaatgcgcctactgcaaaga
Mpmv3         attggtcagagacaggatagacaggggggagagcggaagaggccccaacttgataaggatcaatgcgcctactgcaaaga
Mpmv4         attggtcagagacaggatagacaggggggagagcggaagaggccccaacttgataaggatcaatgcgcctactgcaaaga
Mpmv5         attggtcagagacaggatagacaggggggagagcggaagaggccccaacttgataaggatcaatgcgcctactgcaaaga
Mpmv6         attggtcagagacaggatagacaggggggagagcggaagaggccccaacttgataaggatcaatgcgcctactgcaaaga
Mpmv7         attggtcagagacaggatagacaggggggagagcggaagaggccccaacttgataaggatcaatgcgcctactgcaaaga
Mpmv8         attggtcagagacaggatagacaggggggagagcggaagaggccccaacttgataaggatcaatgcgcctactgcaaaga
Mpmv9         attggtcagagacaggatagacaggggggagagcggaagaggccccaacttgataaggatcaatgcgcctactgcaaaga

MPMV_CONS     AAAGGGACACTGGGCTAAAGACTGCCCAAAGAAGCCACGAGGGCCCCGAGGACCGAGGCCCCAGACCTCCCTCCTGACCT
Mpmv1         aaagggacactgggctaaagactgcccaaagaagccacgagggccccgaggaccgaggccccagacctccctcctgacct
Mpmv10        aaagggacactgggctaaagactgcccaaagaagccacgagggccccgaggaccgaggccccagacctccctcctgacct
Mpmv11        aaagggacactgggctaaagactgcccaaagaagccacgagggccccgaggaccgaggccccagacctccctcctgacct
Mpmv12        aaagggacactgggctaaagactgcccaaagaagccacgagggccccgaggaccgaggccccagacctccctcctgacct
Mpmv13        aaagggacactgggctaaagactgcccaaagaagccacgagggccccgaggaccgaggccccagacctccctcctgacct
Mpmv2         aaagggacactgggctaaagactgcccaaagaagccacgagggccccgaggaccgaggccccagacctccctcctgacct
Mpmv3         aaagggacactgggctaaagactgcccaaagaagccacgagggccccgaggaccgaggccccagacctccctcctgacct
Mpmv4         aaagggacactgggctaaagactgcccaaagaagccacgagggccccgaggaccgaggccccagacctccctcctgacct
Mpmv5         aaagggacactgggctaaagactgcccaaagaagccacgagggccccgaggaccgaggccccagacctccctcctgacct
Mpmv6         aaagggacactgggctaaagactgcccaaagaagccacgagggccccgaggaccgaggccccagacctccctcctgacct
Mpmv7         aaagggacactgggctaaagactgcccaaagaagccacgagggccccgaggaccgaggccccagacctccctcctgacct
Mpmv8         aaagggacactgggctaaagactgcccaaagaagccacgagggccccgaggaccgaggccccagacctccctcctgacct
Mpmv9         aaagggacactgggctaaagactgcccaaagaagccacgagggccccgaggaccgaggccccagacctccctcctgacct
```

```
MPMV_CONS     TAGGTGACGCTCAGGTTGTGGGACCAATGGGACAGCCCCTGCAAGTGCTGACCCTAAACATAGAAGATGAGTATCGGCTA
Mpmv1         taggtgacgctcaggttAtgggaccaatgggacagcccctgcaagtgctgaccctaaacatagaagatgagtatcggcta
Mpmv10        taggtgac-ctcaggttgtgggaccaatgggacagccTctgcaagtgctgaccctaaacatagaagatgagtatcggcta
Mpmv11        taggtgacgctcaggttAtgggaccaatgggacagcccctgcaagtgctgaccctaaacatagaagatgagtatcggcta
Mpmv12        taggtgacgctcaggttgtgggaccaatgggacagcccctgcaagtgctgaccctaaacatagaagatgagtatcggcta
Mpmv13        taggtgacgctcaggttgtgggaccaatgggacagcccctgcaagtgctgaccctaaacatagaagatgagtatcggcta
Mpmv2         taggtgacgctcaggttgtgggaccaatgggacagcccctgcaagtgctgaccctaaacataAaagatgagtatcggcta
Mpmv3         taggtgacgctcaggttgtgggaccaatgggacagcccctgcaagtgctgaccctaaacatagaagatgagtatcggcta
Mpmv4         taggtgacgctcaggttAtgggaccaatgggacagcccctgcaagtgctgaccctaaacatagaagatgagtatcggcta
Mpmv5         taggtgacgctcaggttgtgggaccaatgggacagcccctgcaagtgctgaccctaaacatagaagatgagtatcggcta
Mpmv6         taggtgacgctcaggttgtgggaccaatgggacagcccctgcaagtgctgaccctaaacatagaagatgagtatcggcta
Mpmv7         taggtgacgctcaggttgtgggaccaatgggacagcccctgcaagtgctgaccctaaacatagaagatgagtatcggcta
Mpmv8         taggtgacgctcaggttAtgggaccaatgggacagcccctgcaagtgctgaccctaaacatagaagatgagtatcggcta
Mpmv9         taggtgacgctcaggttgtgggaccaatgggacagcccctgcaagtgctgaccctaaacatagaagatgagtatcggcta

MPMV_CONS     CATGAGACCTCAAAAGAGCCGGATGTTTCTCTAGGGTCCACATGGCTTTCTGATTTTCCCCAGGCCTGGGCGGAAACCGG
Mpmv1         catgagaccCcaaaagagccggatgtttctctagggtccacatggctttctgattttccccaggcctgggcggaaaccgg
Mpmv10        catgagacctcaaaagagccggatgtttctctagggtccacatggctttctgattttccccaggcctgggcggaaaccgg
Mpmv11        catgagacctcaaaagagccggatgtttctctagggtccacatggctttctgattttccccaggcctgggcggaaaccgg
Mpmv12        catgagacctcaaaagagccggatgtttctctagggtccacatggctttctgattttccccaggcctgggcggaaaccgg
Mpmv13        catgagacctcaaaagagccggatgtttctctagggtccacatggctttctgattttccccaggcctgggcggaaaccgg
Mpmv2         catgagacctcaaaagagccggatgtttctctagggtccacatggctttctgattttccccaggcctgggcggaaaccgg
Mpmv3         catgagacctcaaaagagccggatgtttctctagggtccacatggctttctgattttccccaggcctgggcggaaaccgg
Mpmv4         catgagaccCcaaaagagccggatgtttctctagggtccacatggctttctgattttccccaggcctgggcggaaaccgg
Mpmv5         catgagacctcaaaagagccggatgtttctctagggtccacatggctttctgattttccccaggcctgggcAAaaaccgg
Mpmv6         catgagacctcaaaagagccggatgtttctctagggtccacatggctttctgattttccccaggcctgggcggaaaccgg
Mpmv7         catgagacctcaaaagagccggatgtttctctagggtccacatggctttctgattttccccaggcctgggcggaaaccgg
Mpmv8         catgagaccCcaaaagagccggatgtttctctagggtccacatggctttctgattttccccaggcctgggcggaaaccgg
Mpmv9         catgagacctcaaaagagccggatgtttctctagggtccacatggctttctgattttccccaggcctgggcggaaaccgg

MPMV_CONS     GGGCATGGGACTGGCAGTTCGCCAAGCTCCTCTGATCATACCTCTGAAGGCAACCTCTACCCCCGTGTCCATAAAACAAT
Mpmv1         gggcatgggactggcagttcgccaagctcctctgatcatacctctgaaggcaacctctacccccgtgtccataaaacaat
Mpmv10        gggcatgggactggcagttcgccaagctcctctgatcatacctctgaaggcaacctctacccccgtgtccataaaa----
Mpmv11        gggcatgggactggcagttcgccaagctcctctgatcatacctctgaaggcaacctctacccccgtgtccataaaacaat
Mpmv12        gggcatgggactggcagttcgccaagctcctctgatcatacctctgaaggcaacctctacccccgtgtccataaaacaat
Mpmv13        gggcatgggactggcagttcgccaagctcctctgatcatacctctgaaggcaacctctacccccgtgtccataaaacaat
Mpmv2         gggcatgggactggcagttcgccaagctcctctgatcatacctctgaaggcaacctctacccccgtgtccataaaacaat
Mpmv3         gggcatgggactggcagttcgccaagctcctctgatcatacctctgaaggcaacctctacccccgtgtccataaaacaat
Mpmv4         gggcatgggactggcagttcgccaagctcctctgatcatacctctgaaggcaacctctacccccgtgtccataaaacaat
Mpmv5         gggcatgggactggcagttcgccaagctcctctgatTatacctctgaaggcaacctctacccccgtgtccataaaacaat
Mpmv6         gggcatgggactggcagttcgccaagctcctctgatcatacctctgaaggcaacctctacccccgtgtccataaaacaat
Mpmv7         gggcatgggactggcagttcgccaagctcctctgatcatacctctgaaggcaacctctacccccgtgtccataaaacaat
Mpmv8         gggcatgggactggcagttcgccaagctcctctgatcatacctctgaaggcaacctctacccccgtgtccataaaacaat
Mpmv9         gggcatgggactggcagttcgccaagctcctctgatcatacctctgaaggcaacctctacccccgtgtccataaaacaat

MPMV_CONS     ACCCCATGTCACAAGAAGCCAGACTGGGGATCAAGCCCCACATACAGAGACTGTTGGACCAGGGAATACTGGTACCCTGC
Mpmv1         accccatgtcacaagaagccagactggggatcaagTcccacatacagagactgttggaccagggaatactggtaccctgc
Mpmv10        --------------------------------------------------------------------------------
Mpmv11        accccatgtcacaagaagccagactggggatcaagccccacatacagagactgttggaccagggaatactggtaccctgc
Mpmv12        accccatgtcacaagaagccagactggggatcaagccccacatacagagactgttggaccagggaatactggtaccctgc
Mpmv13        accccatgtcacaagaagccagactggggatcaagccccacatacagagactgttggaccagggaatactggtaccctgc
Mpmv2         accccatgtcacaagaagccagactggggatcaagccccacatacagagactgttggaccagggaatactggtaccctgc
Mpmv3         accccatgtcacaagaagccagactggggatcaagccccacatacagagactgttggaccagggaatactggtaccctgc
Mpmv4         accccatgtcacaagaagccagactggggatcaagTcccacatacagagactgttggaccagggaatactggtaccctgc
Mpmv5         accccatgtcacaagaagccagactggggatcaagccccacatacagagactgttggaccagggaatactggtaccctgc
Mpmv6         accccatgtcacaagaagccagactAgggatcaagccccacatacagagactgttggaccagggaatactggtaccctgc
Mpmv7         accccatgtcacaagaagccagactggggatcaagccccacatacagagactgttggaccagggaatactggtaccctgc
Mpmv8         accccatgtcacaagaagccagactggggatcaagTcccacatacagagactgttAgaccagggaatactggtaccctgc
Mpmv9         accccatgtcacaagaagccagactggggatcaagccccacatacagagactgttggaccagggaatactggtaccctgc
```

```
MPMV_CONS     CAGTCCCCCTGGAACACGCCCCTGCTACCCGTTAAGAAACCAGGGACTAATGATTACAGGCCTGTCCAAGATCTGAGAGA
Mpmv1         cagtccccctggaacacgcccctgctacccgttaagaaaccagggactaatgattacaggcctgtccaagatctgagaga
Mpmv10        --------------------------------------------------------------------------------
Mpmv11        cagtccccctggaacaTgcccctgctacccgttaagaaaccagggactaatgattacaggcctgtccaagatctgagaga
Mpmv12        cagtccccctggaacacgcccctgctacccgttaagaaaccagggactaatgattacaggcctgtccaagatctgagaga
Mpmv13        cagtccccctggaacacgcccctgctacccgttaagaaaccagggactaatgattacaggcctgtccaagatctgagaga
Mpmv2         cagtccccctggaacacgcccctgctacccgttaagaaaccagggactaatgattacaggcctgtccaagatctgagaga
Mpmv3         cagtccccctggaacacgcccctgctacccgttaagaaaccagggactaatgattacaggcctgtccaagatctgagaga
Mpmv4         cagtccccctggaacacgcccctgctacccgttaagaaaccagggactaatgattacaggcctgtccaagatctgagaga
Mpmv5         cagtccccctggaacacgcccctgctacccgttaagaaaccagggactaatgattacaggcctgtccaagatctgagaAa
Mpmv6         cagtccccctggaacacgcccctgctacccgttaagaaaccagggactaatgattacaggcctgtccaagatctgagaga
Mpmv7         cagtccccctggaacacgcccctgctacccgttaagaaaccagggactaatgattacaggcctgtccaagatctgagaga
Mpmv8         cagtccccctggaacacgcccctgctacccgttaagaaaccagggactaatgattacaggcctgtccaagatctgagaga
Mpmv9         cagtccccctggaacacgcccctgctacccgttaagaaaccagggactaatgattacaggcctgtccaagatctgagaga

MPMV_CONS     AGTCAACAAGCGGGTGGAAGACATCCACCCCACCGTGCCCAACCCTTACAACCTCTTGAGCGGGCTCCCACCGTCCCACC
Mpmv1         agtcaacaagcgggtggaagacatccaccccaccgtgcccaacccttacaacctcttgagcgggctcccaccgtcccacc
Mpmv10        --------------------------------------------------------------------------------
Mpmv11        agtcaacaagcgggtggaagacatccaccccaccgtgcccaacccttacaacctcttgagcgggctcccaccgtcccacc
Mpmv12        agtcaacaagcgggtggaagacatccaccccaccgtgcccaacccttacaacctcttgagcgggctcccaccgtcccacc
Mpmv13        agtcaacaagcgggtggaagacatccaccccaccgtgcccaacccttacaacctcttgagcgggctcccaccgtcccacc
Mpmv2         agtcaacaagcgggtggaagacatccaccccaccgtgcccaacccttacaacctcttgagcgggctcccaccgtcccacc
Mpmv3         agtcaacaagcgggtggaagacatccaccccaccgtgcccaacccttacaacctcttgagcgggctcccaccgtcccacc
Mpmv4         agtcaacaagcgggtggaagacatccaccccaccgtgcccaacccttacaacctcttgagcgggctcccaccgtcccacc
Mpmv5         agtcaacaagcgggtggaagacatccaccccaccgtgcccaacccttacaacctcttgagcgggctcccaccgtcccacc
Mpmv6         agtcaacaagcgggtggaagacatccaccccaccgtgcccaacccttacaacctcttgagcgggctcccaccgtcccacc
Mpmv7         agtcaacaagcgggtggaagacatccaccccaccgtgcccaacccttacaacctcttgagcgggctcccaccgtcccacc
Mpmv8         agtcaacaagcgggtggaagacatccaccccaccgtgcccaacccttacaacctcttgagcgggctcccaccgtcccacc
Mpmv9         agtcaacaagcgggtggaagacatccaccccaccgtgcccaacccttacaacctcttgagcgggctcccaccgtcccacc

MPMV_CONS     AGTGGTACACTGTGCTTGACTTAAAGGATGCCTTTTTCTGCCTGAGACTCCACCCCACCAGTCAGCCTCTCTTCGCCTTT
Mpmv1         agtggtacactgtgcttgacttaaaggatgcctttttctgcctgagactccaccccaccagtcagcctctcttcgccttt
Mpmv10        --------------------------------------------------------------------------------
Mpmv11        agtggtacactgtgcttgacttaaaggatgcctttttctgcctAagactccaccccaccagtcagcctctcttcgccttt
Mpmv12        agtggtacactgtgcttgacttaaaggatgcctttttctgcctgagactccaccccaccagtcagcctctcttcgccttt
Mpmv13        agtggtacactgtgcttgacttaaaggatgcctttttctgcctgagactccaccccaccagtcagcctctcttcgccttt
Mpmv2         agtggtacactgtgcttgacttaaaggatgcctttttctgcctgagactccaccccaccagtcagcctctcttcgccttt
Mpmv3         agtggtacactgtgcttgacttaaaggatgcctttttctgcctgagactccaccccaccagtcagcctctcttcgccttt
Mpmv4         agtggtacactgtgcttgacttaaaggatgcctttttctgcctgagactccaccccaccagtcagcctctcttcgccttt
Mpmv5         agtggtacactgtgcttgacttaaaggatgcctttttctgcctgagactccaccccaccagtcagcctctcttcgccttt
Mpmv6         agtggtacactgtgcttgacttaaaggatgcctttttctgcctgagactccaccccaccagtcagcctctcttcgccttt
Mpmv7         agtggtacactgtgcttgacttaaaggatgcctttttctgcctAagactccaccccaccagtcagcctctcttcgccttt
Mpmv8         agtggtacactgtgcttgacttaaaggatgcctttttctgcctgagactccaccccaccagtcagcctctcttcgccttt
Mpmv9         agtggtacactgtgcttgacttaaaggatgcctttttctgcctgagactccaccccaccagtcagcctctcttcgccttt

MPMV_CONS     GAGTGGAGAGACCCAGAGATGGGAATCTCAGGACAATTAACCTGGACCAGACTCCCACAGGGTTTCAAAAACAGTCCCAC
Mpmv1         gagtggagagacccagagatgggaatctcaggacaattaacctggaccagactcccacagggtttcaaaaacagtcccac
Mpmv10        --------------------------------------------------------------------------------
Mpmv11        gagtggagagacccagagatgggaatctcaggacaattaacctggaccagactcccacagggtttcaaaaacagtcccac
Mpmv12        gagtggagagacccagagatgggaatctcaggacaattaacctggaccagactcccacagggtttcaaaaacagtcccac
Mpmv13        gagtggagagacccagagatgggaatctcaggacaattaacctggaccagactcccacagggtttcaaaaacagtcccac
Mpmv2         gagtggagagacccagagatgggaatctcaggacaattaacctggaccagactcccacagggtttcaaaaacagtcccac
Mpmv3         gagtggagagacccagagatgggaatctcaggacaattaacctggaccagactcccacagggtttcaaaaacagtcccac
Mpmv4         gagtggagagacccagagatgggaatctcaggacaattaacctggaccagactcccacagggtttcaaaaacagtcccac
Mpmv5         gagtAgagagacccagagatgggaatctcaggacaattaacctggaccagactcccacagggtttcaaaaacagtcccac
Mpmv6         gagtggagagacccagagatgggaatctcaggacaattaacctggaccagactcccacagggtttcaaaaacagtcccac
Mpmv7         gagtggagagacccagagatgggaatctcaggacaattaacctggaccagactcccacagggtttcaaaaacagtcccac
Mpmv8         gagtggagagacccagagatgggaatctcaggacaattaacctggaccagactcccacagggtttcaaaaacagtcccac
Mpmv9         gagtggagagacccagagatgggaatctcaggacaattaacctggaccagactcccacagggtttcaaaaacagtcccac
```

```
MPMV_CONS     CCTGTTTGATGAGGCACTGCACAGAGACCTAGCAGGCTTCCGGATCCAGCACCCAGACTTGATCCTGCTACAGTACGTGG
Mpmv1         cctgtttgatgaggcactgcacagagacctagcaggcttccggatccagcacccagacttgatcctgctacagtacgtgg
Mpmv10        --------------------------------------------------------------------------------
Mpmv11        cctgtttgatgaggcactgcacagagacctagcaggcttccggatccagcacccagacttgatcctgctacagtacgtgg
Mpmv12        cctgtttgatgaggcactgcacagagacctagcaggcttccggatccagcacccagacttgatcctgctacagtacgtgg
Mpmv13        cctgtttgatgaggcactgcacagagacctagcaggcttccggatccagcacccagacttgatcctgctacagtacgtgg
Mpmv2         cctgtttgatgaggcactgcacagagacctagcaggcttccggatccagcacccagacttgatcctgctacagtacgtgg
Mpmv3         cctgtttgatgaggcactgcacagagacctagcaggcttccggatccagcacccagacttgatcctgctacagtacgtgg
Mpmv4         cctgtttgatgaggcactgcacagagacctagcaggcttccggatccagcacccagacttgatcctgctacagtacgtgg
Mpmv5         cctgtttAatgaggcactgcacagagacctagcaggcttccggatccagcacccagacttgatcctgctacagtacgtgg
Mpmv6         cctgtttAatgaggcactgcacagagacctagcaggcttccggatccagcacccagacttgatcctgctacagtacgtgg
Mpmv7         cctgtttgatgaggcactgcacagagacctagcaggcttccggatccagcacccagacttgatcctgctacagtacgtgg
Mpmv8         cctgtttgatgaggcactgcacagagacctagcaggcttccggatccagcacccagacttgatcctgctacagtacgtgg
Mpmv9         cctgtttgatgaggcactgcacagagacctagcaggcttccggatccagcacccagacttgatcctgctacagtacgtgg

MPMV_CONS     ATGACTTACTGCTGGCCGCCACTTCTGAGCTCGACTGCCAACAAGGTACTCGGGCCCTGTTACAAACCCTAGGGGACCTC
Mpmv1         atgaTttactgctggccgccacttctgagctcgactgccaacaaggtactcgggccctgttacaaaccctaggggacctc
Mpmv10        --------------------------------------------------------------------------------
Mpmv11        atgacttactgctggccgccacttctgagctcgactgccaacaaggtactcgggccctgttacaaaccTtaggggacctc
Mpmv12        atgacttactgctggccgccacttctgagctcgactgccaacaaggtactcgggccctgttacaaaccctaggggacctc
Mpmv13        atgacttactgctggccgccacttctgagctcgactgccaacaaggtactcgggccctgttacaaaccctaggggacctc
Mpmv2         atgacttactgctggccgccacttctgagctcgactgccaacaaggtactcgggccctgttacaaaccctaggggacctc
Mpmv3         atgacttactgctggccgccacttctgagctcgactgccaacaaggtactcgggccctgttacaaaccctaggggacctc
Mpmv4         atgaTttactgctggccgccacttctgagctcgactgccaacaaggtactcgggccctgttacaaaccctaggggacctc
Mpmv5         atgacttactgctggccgccacttctgagctcgactgccaacaaggtactcgggccctgttacaaaccctaggggacctc
Mpmv6         atgacttactgctggccgccacttctgagctcgactgccaacaaggtactcgggccctgttacaaaccctaggggacctc
Mpmv7         atgacttactgctggccgccacttctgagctcgactgccaacaaggtactcgggccctgttacaaaccctaggggacctc
Mpmv8         atgaTttactgctggccgccacttctgagctcgactgccaacaaggtactcgggccctgttacaaaccctaggggacctc
Mpmv9         atgacttactgctggccgccacttctgagctcgactgccaacaaggtactcgggccctgttacaaaccctaggggacctc

MPMV_CONS     GGGTATCGGGCCTCGGCCAAGAAAGCCCAAATTTGCCAGAAACAGGTCAAGTATCTGGGGTATCTTCTAAAAGAGGGTCA
Mpmv1         gggtatcgggcctcggccaagaaagcccaaatttgccagaaacaggtcaagtatctggggtatcttctaaaagagggtca
Mpmv10        --------------------------------------------------------------------------------
Mpmv11        gggtatcgggcctcggccaagaaagcccaaatttgccagaaacaggtcaagtatctggggtatcttctaaaagagggtca
Mpmv12        gggtatcgggcctcggccaagaaagcccaaatttgccagaaacaggtcaagtatctggggtatcttctaaaagagggtca
Mpmv13        gggtatcgggcctcggccaagaaagcccaaatttgccagaaacaggtcaagtatctggggtatcttctaaaagagggtca
Mpmv2         gggtatcgggcctcggccaagaaagcccaaatttgccagaaacaggtcaagtatctggggtatcttctaaaagagggtca
Mpmv3         gggtatcgggcctcggccaagaaagcccaaatttgccagaaacaggtcaagtatctggggtatcttctaaaagagggtca
Mpmv4         gggtatcgggcctcggccaagaaagcccaaatttgccagaaacaggtcaagtatctggggtatcttctaaaagagggtca
Mpmv5         gggtatcgggcctcggccaagaaagcccaaatttgccagaaacaggtcaagtatctggggtatcttctaaaagagggtca
Mpmv6         gggtatcgggcctcggccaagaaagcccaaatttgccagaaacaggtcaagtatctggggtatctCctaaaagagggtca
Mpmv7         gggtatcgggcctcggccaagaaagcccaaatttgccagaaacaggtcaagtatctggggtatcttctaaaagagggtca
Mpmv8         gggtatcgggcctcggccaagaaagcccaaatttgccagaaacaggtcaagtatctggggtatcttctaaaagagggtca
Mpmv9         gggtatcgggcctcggccaagaaagcccaaatttgccagaaacaggtcaagtatctggggtatcttctaaaagagggtca

MPMV_CONS     GAGATGGCTGACTGAGGCCAGAAAAGAGACTGTGATGGGGCAGCCTATTCCGAAGACCCCTCGACAACTAAGGGAGTTCC
Mpmv1         gagatggctgactgaggccagaaaagagactgtgatggggcagcctattccgaagacccctcgacaactaagggagttcc
Mpmv10        --------------------------------------------------------------------------------
Mpmv11        gagatggctgactgaggccagaaaagagactgtgatggggcagcctattccgaagacccctcgacaactaagggagttcc
Mpmv12        gagatggctgactgaggccagaaaagagactgtgatggggcagcctattccgaagacccctcgacaactaagggagttcc
Mpmv13        gagatggctgactgaggccagaaaagagactgtgatggggcagcctattccgaagacccctcgacaactaagggagttcc
Mpmv2         gagatggctgactgaggccagaaaagagactgtgatggggcagcctattccgaagacccctcgacaactaagggagttcc
Mpmv3         gagatggctgactgaggccagaaaagagactgtgatggggcagcctattccgaagacccctcgacaactaagggagttcc
Mpmv4         gagatggctgactgaggccagaaaagagactgtgatggggcagcctattccgaagacccctcgacaactaagggagttcc
Mpmv5         gagatggctgactgaggccagaaaagagactgtgatggggcagcctaCtccgaagacccctcgacaactaagggagttcc
Mpmv6         gagatggctgactgaggccagaaaagagactgtgatggggcagcctaCtccgaagacccctcgacaactaagggagttcc
Mpmv7         gagatggctgactgaggccagaaaagagactgtgatggggcTgcctattccgaagacccctcgacaactaagAgagttcc
Mpmv8         gagatggctgactgaggccagaaaagagactgtgatggggcagcctattccgaagacccctcgacaactaagggagttcc
Mpmv9         gagatggctgactgaggccagaaaagagactgtgatggggcagcctattccgaagacccctcgacaactaagggagttcc
```

```
MPMV_CONS     TAGGGACGGCAGGCTTCTGTCGCCTCTGGATCCCTGGGTTTGCAGAAATGGCAGCCCCCTTGTACCCTCTCACCAAAACG
Mpmv1         tagggacggcaggcttctgtcgcctctggatccctgggtttgcagaaatggcCgcccccttgtaccctctcaccaaaacg
Mpmv10        --------------------------------------------------------------------------------
Mpmv11        tagggacggcaggcttctgtcgcctctggatccctgggtttgcagaaatggcagcccccttgtaccctctcaccaaaacg
Mpmv12        tagggacggcaggcttctgtcgcctctggatccctgggtttgcagaaatggcagcccccttgtaTcctctcaccaaaacg
Mpmv13        tagggacggcaggcttctgtcgcctctggatccctgggtttgcagaaatggcagcccccttgtaccctctcaccaaaacg
Mpmv2         tagggacggcaggcttctgtcgcctctggatccctgggtttgcagaaatggcagcccccttgtaTcctctcaccaaaacg
Mpmv3         tagggacggcaggcttctgtcgcctctggatccctgggtttgcagaaatggcagcccccttgtaccctctcaccaaaacg
Mpmv4         tagggacggcaggcttctgtcgcctctggatccctgggtttgcagaaatggcCgcccccttgtaccctctcaccaaaacg
Mpmv5         tagggacggcaggcttctgtcgcctctggatccctgggtttgcagaaatggcagcccccttgtaccctctcaccaaaacg
Mpmv6         tagggacggcaggcttctgtcgcctctggatccctggAtttgcagaaatggcagcccccttgtaccctctcaccaaaacg
Mpmv7         tagggacggcaggcttctgtcgcctctggatccctgggtttgcagaaatggcagcccccttgtaccctctcaccaaaacg
Mpmv8         tagggacggcaggcttctgtcgcctctggatccctgggtttgcagaaatggcCgcccccttgtaccctctcaccaaaacg
Mpmv9         tagggacggcaggcttctgtcgcctctggatccctgggtttgcagaaatggcagcccccttgtaTcctctcaccaaaacg

MPMV_CONS     GGGACTCTGTTTAATTGGGGCCCAGACCAGCAAAAGGCCTATCAAGAAATCAAACAGGCTCTTCTAACTGCCCCAGCCCT
Mpmv1         gggactctgtttaattggggcccagaccagcaaaaggcctatcaagaaatcaaacaggctcttctaactgccccagccct
Mpmv10        --------------------------------------------------------------------------------
Mpmv11        gggactctgtttaattggggcccagaccagcaaaaggcctatcaagaaatcaaacaggctcttctaactgccccagccct
Mpmv12        gggactctgtttaattggggcccagaccagcaaaaggcctatcaagaaatcaaacaggctcttctaactgccccagccct
Mpmv13        gggactctgtttaattggggcccagaccagcaaaaggcctatcaagaaatcaaacaggctcttctaactgccccagccct
Mpmv2         gggactctgtttaattggggcccagaccagcaaaaggcctatcaagaaatcaaacaggctcttctaactgccccagccct
Mpmv3         gggactctgtttaattggggcccagaccagcaaaaggcctatcaagaaatcaaacaggctcttctaactgccccagccct
Mpmv4         gggactctgtttaattggggcccagaccagcaaaaggcctatcaagaaatcaaacaggctcttctaactgccccagccct
Mpmv5         gggactctgtttaattggggcccagaccagcaaaaggcctatcaaAaaatcaaacaggctcttctaactgccccagccct
Mpmv6         gggactctgtttaattggggcccagaccagcaaaaggcctatcaagaaatcaaacaggctcttctaactgccccagccct
Mpmv7         gggactctgtttaattggggcccagaccagcaaaaggcctatcaagaaatcaaacaggctcttctaactgccccagccct
Mpmv8         gggactctgtttaattggggcccagaccagcaaaaggcctatcaagaaatcaaacaggctcttctaactgccccagccct
Mpmv9         gggactctgtttaattggggcccagaccagcaaaaggcctatcaagaaatcaaacaggctcttctaactgccccagccct

MPMV_CONS     GGGGTTGCCAGATTTGACTAAGCCCTTTGAACTCTTTGTCGACGAGAAGCAGGGCTACGCCAAAGGCGTCCTAACGCAAA
Mpmv1         ggggttgccagatttgactaagccctttgaactctttgtcgacgagaagcagggctacgccaaaggcgtcctaacgcaaa
Mpmv10        --------------------------------------------------------------------------------
Mpmv11        ggggttgccagatttgactaagccctttgaactctttgtcgacgagaagcagggctacgccaaaggcgtcctaacgcaaa
Mpmv12        ggggttgccagatttgactaagccctttgaactctttgtcgacgagaagcagggctacgccaaaggcgtcctaacgcaaa
Mpmv13        ggggttgccagatttgactaagccctttgaactctttgtcgacgaAaagcagggctacgccaaaggcgtcctaacgcaaa
Mpmv2         ggggttgccagatttgactaagccctttgaactctttgtcgacgagaagcagggctacgccaaaggcgtcctaacgcaaa
Mpmv3         ggggttgccagatttgactaagccctttgaactctttgtcgacgagaagcagggctacgccaaaggcgtcctaacgcaaa
Mpmv4         ggggttgccagatttgactaagccctttgaactctttgtcgacgagaagcagggctacgccaaaggcgtcctaacgcaaa
Mpmv5         gAgAttgccagatttgactaagccctttgaactctttgtcgacgagaagcagggctacgccaaaggcgtcctaacgcaaa
Mpmv6         gggAttgccagatttgactaagccctttgaactctttgtcgacgagaagcagggctacgccaaaggcgtcctaacgcaaa
Mpmv7         ggggttgccagatttgactaagccctttgaactctttgtcgacgagaagcagggctacgccaaaggcgtcctaacgcaaa
Mpmv8         ggggttgccagatttgactaagccctttgaactctttgtcgacgagaagcagggctacgccaaaggcgtcctaacgcaaa
Mpmv9         ggggttgccagatttgactaagccctttgaactctttgtcgacgagaagcagggctacgccaaaggcgtcctaacgcaaa

MPMV_CONS     AACTGGGACCTTGGCGTCGGCCGGTGGCCTACCTGTCCAAAAAGCTAGACCCAGTGGCAGCTGGGTGGCCCCCTTGCCTA
Mpmv1         aactgggaccttggcgtcggccggtggcctacctgtccaaaaagctagacccagtggcagctgggtggcccccttgccta
Mpmv10        --------------------------------------------------------------------------------
Mpmv11        aactgggaccttggcgtcggccggtggcctacctgtccaaaaagctagacccagtggcagctgggtggcccccttgccta
Mpmv12        aactgggaccttggcgtcggccggtggcctacctgtccaaaaagctagacccagtggcagctgggtggcccccttgccta
Mpmv13        aactgggaccttggcgtcggccggtggcctacctgtccaaaaagctagacccagtggcagctgggtggcccccttgccta
Mpmv2         aactgggaccttggcgtcggccggtggcctacctgtccaaaaagctagacccagtggcagctgggtggcccccttgccta
Mpmv3         aactgggaccttggcgtcggccggtggcctacctgtccaaaaagctagacccagtggcagctgggtggcccccttgccta
Mpmv4         aactgggaccttggcgtcggccggtggcctacctgtccaaaaagctagacccagtggcagctgggtggcccccttgccta
Mpmv5         aactgggaccttggcgtcggccggtggcctacctgtccaaaaagctagacccagtggcagctgggtggcccccttgccta
Mpmv6         aactgggaccttggcgtcggccggtggcctacctgtccaaaaagctagacccagtggcagctgggtggcccccttgccta
Mpmv7         aactgggaccttggcgtcggccggtggcctacctgtccaaaaagctagacccagtggcagctgggtggcccccttgccta
Mpmv8         aactgggaccttggcgtcggccggtggcctacctgtccaaaaagctagacccagtggcagctgggtggcccccttgccta
Mpmv9         aactgggaccttggcgtcggccggtggcctacctgtccaaaaagctagacccagtggcagctgggtggcccccttgccta
```

```
MPMV_CONS     CGGATGGTAGCAGCCATTGCCGTTCTGACAAAGGATGCAGGCAAGCTAACTATGGGACAGCCGCTAGTCATTCTGGCCCC
Mpmv1         cggatggtagcagccattgccgttctgacaaaggatgcaggcaagctaactatgggacagccgctagtcattctggcccc
Mpmv10        --------------------------------------------------------------------------------
Mpmv11        cgAatggtagcagccattgccgttctgacaaaggatgcaggcaagctaactatgggacagccgctagtcattctggcccc
Mpmv12        cggatggtagcagccattgccgttctgacaaaggatgcaggcaagctaactatgggacagccgctagtcattctggcccc
Mpmv13        cggatggtagcagccattgccgttctgacaaaggatgcaggcaagctaactatgggacagccgctagtcattctggcccc
Mpmv2         cggatggtagcagccattgccgttctgacaaaggatgcaggcaagctaactatgggacagccgctagtcattctggcccc
Mpmv3         cggatggtagcagTcattgccgttctgacaaaggatgcaggcaagctaactatgggacagccgctagtcattctggcccc
Mpmv4         cggatggtagcagccattgccgttctgacaaaggatgcaggcaagctaactatgggacagccgctagtcattctggcccc
Mpmv5         cggatggtagcagccattgccgttctgacaaaggatgcaggcaaActaactatgggacagccgctagtcattctggcccc
Mpmv6         cggatggtagcagccattgccgttctgacaaaggatgcaggcaagctaactatgggacagccgctagtcattctggcccc
Mpmv7         cggatggtagcagccattgccgttctgacaaaggatgcaggcaagctaactatgggacagccgctagtcattctggcccc
Mpmv8         cggatggtagcagccattgccgttctgacaaaggatgcaggcaagctaactatgggacagccgctagtcattctggcccc
Mpmv9         cggatggtagcagccattgccgttctgacaaaggatgcaggcaagctaactatgggacagccgctagtcattctggcccc

MPMV_CONS     CCATGCGGTAGAAGCACTGGTCAAACAACCCCCTGACCGCTGGCTATCCAATGCTCGCATGACCCACTATCAGGCAATGC
Mpmv1         ccatgcggtagaagcactggtcaaacaaccccctgaccgctggctatccaatgctcgcatgacccactatcaggcaatgc
Mpmv10        --------------------------------------------------------------------------------
Mpmv11        ccatgcggtagaagcactggtcaaacaaccccctgaccgctggctatccaatgctcgcatgacccactatcaggcaatgc
Mpmv12        ccatgcggtagaagcactggtcaaacaaccccctgaccgctggctatccaatgctcgcatgacccactatcaggcaatgc
Mpmv13        ccatgcggtaAaagcactggtcaaacaaccccctgaccgctggctatccaatgctcgcatgacccactatcaggcaatgc
Mpmv2         ccatgcggtagaagcactggtcaaacaaccccctgaccgctggctatccaatgctcgcatgacccactatcaggcaatgc
Mpmv3         ccatgcggtagaagcactggtcaaacaaccccctgaccgctggctatccaatgctcgcatgacccactatcaggcaatgc
Mpmv4         ccatgcggtagaagcactggtcaaacaaccccctgaccgctggctatccaatgctcgcatgacccactatcaggcaatgc
Mpmv5         ccatgcggtagaagcactggtcaaacaaccccctgaccgctggctatccaatgctcgcatgacccactatcaggcaatgc
Mpmv6         ccatgcggtagaagcactggtcaaacaaccccctgaccgctggctatccaatgctcgcatgacTcactatcaggcaatgc
Mpmv7         ccatgcggtagaagcactggtcaaacaaccccctgaccgctggctatccaatgctcgcatgacccactatcaggcaatgc
Mpmv8         ccatgcggtagaagcactggtcaaacaaccccctgaccgctggctatccaatgctcgcatgacccactatcaggcaatgc
Mpmv9         ccatgcggtagaagcactggtcaaacaaccccctgaccgctggctatccaatgctcgcatgacccactatcaggcaatgc

MPMV_CONS     TCCTAGATACGGACCGAGTTCAGTTCGGACCGGTGGTAGCCTTAAACCCGGCCACGTTGCTCCCCTTGCCGGGAAAAGAG
Mpmv1         tcctagatacggaccgagttcagttcggaccggtggtagccttaaacccggccacgttgctccccttgccgggaaaagag
Mpmv10        ------atacggaccgagttcagttcggaccggtggtagccttaaacccggccacgttgctccccttgccgggaaaagag
Mpmv11        tcctagatacggaccgagttcagttcggaccggtggtagccttaaacccggccacgttgctccccttgccgggaaaagag
Mpmv12        tcctagatacggaccgagttcagttcggaccggtggtagccttaaacccggccacgttgctccccttgccgggaaaagag
Mpmv13        tcctagatacggaccgagttcagttcggacTggtggtagccttaaacccggccacgttgctccccttgccgggaaaagag
Mpmv2         tcctagatacggaccgagttcagttcggaccggtggtagccttaaacccggccacgttgctccccttgccgggaaaagag
Mpmv3         tcctagatacggaccgagttcagttcggaccggtggtagccttaaacccggccacgttgctccccttgccgggaaaagag
Mpmv4         tcctagatacggaccGgttcagttcggaccggtggtagccttaaacccggccacgttgctccccttgccgggaaaagag
Mpmv5         tcctagatacggaccgagttcagttcggaccggtggtagccttaaacccggccacgttgctccccttgccgggaaaagag
Mpmv6         tcctagatacggaccgagttcagttcggaccggtggtagccttaaacccggccacgttgctccccttgccgggGaaagag
Mpmv7         tcctagatacggaccgagttcagttcggaccggtggtagccttaaacccggccacgttgctccccttgccgggaaaagag
Mpmv8         tcctagatacggaccGgttcagttcggaccggtggtagccttaaacccggccacgttgctccccttgccgggaaaagag
Mpmv9         tcctagatacggaccgagttcagttcggaccggtggtagccttaaacccggccacgttgctccccttgccgggaaaagag

MPMV_CONS     ACCCCCCACGACTGCCTCGAGATCTTGGCTGAGACACACGGCACCAGACCGGACCTCACGGACCAGCCCCTCCCAAATGC
Mpmv1         accccccacgactgcctcgagatcttggctgagacacacggcaccagaccggacctcacggaccagcccctcccaaatgc
Mpmv10        accccccacgactgcctcgagatcCtggctgagacacacggcaccagaccggacctcacggaccagcccctcccaaatgc
Mpmv11        accccccacgactgcctcgagatcttggctgagacacacggcaccagaccggacctcacggaccagcccctcccaaatgc
Mpmv12        accccccacgactgcctcgagatcttggctgagacacacggcaccagaccggacctcacggaccagcccctcccaaatgc
Mpmv13        accccccacgactgcctcgagatcttggctgagacacacggcaccagaccggacctcacggaccagcccctcccaaatgc
Mpmv2         accccccacgactgcctcgagatcttggctgagacacacggcaccagaccggacctcacggaccagcccctcccaaatgc
Mpmv3         accccccacgactgcctcgagatcttggctgagacacacggcaccagaccggacctcacggaccagcccctcccaaatgc
Mpmv4         accccccacgactgcctcgagatcttggctgagacacacggcaccagaccggacctcacggaccagcccctcccaaatgc
Mpmv5         accccccacgactgcctcgagatcttggctgagacacacggcaccagaccggacctcacggaccagcccctcccaaatgc
Mpmv6         accccccacgactgcctcgagatcttggctgagacacacggcaccagaccggacctcacggaccagcccctcccaaatgc
Mpmv7         accccccacgactgcctcgagatcttggctgagacacacggcaccagaccggacctcacggaccagcccctcccaaatgc
Mpmv8         accccccacgactgcctcgagatcttggctgagacacacggcaccagaccggacctcacggaccagcccctcccaaatgc
Mpmv9         accccccacgactgcctcgagatcttggctgagacacacggcaccagaccggacctcacggaccagcccctcccaaatgc
```

```
MPMV_CONS    CGACCACACCTGGTATACAGATGGAAGCAGCTTCCTGCAGGAGGGGCAACGTAAGGCTGGAGCAGCGGTGACCACCGAGA
Mpmv1        cgaccacacctggtatacagatggaagcagcttcctgcaggaggggcaacgtaaggctggagcagcggtgaccaccgaga
Mpmv10       cgaccacacctggtatacagatggaagcagcttcctgcaggaggggcaacgtaaggctggagcagcggtgaccaccgaga
Mpmv11       cgaccacacctggtatacagatggaagcagcttcctgcaggaggggcaacgtaaggctggagcagcggtgaccaccgaga
Mpmv12       cgaccacacctggtatacagatggaagcagcttcctgcaggaggggcaacgtaaggctggagcagcggtgaccaccgaga
Mpmv13       cgaccacacctggtatacagatggaagcagcttcctgcaggaggggcaacgtaaggctggagcagcggtgaccaccgaga
Mpmv2        cgaccacacctggtatacagatggaagcagcttcctgcaggaggggcaacgtaaggctggagcagcggtgaccaccgaga
Mpmv3        cgaccacacctggtatacagatggaagcagcttcctgcaggaggggcaacgtaaggctggagcagcggtgaccaccgaga
Mpmv4        cgaccacacctggtatacagatggaagcagcttcctgcaggaggggcaacgtaaggctggagcagcggtgaccaccgaga
Mpmv5        cgaccacacctggtatacagatAAaagcagcttcctgcaggaggggcaacgtaaggctggagcagcggtgaccaccgaga
Mpmv6        cgaccacacctggtatacagatggaagcagcttcctgcaggaggggcaacgtaaggctggagcagcggtgaccaccgaga
Mpmv7        cgaccacacctggtatacagatggaagcagcttcctgcaggaggggcaacgtaaggctggagcagcggtgaccaccgaga
Mpmv8        cgaccacacctggtatacagatggaagcagcttcctgcaggaggggcaacgtaaggctggagcagcggtgaccaccgaga
Mpmv9        cgaccacacctggtatacagatggaagcagcttcctgcaggaggggcaacgtaaggctggagcagcggtgaccaccgaga

MPMV_CONS    CCGAGGTAATCTGGGCCAAGGCGTTGCCAGCCGGGACATCCGCCCAGCGAGCTGAACTAATAGCACTCACCCAGGCCCTA
Mpmv1        ccgaggtaatctgggccaaggcgttgccagTcgggacatccgcccagcgagctgaactaatagcactcacccaggcccta
Mpmv10       ccgaggtaatctgggccaaggcgttgccagccgggacatccgcccagcgagctgaactaatagcactcacccaggcccta
Mpmv11       ccgaggtaatctgggccaaggcgttgccagTcgggacatccgcccagcgagctgaactaatagcactcacccaggcccta
Mpmv12       ccgaggtaatctgggccaaggcgttgccagccgggacatccgcccagcgagctgaactaatagcactcacccaggcccta
Mpmv13       ccgaggtaatctgggccaaggcgttgccagccgggacatccgcccagcgagctgaactaatagcactcacccaggcccta
Mpmv2        ccgaggtaatctgggccaaggcgttgccagccgggacatccgcccagcgagctgaactaatagcactcacccaggcccta
Mpmv3        ccgaggtaatctgggccaaggcgttgccagccgggacatccgcccagcgagctgaactaatagcactcacccaggcccta
Mpmv4        ccgaggtaatctgggccaaggcgttgccagccgggacatccgcccagcgagctgaactaatagcactcacccaggcccta
Mpmv5        ccgaggtaatctgggccaaggcgttgccagccgggacatccgcccagcgagctgaactaatagcactcacccaggcccta
Mpmv6        ccgaggtaatctgggccaaggcgttgccagccgggacatccgcccagcgagctgaactaatagcactcacccaggcccta
Mpmv7        ccgaggtaatctgggccaaggcgttgccagccgggacatccgcccagcgagctgaactaatagcactcacccaggcccta
Mpmv8        ccgaggtaatctgggccaaggcgttgccagccgggacatccgcccagcgagctgaactaatagcactcacccaggcccta
Mpmv9        ccgaggtaatctgggccaaggcgttgccagccgggacatccgcccagcgagctgaactaatagcactcacccaggcccta

MPMV_CONS    AAGATGGCAGAAGGTAAGAAGCTAAATGTTTATACTGATAGCCGCTATGCCTTTGCTACCGCCCATGTCCATGGAGAAAT
Mpmv1        aagatggcagaaggtaagaagctaaatgtttatactgatagccgctatgcctttgctaccgcccatgtccatggagaaat
Mpmv10       aagatggcagaaggtaagaagctaaatgtttatactgatagccgctatgcctttgctaccgcccatgtccatggagaaat
Mpmv11       aagatggcagaaggtaagaagctaaatgtttatactgatagccgctatgcctttgctaccgcccatgtccatggagaaat
Mpmv12       aagatggcagaaggtaagaagctaaatgtttatactgatagccgctatgcctttgctaccgcccatgtccatggagaaat
Mpmv13       aagatggcagaaggtaagaagctaaatgtttatactgatagccgctatgcctttgctaccgcccatgtccatggagaaat
Mpmv2        aagatggcagaaggtaaAaagctaaatgtttatactgatagccgctatgcctttgctaccgcccatgtccatggagaaat
Mpmv3        aagatggcagaaggtaagaagctaaatgtttatactgatagccgctatgcctttgctaccgcccatgtccatggagaaat
Mpmv4        aagatggcagaaggtaagaagctaaatgtttatactgatagccgctatgcctttgctaccgcccatgtccatggagaaat
Mpmv5        aagatggcagaaggtaagaagctaaatgtttatactgatagccgctatgcctttgctaccgcccatgtccatAAaAaaat
Mpmv6        aagatggcagaaggtaagaagctaaatgtttatactgatagccgctatgcctttgctaccgcccatgtccatggagaaat
Mpmv7        aagatggcagaaggtaagaagctaaatgtttatactgatagccgctatgcctttgctaccgcccatgtccatggagaaat
Mpmv8        aagatggcagaaggtaagaagctaaatgtttatactgatagccgctatgcctttgctaccgcccatgtccatggagaaat
Mpmv9        aagatggcagaaggtaagaagctaaatgtttatactgatagccgctatgcctttgctaccgcccatgtccatggagaaat

MPMV_CONS    ATATAGGAGACGTGGGTTGCTCACCTCAGAAGGCAAGGAGATCAAGAACAAGGGCGAAATCTTGGCCTTACTGAAAGCTC
Mpmv1        atataggagacgtgggttgctcacctcagaaggcaaggagatcaagaacaagggcgaaatcttggccttactgaaagctc
Mpmv10       atataggagacgtgggttgctcacctcagaaggcaaggagatcaagaacaagggcgaaatcttggccttactgaaagctc
Mpmv11       atataggagacgtgggttgctcacctcagaaggcaaggagatcaagaacaagggcgaaatcttggccttactgaaagctc
Mpmv12       atataggagacgtgggttgctcacctcagaaggcaaggagatcaagaacaagggcgaaatcttggccttactgaaagctc
Mpmv13       atataggagacgtgggttgctcacctcagaaggcaaggagatcaagaacaagggcgaaatcttggccttactgaaagctc
Mpmv2        atataggagacgtgggttgctcacctcagaaggcaaggagatcaagaacaagggcgaaatcttggccttactgaaagctc
Mpmv3        atataggagacgtgggttgctcacctcagaaggcaaggagatcaagaacaagggcgaaatcttggccttactgaaagctc
Mpmv4        atataggagacgtgggttgctcacctcagaaggcaaggagatcaagaacaagggcgaaatcttggccttactgaaagctc
Mpmv5        atataggagacgtgggttgctcacctcaAaaggcaagAagatcaagaacaagggcAaaatcttggccttactgaaagctc
Mpmv6        atataggagacgtgggttgctcacctcagaaggcaaggagatcaagaacaagggcgaaatcttggccttactgaaagctc
Mpmv7        atataggagacgtgggttgctcacctcagaaggcaaggagatcaagaacaagggcgaaatcttggccttactgaaagctc
Mpmv8        atataggagacgtgggttgctcacctcagaaggcaaggagatcaagaacaagggcgaaatcttggccttactgaaagctc
Mpmv9        atataggagacgtgggttgctcacctcagaaggcaaggagatcaagaacaagggcgaaatcttggccttactgaaagctc
```

```
MPMV_CONS    TCTTTCTGCCCAAAAGACTCAGTATAATTCACTGCCCAGGACATCAGAAAGGCAATAGTGCTGAAGCTAAAGGCAACCGA
Mpmv1        tctttctgcccaaaagactcagtataattcactgcccaggacatcagaaaggcaatagtgctgaagctaaaggcaaccga
Mpmv10       tctttctgcccaaaagactcagtataattcactgcccaggacatcagaaaggcaatagtgctgaagctaaaggcaaccga
Mpmv11       tctttctgcccaaaagactcagtataattcactgcccaggacatcagaaaggcaatagtgctgaagctaaaggcaaccga
Mpmv12       tctttctgcccaaaagactcagtataattcactgcccaggacatcagaaaggcaatagtgctgaagctaaaggcaaccga
Mpmv13       tctttctgcccaaaagactcagtataattcactgcccaggacatcagaaaggcaatagtgctgaagctaaaggcaaccga
Mpmv2        tctttctgcccaaaagactcagtataattcactgcccaggacatcagaaaggcaatagtgctgaagctaaaggcaaccga
Mpmv3        tctttctgcccaaaagactcagtataattcactgcccaggacatcagaaaggcaatagtgctgaagctaaaggcaaccga
Mpmv4        tctttctgcccaaaagactcagtataattcactgcccaggacatcagaaaggcaatagtgctgaagctaaaggcaaccga
Mpmv5        tctttctgcccaaaagactcagtataattcactgcccaggacatcagaaagAcaatagtgctgaagctaaaggcaaccga
Mpmv6        tctttctgcccaaaagactcagtataattcactgcccaggacatcagaaaggcaatagtgctgaagctaaaggcaaccga
Mpmv7        tctttctgcccaaaagactcagtataattcactgcccaggacatcagaaaggcaatagtgctgaagctaaaggcaaccga
Mpmv8        tctttctgcccaaaagactcagtataattcactgcccaggacatcagaaaggcaatagtgctgaagctaaaggcaaccga
Mpmv9        tctttctgcccaaaagactcagtataattcactgcccaggacatcagaaaggcaatagtgctgaagctaaaggcaaccga

MPMV_CONS    ATGGCGGACCAGGCAGCCCGGGAAGCAGCCATGGGGACTGACACAAAGGCCTCCTCACTTCTCATAGAGACCTCAACCCC
Mpmv1        atggcggaccaggcagcccgggaagcagccatggggactgacacaaaggcctcctcacttctcatagagacctcaacccc
Mpmv10       atggcggaccaggcagcccgggaagcagccatggggactgacacaaaggcctcctcacttctcatagagacctcaacccc
Mpmv11       atggcggaccaggcagcccgggaagcagccatggggactgacacaaaggcctcctcacttctcatagagacctcaacccc
Mpmv12       atggcggaccaggcagcccgggaagcagccatggggactgacacaaaggcctcctcacttctcatagagacctcaacccc
Mpmv13       atggcggaccaggcagcccgggaagcagccatggggactgacacaaaggcctcctcacttctcatagagacctcaacccc
Mpmv2        atggcggaccaggcagcccgggaagcagccatggggactgacacaaaggcctcctcacttctcatagagacctcaacccc
Mpmv3        atggcggaccaggcagcccgggaagcagccatggggactgacacaaaggcctcctcaTttctcatagagacctcaacccc
Mpmv4        atggcggaccaggcagcccgggaagcagccatggggactgacacaaaggcctcctcacttctcatagagacctcaacccc
Mpmv5        atggcggaccaggcagcccgggaagcagccatggggactgacacaaaggcctcctcacttctcatagagacctcaacccc
Mpmv6        atggcggaccaggcagcccgggaagcagccatggggactgacacaaaggcctcctcacttctcatagagacctcaacccc
Mpmv7        atggcggaccaggcagcccgggaagcagccatggggactgacacaaaggcctcctcacttctcatagagacctcaacccc
Mpmv8        atggcggaccaggcagcccgggaagcagccatggggactgacacaaaggcctcctcacttctcatagagacctcaacccc
Mpmv9        atggcggaccaggcagcccgggaagcagccatggggactgacacaaaggcctcctcacttctcatagagacctcaacccc

MPMV_CONS    GTACACTCCAGACTTCTTCCATTATACTGAAACAGATATAAAGAACCTACGAGAGTTGGGAGCCACATATGATAGAGAGA
Mpmv1        gtacactccagacttcttccattatactgaaacagatataaagaacctacgagagttgggagccacatatgatagagaga
Mpmv10       gtacactccagacttcttccattatactgaaacagatataaagaacctacgagagttgggagccacatatgatagagaga
Mpmv11       gtacactccagacttcttccattatactgaaacagatataaagaacctacgagagttgggagccacatatgatagagaga
Mpmv12       gtacactccagacttcttccattatactgaaacagatataaagaacctacgagagttgggagccacatatAatagagaAa
Mpmv13       gtacactccagacttcttccattatactgaaacagatataaagaacctacgagagttgggagccacatatgatagagaga
Mpmv2        gtacactccagacttcttccattatactgaaacagatataaagaacctacgagagttgggagccacatatgatagagaga
Mpmv3        gtacactccagacttcttccattatactgaaacagatataaagaacctacgagagttgggagccacatatgatagagaga
Mpmv4        gtacactccagacttcttccattatactgaaacagatataaagaacctacgagagttgggagccacatatgatagagaga
Mpmv5        gtacactccagacttcttccattatactgaaacagatataaagaacctacAagagttgggagccacatatgatagagaga
Mpmv6        gtacactccagacttcttccattatactgaaacagatataaagaacctacAagagttgggagccacatatgatagagaga
Mpmv7        gtacactccagacttcttccattatactgaaacagatataaagaacctacgagagttgggagccacatatgatagagaga
Mpmv8        gtacactccagacttcttccattatactgaaacagatataaagaacctacgagagttgggagccacatatgatagagaga
Mpmv9        gtacactccagacttcttccattatactgaaacagatataaagaacctacgagagttgggagccacatatgatagagaga

MPMV_CONS    AAAAATATTGGGTCCTGCAAGGTAAACCTGTGATGCCTGACCAGTTCACCTTTGAATTATTAGACTTCCTTCACCAGCTC
Mpmv1        aaaaatattgggtcctgcaaggtaaacctgtgatgcctgaccagttcacctttgaattattagacttccttcaccagctc
Mpmv10       aaaaatattgggtcctgcaaggCaaacctgtgatgcctgaccagttcacctttgaattattagacttccttcaccagctc
Mpmv11       aaaaatattgggtcctgcaaggtaaacctgtgatgcctgaccagttcacctttgaattattagacttccttcaccagctc
Mpmv12       aaaaatattgggtcctgcaaggtaaacctgtgatgcctgaccagttcacctttgaattattagacttccttcaccagctc
Mpmv13       aaaaatattgggtcctgcaaggtaaacctgtgatgcctgaccagttcacctttgaattattagacttccttcaccagctc
Mpmv2        aaaaatattgggtcctgcaaggtaaacctgtgatgcctgaccagttcacctttgaattattagacttccttcaccagctc
Mpmv3        aaaaatattgggtcctgcaaggtaaacctgtgatgcctgaccagttcacctttgaattattagacttccttcaccagctc
Mpmv4        aaaaatattgggtcctgcaaggtaaacctgtgatgcctgaccagttcacctttgaattattagacttccttcaccagctc
Mpmv5        aaaaatattgggtcctgcaaggtaaacctgtgatgcctgaccagttcacctttAaattattagacttccttcaccagctc
Mpmv6        aaaaatattgggtcctgcaaggtaaacctgtgatgcctgaccagttcacctttgaattattagacttccttcaccagctc
Mpmv7        aaaaatattgggtcctgcaaggtaaacctgtgatgcctgaccagttcacctttAaattattagacttccttcaccagctc
Mpmv8        aaaaatattgggtcctgcaaggtaaacctgtgatgcctgaccagttcacctttgaattattagacttccttcaccagctc
Mpmv9        aaaaatattgggtcctgcaaggtaaacctgtgatgcctgaccagttcacctttgaattattagacttccttcaccagctc
```

```
MPMV_CONS     ACCCACCTTAGCTATCAGAAGATGAGGGCACTTCTAGACAGAAAAGAAAGCCCCTATTACATGCTAAATAAAGATAAGAT
Mpmv1         acccaccttagctatcagaagatgagggcacttctagacagaaaagaaagcccTtattacatgctaaataaagataagat
Mpmv10        acccaccttagctatcagaagatgagggcacttctagacaAaaaagaaagcccctattacatgctaaataaaAataagat
Mpmv11        acccaccttagctatcagaagatgagggcacttctagacagaaaagaaagcccTtattacatgctaaataaagataagat
Mpmv12        acccaccttagctatcagaagatgagggcacttctagacagaaaagaaagcccctattacatgctaaataaagataagat
Mpmv13        acccaccttagctatcagaagatgagggcacttctagacagaaaagaaagcccctattacatgctaaataaagataagat
Mpmv2         acccaccttagctatcagaagatgagggcacttctagacagaaaagaaagcccctattacatgctaaataaagataagat
Mpmv3         acccaccttagctatcagaagatgagggcacttctagacagaaaagaaagcccctattacatgctaaataaagataagat
Mpmv4         acccaccttagctatcagaagatgagggcacttctagacagaaaagaaagcccctattacatgctaaataaagataagat
Mpmv5         acccaccttagctatcagaagatAagggcacttctagacagaaaagaaagcccctattacatgctaaataaagataagat
Mpmv6         acccaccttagctatcagaagatgagggcacttctagacagaaaagaaagcccctattacatgctaaataaagataagat
Mpmv7         acccaccttagctatcagaagatgagggcacttctagacagaaaagaaagcccctattacatgctaaataaagataagat
Mpmv8         acccaccttagctatcagaagatgagggcacttctagacagaaaagaaagcccctattacatgctaaataaagataagat
Mpmv9         acccaccttagctatcagaagatgagggcacttctagacagaaaagaaagcccctattacatgctaaataaagataagat

MPMV_CONS     CCTCCACGAGGTGGCGGAATCATGCCAAGCCTGTGTCCAAGTAAATGCCAGTAAAGCTAAGGTCGGTCCCGGGGTGCGAG
Mpmv1         cctccacgaggtggcggaatcatgccaagcctgtgtccaagtaaatgccagtaaagctaaggtcggtcccggggtgcgag
Mpmv10        cctccacgaggtggcggaatcatgccaagcctgtgtccaagtaaatgccagtaaagctaaggtcggtcccggggtgcgag
Mpmv11        cctccacgaggtggcggaatcatgccaagcctgtgtccaagtaaatgccagtaaagctaaggtcggtcccggggtgcgag
Mpmv12        cctccacgaggtggcggaatcatgccaagcctgtgtccaagtaaatgccagtaaagctaaggtcggtcccggggtgcgag
Mpmv13        cctccacgaggtggcggaatcatgccaagcctgtgtccaagtaaatgccagtaaagctaaggtcggtcccggggtgcgag
Mpmv2         cctccacgaggtggcggaatcatgccaagcctgtgtccaagtaaatgccagtaaagctaaggtcggtcccggggtgcgag
Mpmv3         cctccacgaggtggcggaatcatgccaagcctgtgtccaagtaaatgccagtaaagctaaggtcggtcccggggtgcgag
Mpmv4         cctccacgaggtggcggaatcatgccaagcctgtgtccaagtaaatgccagtaaagctaaggtcggtcccggggtgcgag
Mpmv5         cctccacgaggtggcggaatcatgccaagcctgtgtccaagtaaatgccagtaaagctaaggtcggtcccggggtgcgag
Mpmv6         cctccacgaggtggcggaatcatgccaagcctgtgtccaagtaaatgccagtaaagctaaggtcggtcccggggtgcgag
Mpmv7         cctccacgaggtggcggaatcatgccaagcctgtgtccaagtaaatgccagtaaagctaaggtcggtcccggggtgcgag
Mpmv8         cctccacgaggtggcggaatcatgccaagcctgtgtccaagtaaatgccagtaaagctaaggtcggtcccggggtgcgag
Mpmv9         cctccacgaggtggcggaatcatgccaagcctgtgtccaagtaaatgccagtaaagctaaggtcggtcccggggtgcgag

MPMV_CONS     TAAGAGGACATCGACCAGGCACCCATTGGGAAATTGACTTTACTGAAGTAAGGCCCGGACTGTATGGGCATAAGTATCTT
Mpmv1         taagaggacatcgaccaggcacccattgggaaattgactttactgaagtaaggcccggactgtatgggcataagtatctt
Mpmv10        taagaggacatcgaccaggcacccattgggaaattgactttactgaagtaaggcccggactgtatgggcataagtatctt
Mpmv11        taagaggacatcgaccaggcacccattgggaaattgactttactgaagtaaggcccggactgtatgggcataagtatctt
Mpmv12        taagaggacatcgaccaggcacccattgggaaattgactttactgaagtaaggcccggactgtatgggcataagtatctt
Mpmv13        taagaggacatcgaccaggcacccattgggaaattgactttactgaagtaaggcccggactgtatgggcataagtatctt
Mpmv2         taagaggacatcgaccaggcacccattgggaaattgactttactgaagtaaggcccggactgtatgggcataagtatctt
Mpmv3         taagaggacatcgaccaggcacccattgggaaattgactttactgaagtaaggcccggactgtatgggcataagtatctt
Mpmv4         taagaggacatcgaccaggcacccattgggaaattgactttactgaagtaaggcccggactgtatgggcataagtatctt
Mpmv5         taagaggacatcgaccaggcacccattgAgaaattgactttactgaagtaaggcccggactgtatgggTataagtatctt
Mpmv6         taagaggacatcgaccaggcacccattgggaaattgactttactgaagtaaggcccggactgtatgggTataagtatctt
Mpmv7         taagaggacatcgaccaggcacccattgggaaattgactttactgaagtaaggcccggactgtatgggcataagtatctt
Mpmv8         taagaggacatcgaccaggcacccattgggaaattgactttactgaagtaaggcccggactgtatgggcataagtatctt
Mpmv9         taagaggacatcgaccaggcacccattgggaaattgactttactgaagtaaggcccggactgtatgggcataagtatctt

MPMV_CONS     CTGGTGTTTGTGGACACGTTCTCTGGCTGGGTGGAAGCCTTCCCAACCAAGCATGAGACTGCCAAAGTTGTGACCAAAAA
Mpmv1         ctggtgtttgtggacacgttctctggctgggtggaagccttcccaaccaagcatgagactgccaaagttgtgaccaaaaa
Mpmv10        ctggtgtttgtggacacgttctctggctgggtggaagccttcccaaccaagcatgagaTtgccaaagttgtgaccaaaaa
Mpmv11        ctggtgtttgtggacacgttctctggctgggtggaaAccttcccaaccaagcatgagactgccaaagttgtgaccaaaaa
Mpmv12        ctggtgtttgtggacacgttctctggctgggtggaagccttcccaaccaagcatgagactgccaaagttgtgaccaaaaa
Mpmv13        ctggtgtttgtggacacgttctctggctgggtggaagccttcccaaccaagcatgagactgccaaagttgtgaccaaaaa
Mpmv2         ctggtgtttgtggacacgttctctggctgggtggaagccttcccaaccaagcatgagactgccaaagttgtgaccaaaaa
Mpmv3         ctggtgtttgtggacacgttctctggctgggtggaagccttcccaaccaagcatgagactgccaaagttgtgaccaaaaa
Mpmv4         ctggtgtttgtggacacgttctctggctgggtggaagccttcccaaccaagcatgagactgccaaagttgtgaccaaaaa
Mpmv5         ctggtgtttgtggacacgttctctggctgggtAgaagccttcccaaccaagcatgagactgccaaagttgtgaccaaaaa
Mpmv6         ctggtgtttgtggacacgttctctggctgggtggaagccttcccaaccaagcatgagactgccaaagttgtgaccaaaaa
Mpmv7         ctggtgtttgtggacacgttctctggctgggtggaagccttcccaaccaagcatgagactgccaaagttgtgaccaaaaa
Mpmv8         ctggtgtttgtggacacgttctctggctgggtggaagccttcccaaccaagcatgagactgccaaagttgtgaccaaaaa
Mpmv9         ctggtgtttgtggacacgttctctggctgggtggaagccttcccaaccaagcatgagactgccaaagttgtgaccaaaaa
```

```
MPMV_CONS     GCTTCTAGAAGAAATATTTCCAAGGTTTGGAATGCCCCAAGTATTGGGGACTGATAATGGGCCTGCCTTCGTCTCCCAGG
Mpmv1         gcttctagaagaaatatttccaaggtttggaatgccccaagtattggggactgataatgggcctgccttcgtctcccagg
Mpmv10        gcttctagaagaaatatttccaaggtttggaatgccccaagtattggggactgataatgggcctgccttcgtctcccagg
Mpmv11        gcttctagaagaaatatttccaaggtttggaatgccccaagtattggggactgataatgggcctgccttcgtctcccagg
Mpmv12        gcttctagaagaaatatttccaaggtttggaatgccccaagtattggggactgataatgggcctgccttcgtctcccagg
Mpmv13        gcttctagaagaaatatttccaaggtttAgaatgccccaagtattggggactgataatgggcctgccttcgtctcccagg
Mpmv2         gcttctagaagaaatatttccaaggtttggaatgccccaagtattggggactgataatgggcctgccttcgtctcccagg
Mpmv3         gcttctagaagaaatatttccaaggtttggaatgccccaagtattggggactgataatgggcctgccttcgtctcccagg
Mpmv4         gcttctagaagaaatatttccaaggtttggaatgccccaagtattggggactgataatgggcctgccttcgtctcccagg
Mpmv5         gcttctagaaAaaatatttccaaggtttggaatgccccaagtattggggactgataatgggcctgccttcgtctcccagg
Mpmv6         gcttctagaagaaatatttccaaggtttggaatgccccaagtattggggactgataatgggcctgccttcgtctcccagg
Mpmv7         gcttctagaagaaatatttccaaggtttggaatgccccaagtattggggactgataatgggcctgccttcgtctcccagg
Mpmv8         gcttctagaagaaatatttccaaggtttggaatgccccaagtattggggactgataatgggcctgccttcgtctcccagg
Mpmv9         gcttctagaagaaatatttccaaggtttggaatgccccaagtattggggactgataatgggcctgccttcgtctcccagg

MPMV_CONS     TAAGTCAGTCGGTGGCCAAGCTACTGGGGATTGATTGGAAACTACATTGTGCTTACAGACCCCAGAGTTCAGGTCAGGTA
Mpmv1         taagtcagtcggtggccaagctactggggattgattggaaactacattgtgcttacagaccccagagttcaggtcaggta
Mpmv10        taagtcagtcggtggccaagctactggggattgattAgaaactacattgtgcttacagaccccagagttcaggtcaggta
Mpmv11        taagtcagtcggtggccaagctactggggattgattggaaactacattgtgcttacagaccccagagttcaggtcaggta
Mpmv12        taagtcagtcggtggccaagctactggggattgattggaaactacattgtgcttacagaccccagagttcaggtcaggta
Mpmv13        taagtcagtcggtggccaagctactggggattgattggaaactacattgtgcttacagaccccagagttcaggtcaggta
Mpmv2         taagtcagtcggtggccaagctactggggattgattggaaactacattgtgcttacagaccccagagttcaggtcaggta
Mpmv3         taagtcagtcggtggccaagctactggggattgattggaaactacattgtgcttacagaccccagagttcaggtcaggta
Mpmv4         taagtcagtcggtggccaagctactggggattgattggaaactacattgtgcttacagaccccagagttcaggtcaggta
Mpmv5         taagtcagtcggtggccaagctactggggattAattAAaaactacattgtgcttacagaccccagagttcaggtcaggta
Mpmv6         taagtcagtcggtggccaagctactggggattgattggaaactacattgtgcttacagaccccagagttcaggtcaggta
Mpmv7         taagtcagtcggtggccaagctactggggattgattggaaactacattgtgcttacagaccccagagttcaggtcaggta
Mpmv8         taagtcagtcggtggccaagctactggggattgattggaaactacattgtgcttacagaccccagagttcaggtcaggta
Mpmv9         taagtcagtcggtggccaagctactggggattgattggaaactacattgtgcttacagaccccagagttcaggtcaggta

MPMV_CONS     GAAAGAATGAATAGGACAATCAAGGAGACTTTGACCAAATTAACGCTTGCAACTGGCACTAGAGACTGGGTACTCCTACT
Mpmv1         gaaagaatgaataggacaatcaaggagactttgaccaaattaacgcttgcaactggcactagagactgggtactcctact
Mpmv10        Aaaagaatgaataggacaatcaaggagactttgaccaaattaacgcttgcaactggcactagagactgggtactcctact
Mpmv11        gaaagaatgaataggacaatcaaggagactttgaccaaattaacgcttgcaactggcactagagactgggtactcctact
Mpmv12        gaaagaatgaataggacaatcaaggagactttgaccaaattaacgcttgcaactggcactagagactgggtactcctact
Mpmv13        gaaagaatgaataggacaatcaaggagactttgaccaaattaacgcttgcaactggcactagagactgggtactcctact
Mpmv2         gaaagaatgaataggacaatcaaggagactttgaccaaattaacgcttgcaactggcactagagactgggtactcctact
Mpmv3         gaaagaatgaataggacaatcaaggagactttgaccaaattaacgcttgcaactggcactagagactgggtactcctact
Mpmv4         gaaagaatgaataggacaatcaaggagactttgacTaaattaacgcttgcaactggcactagagactgggtactcctact
Mpmv5         AaaagaatgaataAgacaatcaaggagactttgaccaaattaacgcttgcaactggcactagagactgggtactcctact
Mpmv6         gaaagaatgaataggacaatcaaggagactttgaccaaattaacgcttgcaactggcactagagactgggtactcctact
Mpmv7         gaaagaatAaataggacaatcaaggagactttgaccaaattaacgcttgcaactggcactagagactgggtactcctact
Mpmv8         AaaagaatgaataggacaatcaaggagactttgacTaaattaacgcttgcaactggcactagagactgggtactcctact
Mpmv9         Aaaagaatgaataggacaatcaaggagactttgaccaaattaacgcttgcaactggcactagagactgggtactcctact

MPMV_CONS     TCCCTTGGCCCTCTACCGAGCCCGCAACACTCCGGGCCCCCATGGACTCACTCCGTATGAAATCCTGTATGGGGCGCCCC
Mpmv1         tcccttggccctctaccgagcccgcaacactccgggcccccatggactcactccgtatgaaatcctgtatggggcgcccc
Mpmv10        tcccttggccctctaccgagcccgcaacactccgggcccccatggactcactccgtatgaaatcctgtatggggcgcccc
Mpmv11        tcccttggccctctaccgagcccgcaacactccgggcccccatggactcactccgtatgaaatcctgtatggggcgcccc
Mpmv12        tcccttggccctctaccgagcccgcaacactccgggcccccatggactcactccgtatgaaatcctgtatggggcgcccc
Mpmv13        tcccttggccctctaccgagcccgcaacactccgggcccccatggactcactccgtatgaaatcctgtatggggcgcccc
Mpmv2         tcccttggccctctaccgagcccgcaacactccgggcccccatggactcactccgtatgaaatcctgtatggggcgcccc
Mpmv3         tcccttggccctctaccgagcccgcaacactccgggcccccatggactcactccgtatgaaatcctgtatggggcgcccc
Mpmv4         tcccttggccctctaccgagcccgcaacactccgggcccccatggactcactccgtatgaaatcctgtatggggcgcccc
Mpmv5         tcccttggccctctaccgagcccgcaacactccgggcccccatggactcactccgtatAaaatcctgtatggggcgcccc
Mpmv6         tcccttggccctctaccgagcccgcaacactccgggcccccatggactcactccgtatgaaatcctgtatggggcgccAc
Mpmv7         tcccttggccctctaccgagcccgcaacactccgggcccccatggactcactccgtatgaaatcctgtatggggcgcccc
Mpmv8         tcccttggccctctaccgagcccgcaacactccgggcccccatggactcactccgtatgaaatcctgtatggggcgcccc
Mpmv9         tcccttggccctctaccgagcccgcaacactccgggcccccatggactcactccgtatgaaatcctgtatggggcgcccc
```

```
MPMV_CONS     CGCCCCTTGTTAATTTCCATGATCCTGAAATGTCAAAGTTTACTAATAGCCCCTCTCTCCAAGCTCACTTACAGGCCCTC
Mpmv1         cgccccttgttaatttccatgatcctgaaatgtcaaagtttactaatagcccctctctccaagctcacttacaggccctc
Mpmv10        cgccccttgttaatttccatgatcctgaaatgtcaaagtttactaatagcccctctctccaagctcacttacaggccctc
Mpmv11        cgccccttgttaatttccatgatcctgaaatgtcaaagtttactaatagcccctctctccaagctcacttacaggccctc
Mpmv12        cgccccttgttaatttccatgatcctgaaatgtcaaagtttactaatagcccctctctccaagctcacttacaggccctc
Mpmv13        cgccccttgttaatttccatgatcctgaaatgtcaaagtttactaatagcccctctctccaagctcacttacaggccctc
Mpmv2         cgccccttgttaatttccatgatcctgaaatgtcaaagtttactaatagcccctctctccaagctcacttacaggccctc
Mpmv3         cgccccttgttaatttccatgatcctgaaatgtcaaagtttactaatagcccctctctccaagctcacttacaggccctc
Mpmv4         cgccccttgttaatttccatgatcctgaaatgtcaaagtttactaatagcccctctctccaagctcacttacaggccctc
Mpmv5         cgccccttgttaatttccatgatcctAaaatgtcaaagtttactaatagcccctctctccaagctcacttacaggccctc
Mpmv6         cgccccttgttaatttccatgatcctgaaatgtcaaagtttactaatagcccctctctccaagctcacttacaggccctc
Mpmv7         cgccccttgttaatttccatgatcctgaaatgtcaaagtttactaatagcccctctctccaagctcacttacaggccctc
Mpmv8         cgccccttgttaatttccatgatcctgaaatgtcaaagtttactaatagcccctctctccaagctcacttacaggccctc
Mpmv9         cgccccttgttaatttccatgatcctgaaatgtcaaagtttactaatagcccctctctccaagctcacttacaggccctc

MPMV_CONS     CAAGCAGTACAACGAGAGGTCTGGAAGCCTCTGGCCGCTGCCTATCAGGACCAACAAGACCAGCCTGTGATACCACACCC
Mpmv1         caagcagtacaacgagaggtctggaagcctctggccgctgcctatcaggaccaacaagaccagcctgtgataccacaccc
Mpmv10        caagcagtacaacAagaggtctggaagccGctggccgctgcctatcaggaccaacaagaccagcctgtgataccacaccc
Mpmv11        caagcagtacaacgagaggtctggaagcctctggccgctgcctatcaggaccaacaagaccagcctgtgataccacaccc
Mpmv12        caagcGgtacaacgagaggtctggaagcctctggccgctgcctatcaggaccaacaagaccagcctgtgataccacaccc
Mpmv13        caagcagtacaacgagaggtctggaagcctctggccgctgcctatcaggaccaacaagaccagcctgtgataccacaccc
Mpmv2         caagcGgtacaacgagaggtctggaagcctctggccgctgcctatcaggaccaacaagaccagcctgtgataccacaccc
Mpmv3         caagcagtacaacgagaggtctggaagcctctggccgctgcctatcaggaccaacaagaccagcctgtAataccacaccc
Mpmv4         caagcagtacaacgagaggtctggaagcctctggccgctgcctatcaggaccaacaagaccagcctgtgataccacaccc
Mpmv5         caagcagtacaacgagaggtctggaagcctctggccgctgcctatcaggaccaacaagaccagcctgtgataccacaccc
Mpmv6         caagcagtacaacgagaggtctggaagcctctggccgctgcctatcaggaccaacaagaccagcTtgtgataccacaccc
Mpmv7         caagcagtacaacgagaggtctggaagcctctggccgctgcctatcaggaccaacaagaccagcctgtgataccacaccc
Mpmv8         caagcagtacaacgagaggtctgAaagcctctggccgctgcctatcaggaccaacaagaccagcctgtgataccacaccc
Mpmv9         caagcGgtacaacgagaggtctggaagcctctggccgctgcctatcaggaccaacaagaccagcctgtgataccacaccc

MPMV_CONS     CTTCCGTGTCGGCGACACCGTGTGGGTACGCCGGCACCAGACTAAGAACTTGGAACCTCGCTGGAAAGGACCCTACACCG
Mpmv1         cttccgtgtcggcgacaccgtgtgggtacgccggcaccagactaagaacttggaacctcgctggaaaggaccctacaccg
Mpmv10        cttccgtgtcggcgacaccgtgtgggtacgccggcaccagactaagaacttggaacctcgctggaaaggaccctacaccg
Mpmv11        cttccgtgtcggcgacaccgtgtgggtacgccggcaccagactaagaacttggaacctcgctggaaaggaccctacaccg
Mpmv12        cttccgtgtcggcgacaccgtgtgggtacgccggcaccagactaagaacttggaacctcgctggaaaggaccctacaccg
Mpmv13        cttccgtgtcggcgacaccgtgtgggtacgccggcaccagactaagaacttggaacctcgctgAaaaggaccctacaccg
Mpmv2         cttccgtgtcggcgacaccgtgtgggtacgccggcaccagactaagaacttggaacctcgctggaaaggaccctacaccg
Mpmv3         cttccgtgtcggcgacaccgtgtgggtacgccggcaccagactaagaacttggaacctcgctggaaaggaccctacaccg
Mpmv4         cttccgtgtcggcgacaccgtgtgggtacgccggcaccagactaagaacttggaacctcgctggaaaggaccctacaccg
Mpmv5         cttccgtgtcggcgacaccgtgtgggtacgccggcaccagactaagaacttgAaacctcgctggaaaggaccctacaccg
Mpmv6         cttccgtgtcggcgacaccgtgtgggtacgccggcaccagactaagaacttggaacctcgctggaaaggaccctacaccg
Mpmv7         cttccgtgtcggcgacaccgtgtgggtacgccggcaccagactaagaacttggaacctcgctggaaaggaccctacaccg
Mpmv8         cttccgtgtcggcgacaccgtgtgggtacgccggcaccagactaagaacttggaacctcgctggaaaggaccctacaccg
Mpmv9         cttccgtgtcggcgacaccgtgtgggtacgccggcaccagactaagaacttggaacctcgctggaaaggaccctacaccg

MPMV_CONS     TCCTGCTGACCACCCCCCACCGCTCTCAAAGTAGACGGCATCGCTGCGTGGATCCACGCCGCTCACGTAAAAGCGGCGACA
Mpmv1         tcctgctgaccaccccccaccgctctcaaagtagacggcatcgctgcgtggatccacgccgctcacgtaaaagcggcgaca
Mpmv10        tcctgctgaccaccccccaccgctctcaaagtagacggcatcgctgcgtggatccacgccgctcacgtaaaagcggcgaca
Mpmv11        tcctgctgaccaccccccaccgctctcaaagtagacggcatcgctgcgtggatccacgccgctcacgtaaaagcggcgaca
Mpmv12        tcctgctgaccaccccccaccgctctcaaagtagacggcatcgctgcgtggatccacgccgctcacgtaaaagcggcgaca
Mpmv13        tcctgctgaccaccccccaccgctctcaaagtagacggcatcgctgcgtggatccacgccgctcacgtaaaagcggcgaca
Mpmv2         tcctgctgaccaccccccaccgctctcaaagtagacggcatcgctgcgtggatccacgccgctcacgtaaaagcggcgaca
Mpmv3         tcctgctgaccaccccccaccgctctcaaagtagacggcatcgctgcgtggatccacgccgctcacgtaaaagcggcgaca
Mpmv4         tcctgctgaccaccccccaccgctctcaaagtagacggcatcgctgcgtggatccacgccgctcacgtaaaagcggcgaca
Mpmv5         tcctgctgaccacTccccaccgctctcaaagtagacggcatcgctgcgtggatccacgccgctcacgtaaaagcggcgaca
Mpmv6         tcctgctgaccaccccccaccgctctcaaagtagacggcatcgctgcgtggatccacgccgctcacgtaaaagcggcgaca
Mpmv7         tcctgctgaccaccccccaccgctctcaaagtagacggcatcgctgcgtggatccacgccgctcacgtaaaagcggcgaca
Mpmv8         tcctgctgaccaccccccaccgctctcaaagtagacggcatcgctgcgtggatccacgccgctcacgtaaaagcggcgaca
Mpmv9         tcctgctgaccaccccccaccgctctcaaagtagacggcatcgctgcgtggatccacgccgctcacgtaaaagcggcgaca
```

```
MPMV_CONS     ACCCCTCCGGCCGGAACAGCATCAGGACCGACATGGAAGGTCCAGCGTTCTCAAAACCCCTTAAAGATAAGATTAACCCG
Mpmv1         acccctccggccggaacagcatcaggaccgacatggaaggtccagcgttctcaaaaccccttaaagataagattaacccg
Mpmv10        acccctccggccggaacagcatcaggaccgacatggaaggtccagcgttctcaaaaccccttaaagataagattaacccg
Mpmv11        acccctccggccggaacagcatcaggaccgacatggaaggtccagcgttctcaaaaccccttaaagataagattaacccg
Mpmv12        acccctccggccggaacagcatcaggaccgacatggaaggtccagcgttctcaaaacccTttaaagataagattaacccg
Mpmv13        acccctccggccggaacagcatcaggaccgacatggaaggtccagcgttctcaaaaccccttaaagataagattaacccg
Mpmv2         acccctccggccggaacagcatcaggaccgacatggaaggtccagcgttctcaaaacccTttaaagataagattaacccg
Mpmv3         acccctccggccggaacagcatcaggaccgacatggaaggtccagcgttctcaaaaccccttaaagataagattaacccg
Mpmv4         acccctccggccggaacagcatcaggaccgacatggaaggtccagcgttctcaaaaccccttaaagataagattaacccg
Mpmv5         acccctccggccggaacagcatcaggaccgacatggaaggtccagcgttctcaaaaccccttaaagataagattaacccg
Mpmv6         acccctccggccggaacagcatcaggaccgacatggaaggtccagcgttctcaaaaccccttaaagataagattaacccg
Mpmv7         acccctccggccggaacagcatcaggaccgacatggaaggtccagcgttctcaaaaccccttaaagataagattaacccg
Mpmv8         acccctccggccggaacagcatcaggaccgacatggaaggtccagcgttctcaaaaccccttaaagataagattaacccg
Mpmv9         acccctccggccggaacagcatcaggaccgacatggaaggtccagcgttctcaaaacccTttaaagataagattaacccg

MPMV_CONS     TGGGCCCCCCATGGAAGGTCCAGCGTTCTCAAAACCCCTTAAAGATAAGATTAACCCGTGGGCCCCCCTGATAGTCCTGG
Mpmv1         tgggccccccatggaaggtccagcgttctcaaaaccccttaaagataagattaacccgtgggcccccctgatagtcctgg
Mpmv10        tgggccccccatggaaggtccagcgttctcaaaaccccttaaagataagattaacccgtgggcccccctgatagtcctgg
Mpmv11        tgggccccccatggaaggtccagcgttctcaaaaccccttaaagataagattaacccgtgggcccccctgatagtcctgg
Mpmv12        tgggccccccatggaaggtccagcgttctcaaaacccTttaaagataagattaacccgtgggcccccctgatagtcctgg
Mpmv13        tgggccccccatggaaggtccagcgttctcaaaaccccttaaagataagattaacccgtgggcccccctgatagtcctgg
Mpmv2         tgggccccccatggaaggtccagcgttctcaaaacccTttaaagataagattaacccgtgggcccccctgatagtcctgg
Mpmv3         tgggccccccatggaaggtccagcgttctcaaaaccccttaaagataagattaacccgtgggcccccctgatagtcctgg
Mpmv4         tgggccccccatggaaggtccagcgttctcaaaaccccttaaagataagattaacccgtgggcccccctgatagtcctgg
Mpmv5         tgggccccccatggaaggtccagcgttctcaaaaccccttaaagataagattaacccgtgggcccccctgatagtcctgg
Mpmv6         tgggccccccatggaaggtccagcgttctcaaaaccccttaaagataagattaacccgtgggcccccctgatagtcctgg
Mpmv7         tgggccccccatggaaggtccagcgttctcaaaaccccttaaagataagattaacccgtgggcccccctgatagtcctgg
Mpmv8         tgggccccccatggaaggtccagcgttctcaaaaccccttaaagataagattaacccgtgggcccccctgatagtcctgg
Mpmv9         tgggccccccatggaaggtccagcgttctcaaaacccTttaaagataagattaacccgtgggcccccctgatagtcctgg

MPMV_CONS     GGATCTTAATAAGGGCAGGAGTATCAGTACCACATGACAGCCCTCATCAGGTCTTCAATGTTACTTGGAGAGTTACCAAC
Mpmv1         ggatcttaataagggcaggagtatcagtaccacatgacagccctcatcaggtcttcaatgttacttggagagttaccaac
Mpmv10        Agatcttaataagggcaggagtatcagtaccacatgacagccctcatcaggtcttcaatgttacttggagagttaccaac
Mpmv11        ggatcttaataagggcaggagtatcagtaccacatgacagccctcatcaggtcttcaatgttacttggagagttaccaac
Mpmv12        ggatcttaataagggcaggagtatcagtaccacatgacagccctcatcaggtcttcaatgttacttggagagttaccaac
Mpmv13        ggatcttaataagggcaggagtatcagtaccacatgacagccctcatcaggtcttcaatgttacttggagagttaccaac
Mpmv2         ggatcttaataagggcaggagtatcagtaccacatgacagccctcatcaggtcttcaatgttacttggagagttaccaac
Mpmv3         ggatcttaataagggcaggagtatcagtaccacatgacagccctcatcaggtcttcaatgttacttggagagttaccaac
Mpmv4         ggatcttaataagggcaggagtatcagtaccacatgacagccctcatcaggtcttcaatgttacttggagagttaccaac
Mpmv5         ggatcttaataagggcaggagtatcagtaccacatgacagccctcatcaggtcttcaatgttacttAgagagttaccaac
Mpmv6         ggatcttaataagggcaggagtatcagtaccacatgacagccctcatcaggtcttcaatgttacttggagagttaccaac
Mpmv7         ggatcttaataagggcaggagtatcagtaccacatgacagccctcatcaggtcttcaatgttacttggagagttaccaac
Mpmv8         ggatcttaataagggcaggagtatcagtaccacatgacagccctcatcaggtcttcaatgttacttggagagttaccaac
Mpmv9         ggatcttaataagggcaggagtatcagtaccacatgacagccctcatcaggtcttcaatgttacttggagagttaccaac

MPMV_CONS     TTAATGACAGGACAAACAGCTAATGCTACCTCCCTCCTGGGGACAATGACCGATGCCTTTCCCAAACTGTACTTTGACTT
Mpmv1         ttaatgacaggacaaacagctaatgctacctccctcctggggacaatgaccgatgcctttcccaaactgtactttgactt
Mpmv10        ttaatgacaggacaaacagctaatgctacctccctcctggggacaatgaccgatgcctttcccaaactgtactttgactt
Mpmv11        ttaatgacaggacaaacagctaatgctacctccctcctggggacaatgaccgatgcctttcccaaactgtactttgactt
Mpmv12        ttaatgacaggacaaacagctaatgctacctccctcctggggacaatgaccgatgcctttcccaaactgtactttgactt
Mpmv13        ttaatgacaggacaaacagctaatgctacctccctcctggggacaatgaccgatgcctttcccaaactgtactttgactt
Mpmv2         ttaatgacaggacaaacagctaatgctacctccctcctggggacaatgaccgatgcctttcccaaactgtactttgactt
Mpmv3         ttaatgacaggacaaacagctaatgctacctccctcctggggacaatgaccgatgcctttcccaaactgtactttgactt
Mpmv4         ttaatgacaggacaaacagctaatgctacctccctcctggggacaatgaccgatgcctttcccaaactgtactttgactt
Mpmv5         ttaatgacaggacaaacagctaatgctacctccctcctggggacaatgaccgatgcctttcccaaactgtactttgactt
Mpmv6         ttaatgacaggacaaacagctaatgctacctccctcctggggacaatgaccgatgcctttcccaaactgtactttgactt
Mpmv7         ttaatgacaggacaaacagctaatgctacctccctcctggggacaatgaccgatgcctttcccaaactgtactttgactt
Mpmv8         ttaatgacaggacaaacagctaatgctacctccctcctggggacaatgaccgatgcctttcccaaactgtactttgactt
Mpmv9         ttaatgacaggacaaacagctaatgctacctccctcctggggacaatgaccgatgcctttcccaaactgtactttgactt
```

```
MPMV_CONS     GTGCGATTTAATAGGGGACGACTGGGATGAGACTGGACTCGGGTGTCGCACTCCCGGGGGAAGAAAAAGGGCAAGAACAT
Mpmv1         gtgcgatttaataggggacgactgggatgagactggactcgggtgtcgcactcccgggggaagaaaaagggcaagaacat
Mpmv10        gtgcgatttaataggggacgactgggatgagactggactcgggtgtcgcactcccgggggaagaaaaagggcaagaacat
Mpmv11        gtgcgatttaataggggacgactgggatgagactggactcgggtgtcgcactcccgggggaagaaaaagggcaagaacat
Mpmv12        gtgcgaCttaataggggacgactgggatgagactggactcgggtgtcgcactcccgggggaagaaaaagggcaagaacat
Mpmv13        gtgcgatttaataggggacgactgggatgagactggactcgggtgtcgcactcccggggAaaAaaaaagggcaagaacat
Mpmv2         gtgcgaCttaataggggacgactgggatgagactggactcgggtgtcgcactcccgggggaagaaaaagggcaagaacat
Mpmv3         gtgcgatttaataggggacgactgggatgagactggactcgggtgtcgcactcccgggggaagaaaaagggcGagaacat
Mpmv4         gtgcgatttaataggggacgactgggatgagactggactcgggtgtcgcactcccgggggaagaaaaagggcaagaacat
Mpmv5         gtgcAatttaataggggacgactgggatgagactggactcgggtgtcgcactcccggggAaagaaaaagggcaaAaacat
Mpmv6         gtgcgatttaataggggacgactgggatgagactggactcgggtgtcgcactcccgggggaagaaaaagggcaagaacat
Mpmv7         gtgcgatttaataggggacgactgggatgagactggactcgggtgtcgcactcccgggggaagaaaaagggcaagaacat
Mpmv8         gtgcgatttaataggggacgactgggatgagactggactcgggtgtcgcactcccgggggaagaaaaagggcaagaacat
Mpmv9         gtgcgaCttaataggggacgactgggatgagactggactcgggtgtcgcactcccgggggaagaaaaagggcaagaacat

MPMV_CONS     TTGACTTCTATGTTTGCCCCGGGCATACTGTACCAACAGGGTGTGGAGGCCCGAGAGAGGGCTACTGTGGCAAATGGGGC
Mpmv1         ttgacttctatgtttgccccgggcatactgtaccaacagggtgtggaggcccgagagagggctactgtggcaaatggggc
Mpmv10        ttgacttctatgtttgccccgggcatactgtaccaacagggtgtggaggcccgagagagggctactgtggcaaatggggc
Mpmv11        ttgacttctatgtttgccccgggcatactgtaccaacagggtgtggaggcccgagagagggctactgtggcaaatggggc
Mpmv12        ttgacttctatgtttgccccgggcatactgtaccaacagggtgtggaggcccgagagagggctactgtggcaaatggggc
Mpmv13        ttgacttctatgtttgccccgggcatactgtaTcaacagggtgtggaggcccgagagagggctactgtggcaaatggggc
Mpmv2         ttgacttctatgtttgccccgggcatactgtaccaacagggtgtggaggcccgagagagggctactgtggcaaatggggc
Mpmv3         ttgacttctatgtttgccccgggcatactgtaccaacagggtgtggaggcccgagagagggctactgtggcaaatggggc
Mpmv4         ttgacttctatgtttgccccgggcatactgtaccaacagggtgtggaggcccgagagagggctactgtggcaaatggggc
Mpmv5         ttgacttctatgtttgccccgggcatactgtaccaacagggtgtggaggcccgagagagggctactgtggcaaatggggc
Mpmv6         ttgacttctatgtttgccccgggcatactgtaccaacagggtgtggaggcccgagagagggctactgtggcaaatggggc
Mpmv7         ttgacttctatgtttgccccgggcatactgtaccaacagggtgtggaggcccgagagagggctactgtggcaaatggggc
Mpmv8         ttgacttctatgtttgccccgggcatactgtaccaacagggtgtggaggcccgagagagggctactgtggcaaatggggc
Mpmv9         ttgacttctatgtttgccccgggcatactgtaccaacagggtgtggaggcccgagagagggctactgtggcaaatggggc

MPMV_CONS     TGTGAGACCACTGGACAGGCATACTGGAAGCCATCATCATCATGGGACCTAATTTCCCTTAAGCGAGGAAACACCCCTCG
Mpmv1         tgtgagaccactggacaggcatactggaagccatcatcatcatgggacctaatttcccttaagcgaggaaacacccctcg
Mpmv10        tgtgagaccactggacaggcatactggaagccatcatcatcatgggacctaatttcccttaagcgaggaaacacccctcg
Mpmv11        tgtgagaccactggacaggcatactggaagccatcatcatcatgggacctaatttcccttaagcgaggaaacacccctcg
Mpmv12        tgtgagaccactggacaggcatactggaagccatcatcatcatgggacctaatttcccttaagcgaggaaacacccctcg
Mpmv13        tgtgagaccactggacaggcatactggaagccatcatcatcatgggacctaatttcccttaagcgaggaaacacccctcg
Mpmv2         tgtgagaccactggacaggcatactggaagccatcatcatcatgggacctaatttcccttaagcgaggaaacacccctcg
Mpmv3         tgtgagaccactggacaggcatactggaagccatcatcatcatgggacctaatttcccttaagcgaggaaacacccctcg
Mpmv4         tgtgagaccactggacaggcatactggaagccatcatcatcatgggacctaatttcccttaagcgaggaaacacccctcg
Mpmv5         tgtgagaccactggacaggcatactggaagccatcatcatcatgggacctaatttcccttaagcgaggaaacacccctcg
Mpmv6         tgtgagaccactggacaggcatactggaagccatcatcatcatgggacctaatttcccttaagcgaggaaacacccctcg
Mpmv7         tgtgagaccactggacaggcatactggaagccatcatcatcatgggacctaatttcccttaagcgaggaaacacccctcg
Mpmv8         tgtgagaccactggacaggcatactggaagccatcatcatcatgggacctaatttcccttaagcgaggaaacacccctcg
Mpmv9         tgtgagaccactggacaggcatactggaagccatcatcatcatgggacctaatttcccttaagcgaggaaacacccctcg

MPMV_CONS     GAATCAGGGCCCCTGTTATGATTCCTCAGCGGTCTCCAGTGGCATCCAGGGTGCCACACCGGGGGGTCGATGCAATCCCC
Mpmv1         gaatcagggcccctgttatgattcctcagcggtctccagtggcatccagggtgccacaccggggggtcgatgcaatcccc
Mpmv10        gaatcagggcccctgttatgattcctcagcggtctccagtggcatccagggtgccacaccggggggtcgatgcaatcccc
Mpmv11        gaatcagggcccctgttatgattcctcagcggtctccagtggcatccagggtgccacaccggggggtcgatgcaatcccc
Mpmv12        gaatcagggcccctgttatgattcctcagcggtctccagtggcatccagggtgccacaccggggggtcgatgcaatcccc
Mpmv13        gaatcagggcccctgttatgattcctcagcggtctccagtggcatccagggtgccacaccggggggtcgatgcaatcccc
Mpmv2         gaatcagggcccctgttatgattcctcagcggtctccagtggcatccagggtgccacaccggggggtcgatgcaatcccc
Mpmv3         gaatcagggcccctgttatgattcctcagcggtctccagtggcatccagggtgccacaccggggggtcgatgcaatcccc
Mpmv4         gaatcagggcccctgttatgattcctcagcggtctccagtggcatccagggtgccacaccggggggtcgatgcaatcccc
Mpmv5         gaatcagggcccctgttatgattcctcagcggtctccagtgAcatccagggtgccacaccggggggtcgatgcaatcccc
Mpmv6         gaatcagggcccctgttatgattcctcagcggtctccagtggcatccagggtgccacacAggggggtcgatgcaatcccc
Mpmv7         gaatcagggcccctgttatgattcctcagcggtctccagtggcatccagggtgccacaccggggggtcgatgcaatcccc
Mpmv8         gaatcagggcccctgttatgattcctcagcggtctccagtggcatccagggtgccacaccggggggtcgatgcaatcccc
Mpmv9         gaatcagggcccctgttatgattcctcagcggtctccagtggcatccagggtgccacaccggggggtcgatgcaatcccc
```

```
MPMV_CONS    TAGTCCTAGAATTCACTGACGCGGGTAAAAAGGCCAGCTGGGATGGCCCCAAAGTATGGGGACTAAGACTGTACCGATCC
Mpmv1        tagtcctagaattcactgacgcgggtaaaaaggccagctgggatggccccaaagtatggggactaagactgtaccgatcc
Mpmv10       tagtcctagaattcactgacgcgggtaaaaaggccagctgggatggccccaaagtatggggactaagactgtaccgatcc
Mpmv11       tagtcctagaattcactgacgcgggtaaaaaggccagctgggatggccccaaagtatggggactaagactgtaccgatcc
Mpmv12       tagtcctagaattcactgacgcgggtaaaaaggccagctgggatggccccaaagtatggggactaagactgtaccgatcc
Mpmv13       tagtcctagaattcactgacgcgggtaaaaaggccagctgggatggccccaaagtatggggactaagactgtaccgatcc
Mpmv2        tagtcctagaattcactgacgcgggtaaaaaggccagctgggatggccccaaagtatggggactaagactgtaccgatcc
Mpmv3        tagtcctagaattcactgacgcgggtaaaaaggccagctgggatggccccaaagtatggggactaagactgtaccgatcc
Mpmv4        tagtcctagaattcactgacgcgggtaaaaaggccagctgggatggccccaaagtatggggactaagactgtaccgatcc
Mpmv5        tagtcctagaattcactgacgcgggtaaaaaggccagctgggatggccccaaagtatggggactaagactgtaccgatcc
Mpmv6        tagtcctagaattcactgacgcgggtaaaaaggccagctgggatggccccaaagtatggggactaagactgtaccgatcc
Mpmv7        tagtcctagaattcactgacgcgggtaaaaaggccagctgggatggccccaaagtatggggactaagactgtaccgatcc
Mpmv8        tagtcctagaattcactgacgcgggtaaaaaggccagctgggatggccccaaagtatggggactaagactgtaccgatcc
Mpmv9        tagtcctagaattcactgacgcgggtaaaaaggccagctgggatggccccaaagtatggggactaagactgtaccgatcc

MPMV_CONS    ACAGGAACCGACCCGGTGACCCGGTTCTCTTTGACCCGCCAGGTCCTCAATATAGGGCCCCGCATCCCCATTGGGCCTAA
Mpmv1        acaggaaccgacccggtgacccggttctctttgacccgccaggtcctcaatatagggccccgcatccccattgggcctaa
Mpmv10       acaggaaccgacccggtgacccggttctctttgacccgccaggtcctcaatatagggccccgcatccccattgggcctaa
Mpmv11       acaggaaccgacccggtgacccggttctctttgacccgccaggtcctcaatatagggccccgcatccccattgggcctaa
Mpmv12       acaggaaccgacccggtgacccggttctctttgacccgccaggtcctcaatatagggccccgcatccccattgggcctaa
Mpmv13       acaggaaccgacccggtgacccggttctctttgacccgccaggtcctcaatatagggccccgcatccccattgggcctaa
Mpmv2        acaggaaccgacccggtgacccggttctctttgacccgccaggtcctcaatatagggccccgcatccccattgggcctaa
Mpmv3        acaggaaccgacccggtgacccggttctctttgacccgccaggtcctcaatatagggccccgcatccccattgggcctaa
Mpmv4        acaggaaccgacccggtgacccggttctctttgacccgccaggtcctcaatatagggccccgcatccccattgggcctaa
Mpmv5        acaAgaaccgacccggtgacccggttctctttgacccgccaggtcctcaatatagggccccgcatccccattgggcctaa
Mpmv6        acaggaaccgacccggtgacccggttctctttgacccgccaggtcctcaatatagggccccgcatccccattgggcctaa
Mpmv7        acaggaaccgacccggtgacccggttctctttgacccgccaggtcctcaatatagggccccgcatccccattgggcctaa
Mpmv8        acaggaaccgacccggtgacccggttctctttgacccgccaggtcctcaatatagggccccgcatccccattgggcctaa
Mpmv9        acaggaaccgacccggtgacccggttctctttgacccgccaggtcctcaatatagggccccgcatccccattgggcctaa

MPMV_CONS    TCCCGTGATCACTGACCAGTTACCCCCCTCCCGACCCGTGCAGATCATGCTCCCCAGGCCTCCTCAGCCTTCCCCTACAG
Mpmv1        tcccgtgatcactgaccagttacccccctcccgacccgtgcagatcatgctccccaggcctcctcagccttcccctacag
Mpmv10       tcccgtgatcactgaccagttacccccctcccgacccgtgcagatcatgctccccaggcctcctcagccttcccctacag
Mpmv11       tcccgtgatcactgaccagttacccccctcccgacccgtgcagatcatgctccccaggcctcctcagccttcccctacag
Mpmv12       tcccgtgatcactgaccagttacccccctcccgacccgtgcagatcatgctccccaggcctcctcagccttcccctacag
Mpmv13       tcccgtgatcactgaccagttacccccctcccgacccgtgcagatcatgctccccaggcctcctcagccttcccctacag
Mpmv2        tcccgtgatcactgaccagttacccccctcccgacccgtgcagatcatgctccccaggcctcctcagccttcccctacag
Mpmv3        tcccgtgatcactgaccagttacccccctcccgacccgtgcagatcatgctccccaggcctcctcagccttcccctacag
Mpmv4        tcccgtgatcactgaccagttacccccctcccgacccgtgcagatcatgctccccaggcctcctcagccttcccctacag
Mpmv5        tcccgtgatcactgaccagttacccccctcccgacccgtgcagatcatgctccccaggcctcctcagccttcccctacag
Mpmv6        tcccgtgatcactgaccagttacccccctcccgacccgtgcagatTatgctccccaggcctcctcagccttcccctacag
Mpmv7        tcccgtgatcactgaccagttacccccctcccgacccgtgcagatcatgctccccaggcctcctcagccttcccctacag
Mpmv8        tcccgtgatcactgaccagttacccccctcccgacccgtgcagatcatgctccccaggcctcctcagccttcccctacag
Mpmv9        tcccgtgatcactgaccagttacccccctcccgacccgtgcagatcatgctccccaggcctcctcagccttcccctacag

MPMV_CONS    GCGCAGCCTCTATACAACCTGGGACGGGAGACAGACTGCTGAACCTGGTAGATGGAGCCTACCAAGCACTCAACCTCACC
Mpmv1        gcgcagcctctatacaacctgggacgggagacagactgctgaacctggtagatggagcctaccaagcactcaacctcacc
Mpmv10       gcgcagcctctatacaacctgggacgggagacagactgctgaacctggtagatggagcctaccaagcactcaacctcacc
Mpmv11       gcgcagcctctatacaacctgggacgggagacagactgctgaacctggtagatggagcctaccaagcactcaacctcacc
Mpmv12       gcgcagcctctatacaacctgggacgggagacagactgctgaacctggtagatggagcctaccaagcactcaacctcacc
Mpmv13       gcgcagcctctatacaacctgggacgggagacagactgctgaacctggtagatggagcctaccaagcactcaacctcacc
Mpmv2        gcgcagcctctatacaacctgggacgggagacagactgctgaacctggtagatggagcctaccaagcactcaacctcacc
Mpmv3        gcgcagcctctatacaacctgggacgggagacagactgctgaacctggtagatggagcctaccaagcactcaacctcacc
Mpmv4        gcgcagcctctatacaacctgggacgggagacagactgctgaacctggtagatggagcctaccaagcactcaacctcacc
Mpmv5        gcgcagcctctatacaacctgggacgggagacagactgctgaacctggtagatAgagcctaccaagcactcaacctcacc
Mpmv6        gcgcagcctctatacaacctgggacgggagacagactgctgaacctggtagatggagcctaccaagcactcaacctcacc
Mpmv7        gcgcagcctctatacaacctgggacgggagacagactgctgaacctggtagatggagcctaccaagcactcaacctcacc
Mpmv8        gcgcagcctctatacaacctgggacgggagacagactgctgaacctggtagatggagcctaccaagcactcaacctcacc
Mpmv9        gcgcagcctctatacaacctgggacgggagacagactgctgaacctggtagatggagcctaccaagcactcaacctcacc
```

```
MPMV_CONS     AGTCCTGACAAAACCCAAGAGTGCTGGTTGTGTCTGGTATCGGGACCCCCCTATTACGAAGGGGTTGCCGTCCTAGGTAC
Mpmv1         agtcctgacaaaacccaagagtgctggttgtgtctggtatcgggacccccctattacgaaggggttgccgtcctaggtac
Mpmv10        agtcctgacaaaacccaagagtgctggttgtgtctggtatcgggacccccctattacgaaggggttgccgtcctaggtac
Mpmv11        agtcctgacaaaacccaagagtgctggttgtgtctggtatcgggacccccctattacgaaggggttgccgtcctaggtac
Mpmv12        agtcctgacaaaacccaagagtgctggttgtgtctggtatcgggacccccctattacgaaggggttgccgtcctaggtac
Mpmv13        agtcctgacaaaacccaagagtgctggttgtgtctggtatcgggacccccctattacgaaggggttgccgtcctaggtac
Mpmv2         agtcctgacaaaacccaagagtgctggttgtgtctggtatcgggacccccctattacgaaggggttgccgtcctaggtac
Mpmv3         agtcctgacaaaacccaagagtgctggttgtgtctggtatcgggacccccctattacgaaggggttgccgtcctaggtac
Mpmv4         agtcctgacaaaacccaagagtgctggttgtgtctggtatcgggacccccctattacgaaggggttgccgtcctaggtac
Mpmv5         agtcctgacaaaacccaagagtgctggttgtgtctggtatcgggacccccctattacgaaggggttgccgtcctaggtac
Mpmv6         agtcctgacaaaacccaagagtgctggttgtgtctggtatcgggacccccctattacgaaggggttgccgtcctaggtac
Mpmv7         agtcctgacaaaacccaagagtgctggttgtgtctggtatcgggacccccctattacgaaggggttgccgtcctaggtac
Mpmv8         agtcctgacaaaacccaagagtgctggttgtgtctggtatcgggacccccctattacgaaggggttgccgtcctaggtac
Mpmv9         agtcctgacaaaacccaagagtgctggttgtgtctggtatcgggacccccctattacgaaggggttgccgtcctaggtac

MPMV_CONS     CTACTCCAACCATACCTCTGCCCCAGCTAACTGCTCCGTGGCCTCCCAACACAAGCTGACCCTGTCCGAAGTAACCGGAC
Mpmv1         ctactccaaccatacctctgccccagctaactgctccgtggcctcccaacacaagctgaccctgtccgaagtaaccggac
Mpmv10        ctactccaaccatacctctgccccagctaactgctccgtggcctcccaacacaagctgaccctgtccgaagtaaccggac
Mpmv11        ctactccaaccatacctctgccccagctaactgctccgtggcctcccaacacaagctgaccctgtccgaagtaaccggac
Mpmv12        ctactccaaccatacctctgccccagctaactgctccgtggcctcccaacacaagctgaccctgtccgaagtaaccggac
Mpmv13        ctactccaaccatacctctgccccagctaactgctccgtggcctcccaacacaagctgaccctgtccgaagtaaccggac
Mpmv2         ctactccaaccatacctctgccccagctaactgctccgtggcctcccaacacaagctgaccctgtccgaagtaaccggac
Mpmv3         ctactccaaccatacctctgccccagctaactgctccgtggcctcccaacacaagctgaccctgtccgaagtaaccggac
Mpmv4         ctactccaaccatacctctgccccagctaactgctccgtggcctcccaacacaagctgaccctgtccgaagtaaccggac
Mpmv5         ctactccaaccatacctctgccccagctaactgctccgtggcctcccaacacaagctgaccctgtccgaagtaaccggac
Mpmv6         ctactccaaccatacctctgccccagctaactgctccgtggcctcccaacacaagctgaccctgtccgaagtaaccggac
Mpmv7         ctactccaaccatacctctgccccagctaactgctccgtggcctcccaacacaagctgaccctgtccgaagtaaccggac
Mpmv8         ctactccaaccatacctctgccccagctaactgctccgtggcctcccaacacaagctgaccctgtccgaagtaaccggac
Mpmv9         ctactccaaccatacctctgccccagctaactgctccgtggcctcccaacacaagctgaccctgtccAaagtaaccggac

MPMV_CONS     AGGGACTCTGCGTAGGAGCAGTTCCCAAAACCCATCAGGCCCTGTGTAATACCACCCAGAATACAAGCGACGGGTCCTAC
Mpmv1         agggactctgcgtaggagcagttcccaaaacccatcaggccctgtgtaataccacccagaatacaagcgacgggtcctac
Mpmv10        agggactctgcgtaggagcagttcccaaaacccatcaggccctgtgtaataccacccagaatacaagcgacgggtcctac
Mpmv11        agggactctgcgtaggagcagttcccaaaacccatcaggccctgtgtaataccacccagaatacaagcgacgggtcctac
Mpmv12        agggactctgcgtaggagcagttcccaaaacccatcaggccctgtgtaataccacccagaatacaagcgacgggtcctac
Mpmv13        agggactctgcgtaggagcagttcccaaaacccatcaggccctgtgtaataccacccagaatacaagcgacgggtcctac
Mpmv2         agggactctgcgtaggagcagttcccaaaacccatcaggccctgtgtaataccacccagaatacaagcgacgggtcctac
Mpmv3         agggactctgcgtaggagcagttcccaaaacccatcaggccctgtgtaataccacccagaatacaagcgacgggtcctac
Mpmv4         agggactctgcgtaggagcagttcccaaaacccatcaggccctgtgtaataccacccagaatacaagcgacgggtcctac
Mpmv5         agggactctgcgtaggagcagttcccaaaacccatcaggccctgtgtaataccacccaAaatacaagcgacgggtcctac
Mpmv6         agggactctgcgtaggagcagttcccaaaacccatcaggccctgtgtaataccacccagaatacaagcgacgggtcctac
Mpmv7         agggactctgcgtaggagcagttcccaaaacccatcaggccctgtgtaataccacccagaatacaagcgacgggtcctac
Mpmv8         agggactctgcgtaggagcagttcccaaaacccatcaggccctgtgtaataccacccagaatacaagcgacgggtcctac
Mpmv9         agggactctgcgtaggagcagttcccaaaacccatcaggccctgtgtaataccacccagaatacaagcgacgggtcctac

MPMV_CONS     TATCTGGCTGCTCCCGCCGGGACCATTTGGGCTTGCAACACCGGGCTCACTCCCTGCCTATCTACTACTGTACTCAACCT
Mpmv1         tatctggctgctcccgccgggaccatttgggcttgcaacaccgggctcactccctgcctatctactactgtactcaacct
Mpmv10        tatctggctgctcccgccgggaccatttgggcttgcaacaccgggctcactccctgccta--------------------
Mpmv11        tatctggctgctcccgccgggaccatttgggcttgcaacaccgggctcactccctgcctatctactactgtactcaacct
Mpmv12        tatctggctgctcccgccgggaccatttgggcttgcaacaccgggctcactccctgcctatctactactgtactcaacct
Mpmv13        tatctggctgctcccgccgggaccatttgggcttgcaacaccgggctcactccctgcctatctactactgtactcaacct
Mpmv2         tatctggctgctcccgccgggaccatttgggcttgcaacaccgggctcactccctgcctatctactactgtactcaacct
Mpmv3         tatctggctgctcccgccgggaccatttgggcttgcaacaccgggctcactccctgcctatctactactgtactcaacct
Mpmv4         tatctggctgctcccgccgggaccatttgggcttgcaacaccgggctcactccctgcctatctactactgtactcaacct
Mpmv5         tatctggctgctcccgccgggaccatttgggcttgcaacaccgggctcactccctgcctatctactactgtactcaacct
Mpmv6         tatctggctgctcccgccgggaccatttgggcttgcaacaccgggctcactccctgcctatctactactgtactcaacct
Mpmv7         tatctggctgctcccgccgggaccatttgggcttgcaacaccgggctcactccctgcctatctactactgtactcaacct
Mpmv8         tatctggctgctcccgccgggaccatttgggcttgcaacaccgggctcactccctgcctatctactactgtactcaacct
Mpmv9         tatctggctgctcccgccgggaccatttgggcttgcaacaccgggctcactccctgcctatctactactgtactcaacct
```

```
MPMV_CONS     CACCACCGATTACTGTGTCCTGGTTGAGCTCTGGCCAAAGGTGACCTACCACTCCCCTGGTTATGTTTATGGCCAGTTTG
Mpmv1         caccaccgattactgtgtcctggttgagctctggccaaaggtgacctaccactcccctggttatgtttatggccagtttg
Mpmv10        --------------------------------------------------------------------------------
Mpmv11        caccaccgattactgtgtcctggttgagctctggccaaaggtgacctaccactcccctggttatgtttatggccagtttg
Mpmv12        caccaccgattactgtgtcctggttgagctctggccaaaggtgacctaccacGcccctggttatgtttatggccagtttg
Mpmv13        caccaccgattactgtgtcctggttgagctctggccaaaggtgacctaccactcccctggttatgtttatggccagtttg
Mpmv2         caccaccgattactgtgtcctggttgagctctggccaaaggtgacctaccactcccctggttatgtttatggccagtttg
Mpmv3         caccaccgattactgtgtcctggttgagctctggccaaaggtgacctaccactcccctggttatgtttatggccagtttg
Mpmv4         caccaccgattactgtgtcctggttgagctctggccaaaggtgacctaccactcccctggttatgtttatggccagtttg
Mpmv5         caccaccgattactgtgtcctggttgagctctggccaaaAgtgacctaccactcccctggttatgtttatggccagtttg
Mpmv6         caccaccgattactgtgtcctggttgagctctggccaaaggtgacctaccactcccctggttatgtttatggccagtttg
Mpmv7         caccaccgattactgtgtcctggttgagctctggccaaaggtgacctaccactcccctggttatgtttatggccagtttg
Mpmv8         caccaccgattactgtgtcctggttgagctctggccaaaggtgacctaccactcccctggttatgtttatggccagtttg
Mpmv9         caccaccgattactgtgtcctggttgagctctggccaaaggtgacctaccactcccctggttatgtttatggccagtttg

MPMV_CONS     AGAGAAAAACCAAATATAAAAGAGAGCCGGTGTCATTAACTCTGGCCCTGCTGTTGGGAGGACTTACTATGGGCGGCATA
Mpmv1         agagaaaaaccaaatataaaagagagccggtgtcattaactctggccctgctgttgggaggacttactatgggcggcata
Mpmv10        -----------------aaaagagagccggtgtcattaactctggccctgctgttgggaggacttactatgggcggcata
Mpmv11        agagaaaaaccaaatataaaagagagccggtgtcattaactctggccctgctgttgggaggacttactatgggcggcata
Mpmv12        agagaaaaaccaaatataaaagagagccggtgtcattaactctggccctgctgttgggaggacttactatgggcggcata
Mpmv13        agagaaaaaccaaatataaaagagagccggtgtcattaactctggccctgctgttgggaggacttactatgggcggcata
Mpmv2         agagaaaaaccaaatataaaagagagccggtgtcattaactctggccctgctgttgggaggacttactatgggcggcata
Mpmv3         agagaaaaaTcaaatataaaagagagccggtgtcattaactctggccctgctgttgggaggacttactatgggcggcata
Mpmv4         agagaaaaaccaaatataaaagagagccggtgtcattaactctggccctgctgttgggaggacttactatgggcggcata
Mpmv5         agagaaaaaccaaatataaaagagagccggtgtcattaactctggccctgctgttgggaggacttactatgggcggcata
Mpmv6         agagaaaaaccaaatataaaagagagccggtgtcattaactctggccctgctgttgggaggacttactatgggcggcata
Mpmv7         aAagaaaaaccaaatataaaagagagccggtgtcattaactctggccctgctgttgggaggacttactatgggcggcata
Mpmv8         agagaaaaaccaaatataaaagagagccggtgtcattaactctggccctgctgttgggaggacttactatgggcggcata
Mpmv9         agagaaaaaccaaatataaaagagagccggtgtcattaactctggccctgctgttgggaggacttactatgggcggcata

MPMV_CONS     GCTGCAGGAGTAGGAACAGGGACTACAGCCCTAGTGGCCACCAAGCAATTCGAGCAGCTCCAGGCAGCCATACATACAGA
Mpmv1         gctgcaggagtaggaacagggactacagccctagtggccaccaagcaattcgagcagctccaggcagccatacatacaga
Mpmv10        gctgcaggagtaAgaacagggactacagccctagtggccaccaagcaattcgagcagctccaggcagccatacatacaga
Mpmv11        gctgcaggagtaggaacagggactacagccctagtggccaccaagcaattcgagcagctccaggcagccatacatacaga
Mpmv12        gctgcaggagtaggaacagggactacagccctagtggccaccaagcaattcgagcagctccaggcagccatacatacaga
Mpmv13        gcC----------------------------------------------tc-agcagctTcaTgca--c-tGc--acTga
Mpmv2         gctgcaggagtaggaacagggactacagccctagtggccaccaagcaattcgagcagctccaggcagccatacatacaga
Mpmv3         gctgcaggagtaggaacagggactacagccctagtggccaccaagcaattcgagcagctccaggcagccatacatacaga
Mpmv4         gctgcaggagtaggaacagggactacagccctagtggccaccaagcaattcgagcagctccaggcagccatacatacaga
Mpmv5         gctAcaggagtaAAaacagggactacagccctagtggccaccaagcaattcgagcagctccaggcagccatacatacaga
Mpmv6         gctgcaggagtaggaacagggactacagccctagtggccaccaagcaattcgagcagctccaggcagccatacatacaga
Mpmv7         gctgcaggagtaggaacagggactacagccctagtggccaccaagcaattcgagcagctccaggcagccatacatacaga
Mpmv8         gctgcaggagtaggaacagggactacagccctagtggccaccaagcaattcgagcagctccaggcagccatacatacaga
Mpmv9         gctgcaggagtaAgaacagggactacagccctagtggccaccaagcaattcgagcagctccaggcagccatacatacaga

MPMV_CONS     CCTTGGGGCCTTAGAAAAATCAGTCAGTGCCCTAGAAAAGTCTCTGACCTCGTTGTCTGAGGTGGTCCTACAGAACCGGA
Mpmv1         ccttggggccttaAaaaaatcagtcagtgccctagaaaagtctctgacctcgttgtctgaggtggtcctacagaaccgga
Mpmv10        ccttggggccttagaaaaatcagtcagtgccctagaaaagtctctgacctcgttgtctgaggtggtcctacagaaccgga
Mpmv11        ccttggggccttagaaaaatcagtcagtgccctaAaaaagtctctgacctcgttgtctgaggtggtcctacagaaccgga
Mpmv12        ccttggggccttagaaaaatcagtcagtgccctagaaaagtctctgacctcgttgtctgaggtggtcctacagaaccgga
Mpmv13        c--tgg---------------agtcagtgccctagaaaagtctctgacctcgttgtctgaggtggtcctacagaaccgga
Mpmv2         ccttggggccttagaaaaatcagtcagtgccctagaaaagtctctgacctcgttgtctgaggtggtcctacagaaccgga
Mpmv3         ccttggggccttagaaaaatcagtcagtgccctagaaaagtctctgacctcgttgtctgaggtggtcctacagaaccgga
Mpmv4         ccttggggccttaAaaaaatcagtcagtgccctagaaaagtctctgacctcgttgtctgaggtggtcctacagaaccgga
Mpmv5         ccttggggccttagaaaaatcagtcagtgccctaAaaaagtctctgacctcgttgtctgaggtggtcctacagaaccgga
Mpmv6         ccttggggccttagaaaaatcagtcagtgccctagaaaagtctctgacctcgttgtcCgaggtggtcctacagaaccgga
Mpmv7         ccttggggccttagaaaaatcagtcagtgccctagaaaagtctctgacctcgttgtctgaggtggtcctacagaaccgga
Mpmv8         ccttggggccttagaaaaatcagtcagtgccctagaaaagtctctgacctcgttgtctgaggtggtcctacagaaccgga
Mpmv9         ccttggggccttagaaaaatcagtcagtgccctagaaaagtctctgacctcgttgtctgaggtggtcctacagaaccgga
```

```
MPMV_CONS     GAGGATTAGATCTACTGTTCCTAAAAGAAGGAGGATTATGTGCTGCCCTAAAAGAAGAATGCTGTTTCTACGCGGACCAC
Mpmv1         gaggattagatctactgttcctaaaagaaggaggattatgtgctgccctaaaagaagaatgctgtttctacgcggaccac
Mpmv10        gaggattagatctactgttcctaaaagaaggaggattatgtgctgccctaaaagaagaatgctgCttctacgcggaccac
Mpmv11        gaggattagatctactgttcctaaaagaaggaggattatgtgctgccctaaaagaagaatgctgtttctacgcggaccac
Mpmv12        gaAgattagatctactgttcctaaaagaaggaggattatgtgctgccctaaaagaagaatgctgtttctacgcggaccac
Mpmv13        gaggattagatctactgttcctaaaaAaaggaAgattatgtgctgccctaaaagaagaatgctgtttctacgcggaccac
Mpmv2         gaggattagatctactgttcctaaaagaaggaggattatgtgctgccctaaaagaagaatgctgtttctacgcggaccac
Mpmv3         gaggattagatctactgttcctaaaagaaggaggattatgtgctgccctaaaagaagaatgctgtttctacgcggaccac
Mpmv4         gaggattagatctactgttcctaaaagaaggaggattatgtgctgccctaaaagaagaatgctgtttctacgcggaccac
Mpmv5         gaggattagatctactgttcctaaaaAaaggaAgattatgtgctgccctaaaagaagaatgctgtttctacgcggaccac
Mpmv6         gaggattagatctactgttcctaaaagaaggaggattatgtgctgccctaaaagaagaatgctgtttctacgcggaccac
Mpmv7         gaggattagatctactgttcctaaaagaaggaggattatgtgctgccctaaaagaagaatgctgtttctacgcggaccac
Mpmv8         gaAgattagatctactgttcctaaaagaaggaggattatgtgctgccctaaaagaagaatgctgtttctacgcggaccac
Mpmv9         gaggattagatctactgttcctaaaagaaggaggattatgtgctgccctaaaagaagaatgctgtttctacgcggaccac

MPMV_CONS     ACTGGCGTAGTGAGAGATAGCATGGCAAAGCTAAGAGAAAGGTTAAACCAGAGACAAAAATTGTTCGAATCAGGACAAGG
Mpmv1         actggcgtagtgagagatagcatggcaaagctaagagaaaggttaaaccagagacaaaaattgttcgaatcaggacaagg
Mpmv10        actggcgtagtgagagatagcatggcaaagctaagagaaaggttaaaccagagacaaaaattgttcgaatcaggacaagg
Mpmv11        actggcgtagtgagagatagcatggcaaagctaagagaaaggttaaaccagagacaaaaattgttcgaatcaggacaagg
Mpmv12        actggcgtagtgagagatagcatggcaaagctaagagaaaggttaaaccagagacaaaaattgttcgaatcaggacaagg
Mpmv13        actggcgtagtgagagatagcatggcaaagctaagagaaaggttaaaccagagacaaaaattgttcgaatcaggacaagg
Mpmv2         actggcgtagtgagagatagcatggcaaagctaagagaaaggttaaaccagagacaaaaattgttcgaatcaggacaagg
Mpmv3         actggcgtagtgagagatagcatgAcaaagctaagagaaaggttaaaccagagacaaaaattgttcgaatcaggacaagg
Mpmv4         actggcgtagtgagagatagcatggcaaagctaagagaaaggttaaaccagagacaaaaattgttcgaatcaggacaagg
Mpmv5         actggcgtagtgagagatagcatggcaaagctaagaAaaaggttaaaccagagacaaaaattgttcgaatcaggacaagg
Mpmv6         actggcgtagtgagagatagcatggcaaagctaagagaaaggttaaaccagagacaaaaattgttcgaatcaggacaagg
Mpmv7         actggcgtagtgagagatagcatggcaaagctaagagaaaggttaaaccagagacaaaaattgttcgaatcaggacaagg
Mpmv8         actggcgtagtgagagatagcatggcaaagctaagagaaaggttaaaccagagacaaaaattgttcgaatcaggacaagg
Mpmv9         actggcgtagtgagagatagcatggcaaagctaagagaaaggttaaaccagagacaaaaattgttcgaatcaggacaagg

MPMV_CONS     GTGGTTTGAGGGACTGTTTAACAGGTCCCCATGGTTCACGACCTTGATATCCACCATTATGGGCCCCTTGATAATACTCT
Mpmv1         gtggtttgagggactgtttaacaggtccccatggttcacgaccttAatatccaccattatgggccccttgataatactct
Mpmv10        gtggtttgagggactgtttaacaggtccccatggttcacgaccttgatatccaccattatgggccccttgataatactct
Mpmv11        gtggtttgagggactgtttaacaggtccccatggttcacgaccttgatatccaccattatgggccccttgataatactct
Mpmv12        gtggtttgagggactgtttaacaggtccccatggttcacgaccttgatatccaccattatgggccccttgataatactct
Mpmv13        gtggtttgagggactgtttaacaggtccccatggttcacgaccttgatatccaccattatgggccccttgataatactct
Mpmv2         gtggtttgagggactgtttaacaggtccccatggttcacgaccttgatatccaccattatgggccccttgataatactct
Mpmv3         gtggtttgagggactgtttaacaggtccccatggttcacgaccttgatatccaccattatgggccccttgataatactct
Mpmv4         gtggtttgagggactgtttaacaggtccccatggttcacgaccttgGtatccaccattatgggccccttgataatactct
Mpmv5         gtggtttgagggactgtttaacaggtccccatAgttcacgaccttgatatccaccattatgggccccttgataatactct
Mpmv6         gtggtttgagggactgtttaacaggtccccatggttcacgaccttgatatccaccattatgggccccttgataatactct
Mpmv7         gtggtttgagggactgtttaacaggtccccatggttcacgaccttgatatccaccattatgggccccttgataatactct
Mpmv8         gtggtttgagggactgtttaacaggtccccatggttcacgaccttgGtatccaccattatgggccccttgataatactct
Mpmv9         gtggtttgagggactgtttaacaggtccccatggttcacgaccttgatatccaccattatgggccccttgataatactct

MPMV_CONS     TATTAATCCTACTCTTCGGACCCTGTATTCTCAACCGCTTGGTCCAGTTTGTAAAAGACAGAATTTCGGTGGTG
Mpmv1         tattaatcctactcttcggaccctgtattctcaaccgcttggtccagtttgtaaaagacagaatttcggtggtg
Mpmv10        tattaatcctactcttcggaccctgtattctcaaccgcttggtccagtttgtaaaagacagaatttcggtggtg
Mpmv11        tattaatcctactcttcggaccctgtattctcaaccgcttggtccagtttgtaaaagacagaatttcggtggtg
Mpmv12        tattaatcctactcttcggaccctgtattctcaaccgcttggtccagtttgtaaaagacagaatttcggtggtg
Mpmv13        tattaatcctactcttcggaccctgtattctcaaccgcttggtccagtttgtaaaagacagaatttcggtggtg
Mpmv2         tattaatcctactcttcggaccctgtattctcaaccgcttggtccagtttgtaaaagacagaatttcggtggtg
Mpmv3         tattaatcctactcttcggaccctgtattctcaaccgcttggtccagtttAtaaaagacagaatttcggtggtg
Mpmv4         tattaatcctactcttcggaccctgtattctcaaccgcttggtccagtttgtaaaagacagaatttcggtggtg
Mpmv5         tattaatcctactcttcggaccctgtattctcaaccgcttggtccagtttgtaaaagacagaatttcggtggtg
Mpmv6         tattaatcctactcttcggaccctgtattctcaaccgcttggtccagtttgtaaaagacagaatttcggtggtg
Mpmv7         tattaatcctactcttcggaccctgtattctcaaccgcttggtccagtttgtaaaagacagaatttcggtggtg
Mpmv8         tattaatcctactcttcggaccctgtattctcaaccgcttggtccagtttgtaaaagacagaatttcggtggtg
Mpmv9         tattaatcctactcttcggaccctgtattctcaaccgcttggtccagtttgtaaaagacagaatttcggtggtg
```

Xmv (concatenated *gag*, *pol* and *env*)

```
XMV_CONS    ATGGGACAGACCGTAACYACYCCTYTGAGTCTRACCYTDSARCACTGGGGAGAYGTCCAGCGCATTGCRTCCAAYCAGTC
Xmv10       atgggacagaccgtaacCacTcctTtgagtctGaccCtAGaAcactggggagaCgtccagcgcattgcGtccaaCcagtc
Xmv12       atgggacagaccgtaacTacCcctCtgagtctAaccTtGCaGcactggggagaTgtccagcgcattgcAtccaaTcagtc
Xmv13       atgggacagaccgtaacCacTcctTtgagtctGaccCtAGaAcactggggagaCgtccagcgcattgcGtccaaCcagtc
Xmv15       atgggacagaccgtaacTacCcctCtgagtctAaccTtGCaGcactggggagaTgtccagcgcattgcAtccaaTcagtc
Xmv16       --------------------------------------------------------------------------------
Xmv17       atgggacagaccgtaacTacCcctCtgagtctAaccTtGCaGcactggggagaTgtccagcgcattgcAtccaaTcagtc
Xmv18       atgggacagaccgtaacTacCcctCtgagtctAaccTtGCaGcactggggagaTgtccagcgcattgcAtccaaTcagtc
Xmv19       --------------------------------------------------------------------------------
Xmv41       atgggacagacTgtaacCacTcctTtgagCctGaccCtTGaAcactggggagaCgtccagcgcattgcGtccaaCcagtc
Xmv42       atgggacagaccgtaacTacCcctCtgagtctAaccTtGCaGcactggggagaTgtccagcgcattgcAtccaaTcagtc
Xmv43       atgggacagaccgtaacCacCcctCtgagtctGaccCtAGaAcactggggagaCgtccagcgcatCgcGtccaaCcagtc
Xmv8        atgggacagaccgtaacCacTcctTtgagtctGaccCtAGaAcactggggagaCgtccagcgcattgcGtccaaCcagtc
Xmv9        atgggGcagaccgtaacCacTcctTtgagCctGaccCtAGaAcactggggagaCgtccagcgcattgcGtccaaCcagtc

XMV_CONS    YGTGGAYGTCARGAAGAGRCGCTGGGTYACCTTCTGYTCYGCCGARTGGCCAACTTTCAATGTGGGRTGGCCDCARGATG
Xmv10       CgtggaCgtcaAgaagagAcgTtgggtCaccttctgCtcTgccgaGtggccaactttcGatgtgggGtggccGcaAgatg
Xmv12       TgtggaTgtcaGgaagagGcgctgggtTaccttctgTtcCgccgaAtggccaactttcaatgtgggAtggccTcaGgatg
Xmv13       CgtggaCgtcaAgaagagAcgTtgggtCaccttctgCtcTgccgaAtggccaactttcaatgtgggAtggccTcaGgatg
Xmv15       TgtggaTgtcaGgaagagGcgctgggtTaccttctgTtcCgccgaAtggccaactttcaatgtgggAtggccTcaGgatg
Xmv16       --------------------------------------------------------------------------------
Xmv17       TgtggaTgtcaGgaagagGcgctgggtTaccttctgTtcCgccgaAtggccaactttcaatgtgggAtggccTcaGgatg
Xmv18       TgtggaTgtcaGgaagagGcgctgggtTaccttctgTtcCgccgaAtggccaactttcaatgtgggAtggccTcaGgatg
Xmv19       --------------------------------------------------------------------------------
Xmv41       CgtggaCgtcaAgaagagAcgctgggtCaccttctgCtcTgccgaGtggccaactttcGatgtgggGtggccAcaAgatg
Xmv42       TgtggaTgtcaGgaagagGcgctgggtTaccttctgTtcCgccgaAtggccaactttcaatgtgggAtggccTcaGgatg
Xmv43       CgtggaCgtcaAgaagagAcgctgggtCaccttctgCtcTgccgaGtggccaactttcGGtgtAggGtggccGcaAgatg
Xmv8        CgtggaCgtcaAgaagagAcgTtgggtCaccttctgCtcTgccgaGtggccaactttcaatgtgggGtggccGcaAgatg
Xmv9        CgtggaCgtcaAgaagagAcATtgggtCaccttctgCtcTgccgaGtggccaactttcGatgtgggGtggccGcaAgatg

XMV_CONS    GTACTTTTAATTTRGRYATTATYTYWCAGGTYAARTCTARRGTGTTYTSTCCYGGTCCCCACGGACACCCGGATCAGGTC
Xmv10       gtacttttaatttGgACattatTtTAcaggtTaaAtctaAGgtgttCtCtccCggtccccacggacacccggatcaggtc
Xmv12       gtacttttaatttAgGTattatCtCTcaggtCaaGtctaGAgtgttTtGtccTggtccccacggacacccggatcaggtc
Xmv13       gtacttttaatttGgACattatTtTAcaggtTaaAtctaAGgtgttCtCtccCggtccccacggacacccggatcaggtc
Xmv15       gtacttttaatttAgGTattatCtCTcaggtCaaGtctaGAgtgttTtGtccTggtccccacggacacccggatcaggtc
Xmv16       --------------------------------------------------------------------------------
Xmv17       gtacttttaatttAgGTattatCtCTcaggtCaaGtctaGAgtgttTtGtccTggtccccacggacacccggatcaggtc
Xmv18       gtacttttaatttAgGTattatCtCTcaggtCaaGtctaGAgtgttTtGtccTggtccccacggacacccggatcaggtc
Xmv19       --------------------------------------------------------------------------------
Xmv41       gtacttttaatttAgACatCatTtCAcaggtTaaAtctaAGgtgttCtCtccCggtccccacggacacccggatcaggtc
Xmv42       gtacttttaatttAgGTattatCtCTcaggtCaaGtctaGAgtgttTtGtccTggtccccacggacacccggatcaggtc
Xmv43       gtacttttaatttGgACattatTtTAcaggtTaaAtctaAGgtgttCtCtccCggtccccacggacacccggatcaggtc
Xmv8        gtacttttaatttGgACattatTtTAcaggtTaaAtctaAGgtAttCtCtccCggtccccacggacacccggatcaggtc
Xmv9        gtacttttaatttGgACattatTtTAcaggtTaaAtctaAGgtgttCtCtccCggtccccacggacacccggatcaggtc

XMV_CONS    CCATAYATYGTCACCTGGGAGGCACTTGCCTATGACCCCCCTCCGTGGGTCAAACCGTTTGTCTCTCCAAAACCCCCTCC
Xmv10       ccataCatTgtcacctgggaggcacttgcctatgacccccctccgtgggtcaaaccgtttgtctctccaaaaccccctcc
Xmv12       ccataTatCgtcacctgggaggcacttgcctatgacccccctccgtgggtcaaaccgtttgtctcCccaaaaccccctcc
Xmv13       ccataCatTgtcacctgggaggcacttgcctatgacccccctccgtgggtcaaaccgtttgtctctccaaaaccccctcc
Xmv15       ccataTatCgtcacctgggaggcacttgcctatgacccccctccgtgggtcaaaccgtttgtctctccaaaaccccctcc
Xmv16       --------------------------------------------------------------------------------
Xmv17       ccataTatCgtcacctgggaggcacttgcctatgacccccctccgtgggtcaaaccgtttgtctctccaaaaccccctcc
Xmv18       ccataTatCgtcacctgggaggcacttgcctatgacccccctccgtgggtcaGaccgtttgtctctccaaaaccccctcc
Xmv19       --------------------------------------------------------------------------------
Xmv41       ccataCatTgtcacctgggaggcTAttgcctatgacccccctccgtgggtcaaaccTtttgtctctccCaaacTcTctcT
Xmv42       ccataTatCgtcacctgggaggcacttgcctatgacccccctccgtgggtcaaaccgtttgtctctccaaaaccccctcc
Xmv43       ccataCatTgtcacctgggaggcTAttgcctatgaAccccctccgtgggtcaaaccTtttgtctctccCaaacTcTcCcT
Xmv8        ccataCatTgtcacctgggaggcacttgcctatgacccccctccgtgggtcaaaccgtttgtctctccaaaaccccctcc
Xmv9        ccataCatTgtcacctgggaggcacttgcctatgacccccctccgtgggtcaaaccgtttgtctctccaaaaccccctcc
```

```
XMV_CONS    TTTACCGACAGCTCCCGTCCTCCCGCCCGGTCCTTCTGCGCAACCTCCGTCCCGATCTGCCCTTTACCCTGCCCTTACCC
Xmv10       tttaccgacagctcccgtcctcccgcccggtccttctgcgcaacctccgtcccgatctgccctttaccctgcccttaccc
Xmv12       tttaccgacagctcccgtcctcccgcccggtccttctgcgcaacctccgtcccgatctgccctttaccctgcccttaccc
Xmv13       Cttaccgacagctcccgtcctcccgcccggtccttctgcgcaacctccgtcccgatctgccctttaccctgcccttaccc
Xmv15       tttaccgacagctcccgtcctcccgcccggtccttctgcgcaacctccgtcccgatctgccctttaccctgcccttaccc
Xmv16       --------------------------------------------------------------------------------
Xmv17       tttaccgacagctcccgtcctcccgcccggtccttctgcgcaacctccgtcccgatctgccctttaccctgcccttaccc
Xmv18       tttaccgacagctcccgtcctcccgcccggtccttctgcgcaacctccgtcccgatctgccctttaccctgcccttaccc
Xmv19       --------------------------------------------------------------------------------
Xmv41       CtCTccAacCgctcccAtcctcccATccggCccttcGAcCcaacctccgCcccgatctgccctttaccctgcTcttaccc
Xmv42       ttCaccgacagctcccgtcctcccgcccgAtccttctgcgcaacctccgtcccgatctgccctttaccctgcccttaccc
Xmv43       CtCTccAacCgctcccAtcctcccATccggtccttcGAcCcaacctccgCcccgatctgccctttaccctgcTcttaccc
Xmv8        tttaccgacagctcccgtcctcccgcccggtccttctgcgcaacctccgtcccgatctgccctttaccctgcccttaccc
Xmv9        tttaccgacagctcccgtcctcccgcccggtccttctgcgcaacctccgtcccgatctgccctttaccctgcccttaccc

XMV_CONS    CCTCTATAAAGCCCAAACCTCCTAAGCCCCAGGTTCTCCCTGATAACGGCGGACCTCTCATTGACCTTCTCACAGAGGAC
Xmv10       cctctataaagAccaaacctcctaagccccaggttctccctgataacggcggaccGctcattgaccttctcacagaggac
Xmv12       cctctataaagcccaaacctcctaagccccaggttctccctgataacggcggacctctcattgaccttctcacagaggac
Xmv13       cctctataaagTccaaacctcctaagccccaggttctccctgataGcggcggacctctcattgaccttctcacagaggac
Xmv15       cctctataaagcccaaacctcctaagccccaggttctccctgataacggcggacctctcattgaccttctcacagaggac
Xmv16       --------------------------------------------------------------------------------
Xmv17       cctctataaagcccaaacctcctaagccccaggttctccctgataacggcggacctctcattgaccttctcacagaggac
Xmv18       cctctataaagcccaaacctcctaagccccaggttctccctgataacggcggacctctcattgaccttctcacagaggac
Xmv19       -----------------------------------------------------------------------------ga-
Xmv41       cctctataaaAcccaGacctTctaaAccTcaggttctcTcCgataacggcggacctctcattgaccttctcacagaAgac
Xmv42       cctctataaagTccaaacctcctaagccccaggttctccctgataGcggcggaccCctcattgaccttctcacagaggac
Xmv43       cctctataaaAcccaGacctTctaaAccTcaggttctcTcCgataaTggcggacctctcattgaccttctcacagaAgac
Xmv8        cctctataaagcccaaacctcctaagccccaggttctccctgataGcggcggacctctcattgaccttctcacagaggac
Xmv9        cctctataaagcccaaacctcctaagccccaggttctccctgatGGcggcggacctctcattgaccttctcacagaggac

XMV_CONS    CCCCCGCCGTACGGAGCACAA---CCTTCCTCCTCTGCCAGAGGGAACGATGAAGAAGAGGCGGCCDCCACCTCCGAGGT
Xmv10       cccccgccgtacggagcacaa---ccttcctcctctgccagagggaacgatgaagaagaggcggccGccacctccgaggt
Xmv12       cccccgccgtacggagcacaa---ccttcctcctctgccagagggaacgatAaagaagaggcggccAccacctccgaggt
Xmv13       cccccgccgtacggagcacaa---ccttcctcctctgccagagAAaacAatgaagaagaggcggccGccacctccgaggt
Xmv15       cccccgccgtacggagcacaa---ccttcctcctctgccagagggaacgatgaagaagaggcggccAccacctccgaggt
Xmv16       --------------------------------------------------------------------------------
Xmv17       cccccgccgtacggagcacaa---ccttcctcctctgccagagggaacgatAaagaagaggcggccAccacctccgaggt
Xmv18       cccccgccAtacggagcacaa---ccttcctcctctgccagagggaacgatgaagaagaggcggccAccaccCccgaggt
Xmv19       cccccgccgtacggagcacaa---ccttcctcctctgccagagggaacgatgaagaagaggcggccAccacctccgaggt
Xmv41       ccTccgccgtacggagAacaGGGAccGtcctcctctgAcGgagATGGcgaCAGagaagaggcCAccTccacTtcTgagAt
Xmv42       cccccgccgtacAgagcacaa---ccCtcctcctctgccagGgAgaacgaCgaagaagaggcggccAcc---tccgaggt
Xmv43       ccTccgccgtacggagAacaGGGAccGtcctcctctgAcGgagATGGcgaCAGagaagaggcCAccTccacTCcTgagAt
Xmv8        cccccgccgtacggagcacaa---ccttcctcctctgccagagAAaacAatgaagaagaggcggccGccacctccgaggt
Xmv9        cccccgccgtacggagcacaa---ccttcctcctctgccagagggaacgatgaaAaagaggcggccGccacctccgaggC

XMV_CONS    TTCCCCCCCTTCTCCCATGGTGTCTCGACTGCGGGGAAGGAGAGACCCTCCCGCAGCGGACTCCACCTCCTCCCAGGCAT
Xmv10       ttcccccccttctcccatggtgtctcgactgcggggaaggagGgaccctcccgcagcggactccacctcctcccaggcat
Xmv12       ttcccccccttctcccatggtgtctcgactgcggggaaggagagaccctcccgcagcggactccacctcctcccaggcat
Xmv13       ttcccccccttctcccatggtgtctcgactgcggggaaggagGgaccctcccgcagcggactccacctcctcccaggcat
Xmv15       ttcccccccttctcccatggtgtctcgactgcggggaaggagagaccctcccgcagcggactccacctcctcccaggcat
Xmv16       --------------------------------------------------------------------------------
Xmv17       ttcccccccttctcccatggtgtctcgactgcggggaaggagagaccctcccgcagcggactccacctcctcccaggcat
Xmv18       ttcccccccttctcccatggtgtctcgactgcggggaaggagagaccctcccgcagcggactccacctcctcccaggcat
Xmv19       ttcccccccttctcccatggtgtctcgactgcggggaaggagagaccctcccgcagcggactccacctcctcccaggcat
Xmv41       tCcTGccccCtctcccatggtgtctcgCctgcggggCaAAagagacccCcccgcGgcAgaTtccaccAcctcTcGggcTt
Xmv42       ttcccccccttctcccatggtgtctcgactgcggggaaggagagaccctcccgcagTggactccaccAcctcccaggcat
Xmv43       tCcTGccccCtctcccatggtgtctcgCTtgcggggCaAAagagacccCcccgcGgcAgTTtccaccAcctcTcGggcTt
Xmv8        ttcccccccttctcccatggtgtctcgactgcggggaaggagGgaccctcccgcagcggactccacctcctcccaggcat
Xmv9        ttccccccCtctcccatggtgtctcgactgcggggaaggagGgaccctcccgcagcggactccacctcctcccaggcat
```

```
XMV_CONS    TCCCACTCCGCATGGGGGGAGATGGCCAGCTTCAGTAYTGGCCGTTTTCCTCCTCGGACTTATATAATTGGAAAAATAAT
Xmv10       tcccactccgcatggggggagatggccagcttcagtaTtggccgttttcctcctcggacttataCaattggaaaaataat
Xmv12       tcccactccgcatggggggagatggccagcttcagtaCtggccgttttcctcctcAgacttatataattggaaaaataat
Xmv13       tcccactccgcatggggggagatggccagcttcagtaTtggccgttttcctcctcggacttataCaattggaaaaataat
Xmv15       tcccactccgcatggggggagatggccagcttcagtaCtggccgttttcctcctcTgacttatataattggaaaaataat
Xmv16       --------------------------------------------------------------------------------
Xmv17       tcccactccgcatggggggagatggccagcttcagtaCtggccgttttcctcctcggacttatataattggaaaaataat
Xmv18       tcccactccgcatggggggagatggccagcttcagtaCtggccgttttcctcctcTgacttatataattggaaaaataat
Xmv19       tcccactccgcatggggggagatggccagcttcagtaTtggccgttttcctcctcAgacttatataattggaaaaataat
Xmv41       tcccactccgTTtggggggTAatggTcagTtGcagtaCtggccgttttcctcctcTgaTCtatataaCtggaaaaataat
Xmv42       tTccactccgcatggggggagatggccagcttcagtaTtggccgttttcctcTtcggaTttataCaattggaaaaataat
Xmv43       tcccactccgTTtggggggTAatggTcagTtGcagtaCtggccgttttcctcctcggaTCtatataaCtggaaaaataat
Xmv8        tcccactccgcatggggggagatggccagcttcagtaTtggccgttttcctcctcggacttataCaattggaaaaataat
Xmv9        tcccactccgcatggggggagatggccagcttcagtaTtggccgttttcctcTtcggacttataCaattggaaaaataat

XMV_CONS    AACCCTTCCTTTTCTGAAGAYCCAGGTAAATTGACGGCCTTGATTGAGTCCGTCCTCATCACCCACCAGCCCACCTGGGA
Xmv10       aacccttccttttctgaagaCccaggtaaattgacggccttgattgagtccgtcctcatcacccaccagcccacctggga
Xmv12       aacccttccttttctgaagaCccaggtaaattgacggccttgattgagtccgtcctcatcacccaccagcccacctggga
Xmv13       aacccttccttttctgaagaCccaggtaaattgacggccttgattgagtccgtcctcatcacccaccagcccacctggga
Xmv15       aacccttccttttctgaagaTccaggtaaattgacggccttgattgagtccgtcctcatcacccaccagcccacctggga
Xmv16       --------------------------------------------------------------------------------
Xmv17       aacccttccttttctgaagaTccaggtaaattgacggccttgattgagtccgtcctcatcacccaccagcccacctggga
Xmv18       aacccttccttttctgaagaCccaggtaaattgacggccttgattgagtccgtcctcatcacccaccagcccacctggga
Xmv19       aacccttccttttctgaagaCccaggtaaattgacggccttgattgagtccgtcctcatcacccaccagcccacctggga
Xmv41       aaTccttccttttctgaagaTccaggtaaattgacTgccttAatCgagtcTgtTctcatcacccaTcagccTacctggga
Xmv42       aacccttccttttctgaagaTccaggtaaattgacggccttgattgagtccgtcctcatcacccaccagcccacctggga
Xmv43       aacccttccttCtctgaagaTccaggtaaattgacTgccttAatCgagtcTgtcctcaCcacccaccagccTacTtggga
Xmv8        aacccttccttttctgaagaTccaggtaaattgacggcTttgattgagtccgtcctcatcacccaccagcccacctggga
Xmv9        aacccttccttttctgaagaCccaggtaaattgacggcTttgattgagtccgtcctcatcacccaccagcccacctggga

XMV_CONS    CGACTGTCAGCAGTTGTTGGGGACCCTGCTGACCGGAGAAGAAAAGCAGCGGGTGCTCCTAGAGGCTAGAAAGGCAGTCC
Xmv10       cgactgtcagcagttgttggggaccctgctgaccggagaagaaaagcagcgggtgctcctagaggctagaaaggcagtcc
Xmv12       cgactgtcagcagttgttggggaccctgctgaccggagaagaaaagcagcgggtgctcctagaggctagaaaggcagtcc
Xmv13       cgactgtcagcagttgttAgggaccctgctgaccggagaagaaaagcagcgggtgctcctagaggctagaaaggcagtcc
Xmv15       cgactgtcagcagttgttggggaccctgctgaccggagaagaaaagcagcgggtgctcctagaggctagaaaggcagtcc
Xmv16       --------------------------------------------------------------------------------
Xmv17       cgactgtcagcagttgttggggaccctgctgaccggagaagaaaagcagcgggtgctcctagaggctagaaaggcagtcc
Xmv18       cgactgtcagcagttgttggggaccctgctgaccggagaagaaaagcagcgggtgctcctagaggctagaaaggcagtcc
Xmv19       cgactgtcagcagttgttggggaccctgctgaccggagaagaaaagcagcgggtgctcctagaggctagaaaggcagtcc
Xmv41       TgactgtcagcagttgCtggggacTctgctgacAggagaGgaGaagcagcgggtgctcctGgaggctagaaaggcagtcc
Xmv42       cgactgtcagcagttgCtggggaccctgctgaccggagaagaaaagcagcgggtgctcctagaggctagaaaggcagtcc
Xmv43       TgactgtcaAcagttgCtggggacTctgctgacAggagaagaaaagcagcgggtgctcctGgaAgcCagaaaggcagtcc
Xmv8        cgactgtcagcagttgttAgggaccctgctgaccggagaagaaaagcagcgggtgctcctagaggctagaaaggcagtcc
Xmv9        cgactgtcagcagttgttggggaccctgctgaccggagaagaaaagcagcgggtgctcctagaggctagaaaggcagtcc

XMV_CONS    GGGGCAATGATGGACGCCCCACTCAGTTGCCTAATGAAGTCAATGCTGCTTTTCCCCTTGAACGCCCCGATTGGGATTAC
Xmv10       ggggcaatgatggacgccccactcagttgcctaatgaagtcaatgctgcttttccccttgaacgccccgattgggattac
Xmv12       ggggcaatgatggacgccccactcagttgcctaatgaagtcaatgctgcttttccccttgaacgccccgattgggattac
Xmv13       ggggcaatgatggacgccccactcagttgcctaatgaagtcaatgctgcttttccccttgaacgccccgattgggattac
Xmv15       ggggcaatgatggacgccccactcagttgcctaatgaagtcaatgctgcttttccccttgaacgccccgattgggattac
Xmv16       --------------------------------------------------------------------------------
Xmv17       ggggcaatgatggacgccccactcagttgcctaatgaagtcaatgctgcttttccccttgaacgccccgattgggattac
Xmv18       ggggcaatgatggacgccccactcagttgcctaatgaagtcaatgctgcttttccccttgaacgccccgattgggattac
Xmv19       ggggcaatgatggacgccccactcagttgcctaatgaagtcaatgctgcCtttccccttgaacgccccgattgggattac
Xmv41       ggggcGaCgaCggGcgccccacCcagttgccCaaCgaGAtcGaGgcCgcCtttcccctCgaacgTcccgaCtgggaCtaT
Xmv42       ggggcaatgatggacgccccactcagttgcctaatgaagtcaatgctgcttttccccttgaacgccccgattgggattac
Xmv43       ggggcGaCgatggCcgccccacCcaAttgccCaatgaGAtcGaGgctgcCtttcccctCgaacgTcccgaCtgggaCtac
Xmv8        ggggcaatgatggacgccccactcagttgcctaatgaagtcaatgctgcttttccccttgaacgccccgattgggattac
Xmv9        ggggcaatgatggacgccccactcagttgcctaatgaagtcaatgctgcttttccccttgaacgccccgattgggatCac
```

```
XMV_CONS   ACCACTACAGAAGGTAGGAACCACCTAGTCCTCTATCGCCAGTTGCTCTTAGCGGGTCTCCAAAACGCGGGCAGGAGCCC
Xmv10      accactacagaaggtaggaaccacctagtcctctatcgccagttgctcttagcgggtctccaaaacgcgggcagAagccc
Xmv12      accactacagaaggtaggaaccacctagtcctctatcgccagttgctcttagcgggtctccaaaacgcgggcaggagccc
Xmv13      accactacagaaggtaggaaccacctagtcctctatcgccagttgctcttagcgggtctccaaaacgcgggcagAagccc
Xmv15      accactacagaaggtaggaaccacctagtcctctatcgccagttgctcttagcgggtctccaaaacgcgggcaggagccc
Xmv16      --------------------------------------------------------------------------------
Xmv17      accactacagaaggtaggaaccacctagtcctctatcgccagttgctcttagcgggtctccaaaacgcgggcaggagccc
Xmv18      accactacagaaggtaggaaccacctagtcctctatcgccagttgctcttagcgggtctccaaaacgcgggcaggagccc
Xmv19      accactacagaaggtaggaaccacctagtcctctatcgccagttgctcttagcgggtctccaaaacgcgggcaggagccc
Xmv41      accacCCcTgGaggtaggaaccacctagtTctctatcgccagCtgctcttagcgggtctccaGaaTgcgggcCggagccc
Xmv42      accactacagaaggtaggaaccacctagtcctctatcgccagttgctcttagcgggtctccaaaacgcgggcagAagccc
Xmv43      accacCCTTAGaggtaggaaccacctagtTctctatcgccagCtgctcttGgcgggtctccaaaaTgcgggcaggagccc
Xmv8       accactacagaaggtaggaaccacctagtcctctatcgccagttgctcttagcgggtctccaaaacgcgggcagAagccc
Xmv9       accactacagaaggtaggaaccacctagtcctctatcgccagttgctcttagcgggtctccaaaacgcgggcagAagccc

XMV_CONS   CACCAATTTGGCCAAGGTAAAAGGGATAACCCAGGGACCTAATGAGTCTCCCTCAGCCTTTTTAGAGAGACTCAAGGAGG
Xmv10      caccaatttggccaaggtaaaagggataacccagggacctaatgagtctccctcagcctttttagagagactcaaggagg
Xmv12      caccaatttggccaaggtaaaagggataacccagggacctaatgagtctccctcagcctttttagagagactcaaggagg
Xmv13      caccaatttggccaaggtaaaagggataacccagggacctaatgagtctccctcagcctttttagagagactcaaggagg
Xmv15      caccaatttggccaaggtaaaagggataacccagggacctaatgagtctccctcagcctttttagagagactcaaggagg
Xmv16      --------------------------------------------------------------------------------
Xmv17      caccaatttggccaaggtaaaagggataacccagggacctaatAagtctccctcagcctttttagagagactcaagAagg
Xmv18      caccaatttggccaaggtaaaagggataacccagggacctaatgagtctccctcagcctttttagagagactcaaggagg
Xmv19      caccaatttggccaaggtaaaagggataacccagggacctaatgagtctccctcagcctttttagagagactcaaggagg
Xmv41      caccaatttggccaaggtaaaagggataacTcagggaccCaatgagtctccctcGgcctttttagagagactcaaAgagg
Xmv42      caccaatttggccaaggtaaaaggAataacccagggacctaatgagtctccctcagcctttttagagagactcaaggagg
Xmv43      caccaatttggcTaaggtaaaaggAataacccagggGTcCaaCgagtcGccctcGgcctttCtagagagactcaaAgagg
Xmv8       caccaatttggccaaggtaaaagggataacccagggacctaatgagtctccctcagcctttttagagagactcaaggagg
Xmv9       caccaatttggccaaggtaaaagggataacccagggacctaatgagtctccctcagcctttttagagagactcaaggagg

XMV_CONS   CCTATCGCAGGTACACTCCTTATGACCCTGAGGACCCAGGGCAAGAAACCAATGTGTCTATGTCATTCATCTGGCAGTCT
Xmv10      cctatcgcaggtacactccttatgaccctgaggacccagggcaagaaaccaatgtgtctatgtcattcatctggcagtct
Xmv12      cctatcgcaggtacactccttatgaccctgaggacccagggcaagaaaccaatgtgtctatgtcattcatctggcagtct
Xmv13      cctatcgcaggtacactccttatgaccctgaggacccagggcaagaaaccaatgtgtctatgtcattcatctggcagtct
Xmv15      cctatcgcaggtacactccttatgaccctgaggacccagggcaagaaaccaatgtgtctatgtcattcatctggcagtct
Xmv16      --------------------------------------------------------------------------------
Xmv17      cctatcgcaggtacactccttatgaccctgaggacccagggcaagaaaccaatgtgtctatgtcattcatctggcagtct
Xmv18      cctatcgcaggtacactccttatgaccctgaggacccagggcaagaaaccaatgtgtctatgtcattcatctggcagtct
Xmv19      cctatcgcaggtacactccttatgaccctgaggacccagggcaagaaaccaatgtgtctatgtcattcatctggcagtct
Xmv41      cctatcgcaggtacactccttatgaccctgaggacccTgggcaagaaaccaatgtAtcCatgtcGttcatctggcagtcC
Xmv42      cctatcgcaggtacactccttatgaccctgaggacccagggcaagaaaccaatgtgtctatgtcattcatctggcagtct
Xmv43      cctatcgcagAtacactccttatgaccctgaggacccTgggcaagaaaccaatgtAtcCatgtcGttcatctggcagtct
Xmv8       cctatcgcaggtacactccttatgaccctgaggacccagggcaagaaaccaatgtgtctatgtcattcatctggcagtct
Xmv9       cAtatTgcaggtacactccttatgaccctgaggacccagggcaagaaaccaatgtgtctatgtcattcatctggcagtct

XMV_CONS   GCCCCGGATATCGGGCGAAAGTTAGAGCGGTTAGAAGATTTAAAGAGCAAGACCTTAGGAGACTTAGTGAGGGAAGCTGA
Xmv10      gccccggatatcgggcgaaagttagagcggttagaagatttaaagagcaagaccttaggagacttagtgagggaagctga
Xmv12      gccccggatatcgggcgaaagttagagcggttagaagaCttaaagagcaagaccttaggagacttagtgagggaagctga
Xmv13      gccccggatatcgggcgaaagttagagcggttagaagatttaaagagcaagaccttaggagaTttagtgagggaagctga
Xmv15      gccccggatatcgggcgaaagttagagcggttagaagatttaaagagcaagaccttaggagacttagtgagggaagctga
Xmv16      --------------------------------------------------------------------------------
Xmv17      gccccggatatcgggcgaaagttagagcggttaAaagatttaaagagcaagaccttaggagacttagtgagggaagctga
Xmv18      gccccggatatcgggcgaaagttagagcggttagaagaCttaaagagcaagaccttaggagacttagtgagggaagctga
Xmv19      gccccggatatcgggcgaaagttagagcggttagaagatttaaagagcaagaccttaggagacttagtgagggaagctga
Xmv41      gcTccAgaCatTggTcgGaagttagagcggttagaagaCttaaaAagcaaAaccttaggGgacttagtgagAgaagcCga
Xmv42      gccccggatatcgggcgaaagttagagcggttagaagatttaaagagcaagacTttaggagacttagtgagggaagctga
Xmv43      gcTccAgaCatTggTcgaaagttagagcggttagaagaCttaaaAaATaagaccttaggGgacttagtgagAgaagcAga
Xmv8       gccccggatatcgggcgaaagttagagcggttagaagatttaaagagcaagaccttaggagaTttagtgagggaagctga
Xmv9       gccccAgatatcgggcgaaagttagagcggttagaagatttaaagagcaagacAttaggagaTttagtgagggaagctga
```

```
XMV_CONS      AAGGATCTTTAATAAGCGAGAAACCCCGGAAGAAAGAGAGGAACGTATCAGGAGAGAAACAGAGGAAAAAGAAGAACGCC
Xmv10         aaggatctttaataagcgagaaaccccggaagaaagagaggaacgtatcaggagagaaacagaggaaaaagaagaacgcc
Xmv12         aaggatctttaataagcgagaaaccccggaagaaagagaggaacgtatcaggagagaaacagaggaaaaagaagaacgcc
Xmv13         aaAgatctttaataagcgagaaaccccggaagaaagagaggaacgtatcaggagagaaacagaggaaaaagaagaacgcc
Xmv15         aaggatctttaataagcgagaaaccccggaagaaagagaggaacgtatcaggagagaaacagaggaaaaagaagaacgcc
Xmv16         --------------------------------------------------------------------------------
Xmv17         aaggatctttaataagcgagaaaccccggaagaaagagaggaacgtatcaggagagaaacagaggaaaaagaagaacgcc
Xmv18         aaggatctttaataagcgagaaaccccggaagaaagagaggaacgtatcaggagagaaacagaggaaaaagaagaacgcc
Xmv19         aaggatctttaataagcgagaaaccccggaagaGagagaggaacgtatcaggagagaaacagaggaaaaagaagaacgcc
Xmv41         GaggatctttaataaAcgagaaaccccggaagaaagagaAgaacgtatcaggagagaaacagaggaaaaagaagaGcgcc
Xmv42         aaAgatctttaataagcgagaaaccccggaagaaagagaggaacgtatcaggagagaaaCgaggaaaGagaagaacgcc
Xmv43         aaggatctttaataagAgagaGaccccAgaagaGagagaAgaacgtatTaAgagagaaacagaggaaaaagaGgaGcgcc
Xmv8          aaAgatctttaataagcgagaaaccccggaagaaagagaggaacgtatcaggagagaaacagaggaaaaagaagaacgcc
Xmv9          aaAgatctttaataagcgagaaaccccggaagaaagagaggaacgtatcaggagagaaacagaggaaaaGgaagaacgcc

XMV_CONS      GTAGGGCAGAGGATGAGCAGAGAGAGAAAGAAAGGGACCGCAGAAGACATAGAGAGATGAGCAAGCTCTTGGCCACTGTA
Xmv10         gtagggcagaggatgagcagagagagaaagaaagggaccgcagaagacatagagagatgagcaagctcttggccactgta
Xmv12         gtagggcagaggatgagcagagagagaaagaaagggaccgcagaagacatagagagatgagcaagctcttggccactgta
Xmv13         gtagggcagaggatgagcagagagagaaagaaagggaccgcagGagacatagagagatgagcaagctcttggccactgta
Xmv15         gtagggcagaggatgagcagagagagaaagaaagggaccgcagaagacatagagagatgagcaagctcttggccactgta
Xmv16         --------------------------------------------------------------------------------
Xmv17         gtagggcagaggatgagcagagagagaaagaaagggaccgcagaagacatagagagatgagcaagctcttggccactgta
Xmv18         gtagggcagaggatgagcagagagagaaagaaagggaccgcagaagacatagagagatgagcaagctcttggccactgta
Xmv19         gtagggcagaggatgagcagagagagaaagaaagggaccgcagaagacatagagagatgagcaagctcttggccactgta
Xmv41         gtagggcagaggatgagcagagagaAaaagaaagggaccgcagaagacaAagaAaAatgagcaagctAttggccactgta
Xmv42         gtagggcagaAgatgagcagagagagaaagaaagggaccgcagaagacatagagagatgagcaagctcttggccactgta
Xmv43         gtagggcagaggatgagcagaAagagaaagaGagggaccgcagaagacaGagagaAatgagcaaActcttggccacCgta
Xmv8          gtagggcagaggatgagcagagagagaaagaaagggaccgcagGagacatagagaAatgagcaagctcttggccactgta
Xmv9          gtagggcagaggatgagcagagagagaaagaaagggaccgcagGagacatagagagatgagcaagctcttggccactgtG

XMV_CONS      GTTANTGGTCAGAGACAGGATAGACAGGGGGGAGAGCGRAGGAGGCCCCAACTTGATAAGGACCAATGCGCCTACTGCAA
Xmv10         gttaTtggtcagagacaggatagacaggggggagagcgGaggaggccccaacttgataaggaccaatgcgcctactgcaa
Xmv12         gttaTtggtcagagacaggatagacaggggggagagcgGaggaggccccaacttgataaggaccaatgcgcctactgcaa
Xmv13         gttaGtggAcagagacaggatagacaggggggagagcgAaggaggccccaacttgataaggaccaatgcgcctactgcaa
Xmv15         gttaTtggtcagagacaggatagacaggggggagagcgAaggaggccccaacttgataaggaccaatgcgcctactgcaa
Xmv16         ----------------------------------------ggaggccccaGcGAgataaggaccaatgcgcctactgcaa
Xmv17         gttaAtggtcagagacaggatagacaggggggagagcgGaggaggccccaacttgataaggaccaatgcgcctactgcaa
Xmv18         gttaTtggtcagagacaggatagacaggggggagagcgGaggaggccccaacttgataaggaccaatgcgcctactgcaa
Xmv19         gttaTtggtcagagGcaggatagacaggggggagagcgGaggaggccccaacttgataaggaccaatgcgcctactgcaa
Xmv41         gttaCtggtcagagacaggatagacaggggggagagcgAaggaggccccaactCgataaggaccaatgcgcctactgcaa
Xmv42         gttaTtggtcagagacaggatagacaggggggagagcgGaggaggccccaacttgataaggaccaatgcgcctactgcaa
Xmv43         gttaCAggtcagagacaggatagacaggggggagagcgAaggaggccccaactCgataaggaccaatgcgcctactgcaa
Xmv8          gttaGtggAcagagacaggatagacaggggggagagcgAaggaggccccaacttgataaggaccaatgcgcctactgcaa
Xmv9          gttaGtggAcagagacaggatagacaggggggagagcgAaggaggccccaacttgataaggaccaatgcgcctactgcaa

XMV_CONS      AGAAAAGGGACACTGGGCTAAGGACTGCCCAAAGAAGCCACGAGGGCCCCGAGGACCGAGGCCCCAGACCTCCCTCCTGA
Xmv10         agaaaagggacactgggctaaggactgcccaaagaagccacgagggccccgaggaccgaggccccagacctccctcctga
Xmv12         agaaaagggacactgggctaaggactgcccaaagaagccacgagggccccgaggaccgaggccccagacctccctcctga
Xmv13         agaaaagggacactgggctaaggactgcccaaagaagccacgagggccccgaggaccgaggccccagacctccctcctga
Xmv15         agaaaagggacactgggctaaggactgcccaaagaagccacgagggccccgaggaccgaggccccagacctccctcctga
Xmv16         agaaaagggacactgggctaaggactgcccaaagaagccacgagggccccgaggaccgaggccccagacctccctcctga
Xmv17         agaaaagggacactgggctaaggactgcccaaagaagccacgagggccccgaggaccgaggccccagacctccctcctga
Xmv18         agaaaagggacactgggctaaggactgcccaaagaagccacgagggccccgaggaccgaggccccagacctccctcctga
Xmv19         agaaaagggacactgggctaaggactgcccaaagaagccacgagggccccgaggaccgaggccccagacctccctcctga
Xmv41         agaaaagggacactgggctaaggaTtgcccaaagaagccacgGgggccccgaggaccgaggccccagacctccctcctga
Xmv42         agaaaagggacactgggctaaggactgcccaaagaagccacgagggccccgaggaccgaggccccagacctccctcctga
Xmv43         agaaaagggacactgggctaGggaTtgcccaaagaagccacgGgggccccgaggaccgaggccccagacctccctcctga
Xmv8          Ggaaaagggacactgggctaaggactgcccaaagaagccacgagggccccgaggaccgaggccccagacctccctcctga
Xmv9          agaaaagggacactgggctaaggactgcccaaagaagccacgagggccccgaggaccgaggccccagacctccctcctga
```

```
XMV_CONS      CCTTAGGTGACGCTCAGGTTGTGGGACCAATGGGACAGCCCCTGCAAGTGCTGACCCTAAACATAGARGATGAGTATCGG
Xmv10         ccttaggtgac---------------------------------------------------------------------
Xmv12         ccttaggtgacgctcaggttgtgggaccaatgggacagcccctgcaagtgctgaccctaaacatagaAgatgagtatcgg
Xmv13         ccttaggtgacgctcaggttgtgggaccaatgggacagcccctgcaagtgctgaccctaaacatagaGgatgagtatcgg
Xmv15         ccttaggtgacgctcaggttgtgggaccaatgggacagcccctgcaagtgctgaccctaaacatagaAgatgagtatcgg
Xmv16         ccttaggtgacgctcaggttgtgggaccaatgggacagcccctgcaagtgctgaccctaaacatagaGgatgagtatcgg
Xmv17         ccttaggtgac---------------------------------------------------------------------
Xmv18         ccttaggtgacgctcaggttgtgggaccaatgggacGgcccctgcaagtgctgaccctaaacatagaAgatgagtatcgg
Xmv19         ccttaggtgacgctcaggttgtgggaccaatgggGcagcccctgcaCgtgctgaccctaaacatagaAgatgagtatcgg
Xmv41         ccCtagAtgacgctcaggttgtgggaccaaAAggacagcccctAcaGgtActgaccctaaGcatagaGgatgagtatcgg
Xmv42         ccttaggtgacgctcaggttgtgggaccaatgggacagcccctgcaagtgctgaccctaaacatagaAgatgagtatcgg
Xmv43         ccCtagAtgacgctcaggttgtgggaccaaAAggacagcccctgcaGgtgTtgaccctTGGcatagaGgatgagtatcgg
Xmv8          ccttaggtgacgctcaggttgtgggaccGatgggacagcccctgcaagtgctgaccctaaacatagaGgatgagtatcgg
Xmv9          ccttaggtgacgctcaggttgtgggaccaatgggacagcccctgcaagtgctgaccctaaacatagaGgatgagtatcgg

XMV_CONS      CTACATGAGACCTCAAMAGAGCCGGATGTTTCTCTAGGGTCCACMTGGCTTTCTGATTTTCCCCAGGCCTGGGCAGAAAC
Xmv10         --------------------------------------------------------------------------------
Xmv12         ctacatgagacctcaaAagagccggatgtttctctagggtccacAtggctttctgattttccccaggcctgggcagaaac
Xmv13         ctacatgagacctcaaCagagccggatgtttctctagggtccacCtggctttctgattttccccaggcctgggcGgaaac
Xmv15         ctacatgagacctcaaAagagccggatgtttctctagggtccacAtggctttctgattttccccaggcctgggcagaaac
Xmv16         ctacatgagacctcaaCagagccggatgtttctctagggtccacCtggctttctgattttccccaggcctgggcGgaaac
Xmv17         --------------------------------------------------------------------------------
Xmv18         ctacatgagacctcaaAagagccggatgtttctctagggtccacAtggctttctgattttccccaggcctgggcagaaac
Xmv19         ctacatgagacctcaaAagagccggatgtttctctagggtcAacAtggctttctgattttccccaggcctgggcagaaac
Xmv41         ctacatgagacctcaaCagagccggatgtttctctagggtccacCtggctttctgaCtttccccaggcctgggcagaaac
Xmv42         ctacatgagacctcaaAagagccggatgtttctctagggtccacAtggctttctgattttccccaggcctgggcagaaac
Xmv43         ctacatgagacctcaaCagagccggatgtttctctagggtccacCtggctttctgaCtttccccaggcctgggcagaaac
Xmv8          ctacatgagacctcaaCagagccggatgtttctctagggtccacCtggctttctgattttccccaggcctgggcGgaaac
Xmv9          ctacatgagacctcaaCagagccggatgtttctctagggtccacCtggctttctgattttccccaggcctgggcGgaaac

XMV_CONS      CGGGGGCATGGGACTGGCAGTTCGCCAAGCGCCTCTGATTATACCTCTAAAGGCAACCTCYACCCCBGTGTCCATAAAAC
Xmv10         --------------------------------------------------------------------------------
Xmv12         cgggggcatgggactggcagttcgccaagcgcctctgattatacctctaaaggcaacctcTaccccCgtgtccataaaac
Xmv13         cgggggcatgggactggcagttcgccaagcgcctctgattatacctctaaaggcaacctcCaccccTgtgtccataaaac
Xmv15         cgggggcatgggactggcagttcgccaagcgcctctgattatacctctaaaggcaacctcTaccccCgtgtccataaaac
Xmv16         cgggggcatgggactggcagttcgccaagcgcctctgattatacctctaaaggcaacctcCaccccTgtgtccataaaac
Xmv17         --------------------------------------------------------------------------------
Xmv18         cgggggcatgggactggcagttcgccaagcgcctctgattatacctctaaaggcaacctcTaccccCgtgtccataaaac
Xmv19         cgggggcatgggactggcagttcgccaagcgcctctgattatacctctaaaggcaacctcTaccccCgtgtccataaaac
Xmv41         cgggggcatgAgactggcagttcgccaagcgccCctgattatacctctaaaggcaacctcCaccccTgtgtccatCaaac
Xmv42         cgggggcatgggactggcagttcgccaagcTcctctgatCatacctctGaaggcaacctcTaccccCgtgtccataaaac
Xmv43         cgggggcatgggactggcagttcgccaagcgcctctgattatacctctaaaggcaacctcCaccccTgtgtccatCaaac
Xmv8          TgggggcatgggactggcagttcgccaagcgcctctgattatacctctGaaggcaacctcCaccccTgtgtccataaaac
Xmv9          TgggggcatgggactggcagttcgccaagcgcctctgattatacctctGaaggcaacctcCaccccGgtgtccataaaac

XMV_CONS      AATACCCCATGTCACAGGAAGCCAGACTGGGGATCAAGCCCCACATACAGAGGCTGTTGGACCAGGGAATACTGGTACCC
Xmv10         --------------------------------------------------------------------------------
Xmv12         aataccccatgtcacaggaagccagactggggatcaagccccacatacagaggctgttggaccagggaatactggtaccc
Xmv13         aataccccatgtcGcaAgaagccagactggggatcaagccccacatacagaggctgttggaccagggaataTtggtaccc
Xmv15         aataccccatgtcacaggaagccagactggggatcaagccccacatacagaggctgttggaccagggaatactggtaccc
Xmv16         aataccccatgtcGcaAgaagccagactggggatcaagccccacatacagaggctgttggaccagggaataTtggtaccc
Xmv17         --------------------------------------------------------------------------------
Xmv18         aataccccatgtcacaggaagccagactggggatcaagccccacatacagaggctgttggaccagggaatactggtaccc
Xmv19         aataccccatgtcacaggaagccagactggggatcaagccccacatacagaggctgttggaccagggaatactgAtaccc
Xmv41         aataccccatgtcacaCgaagccagactggggatcaagccccacatacagagActgttggaccagggaataTtggtaccT
Xmv42         aataccccatgtcacaggaagccagactggggatcaagccccacatacagaggctgttggaccagggaatactggtaccc
Xmv43         aGtaccccatgtcacaCgaagccagactggggatcaagccccacatacagagActgttggaccagggaataTtggtaccT
Xmv8          aataccccatgtcacaggaagccagactggggatcaagccccacatacagaggctgttggaccagggaatactggtaccc
Xmv9          aataccccatgtcacaggaagccagactggggatcaagccccacatacagaggctgttggaccagggaatactggtaccc
```

```
XMV_CONS      TGCCAGTCCCCCTGGAACACRCCCCTGCTACCCGTTAAGAAACCAGGGACTAATGATTACAGGCCTGTCCAGGATCTGAG
Xmv10         --------------------------------------------------------------------------------
Xmv12         tgccagtccccctggaacacGcccctgctacccgttaagaaaccagggactaatgattacaggcctgtccaggatctgag
Xmv13         tgccagtccccctggaaTacAcccctgctacccgttaagaaaccagggactaatgattacaggcctgtccaggatctgag
Xmv15         tgccagtccccctggaacacGcccctgctacccgttaagaaaccagggactaatgattacaggcctgtccaggatctgag
Xmv16         tgccagtccccctggaaTacAcccctgctacccgttaagaaaccagggactaatgattacaggcctgtccaggatctgag
Xmv17         --------------------------------------------------------------------------------
Xmv18         tgccagtccccctggaacacGcccctgctacccgttaagaaaccagggactaatgattacaggcctgtccaggatctgag
Xmv19         tgccagtccccctggaacacGcccctgctacccgttaagaaaccagggactaatgattacaggcctgtccaggatctgag
Xmv41         tgccagtccccctggaacacAcccctgctGcccgttaagaaaccagggactaatgattacaggcctgtccaggatctgag
Xmv42         tgccagtccccctggaacacGcccctgctacccgttaagaaaccagggactaatgattacaggcctgtccaggatctgag
Xmv43         tgccagtccccctggaacacAcccctgctGcccgttaagaaaccagggactaatgattacaggcctgtccaggatctgag
Xmv8          tgccagtccccctggaacacGcccctgctacccgttaagaaaccagggactaatgattacaggcctgtccaggatctgag
Xmv9          tgccagtccccctggaaTacAcccctgctacccgttaagaaaccagggactaaCgattaTaggcctgtccaggatctgag

XMV_CONS      AGAAGTCAACAAGCGGGTGGAAGACATCCACCCCACCGTGCCCAACCCTTACAACCTCTTRAGYGGGCTCCCACCGTCCC
Xmv10         --------------------------------------------------------------------------------
Xmv12         agaagtcaacaagcgggtggaagacatccaccccaccgtgcccaacccttacaacctcttGagCgggctcccaccgtccc
Xmv13         agaagtTaacaagcgggtAgaagacatccaccccaccgtgcccaacccttacaacctcttAagTgggctcccaccgtccc
Xmv15         agaagtcaacaagcgggtggaagacatccaccccaccgtgcccaacccttacaacctcttGagCgggctcccaccgtccc
Xmv16         agaagtTaacaagcgggtAgaagacatccaccccaccgtgcccaacccttacaacctcttAagTgggctcccaccgtccc
Xmv17         --------------------------------------------------------------------------------
Xmv18         agaagtcaacaagcgggtggaagacatccaccccaccgtgcccaacccttacaacctcttGagCgggctcccaccgtccc
Xmv19         agaagtcaacaagcgggtggaagacatccaccccaccgtgcccaacccttacaacctcttGagCgggctcccaccgtccc
Xmv41         agaagtcaacaagcgggtggaagaTatccaccccaccgtgcccaaTccttacaacctcttAagTggActcccTccgtccc
Xmv42         agaagtcaacaagcgggtggaagacatccaccccaccgtgcccaacccttacaacctcttGagCgggctcccaccgtccc
Xmv43         agaagtcaacaagcgggtggaagaTatccaccccaccgtgcccaaTccttacaacctcttAagTggActcccTccgtccc
Xmv8          agaagtcaacaagcAggtggaagacatccaccccaccgtgcccaacccttacaacctcttGagCgggctcccaccgtccc
Xmv9          agaagtcaacaagcgggtggaagacatccaccccaccgtgcccaaTccttacaacctcttAagTgggctcccaccAtccc

XMV_CONS      ACCAGTGGTACACTGTGCTTGATTTAAAGGATGCCTTTTTCTGCCTGAGACTCCACCCCACCAGTCAGCCTCTCTTCGCC
Xmv10         --------------------------------------------------------------------------------
Xmv12         accagtggtacactgtgcttgatttaaaggatgcctttttctgcctgagactccaccccaccagtcagcctctcttcgcc
Xmv13         accagtggtacactgtgcttgatttaaaggatgcctttttctgcctgagactccaccccaccagtcagcctctcttcgcc
Xmv15         accagtggtacactgtgcttgatttaaaggatgcctttttctgcctgagactccaccccaccagtcagcctctcttcgcc
Xmv16         accagtggtacactgtgcttgatttaaaggatgcctttttctgcctgagactccaccccaccagtcagcctctcttcgcc
Xmv17         --------------------------------------------------------------------------------
Xmv18         accagtggtacactgtgcttgatttaaaggatgcctttttctgcctgagactccaccccaccagtcagcctctcttcgcc
Xmv19         accagtggtacactgtgcttgatttaaaggatgcctttttctgcctgagactccaccccaccagtcagcctctcttcgcc
Xmv41         accagtggtacactgtgctCgatttaaaAgatgcctttttctgcctgagactccaccccaccagtcagcctctcttcgcc
Xmv42         accagtggtacactgtgcttgatttaaaggatgcctttttctgcctgagactccaccccaccagtcagcctctcttcgcc
Xmv43         accagtggtacactgtgcttgatttaaaAgatgcctttttctgcctgagactccaccccaccagtcagcctctcttTgcc
Xmv8          accagtggtacactgtgcttgatttaaaggatgcctttttctgcctgagactccaccccaccagtcagcctctcttcgcc
Xmv9          accagtggtaTactgtgcttgatttaaaggatgcctttttctgcctgagactccaccccaccagtcagcctctcttcgcc

XMV_CONS      TTTGAGTGGAGAGATCCAGGGATGGGAATCTCAGGACAATTGACCTGGACCAGACTCCCACAGGGTTTCAAAAACAGTCC
Xmv10         --------------------------------------------------------------------------------
Xmv12         tttgagtggagagatccagggatgggaatctcaggacaattgacctggaccagactcccacagggtttcaaaaacagtcc
Xmv13         tttgagtggagagatccagggatgggaatctcaggacaattgacctggaccagactcccacagggtttcaaaaacagtcc
Xmv15         tttgagtggagagatccagggatgggaatctcaggacaattgacctggaccagactcccacagggtttcaaaaacagtcc
Xmv16         tttgagtggagagatccagggatgggaatctcaggacaattgacctggaccagactcccacagggtttcaaaaacagtcc
Xmv17         --------------------------------------------------------------------------------
Xmv18         tttgagtggagagatccagggatgggaatctcaggacaattgacctggaccagactcccacagggtttcaaaaacagtcc
Xmv19         tttgagtggagagatccagAgatgggaatctcaggacaattgacctggaccagactcccacagggtttcaaaaacagtcc
Xmv41         tttgagtggagagatccagAAatgggaatctcTggGcaattgacctAgaccagactcccacagggtttcaaaaacagtcc
Xmv42         tttgagtggagagatccagggatgggaatctcaggacaattgacctggaccagactcccacagggtttcaaaaacagtcc
Xmv43         tttgagtggagagatccagAAatgggaatctcTggacaattgacctggaccagactcccacagggtttcaaaaacagtcc
Xmv8          tttgagtggagagatccagggatgggaatctcaggacaattgacctggaccagactcccacagggtttcaaaaacagtcc
Xmv9          tttgagtggagagatccagAAatgggaatctcaggacaattgacctggaccagactcccacagggtttcaaaaacagtcc
```

```
XMV_CONS    CACCCTGTTTGATGAGGCACTGCACAGAGACCTAGCAGACTTCCGGATCCAGCACCCAGACTTGATCCTGCTACAGTACG
Xmv10       --------------------------------------------------------------------------------
Xmv12       caccctgtttgatgaggcactgcacagagacctagcagacttccggatccagcacccagacttgatcctgctacagtacg
Xmv13       caccctgtttgatgaggcactgcacagagacctagcagacttccggatccagcacccagacttgatcctgctacagtacg
Xmv15       caccctgtttgatgaggcactAcacagagacctagcagacttccggatccagcacccagacttgatcctgctacagtacg
Xmv16       caccctgtttgatgaggcactgcacagagacctagcagacttccggatccagcacccagacttgatcctgctacagtacg
Xmv17       --------------------------------------------------------------------------------
Xmv18       caccctgtttgatgaggcactgcacagagacctagcagacttccggatccagcacccagacttgatcctgctacagtacg
Xmv19       caccctgtttgatgaggcactAcacagagacctagcagacttccggatccagcacccagacttgatcctgctacagtacg
Xmv41       caccctgtttAatgaggcactgcacagagacctagcagacttccggatccagcacccagacttgatcctgctacagtacg
Xmv42       caccctgtttgatgaggcactgcacagagacctagcagacttccggatccagcacccagacttgatcctgctacagtacg
Xmv43       caccctgtttgatgaggcaTtgcacagagacctagcagacttccggatccagcacccagacttgatcctgctacagtacg
Xmv8        caccctgtttgaCgaggcTctgcacagagacctagcCgacttccggatccagcacccagacttgatcctgTtGcagtacg
Xmv9        caccctgtttgaCgaggcTctgcacagagacctagcCgacttccggatccagcacccagacttgatcctgTtGcagtacg

XMV_CONS    TGGATGACATACTACTGGCCGCCACTTCTGAGCTCGACTGCCAACAAGGTACTCGGGCCCTDTTACAAACCCTAGGRRAC
Xmv10       --------------------------------------------------------------------------------
Xmv12       tggatgacatactactggccgccacttctgagctcgactgccaacaaggtactcgggccctGttacaaaccctaggAAac
Xmv13       tggatgacatactactggccgccacttctgagctcgactgccaacaaggtactcgggccctGttacTaaccctaggAAac
Xmv15       tggatgacatactactggccgccacttctgagctcgactgccaacaaggtactcgggccctGttacaaaccctaggAAac
Xmv16       tggatgacatactactggccgccacttctgagctcgactgccaacaaggtactcgggccctGttacTaaccctaggAAac
Xmv17       --------------------------------------------------------------------------------
Xmv18       tggatgacatactactggccgccacttctgagctcgactgccaacaaggtactcgggccctGttacaaaccctaggAAac
Xmv19       tggatgacatactactggccgccacttctgagctcAactgccaacaaggtactcgggccctGttacaaaccctaggAAac
Xmv41       tggatgacTtactGctggccgcTacttcCgaActAgactgccaacaaggtactcgggccctACtacaaaccctaggGGac
Xmv42       tggatgacatactGctggccgccacttctgagctcgactgccaacaag--------------------------------
Xmv43       tggatgacTtactGctggccgcTacttcCgaActAgactgccaacaaggtactcgggccctTCtacaaaccctaggGGac
Xmv8        tAgatgacTtactGctggccgcGacttcCgagcCTgactgccaacaaggtactcgAgccctAttacaaaccTtaggGGac
Xmv9        tAgatgacTtactactggccgcGacttcCgagcCTgactgccaacaaggtactcgAgccctAttacaaaccTtaggGGac

XMV_CONS    CTCGGRTAYCGGGCCTCGGCCAAGAAAGCCCAACTTTGCCAGAAACAGGTCAAGTATCTGGGGTATCTTCTAAAAGAGGG
Xmv10       --------------------------------------------------------------------------------
Xmv12       ctcggGtaTcgggcctcggccaagaaagcccaactttgccagaaacaggtcaagtatctggggtatcttctaaaagaggg
Xmv13       ctcggGtaTcgggcctcggccaagaaagcccaactttgccagaaGcaggtcaagtatctggggtatcttctaaaagaggg
Xmv15       ctcggGtaTcgggcctcggccaagaaagcccaactttgccagaaacaggtcaagtatctggggtatcttctaaaagaggg
Xmv16       ctcggGtaTcgggcctcggccaagaaagcccaactttgccagaaGcaggtcaagtatctggggtatcttctaaaagaggg
Xmv17       --------------------------------------------------------------------------------
Xmv18       ctcggGtaTcgggcctcggccaagaaagcccaactttgccagaaacaggtcaagtatctggggtatcttctaaaagaggg
Xmv19       ctcggGtaTcgggcctcggccaagaaagcccaactttgccagaaacaggtcaagtatctggggtatcttctGaaagaggg
Xmv41       ctcggAtaCcgggcctcggccaagaaagcccaaAtCtgccagaaacaggtTaaAtaCctggggtaCcttctGaGGgaggg
Xmv42       --------------------------------------------------------------------------------
Xmv43       ctcggAtaCcgggcctcggccaagaaagcccaaAtCtgccagaaacaggtTaaAtaCctggggtaCcttctGaGGgaggg
Xmv8        ctcggAtaCcgggcctcggccaagaaagcccaactttgccagaaacaggtcaagtatctggggtatcttctaaaagaggg
Xmv9        ctcggAtaCcgggcctcggccaagaaagcccaactttgccagaaacaggtcaagtatctggggtatcttctaaaagaggg

XMV_CONS    TCAGAGATGGCTGACTGAGGCCAGAAAAGAGACTGTGATGGGGCAGCCTACTCCGAAGACCCCTCGACAACTAAGGGAGT
Xmv10       --------------------------------------------------------------------------------
Xmv12       tcagagatggctgactgaggccagaaaagagactgtgatggggcagcctactccgaagacccctcgacaactaagggagt
Xmv13       tcagagatggctgactgaggccagaaaagagactgtgatggggcagcctactccgaagacccctcgacaactaagggagt
Xmv15       tcagagatggctgactgaggccagaaaagagactgtgatggggcagcctactccgaagacccctcgacaactaagggagt
Xmv16       tcagagatggctgactgaggccagaaaagagactgtgatggggcagcctactccgaagacccctcgacaactaagggagt
Xmv17       --------------------------------------------------------------------------------
Xmv18       tcagagatggctgactgaggccagaaaagagactgtgatggggcagcctactccgaagacccctcgacaactaagggagt
Xmv19       tcagagatggctgactgaggccagaaaagagactgtgatggggcagcctactccgaagacccctcgacaactaagggagt
Xmv41       tcagagatggctgactgaggcTagaaaagagactgtgatggggcaAccCGTtccAaagacTcctcgacaactTagggagt
Xmv42       --------------------------------------------------------------------------------
Xmv43       tcagagatggctgactgaggcTagaaaagagactgtgatggggcaAccCGTtccAaagacTcctcgacaactaagggagt
Xmv8        tcagagatggctgactgaggccagaaaagagactgtgatggggcagcctactccgaagacccctcgacaactaagggagt
Xmv9        tcagagatggctgactgaggccagaaaagagactgtgatggggcagcctactccgaagacccctcgacaactaagggagt
```

```
XMV_CONS      TCCTAGGGACGGCAGGCTTCTGTCGCCTCTGGATCCCTGGGTTTGCGGAAATGGCAGCCCCCTTGTATCCTCTTACCAAA
Xmv10         --------------------------------------------------------------------------------
Xmv12         tcctagggacggcaggcttctgtcgcctctggatccctgggtttgcggaaatggcagccccccttgtatcctcttaccaaa
Xmv13         tcctagggacggcaggcttctgtcgcctctggatccctgggtttgcggaaatggcagccccccttgtatcctcttaccaaa
Xmv15         tcctagggacggcaggcttctgtcgcctctggatccctgggtttgcggaaatggcagccccccttgtatcctcttaccaaa
Xmv16         tcctagggacggcaggcttctgtcgcctctggatccctgggtttgcggaaatggcagccccccttgtatcctcttaccaaa
Xmv17         --------------------------------------------------------------------------------
Xmv18         tcctagggacggcaggcttctgtcgcctctggatccctgggtttgcggaaatggcagccccccttgtatcctcttaccaaa
Xmv19         tcctagggacggcaggcttctgtcgcctctggatccctgggtttgcggaaatggcagccccccttgtatcctcttaccaaa
Xmv41         tcctagggacggcaggcttctgCcgcctctggatccctgggtttgcggaaatggcGgccccccttAtaCcctctCaccaaa
Xmv42         --------------------------------------------------------------------------------
Xmv43         tcctagggacggcaggcttctgCcgcctctggatccctgggtttgcggaaatggcGgccccccttgtatcctcttaccaaa
Xmv8          tcctagggacggcaggcttctgtcgcctctggatccctgggtttgcggaaatggcagccccccttgtatcctcttaccaaa
Xmv9          tcctagggacggcaggcttctgtcgcctctggatccctgggtttgcggaaatggcagccccccttgtatcctcttaccaaa

XMV_CONS      ACGGGGACTCTGTTTAATTGGGGCCCAGACCAGCAAAAGGCCTATCAAGAAATCAAACAGGCCCTTCTAACTGCCCCCGC
Xmv10         --------------------------------------------------------------------------------
Xmv12         acggggactctgtttaattggggcccagaccagcaaaaggcctatcaagaaatcaaacaggcccttctaactgcccccgc
Xmv13         acggggactctgtttaattggggcccagaccagcaaaaggcctatcaagaaatcaaacaggcccttctaactgcccccgc
Xmv15         acggggactctgtttaattggggcccagaccagcaaaaggcctatcaagaaatcaaacaggcccttctaactgcccccgc
Xmv16         acggggactctgtttaattggggcccagaccagcaaaaggcctatcaagaaatcaaacaggcccttctaactgcccccgc
Xmv17         --------------------------------------------------------------------------------
Xmv18         acggggactctgtttaattggggcccagaccagcaaaaggcctatcaagaaatcaaacaggcccttctaactgcccccgc
Xmv19         acggggactctgtttaattggggcccagaccagcaaaaggcctatcaagaaatcaaacaggcccttctaactgcccccgc
Xmv41         acTgggactctgtttaattggggcccagaTcagcaGaGggcctatcaagaaatcaaacaggccctCctGacGccccTgc
Xmv42         --------------------------------------------------------------------------------
Xmv43         acggggactctgtttaattggggcccagaccagcaaaaggcctatcaagaaatcaaacaggcccttctaactgcccccgc
Xmv8          acggggactctgtttaattggggcccagaccagcaaaaggcctatcaagaaatcaaacaggcccttctaactgcccccgc
Xmv9          acggggactctgtttaattggggcccagaccagcaaaaggcctatcaagaaatcaaacaggcccttctaactgcccccgc

XMV_CONS      CCTGGGATTGCCAGATTTGACTAAGCCCTTTGAACTCTTTGTCGACGAGAAGCAGGGCTACGCCAAAGGCGTCCTAACGC
Xmv10         --------------------------------------------------------------------------------
Xmv12         cctgggattgccagatttgactaagccctttgaactctttgtcgacgagaagcagggctacgccaaaggcgtcctaacgc
Xmv13         cctgggattgccagatttgactaagccctttgaactctttgtcgacgagaagcagggctacgccaaaggcgtcctaacgc
Xmv15         cctgggattgccagatttgactaagccctttgaactctttgtcgacgagaagcagggctacgccaaaggcgtcctaacgc
Xmv16         cctgggattgccagatttgactaagccctttgaactctttgtcgacgagaagcagggctacgccaaaggcgtcctaacgc
Xmv17         --------------------------------------------------------------------------------
Xmv18         cctgggattgccagatttgactaagccctttgaactctttgtcgacgagaagcagggctacgccaaaggcgtcctaacgc
Xmv19         cctgggattgccagatttgactaagccctttgaactctttgtcgacgagaagcagggctacgccaaaggcgtcctaacgc
Xmv41         cctgggattgccagaCttgacCaagccctttgaactctttgtcgaTgaAaagcagggctacgccaaaggGgtcctaacgc
Xmv42         --------------------------------------------------------------------------------
Xmv43         cctgggattgccagatttgactaagccctttgaactctttgtcgacgagaagcagggctacgccaaaggcgtcctaacgc
Xmv8          cctgggattgccagatttgactaaAccctttgaactctttgtcgacgagaagcagggctacgccaaaggcgtcctaacgc
Xmv9          cctgggattgccagatttgactaaAccctttgaactctttgtcgacgagaagcagggctacgccaaaggcgtcctaacgc

XMV_CONS      AAAAACTGGGACCTTGGCGTCGGCCTGTGGCCTACCTGTCCAAAAAGCTAGACCCAGTGGCAGCTGGGTGGCCCCCTTGC
Xmv10         --------------------------------------------------------------------------------
Xmv12         aaaaactgggaccttggcgtcggcctgtggcctacctgtccaaaaagctagacccagtggcagctgggtggccccccttgc
Xmv13         aaaaactgggaccttggcgtcggcctgtggcctacctgtccaaaaagctagacccagtggcagctgggtggccccccttgc
Xmv15         aaaaactgggaccttggcgtcggcctgtggcctacctgtccaaaaagctagacccagtggcagctgggtggccccccttgc
Xmv16         aaaaactgggaccttggcgtcggcctgtggcctacctgtccaaaaagctagacccagtggcagctgggtggccccccttgc
Xmv17         --------------------------------------------------------------------------------
Xmv18         aaaaactgggaccttggcgtcggcctgtggcctacctgtccaaaaagctagacccagtggcagctgggtggccccccttgc
Xmv19         aaaaactgggaccttggcgtcggcctgtggcctacctgtccaaaaagctagacccagtggcagctgggtggccccccttgc
Xmv41         aGaaactgggaccttggcgCcggcctgtggcctacctgtccaaaaagctGgacccagtggcagcCgggtggcccccCtgc
Xmv42         --------------------------------------------------------------------------------
Xmv43         aaaaactgggaccttggcgtcggcctgtggcctacctgtccaaaaagctagacccagtggcagcCgggtggccccccttgc
Xmv8          aaaaactgggaccttggcgtcggcctgtggcctacctgtccaaaaagctagacccagtggcagctgggtggccccccttgc
Xmv9          aaaaactgggaccttggcgtcggcctgtggcctacctgtccaaaaagctagacccagtggcagctgggtggccccccttgc
```

```
XMV_CONS    CTACGGATGGTAGCAGCCATTGCCGTTCTGACAAAAGATGCAGGCAAGCTAACTATGGGACAGCCGCTAGTCATCCTGGC
Xmv10       -----------------------------------------------------------------------catcctggc
Xmv12       ctacggatggtagcagccattgccgttctgacaaaagatgcaggcaagctaactatgggacagccgctagtcatcctggc
Xmv13       ctacggatggtagcagccattgccAttctgacaaaagatgcaggcaagctaactatgggacagccgctagtcatcctggc
Xmv15       ctacggatggtagcagccattgccgttctgacaaaagatgcaggcaagctaactatgggacagccgctagtcatcctggc
Xmv16       ctacggatggtagcagccattgccAttctgacaaaagatgcaggcaagctaactatgggacagccgctagtcatcctggc
Xmv17       --------------------------------------------------------------------------------
Xmv18       ctacggatggtagcagccattgccgttctgacaaaagatgcaggcaagctaactatgggacagccgctagtcatcctggc
Xmv19       ctacggatggtagcagccattgccgttctgacaaaagatgcaggcaagctaactatgggacagccgctagtcatcctggc
Xmv41       TtacggatggtagcagccattgccAttctgacaaaGgatgcaggcaaActaacGatgggacagccgctagtcatTctggc
Xmv42       --------------------------------------------------------------------------------
Xmv43       ctacggatggtagcagccattgccgttctgacaaaagatgcaggcaagctaactatgggacagccgctagtcatcctggc
Xmv8        ctacggatggtagcagccattgccgttTtgaTaaaagatgcaggcaaActaactatgggacagccgctagtcatcctggc
Xmv9        ctacggatggtagcagccattgccgttctgaTaaaagatgcaggcaagctaactatgggacagccgctagtcatcctggc

XMV_CONS    CCCCCATGCAGTAGAGGCACTGGTCAAGCAACCCCCTGACCGCTGGCTATCCAAYGCCCGCATGACCCACTACCAGGCAA
Xmv10       cccccatgcagtagaggcactggtcaagcaaccccctgaccgctggctatccaaTgcccgcatgacccactaccaggcaa
Xmv12       cccccatgcagtagaggcactggtcaagcaaccccctgaccgctggctatccaaCgcccgcatgacccactaccaggcaa
Xmv13       cccccatgcagtagaggcactggtcaagcaaccccctgaccgctggctatccaaTgcccgcatgacccactaccaggcaa
Xmv15       cccccatgcagtagaggcactggtcaagcaaccccctgaccgctggctatccaaCgcccgcatgacccactaccaggcaa
Xmv16       cccccatgcagtagaggcactggtcaagcaaccccctgaccgctggctatccaaTgcccgcatgacccactaccaggcaa
Xmv17       --------------------------------------------------------------------------------
Xmv18       cccccatgcagtagaggcactggtcaagcaaccccctgaccgctggctatccaaCgcccgcatgacccactaccaggcaa
Xmv19       cccccatgcagtagaggcactggtcaagcaaccccctgaccgctggctatccaaCgcccgcatgacccactaccaggcaa
Xmv41       cccccatgcGgtagaAgcactggtcaaAcaaccccctgaccgTtggctatccaaTgcccgcatgacccactaTcaggcCa
Xmv42       --------------------------------------------------------------------------------
Xmv43       cccccatgcagtagaggcactggtcaagcaaccccctgaccgctggctatccaaCgcccgcatgacccactaccaggcaa
Xmv8        cccccatgcagtagaggcactggtcaagcaaccccctgaccgctggctatccaaTgcccgcatgacccactaccagAcaa
Xmv9        cccccatgcagtagaggcactggtcaagcaaccccctgaccgctggctatccaaTgcccgcatgacccactaccaggcaa

XMV_CONS    TGCTCCTAGACACTGACCGAGTTCAGTTCGGACCAGTGGTGGCCCTCAATCCTGCCACCTTGCTCCCTCTACCGGARAAA
Xmv10       tgctcctagacactgaccgagttcagttcggaccagtggtggccctcaatcctgccaccttgctccctctaccggaGaaa
Xmv12       tgctcctagacactgaccgagttcagttcggaccagtggtggccctcaatcctgccaccttgctccctctaccggaAaaa
Xmv13       tgctcctagacactgaccgagttcagttcggaccagtggtggccctcaatcctgccaccttgctccctctaccggaGaaa
Xmv15       tgctcctagacactgaccgagttcagttcggaccagtggtggccctcaatcctgccaccttgctccctctaccggaAaaa
Xmv16       tgctcctagacactgaccgagttcagttcggaccagtggtggccctcaatcctgccaccttgctccctctaccggaGaaa
Xmv17       --------------------------------------------------------------------------------
Xmv18       tgctcctagacactgaccgagttcagttcggaccagtggtggccctcaatcctgccaccttgctccctctaccggaAaaa
Xmv19       tgctcctagacactgaccgagttcagttcggaccagtggtggccctcaatcctgccaccttgctccctctaccggaAaaa
Xmv41       tgctcctGgaTacGgaAcgGgttcagttcggaccGgtggtgAccctcaaCccGgccaccttgctcccCctaccggaAaaa
Xmv42       --------------------------------------------------------------------------------
Xmv43       tgctcctagacactgaccgagttcagttcggaccagtggtggccctcaatcctgccaccttgctccctctaccggaAaaa
Xmv8        tgctcctagacactgaccgagttcagttcggaccagtggtggccctcaatcctgccaccttgctccctctaccggaGaaa
Xmv9        tgctcctagacactgaccgagttcagttcggaccagtggtggccctcaatcctgccaccttgctccctctaccggaGaaa

XMV_CONS    GGAGCCCCCCATGATTGCCTCGAGATCTTGGCTGAAACGCATGGAACCAGACCGGAYCTCACCGACCAGCCCATCCCAGA
Xmv10       ggagccccccatgattgcctcgagatcttggctgaaacgcatggaaccagaccggaCctcaccgaccagcccatcccaga
Xmv12       ggagccccccatgattgcctcgagatcttggctgaaacgcatggaaccagaccggaTctcaccgaccagcccatcccaga
Xmv13       ggagccccccatgattgcctcgagatcttggctgaaacgcatggaaccagaccggaCctcaccgaccagcccatcccaga
Xmv15       ggagccccccatgattgcctcgagatcttggctgaaacgcatggaaccagaccggaTctcaccgaccagcccatcccaga
Xmv16       ggagccccccatgattgcctcgagatcttggctgaaacgcatggaaccagaccggaCctcaccgaccagcccatcccaga
Xmv17       ----ccccccatgattgcctcgagatcttggctgaaacgcatggaaccagaccggaTctcaccgaccagcccatcccaga
Xmv18       ggagccccccatgattgcctcgagatcttggctgaaacgcatggaaccagaccggaTctcaccgaccagcccatcccaga
Xmv19       ggagccccccatgattgcctcgagatcttggctgaaacgcatggaaccagaccggaTctcaccgaccagcccatcccaga
Xmv41       ggagccccccatgaCtgcctcgagatcttggctgaGacAcatggaaccagaccggaCctcacGgaccagcccatcccaga
Xmv42       --------------------------------------------------------------------------------
Xmv43       ggagccccccatgattgcctcgagatcttggctgaaacgcatggaaccagaccggaTctcaccgaccagcccatcccaga
Xmv8        ggagccccccatgattgcctcgagatcttggctgaaacgcatggaaccagaccggaCctcaccgaccagcccatcccaga
Xmv9        ggagccccccatgattgcctcgagGtcttggctgaaacgcatggaaccagaccggaCctcaccgaccagcccatcccaga
```

```
XMV_CONS    CGCCGACCACACCTGGTATACCGATGGGAGCAGCTTTTTGCAAGAAGGACAGCGAAAGGCTGGGGCAGCAGTGACGACTG
Xmv10       cgccgaccacacctggtataccgatgggagcagctttttgcaagaaggacagcgaaaggctggggcagcagtgacgactg
Xmv12       cgccgaccacacctggtataccgatgggagcagctttttgcaagaaggacagcgaaaggctggggcagcagtgacgactg
Xmv13       cgccgaccacacctggtataccgatgggagcagctttttgcaagaaggacagcgaaaAgctggggcagcagtgacgacAg
Xmv15       cgccgaccacacctggtataccgatgggagcagctttttgcaagaaggacagcgaaaggctggggcagcagtgacgactg
Xmv16       cgccgaccacacctggtataccgatgggagcagctttttgcaagaaggacagcgaaaAgctggggcagcagtgacgacAg
Xmv17       cgccgaccacaccCggtataccgatgggagcagctttttgcaagaaggacagcgaaaggctggggcagcagtgacgactg
Xmv18       cgccgaccacacctggtataccgatgggagcagctttttgcaagaaggacagcgaaaggctggggcagcagtgacgactg
Xmv19       cgccgaccacacctggtataccgatgggagcagctttttgcaagaaggacagcgaaaggctggggcagcagtgacgactg
Xmv41       cgccgaccacacctggtatacAgatggAagcagcttCCtAcaagaaggacagcgGaGAgctggAgcCgcGgtgacCactg
Xmv42       --------------------------------------------------------------------------------
Xmv43       cgccgaccacacctggtataccgatgggagcagctttCtgcaagaaggacagcgaaaggctggggcagcagtgacgactg
Xmv8        cgccgaccacacctggtataccgatgggagcagctttttgcaagaaggacagcgaaaAgctggggcagcagtgacgacAg
Xmv9        cgccgaccacacctggtataccgatgggagcagctttttgcaagaaggacagcgaaaAgctggggcagcagtgacgacAg

XMV_CONS    ARACCGAGGTAATCTGGGCGAGGGCCCTGCCAGCTGGAACGTCAGCCCAGCGAGCCGAACTGATCGCACTCACCCAAGCC
Xmv10       aGaccgaggtaatctgggcgagggccctgccagctggaacgtcagcccagcgagccgaactgatcgcactcacccaagcc
Xmv12       aAaccgaggtaatctgggcgagggccctgccagctggaacgtcagcccagcgagccgaactgGtcgcactcacccaagcc
Xmv13       aGaccgaggtaatctgggcgagggccctgccagctggaacgtcagcccagcgagccgaactgatcgcactcacccaagcc
Xmv15       aAaccgaggtaatctgggcgagggccctgccagctggaacgtcagcccagcgagccgaactgatcgcactcacccaagcc
Xmv16       aGaccgaggtaatctgggcgagggccctgccagctggaacgtcagcccagcgagccgaactgatcgcactcacccaagcc
Xmv17       aAaccgaggtaatctgggcgagggccctgccagctggaacgtcagcccagcgagccgaactgatcgcactcacccaagcc
Xmv18       aAaccgaggtaatctgggcgagggccctgccagctggaacgtcagcccagcgagccgaactgatcgcactcacccaagcc
Xmv19       aAaccgaggtaatctgggcgagggccctgccagctggaacgtcagcccagcgagccgaactgatcgcactcacccaagcc
Xmv41       aGaccgaggtaatctgggcAagggcActgccGgctggaacAtcCgcccagcgagccAaactgatAgcactcacccaagcc
Xmv42       --------------------------------------------------------------------------------
Xmv43       aAaccgaggtaatctgggcgagggccctgccagctggaacgtcagcccagcgagccgaactgatcgcactcacccaagcc
Xmv8        aGaccgaggtaatctgggcgagggccctgccagctggaacgtcagcccagcgagccgaactgatcgcactcacccaagcc
Xmv9        aGaccgaggtaatctgggcgagggccctgccagctggaacgtcagcccagcgagccgaactgatcgcactcacccaagcc

XMV_CONS    CTGAAAATGGCAGAAGGTAAGAAGCTAAATGTTTACACTGATAGCCGCTATGCCTTCGCTACGGCCCATGTTCATGGGGA
Xmv10       ctgaaaatggcagaaggtaagaagctaaatgtttaTactgaCagccgAtatgcTttcgcCacggcccatgtCcatggAga
Xmv12       ctgaaaatggcagaaggtaagaagctaaatgtttacactgatagccgctatgccttcgctacggcccatgttcatgggga
Xmv13       ctgaaaatggcagaaggtaagaagctaaatgtttaTactgaCagccgAtatgcTttcgcCacggcccatgtCcatggAga
Xmv15       ctgaaaatggcagaaggtaagaagctaaatgtttacactgatagccgctatgccttcgctacggcccatgttcatgggga
Xmv16       ctgaaaatggcagaaggtaagaagctaaatgtttaTactgaCagccgAtatgcTttcgcCacggcccatgtCcatggAga
Xmv17       ctgaaaatggcagaaggtaagaagctaaatgtttacactgatagccgctatgccttcgctacggcccatgttcatgggAa
Xmv18       ctgaaaatggcagaaggtaagaagctaaatgtttacactgatagccgctatgccttcgctacggcccatgttcatgggga
Xmv19       ctgaaaatggcagaaggtaagaagctaaatgtttacactgatagccgctatgccttcgctacggcccatgttcatgggga
Xmv41       TtAaaaatggcagaaggtaagaagctaaaCgtttacactgatagccgctatgccttcgcCacggcccatgtCcatggAAa
Xmv42       --------------------------------------------------------------------------------
Xmv43       ctgaaaatggcagaaggtaagaagctaaatgtttacactgatagccgctatgccttcgctacggcccatgttcatgggga
Xmv8        ctgaaaatggcagaaggtaagaagctaaatgtttacactgaCagccgAtatgcTttcgcCacggcccatgtCcatggAga
Xmv9        ctgaaaatggcagaaggtaagaagctaaatgtttaTactgatagccgctatgccttcgctacggcccatgttcatgggga

XMV_CONS    AATATATAGGAGACGGGGGTTGCTGACCTCAGAAGGCAAGGAAATCAAGAACAAAAGCGAGATCCTAGCCTTGCTGAAAG
Xmv10       aatCtataggagGcgAgggttgctgacctcagaGggcaGAgaaatcaaAaacaaGagcgagatcctGgcTttActgaaag
Xmv12       aatatataggagacgggggttgctgacctcagaaggcaaggaaatcaagaacaaaagcgagatcctagccttgctgaaag
Xmv13       aatCtataggagGcgAgggttgctgacctcagaGggcaGAgaaatcaaAaacaaGagcgagatcctGgcTttActgaaag
Xmv15       aatatataggagacgggggttgctgacctcagaaggcaaggaaatcaagaacaaaagcgagatcctagccttgctgaaag
Xmv16       aatCtataggagGcgAgggttgctgacctcagaGggcaGAgaaatcaaAaacaaGagcgagatcctGgcTttActgaaag
Xmv17       aatatataggagacgggggttgctgacctcagaaggcaaggaaatcaagaacaaaagcgagatcctagccttgctgaaag
Xmv18       aatatataggagacgggggttgctgacctcagaaggcaaggaaatcaagaacaaaagcgagatcctagccttgctgaaag
Xmv19       aatatataggagacgggggttgctgacctcagaaggcaaggaaatcaagaacaaaagcgagatcctagccttgctgaaag
Xmv41       aatatataggagGcgAgggttgctgacctcagaaggcaGAgaaatTaaAaacaaaagcgagatcTtGgccttgctAaaag
Xmv42       --------------------------------------------------------------------------------
Xmv43       aatatataggagacgggggttgctgacctcagaaggcaaggaaatcaagaacaaaagcgagatcctagccttgctgaaag
Xmv8        aatCtataggagGcgAgggttgctgacctcagaGggcaGAgaaatcaaAaacaaGagcgagatcctGgcTttActgaaag
Xmv9        aatatataggagacgggggttgctgacctcagaaggcaaggaaatcaagaacaaaagcgagatcctagccttgctgaaag
```

```
XMV_CONS    CCCTCTTTTTGCCAAAGAGRCTCAGTATTATCCATTGCCCVGGACAYCAAAAAGGRAACAGTGCTGAAGCCAGGGGCAAC
Xmv10       cTctTttCCtgccTaaAagActcagtatAatTcaCtgcccCggGcaTcaaaaGggAaacagtgctgaagccaggggcaac
Xmv12       ccctctttttgccaaagagGctcagtattatccattgcccGggacaCcaaaaaggGaacagtgctgaagccaggggcaac
Xmv13       cTctTttCCtgccTaaAagActcagtatAatTcaCtgcccCggGcaTcaaaaGggAaacagtgctgaagccaggggcaac
Xmv15       ccctctttttgccaaagagGctcagtattatccattgcccGggacaCcaaaaaggGaacagtgctgaagccaggggcaac
Xmv16       cTctTttCCtgccTaaAagActcagtatAatTcaCtgcccCggGcaTcaaaaGggAaacagtgctgaagccaggggcaac
Xmv17       ccctctttttgccaaagagGctcagtattatccattgcccGggacaCcaaaaaggGaacagtgctgaagccaggggcaac
Xmv18       ccctctttttgccaaagagGctcagtattatccattgcccGggacaCcaaaaaggGaacagtgctgaagccaggggcaac
Xmv19       ccctctttttgccaaagagGctcagtattatccattgcccGggacaCcaaaaaggGaacagtgctgaagccaggggcaac
Xmv41       cTctctttCtgccCaaAagActTagtatAatTcattgTccAggacaTcaGaaaAgAGacagtgcCgaagccagAggcaac
Xmv42       --------------------------------------------------------------------------------
Xmv43       ccctctttttgccaaagagActcagtattatccattgcccAggacaTcaGaaaggAGacagtgcCgaagccagAggcaac
Xmv8        cTctTttCCtgccTaaAagActcagtatAatTcaCtgcccCggGcaTcaaaaGggAaacagtgctgaagccaggggcaac
Xmv9        ccctctttttgccaaagagGctcagtattatccattgcccGggacaCcaaaaaggGaacagtgctgaagccaggggcaac

XMV_CONS    CGWATGGCRGAYCAAGCRGCCMGRGAGGCAGCCATRAGRRVRDBYVCAGAAACTTCCACVCTCYTHATAGAGGAYTCGWC
Xmv10       cgTatggcAgaCcaagcGgccCgAgaggcagccatA--------------------------------------------
Xmv12       cgAatggcGgaTcaagcAgccAgGgaggcagccatGagAGAGATCAcagaaacttccacGctcTtAatagaggaTtcgTc
Xmv13       cgTatggcAgaCcaagcGgccCgAgaggcagccatAagGACATCTCcagaaacttccacCctcCtCatagaggaCtcgAc
Xmv15       cgAatggcGgaTcaagcAgccAgGgaggcagccatGagAGAGATCAcagaaacttccacGctcTtAatagaggaTtcgTc
Xmv16       cgTatggcAgaCcaagcGgccCgAgaggcagccatAagGACATCTCcagaaacttccacCctcCtCatagaggaCtcgAc
Xmv17       cgAatggcGgaTcaagcAgccAgGgaggcagccatG--------------------------------------------
Xmv18       cgAatggcGgaTcaagcAgccAgGgaggcagccatGagAGAGATCAcagaaacttccacGctcTtAatagaggaTtcgTc
Xmv19       cgAatggcGgaTcaagcAgccAgGgaggcagccatGagAGGGATCAcagaaacttccacGctcTtAatagaggaTtcgTc
Xmv41       cgTatggcAgaCcaGgcGgccCgAgaggcagccaCAaAGACAGTTCcagaaGcCtcTacActcCtTatagaggaCtcgAc
Xmv42       --------------------------------------------------------------------------------
Xmv43       cgTatggcAgaCcaGgcGgccCgAgaggcagccaCAaAAACAGTTCcagaaGcCtcTacActcCtTatagaggaCtcgAc
Xmv8        cgTatggcAgaCcaagcGgccCgAgaggcagccatAagGACATCTCcagaaacttccacCctcCtCatagaggaCtcgAc
Xmv9        cgAatggcGgaTcaagcAgccAgGgaggcagccatGagAGAGAGCGcagaaacGtccacGctcTtAatagaggaTtcgTc

XMV_CONS    CCCGTATACKCCCDSYTAYTTCCACTACACHGARACWGAYMKAAAARRMYTDCKRRRACTGGGAGCCRYMTAYRATSAGR
Xmv10       --------------------------------------------------------------------------------
Xmv12       cccgtatacTcccAGTtaCttccactacacAgaGacTgaCAGaaaaGACCtTcTGAGactgggagccGTAtaCGatGagG
Xmv13       cccgtatacGcccTCCCaTttccactacacTgaAacAgaTCTaaaGAGATtAcGAGAactgggagccACCtaTAatCagA
Xmv15       cccgtatacTcccAGTtaCttccactacacAgaGacTgaCAGaaaaGACCtTcTGAGactgggagccGTAtaCGatGagG
Xmv16       cccgtatacGcccTCCCaTttccactacacTgaAacAgaTCTaaaGAGATtAcGAGAactgggagccACCtaTAatCagA
Xmv17       --------------------------------------------------------------------------------
Xmv18       TccgtatacTcccAGTtaCttccactacacAgaGacTgaCAGaaaaGACCtTcTGAGactgggagccGTAtaCGatGagG
Xmv19       cccgtatacTcccAGTtaCttccactacacAgaGacTgaCAGaaaaGACCtTcTGAGactgggagccGTAtaCGatGagG
Xmv41       cccgtaCacGccTGCCtaTttccaTtacacCgaAacAgaTCTaaaaAGATtAcGAGAactgggagccACCtaTAaCCagA
Xmv42       --------------------------------------------------------------------------------
Xmv43       cccgtaCacGccTGCCtaTCtccaTtacacCgaAacAgaTCTaaaaAGATtGcGAGAactgggGgccACCtaTAatCagA
Xmv8        cccgtatacGcccTCCCaTttccactacacTgaAacAgaTCTaaaGAGATtAcGAGAactgggagccACCtaTAatCagA
Xmv9        cccgtatacTcccAGCtaCttccactacacAgaGacTgaCAGaaaaGACCtTcTGAGactgggagccGTAtaCGatGagG

XMV_CONS    WMAAARGRTATTGGGTCCTRCARGGMAARCCKGTAATGCCYGAYCARTTYRYSTTTGARCTMTTAGACTCCCTACACMGG
Xmv10       --------tattgggtcctAcaAggCaaGccGgtaatgccCgaTcaGttTGTGtttgaActAttagactccTtacacAgg
Xmv12       ACaaaAgGtattgggtcctGcaGggAaaAccTgtaatgccTgaCcaAttCACCtttAaGctCttagactccctacacCgg
Xmv13       TAaaaGgAtattgggtcctAcaAggCaaGccGgtaatgccCgaTcaGttTGTGtttgaActAttagactccTtacacAgg
Xmv15       ACaaaAgGtattgggtcctGcaGggAaaAccTgtaatgccTgaCcaAttCACCtttgaGctCttagactccctacacCgg
Xmv16       TAaaaGgAtattgggtcctAcaAggCaaGccGgtaatgccCgaTcaGttTGTGtttgaActAttagactccTtacacAgg
Xmv17       --------tattgggtcctGcaGggAaaAccTgtaatgccTgaCcaAttCACCtttgaGctCttagactccctacacCgg
Xmv18       ACaaaAgGtattgggtcctGcaGggAaaAccTgtaatgccTgaCcaAttCACCtttgaGctCttagactccctacacCgg
Xmv19       ACaaaAgGtattgggtcctGcaGggAaaAccTgtaatgccTgaCcaAttCACCtttgaGctCttagactccctacacCgg
Xmv41       TAaaaGgAtattgggtcctAcaAggCaaGccGgtGatgccCgaTcaGttTGTGtttgaActAttagactccctTcacAgA
Xmv42       --------------------------------------------------------------------------------
Xmv43       TAaaaGgAtattgggtcctAcaAggCaaGccGgtGatgccCgaTcaGttTGTGtttgaATtAttagactccctTcaTAgA
Xmv8        TAaaaGgAtattgggtTctAcaAggCaaGccGgtaatgccCgaTcaGttTGTGtttgaActAttagactccTtacacAgg
Xmv9        ACaaaAAGtattgggtcctGcaGggAaaAccTgtGatgccTgaCcaAttCACCtttgaGctCttagaTtccctacacCgg
```

```
XMV_CONS    CTCACCCACCTCAGCYMTCARAAGATGAAGGCACTCCTTGACMGRGAAGARAGCCCCTACTACATGTTAAATMGRGAYAG
Xmv10       ctcacTcacctcagcCCtcaAaagatgaaggcactccttgacAgAgaagaAagcccctactacatgttaaat--------
Xmv12       ctcacccacctcaAcTAtcaGaagatgaaggcactccttgacCgGgaagaGagcccctactacatgttaaatCgGgaTaA
Xmv13       ctcacTcacctcagcCCtcaAaagatgaaggcactccttgacAgAgaagaAagcccctactacatgttaaaCAgAgaCag
Xmv15       ctcacccacctcagcTAtcaGaagatgaaggcactccttgacCgGgaagaGagcccctactacatgttaaatCgGgGTag
Xmv16       ctcacTcacctcagcCCtcaAaagatgaaggcactccttgacAgAgaagaAagcccctactacatgttaaaCAgAgaCag
Xmv17       ctcacccacctcagcTAtcaGaGgatgaaggcactccttgacCgGgaagaGagcccctactacatgttaaat--------
Xmv18       ctcacccacctcagcTAtcaGaagatgaaggcactccttgacCgGgaagaGagcccctactacatgttaaatCgGgaTag
Xmv19       ctcacccacctcagcTAtcaGaagatgaaggcactccttgacCgGgaagaGagcccctactacatgttaaatCgGgaTag
Xmv41       ctcacccaTctcagcCCtcaAaagatgaaggcactccttgacAgAgaagaAagcccctactacatgttaaaCCgAgaCag
Xmv42       --------------------------------------------------------------------------------
Xmv43       ctcacccaTctcagcCCtcaAaagatgaaggcGctccttgacAgAgaagaAagcccctactacatgttaaaCAgGgaCag
Xmv8        ctcacTcacctcagcCCtcaAaagatgaaggcactccttgacAgAgaagaAagcccctactacatgttaaaCAgAgaCag
Xmv9        ctcacccacctcagcTAtcaGaagatgaaggcactccttgacCgGgaagaGagcccctactacatgttaaatCgGgaTaA

XMV_CONS    AACTCTCCAGYAYGTRRCAGAATCCTGYACAGCTTGTGCTCAAGTRAATGCTAGTAAAGCCAAAATCGGRGCARGGGTRC
Xmv10       --------------------------------------------------------------------------------
Xmv12       aactctccagCaCgtAAcagaatcctgTacagcttgtgctcaagtAaatgctagtaaagccaaaatcggAgcaAgggtGc
Xmv13       aactctccagTaTgtGGcagaatcAtgCacagcttgtgctcaagtGaatgctagtaaagccaaGatcggGgcaGgggtAc
Xmv15       aactctccagCaCgtAAcagaatcctgTacagcttgtgctcaagtAaatgctagtaaagccaaaatcggAgcaAgggtGc
Xmv16       aactctccagTaTgtGGcagaatcAtgCacagcttgtgctcaagtGaatgctagtaaagccaaGatcggGgcaGgggtAc
Xmv17       --------------------------------------------------------------------------------
Xmv18       aactctccagCaCgtAAcagaatcctgTacagcttgtgctcaagtAaatgctagtaaagccaaaatcggAgcaAgggtGc
Xmv19       aactctccagCaCgtAAcagaatcctgTacagcttgtgctcaagtAaatgctagtaaagccaaaatcggAgcaAgggtGc
Xmv41       aactctTcagTaTgtGGcGgaatcctgCacaActtgtgctcaagtGaatgctagtaaagccaaaatcggGgcaGgggtAc
Xmv42       --------------------------------------------------------------------------------
Xmv43       aactctTcagTaTgtGGcagaatcctgCacagTCtgtgctcaagtAaatgctagtaaagccaaaatcggGgcaGgggtAc
Xmv8        aactctccagTaTgtGGcagaatcAtgCacagcttgtgctcaagtGaatgctagtaaagccaaGatcggGgcaGgggtAc
Xmv9        aactctccagCaCgtAAcGgaatcctgTacagcttgtgctcaagtAaatgctagtaaagccaaaatcggAgcaAgggtGc

XMV_CONS    GAGYRCGSGGACAYCGACCMGGYACCCATTGGGAAATYGAYTTTACYGAAGTTAAGCCAGGGCTGTAYGGRTAYAAGTAC
Xmv10       -----------------------acccattgggaaatTgaCtttacTgaagttaagccaggActgtaCggGtaCaagtac
Xmv12       gagCGcgGggacaCcgaccCggCacccattgggaaatCgaTtttacCgaagttaagccagggctgtaTggACaTaagtaT
Xmv13       gagTAcgCggacaTcgaccAggTacccattgggaaatTgaCtttacTgaagttaagccaggActgtaCggGtaCaagtac
Xmv15       gagCGcgGggacaCcgaccCggCacccattgggaaatCgaTtttacCgaagttaagccagggctgtaTggACaTaagtac
Xmv16       gagTAcgCggacaTcgaccAggTacccattgggaaatTgaCtttacTgaagttaagccaggActgtaCggGtaCaaAtac
Xmv17       --------ggacaCcgaccCggCacccattgAgaaatCgaTtttacCgaagttaagccagggctgtaTggACaTaagtaT
Xmv18       gagCGcgGggacaCcgaccCggCacccattgggaaatCgaTtttacCgaagttaagccagggctgtaTggACaTaagtaT
Xmv19       gagCGcgGggacaCcgaccCggCacccattgggaaatCgaTtttacCgaagttaagccagggctgtaTggACaTaagtaT
Xmv41       gagTAcgCggacaTcgaccAggTacccattgggaaatTgaCttCacTgaagttaaAccagggctgtaCggGtaCaagtac
Xmv42       --------------------------------------------------------------------------------
Xmv43       gagTAcgCggacaTcgaccAggTacccattgggaaatTgaCttCacTgaagttaaAccagggctgtaCggGtaCaagtac
Xmv8        gagTAcgCggacaTcgaccAggTacccattgggaaatTgaCtttacTgaagttaagccaggActgtaCggGtaCaagtac
Xmv9        gagCGcgGggacaCcgaccCggCacccattgggaaatCgaTtttacCgaagttaagccagggctgtaTggAtaTaagtaT

XMV_CONS    CTCCTGGTGTTYGTGGACACMTTCTCTGGCTGGGTAGAAGCMTTYCCAACYAARCGTGAAACTGCCARGGTTGTGACCAA
Xmv10       ctcctAgtgttCgtggacacCttctctggctgggtagaagcCttCccaacTaaAcgtgaaacCgccaAggtGgtAaccaa
Xmv12       ctcctggtgttTgtggacacAttctctggctgggtagaagcAttTccaacCaaGcgtgaaactgccaGggttgtgaccaa
Xmv13       ctcctAgtgttCgtggacacCttctctggctgggtagaagcCttCccaacTaaAcgtgaaacCgccaAAgtGgtAaccaa
Xmv15       ctcctggtgttTgtggacacAttctctggctgggtagaagcAttTccaacCaaGcgtgaaactgccaGggttgtgaccaa
Xmv16       ctcctAgtgttCgtggacacCttctctggctgggtagaagcCttCccaacTaaAcgtgaaacCgccaAggtGgtAaccaa
Xmv17       ctcctggtgttTgtggacacAttctctggctgggtagaagcAttTccaacCaaGcgtgaaactgccaGggttgtgaccaa
Xmv18       ctcctggtgttTgtggacacAttctctggctgggtagaagcAttTccaacCaaGcgtgaaactgccaGggttgtgaccaa
Xmv19       ctcctggtgttTgtggacacAttctctggctgggtagaagcAttTccaacCaaGcgtgaaactgccaGggttgtgaccaa
Xmv41       ctcctggtgttCgtAgacacCttctctggctgggtGAaagcCttCccaacTaaAcgtgaaactgccaAggttgtAaccaa
Xmv42       --------------------------------------------------------------------------------
Xmv43       ctcctggtgttCgtAgacacCttctctggctgggtGgaagcCttCccaacTaaAcgtgaaactgccaAggttgtgaccaa
Xmv8        ctcctAgtgttCgtggacacCttctctggctgggtagaagcCttCccaacTaaAcgtgaaacCgccaAggtGgtAaccaa
Xmv9        ctcctggtgttTgtggacacAttctctggctgggtagaagcAttTccaacCaaGcgtgaaactgccaGggttgtgaccaa
```

```
XMV_CONS   GAAGCTRYTWGAAGAAATWTTYCCAAGATTCGGGATGCCMCAGGTATTGGGDWCHGATAATGGGCCTGCCTTCRTCTCCC
Xmv10      gaagctATtAgaagaaatAttCccaagattcgggatgccAcaggtaCtgggTTcTgaCaatgggcctgccttcGtctccc
Xmv12      gaagctGCtTgaagaaatTttTccaagattcgggatgccCcaggtattgggAAcAgataatgggcctgccttcAtctccc
Xmv13      gaagctATtAgaagaaatAttCccaagattcgggatgccAcaggtaCtgggTTcTgaCaatgggcctgccttcGtctccc
Xmv15      gaagctGCtTgaagaaatTttTccaagattcgggatgccCcaggtattgggGAcAgataatgggcctgccttcAtctccc
Xmv16      gaagctATtAgaagaaatAttCccaagattcgggatgccAcaggtaCtgggTTcTgaCaatgggcctgccttcGtctccc
Xmv17      gaagctGCtTgaagaaatTttTccaagattcgggatgccCcaggtattgggGAcAgataatgggcctgccttcAtctccc
Xmv18      gaagctGCtTgaagaaatTttTccaagattcgggatgccCcaggtattgggGAcAgataatgggcctgccttcAtctccc
Xmv19      gaagctGCtTgaagaaatTttTccaagattcgggatgccCcaggtattgggGAcAgataatgggcctgccttcAtctccc
Xmv41      gaagctATtAgaagaaatAttCccaagattcgggatgccAcaggtattgggTTcCgataatgggcctgccttcGtctccc
Xmv42      --------------------------------------------------------------------------------
Xmv43      gaagctATtAgaagaaatAttCccaagattcgggatgccAcaggtattgggTTcCgataatgggcctgccttcGtctccc
Xmv8       gaagctATtAgaagaaatAttCccaagattcgggatgccAcaggtaCtgggTTcTgaCaatgggcctgccttcGtctccc
Xmv9       gaagctGCtTgaagaaatTttTccaagattcgggatgccCcaggtattgggGAcAgataatgggcctgccttcAtctccc

XMV_CONS   AGGTAAGTCAGTCGGTGGCCGATTTRCTGGGGATTGATTGGAAAYTRCATTGTGCTTATAGACCCCAGAGTTCAGGTCAG
Xmv10      aggtaagtcaTtcggtggccgatttActggggattgattggaaGTtAcattgtgcttatagaccccagagttcaggtcag
Xmv12      aggtaagtcagtcggtggccAaGttGctggggattgattggaaaCtGcattgtgcttaCagaccccagagttcaggtcag
Xmv13      aggtaagtcaTtcggtggccgatttActggggattgattggaaGTtAcattgCgcttatagaccccagagttcaggtcag
Xmv15      aggtaagtcagtcggtggccAaGttGctggggattgattggaaaCtGcattgtgcttaCagaccccagagttcaggtcag
Xmv16      aggtaagtcaTtcggtggccgatttActggggattgattggaaGTtAcattgtgcttatagaccccagagttcaggtcag
Xmv17      aggtaagtcagtcggtggccAaGttGctggggattgattggaaaCtGcattgtgcttaCagaccccagagttcaggtcag
Xmv18      aggtaagtcagtcggtggccAaGttGctggggattgattggaaaCtGcattgtgcttaCagaccccagagttcaggtcag
Xmv19      aggtaagtcagtcggtggccAaGttGctggggattgattggaaaCtGcattgtgcttaCagaccccagagttcaggtcag
Xmv41      aggtaagtcagtcggtggccgatttActggggatCgattAgaaaTtAcattgtgcttatagaccccagagttcaggtcag
Xmv42      --------------------------------------------------------------------------------
Xmv43      aggtaagtcagtcggtggccgatttActggggatCgattggaaaTtAcattgtgcttatagaccccagagttcaggtcag
Xmv8       aAgtaagtcaTtcggtggccgatttActggggattgattggaaGTtAcattgtgcttatagaccccagagttcaggtcag
Xmv9       aggtaagtcagAcAgtggccgatttGTtggggatCgattggaaaCtGcattgtgcttatagaccccagagttcaggtcag

XMV_CONS   GTAGAAAGAATGAATAGAACMATCAAGGAGACTTTAACCAAATTAACGCTTGCAGCTGGCACTAGAGACTGGGTACTCCT
Xmv10      gtagaGagaatgaatagaacCatcaaggagactttaaccaaattaacgctGgcagctggcactagagactgggtactcct
Xmv12      gtagaaagaatgaaCagGacAatcaaggagactttaaccaaattaacgcttgcagctggcactagagactgggtactcct
Xmv13      gtagaGagaatgaatagaacCatcaaggagactttaaccaaattaacgcttgcagctggcactagagactgggtactcct
Xmv15      gtagaaagaatgaaCagGacAatcaaggagactttaaccaaattaacgcttgcagctggcactagagactgggtactcct
Xmv16      gtagaGagaatgaatagaacCatcaaggagactttaaccaaattaacgcttgcagctggcactagagactgggtactcct
Xmv17      gtaAaaaAaatgaaCagGacAatcaaggagactttaaccaaattaacgcttgcagctggcactagagactgggtactcct
Xmv18      gtagaaagaatgaaCagGacAatcaaggagactttaaccaaattaacgcttgcagctggcactagagactgggtactcct
Xmv19      gtagaaagaatgaaCagGacAatcaaggagactttaaccaaattaacgcttgcagctggcactagagactgggtactcct
Xmv41      gtaAaaagaatAaatagaacCatcaaggagactCtaacTaaattaacgcttgcagctggcactagagactgggtactcct
Xmv42      --------------------------------------------------------------------------------
Xmv43      gtagaaagaatgaatagaacCatcaaggagactCtaacTaaattaacgcttgcagctggcactagagactgggtactcct
Xmv8       gtagaGagaatgaatagaacCatcaaggagactttaaccaaattaacgcttgcagctggcactagagactgggtactcct
Xmv9       gtagaaagaatgaatagaacAatcaaggagactttGaccaaattaacgcttgcagctggcactagagactgggtactcct

XMV_CONS   ACTCCCCCTGGCCCTCTACCGAGCCCGGAATACTCCGGGCCCCCATGGACTTACTCCGTATGAAATTCTGTATGGGGCAC
Xmv10      actccccctggccctctaccgagcccggaatactccgggcccccatggacttactccgtatgaaatCctgtatggggcac
Xmv12      actccccctggccctctaccgagcccggaatactccgggcccccatggacttactccgtatgaaattctgtatggggcac
Xmv13      actccccTtggccctctaccgagcccggaatactccgggcccAcatggacttactccgtatgaaattctgtatggggcac
Xmv15      actccccctggccctctaccgagcccggaatactccgggcccccatggacttactccgtatgaaattctgtatggggcac
Xmv16      actccccctggccctctaccgagcccggaatactccgggcccccatggacttactccgtatgaaatCctgtatggggcac
Xmv17      actccccctggccctctaccgagcccggaatactccgggcccccatggacttactccgtatgaaattctgtatggggcac
Xmv18      actccccctggccctctaccgagcccggaatactccgggcccccatggacttactccgtatgaaattctgtatggggcac
Xmv19      actccccctggccctctaccgagcccggaatactccgggcccccatggacttactccgtatgaaattctgtatggggcac
Xmv41      actccccTtAgccctctaccgagcccggaaCactccgggcccccatggactGacCccgtatgaaattctgtatggggcac
Xmv42      --------------------------------------------------------------------------------
Xmv43      actccccTtAgccctctaccgagcccggaaCactccgggcccccatggactGactccgtatgaaattctgtatggggcac
Xmv8       actccccctggccctctaccgagcccggaatactccgggcccccatggacttactccgtatgaaatCctgtatggggcac
Xmv9       actccccctggccctctaccgagcccggaatactccgggcccAcatggactCactccgtatgaaattctgtatggggcac
```

```
XMV_CONS     CCCCGCCCCTTGTCAATTTTCATGATCCTGAAATGTCAAAGTTAACTAATAGTCCCTCTCTCCAAGCTCACTTACAGGCC
Xmv10        ccccgcccctCgtcaattttcatgatcctgaaatgtcaaagttaactaatagtccctctctccaagctcacttacaggcc
Xmv12        ccccgccccttgtcaattttcatgatcctgaaatgtcaaagttaactaatagtccctctctccaagctcacttacaggcc
Xmv13        ccccgccccttgtcaattttcatgatcctgaaatgtcaaagttaactaatagtccctctctccaagctcacttacaggcc
Xmv15        ccccgccccttgtcaattttcatgatcctgaaatgtcaaagttaactaatagtccctctctccaagctcacttacaggcc
Xmv16        ccccgcccctCgtcaattttcatgaGcctgaaatgtcaaagttaactaatagtccctctctccaagctcacttacaggcc
Xmv17        ccccgccccttgtcaattttcatgatcctgaaatgtcaaagttaactaatagtccctctctccaagctcacttacaggcc
Xmv18        ccccgccccttgtcaattttcatgatcctgaaatgtcaaagttaactaatagtccctctctccaagctcacttacaggcc
Xmv19        ccccgccccttgtcaattttcatgatcctgaaatgtcaaagttaactaatagtccctctctccaagctcacttacaggcc
Xmv41        ccccgccccttgtcaattttcatgatcctgaaatgtcaaagttaactaatagtccctctctccaagctcacttacaggcc
Xmv42        --------------------------------------------------------------caagctcacttacaggcc
Xmv43        ccccgccccttgtcaattttcatgatcctgaaatgtcaaagttaactaatagtccctctctccaagctcacttacaggcc
Xmv8         ccccgcccctCgtcaattttcatgatcctgaaatgtcaaagttaactaatagtccctctctccaagctcacttacaggcc
Xmv9         ccAcgccccttgtcaattttcatgatcctgaaatgtcaaagttaactaatagtccctctctccaagctcacttacaggcc

XMV_CONS     CTCCAAGCAGTACAACGAGAGGTCTGGAAGCCACTGGCCGCTGCTTATCAGGACCAGCTGGATCAGCCAGTGATACCACA
Xmv10        ctccaagcagtacaacgagaggtctggaagccactggccgctgcttatcaggaccagctggatcagccagtgataccaca
Xmv12        ctccaagcagtacaacgagaggtctggaagccactggccgctgcttatcaggaccagctggatcagccagtgataccaca
Xmv13        ctccaagcagtacaacgagaggtctggaagccactggccgctgcttatcaggaccagctggatcagccagtgataccaca
Xmv15        ctccaagcagtacaacgagaggtctggaagccactggccgctgcttatcaggaccagctggatcagccagtgataccaca
Xmv16        ctccaagcagtacaacgagaggtctggaagccactggccgctgcttatcaggaccagctggatcagccagtgataccaca
Xmv17        ctccaagcagtacaacgagaggtctggaagccactggccgctgcttatcaggaccagctggatcagccagtgataccaca
Xmv18        ctccaagcagtacaacgagaggtctggaagccactggccgctgcttatcaggaccagctggatcagccagtgataccaca
Xmv19        ctccaagcagtacaacgagaggtctggaagccactggccgctgcttatcaggaccagctggatcagccagtgataccaca
Xmv41        ctccaagcagtacaacgagaggtctggaagccGctggccgctgcttatcGggaccagctAgatcagccagtgataccaca
Xmv42        ctccaagcagtacaacgagaggtctggaGgccactggcTgctgcttatcaggaccagctggatcagccagtgataccaca
Xmv43        ctccaagcagtacaacgagaggtctggaagccGctggccgctgcttatcaggaccagctAgatcagccagtgataccaca
Xmv8         ctccaagcagtacaacgagaggtctggaagccactggccgctgcttatcaggaccagctggatcagccagtgataccaca
Xmv9         ctccaagcagtacaacgagaggtctggaagccGctggccgctgcttatcaggaccagctggatcagccagtgataccaca

XMV_CONS     CCCCTTCCGTGTCGGTGACGCCGTGTGGGTACGCCGGCACCAGACTAAGAACTTAGAACCCCGCTGGAAAGGACCCTACA
Xmv10        ccccttccgtgtcggtgacgccgtgtgggtacgccggcaccagactaagaacttagaaccccgctggaaaggaccctaca
Xmv12        ccccttccgCgtcggtgacgccgtgtgggtacgccggcaccagactaagaacttagaaccccgctggaaaggaccctaca
Xmv13        ccccttccgtgtcggtgacgccgtgtgggtacgccggcaccagactaagaacttagaaccccgctggaaaggaccctaca
Xmv15        ccccttccgCgtcggtgacgccgtgtgggtacgccggcaccagactaagaacttagaaccccgctggaaaggaccctaca
Xmv16        ccccttccgtgtcggtgacgccgtgtgggtacgccggcaccagactaagaacttagaaccccgctggaaaggaccctaca
Xmv17        ccccttccgCgtcggtgacgccgtgtgggtacgccggcaccagactaagaacttagaaccccgctgAaaaggaccctaca
Xmv18        ccccttccgCgtcggtgacgccgtgtgggtacgccggcaccagactaagaacttagaaccccgctggaaaggaccctaca
Xmv19        ccccttccgCgtcggtgacgccgtgtgggtacgccgAcaccagactaagaacttagaaccccgctggaaaggaccctaca
Xmv41        ccccttccgtgtcggtgacgccgtgtgggtacgccggcaccagactaagaacttGgaaccTcgctggaaaggaccctaca
Xmv42        ccccttccgtgtcggtgacgccgtgtgggtacgccggcaccagactaagaacttagaaccccgctggaaaggaccctaca
Xmv43        ccccttccgtgtcggtgacgccgtgtgggtacgccggcaccagactaagaacttGgaaccTcgctggaaaggaccctaca
Xmv8         ccccttccgtgtcggtgacgccgtgtgggtacgccggcaccagactaagaacCtagaaccTcgctggaaaggaccctaca
Xmv9         ccccttccgtgtcggtgacgccgtgtgggtGcgccggcaTcagactaagaacCtagaaccTcgctggaaaggaccctaca

XMV_CONS     CCGTCCTGCTGACCACCCCCACCGCTCTCAAAGTAGACGGCATCTCTGCGTGGATACACGCCGCTCACGTAAAGGCGGCG
Xmv10        ccgtcctgctgaccacccccaccgctctcaaagtagacggcatctctgcgtggatacacgccgctcacgtaaaggcggcg
Xmv12        ccgtcctgctgaccacccccaccgctctcaaagtagacggcatctctgcgtggatacacgccgctcacgtaaaggcggcg
Xmv13        ccgtcctgctgaccacccccaccgctctcaaagtagacggcatctctgcgtggatacacgccgctcacgtaaaggcggcg
Xmv15        ccgtcctgctgaccacccccaccgctctcaaagtagacggcatctctgcgtggatacacgccgctcacgtaaaggcggcg
Xmv16        ccgtcctgctgaccacccccaccgctctcaaagtagacggcatctcCgcgtggatacacgccgctcacgtaaaggcggcg
Xmv17        ccgtcctgctgaccacccccaccgctctcaaagtagacggcatctctgcgtggatacacgccgctcacgtaaaggcggcg
Xmv18        ccgtcctgctgaccacccccaccgctctcaaagtagacggcatctctgcgtggatacacgccgctcacgtaaaggcggcg
Xmv19        ccgtcctgctgaccacccccaccgctctcaaagtagacggcatctctgcgtggatacacgccgctcacgtaaaggcggcg
Xmv41        ccgtcctgctgaccacccccaccgctctcaaagtTgacggcatctctgcgtggatacacgccgctcacgtaaaggcggcg
Xmv42        ccgtcctgctgaccTcccccaccgctctcaaagtagacggcatctctgcgtggatacacgccgctcacgtaaaggcggcg
Xmv43        ccgtcctgctgaccacccccaccgctctcaaagtTgacggcatctctgcgtggatacacgccgctcacgtaaaggcggcg
Xmv8         ccgtcctActgaccacccccaccgctctcaaagtagacggcatctctgcgtggatacacgccgctcacgtaaaggcggcg
Xmv9         ccgtcctActAaccacccccaccgctctcaaagtagacggcatctctgcgtggatacacgccgctcacgtaaaggcggcg
```

```
XMV_CONS      ACAACTCCTCCGGCCGGAACAGCATGGAAGGTCCAGCGTTCTCAAAATCCCTTAAAGATAAGATTAACCCGTGGGGCCCC
Xmv10         acaactcctccAgccggaacagca-----------------tcaaaaCcccttaaagataagattaacccgtggggcccc
Xmv12         acaactcctccggccggaacagcatggaaggtccagcgttctcaaaatcccttaaagataagattaacccgtggggcccc
Xmv13         acaactcctccAgccggaacagcatggaaggtccagcgttctcaaaaCcccttaaagataagattaacccgtggggcccc
Xmv15         acaactcctccggccggaacagcatggaaggtccagcgttctcaaaatcccttaaagataagattaacccgtggggcccc
Xmv16         acaactcctccAgccggaacagcatggaaggtccagcgttctcaaaaCcccttaaagataagattaacccgtggggcccc
Xmv17         acaactcctccggccggaacagca-----------------tcaaaatcccttaaagataagattaacccgtggggcccc
Xmv18         acaactcctccggccggaacagcatggaaggtccagcgttctcaaaatcccttaaagataagattaacccgtggggcccc
Xmv19         acaactcctccggccggaacagcatggaaggtccagcgttctcaaaatcccttaaagataagattaacccgtggggcccc
Xmv41         acaactcctccggccggaGcagcatggaaggtccagcgttctcaaaaCcccttaaagataagattaacccgtggggcccc
Xmv42         acaactcctccggccggaacagca-----------------tcaaaatcccttaaagataagattaacccgtggggcccc
Xmv43         acaactcctccggccggaGcagcatggaaggtccagcgttctcaaaaCcccttaaagataagattaacccgtggggcccc
Xmv8          acaactcctccggccggaacagcatggaaggtccagcgttctcaaaatcccttaaagataagattaacccgtggggcccc
Xmv9          acaactcctccggccggaacagcatggaaggtccagcgttctcaaaatcccttaaagataagaCtaacccgtggggcccc

XMV_CONS      CATGGAAGGTCCAGCGTTCTCAAAAHCCCTTAAAGATAAGATTAACCCGTGGGGCCCCCTVATAGTTATGGGGATCTTAG
Xmv10         catgAaaggtccagcgttctcaaaaCcccttaaagataagattaacccgtggggccccctAatagttatggggatcttGg
Xmv12         catggaaggtccagcgttctcaaaaTcccttaaagataagattaacccgtggggccccctGatagttatggggatcttag
Xmv13         catggaaggtccagcgttctcaaaaCcccttaaagataagattaacccgtggggccccctAatagttatggggatcttGg
Xmv15         catggaaggtccagcgttctcaaaaTcccttaaagataagattaacccgtggggccccctGatagttatggggatcttag
Xmv16         catggaaggtccagcgttctcaaaaCcccttaaagataagattaacccgtggggccccctAatagttatggggatcttGg
Xmv17         catggaaggtccagcgttctcaaaaTcccttaaagataagattaacccgtggggccccctGatagttatggggatcttag
Xmv18         catggaaggtccagcgttctcaaaaTcccttaaagataagattaacccgtggggccccctGatagttatggggatcttag
Xmv19         catggaaggtccagcgttctcaaaaTcccttaaagataagattaacccgtggggccccctGatagttatggggatcttag
Xmv41         catggaaggtccagcgttctcaaaaCcccttaaagataagattaacccgtggggccccctAatagttatggggatcttGg
Xmv42         catggaaggtccagcgttctcaaaaTcccttaaagataagattaacccgtggggccccctGatagttatggggatcttag
Xmv43         catggaaggtccagcgttctcaaaaCcccttaaagataagattaacccgtggggccccctAatagttatAgggatcttGg
Xmv8          catggaaggtccagcgttctcaaaaTcccttaaagataagattaacccgtggggccccctGatagttatggggatcttag
Xmv9          catggaaggtccagcgttctcaaaaTcccttaaagataagaCtaacccgtggggccccctGatagtCatggggatcttag

XMV_CONS      TGAGGGCAGGAGCCTCGGTACAACGTGACAGCCCTCACCAGGTCTTCAATGTTACTTGGAGAGTTACCAACCTAATGACA
Xmv10         tgagggcaggagcctcggtacaacgtgacagccctcaccagAtcttcaatgttacttggagagttaccaacctaatgaca
Xmv12         tgagggcaggagcctcggtacaacgtgacagccctcaccaggtcttcaatgttacttggagagttaccaacctaatgaca
Xmv13         tgagggcaggagcctcggtacaacgtgacagccctcaccagAtcttcaatgttacttggagagttaccaacctaatgaca
Xmv15         tgagggcaggagcctcggtacaacgtgacagccctcaccaggtcttcaatgttacttggagagttaccaacctaatgaca
Xmv16         tgagggcaggagcctcggtacaacgtgacagccctcaccagAtcttcaatgttacttggagagttaccaacctaatgaca
Xmv17         tgagggcaggagcctcggtacaacgtgacagccctcaccaggtcttcaatgttacttggagagttaccaacctaatgaca
Xmv18         tgagggcaggagcctcggtacaacgtgacagccctcaccaggtcttcaatgttacttggagagttaccaacctaatgaca
Xmv19         tgagggcaggagcctcggtacaacgtgacagccctcaccaggtcttcaatgttacttggagagttaccaacctaatgaca
Xmv41         tgagggcaggagcctcggtacaacgtgacagccctcaccaggtcttcaatgtCacttggagagttaccaacctaatgaca
Xmv42         tgagggcaggagcctcggtacaacgtgacagccctcaccaggtcttcaatgttacttggagagttaccaacctaatgaca
Xmv43         tgagggcaggagcctcggtacaacgtgacagccctcaccaggtcttcaatgtCacttggagagttaccaacctaatgaca
Xmv8          tgGgggcaggagcctcggGacaacgtgacagccctcaccaggtcttcaatgttacttggagagttaccaacctaatgaca
Xmv9          tgGgggcaggagcctcggGacaacgtgacagccctcaccaggtcttcaatgttacttggaAagttaccaacctaatgaca

XMV_CONS      GGACAAACAGCTAACGCCACCTCCCTCCTGGGGACGATGACAGACACCTTCCCTAAACTATATTTTGACCTGTGTGATTT
Xmv10         ggacaaacagctaacgccacctccctcctggggacgatgacTgacaccttccctaaactatattttgacctgtgtgattt
Xmv12         ggacaaacagctaacgccacctccctcctggggacgatgacagacaccttccctaaactatattttgacctgtgtgattt
Xmv13         ggacaaacagctaacgccacctccctcctggggacgatgacagacaccttccctaaactatattttgacctgtgtgattt
Xmv15         ggacaaacagctaacgccacctccctcctggggacgatgacagacaccttccctaaactatattttgacctgtgtgattt
Xmv16         ggacaaacagctaacgccacctccctcctggggacgatgacagacaccttccctaaactatattttgacctgtgtgattt
Xmv17         ggacaaacagctaacgccacctccctcctggggacgatgacagacaccttccctaaactatattttgacctgtgtgattt
Xmv18         ggacaaacagctaacgccacctccctcctggggacgatgacagacaccttccctaaactatattttgaTctgtgtgattt
Xmv19         ggacaaacagctaacgccacctccctcctggggacgatgacagacaccttccctaaactatattttgacctgtgtgattt
Xmv41         ggacaaacagctaacgcTacctccctcctggggacgatgacagacaccttccctaaactatattttgacTtgtgtgattt
Xmv42         ggacaaacagctaacgccacctccctcctggggacgatgacagacaccttccctaaactatattttgacctgtgtgattt
Xmv43         ggacaaacagctaacgcTacctccctcctggggacgatgacagacaccttccctaaactatattttgacTtgtgtgattt
Xmv8          ggacaaacagctaacgccacctccctcctggggacgatgacagacaccttccctaaactatattttgacctgtgtgattt
Xmv9          ggacaaacagctaacgccacctccctTctggggacgGtgacagacaccttccctaaactatattttgacctgtgtgattt
```

```
XMV_CONS    AGTAGGAGACTACTGG------GATGACCCAVMMM--GAT--DGGAGATGGTTGCCGCTCTCCCGGGGGAAGAAAAAGGA
Xmv10       agtaggagactactgg------gatgacccaGAACCCgatATTggGgatggttgccgcActcccgggggaagaaGaagga
Xmv12       agtaggagactactggGATTGGgatgaccca------gat---ggagatggttgccgctctcccgggggaagaaaaagga
Xmv13       agtaggagactactgg------gatgacccaGAACCCgatATTggGgatggttgccgcActcccgggggaagaaGaagga
Xmv15       agtaggagactactgg------gatgaccca------gat---ggagatggttgccgctctcccgggggaagaaaaagga
Xmv16       agtaggagactactgg------gatgacccaGAACCCgatATTggGgatggttgccgcActcccgggggaagaaGaagga
Xmv17       agtaggaAactactggGATTGGgatgaccca------gat---ggagatggttgccgctctcccgggggaagaaaaagga
Xmv18       agtaggagactactgg------gatgaccca------gat---ggagatggttgccgctctcccgggggaagaaaaagga
Xmv19       agtaggagactactggGATTGGgatgaccca------gat---ggagatggttgccgctctcccgggggaagaaaaagga
Xmv41       agtTggagacAactgg------gatgacccGGAACCCgatATTggagatggttgccgctctccGgggggaagaaaaagga
Xmv42       agtaggagaTtactgg------gatgaccca------gat---ggagatggttgccgctctcccgggAgaagaaaaagga
Xmv43       agtTggagacCaTtgg------gatgacccaGAACCCgatATTggagatggttgccgctctccGgggggaagaaaaagga
Xmv8        agtaggagactactgg------gaCgaccca------gat---ggagatggttgccgctctcccggAAgaagaaaaagga
Xmv9        agtaggagactactgg------gaCgaccca------gat---ggagTtggttgccgctctcccggAAgaagaaaaagga

XMV_CONS    CAAGACTGTATGACTTCTATGTTTGCCCCGGTCATACTGTACCAATAGGGTGTGGAGGGCCGGGAGAGGGCTACTGTGGC
Xmv10       caagactgtatgacttctatgtttgccccggtcatactgtaccaatagggtgtggagggccgggagagggctactgtggc
Xmv12       caagactgtatgacttctatgtttgccccggtcatactgtaccaatagggtgtggaggAccgggagagggctactgtggc
Xmv13       caagactgtatgacttctatgtttgccccggtcatactgtaccaatagggtgtggagggccgggagagggctactgtggc
Xmv15       caagactgtatgacttctatgtttgccccggtcatactgtaccaatagggtgtggagggccgggagagggctactgtggc
Xmv16       caagactgtatgacttctatgtttgccccggtcatactgtaccaatagggtgtggagggccgggagagggctactgtggc
Xmv17       caagactgtatgacttctatgtttgccccggtcatactgtaccaatagggtgtggagggccgggagagggctactgtggc
Xmv18       caagactgtatgacttctatgtttgccccggtcatactgtaccaatagggtgtggagggccgggagagggctactgtggc
Xmv19       caagactgtatgacttctatgtttgccccggtcatactgtaccaatagggtgtggagggccgggagagggctactgtggc
Xmv41       caagactAtatgaTttctatgtttgccccggCcatactgtGcTaaCagggtgtggagggccgAgagagggctactgCggc
Xmv42       caagactgtatgacttctatgtttgccccggtcatactgtaccaGtagggtgtggagggccgAgagagggctactgtggc
Xmv43       caagactgtatgacttctatgtttgccccggtcatactgtaccaatagggtgtggagggccgggagagggctactgtggc
Xmv8        caagactgtatgacttctatgtttgcTccggtcatactgtaccaatagggtgtggagggccgggagagggctactgtggc
Xmv9        caagactgtatgacttctatgtttgccccggtcatactgtaccaatagggtgtggagggccgggagagggctactgtggc

XMV_CONS    AAATGGGGATGTGAGACCACTGGACAGGCATACTGGAAGCCATCATCATCATGGGACCTAATTTCCCTTAAGCGAGGAAA
Xmv10       aaatggggatgtgaAaccactggacaggcatactggaagccatcatcatcatgggacctaatttcccttaagcgaggaaa
Xmv12       aaatggggatgtgagaccactggacaggcatactggaagccatcatcatcatgggacctaatttcccttaagcgaggaaa
Xmv13       aaatggggatgtgagaccactggacaggcatactggaagccatcatcatcatgggacctaatttcccttaagcgaggaaa
Xmv15       aaatggggatgtgagaccactggacaggcatactggaagccatcatcatcatgggacctaatttcccttaagcgaggaaa
Xmv16       aaatggggatgtgagaccactggacaggcatactggaagccatcatcatcatgggacctaatttcccttaagcgaggaaa
Xmv17       aaatggggatgtgagaccactggacaggcatactggaagccatcatcatcatgggacctaatttcccttaagcgaggaaa
Xmv18       aaatggggatgtgagaccactggacaggcatactggaagccatcatcatcatgggacctaatttcccttaagcgaggaaa
Xmv19       aaatggggatgtgagaccactggacaggcatactggaagccatcatcatcatgggacctaatttcccttaagcgaggaaa
Xmv41       aaatggggatgtgagaccactggacaggcatactggaagccatcatcatcatgggacctaatttcccttaagcgaggaaa
Xmv42       aaatggggatgtgagaccactggacaggcatactggaagccatcatcatcatgggacctaatttcccttaagcgaggaaa
Xmv43       aaatggggatgtgagaccactggacaggcatactggaagccatcatcatcatgggacctaatttcccttaagcgaggaaa
Xmv8        aaatggggatgtgagaccactggacaggcatactggaagccCtcatcatcatgggacctaatttcccttaagcgaggaaa
Xmv9        aaatggggatgtgagaccactggacaggcatactggaagccCtcatcatcatgggacctaatttcccttaagcgaggaaa

XMV_CONS    CACTCCTAAGGATCAGGGCCCCTGTTATGATTCCTC---GGTCTCCAGTGGCGTCCAGGGTGCCACACCGGGGGGTCGAT
Xmv10       cactcctaaggatcagggcccctgttatgattcctc---ggtctccagtggcgtccagggtgccacaccggggggtcgat
Xmv12       cactcctaaggatcagggcccctgttatgattcctc---ggtctccagtggcgtccagggtgccacaccggggggtcgat
Xmv13       cactcctaaggatcagggcccctgttatgattcctc---ggtctccagtggcgtccagggtgccacaccggggggtcgat
Xmv15       cactcctaaggatcagggcccctgttatgattcctc---ggtctccagtggcgtccagggtgccacaccggggggtcgat
Xmv16       cactcctaaggatcagggcccctgttatgattcctc---ggtctccagtggcgtccagggtgccacaccggggggtcgat
Xmv17       cactcctaaggatcagggcccctgttatgattcctc---ggtctccagtggcgtccagggtgccacaccggggggtcgat
Xmv18       cactcctaaggatcagggcccctgttatgattcctc---ggtctccagtggcgtccagggtgccacaccggggggtcgat
Xmv19       cactcctaaggatcagggcccctgttatgattcctc---ggtctccagtggcgtccagggtgccacaccggggggtcgat
Xmv41       cactcctaaggGtcagggcccctgttatgattcctcAGTggtctccagtAgcgtccagggGgccacaccggggggtcgat
Xmv42       cactcctaaggatcagggcccctgttatgattcctc---ggtctccagtgAcgtccagggtgccacaccggggggtcgat
Xmv43       cactcctaaggatcagggcccctgttatgattcctc---ggtctccagtggcgtccagggtgccacaccggggggtcgat
Xmv8        cactcctaaggatcagggcccctgttatgattcctc---ggtctccagtggcgtccagggtgcTacaccggggggtcgat
Xmv9        cactcctaaggatcagggcccctgttaCgattcctc---ggtctccagtggcgtccagggtgcTacaccggggggtcgat
```

```
XMV_CONS     GCAACCCCCTAGTCTTAGAATTCACTGACGCGGGTAAAAAGGCCAGCTGGGATGCCCCCAAAGTTTGGGGACTAAGACTC
Xmv10        gcaaccccctGgtcttagaattcactgacgcgggtaGaaaggccagctgggatgcccccaaagtttggggactaagactc
Xmv12        gcaaccccctagtcttagaattcactgacgcgggtaaaaaggccagctgggatgcccccaaagtttggggactaagactc
Xmv13        gcaaccccctGgtcttagaattcactgacgcgggtaGaaaggccagctgggatgcccccaaagtttggggactaagactc
Xmv15        gcaaccccctagtcttagaattcactgacgcgggtaaaaaggccagctgggatgcccccaaagtttggggactaagactc
Xmv16        gcaaccccctGgtcttagaattcactgacgcgggtaaaaaggccagctgggatgcccccaaagtttggggactGagactc
Xmv17        gcaaccccctagtcttagaattcactgacgcgggtaaaaaggccagctgggatgcccccaaagtttggggactaagactc
Xmv18        gcaaccccctagtcttagaattcactgacgcgggtaaaaaggccagctgggatgcccccaaagtttggggactaagactc
Xmv19        gcaaccccctagtcttagaattcactgacgcgggtaaaaaggccagctgggatgcccccaaagtttggggactaagactc
Xmv41        gcaaccccctagtcCtagaattcactgacgcAggtaaaaaggccagctgggatgcccccaaagtttggggactaagactc
Xmv42        gcaaccccctagtcttagaattcactgacgcgggtaaaaaggccagctgggatgcccccaaagtttggggactaagactc
Xmv43        gcaaccccctagtcttagaattcactgacgcgggtaaaaaggccagctgggatgcccccaaagtttggggactaagactc
Xmv8         gcaaccccctagtcttagaattcactgacgcgggtaaaaaggccagctgggatgcccccaaagtttggggactaagactc
Xmv9         gcaaccccctagtcttagaattcactgacgcgggtaaaaaggccagctgggatgcccccaaagtttggggactaagactc

XMV_CONS     TACCGATCCACAGGGGCCGACCCGGTGACCCGGTTCTCTTTGACCCGCCAGGTCCTCAATGTAGGACCCCGCGTCCCCAT
Xmv10        taTcgatccacaggggccgacccggtgacccggttctctttgacccgccaggtcctcaatgtaggaccccgcgtccccat
Xmv12        taccgatccacaggggccgacccggtgacccggttctctttgacccgccaggtcctcaatgtaggaccccgcgtccccat
Xmv13        taTcgatccacaggggccgacccggtgacccggttctctttgacccgccaggtcctcaatgtaggaccccgcgtccccat
Xmv15        taccgatccacaggggccgacccggtgacccggttctctttgacccgccGggtcctcaatgtaggaccccgcgtccccat
Xmv16        taTcgatccacaggggccgacccggtgacccggttctctttgacccgccaggtcctcaatgtaggaccccgcgtccccat
Xmv17        taccgatccacaggggccgacccggtgacccggttctctttgacccgccaggtcctcaatgtaggaccccgcgtccccat
Xmv18        taccgatccacaggggccgacccggtgacccggttctctttgacccgccaggtcctcaatgtaggaccccgcgtccccat
Xmv19        taccgatccacaggggccgacccggtgacccggttctctttgacccgccaggtcctcaatgtaggaccccgcgtccccat
Xmv41        taccgatccacaggggccgacccggtgacccggttctctttgacccgccaggtcctcaatgtaggaccccgcgtccccat
Xmv42        taTcgatccacaggggccgacccggtgacccggttctctttgacccgccaggtcctcaatgtaggaccccgcgCcccat
Xmv43        taccgatccacGggggccgacccggtgacccggttctctttgacccgccaggtcctcaatgtaggaccccgcgtccccat
Xmv8         taTcgatccacaAgggccgacccggtgacccggttctctttgacccgccaggtcctcaatgtaggaccccgcgtccccat
Xmv9         taTcgatccacaAgggccgacccggtgacccggttctctttgacccgccaggtcctcaatgtaggaccccgcgtccccat

XMV_CONS     TGGGCCTAATCCCGTGATCACTGACCAGCTACCCCCATCCCAACCCGTGCAGATCATGCTCCCCAGGCCTCCTCATCCTC
Xmv10        tgggcctaatcccgtgatcactgaccagctacccccatcccaacccgtgcagatcatgctccccaggcctcctcatcctc
Xmv12        tgggcctaatcccgtgatcactgaccagctacccccatcccaacccgtgcagatcatgctccccaggcctcctcatcctc
Xmv13        tgggcctaatcccgtgatcactgaccagctacccccatcccaacccgtgcagatcatgctccccaggcctcctcatcctc
Xmv15        tgggcctaatcccgtgatcactgaccagctacccccatcccaacccgtgcagatcatgctccccaggcctcctcatcctc
Xmv16        tgggcctaatcccgtgatcactgaccagctacccccatcccaacccgtgcagatcatgctccccaggcctcctcatcctc
Xmv17        tgggcctaatcccgtgatcactgaccagctacccccatcccaacccgtgcagatcatgctccccaggcctcctcatcctc
Xmv18        tgggcctaatcccgtgatcactgaccagctacccccatcccaacccgtgcagatcatgctccccaggcctcctcatcct-
Xmv19        tgggcctaatcccgtgatcactgaccagctacccccatcccaacccgtgcagatcatgctccccaggcctcctcatcctc
Xmv41        tgggcctaatcccgtgatcactgaccagctacccccatcccaacccgtgcagatcatgctccccaggcctcctcatcctc
Xmv42        tgggcctaatcccgtgatcGctgaccagctacccccatcccaacccgtgcagatcatgctccccaggcctcctcatcctc
Xmv43        tgggcctaatcccgtgatcactgaAcagctacccccCtcccaacccgtgcagatcatgctccccaggcctcctcatcctc
Xmv8         tgggcctaatcccgtgatcactgaccagctacccccatcccaacccgtgcagatcatgctccccaggcctcctcatcAtc
Xmv9         tgggcctaatcccgtgatcactgaccagctacccccatcccaacccgtgcagatcatgctccccaggcctcctcatcAtc

XMV_CONS     CTCCTTCAGGCACGGTCTCTATGGTACCTGGGGCTCCCCCGCCTTCTCAACAVCCTGGGACGGGAGACAGGCTGCTAAAT
Xmv10        ctccttcaggcacggtctctatggtacctggggctcccccgccttctcaacaAcctgggacgggagacaggctgctaaat
Xmv12        ctccttcaggcacggtctctatggtacctggggctcccccgccttctcaacaGcctgggacgggagacaggctgctaaat
Xmv13        ctccttcaggcacggtctctatggtacctggggctcccccgccttctcaacaAcctgggacgggagacaggctgctaaat
Xmv15        ctccttcaggcacggtctctatggtacctggggctcccccgccttctcaacaGcctgggacgggagacaggctgctaaat
Xmv16        ctccttcaggcacggtctctatggtacctggggctcccccgccttctcaacaAcctgggacgggagacaggctgctaaat
Xmv17        ctccttcaggcacggtctctatggtacctggggctcccccgccttctcaacaGcctgggacgggagacaggctgctaaat
Xmv18        --ccttcaggcacggtctctatggtacctggggctcccccgccttctcaacaGcctgggacgggagacaggctgctaaat
Xmv19        ctccttcaggcacggtctctatggtacctggggctcccccgccttctcaacaGcctgggacgggagacaggctgctaaat
Xmv41        ctccttcaggcacggtctctatggtGcctggggctcccccgccttctcaacaAcctgggacgggGgacaggctgctaaaC
Xmv42        ctccttcaggcacggtctctatAgtacctggggctcccccgccttctcaacaAcctgggacgggagacaggctgctaaat
Xmv43        ctccttcaggcGcggCctctatggtGcctggggctcccccgccttctcaacaAcctgggacgggGgacaggctgctaaaC
Xmv8         ctccttcaggcacggtctctatggtacctggggctcccccgccttctcaacaGcctgggacAggagacaggctActaaat
Xmv9         ctcctCcaggcacggtctctatggtacctggggctcccccgccttctcaacaGcctgggacAggagacaggctActaaGt
```

```
XMV_CONS     CTGGTAGANGGAGCCTACCAAGCACTCAACCTCACCAGTCCTGACAAAACCCAAGAGTGCTGGTTGTGTCTGGTATCGGG
Xmv10        ctggtagaAggagcctaccaagcactcaacctcaccagtcctgacaaaacccaagagtgctggttgtgtctggtatcggg
Xmv12        ctggtagaCggagcctaccaagcactcaacctcaccagtcctgacaaaacccaagagtgctggttgtgtctggtatcggg
Xmv13        ctggtagaAggagcctaccaagcactcaacctcaccagtcctgacaaaacccaagagtgctggttgtgtctggtatcggg
Xmv15        ctggtagaCggagcctaccaagcactcaacctcaccagtcctgacaaaacccaagagtgctggttgtgtctggtatcggg
Xmv16        ctggtagaAggagcctaccaagcactcaacctcaccagtcctgacaaaacccaagagtgctggttgtgtctggtatcggg
Xmv17        ctggtagaCggagcctaccaagcactcaacctcaccagtcctgacaaaacccaagagtgctggttgtgtctggtatcggg
Xmv18        ctAgtagaCggagcctaccaagcactcaacctcaccagtcctgacaaaacccaagagtgctggttgtgtctggtatcggg
Xmv19        ctggtagaCggagcctaccaagcactcaacctcaccagtcctgacaaaacccaagagtgctggttgtgtctggtatcggg
Xmv41        ctAgtagaAggagcctaccaagcactcaacctcaccagtccCgacaGaacccaagagtgctggCtgtgtctggtatcggg
Xmv42        ctggtaAaAggagcctaccaagcactcaacctcaccagtcctgacaaaacccaagagtgctggttAtgtctggtatcggg
Xmv43        ctAgtaAaAggagcctaTcaagcactcaacctcaccagtccCgacaGaacccaagagtgctggCtgtgtctggtatcggg
Xmv8         ctggtagaCggagcctaccaagcactcaacctcaccagtcctgacaaaacccaagagtgctggttgtgtctggtatcggg
Xmv9         ctggtagaCggagcctaccaagcactcaacctcaccagtcctgacaaaacTcaagagtgctggttgtgtctggtatcggg

XMV_CONS     ACCCCCCTACTACGAAGGGGTTGCCGTCCTAGGTACCTACTCCAACCATACCTCTGCCCCAGCTAACTGCTCCGTGGCCT
Xmv10        acccccctactacgaaggggttgccgtcctaggtacctactccaaccatacctctgccccagctaactgctccgtggcct
Xmv12        acccccctactacgaaggggttgccgtcctaggtacctactccaaccatacctctgccccagctaactgctccgtggcct
Xmv13        acccccctactacgaaggggttgccgtcctaggCacctactccaaccatacctctgccccagctaactgctccgtggcct
Xmv15        acccccctactacgaaggggttgccgtcctaggtacctactccaaccatacctctgccccagctaactgctccgtggcct
Xmv16        acccccctactacgaaggggttgccgtcctaggtacctactccaaccatacctctgccccagctaactgctccgtggcct
Xmv17        acccccctactacgaaggggttgccgtcctaggtacctactccaaccatacctctgccccagctaactgctccgtggcTt
Xmv18        acccccctactacgaaggggttgccgtcctaggtacctactccaaccatacctctgccccagctaactgctccgtggcct
Xmv19        acccccctactacgaaggggttgccgtcctaggtacctactccaaccatacctctgccccagctaactgctccgtggcct
Xmv41        acccccctactacgaaggggttgccgtcctaggtacctactccaaccatacctctgccccagctaactgctccgtggcct
Xmv42        acccccctactacgaaggggttgccgtcctaggtacctactccaaccatacctctgccccagctaactgctccgtggcct
Xmv43        acccccctactacgaaggggttgccgtcctaggtacctactccaaccatacctctgccccagctaactgctccgtggcct
Xmv8         acccccctactacgaaggggttgccgtcctaggtacctactccaaccatacctctgccccagctaactgctccgtggcct
Xmv9         acccccctactacgaaggggttgccgtcctaggtacctactccaaccatacctctgccccagctaactgctccgtggcct

XMV_CONS     CCCAACACAAGCTGACCCTGTCCGAAGTGACCGGACAGGGACTCTGCGTAGGAGCAGTTCCCAAAACCCATCAGGCCCTG
Xmv10        cccaacacaagctgaccctgtccgaagtgaccggacagggactctgcgtaggagcagttcccaaaacccatcaggccctg
Xmv12        cccaacacaagctgaccctgtccgaagtgaccggacagggactctgcgtaggagcagttcccaaaacccatcaggccctg
Xmv13        cccaacacaagctgaccctgtccgaagtAaccggacagggactctgcgtaggagcagttcccaaaacccatcaggccctg
Xmv15        cccaacacaagctgaccctgtccgaagtgaccggacagggactctgcgtaggagcagttcccaaaacccatcaggccctg
Xmv16        cccaacacaagctgaccctgtccgaagtgaccggacagggactctgcgtaggagcagttcccaaaacccatcaggccctg
Xmv17        cccaacacaagctgaccctgtccgaagtgaccggacagggactctgcgtaggagcagttcccaaaacccatcaggccctg
Xmv18        cccaacacaagctgaccctgtccgaagtgaccggacagggactctgcgtaggagcagttcccaaaacccatcaggccctg
Xmv19        cccaacacaagctgaccctgtccgaagtgaccggacagggactctgcgtaggagcagttcccaaaacccatcaggccctg
Xmv41        cccaacacaagctgaccctgtccgaagtgaccggacagggactctgcgtaggagcagttcccaaaacccatcaggccctg
Xmv42        cccaacacaagctgaccctgtccgaagtgaccggacagggactctgcgtaggagcagttcccaaaacccatcaggccctg
Xmv43        cccaacacaagctgaccctgtccgaagtgaccggGcagggactctgcgtaggagcagttcccaaaacccatcaggccctg
Xmv8         cccaacacaagctgaccctgtccgaagtgaccggacagggactctgcgtaggagcagttcccaaaacccatcaggccctg
Xmv9         cccaacacaagctgaccctgtccgaagtgaccggacagggactctgcgtaggagcagttcccaaaacccatcaggccctg

XMV_CONS     TGTAATACCACCCAGAAGACGAGCGACGGGTCCTACTATCTGGCTGCTCCCGCCGGGACCATCTGGGCTTGCAACACCGG
Xmv10        tgtaataccacccagaagacgagcgacgggtcctactatctggctgctcccgccgggaccatctgggcttgcaacaccgg
Xmv12        tgtaataccacccagaagacgagcgacgggtcctactatctggctgctcccgccgggaccatctgggcttgcaacaccgg
Xmv13        tgtaataccacccaAaagacgagcgacgggtcctactatctggctgctcccgccgggaccatctgggcttgcaacaccgg
Xmv15        tgtaataccacccagaagacgagcgacgggtcctactatctggctgctcccgccgggaccatctgggcttgcaacaccgg
Xmv16        tgtaataccacccagaagacgagcgacgggtcctactatctggctgctcccgccgggaccatctgggcttgcaacaccgg
Xmv17        tgtaataccacccagaagacgagcgacgggtcctactatctggctgctcccgccgggaccatctgggcttgcaacaccgg
Xmv18        tgtaataccacccagaagacgagcgacgggtcctactatctggctgctcccgccgggaccatctgggcttgcaacaccgg
Xmv19        tgtaataccacccagaagacgagcgacgggtcctactatctggctgctcccgccgggaccatctgggcttgcaacaccgg
Xmv41        tgtaataccacccaAaagacgagcgacgggtcctactatctggctgctcccgccgggaccatctgggcttgcaacaccgg
Xmv42        tgtaataccacccagaagacAagcgacgggtcctactatctggctgctcccgccgggaccatctgggcttgcaacaccgg
Xmv43        tgtaataccacccagaagGcgagcgacgggtcctactatctggctgctcccgccgggaccatctgggcttgcaacaccgg
Xmv8         tgtaataccacccagaagacgagcgacgggtcctactatctggctgctcccgccgggaccatctgggcttgcaacaccgg
Xmv9         tgtaataccacccagaagacgaAcgacgggtcctactatctggctgctcccgccgggaccatctgggcttgcaacaccgg
```

```
XMV_CONS    GCTCACTCCCTGCCTATCTACTACTGTACTCAACCTCACCACCGATTACTGTGTCCTGGTTGAGCTCTGGCCAAAGGTGA
Xmv10       gctcactccctgcctatctactactgtactcaacctcaccaccgattactgtgtcctggttgagctctggccaaaggtga
Xmv12       gctcactccctgcctatctactactgtactcaacctcaccaccgattactgtgtcctggttgagctctggccaaaggtga
Xmv13       gctcactccctgcctatctactactgtactcaacctcaccaccgattactgtgtcctggttgagctctggccaaaggtga
Xmv15       gctcactccctgcctatctactactgtactcaacctcaccaccgattactgtgtcctggttgagctctggccaaaggtga
Xmv16       gctcactccctgcctatctactactgtactcaacctcaccaccgattactgtgtcctggttgagctctggccaaaggtga
Xmv17       gctcactccctgcTtatctactactgtactcaacctcaccaccgattactgtgtcctggttgagctctggccaaaggtga
Xmv18       gctcactccctgcctatctactactgtactcaacctcaccaccgattactgtgtcctggttgagctctggccaaaggtga
Xmv19       gctcactccctgcctatctactactgtactcaacctcaccaccgattactgtgtcctggttgagctctggccaaaggtga
Xmv41       gctcactccctgcctatctacCactgtactcaacctcaccaccgattactgtgtcctggttgagctctggccaaaggtga
Xmv42       gctcactccctgcctatctactactgtactcaacctcaccaccgattactgtgtcctggttgaActctggccaaGggtga
Xmv43       gctcactccctgcctatctacCactgtactcaacctcaccaccgattactgtgtcctggttgagctctggccaaaggtga
Xmv8        gctcactccctgcctatctactactgtactcaacctcaccaccgattactgtgtcctggttgagctctggccaaaggtga
Xmv9        gctcactccctgcctatctactactgtactTaacctcaccaccgattactgtgtcctggttgagctctggccaaaggtga

XMV_CONS    CCTACCACTCCCCTGGTTATGTTTATGGCCAGTTTGAGAGAAAAACCAAATATAAAAGAGAGCCGGTGTCATTAACTCTG
Xmv10       cctaccactcccctggttatgtttatggccagtttgagaAaaaaaccaaatataaaagGgagccggtgtcattaactctg
Xmv12       cctaccactcccctggttatgtttatggccagtttgagagaaaaaccaaatataaaagagagccggtgtcattaactctg
Xmv13       cctaccactcccctggttatgtttatggccagtttgagagaaaaaccaaatataaaagagagccggtgtcattaactctg
Xmv15       cctaccactcccctggttatgtttatggccagtttgagagaaaaaccaaatataaaagagagccggtgtcattaactctg
Xmv16       cctaccactcccctggttatgtttatggccagtttgagagaaaaaccaaatataGaagGgagccggtgtcattaactctg
Xmv17       cctaccactcccctggttatgtttatggccagtttgagagaaaaaccaaatataTaagagagccggtgtcattaactctg
Xmv18       cctaccactcccctggttatgtttatggccagtttgagagaaaaaccaaatataTaagagagccggtgtcattaactctg
Xmv19       cctaccactcccctggttatgtttatggccagtttgagagaaaaaccaaatataaaagagagccggtgtcattaactctg
Xmv41       cctaccactcccctggttatgtttatggccagtttgagagaaaaaccaaatataaaagagagccggtgtcattaactctg
Xmv42       cctaccactcccctggttatgtttatggccagtttgagagaaaaaccaaatataaaagagagccggtgtcattaactctg
Xmv43       cctaccactcccctggttatgtttatgAccagtttgagagaaaaaccaaatataaaagagagccggtgtcattaactctg
Xmv8        cctaccactcccctggttatgtttatggccagtttgagagaaaaaccaaatataaaagGgagccggtgtcattaactctg
Xmv9        cctaccactcccctggttatgtGtatggccagtttgagagaaaaaccaaGtatCaaagagagccggtgtcattaactctg

XMV_CONS    GCCCTGCTGTTGGGAGGACTTACTATGGGCGGCATAGCTGCAGGAGTAGGAACAGGGACTACAGCCCTAGTGGCCACCAA
Xmv10       gccctgctgttgggaggacttactatgggcggcatagctgcaggagtaggaacagggactacagccctagtggccaccaa
Xmv12       gccctgctgttgggaggacttactatgggcggcatagctgcaggagtaggaacagggactacagccctagtggccaccaa
Xmv13       gccctgctgttAggaggacttactatgggcggcatagctgcaggagtaggaacagggactacagccctagtggccaccaa
Xmv15       gccctgctgttgggaggacttactatgggcggcatagctgcaggagtaggaacagggactacagccctagtggccaccaa
Xmv16       gccctgctgttgggaggacttactatgggcggcatagctgcaggagtaggaacagggactacagccctagtggccaccaa
Xmv17       gccctgctgttgggaggacttactatgggcggcatagctgcaggagtaggaacagggactacagccctagtggccaccaa
Xmv18       gccctgctgttgggaggacttactatgggcggcatagctgcaggagtaggaacagggactacagccctagtggccaccaa
Xmv19       gccctgctgttgggaggacttactatgggcggcatagctgcaggagtaggaacagggactacagccctagtggccaccaa
Xmv41       gccctgctgttgggaggacttactatgggcggcatagctgcaggagtagAaacagggactacagccctagtggccaccaa
Xmv42       gccctgctgttgggaggacttactatgggcggcatagctgcaggagtaggaacagggactacagccctGgtggccaccaa
Xmv43       gccctgctgttgggaggacttactatgggcggcatagctgcaggagtaggaacagggactacagccctagtggccaccaa
Xmv8        gccctgctgttgggaggacttactatgggcggcatagctgcaggagtaggaacagggactacagccctagtggccaccaa
Xmv9        gccctActgttgggaggacttactatgggcggcatagctgcaggagtaggaacagggactacagccctagtggccaccaa

XMV_CONS    ACAATTCGAGCAGCTCCAGGCAGCCATACATACAGACCTTGGGGCCTTAGAAAAATCAGTCAGTGCCCTAGAAAAGTCTC
Xmv10       acaattcgagcagctccaggcagccatacatacagaccttggggccttagaaaaatcagtcagtgccctagaaaagtctc
Xmv12       acaattcgagcagctccaggcagccatacatacagaccttggggccttagaaaaatcagtcagtgccctagaaaagtctc
Xmv13       acaattcgagcaActccaggcagccatacatacagaccttggggccttagaaaaatcagtcagtgccctagaaaagtctc
Xmv15       acaattcgagcagctccaggcagccatacatacagaccttggggccttagaaaaatcagtcagtgccctagaaaagtctc
Xmv16       acaattcgagcagctccaggcagccatacatacagaccttggggccttagaaaaatcagtcagtgccctagaaaagtctc
Xmv17       acaattcgagcagctccaggcagccatacatacagaccttggggccttagaaaaatcagtcagtgccctagaaaagtctc
Xmv18       acaattcgagcagctccaggcagccatacatacagaccttggggccttagaaaaatcagtcagtgccctagaaaagtctc
Xmv19       acaattcgagcagctccaggcagccatacatacagaccttggggccttagaaaaatcagtcagtgccctagaaaagtctc
Xmv41       acaattcgagcagctccaggcagccatacatacagaccttggggccttagaaaaatcagtcagtgccctagaaaagtctc
Xmv42       acaattcgagcagctccaggcagccatacatacagaccttggggccttagaaaaatcagtcagtgccctagaaaagtctc
Xmv43       acaattcgagcagctccaggcagccatacatacagaccttggggccttagaaaaatcagtcagtgccctagaaaagtctc
Xmv8        acaattcgagcagctccaggcagccatacatacagaccttggggccttagaaaaatcagtcagtgccctagaaaagtctc
Xmv9        acaattcgagcagctccaggcagccatacatacagaccttggggccttagaaaaatcagtcagtgccctagaaaagtctc
```

```
XMV_CONS    TGACCTCGTTGTCTGAGGTGGTCCTACAGAACCGGAGAGGATTAGATCTGCTGTTCCTAAAAGAAGGAGGATTATGTGCT
Xmv10       tgacctcgttgtctgaggtggtcctacagaaccggagaggattagatctgctgttcctaaaagaGggaggattatgtgct
Xmv12       tgacctcgttgtctgaggtggtcctacagaaccggagaggattagatctgctgttcctaaaagaaggaggattatgtgct
Xmv13       tgacctcgttgtctgaggtggtcctacagaaccggagaggattagatctgctgttcctaaaagaaggaggattatgtgct
Xmv15       tgacctcgttgtctgaggtggtcctacagaaccggagaggattagatctgctgttcctaaaagaaggaggattatgtgct
Xmv16       tgacctcgttgtctgaggtggtcctacagaaccggagaggattagatctgctgttcctaaaagaaggaggattatgtgct
Xmv17       tgacctcgttgtctgaggtggtcctacagaaccggagaggattagatctgctgttcctaaaagaaggaggattatgtgct
Xmv18       tgacctcgttgtctgaggtggtcctacagaaccggagaggattagatctgctgttcctaaaagaaggaggattatgtgct
Xmv19       tgacctcgttgtctgaggtggtcctacagaaccggagaggattagatctgctgttcctaaaagaaggaggattatgtgct
Xmv41       tgacctcgttgtctgaAgtggtcctacagaaccggagaggattagatctgctgttcctaaaagaaggaggattatgtgct
Xmv42       tgacctcgttgtctgaggtggtcctacagaaccggagaggattagatctgctgttcctaaaagaaggaggattatgtgct
Xmv43       tgacctcgttgtctgaggtggtcctacagaaccggagaggattagatctgctgttcctaaaagaaggaggattatgtgct
Xmv8        tgacctcgttgtctgaggtggtcctacagaaccggagaggattagatctgctgttcctaaaagaaggaggattatgtgct
Xmv9        tgacctcgttgtctgaAgtggtcctacagaaccggagaggattagatctgctgttcctaaaagaaggaggattatgtgct

XMV_CONS    GCCCTAAAAGAAGAATGCTGTTTCTACGCGGACCACACTGGCGTAGTAAGGGATAGCATGGCTAAGCTAAGAGAAAGGCT
Xmv10       gccctaaaagaagaatgctgtttctacgcggaccacactggcgtagtaagggatagcatggctaagctaagagaGagAct
Xmv12       gccctaaaagaagaatgctgtttctacgcggaccacactggcgtagtGagAgatagcatggcAaagctaagagaaaggTt
Xmv13       gccctaaaagaagaatgctgtttctacgcggaccacactggcgtagtaagggatagcatggctaagctaagagaGagAct
Xmv15       gccctaaaagaagaatgctgtttctacgcggaccacactggcgtagtGagAgatagcatggcAaagctaagagaaaggTt
Xmv16       gccctaaaagaagaatgctgtttctacgcggaccacactggcgtagtaagggatagcatggctaagctaagagaGagAct
Xmv17       gccctaaaagaagaatgctgtttctacgcggaccacactggcgtagtaagAgatagcatggcAaagctaagagaaaggTt
Xmv18       gccctaaaagaagaatgctgtttctacgcggaccacactggcgtagtGagAgatagcatggcAaagctaagagaaaggTt
Xmv19       gccctaaaagaagaatgctgtttctacgcggaccacactggcgtagtGagAgatagcatggcAaagctaagagaaaggTt
Xmv41       gcTctaaaagaagaatgctgtttctaTgcAgaccacactggcgtagtaagggatagcatggctaagctGagagaaaggct
Xmv42       gccctaaaagaagaatgctgtttctacgcggaccacactggcgtagtGagAgatagcatggcAaagctGagagaaaggTt
Xmv43       gccctaaaagaagaatgctgtttctaTgcAgaccacactggcgtagtaagggatagcatggctaagctaagagaaaggct
Xmv8        gccctaaaagaagaatgctgtttctacgcggaccacactggcgtagtaagggatagcatggctaagctaagagaGagAct
Xmv9        gccctaaaagaagaatgctgtttctacgcggaccacactggcgtagtaagggatagcatggctaagctaagagaGagAct

XMV_CONS    AAACCAGAGACAAAAATTGTTCGAATCAGGACAAGGGTGGTTTGAGGGACTGTTTAACAGGTCCCCATGGTTCACGACCC
Xmv10       aaaccagagacaaaaattgttcgaatcaggacaagggtggtttgagggactgtttaacaggtccccatggttcacgaccc
Xmv12       aaaccagagacaaaaattgttcgaatcaggacaagggtggtttgagggactgtttaacaggtccccatggttcacgaccT
Xmv13       aaaccagagacaaaaattgttcgaatcaggacaagggtggtttgagggactgtttaacaggtccccatggttcacgaccc
Xmv15       aaaccagagacaaaaattgttcgaatcaggacaagggtggtttgagggactgtttaacaggtccccatggttcacgaccT
Xmv16       aaaccagagacaaaaattgttcgaatcaggacaagggtggtttgagggactgtttaacaggtccccatggttcacgaccc
Xmv17       aaaccagagacaaaaattgttcgaatcaggacaagggtggtttgagggactgtttaacaggtccccatggttcacgaccT
Xmv18       aaaccagagacaaaaattgttcgaatcaggacaagggtggtttgagggactgtttaacaggtccccatggttcacgaccT
Xmv19       aaaccagagacaaaaattgttcgaatcaggacaagggtggtttgagggactgtttaacaggtccccatggttcacgaccT
Xmv41       aaaccagagacaaaaattgttcgaatcaggacaagggtggtttgagggactgtttaacaggtccccatggttcacgaccc
Xmv42       aaaccagagacaaaaattgttcgaatcaggacaagggtggttCgagggactgtttaacaggtccccatggttcacgaccT
Xmv43       aaaccagagGcaaaaattgttcgaatcaggacaagggtggtttgagggactgtttaacaggtccccatggttcacgaccc
Xmv8        aaaccagagacaaaaattgttcgaatcaggacaagggtggtttgagggactgtttaacaggtccccatggttcacgaccc
Xmv9        aaaccagagacaaaaattgttcgaatcaggacaagggtggtttgagggactgtttaacaggtccccatggttcacgaccT

XMV_CONS    TGATATCCACCATTATGGGCCCBCTGATAGTACTTTTATTAATCCTACTCCTCGGACCCTGTATTCTCAACCGCTTGGTC
Xmv10       tgatatccaccattatgggcccTctgatagtacttttattaatcctactcTtcggaccctgtattctcaaccgcttggtc
Xmv12       tgatatccaccattatgggcccCTtgataAtacttttattaatcctactcctcggaccctgtattctcaaccgcttggtc
Xmv13       tgaCatccaccattatgggcccTctgatagtacttttattaatcctactcTtcggaccctgtattctcaaccgcttggtc
Xmv15       tgatatccaccattatgggcccCTtgataAtacttttattaatcctactcctcggaccctgtattctcaaccgcttggtc
Xmv16       tgatatccaccattatgggcccTctgatagtacttttattaatcctactcTtcggaccctgtattctcaaccgcttggtc
Xmv17       tAatatccaccattatgggcccCTtgataAtacttttattaatcctactcctcggaccctgtattctcaaccgcttggtc
Xmv18       tgatatccaccattatgggcccCTtgataAtacttttattaatcctactcctcggaccctgtattctcaaccgcttggtc
Xmv19       tgatatccaccattatgggcccCTtgataAtacttttattaatcctactcctcggaccctgtattctcaaccgcttggtc
Xmv41       tgatatccaccattatgggcccTctgatagtacttttattaatcctactcctcggaccctgCattctcaaccgcttggtc
Xmv42       tgatatccaccattatgggcccCTtgataAtacttttattaatcctactcTtcggaccctgtattctcaaccgcttggtc
Xmv43       tgatatccaccattatgggcccTctgatagtacttttattaatcctactcctcggaccctgCattctcaaccgcttggtc
Xmv8        tgatatccaccattatgggcccTctgatagtacttttattaatcctactcTtcggaccctgtattctcaaccgcttggtc
Xmv9        tgatatccaccattatgggcccCTtgataAtacttttattaatcctactcctcggaccctgtattctcaaccgAttggtc
```

```
XMV_CONS      CAGTTTGTAAAAGACAGAATTTCGGTGGTG
Xmv10         cagtttgtaaaagacagaatttcggtggtg
Xmv12         cagtttgtaaaagacagaatttcggtggtg
Xmv13         cagtttgtaaaagacagaatttcggtggtg
Xmv15         cagtttgtaaaagacagaatttcggtggtg
Xmv16         cagtttgtaaaagacagaatttcggtggtg
Xmv17         cagtttgtaaaagacagaatttcggtggtg
Xmv18         cagtttgtaaaagacagaatttcggtggtg
Xmv19         cagtttgtaaaagacagaatttcggtggtg
Xmv41         cagtttgtaaaagacagaatttcggtggtg
Xmv42         cagtttgtaaaagacagaatttcggtggtg
Xmv43         cagtttgtaaaagacagaatttcggtggtg
Xmv8          cagtttgtaaaagacagaatttcggtggtg
Xmv9          cagtttgtaaaagacagaatttcggtggtg
```
