# Supplementary figures and images for: Role of APOBEC3 in Genetic Diversity among Endogenous Murine Leukemia Viruses
Source: PLoS Genet. 2007 Oct 26;3(10):e183. doi: 10.1371/journal.pgen.0030183 (PMC2041998; doi:10.1371/journal.pgen.0030183)

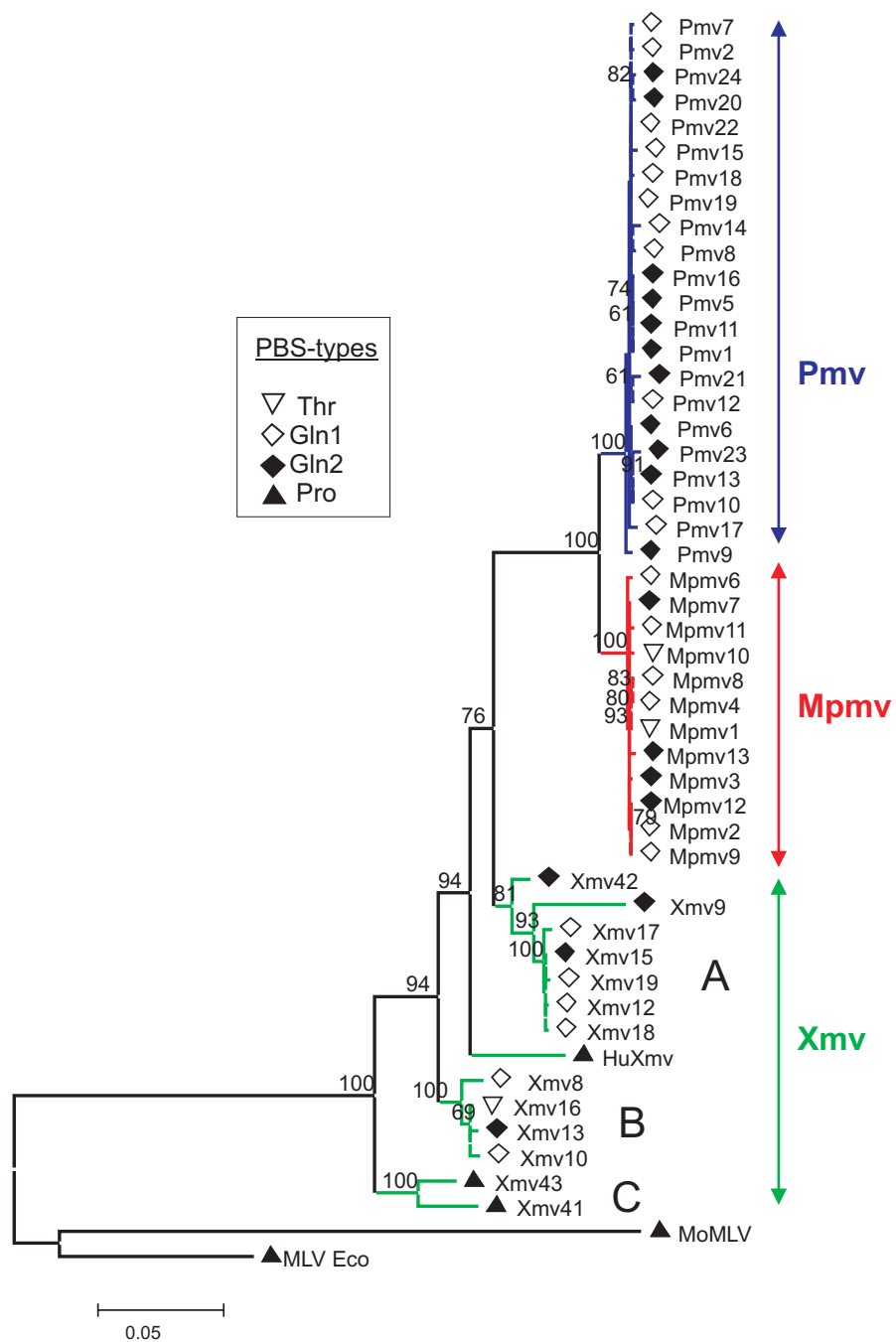

Figure S4

Supplement: Figure S4 — (19 KB PDF) [file pgen.0030183.sg004.pdf]

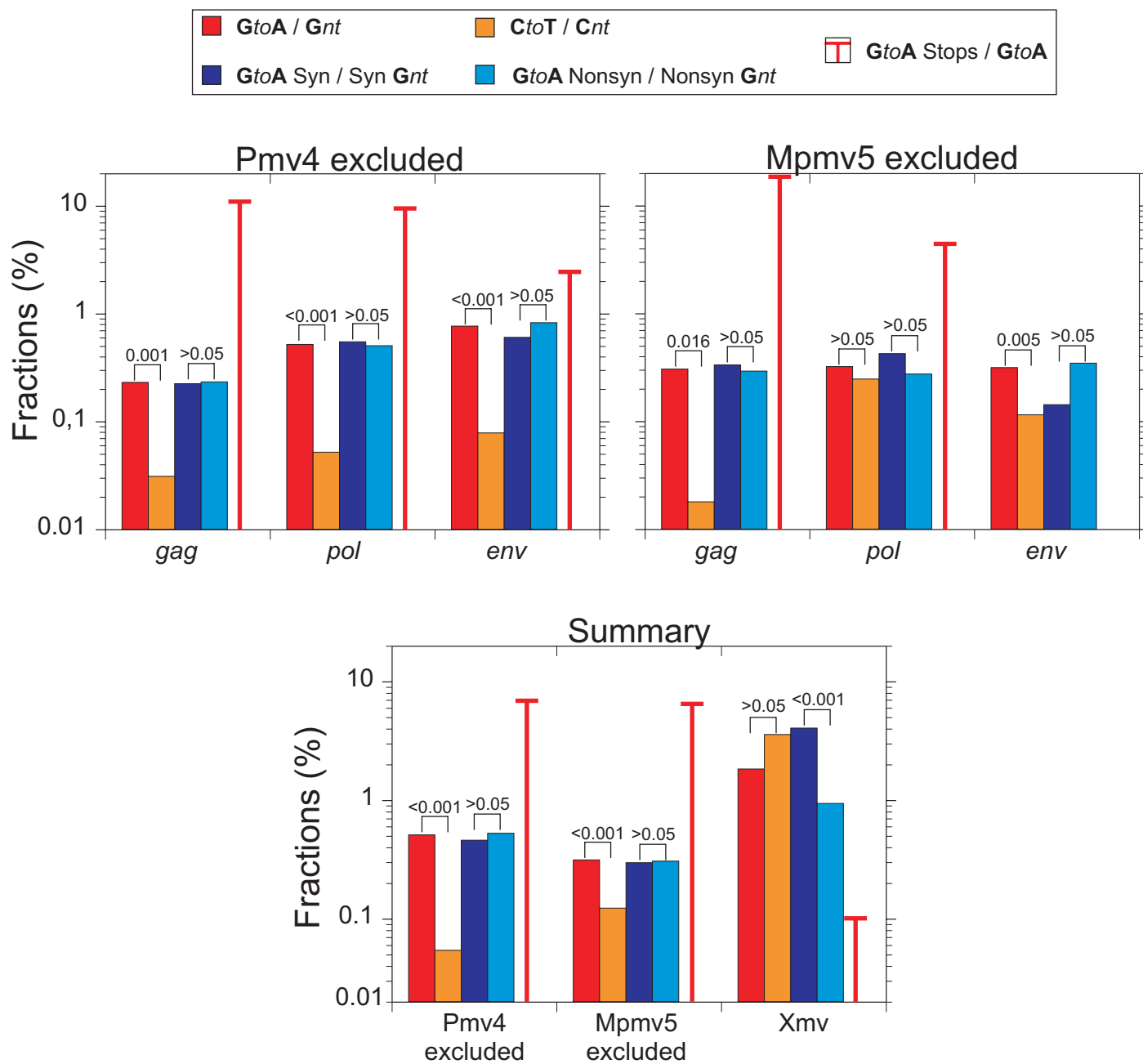

Figure S5

Supplement: Figure S5 — (20 KB PDF) [file pgen.0030183.sg005.pdf]

Pmv4 excluded

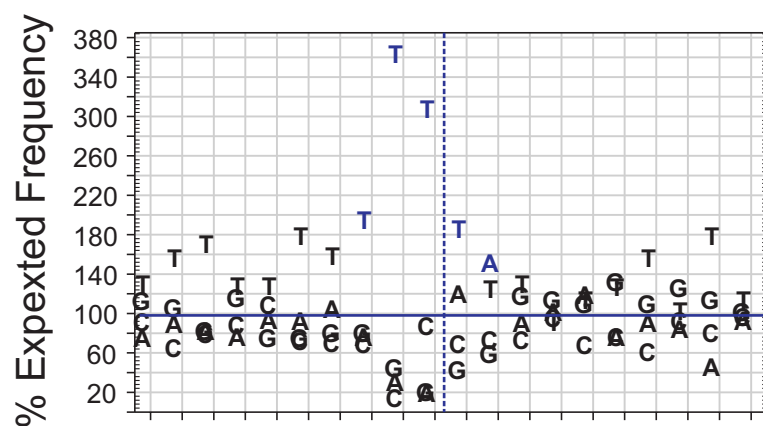

Mpmv5 excluded

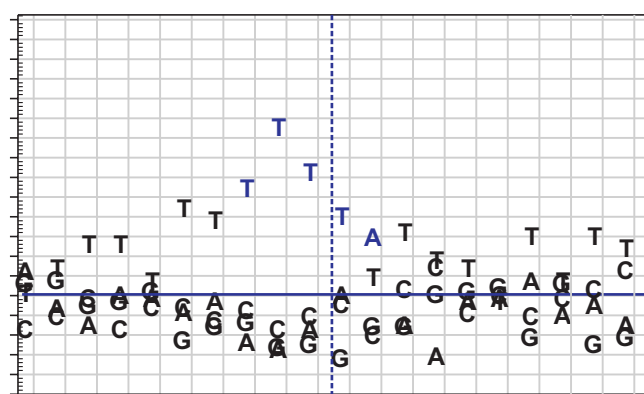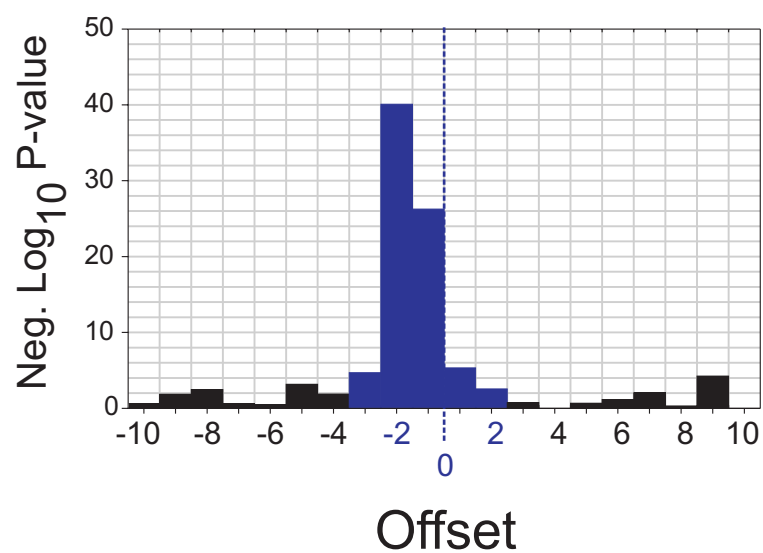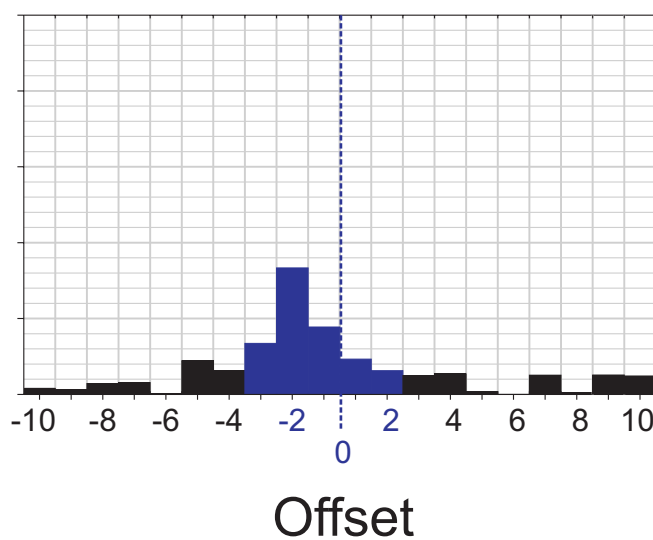

Figure S6

Supplement: Figure S6 — (22 KB PDF) [file pgen.0030183.sg006.pdf]

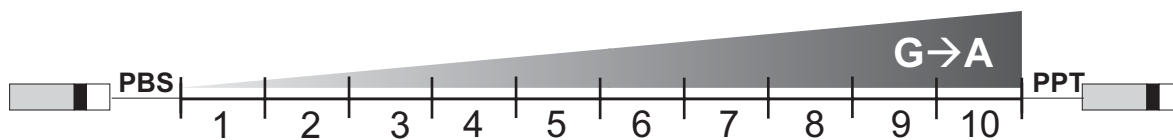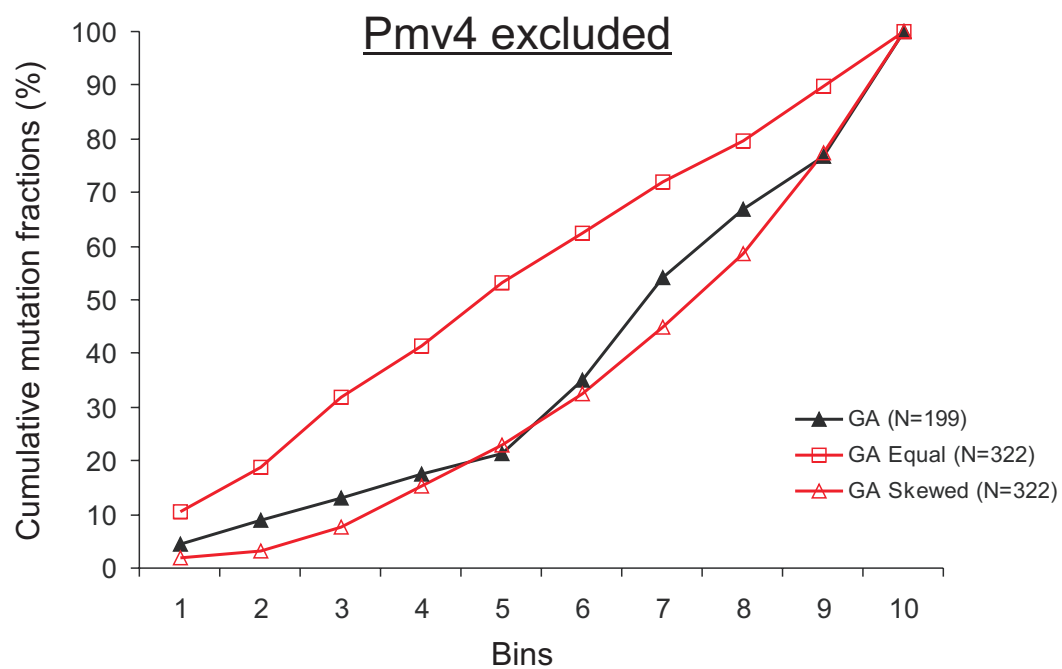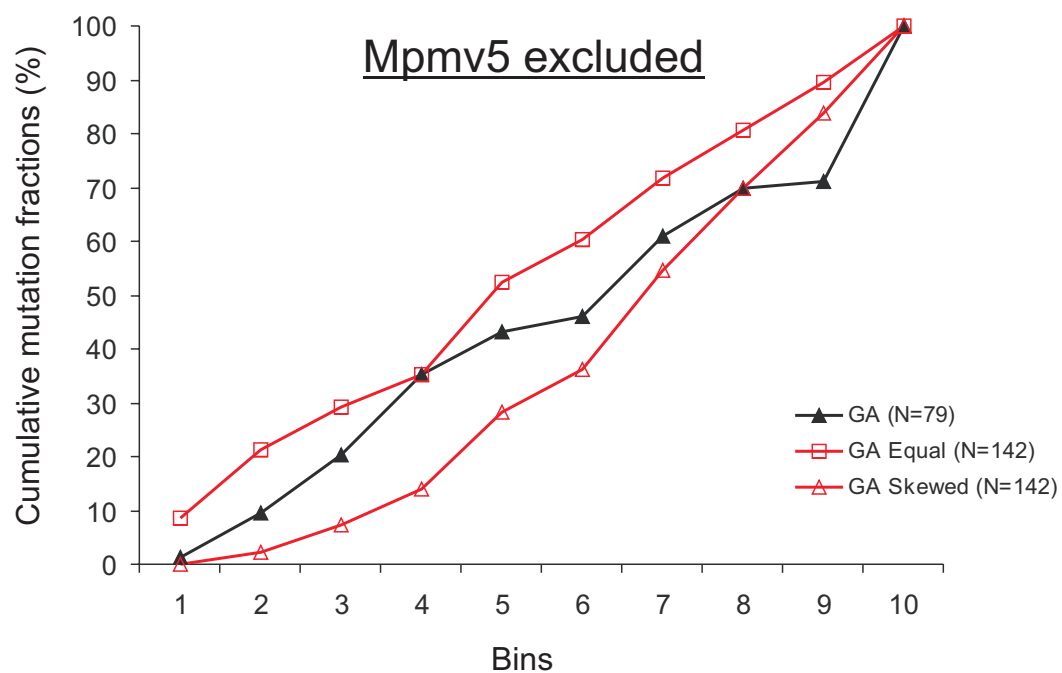

Figure S7

Supplement: Figure S7 — (21 KB PDF) [file pgen.0030183.sg007.pdf]
