# Supplementary material for: Role of APOBEC3 in Genetic Diversity among Endogenous Murine Leukemia Viruses
Source: PLoS Genet. 2007 Oct 26;3(10):e183. doi: 10.1371/journal.pgen.0030183 (PMC2041998; doi:10.1371/journal.pgen.0030183)
Supplement: Table S1 — (12 KB PDF) [file pgen.0030183.st001.pdf]

Table S1. Nonecotropic endogenous MLVs

		BLAT mouse (freeze Feb. 2006)				LTR lengths		LTR diff.		Gag[a]		Pol[a]		Env[a]		
Locus	Syn. name	Chr.	Start	Orientation	Length	5'LTR	3'LTR	(%)	PBS	stops	f.s.[b]	stops	f.s.[b]	stops	f.s.[b]	ORFs
Pmv1	Xa	chrX	14631006	plus	8981	697	697	0	Gln2	0	0	1	0	0	0	
Pmv10		chr8	122775542	minus	7476	697	697	0	Gln1	0	0	0	0	0	0	X
Pmv11	5c	chr5	78005290	plus	8982	697	697	0	Gln2	0	0	0	0	0	0	X
Pmv12	5b	chr5	144923365	plus	8981	697	697	0	Gln1	1	0	0	0	0	0	
Pmv13		chr10	41125063	plus	8981	697	697	0	Gln2	0	0	0	0	0	0	X
Pmv14	16b	chr16	76026704	minus	8982	697	697	0	Gln1	0	0	1	0	1	0	
Pmv15	7a	chr7	30397399	plus	8981	697	697	0	Gln1	0	0	1	0	0	0	
Pmv16	16a	chr16	93594977	minus	8981	697	697	0	Gln2	1	0	0	0	0	0	
Pmv17	15a	chr15	76394257	minus	7186	697	697	0	Gln1	0	0	nd[c]	nd[c]	0	0	
Pmv18	7b	chr7	29323966	plus	8699	697	697	0	Gln1	0	0	0	0	0	0	X
Pmv19	4b	chr4	107652948	minus	8983	698	698	0.14	Gln1	0	0	0	0	0	0	X
Pmv2	11a	chr11	6658394	minus	8970	697	697	0	Gln1	0	0	1	1	0	0	
Pmv20	18a	chr18	82827978	plus	8880	697	697	0	Gln2	0	1	0	0	0	0	
Pmv21	1b	chr1	193634227	plus	8981	697	697	0	Gln2	0	0	2	0	1	0	
Pmv22	11b	chr11	8820300	plus	8980	697	697	0	Gln1	0	0	0	0	0	0	X
Pmv23	4a	chr4	101370070	minus	8982	697	697	0.14	Gln2	0	0	1	0	0	0	
Pmv24	1a	chr1	184093794	plus	8981	697	697	0	Gln2	0	0	1	0	0	0	
Pmv4	7c	chr7	6682978	minus	8982	697	697	0.29	Gln2	0	1	7	0	5	0	
Pmv5	5a	chr5	44422607	plus	8982	697	697	0	Gln2	0	1	0	0	0	0	
Pmv6	Xb	chrX	51501606	plus	8981	697	697	0	Gln2	0	0	0	0	0	0	X
Pmv7	2a	chr2	57038778	minus	8981	697	697	0	Gln1	0	0	0	0	0	0	X
Pmv8	10a	chr10	50115990	minus	8027	682	683	0.15	Gln1	0	0	1	1	1	0	
Pmv9	13a	chr13	100095412	minus	8981	697	697	0	Gln2	0	0	1	0	0	0	
Mpmv1	7a	chr7	64005511	plus	9041	741	741	0	Thr	0	0	0	0	0	0	X
Mpmv10	3a	chr3	67184006	plus	7717	742	742	0	Thr	0	0	1	1	0	1	
Mpmv11	12a	chr12	70465410	plus	9041	741	741	0	Gln1	1	0	0	0	0	0	
Mpmv12	10b	chr10	22423693	plus	9045	743	743	0	Gln2	0	0	0	0	0	0	X
Mpmv13	5a	chr5	24740763	plus	8972	741	741	0	Gln2	0	0	1	0	0	0	
Mpmv2	11c	chr11	76365002	plus	9045	743	743	0	Gln1	0	0	0	0	0	0	X
Mpmv3	2a	chr2	15947290	minus	9042	741	741	0	Gln2	0	1	0	0	0	0	
Mpmv4	11b	chr11	86698510	plus	9041	741	741	0	Gln2	0	0	0	0	0	0	X
Mpmv5	10a	chr10	4628826	minus	9039	741	741	0.54	Gln2	0	1	3	0	2	0	
Mpmv6	1a	chr1	133470112	plus	9054	748	746	0.27	Gln1	1	0	0	0	0	0	
Mpmv7	5b	chr5	43496188	plus	9041	741	741	0.13	Gln2	0	0	0	0	0	0	X
Mpmv8	11a	chr11	102899752	plus	9042	741	741	0.13	Gln1	1	0	1	0	0	0	
Mpmv9	3b	chr3	152260525	plus	9043	742	742	0	Gln1	0	0	0	0	0	0	X
Xmv10	2a	chr2	156055046	minus	7050	502	502	0	Gln1	0	0	nd[c]	nd[c]	0	0	
Xmv12	8a	chr8	44819280	minus	8734	573	573	0.17	Gln1	0	0	0	0	0	0	X
Xmv13	13a	chr13	68392677	minus	8687	547	547	0	Gln2	0	0	0	0	0	0	X
Xmv15	9a	chr9	62237179	plus	8759	574	613	6.36	Gln2	0	0	0	0	0	0	X
Xmv16	9b	chr9	41663263	minus	6784	574	574	0	(Thr)	nd[c]	nd[c]	0	0	0	0	
Xmv17	5a	chr5	23725372	plus	6846	535	535	0	Gln1	0	0	nd[c]	nd[c]	0	0	
Xmv18	19a	chr19	60988696	minus	8727	574	574	0	Gln1	0	0	0	0	0	0	X
Xmv19	14a	chr14	53484569	plus	7824	574	574	0.17	Gln1	nd[c]	nd[c]	0	0	0	0	
Xmv41	1a	chr1	173317820	plus	8670	531	531	0	Pro	0	0	2	0	0	0	
Xmv42	11a	chr11	88713056	minus	6787	697	697	0	Gln2	0	0	nd[c]	nd[c]	0	0	
Xmv43	1b	chr1	172778195	plus	8657	525	525	0	Pro	0	0	0	0	0	0	X
Xmv8	4b	chr4	145371740	minus	8759	588	588	0	Gln1	0	0	0	0	0	0	X
Xmv9	4a	chr4	146062627	plus	8722	576	576	0	Gln2	0	0	0	0	0	0	X

[a] Automated predictions using RetroTector Program (Sperber G.O., Airola T., Jern P. And J. Blomberg. *Nucleic Acids Res. In press 2007*)

[b] f.s. Frame shifts

[c] Not determined (complicated by deletions.)
