# Supplementary material for: Role of APOBEC3 in Genetic Diversity among Endogenous Murine Leukemia Viruses
Source: PLoS Genet. 2007 Oct 26;3(10):e183. doi: 10.1371/journal.pgen.0030183 (PMC2041998; doi:10.1371/journal.pgen.0030183)
Supplement: Table S2 — (7 KB PDF) [file pgen.0030183.st002.pdf]

Table S2. PBS types in Nonecotropic

Provirus	PBS-type	Sequence
**Gln1**	**Gln1**	**TGGAGGTTCCACCGAGAT**
Mpmv11	Gln1	TGGAGGTTCCACCGAGAT
Mpmv2	Gln1	TGGAGGTTCCACCGAGAT
Mpmv6	Gln1	TGGAGGTTCCACCGAGAT
Mpmv8	Gln1	TGGAGGTTCCACCGAGAT
Mpmv9	Gln1	TGGAGGTTCCACCGAGAT
Pmv10	Gln1	TGGAGGTTCCACCGAGAT
Pmv12	Gln1	TGGAGGTTCCACCGAGAT
Pmv14	Gln1	TGGAGGTTCCACCGAGAT
Pmv15	Gln1	TGGAGGTTCCACCGAGAT
Pmv17	Gln1	TGGAGGTTCCACCGAGAT
Pmv18	Gln1	TGGAGGTTCCACCGAGAT
Pmv19	Gln1	TGGAGGTTCCACCGAGAT
Pmv2	Gln1	TGGAGGTTCCACCGAGAT
Pmv22	Gln1	TGGAGGTTCCACCGAGAT
Pmv7	Gln1	TGGAGGTTCCACCGAGAT
Pmv8	Gln1	TGGAGGTTCCACCGAGAT
Xmv10	Gln1	TGGAGGTTCCACCGAGAT
Xmv12	Gln1	TGGAGGTTCCACCGAGAT
Xmv17	Gln1	TGGAGGTTCCACCGAGAT
Xmv18	Gln1	TGGAGGTTCCACCGAGAT
Xmv19	Gln1	TGGAGGTTCCACCGAGAT
Xmv8	Gln1	TGGAGGTTCCACCGAGAT
**Gln2**	**Gln2**	**TGGAGGTCCCACCGAGAT**
Mpmv12	Gln2	TGGAGGTCCCACCGAGAT
Mpmv13	Gln2	TGGAGGTCCCACCGAGAT
Mpmv3	Gln2	TGGAGGTCCCACCGAGAT
Mpmv4	Gln2	TGGAGGTCCCACCGAGAT
Mpmv5	Gln2	TGGAGGTCCCACCGAGAT
Mpmv7	Gln2	TGGAGGTCCCACCGAGAT
Pmv1	Gln2	TGGAGGTCCCACCGAGAT
Pmv11	Gln2	TGGAGGTCCCACCGAGAT
Pmv13	Gln2	TGGAGGTCCCACCGAGAT
Pmv16	Gln2	TGGAGGTCCCACCGAGAT
Pmv20	Gln2	TGGAGGTCCCACCGAGAT
Pmv21	Gln2	TGGAGGTCCCACCGAGAT
Pmv23	Gln2	TGGAGGTCCCACCGAGAT
Pmv24	Gln2	TGGAGGTCCCACCGAGAT
Pmv4	Gln2	TGGAGGTCCCACCGAGAT
Pmv5	Gln2	TGGAGGTCCCACCGAGAT
Pmv6	Gln2	TGGAGGTCCCACCGAGAT
Pmv9	Gln2	TGGAGGTCCCACCGAGAT
Xmv13	Gln2	TGGAGGTCCCACCGAGAT
Xmv15	Gln2	TGGAGGTCCCACCGAGAT
Xmv42	Gln2	TGGAGGTCCCACCGAGAT
Xmv9	Gln2	TGGAGGTCCCACCGAGAT
**Pro1&2**	**Pro**	**TGGGGGCTCGTCCGGGAT**
Xmv41	Pro	TGGGGGCTCGTCCGGGAT
Xmv43	Pro	TGGGGGCTCGTCCGGGAT
HuXmv	Pro	TGGGGGCTCGTCCGGGAT
MLV_Eco	Pro	TGGGGGCTCGTCCGGGAT
MoMLV	Pro	TGGGGGCTCGTCCGGGAT
**Thr**	**Thr**	**TGGAGGCCCCAGCGAGAT**
Mpmv1	Thr	TGGAGGCCCCAGCGAGAT
Mpmv10	Thr	TGGAGGCCCCAGCGAGAT
Xmv16	Thr	TGGAGGCCCCAGCGAGAT
